# What are the beneficial treatment strategies in maintaining T lymphocyte subsets after cancer surgery? A systematic review and network meta-analysis

**DOI:** 10.3389/fimmu.2026.1854279

**Published:** 2026-07-14

**Authors:** Ziang Xu, Yikun Zhang, Yanyu Huo, Zihua Xu, Chenze Wang, Chengkang Wang, Yingshi Zhang, Qingchun Zhao

**Affiliations:** 1 Department of Clinical Pharmacy, Shenyang Pharmaceutical University, Shenyang, Liaoning, China; 2 Department of Pharmacy, General Hospital of Northern Theater Command, Shenyang, Liaoning, China

**Keywords:** cancer, CD4, CD8, surgery, T cell

## Abstract

**Background:**

Perioperative alterations in T lymphocyte subsets significantly impact tumor recurrence and postoperative recovery; therefore, identifying optimal strategies to maintain these immune parameters is essential for improving cancer surgery outcomes. The immunomodulatory effects of different tumor therapies, especially those on key T cell subsets, still lack systematic comparisons.

**Objective:**

This study aims to systematically evaluate the impact of various treatment strategies (including systemic anti-cancer therapy (SACT), nutritional support, anesthesia, analgesia, and other methods) on key immune parameters (CD3^+^, CD4^+^, CD8^+^ cells, CD4^+^/CD8^+^ ratio, NK cells) in patients with solid tumors and provide a comparative ranking of their efficacy.

**Methods:**

Systematic reviews and network meta-analyses were conducted on randomized controlled trials included in the PubMed, Embase and Cochrane CENTRAL databases using the PRISMA guidelines. Pairwise meta-analyses and network meta-analyses were completed using RevMan 5.3 and the gemtc package of R software. The efficacy was ranked by the area under the cumulative ranking curve (SUCRA), and the quality of the studies was evaluated using the Cochrane Risk of Bias Tool 2.0 and the Newnews-Ottawa Scale.

**Results:**

The analysis included 82 RCTs. In maintaining T lymphocyte subsets, immune-enhanced enteral nutrition combined with neoadjuvant chemotherapy (IEN+NACT), targeted therapy (TD), the Enhanced Recovery After Surgery (ERAS) protocol, anesthesia techniques incorporating Transcutaneous Electrical Acupoint Stimulation (TEAS), and analgesia with Dexmedetomidine (DEX) ranked as the most effective strategies, with IEN+NACT showing the greatest improvement in CD4+ counts (SMD 12.33, 95% CI 7.44-17.04). Safety analysis indicated that the risks associated with these top-ranking interventions were acceptable.

**Conclusion:**

This network meta-analysis determined that for tumor surgery patients, the perioperative management plan should combine a multimodal strategy of IEN + NACT, ERAS, TD, TEAS and DEX to maintain postoperative T lymphocyte homeostasis and thereby protect immune function.

**Systematic Review Registration:**

https://www.crd.york.ac.uk/PROSPERO/view/CRD420251233455, identifier CRD420251233455.

## 1 Introduction

Cancer is one of the leading causes of death worldwide (1). Its pathogenesis is a dynamic process which is closely related to the interaction between tumors and the host’s immune system (2). The surgery itself can damage the immune system, and this postoperative immunosuppression may promote tumor metastasis and recurrence. Therefore, it is crucial to provide immune protection for tumor patients undergoing surgery. The response to many anti-cancer therapies including chemotherapy, targeted therapy and immunotherapy is markedly affected by the patients’ immunologic status (3). In clinical practice, T lymphocyte subsets have become quantifiable and practical indicators of immune status. CD3^+^, CD4^+^, CD8^+^ T cells, and CD4^+^/CD8^+^ ratio are markers of stable immune status, and we need to find the most effective way to stabilize these markers (4). It limits the ability to individualize treatment and preserve the patients’ immune function which is essential for survival and quality of life (5).

Accumulating evidence shows that peripheral T-cell subsets, especially CD4^+^ and CD8^+^ T lymphocytes, are closely linked to immune status (6). CD4^+^ T-helper cells and CD8^+^ cytotoxic T-cells play vital roles in adaptive and antitumor immunity, respectively (7, 8). However, their postoperative depletion and a decreased CD4^+^/CD8^+^ ratio are associated with worse clinical outcomes, including increased treatment toxicity and reduced survival in solid cancers (9, 10). Therefore, quantitatively preserving or restoring these T-cell populations is crucial, as it reflects stable immune function (11). For patients, maintaining robust T-lymphocyte levels is a key desired outcome, enabling the immune system to effectively combat both malignancy and infections (12).

All of the previous studies examined the immunologic impact of different therapies one by one (13). However, there is a lack of evidence on the comparative effectiveness of different treatment modalities on the immunologic parameters. Very few previous study synthesized the direct and indirect evidence from randomized controlled trials (14). The aim of this study is to rank the effects of different strategies, including Systemic anti-cancer therapies (SACT), nutritional support, anesthesia, analgesia and etc. methods, on the data of CD3^+^, CD4^+^ and CD8^+^ cells, and CD4^+^/CD8^+^ ratio in patients with tumors through head-to-head and network meta-analysis. We aim to determine the most comprehensive strategy that meets the requirement of immunology to be incorporated into cancer care, which may further influence clinical practice and clinical outcomes (15).

## 2 Methods

The review was reported following the Preferred Reporting Items for Systematic Reviews and Meta-Analyses (PRISMA) which offers a detailed check list for transparently describing the treatment comparisons process. To reduce the risk of research duplication and reporting bias, an *a priori* study protocol was retrospectively registered with the International Prospective Register of Systematic Reviews (PROSPERO) before commencement of formal literature screening (16). The PROSPERO registration number is NO(CRD420251233455).

### 2.1 .Data sources and search strategies

A systematic literature search was conducted across multiple electronic databases. This search includes PubMed, Embase and the Cochrane Central Register of Controlled Trials (CENTRAL) in the analysis. The study also examines the ClinicalTrials.gov registry that provides data for the analysis. The search was conducted up to June 20, 2026. The treatment types included therapy using radiation, therapy using chemical agents, procedures involving operation, therapy targeting molecules, and disease returning after treatment. The search combined these treatment terms with terms for specific cell types. The cell types included the key T lymphocyte subsets that are crucial to maintain stability after surgery—Specifically for CD3^+^ T cells, CD4^+^ T cells, CD8^+^ T cells, and NK cells. The detailed search queries for each concept are provided in the **Supplementary Material** (**Supplementary Table 1**). No language restrictions were applied during the search.

The study selection process was carried out independently by two experienced reviewers (XZA and ZYK) to minimize bias. Initially, all identified records were collated, and duplicates were excluded. The remaining records were then screened based on their titles and abstracts. The full texts of potentially eligible studies were retrieved and assessed against the predefined inclusion and exclusion criteria. Any disagreements between the two reviewers at any stage of the selection process were resolved through discussion or by consultation with a third senior reviewer (ZQC and ZYS).

### 2.2 .Data abstraction and assessment of risk of bias

Two reviewers (XZA and ZYK) independently extracted data from each included study using a standardized form, with disagreements resolved by consensus or adjudication by a third reviewer (ZQC and ZYS). Extracted information encompassed baseline characteristics (e.g., trial name, cancer type, treatment details, sample size, population age, treatment duration) and outcome measures. For multi-arm trials, each treatment arm was recorded separately to facilitate analysis.

The primary outcomes were changes in key immune cell populations: absolute counts or percentages of CD3^+^, CD4^+^, and CD8^+^ T lymphocytes, the CD4^+^/CD8^+^ ratio, and NK cells. Data were extracted at multiple time points where available, including pre-treatment, during treatment, end of treatment, and post-treatment follow-up, to comprehensively assess temporal dynamics. For missing outcome data, we first attempted to contact the original authors; if unavailable, multiple imputation methods were applied. Two reviewers assessed the quality of studies included in the analysis. The assessment used different approaches for different study types. The risk of bias in RCTs was assessed using the Cochrane Risk of Bias Tool 2.0 (RoB 2) (17). Studies that used non-randomized or retrospective observational studies using the Newcastle-Ottawa Scale (18). The assessment was conducted independently by two reviewers. This dual-independent assessment ensured consistency in methodological evaluation. For the Cochrane RoB 2.0 tool, a predominance of ‘low risk’ judgments across domains signified lower bias and higher study quality. Conversely, higher scores on the Newcastle-Ottawa Scale (NOS) indicated superior quality in non-randomized studies. Additionally, we applied the GRADE framework to evaluate the certainty of evidence from head-to-head meta-analyses (19). While randomized trials initially confer high certainty, this rating may be downgraded due to study limitations, inconsistency, indirectness, imprecision, or publication bias.

### 2.3 PICOS framework

Population (P): Adult patients (≥18 years) with histologically confirmed cancer undergoing surgical resection.

Intervention (I): Any perioperative intervention.

Comparison (C): Placebo, no intervention, standard care, or alternative active interventions.

Outcome (O): Primary outcomes: CD3+, CD4+, CD8+ T cell percentage, CD4+/CD8+ ratio, and NK cell counts; secondary outcome: safety (adverse events).

Study design (S): Randomized controlled trials (RCTs) and non-randomized controlled studies.

### 2.4 Outcomes

This meta-analysis combining studies examined outcomes chosen in advance to evaluate how combination treatment affects patients after surgically treated cancer, with the aim of identifying the most effective comprehensive strategies to stabilize T lymphocyte subsets. The main findings focused on immune function, particularly the mean changes from initial measure in key cell types in blood—specifically T cells and cells providing natural defense against infection (NK cells). These measures, including total counts or percentages of different T cell forms (CD3^+^, CD4^+^, CD8^+^), the relationship between CD4^+^ and CD8^+^ cells, and NK cells, show direct indicators of how the immune system responds and rebuilds itself. For these measures of the system responding to disease, values that increase indicate outcomes that support the function of this system (20). The identification of these high-priority interventions was determined through our network meta-analysis, which provided a hierarchical ranking of all treatments based on their efficacy in modulating T-cell subsets and NK cell populations. Safety analysis was prioritized for the most effective interventions identified in each treatment category. These important safety outcomes were categorized by system organ classes and included gastrointestinal disorders, infection, skin disorders, abnormal blood pressure, nervous system disorders, and a composite of other disorders. This comprehensive approach ensured that our safety assessment would provide clinically meaningful data to inform the risk-benefit profile of the most efficacious immune-modulating interventions in the surgical oncology setting.

### 2.5 Data synthesis and statistical analysis

We employed a comprehensive statistical framework that integrated both conventional head-to-head meta-analysis and Bayesian network meta-analysis (NMA) methodologies to thoroughly evaluate the comparative efficacy and safety profiles of various therapeutic interventions in surgically treated patients with cancer. The analysis of continuous outcomes-particularly the changes in critical immune parameters including CD3^+^ T cells, CD4^+^ T cells, CD8^+^ T cells, CD4^+^/CD8^+^ ratio, and NK cells - was performed using either Mean Difference (MD) or Standardized Mean Difference (SMD) with 95% confidence intervals. For dichotomous safety outcomes, we synthesized data using Mantel-Haenszel odds ratios (ORs) with 95% CIS. Each head-to-head comparison was accompanied by comprehensive statistical reporting that included: precise effect estimates (MD, SMD, or OR) with 95% CIs; explicit specification of the statistical model employed (primary analysis used random-effects models, with fixed-effect models for sensitivity analysis); detailed heterogeneity measures (*P-*value and *I²* statistic),along with interpretation; systematic risk of bias assessment based on Cochrane RoB 2.0 criteria; and GRADE certainty ratings evaluated through the GRADEpro GDT framework (19). Our NMA employed an advanced Bayesian framework using R software version 4.5.1 with the ‘gemtc’ package, implementing a multivariate random-effects model under consistency assumptions. Treatment ranking was performed using surface under the cumulative ranking curve (SUCRA) probabilities and median ranks, with uncertainty characterized through rankograms (21).

The safety analysis incorporated a systematic evaluation of adverse events across multiple organ systems, with particular focus on gastrointestinal disorders, infections, skin manifestations, hemodynamic instability, neurological complications, and other treatment-emergent adverse events. For each safety domain, we calculated pooled ORs with 95% CIs using random-effects models, complemented by comprehensive heterogeneity quantification and GRADE certainty assessments. We employed comparison-adjusted funnel plots to evaluate small-study effects across the treatment networks. Statistical analyses were performed using Review Manager (RevMan) version 5.3 for head-to-head comparisons.

## 3 Result

### 3.1 Study selection, study characteristics and risk of bias

**Figure 1:**
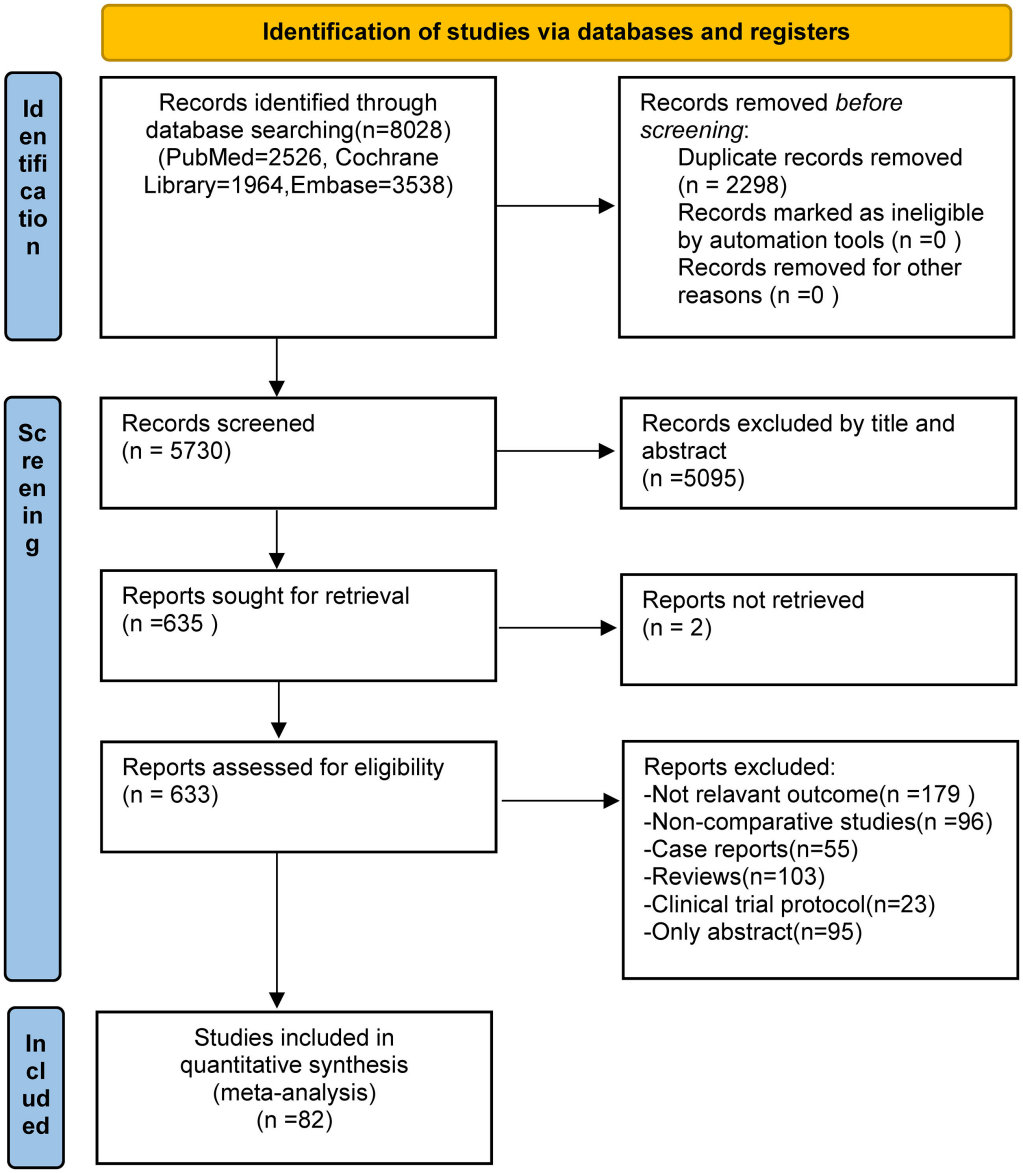
PRISMA flow diagram for a summary of the search process.

The initial database search yielded a total of 8,028 records. Following removal of 2,298 records that appeared more than one time, 5,730 different records remained for examination of the main features. The complete text of the remaining 633 studies received review in detail. Of these, 551 studies were excluded after full-text review, leaving 82 studies that met all eligibility criteria for inclusion in the final analysis. The process of finding studies and making selections appears in the diagram showing the flow of this process (**Figure 1**). **Table 1** provides a complete summary of the features of these studies. These studies examined different approaches to treatment that received organization into nine main categories for analysis: Surgical & Technical Modalities (e.g., VATS, LN), Nutrition Support Modalities (e.g., EN, PN, IEN), Anesthesia Techniques (e.g., TIVA, regional blocks), Analgesia Protocols (e.g., Dexmedetomidine, various opioids), Systemic Anti-Cancer Therapies (SACT) (e.g., TACE, targeted therapy, immunotherapy), Traditional Chinese Medicine (TCM), Psychotherapy, Enhanced Recovery After Surgery (ERAS) protocols, and others. Sample sizes across included studies varied considerably, ranging from approximately 32 to 400 participants (**Supplementary Table 2**). Enrolled patients represented a spectrum of common solid tumors, with ages predominantly falling within the adult to older adult range. Immune parameter assessments were conducted over varying timeframes, from several days to multiple months following intervention. Methodological quality of the RCTs was generally acceptable. For non-randomized studies, most achieved moderate to high scores on the NOS, reflecting satisfactory rigor in participant selection, comparability, and outcome assessment. The detailed results of the risk of bias assessment for all individual studies are provided in the **Supplementary Material** (**Supplementary Table 4**; **Supplementary Figure 4**).

**Table 1:** Characteristics of the included studies.

Treatment strategies	Author	Year	Type of cancer	Sex	Age	Intervention		Duration	Outcomes
						I	C		
Surgical & Technical Modalities	Zhang, L.	2016	NSCLC	57/23	60.4 ± 9.2	VATS	TOS	/	CD4+/CD8+/NK
	Zhao, G.	2005	CRC	34/26	57.1 ± 12.5	LN	TOS	/	CD3+/CD4+/CD8+/CD4/CD8
	Peng, B.	2008	KC	29/23	50.67 ± 15.46	LN	TOS	/	CD3+/CD4+/CD8+
	Han, L.	2018	NSCLC	110/48	61.42 ± 5.02	VATS	TOS	/	CD4+/CD8+
	Zhang, L. B.	2015	NSCLC	66/56	58.0 ± 5.7	VATS	TOS	/	CD3+/CD4+/CD8+/CD4/CD8
	Lv, W.	2022	BC	F	51.66 ± 10.38	ES	TOS	/	CD4+/CD8+
	Zhao, J.	2022	LC	99/21	64.19 ± 12.68	VATS+TP	VATS	42d	CD4+/CD8+/CD4/CD8/NK
	Zhou,Y	2017	EC	46/29	59.3 ± 5.3	FLB+VATS	TOS	/	CD3+/CD4+/CD8+/CD4/CD8
							C1:VATS		
	Shao, Y. J.	2018	GC	61/27	53.3± 4.9	LN+DZO+ROP	LN+FEN+ROP	/	CD3+/CD4+/CD8+/NK
	Gao, Y.	2021	EC	67/51	45.85 ± 16.3	TLS+RT	TLS+PF	4w	CD3+/CD4+/CD8+
	Liu, M.	2022	CC	F	44.21 ± 6.18	LN+NACT	TOS+NACT	6-9w	CD3+/CD4+/CD8+/CD4/CD8
Nutrition Support Modalities	Li, K.	2009	CRC	318/302	62.58	EN	EN+PN	7d	CD3+/CD4+/CD4/CD8
	Mi, L.	2012	GC	33/27	57.2 ± 9.5	EN	EN+PN	7d	CD3+/CD4+/CD8+/CD4/CD8/NK
	Ding, D.	2015	GC	70/22	42-75	EN Before	TPN	16d	CD4+/CD8+/CD4/CD8
	Marano, L.	2013	GC	71/38	65.85	IEN	EN	7d	CD4+
	Liu, H.	2012	GC	42/36	59.3	IEN	EN	7d	CD4+/CD8+
	Huang, X.	2010	GC	16/24	65.6 ± 8.3	IEN	EN	8d	CD3+/CD4+/CD8+/CD4/CD8
	Liu, Z.	2011	GC	31/11	65.35 ± 7.35	IEN	EN	8d	CD3+/CD4+/CD8+/CD4/CD8
	Chen, H.	2006	GC	30/16	64.7 ± 10.2	EEN	PN	7d	CD3+/CD4+/CD8+/CD4/CD8
	Wang, H. X.	2011	GC	82/44	60.1	EN Before	EEN	16d	CD4+/CD8+/CD4/CD8
	Zhao, H.	2013	GC	40/33	58	IEN	EN	7d	CD4+/CD8+
	Li, B.	2015	GC	216/184	60.8 ± 5.9	EEN	TPN	7d	CD3+/CD4+/CD8+/CD4/CD8
	Sorensen, D.	2009	HNSC	M	46-73	IEN	EN	8d	CD3+/CD4+/CD8+/CD4/CD8
	Liang, B.	2008	CRC	25/16	55.8 ± 10.1	ITPN	TPN	7d	CD3+/CD4+/CD8+/CD4/CD8
	Ma, B. Q.	2020	CHOL	33/23	59.08 ± 7.64	EEN + PN	TPN	7d	CD3+/CD4+/CD8+/CD4/CD8
	Tang, J.	2019	EC	62/44	48.2 ± 9.1	EN + PN	EN	14d	CD3+/CD4+/CD4/CD8
	Yang,J	2022	GC	82/38	57.8 ± 10.5	EN + IPN	EN + PN	5d	CD3+/CD4+/CD8+/CD4/CD8
	Ding, H.	2020	LC	51/49	58.47 ± 6.25	ERAS	EN + PN	7d	CD3+/CD4+/CD4/CD8
	Liu, H.	2011	GC	57/27	70.2	IEN	EN	7d	CD4+/CD8+/CD4/CD8
						I1:TPN			
	Zong, L.	2019	GC	58/38	39-70	IEN+NACT	NACT	14d	CD4+/CD8+/CD4/CD8
	Cai, J.	2008	GC	/	/	EN+TCM	TPN	9d	CD3+/CD4+/CD4/CD8
						I1:EN			
	Gu, R. M.	2012	GC	48/17	58	HIIC + EN	EN	7d	CD4+/CD8+/CD4/CD8/NK
						I1:HIIC + PN			
Anesthesia	Wang, M. Q.	2018	HCC	24/16	57.05 ± 3.45	NIV+IV	IV	/	CD4+/CD8+/CD4/CD8
	Xing, R.	2022	GC	63/26	69.38	TEAS + TAPB	IV	/	CD3+/CD4+/CD8+/CD4/CD8
						I1: TAPB			
	Woo, J. H.	2015	BC	F	50.45 ± 9.37	PPF	STP	/	CD4+/CD8+/CD4/CD8
	Xin, L.	2019	GC	138/62	59.84 ± 5.67	NIV+IV	IV	/	CD3+/CD4+/CD8+/CD4/CD8
	Zhu, J.	2017	GBC	86/58	47.01 ± 6.31	NIV+IV	IV	/	CD3+/CD4+/CD8+/CD4/CD8
	Zhu, R.	2020	EC	114/6	61.25	IV+PNIV	IV+PIV	/	CD3+/CD4+/CD8+/CD4/CD8
						I1:IV+NIV+PIV	I2:IV+NIV+PNIV		
	Zhang, T.	2014	TC	43/17	58.5	TIVA	MIXED	/	CD3+/CD4+/CD8+/CD4/CD8
						I1:SEVO			
	Liu, S.	2016	CC	F	48.31 ± 9.78	TIVA	SEVO	/	CD3+/CD4+/CD8+/CD4/CD8
	Wang, L.	2019	GC	21/19	59.3 ± 4.8	IV	NIV+IV	/	CD3+/CD4+/CD8+/CD4/CD8
Analgesia	Lin, Y.	2019	LC	33/36	55.46	Naloxone+Fentanyl	Fentanyl	48h	CD3+/CD4+/CD8+/CD4/CD8
	Zong, S.	2021	LC	37/23	39-56	Dexmedetomidine+Flurbiprofen	Flurbiprofen	/	CD3+/CD4/CD8
	Ai, Z.	2023	GC	65/39	65-75	Dexmedetomidine	Fentanyl	/	CD3+/CD4+/CD8+/CD4/CD8
	Wang, K.	2018	CRC	69/72	42.5 ± 4.3	Dexmedetomidine	N	/	CD3+/CD4+/CD8+/CD4/CD8
	Bai, Y.	2020	BC	F	34-63	Dexmedetomidine	Oxycodone	/	CD3+/CD4+/CD8+/CD4/CD8/NK
						I1:Combination			
	Chen, M.	2022	NSCLC	31/29	40-64	Parecoxib	N	12h	CD3+/CD4+/CD8+/NK
	Gao, Y. F.	2014	GC	29/11	40-70	Dezocine+Flurbiprofen	Fentanyl	48h	CD3+/CD4+/CD8+/CD4/CD8/NK
	Zhou,Y	2017	EC	46/29	59.3 ± 5.3	FLB+VATS	TOS	/	CD3+/CD4+/CD8+/CD4/CD8
							C1:VATS		
	Li, W. K.	2008	Osteosarcoma	21/11	52	Fentanyl	Morphine	/	CD3+/CD4+/CD8+/CD4/CD8
	Shen, J. C.	2014	GC	31/28	58 ± 10	Morphine	N	48h	CD3+/CD4+/CD8+/CD4/CD8
	Shao, Y. J.	2018	GC	61/27	53.3 ± 4.9	Dezocine+Ropivacaine	Fentanyl+Ropivacaine	/	CD3+/CD4+/CD8+/CD4/CD8
	Wang, R. D.	2020	HCC	64/16	55.18 ± 11.09	Parecoxib	N	48h	CD3+/CD4+/CD8+/CD4/CD8/NK
	Wang, Z. Y.	2006	GC	/	26-40	Morphine	Tramadol	/	CD3+/CD4+/CD8+/CD4/CD8
						I1:Tramadol+Lornoxicam			
	Bakr, M. A. E. M.	2016	BC	F	50.90 ± 2.32	Morphine	Tramadol	/	CD3+/CD4+/CD8+
						I1:Ketorolac			
	Li, A.	2018	CRC	33/26	52.97 ± 14.72	Ropivacaine+Dexmedetomidine	Ropivacaine	/	CD4+/CD8+/CD4/CD8
SACT	Zhao, J.	2022	LC	99/21	64.19 ± 12.68	TP	N	42d	CD4+/CD8+/CD4/CD8/NK
	Wang, D.	2018	HCC	38/18	61.3 ± 19.5	TP	TACE	24w	CD3+/CD4+/CD8+/CD4/CD8
	Chen, C.	2023	HCC	80/70	53.16 ± 8.85	TD	TACE	/	CD3+/CD4/CD8
	Li, X.	2019	HCC	46/38	46.1 ± 1.4	TD	TACE	/	CD3+/CD4+/CD8+/CD4/CD8
	Lin, S.	2022	BC	F	45.23 ± 3.79	TD	CD	/	CD4+/CD8+/CD4/CD8/NK
	Gao, Y.	2021	EC	67/51	45.1 ± 18.0	RT	CD	6m	CD3+/CD4+/CD8+
	Quan, Y.	2009	HCC	Aug-32	47	TST+TACE	TACE	/	CD3+/CD4+/CD8+/CD4/CD8
	Zhao,Y.S.	2016	HCC	Dec-48	53	TCM+TACE	TACE	4w	CD3+/CD4+/CD8+/CD4/CD8
	Li, T	2007	HCC	32/18	36-68	TP+TACE	TACE	3w	CD4+/CD8+/NK
	Yu, H.	2023	OC	F	58.62 ± 5.73	CRS+HIPEC	HIPEC	/	CD3+/CD4+/CD3/CD4
	Gu, R. M.	2012	GC	48/17	58	HIIC+EN	EN	7d	CD4+/CD8+/CD4/CD8/NK
						I1:HIIC+PN			
	Nie, Z.	2021	AEG	/	/	HIPEC	N	/	CD3+/CD4+/CD8+
	Zhou, L.	2019	LC	63/73	72.7 ± 3.1	HIIC+HT	HIIC	12w	CD3+/CD4+/CD8+
	Zong, L.	2019	GC	58/38	39-70	IEN+NACT	NACT	14d	CD4+/CD8+/CD4/CD8
	Zhang, J.	2022	CC	F	46.3 ± 7.4	PC	PF	/	CD3+/CD4+/CD8+
	Liu, M.	2022	CC	F	44.21 ± 6.18	LN+NACT	TOS+NACT	6-9w	CD3+/CD4+/CD8+/CD4/CD8
	Cesana, G. C.	2007	GC	41/27	69	I	N	3d	CD3+/CD4+/CD8+/NK
	Dai, C. M.	2021	CHOL/HCC	/	18-75	TCM+TACE	TACE	4w	CD3+/CD4+/CD4/CD8
TCM	Wu, B.	2007	GC	22/18	32-77	TCM	N	/	CD3+/CD4+/CD8+/CD4/CD8
	Wang, J. Y.	2007	EC	Feb-58	58.25	TCM	N	7d	CD3+/CD4+/CD8+/CD4/CD8
	Zhao, S.	2017	HC	43/37	65.82 ± 8.11	TCM	N	3m	CD3+/CD4+/CD8+/CD4/CD8
	Dai, C. M.	2021	CHOL/HCC	/	18-75	TCM+TACE	TACE	4w	CD3+/CD4+/CD4/CD8
	Cai, J.	2008	GC	/	/	EN+TCM	TPN	9d	CD3+/CD4+/CD4/CD8
						I1:EN			
	Huang, Z. R.	2019	LC	39/21	57	TCM	N	12d	CD3+/CD4+/CD8+/CD4/CD8/NK
Psychotherapy	Zhao, X.	2016	LC	84/40	52.63± 10.65	CPI	N	/	CD3+/CD4+/CD8+/CD4/CD8
	Lengacher, C. A.	2013	BC	F	58	MBSR	N	/	CD4+/CD8+/CD4/CD8
ERAS	Chen, L.	2016	EC	209/67	56.43 ± 13.28	ERAS	N	/	CD3+/CD4+/CD8+/CD4/CD8
	Ding, H.	2020	LC	51/49	58.47 ± 6.25	ERAS	N	2w	CD3+/CD4+/CD4/CD8
Other	Zhao, X.	2020	GC/EC/CRC	47/42	53.19 ± 3.22	IW	N	/	CD3+/CD4+/CD8+/CD4/CD8
	Zhao, J. L.	2021	NSCLC	29/41	56.03 ± 1.17	SLD	N	/	CD4+/CD8+/CD4/CD8
	Chen, X. Y.	2017	LC/EC	83/71	67.8 ± 18.6	MPS	N	/	CD3+/CD4+/CD8+/CD4/CD8
	Xing, R.	2022	GC	63/26	69.38	TEAS + TAPB	IV	/	CD3+/CD4+/CD8+/CD4/CD8
						I1: TAPB			
	Zhu, D.	2012	CRC	36/24	61.2 ± 10.5	Probiotics	Antibiotics	7d	CD3+/CD4+/CD8+/CD4/CD8/NK
	Zhang, L.	2013	EC/LC	67/13	56	Ulinastatin	N	7d	CD3+/CD4+/CD8+/CD4/CD8
	Li, Y.	2005	GC/CRC	29/20	51.5	CIM	N	18d	CD4+/CD8+/CD4/CD8
	Gao, Y.	2021	EC	67/51	45.1 ± 18.0	RT	CD	6m	CD3+/CD4+/CD8+
	Quan, Y.	2009	LC	Aug-32	47	TST+TACE	TACE	/	CD3+/CD4+/CD8+/CD4/CD8

### 3.2 Head-to-head meta-analysis of treatment efficacy

We conducted a comprehensive head-to-head meta-analysis to evaluate the impact of various intervention measures on T lymphocytes in patients with cancer undergoing surgical treatment. The results are summarized in **Table 2** and supported by detailed forest plots and funnel plots (**Supplementary Figures 1, S2**). This analysis encompasses multiple categories of intervention and evaluate the impact of these intervention measures on key immune parameters: CD3^+^ T cells, CD4^+^ T cells, CD8^+^ T cells, CD4^+^/CD8^+^ ratio and NK cells.

**Table 2:** Pairwise comparisons of intervention effects on immune cell counts.

Intervention	Outcomes	Studies(participants)	Statistical method(95% CI)	Effect estimate	P	I²	RoB	GRADE
Hysteroscopy	CD3	13 (884)	Mean Difference (IV, Random)	-0.08 [-0.64, 0.48]	0.0003	67	Low	Low
	CD4	20 (1370)	Mean Difference (IV, Random)	-0.31 [-0.69, 0.07]	0.001	56	Some Concerns	Low
	CD8	20 (1370)	Mean Difference (IV, Random)	-0.70 [-1.14, -0.26]*	0.002	55	Some Concerns	Low
	CD4/CD8	10 (728)	Mean Difference (IV, Random)	0.08 [0.00, 0.16]*	0.0001	74	Low	Moderate
	NK	2 (160)	Mean Difference (IV, Fixed)	-2.00 [-5.16, 1.15]	0.98	0	Low	Low
Immune	CD3	3 (134)	Mean Difference (IV, Random)	-0.29 [-5.09, 4.51]	0.31	15	Low	Moderate
	CD4	10 (727)	Std. Mean Difference (IV, Random)	0.29 [-0.20, 0.78]	0.00001	90	Low	Low
	CD8	10 (727)	Std. Mean Difference (IV, Fixed)	0.19 [0.05, 0.34]*	0.7	0	Low	High
	CD4/CD8	6 (382)	Mean Difference (IV, Fixed)	0.21 [0.10, 0.32]*	0.29	19	Low	High
Dexmedetomidine	CD3	14 (1812)	Mean Difference (IV, Random)	3.76 [1.88, 5.63]*	0.00001	85	Low	Moderate
	CD4	14 (2044)	Mean Difference (IV, Random)	1.76 [-1.13, 4.64]	0.00001	97	Low	Low
	CD8	14 (2044)	Mean Difference (IV, Random)	-1.20 [-2.10, -0.30]*	0.00001	81	Low	Moderate
	CD4/CD8	18 (2284)	Mean Difference (IV, Random)	0.17 [0.07, 0.28]*	0.00001	95	Low	Low
	NK	4 (800)	Mean Difference (IV, Random)	1.08 [-0.39, 2.56]	0.02	69	Some Concerns	Low
ERAS	CD3	4 (880)	Mean Difference (IV, Random)	5.00 [3.43, 6.58]*	0.0001	86	Low	Moderate
	CD4	4 (880)	Mean Difference (IV, Random)	3.14 [0.78, 5.51]*	0.0001	86	Low	Moderate
	CD8	3 (780)	Mean Difference (IV, Fixed)	-0.14 [-1.01, 0.73]	0.18	42	Some Concerns	Low
	CD4/CD8	4 (880)	Mean Difference (IV, Random)	0.13 [0.02, 0.25]*	0.04	63	Low	Moderate
Psychotherapy	CD3	2 (206)	Mean Difference (IV, Fixed)	-0.92 [-6.00, 4.16]	0.88	0	Low	Moderate
	CD4	2 (206)	Mean Difference (IV, Fixed)	1.33 [-2.88, 5.54]	0.43	0	Low	Moderate
	CD8	2 (206)	Mean Difference (IV, Fixed)	-2.25 [-6.45, 1.95]	0.66	0	Low	Moderate
	CD4/CD8	2 (206)	Mean Difference (IV, Fixed)	0.26 [-0.13, 0.65]	0.97	0	Low	Moderate
TCM	CD3	10 (561)	Mean Difference (IV, Random)	8.53 [-2.68, 19.74]	0.00001	97	Some Concerns	Low
	CD4	10 (561)	Mean Difference (IV, Random)	6.51 [-1.68, 14.69]	0.00001	96	Some Concerns	Low
	CD8	7 (400)	Mean Difference (IV, Random)	-3.98 [-8.30, 0.34]	0.0008	74	Low	Low
	CD4/CD8	10 (561)	Mean Difference (IV, Random)	0.15 [0.00, 0.29]	0.005	62	Some Concerns	Low
	NK	2 (120)	Mean Difference (IV, Fixed)	34.81 [33.34, 36.27]*	0.77	0	Low	High
TP	CD3	2 (112)	Mean Difference (IV, Fixed)	10.31 [3.66, 16.96]*	0.88	0	Low	High
	CD4	5 (332)	Mean Difference (IV, Random)	10.91 [4.36, 17.46]*	0.0001	84	Low	Moderate
	CD8	5 (332)	Mean Difference (IV, Random)	2.44 [-1.69, 6.56]	0.03	64	Low	Low
	CD4/CD8	3 (232)	Mean Difference (IV, Random)	0.36 [0.14, 0.58]*	0.1	56	Low	High
	NK	3 (220)	Mean Difference (IV, Random)	8.35 [1.28, 15.42]*	0.002	84	Low	Moderate
TD	CD3	2 (264)	Mean Difference (IV, Random)	11.26 [0.97, 21.54]*	0.00001	96	Low	Moderate
	CD4	3 (294)	Mean Difference (IV, Fixed)	10.56 [8.66, 12.46]*	0.96	0	Low	High
	CD8	3 (294)	Mean Difference (IV, Random)	-6.01 [-7.94, -4.09]*	0.1	56	Low	Moderate
	CD4/CD8	4 (444)	Mean Difference (IV, Fixed)	0.37 [0.31, 0.42]*	0.31	15	Low	High
	NK	2 (180)	Mean Difference (IV, Fixed)	2.91 [1.80, 4.03]*	0.77	0	Some Concerns	Moderate
CD	CD3	2 (318)	Mean Difference (IV, Fixed)	2.77 [1.16, 4.38]*	0.8	0	Low	High
	CD4	3 (360)	Mean Difference (IV, Fixed)	2.25 [0.67, 3.82]*	0.96	0	Low	High
	CD8	2 (294)	Mean Difference (IV, Fixed)	-1.97 [-3.90, -0.05]*	0.55	0	Low	High
	CD4/CD8	1 (42)	Mean Difference (IV, Random)	0.13 [-0.28, 0.54]	0.53	0	Some Concerns	Moderate
	NK	1 (42)	Mean Difference (IV, Fixed)	-1.00 [-4.76, 2.76]	0.6	0	Some Concerns	Moderate
NIV	CD3	18 (1776)	Mean Difference (IV, Random)	5.08 [3.10, 7.07]*	0.00001	83	Low	Moderate
	CD4	19 (1816)	Mean Difference (IV, Random)	2.30 [0.73, 3.87]*	0.00001	76	Low	Moderate
	CD8	19 (1816)	Mean Difference (IV, Random)	-0.70 [-2.20, 0.80]	0.00001	89	Low	Low
	CD4/CD8	19 (1816)	Mean Difference (IV, Fixed)	0.10 [0.06, 0.15]*	0.23	19	Low	Low
PPF	CD3	11 (491)	Mean Difference (IV, Fixed)	0.36 [0.17, 0.55]*	0.15	31	Low	High
	CD4	13 (571)	Mean Difference (IV, Random)	0.14 [-0.32, 0.59]	0.00001	81	Low	Low
	CD8	13 (571)	Mean Difference (IV, Fixed)	-0.02 [-0.12, 0.09]	0.1	35	Low	Moderate
	CD4/CD8	13 (571)	Mean Difference (IV, Fixed)	0.20 [0.04, 0.35]*	1	0	Low	High
SEV	CD3	11 (491)	Mean Difference (IV, Fixed)	-0.36 [-0.55, -0.17]*	0.15	31	Low	High
	CD4	11 (491)	Mean Difference (IV, Random)	-0.21 [-0.48, 0.06]	0.007	59	Low	Low
	CD8	11 (491)	Mean Difference (IV, Fixed)	0.01 [-0.09, 0.12]	0.96	0	Low	Moderate
	CD4/CD8	11 (491)	Mean Difference (IV, Fixed)	-0.19 [-0.36, -0.03]*	1	0	Low	High
Fentanyl	CD3	13 (722)	Mean Difference (IV, Random)	-1.03 [-3.63, 1.56]	0.00001	77	Low	Low
	CD4	13 (722)	Mean Difference (IV, Random)	-2.73 [-4.72, -0.73]*	0.0001	71	Low	Moderate
	CD8	13 (722)	Mean Difference (IV, Fixed)	2.07 [1.52, 2.62]*	0.04	46	Low	High
	CD4/CD8	12 (634)	Mean Difference (IV, Random)	-0.14 [-0.24, -0.05]*	0.0001	73	Some Concerns	Moderate
	NK	5 (248)	Mean Difference (IV, Fixed)	-2.50 [-5.16, 0.16]	0.93	0	Some Concerns	Low
Flurbiprofen	CD3	10 (500)	Mean Difference (IV, Random)	-3.41 [-7.84, 1.02]	0.01	58	Low	Moderate
	CD4	6 (260)	Mean Difference (IV, Fixed)	3.35 [-0.48, 7.17]	0.88	0	Some Concerns	Low
	CD8	6 (260)	Mean Difference (IV, Fixed)	-0.44 [-3.89, 3.01]	1	0	Some Concerns	Low
	CD4/CD8	10 (500)	Mean Difference (IV, Fixed)	-0.17 [-0.36, 0.02]	0.06	46	Low	Moderate
Parecoxib	CD3	6 (420)	Mean Difference (IV, Fixed)	0.73 -0.52, 1.99]	0.05	55	Low	Moderate
	CD4	6 (420)	Mean Difference (IV, Random)	0.28 [-1.65, 2.21]	0.001	75	Low	Low
	CD8	6 (420)	Mean Difference (IV, Fixed)	0.19 [-0.46, 0.84]	0.05	54	Low	Moderate
	CD4/CD8	3 (240)	Mean Difference (IV, Fixed)	0.10 [-0.04, 0.24]	0.94	0	Some Concerns	Low
	NK	6 (420)	Mean Difference (IV, Fixed)	-1.08 [-1.91, -0.25]*	0.02	63	Low	Moderate
Morphine	CD3	20 (756)	Mean Difference (IV, Fixed)	-2.03 [-3.03, -1.03]*	0.03	41	Low	High
	CD4	20 (756)	Mean Difference (IV, Random)	-2.71 [-5.53, 0.11]	0.00001	93	Low	Moderate
	CD8	20 (756)	Mean Difference (IV, Random)	-1.37 [-3.00, 0.26]	0.00001	80	Low	Moderate
	CD4/CD8	16 (596)	Mean Difference (IV, Random)	0.02 [-0.09, 0.13]	0.0004	63	Some Concerns	Low
Dezocine	CD3	5 (248)	Mean Difference (IV, Fixed)	5.31 [2.13, 8.48]*	0.13	44	Some Concerns	Moderate
	CD4	5 (248)	Mean Difference (IV, Fixed)	3.70 [0.47, 6.92]*	0.64	0	Some Concerns	Moderate
	CD8	5 (248)	Mean Difference (IV, Fixed)	-0.12 [-2.98, 2.74]	1	0	Some Concerns	Low
	NK	5 (248)	Mean Difference (IV, Fixed)	2.50 [-0.16, 5.16]	0.93	0	Some Concerns	Low
Tramadol	CD3	12 (396)	Mean Difference (IV, Fixed)	-0.56 [-1.62, 0.50]	0.09	38	Low	Moderate
	CD4	12 (396)	Mean Difference (IV, Random)	0.87 [-2.16, 3.90]	0.00001	93	Low	Low
	CD8	12 (396)	Mean Difference (IV, Random)	0.95 [-0.53, 2.43]	0.0001	74	Low	Low
EN	CD3	12 (2620)	Mean Difference (IV, Random)	5.47 [1.27, 9.68]*	0.00001	98	Low	Moderate
	CD4	14 (2812)	Mean Difference (IV, Random)	4.93 [1.35, 8.51]*	0.00001	98	Low	Moderate
	CD8	7 (1172)	Mean Difference (IV, Random)	0.28 [-1.85, 2.42]	0.00001	94	Low	Low
	CD4/CD8	14 (2812)	Mean Difference (IV, Random)	0.11 [-0.02, 0.24]	0.00001	91	Low	Low
	NK	3 (180)	Mean Difference (IV, Random)	3.19 [-0.95, 7.34]	0.008	79	Some Concerns	Low
EEN	CD3	3 (846)	Std. Mean Difference (IV, Random)	0.61 [-0.51, 1.73]	0.00001	98	Some Concerns	Low
	CD4	5 (1098)	Std. Mean Difference (IV, Random)	0.38 [-0.37, 1.14]	0.00001	97	Low	Moderate
	CD8	5 (1098)	Std. Mean Difference (IV, Random)	0.20 [-0.02, 0.42]	0.02	65	Low	Moderate
	CD4/CD8	5 (1098)	Mean Difference (IV, Random)	0.19 [-0.09, 0.46]	0.00001	88	Low	Moderate
EN+PN	CD3	7 (532)	Std. Mean Difference (IV, Random)	0.28 [0.02, 0.55]*	0.04	55	Low	High
	CD4	7 (532)	Std. Mean Difference (IV, Random)	0.63 [0.12, 1.14]*	0.00001	87	Low	High
	CD8	4 (214)	Std. Mean Difference (IV, Fixed)	0.07 [-0.20, 0.34]	0.93	0	Some Concerns	Low
	CD4/CD8	7 (532)	Mean Difference (IV, Random)	0.13 [-0.12, 0.38]	0.00001	84	Low	Moderate
Immune+PN	CD3	5 (442)	Mean Difference (IV, Random)	0.90 [-3.80, 5.61]	0.02	66	Low	Moderate
	CD4	5 (442)	Mean Difference (IV, Random)	3.18 [-1.00, 7.36]	0.007	72	Low	Moderate
	CD8	5 (442)	Mean Difference (IV, Fixed)	-0.02 [-2.40, 2.35]	0.95	0	Low	Moderate
	CD4/CD8	5 (442)	Mean Difference (IV, Random)	0.05 [-0.28, 0.37]	0.06	55	Low	Moderate
EN before	CD4	4 (444)	Mean Difference (IV, Random)	6.04 [1.15, 10.94]*	0.0001	86	Low	Moderate
	CD8	4 (444)	Mean Difference (IV, Fixed)	1.04 [-1.08, 3.15]	0.46	0	Low	Low
	CD4/CD8	4 (444)	Mean Difference (IV, Fixed)	0.20 [0.03, 0.37]*	0.71	0	Low	Moderate

ERAS, ERAS, Enhanced Recovery after Surgery; TCM, Traditional Chinese medicine; TP, Thymic peptide; TD, Targeted therapy; CD, Chemotherapy; NIV,Non-intravenous administration; PPF, Propofol; SEV, Sevoflurane; EN, Enteral nutrition; EEN, Early enteral nutrition; PN, Parenteral nutrition. *significant difference.

Regarding surgical modalities, hysteroscopy was associated with a significant decrease in CD8^+^ T cells (MD = -0.70, 95% CI [-1.14, -0.26]) and an increase in the CD4^+^/CD8^+^ ratio (MD = 0.08, 95% CI [0.00, 0.16])(**Table 2**). Among anesthetic interventions, dexmedetomidine demonstrated a statistically significant increase in CD3^+^ T cells (MD = 3.76, 95% CI [1.88, 5.63], P < 0.05) and a significant reduction in CD8^+^ T cells (MD = -1.20, 95% CI [-2.10, -0.30], P < 0.05), while significant increase in the CD4^+^/CD8^+^ ratio (MD = 0.17, 95% CI [0.07, 0.28], P < 0.05). On the contrary, Fentanyl was associated with a significant reduction in CD4^+^ T cells (MD = -2.73, 95% CI [-4.72, -0.73], P < 0.05) and an unfavorable CD4^+^/CD8^+^ ratio (MD = -0.14, 95% CI [-0.24, -0.05]), P < 0.05).Morphine also showed significant immunosuppressive effects, particularly on CD3^+^ T cells (MD = -2.03, 95% CI [-3.03, -1.03], P < 0.05). In the domain of nutritional support, Enteral Nutrition (EN) demonstrated beneficial effects, with significant increases in both CD3^+^ T cells (MD = 5.47, 95% CI [1.27, 9.68], P < 0.05) and CD4^+^ T cells (MD = 4.93, 95% CI [1.35, 8.51], P < 0.05). Preoperative EN before surgery specifically enhanced CD4^+^ T cells (MD = 6.04, 95% CI [1.15, 10.94], P < 0.05). Among systemic anti-cancer therapies, thymic peptide (TP) and targeted therapy (TD) exhibited particularly strong immunomodulatory effects. TP significantly increased CD4^+^T cells (MD = 10.91, 95% CI [4.36,17.46], P < 0.05) and NK cells (MD = 8.35, 95%CI [1.28,15.42], P < 0.05). And it showed a potent effect on CD3^+^T cells (MD = 11.26, 95%CI [0.97,21.54], P < 0.05)(**Table 2**). The assessment of publication bias using a funnel plot (**Supplementary Figure 2**) shows. Most of the comparison results show an overall symmetrical distribution. The grade certainty scores in different comparisons ranged from low to high, and most outcomes were rated as moderately certain, which provided support for the reliability of the main research results. Bias risk assessment indicated that the included studies mostly have low risk in key areas.

These comprehensive head-to-head analyses provide strong evidence for the immunomodulatory effects of various perioperative intervention measures. It provides important insights for choosing strategies to maintain the immune function of patients with cancer during surgical treatment. The part head-to-head meta-analysis showed that the careful selection of anesthetics, analgesic regimens and nutritional support can significantly affect the recovery of postoperative immune function. And it may have an impact on the long-term oncological prognosis. Time-stratified analyses for major intervention nodes, as presented in **Supplementary Figure 9**, showed that for most interventions, no significant heterogeneity was detected across different postoperative measurement time points, thus supporting the validity of pooling data from various time points in our primary network meta-analysis; for the few interventions where some temporal heterogeneity was suggested, further stratified subgroup analyses could not be meaningfully performed because the number of available studies within each time stratum was fewer than 10 in all cases, which precluded reliable statistical comparisons.

### 3.3 Network meta-analysis of treatment efficacy

**Figure 2:**
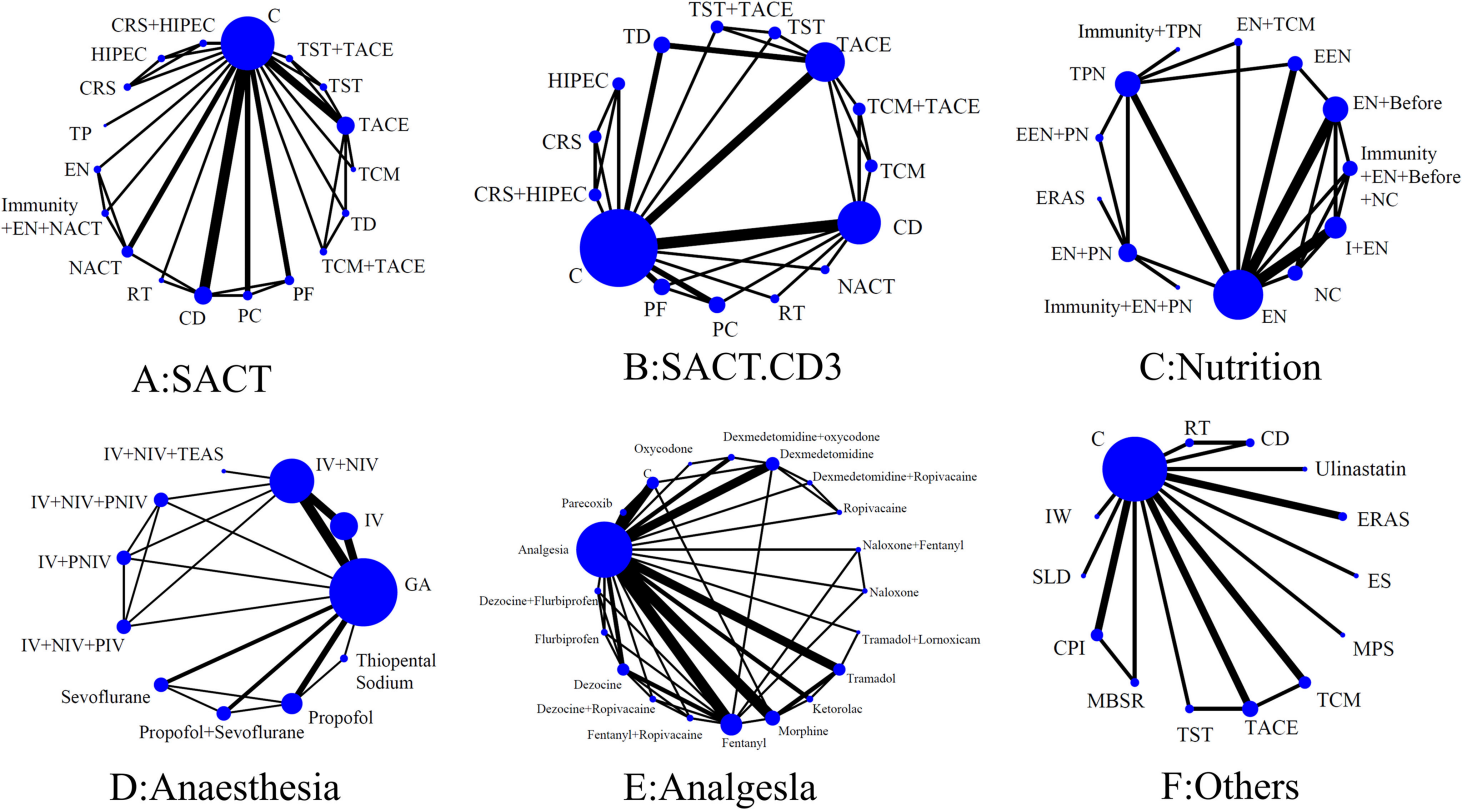
Network plot of available comparisons among included interventions. Thickness of line connecting nodes is altogether to number of trials which directly comparing. **(A)** Comparison of efficacy in solid cancers with SACT from CD4 outcomes, **(B)** Comparison of efficacy in solid cancers with SACT from CD3 outcomes, **(C) **Comparison of efficacy in solid cancers with different nutrition therapy methods from CD4 outcomes, **(D)** Comparison of efficacy in solid cancers with different anesthesia therapy methods from CD4 outcomes, **(E)** Comparison of efficacy in solid cancers with different analgesia therapy methods from CD4 outcomes, **(F)** Comparison of efficacy in solid cancers with other therapy methods from CD4 outcomes. C, Control group; CD, Chemotherapy; CRS, Cytoreductive surgery; EN, Enteral nutrition; HIPEC, Hyperthermic intraperitoneal chemotherapy; IEN+NACT, Immune-boosting enteral nutrition+Neoadjuvant chemotherapy; NACT, Neoadjuvant chemotherapy; PC, Paclitaxel+Cisplatin; PF, Cisplatin+Fluorouracil; RT, Radiotherapy; TACE, Transarterial chemoembolization; TD, Targeted therapy; TP, Thymic peptide; TCM, Traditional Chinese medicine; TST, Tumor stromal therapy; EEN, Early enteral nutrition; EN+Before, Enteral nutrition before surgery; ERAS, Enhanced Recovery after Surgery; HIC, Hyperthermic intraperitoneal chemotherapy; Immunity+EN+Before+NC, Preoperative immune-boosting diet + neoadjuvant chemotherapy; NC, Neoadjuvant chemotherapy; PN, Parenteral nutrition; TPN, Total parenteral nutrition; GA, General anesthesia; IV, Intravenous administration; NIV, Non-intravenous administration; PIV, Postoperative intravenous administration; PNIV, Postoperative non-intravenous administration; TEAS, Transcutaneous electrical acupoint stimulation; CIM, Cimetidine; CPI, Comprehensive Psychological intervention; ES, Electrical stimulation therapy; IW, Intraoperative Warming; MPS, Mannan peptide; MBSR, Mindfulness-based stress reduction therapy; SLD, Selective lymph node dissection; TST, Tumor stromal therapy. Network visualization was achieved through proportional line weighting in network plots, where the thickness of connections between intervention nodes directly corresponded to the number of trials providing direct comparison evidence.

**Figure 3:**
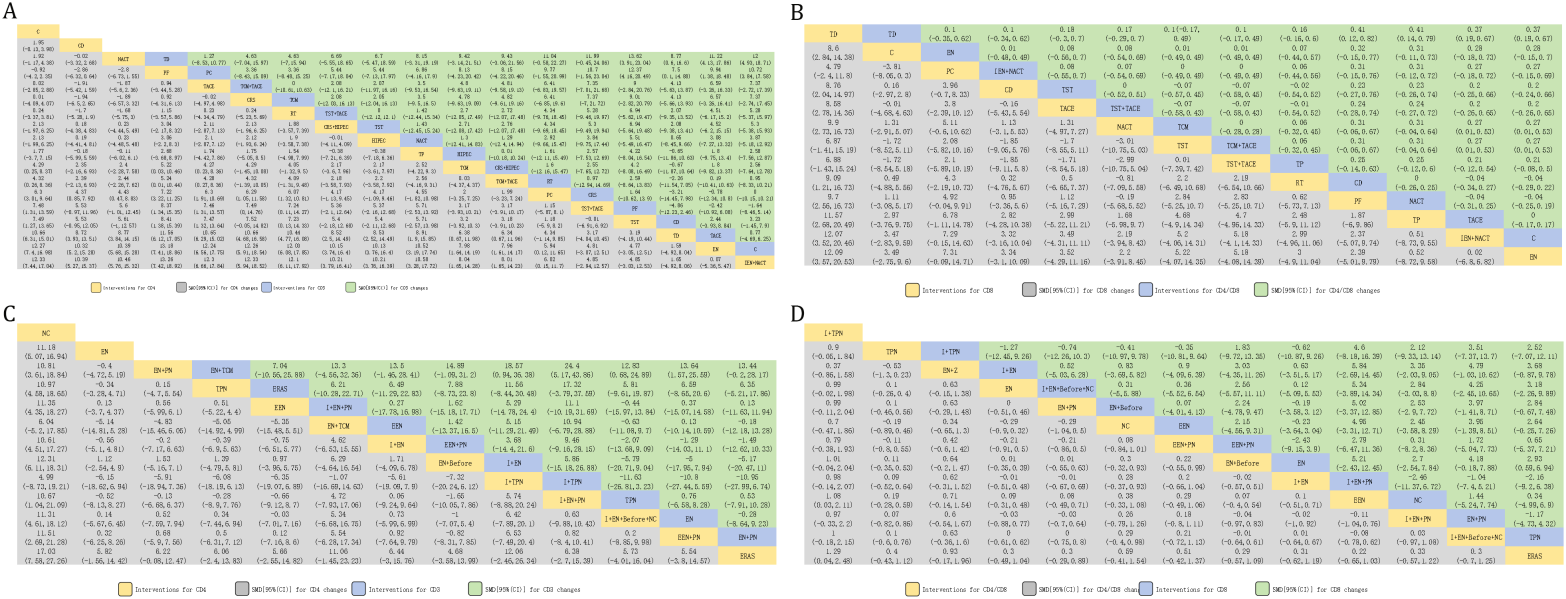
Network league figures of available comparisons among SACT and nutrition therapy for 24 hours after therapy. **(A) **CD3 and CD4 in SACT, **(B) **CD8 and CD4/CD8 in SACT, **(C) **CD3 and CD4 in nutrition therapy, **(D) **CD8 for 48 hours and CD4/CD8 in nutrition therapy. The SMD for comparisons are in the cell in common between the row column and column treatment, a SMD higher than 0 favor raw-defining treatment. C, Control group; CD, Chemotherapy; CRS, Cytoreductive surgery; EN, Enteral nutrition; HIPEC, Hyperthermic intraperitoneal chemotherapy; IEN+NACT, Immune-boosting enteral nutrition+Neoadjuvant chemotherapy; NACT, Neoadjuvant chemotherapy; PC, Paclitaxel+Cisplatin; PF, Cisplatin+Fluorouracil; RT, Radiotherapy; TACE, Transarterial chemoembolization; TD, Targeted therapy; TP, Thymic peptide; TCM, Traditional Chinese medicine; TST, Tumor stromal therapy; EEN, Early enteral nutrition; EN+Before, Enteral nutrition before surgery; ERAS, Enhanced Recovery after Surgery; HIC, Hyperthermic intraperitoneal chemotherapy; Immunity+EN+Before+NC, Preoperative immune-boosting diet + neoadjuvant chemotherapy; NC, Neoadjuvant chemotherapy; PN, Parenteral nutrition; TPN, Total parenteral nutrition.

To comprehensively evaluate and compare the efficacy of various therapeutic interventions on postoperative immune function in patients with solid cancers, a NMA was conducted, focusing on outcomes including CD3^+^ T-cell counts, CD4^+^ T-cell counts, CD8^+^ T-cell counts, and CD4^+^/CD8^+^ ratios. The network plots of available comparisons among the included interventions are illustrated in **Figure 2**, where the thickness of the lines connecting the nodes corresponds to the number of trials directly comparing the connected treatments. Efficacy of Systemic Anti-Cancer Therapies (SACT) on CD3^+^ and CD4^+^ Outcomes. (**Figures 2A, B**) The network for SACT interventions incorporated multiple treatment.

modalities, including chemotherapy (CD), neoadjuvant chemotherapy (NACT), targeted therapy (TD), transarterial chemoembolization (TACE), and combinations such as Paclitaxel+Cisplatin (PC) and Cisplatin+Fluorouracil (PF). The league table results (**Figures 3A, B**) revealed significant variations in efficacy. For CD4^+^ T cell levels, immune-enhanced enteral nutrition combined with neoadjuvant chemotherapy (IEN+NACT) significantly improved CD4^+^ count (SMD vs. Control: 12.33, 95% CI: 7.44 to 17.04). It is one of the most effective intervention measures. Similarly, targeted therapy (TD) also demonstrated significant positive effects (SMD vs. Control: 10.66, 95% CI: 6.31 to 15.01). In contrast, some conventional chemotherapy regimens showed less obvious or no significant effect. A similar hierarchical structure was observed for the results of CD3^+^ T cells. Among them, IEN + NACT and TD once again ranked high, highlighting the potential benefits of combining immunomodulatory nutritional support with systemic therapy, and the specific efficacy of targeted drugs in maintaining or enhancing the T cell population. The network for nutritional interventions (**Figure 2C**) included Enteral Nutrition (EN), Parenteral Nutrition (PN), Total Parenteral Nutrition (TPN), Early Enteral Nutrition (EEN), and Enhanced Recovery After Surgery (ERAS) protocols, among others. The comparative league figures (**Figures 3C, D**) indicated that Enhanced Recovery After Surgery (ERAS) was associated with the most substantial improvement in CD4^+^ T-cell counts (SMD vs. Control: 17.03, 95% CI: 7.58 to 27.26). This was followed by Enteral Nutrition administered preoperatively (EN+Before) and Immune-boosting Enteral Nutrition combined with Preoperative immune-boosting diet and Neoadjuvant Chemotherapy (I+EN+Before+NC). These findings suggest that perioperative nutritional strategies, particularly those integrated within a structured recovery pathway or incorporating immune-enhancing components, are highly effective in mitigating the immunosuppressive effects of postoperative cancer treatment.

**Figure 4:**
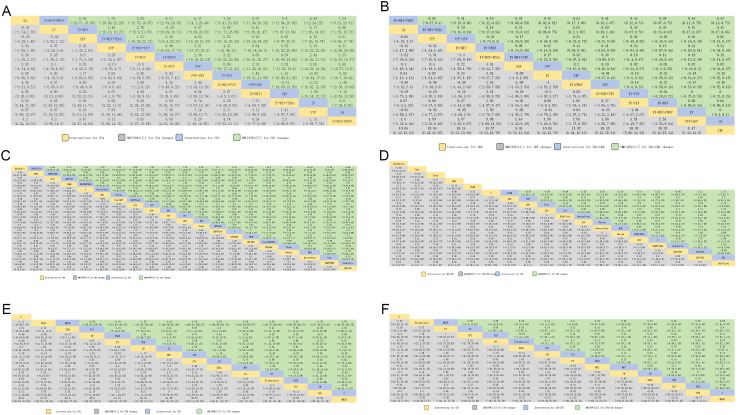
Network league figures of available comparisons among anesthesia therapy, analgesia therapy and other therapy methods for 24 hours after therapy. **(A)** CD3 and CD4 in anesthesia therapy, **(B) **CD8 and CD4/CD8 in anesthesia therapy, **(C) **CD3 and CD4 in analgesia therapy, **(D) **CD8 and CD4/CD8 in analgesia therapy, **(E) **CD3 and CD4 in other therapy methods, **(F)** CD8 and CD4/CD8 in other therapy methods. The SMD for comparisons are in the cell in common between the row column and column treatment, a SMD higher than 0 favor raw-defining treatment. GA, General anesthesia; IV, Intravenous administration; NIV, Non-intravenous administration; PIV, Postoperative intravenous administration; PNIV, Postoperative non-intravenous administration; TEAS, Transcutaneous electrical acupoint stimulation; CIM, Cimetidine; CPI, Comprehensive Psychological intervention; ES, Electrical stimulation therapy; IW, Intraoperative Warming; MPS, Mannan peptide; MBSR, Mindfulness-based stress reduction therapy; SLD, Selective lymph node dissection; TST, Tumor stromal therapy.

The networks for anesthesia (**Figure 2D**) and analgesia (**Figure 2E**) therapies evaluated various administration routes and drug combinations. For anesthesia, techniques involving Transcutaneous Electrical Acupoint Stimulation (TEAS) combined with intravenous/non-intravenous methods (IV+NIV+TEAS) showed promising results in preserving CD4^+^ levels (SMD vs. General Anesthesia (GA): 6.63, 95% CI: 2.44 to 11.02) (**Figures 4A, B**). In analgesia, regimens containing Dexmedetomidine (DEX), particularly DEX+Oxycodone (OXY), demonstrated significant efficacy in maintaining CD4^+^ counts (SMD vs. standard Analgesia control: 4.38, 95% CI: 0.75 to 8.01) (**Figures 4C, D**). The findings suggest that selecting specific agents for use during procedures and for managing responses after procedures can affect function of the system that provides responses to conditions. Some adjuvant drugs, such as transcutaneous electrical stimulation and α-2 receptor agonists like dexmedetomidine, have protective effects. For other supportive therapies (network in **Figure 2F**), electrical stimulation therapy (ES) and comprehensive psychological intervention (CPI) showed non-significant but positive trends **Figures 4E, F**. These findings emphasize the importance of integrating specific immunomodulatory methods into conventional postoperative cancer treatment regimens to optimize patient prognosis by supporting immune capacity.

### 3.4 Head-to-head meta-analysis of treatment safety

The analysis examining treatment safety combined multiple studies to assess how different treatment approaches relate to various negative outcomes. The treatment approaches included targeted therapy, immunotherapy, non-intravenous administration, and dexmedetomidine. The analysis categorized safety outcomes into problems affecting digestion, problems from infection, problems affecting skin, problems with blood pressure measures, problems affecting the nervous system, and other various problems. Each category was further subdivided into specific adverse reactions to facilitate a detailed subgroup analysis, as summarized in **Table 3**. The corresponding forest plots and funnel plots for these comparisons are shown in **Supplementary Figures 1** and **2**. The pooled OR of gastrointestinal disorders was 1.05 (95% CI: 0.74–1.51), indicating no significant increase in risk, with low heterogeneity (*P* = 0.44, *I²* = 1%). Notably, nausea and vomiting (4 studies, 284 participants) demonstrated a significantly reduced risk (OR = 0.33, 95% CI: 0.11–0.99), suggesting a potential protective effect of the interventions in this subgroup. The overall risk of infection was significantly reduced (OR = 0.66, 95% CI: 0.47–0.92). Subgroup analyses indicated non-significant trends for most specific infection types. The overall risk of skin disorders was significantly increased (OR = 1.75, 95% CI: 1.11–2.77), with moderate heterogeneity (*P* = 0.20, *I²* = 35%). Three studies involving 214 participants reported on blood pressure abnormalities. The overall effect was not significant (OR = 1.50, 95% CI: 0.68–3.31). Subgroup analyses for hypertension and hypotension also showed non-significant results. These outcomes warrant careful monitoring in clinical practice, especially in vulnerable populations. The overall risk of nervous system disorders was not significantly different (OR = 0.70, 95% CI: 0.39–1.26). Subgroup outcomes for pain, neurological dysfunction, dizziness, and respiratory depression were generally non-significant. A pooled analysis of 10 studies and 1,217 participants for other disorders revealed a significant reduction in risk (OR = 0.62, 95% CI: 0.44–0.88). The analysis combining studies demonstrates that approaches involving targeted therapy, immunotherapy, non-intravenous administration, and dexmedetomidine relate to outcomes showing favorable patterns for most categories of events that occur. The consistency of these findings is supported by the funnel plot (**Supplementary Figures 1, 2**), which showed no substantial publication bias, strengthening the evidence base. The head-to-head meta-analysis confirmed that specific measures such as dexmedetomidine and enteral nutrition can improve immune indicators. However it is unable to sort and determine the most suitable comprehensive treatment strategy. Therefore, we further conducted a network meta-analysis, grouping the intervention measures by clinical domains Systemic Anti-Cancer Therapies (SACT) nutritional support, anesthesia and analgesia) for the network meta-analysis. Collectively, the head-to-head analysis suggests that an anesthetic strategy centered on dexmedetomidine and a perioperative nutritional plan based on enteral nutrition may offer immunoprotective benefits to surgical oncology patients.

**Table 3:** Subgroup analyses of safety outcomes in comparisons of targeted therapy, immunotherapy, non-intravenous administration and dexmedetomidine.

Adverse reactions	Groups	Study (n)	Participants	OR (95% CI)	Heterogeneity (P, I2)	GRADE
Gastrointestinal disorders	Total	13	1120	1.05[0.74, 1.51]	0.44,1%	Moderate
	diarrhea	3	464	1.29 [0.81, 2.06]	0.51,0%	Low
	Gastrointestinal symptoms	4	237	1.30 [0.61, 2.73]	0.50,0%	Moderate
	Nausea and vomiting	4	284	0.33 [0.11, 0.99]*	0.22,33%	Moderate
	Dysphagia	1	15	0.80 [0.10, 6.35]	0.83	Low
	Chylous fistula	1	120	0.33 [0.01, 8.21]	0.50	Low
Infection	Total	29	2455	0.66 [0.47, 0.92]*	0.99,0%	Moderate
	Infectious complications	1	109	0.32 [0.10, 1.08]	0.07	Low
	Incision infection	8	681	0.86 [0.44, 1.68]	0.85,0%	Moderate
	Respiratory tract infection	8	681	0.75 [0.42, 1.31]	0.82,0%	
	Urinary tract infection	2	124	0.65 [0.12, 3.39]	0.76,0%	Moderate
	Abdominal cavity infection	1	120	0.49 [0.04, 5.57]	0.57	Low
	Sepsis	1	109	0.33 [0.01, 8.36]	0.50	Low
	Abscess	1	109	0.49 [0.09, 2.80]	0.42	Low
	Anastomosis leakage	6	372	0.22 [0.03, 1.42]	0.92,0%	Moderate
	Fever	1	150	0.76 [0.33, 1.76]	0.52	Low
Skin disorders	Total	4	418	1.75 [1.11, 2.77]*	0.20,35%	High
	Hand-foot syndrome	2	264	2.17 [0.78, 6.06]	0.07,69%	Low
	Itchy skin	2	40	0.32 [0.01, 8.26]	0.49	Low
	Hair loss	1	114	1.42 [0.55, 3.70]	0.47	Low
Abnormal blood pressure	Total	3	214	1.50 [0.68, 3.31]	0.87,0%	Moderate
	Hypertension	1	114	1.46 [0.62, 3.46]	0.39	Low
	Hypotension	2	100	1.70 [0.22, 13.32]	0.60,0%	Moderate
Nervous system disorders	Total	5	409	0.70 [0.39, 1.26]	0.77,0%	Moderate
	Pain	1	150	0.85 [0.44, 1.63]	0.62	Low
	Neurological dysfunction	1	15	0.86 [0.04, 16.85]	0.92	Low
	Dizziness	2	204	0.32 [0.05, 2.10]	0.65,0%	Moderate
	Respiratory depression	1	40	0.18 [0.01, 4.01]	0.28	Low
Other disorders	Total	10	1217	0.62 [0.44, 0.88]*	0.04,49%	Moderate

*significant difference.

## 4 Discussion

This systematic review and network meta-analysis represents a comprehensive evaluation of the comparative effects of diverse therapeutic interventions on immune function in patients with cancers. Through rigorous synthesis of evidence, we employed both head-to-head and network meta-analytical methods to establish a clear hierarchy of treatments based on their efficacy in modulating key immune parameters. Our analysis reveals several crucial findings. These findings provide individuals managing treatment with a structure based on data for selecting strategies that target disease but also actively maintain and support the individual’s defense system throughout the period of treatment.

Firstly, intervention measures that combine immunonutrition with systemic treatment, especially immune-enhanced enteral nutrition combined with neoadjuvant chemotherapy (IEN + NACT), have demonstrated the most significant benefits in increasing CD4^+^ T cell counts. Similarly, Duan et al. (22) reported that early enteral nutrition could significantly improve the nutritional status and immune function of patients with gastric cancer undergoing neoadjuvant chemotherapy. Secondly, among perioperative management strategies, the Enhanced Recovery after Surgery (ERAS) program was particularly effective in maintaining immune function. It showed the most significant improvement in CD4^+^ T cell count among all nutritional intervention measures. Wang et al. (23) directly demonstrated this point, showing that ERAS intervention could significantly promote the recovery of gastrointestinal and immune functions in patients after gastric tumor surgery. Thirdly, in the field of anesthesia and analgesia, the immune effect of NIV is the better, anesthesia techniques combining transcutaneous electrical acupoint stimulation (TEAS) and analgesia regimens based on dexmedetomidine (DEX) have been identified as the best options for alleviating treatment-induced immunosuppression, just like the research of Chen et al (24). and that of Lee et al (25). Kadantseva et al. showed that sevoflurane significantly altered the postoperative CD4+/CD8+ ratio change from baseline compared with propofol (P = 0.033), suggesting anesthetic technique affects early T−cell balance (26). Oh et al. found no significant difference between propofol and sevoflurane anesthesia on circulating NK cells or T lymphocytes in colorectal cancer surgery, suggesting that immunoprotection may depend more on multimodal strategies than on single anesthetic choice (27). In SACT, targeted therapy exerted the most favorable immune impact, which is consistent with the research conclusion of Lee et al (28). These alignments between our findings and previous focused studies underscore the consistency and robustness of our evidence synthesis.

### 4.1 Comparison with previous research and future perspectives

Multiple previous analyses combining studies examine effects from individual treatment approaches, our study provides the initial comprehensive effort for assessment across treatment approaches in cancer care. Previous research establishes that common cancer treatments show effects limiting immune function (29, 30). Other research shows separately that providing nutrients or particular agents for procedures during treatment may indicate benefits for immune outcomes (31, 32). Looking to the future, our research results have proposed several important research directions. Future research should prioritize verifying these grade rankings in large-scale prospective trials. These trials are specifically designed to compare the top-ranked interventions in different categories (for example, directly comparing IEN + NACT with the comprehensive ERAS pathway). Research should also explore the molecular mechanisms behind these observed effects. And investigate whether these immune protection strategies have enhanced the efficacy of modern immunotherapy. Furthermore, we hope that more basic research and large-scale prospective studies will become available in the future, which would enable more refined subgroup analyses stratified by different postoperative time points, anesthetic regimens, and tumor types, thereby further validating and extending the conclusions of the present network meta-analysis.

### 4.2 Potential mechanisms

The observed hierarchical results can be explained by several mature biological mechanisms. The outstanding performance of IEN + NACT may function through multiple pathways: providing immune nutrients to directly support the proliferation of lymphocytes; enhancing the integrity of the intestinal barrier to reduce systemic inflammation; regulating the inflammatory cytokine spectrum creates a more favorable microenvironment for immune recovery in neoadjuvant chemotherapy (33). The ERAS protocol mainly minimizes surgical stress responses by reducing tissue trauma, optimizing pain control, and maintaining normal body temperature and fluid balance, thereby maintaining the stability of the immune system (34). The effectiveness of TEAS in anesthesia may be mediated by neuroimmune regulation, reducing the production of pro-inflammatory cytokines and better protecting the function of immune cells (35). Dexmedetomidine, as a selective α -2 adrenergic receptor agonist, has demonstrated significant anti-inflammatory and stress-suppressing effects, thereby maintaining the T cell balance (36).

### 4.3 Strengths of the study

The first strength of this study appears in the approach that follows PRISMA requirements and that uses a method examining multiple studies in a systematic form. Unlike previous reviews that typically focus on a single type of intervention, our network meta-analysis is within an integrated framework. This innovative approach enabled both direct and indirect comparisons across a wide spectrum of interventions, generating clinically valuable rankings that bridge specialty divisions.

The methodological rigor of our study is reflected in several aspects: First, we employed a comprehensive search strategy covering multiple major databases to ensure the completeness of included evidence. Second, we established clear inclusion and exclusion criteria, with independent literature screening and data extraction conducted by two researchers to ensure process reproducibility. Third, we used the Cochrane Risk of Bias tool to rigorously assess the quality of all included studies. Finally, we adopted a Bayesian approach to network meta-analysis, evaluating the consistency between direct and indirect comparison results through consistency tests to ensure result reliability.

The clinical relevance of our findings is substantial and multifaceted. By identifying specific intervention measures such as IEN + NACT and ERAS as the top strategies for immune protection, we provide action insights that can be immediately incorporated into multidisciplinary postoperative cancer treatment pathways. Furthermore, our research surpasses existing literature in several key aspects: firstly, we have made a comprehensive comparison of different therapeutic areas for the first time, and previous research mainly focused on a specific field (13, 37, 38). This enables clinicians to evaluate different intervention measures on a unified scale. Second, we focus on immune parameters with direct clinical applicability, offering practical tools for bedside decision-making. Third, our hierarchical analysis provides targeted immune protection strategies for different treatment phases (preoperative, intraoperative, postoperative).This study therefore advances beyond simply establishing efficacy to offering a practical, comparative tool for optimizing the entire postoperative cancer treatment continuum from an immunological perspective, potentially informing clinical decision-making in ways that could meaningfully impact patient outcomes and quality of life. By identifying the most effective immune-preserving strategies, our research establishes an evidence base for improving individualized management of immune function during postoperative cancer treatment.

### 4.4 Limitations

Several limitations of this analysis warrant careful consideration. For the head-to-head meta-analyses, pooling data from different postoperative time points within the same study remains a limitation. A significant constraint was the relatively limited number of studies available for certain intervention comparisons within the networks. This scarcity can increase the uncertainty around effect estimates for less-studied interventions and potentially limit the stability of the network. Consequently, findings for these sparsely represented interventions should be interpreted with appropriate caution. Secondly, the specific timing of immune outcome measurement presents a limitation. Our analysis primarily focused on outcomes measured within 24 hours following the end of the operation initiation, reflecting the data most commonly reported in the included studies. However, the 24-hour time node of targeted therapy refers to the period within 24 hours after the start of treatment. Differences in baseline patient characteristics, cancer types, and treatment protocols across studies, although accounted for in our analytical models using appropriate measures, may still introduce residual heterogeneity into the pooled estimates.

## 5 Conclusion

In conclusion, this network meta-analysis identified the most effective strategies for protecting the immune function of patients with cancer. The continuous advantages of these intervention measures at different treatment stages indicate that integrating immune protection into postoperative cancer treatment requires a multimodal strategy, such as postoperative radiotherapy and chemotherapy for patients, supplemented by immune support therapy, intraoperative non-intravenous anesthesia, dexmedetomidine analgesia, and MBSR throughout the treatment process. These evidence-based strategies may enhance immune protection during postoperative cancer treatment in clinical implementation, thereby potentially improving treatment tolerance, reducing complications, and ultimately contributing to a better prognosis for patients.

## Data Availability

The original contributions presented in the study are included in the article/**Supplementary Material**. Further inquiries can be directed to the corresponding authors.
