# Supplementary material for: What are the beneficial treatment strategies in maintaining T lymphocyte subsets after cancer surgery? A systematic review and network meta-analysis
Source: Front Immunol. 2026 Jul 14;17:1854279. doi: 10.3389/fimmu.2026.1854279 (PMC13408238; doi:10.3389/fimmu.2026.1854279)

Figure S1 Forest plots of available comparisons among all included interventions.

1.1 CD3(Hysteroscopy)

Study or Subgroup	Experimental Mean	Experimental SD	Experimental Total	Control Mean	Control SD	Control Total	Weight	Mean Difference IV, Random, 95% CI
Liu, M.2022	-8.78	3.15	21	-4.3	3.08	21	7.2%	-4.48 [-6.36, -2.60]
Peng, B.2008 1d	-2.99	14.29	26	-6.17	16.51	26	0.4%	3.18 [-5.21, 11.57]
Peng, B.2008 3d	-5.06	17.88	26	-4.7	17.79	26	0.3%	-0.36 [-10.06, 9.34]
Peng, B.2008 5d	-1.16	13.8	26	0.12	15.6	26	0.5%	-1.28 [-9.29, 6.73]
Zhang, L. B.2015 1d	-17.5	7.69	61	-19	6.77	61	4.2%	1.50 [-1.07, 4.07]
Zhang, L. B.2015 3d	-8.4	8.35	61	-11.4	7.16	61	3.7%	3.00 [0.24, 5.76]
Zhang, L. B.2015 7d	-3	9.08	61	-6	7.59	61	3.3%	3.00 [0.03, 5.97]
Zhao, G.2005 3d	-0.07	0.33	30	-0.1	0.38	30	39.3%	0.03 [-0.15, 0.21]
Zhao, G.2005 7d	-0.01	0.37	30	-0.07	0.35	30	39.3%	0.06 [-0.12, 0.24]
Zhou,Y2017C.C.O.	-3.97	15.26	25	0.05	16.33	25	0.4%	-4.02 [-12.78, 4.74]
Zhou,Y2017C.O.	0.67	14.02	25	3.27	13.8	25	0.5%	-2.60 [-10.31, 5.11]
Zhou,Y2017VATS.C.C.O.	-4.81	18.83	25	0.05	16.33	25	0.3%	-4.86 [-14.63, 4.91]
Zhou,Y2017VATS.C.O.	-2.17	16.75	25	3.27	13.8	25	0.4%	-5.44 [-13.95, 3.07]
**Total (95% CI)**			**442**			**442**	**100.0%**	**-0.08 [-0.64, 0.48]**

Heterogeneity: Tau² = 0.20; Chi² = 36.12, df = 12 (P = 0.0003); I² = 67%
Test for overall effect: Z = 0.27 (P = 0.78)

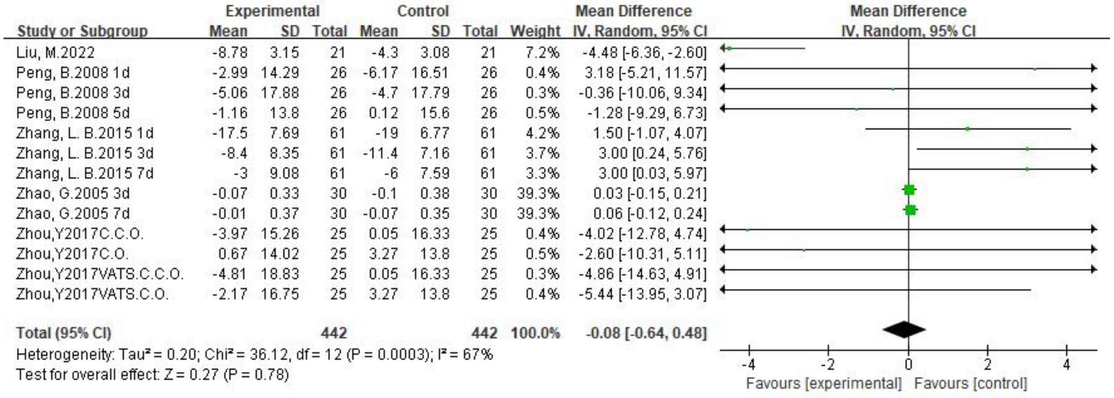

1.2 CD4(Hysteroscopy)

Study or Subgroup	Experimental Mean	Experimental SD	Experimental Total	Control Mean	Control SD	Control Total	Weight	Mean Difference IV, Random, 95% CI
Han, L.2018	-5.81	10.23	83	-1.64	9.46	75	1.5%	-4.17 [-7.24, -1.10]
Liu, M.2022	-3.95	1.67	21	-1.26	1.85	21	9.8%	-2.69 [-3.76, -1.62]
Lv, W.2022 1d	-1.67	11.85	21	-0.03	12.41	21	0.3%	-1.64 [-8.98, 5.70]
Lv, W.2022 1h	-5.05	11.75	21	-1.95	11.55	21	0.3%	-3.10 [-10.15, 3.95]
Lv, W.2022 5d	3.65	11.13	21	2.99	10.88	21	0.3%	0.66 [-6.00, 7.32]
Lv, W.2022 7d	2.6	12.38	21	2.76	10.76	21	0.3%	-0.16 [-7.18, 6.86]
Peng, B.2008 1d	-3.53	10.84	26	-5.76	11.16	26	0.4%	2.23 [-3.75, 8.21]
Peng, B.2008 3d	-3.31	11.98	26	-4.05	10.88	26	0.4%	0.74 [-5.48, 6.96]
Peng, B.2008 5d	2.29	11.66	26	0.46	11.77	26	0.4%	1.83 [-4.54, 8.20]
Zhang, L. B.2015 1d	-11.1	7.94	61	-9.2	8.38	61	1.7%	-1.90 [-4.80, 1.00]
Zhang, L. B.2015 3d	-5.4	8.12	61	-7.1	8.5	61	1.6%	1.70 [-1.25, 4.65]
Zhang, L. B.2015 7d	-1	8.87	61	-2.9	9.2	61	1.4%	1.90 [-1.31, 5.11]
Zhang, L.2016 3d	3.36	9.87	40	5.3	9.31	40	0.8%	-1.94 [-6.14, 2.26]
Zhang, L.2016 7d	3.74	10.45	40	2.97	9.84	40	0.7%	0.77 [-3.68, 5.22]
Zhao, G.2005 3d	-0.03	0.24	30	-0.09	0.23	30	39.7%	0.06 [-0.06, 0.18]
Zhao, G.2005 7d	-0.02	0.27	30	-0.04	0.23	30	39.5%	0.02 [-0.11, 0.15]
Zhou,Y2017C.C.O.	2.28	16.56	25	3.18	12.93	25	0.2%	-0.90 [-9.14, 7.34]
Zhou,Y2017C.O.	0.75	15.99	25	3.65	12.36	25	0.2%	-2.90 [-10.82, 5.02]
Zhou,Y2017VATS.C.C.O.	-0.56	16.38	25	3.18	12.93	25	0.2%	-3.74 [-11.92, 4.44]
Zhou,Y2017VATS.C.O.	-2.19	13.99	25	3.65	12.36	25	0.3%	-5.84 [-13.16, 1.48]
**Total (95% CI)**			**689**			**681**	**100.0%**	**-0.31 [-0.69, 0.07]**

Heterogeneity: Tau² = 0.09; Chi² = 43.33, df = 19 (P = 0.001); I² = 56%
Test for overall effect: Z = 1.58 (P = 0.11)

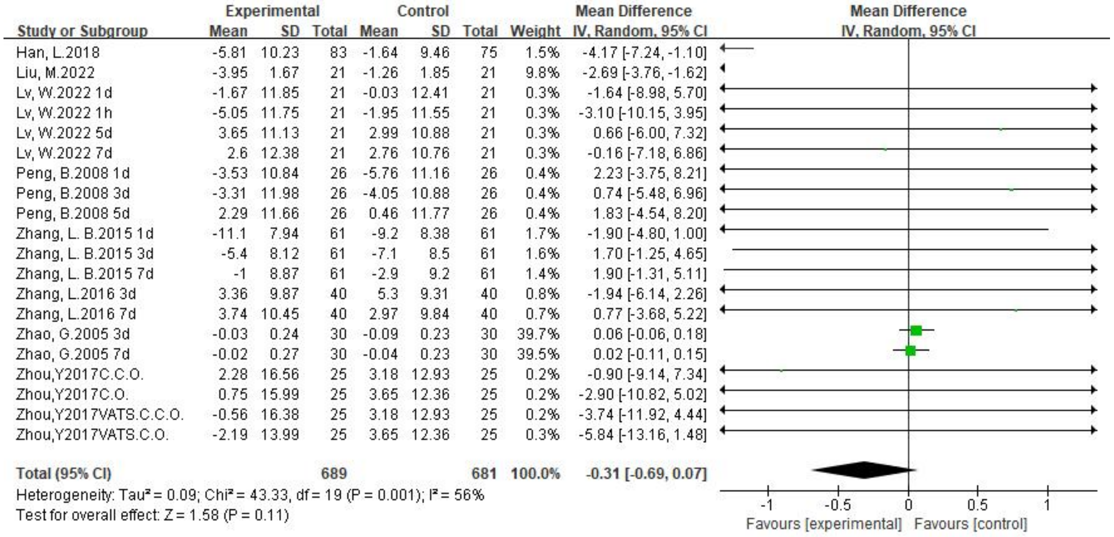

1.3 CD8(Hysteroscopy)

Study or Subgroup	Experimental Mean	Experimental SD	Experimental Total	Control Mean	Control SD	Control Total	Weight	Mean Difference IV, Random, 95% CI
Han, L.2018	-5.38	9.35	83	-2.96	8.94	75	2.2%	-2.42 [-5.27, 0.43]
Liu, M.2022	-5.36	1.8	21	-1.93	2.01	21	10.4%	-3.43 [-4.58, -2.28]
Lv, W.2022 1d	-4.1	10.22	21	-1.74	8.31	21	0.6%	-2.36 [-7.99, 3.27]
Lv, W.2022 1h	-0.24	10.18	21	-0.12	8.9	21	0.6%	-0.12 [-5.90, 5.66]
Lv, W.2022 5d	-0.29	10.66	21	1.26	8.37	21	0.6%	-1.55 [-7.35, 4.25]
Lv, W.2022 7d	-0.27	10.08	21	0.5	8.93	21	0.6%	-0.77 [-6.53, 4.99]
Peng, B.2008 1d	0.09	9.16	26	1.28	14.95	26	0.4%	-1.19 [-7.93, 5.55]
Peng, B.2008 3d	0.92	10.54	26	0.4	15.26	26	0.4%	0.52 [-6.61, 7.65]
Peng, B.2008 5d	-1.5	8.59	26	0.02	14.16	26	0.5%	-1.52 [-7.89, 4.85]
Zhang, L. B.2015 1d	-5.2	7.92	61	-4.7	5.2	61	3.1%	-0.50 [-2.88, 1.88]
Zhang, L. B.2015 3d	-3.4	8.46	61	-3.5	5.26	61	2.8%	0.10 [-2.40, 2.60]
Zhang, L. B.2015 7d	-1.6	9.17	61	-1.8	5.52	61	2.5%	0.20 [-2.49, 2.89]
Zhang, L.2016 3d	1.89	11.02	40	5.08	9.96	40	0.9%	-3.19 [-7.79, 1.41]
Zhang, L.2016 7d	-1.24	11.41	40	4.89	10.21	40	0.8%	-6.13 [-10.87, -1.39]
Zhao, G.2005 3d	0.01	0.15	30	0.2	0.4	30	36.1%	-0.19 [-0.34, -0.04]
Zhao, G.2005 7d	0	0.16	30	0.21	0.4	30	36.1%	-0.21 [-0.36, -0.06]
Zhou,Y2017C.C.O.	-5.81	12.51	25	-2.85	12.7	25	0.4%	-2.96 [-9.95, 4.03]
Zhou,Y2017C.O.	-0.76	13.24	25	-0.48	12.64	25	0.4%	-0.28 [-7.46, 6.90]
Zhou,Y2017VATS.C.C.O.	-5.13	15.53	25	-2.85	12.7	25	0.3%	-2.28 [-10.14, 5.58]
Zhou,Y2017VATS.C.O.	0.53	15.25	25	-0.48	12.64	25	0.3%	1.01 [-6.75, 8.77]
**Total (95% CI)**			**689**			**681**	**100.0%**	**-0.70 [-1.14, -0.26]**

Heterogeneity: Tau² = 0.13; Chi² = 41.86, df = 19 (P = 0.002); I² = 55%
Test for overall effect: Z = 3.12 (P = 0.002)

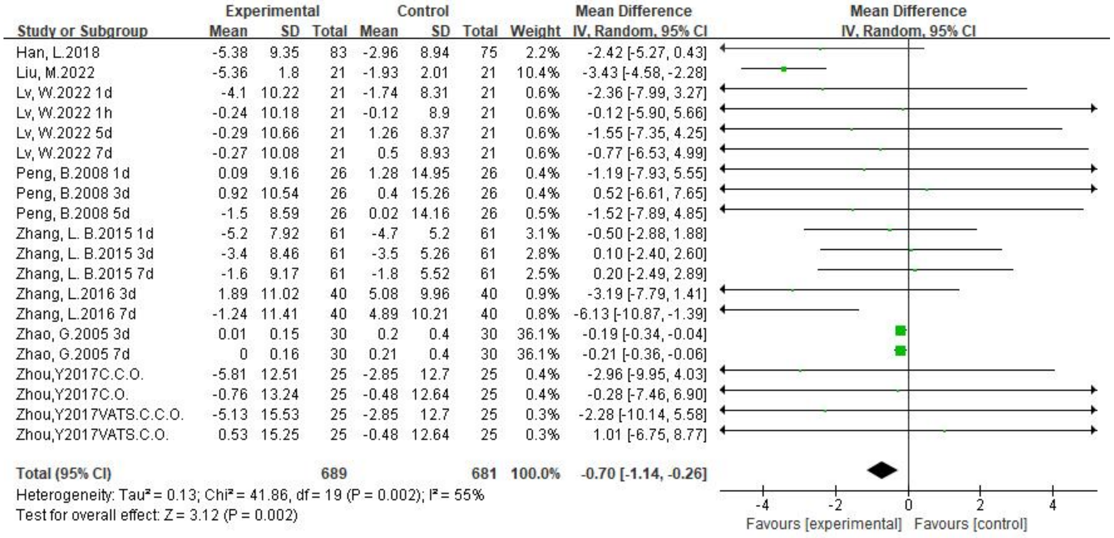

1.4 CD4/CD8(Hysteroscopy)

Study or Subgroup	Experimental Mean	Experimental SD	Experimental Total	Control Mean	Control SD	Control Total	Weight	Mean Difference IV, Random, 95% CI
Liu, M.2022	0.05	0.07	21	0.01	0.05	21	21.7%	0.04 [0.00, 0.08]
Zhang, L. B.2015 1d	-0.1	0.14	61	-0.2	0.22	61	19.7%	0.10 [0.03, 0.17]
Zhang, L. B.2015 3d	0	0.14	61	-0.2	0.22	61	19.7%	0.20 [0.13, 0.27]
Zhang, L. B.2015 7d	0.1	0.22	61	-0.1	0.22	61	18.6%	0.20 [0.12, 0.28]
Zhao, G.2005 3d	-0.07	0.51	30	0.03	0.47	30	7.1%	-0.10 [-0.35, 0.15]
Zhao, G.2005 7d	-0.09	0.44	30	0.07	0.54	30	7.0%	-0.16 [-0.41, 0.09]
Zhou,Y2017C.C.O.	0.64	1.37	25	0.36	1.12	25	1.2%	0.28 [-0.41, 0.97]
Zhou,Y2017C.O.	0.08	0.93	25	0.19	1.13	25	1.8%	-0.11 [-0.68, 0.46]
Zhou,Y2017VATS.C.C.O.	0.36	1.18	25	0.36	1.12	25	1.5%	0.00 [-0.64, 0.64]
Zhou,Y2017VATS.C.O.	-0.18	0.97	25	0.19	1.13	25	1.7%	-0.37 [-0.95, 0.21]
**Total (95% CI)**			**364**			**364**	**100.0%**	**0.08 [0.00, 0.16]**

Heterogeneity: Tau² = 0.01; Chi² = 35.00, df = 9 (P < 0.0001); I² = 74%
Test for overall effect: Z = 2.01 (P = 0.04)

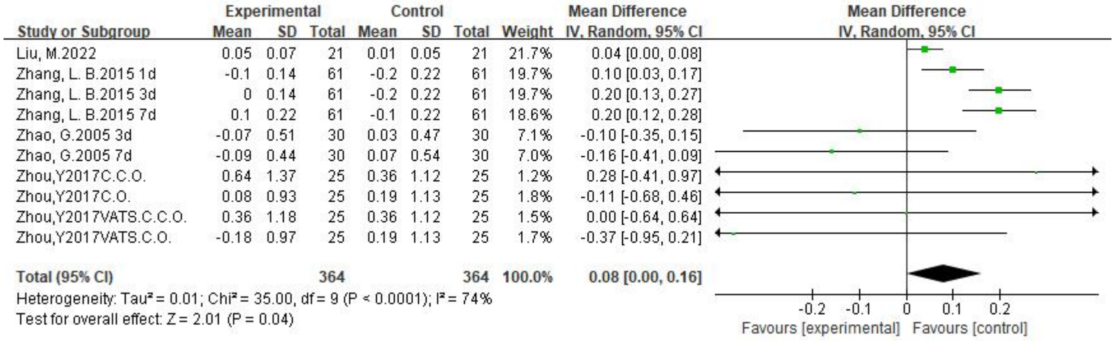

1.5 NK(Hysteroscopy)

Study or Subgroup	Experimental Mean	Experimental SD	Experimental Total	Control Mean	Control SD	Control Total	Weight	Mean Difference IV, Fixed, 95% CI
Zhang, L.2016 3d	-8.88	9.41	40	-6.83	10.44	40	52.4%	-2.05 [-6.41, 2.31]
Zhang, L.2016 7d	-6.66	9.94	40	-4.71	10.9	40	47.6%	-1.95 [-6.52, 2.62]
**Total (95% CI)**			**80**			**80**	**100.0%**	**-2.00 [-5.16, 1.15]**

Heterogeneity: Chi² = 0.00, df = 1 (P = 0.98); I² = 0%
Test for overall effect: Z = 1.24 (P = 0.21)

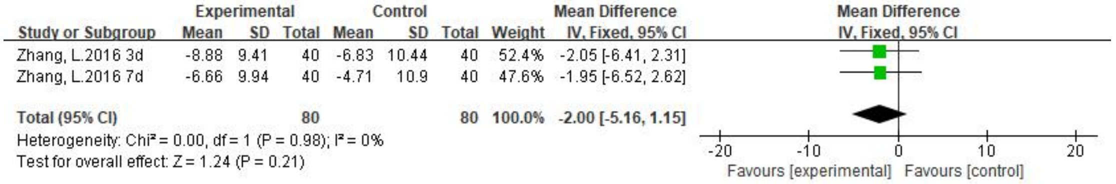

1.6CD3(Immune)

Study or Subgroup	Experimental Mean	Experimental SD	Experimental Total	Control Mean	Control SD	Control Total	Weight	Mean Difference IV, Random, 95% CI
Huang, X.2010 8d	1.54	17.83	25	0.97	13.21	25	26.9%	0.57 [-8.13, 9.27]
Liu, Z.2011 1d	-5.4	15.85	21	-0.4	8.7	21	33.0%	-5.00 [-12.73, 2.73]
Liu, Z.2011 9d	1.3	13.5	21	-1.7	8.72	21	40.2%	3.00 [-3.87, 9.87]
**Total (95% CI)**			**67**			**67**	**100.0%**	**-0.29 [-5.09, 4.51]**

Heterogeneity: Tau² = 2.66; Chi² = 2.34, df = 2 (P = 0.31); I² = 15%
Test for overall effect: Z = 0.12 (P = 0.91)

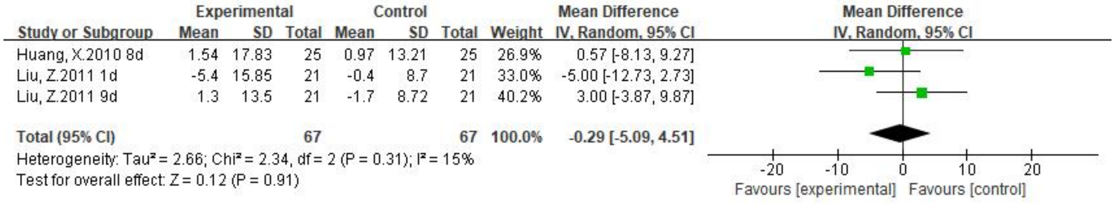

1.7 CD4(Immune)

Study or Subgroup	Experimental Mean	Experimental SD	Experimental Total	Control Mean	Control SD	Control Total	Weight	Std. Mean Difference IV, Random, 95% CI
Huang, X.2010 8d	1.17	10.9	25	0.93	11.1	25	9.8%	0.02 [-0.53, 0.58]
Liu, H.2011 10d	-1.74	13.43	28	-10.55	13.27	28	9.8%	0.65 [0.11, 1.19]
Liu, H.20127d	9.27	13.13	28	-1.95	10.26	26	9.7%	0.93 [0.37, 1.50]
Liu, Z.2011 1d	-5.5	10.48	21	-0.4	9.22	21	9.5%	-0.51 [-1.12, 0.11]
Liu, Z.2011 9d	1.7	9.87	21	2.8	9.65	21	9.5%	-0.11 [-0.72, 0.49]
Marano, L.20131d	462	71.55	54	422	100.18	55	10.5%	0.46 [0.08, 0.84]
Marano, L.20137d	-171	78.24	54	-59	103.59	55	10.4%	-1.21 [-1.62, -0.80]
Zhao, H.2013 7d	9.18	12.29	37	1.36	11.71	36	10.1%	0.64 [0.17, 1.12]
Zong, L.2019 1d	0.56	10.6	48	-10.81	9.37	48	10.3%	1.13 [0.70, 1.56]
Zong, L.2019 7d	0.57	10.62	48	-9.09	11.1	48	10.3%	0.88 [0.46, 1.30]
**Total (95% CI)**			**364**			**363**	**100.0%**	**0.29 [-0.20, 0.78]**

Heterogeneity: Tau² = 0.56; Chi² = 92.20, df = 9 (P < 0.00001); I² = 90%
Test for overall effect: Z = 1.17 (P = 0.24)

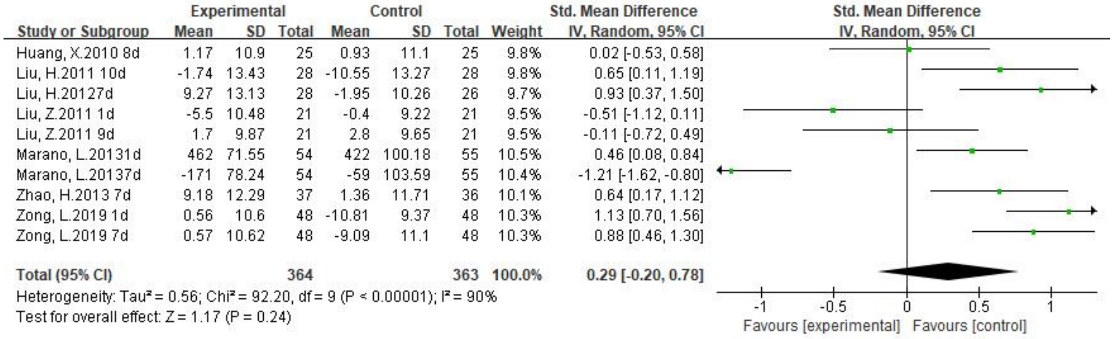

1.8 CD8(Immune)

Study or Subgroup	Experimental Mean	Experimental SD	Experimental Total	Control Mean	Control SD	Control Total	Weight	Std. Mean Difference IV, Fixed, 95% CI
Huang, X.2010 8d	-3.48	8.28	25	-1.42	10.9	25	6.9%	-0.21 [-0.77, 0.35]
Liu, H.2011 10d	-0.97	9.94	28	-5.78	9.91	28	7.5%	0.48 [-0.05, 1.01]
Liu, H.20127d	3.05	8.32	28	-0.29	7.07	28	7.6%	0.43 [-0.10, 0.96]
Liu, Z.2011 1d	-0.1	9.9	21	-1.2	9.17	21	5.8%	0.11 [-0.49, 0.72]
Liu, Z.2011 9d	1.6	7.79	21	-0.2	8.25	21	5.8%	0.22 [-0.39, 0.83]
Marano, L.20131d	222	69.4	54	201	84.5	54	14.9%	0.27 [-0.11, 0.65]
Marano, L.20137d	-62	55.23	54	-56	96.26	54	15.0%	-0.08 [-0.45, 0.30]
Zhao, H.2013 7d	1.09	8.72	37	-1.42	9	36	10.0%	0.28 [-0.18, 0.74]
Zong, L.2019 1d	-0.77	10.68	48	-3.62	9.77	48	13.2%	0.28 [-0.13, 0.68]
Zong, L.2019 7d	-0.65	10.73	48	-2.6	10.18	48	13.3%	0.18 [-0.22, 0.59]
**Total (95% CI)**			**364**			**363**	**100.0%**	**0.19 [0.05, 0.34]**

Heterogeneity: Chi² = 6.35, df = 9 (P = 0.70); I² = 0%
Test for overall effect: Z = 2.56 (P = 0.01)

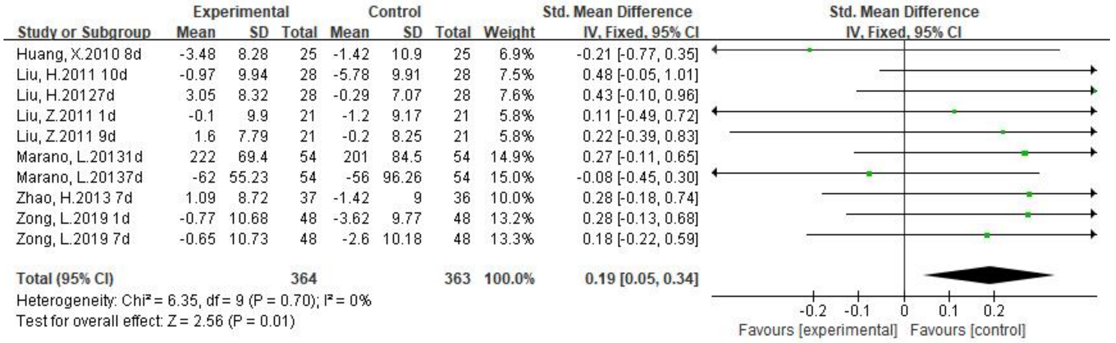

1.9 CD4/CD8(Immune)

Study or Subgroup	Experimental Mean	Experimental SD	Experimental Total	Control Mean	Control SD	Control Total	Weight	Mean Difference IV, Fixed, 95% CI
Huang, X.2010 8d	0.25	0.32	25	0.05	0.37	25	34.3%	0.20 [0.01, 0.39]
Liu, H.2011 10d	-0.04	0.31	28	-0.21	0.34	28	43.4%	0.17 [-0.00, 0.34]
Liu, Z.2011 1d	-0.1	1.06	21	-0.05	0.88	21	3.6%	-0.05 [-0.64, 0.54]
Liu, Z.2011 9d	0.5	1.06	21	-0.34	0.75	21	4.1%	0.84 [0.28, 1.40]
Zong, L.2019 1d	-0.08	1.04	48	-0.37	1	48	7.6%	0.29 [-0.12, 0.70]
Zong, L.2019 7d	-0.06	1.08	48	-0.21	1.05	48	6.9%	0.15 [-0.28, 0.58]
**Total (95% CI)**			**191**			**191**	**100.0%**	**0.21 [0.10, 0.32]**

Heterogeneity: Chi² = 6.14, df = 5 (P = 0.29); I² = 19%
Test for overall effect: Z = 3.62 (P = 0.0003)

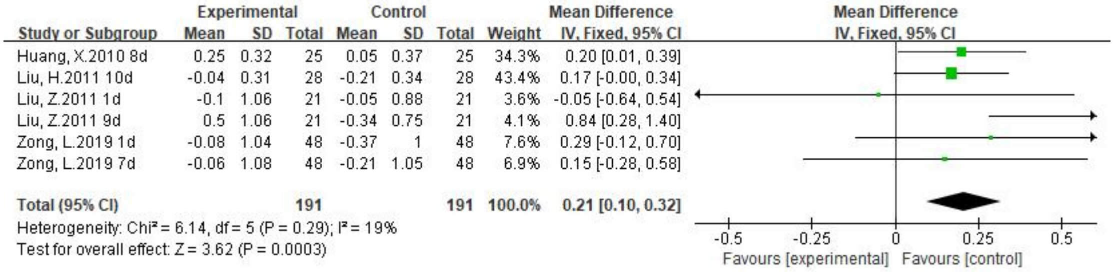

1.10 CD3(Dexmedetomidine)

Study or Subgroup	Experimental Mean	Experimental SD	Experimental Total	Control Mean	Control SD	Control Total	Weight	Mean Difference IV, Random, 95% CI
Ai, Z.2023 1d	-2.65	4.96	52	-6.9	4.58	52	8.0%	4.25 [2.42, 6.08]
Ai, Z.2023 3d	-1.23	4.62	52	-4.25	4.59	52	8.1%	3.02 [1.25, 4.79]
Bai, Y.2020 12hDEX	-6	9.6	100	-10.13	10.29	100	7.3%	4.13 [1.37, 6.89]
Bai, Y.2020 12hDEX+OXY	-2.94	9.43	100	-7.72	9.58	100	7.4%	4.78 [2.15, 7.41]
Bai, Y.2020 24hDEX	-8.96	9.85	100	-10.13	10.29	100	7.3%	1.17 [-1.62, 3.96]
Bai, Y.2020 24hDEX+OXY	-8.09	9.48	100	-7.72	9.58	100	7.4%	-0.37 [-3.01, 2.27]
Wang, K.2018 0.5h	-3.6	7.99	72	-1.8	8.15	69	7.4%	-1.80 [-4.47, 0.87]
Wang, K.2018 1d	-4.7	7.99	72	-5	8.15	69	7.4%	0.30 [-2.37, 2.97]
Wang, K.2018 2d	-3.5	7.99	72	-7.3	8.15	69	7.4%	3.80 [1.13, 6.47]
Wang, K.2018 3d	-1.8	7.99	72	-0.3	8.15	69	7.4%	-1.50 [-4.17, 1.17]
Zong, S.2021 12h	-11.71	7.99	30	-22.39	8.15	30	6.2%	10.68 [6.60, 14.76]
Zong, S.2021 1d	-13.96	7.99	30	-26.49	8.15	30	6.2%	12.53 [8.45, 16.61]
Zong, S.2021 1w	-9.12	7.99	30	-16.73	8.15	30	6.2%	7.61 [3.53, 11.69]
Zong, S.2021 2d	0.87	7.99	30	-7.15	8.15	30	6.2%	8.02 [3.94, 12.10]
**Total (95% CI)**			**912**			**900**	**100.0%**	**3.76 [1.88, 5.63]**

Heterogeneity: Tau² = 10.50; Chi² = 84.96, df = 13 (P < 0.00001); I² = 85%
Test for overall effect: Z = 3.93 (P < 0.0001)

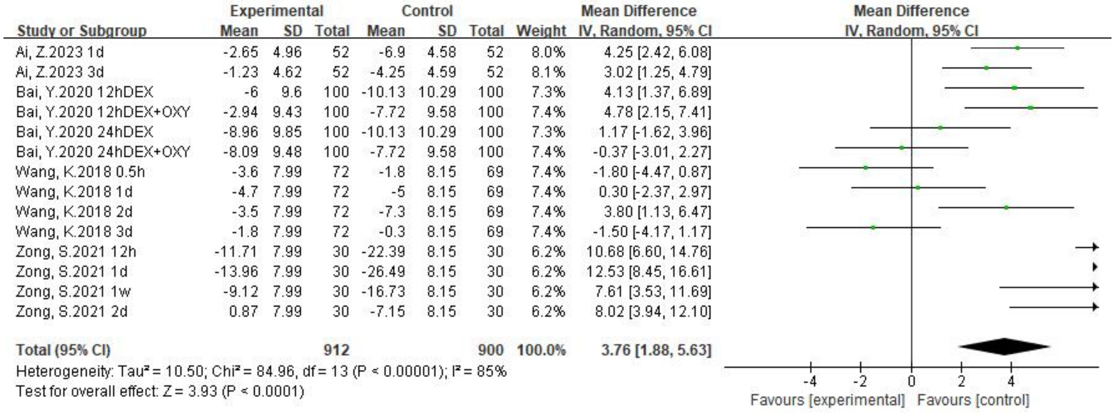

1.11 CD4(Dexmedetomidine)

Study or Subgroup	Experimental Mean	Experimental SD	Experimental Total	Control Mean	Control SD	Control Total	Weight	Mean Difference IV, Random, 95% CI
Ai, Z.2023 1d	-0.41	4.37	52	-6.38	3.81	52	7.2%	5.97 [4.39, 7.55]
Ai, Z.2023 3d	2.59	3.99	52	-4.02	3.76	52	7.2%	6.61 [5.12, 8.10]
Bai, Y.2020 12hDEX	-4.61	8.43	100	-8.2	8.51	100	7.0%	3.59 [1.24, 5.94]
Bai, Y.2020 12hDEX+OXY	-2.48	7.67	100	-6.99	8.07	100	7.1%	4.51 [2.33, 6.69]
Bai, Y.2020 24hDEX	-7.62	8.66	100	-8.2	8.51	100	7.0%	0.58 [-1.80, 2.96]
Bai, Y.2020 24hDEX+OXY	-6.02	8.38	100	-6.99	8.07	100	7.1%	0.97 [-1.31, 3.25]
Li, A.2018 1d	-11	5.11	59	-0.08	0.3	59	7.3%	-10.92 [-12.23, -9.61]
Li, A.2018 1h	-6.6	5.23	59	-9.06	5.26	59	7.2%	2.46 [0.57, 4.35]
Li, A.2018 2d	-3.73	4.75	59	-6.7	4.84	59	7.2%	2.97 [1.24, 4.70]
Li, A.2018 3d	-2.2	5.02	59	-4.98	4.89	59	7.2%	2.78 [0.99, 4.57]
Wang, K.2018 0.5h	-0.7	6.16	72	-2.2	5.6	69	7.1%	1.50 [-0.44, 3.44]
Wang, K.2018 1d	-1.4	6.16	72	-4.1	5.6	69	7.1%	2.70 [0.76, 4.64]
Wang, K.2018 2d	-1.8	6.16	72	-4	5.6	69	7.1%	2.20 [0.26, 4.14]
Wang, K.2018 3d	-1.2	6.16	72	0	5.6	69	7.1%	-1.20 [-3.14, 0.74]
**Total (95% CI)**			**1028**			**1016**	**100.0%**	**1.76 [-1.13, 4.64]**

Heterogeneity: Tau² = 29.42; Chi² = 447.66, df = 13 (P < 0.00001); I² = 97%
Test for overall effect: Z = 1.19 (P = 0.23)

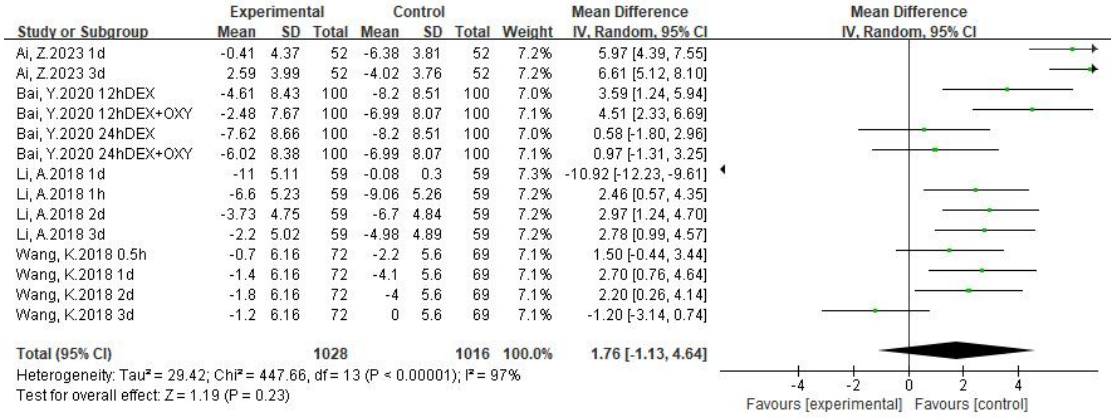

1.12CD8(Dexmedetomidine)

Study or Subgroup	Experimental Mean	Experimental SD	Experimental Total	Control Mean	Control SD	Control Total	Weight	Mean Difference IV, Random, 95% CI
Ai, Z.2023 1d	-2.22	1.96	52	-0.57	2.35	52	8.5%	-1.65 [-2.48, -0.82]
Ai, Z.2023 3d	-3.83	1.89	52	-0.25	2.74	52	8.4%	-3.58 [-4.48, -2.68]
Bai, Y.2020 12hDEX	0.46	8.12	100	3.42	7.31	100	6.1%	-2.96 [-5.10, -0.82]
Bai, Y.2020 12hDEX+OXY	-0.41	6.98	100	-0.44	6.11	100	6.7%	0.03 [-1.79, 1.85]
Bai, Y.2020 24hDEX	4.71	9.1	100	3.42	7.31	100	5.8%	1.29 [-1.00, 3.58]
Bai, Y.2020 24hDEX+OXY	1.54	7.97	100	-0.44	6.11	100	6.4%	1.98 [0.01, 3.95]
Li, A.2018 1d	8.93	3.84	59	8.29	4.27	59	7.4%	0.64 [-0.83, 2.11]
Li, A.2018 1h	4.13	3.29	59	7.17	4.51	59	7.5%	-3.04 [-4.46, -1.62]
Li, A.2018 2d	2.22	3.92	59	5	4.14	59	7.4%	-2.78 [-4.23, -1.33]
Li, A.2018 3d	0.8	3.82	59	3.39	3.66	59	7.6%	-2.59 [-3.94, -1.24]
Wang, K.2018 0.5h	1.1	5.09	72	2.1	4.85	69	7.1%	-1.00 [-2.64, 0.64]
Wang, K.2018 1d	1.2	5.09	72	2.6	4.85	69	7.1%	-1.40 [-3.04, 0.24]
Wang, K.2018 2d	1.4	5.09	72	2.7	4.85	69	7.1%	-1.30 [-2.94, 0.34]
Wang, K.2018 3d	0	5.09	72	-0.9	4.85	69	7.1%	0.90 [-0.74, 2.54]
**Total (95% CI)**			**1028**			**1016**	**100.0%**	**-1.20 [-2.10, -0.30]**

Heterogeneity: Tau² = 2.30; Chi² = 68.97, df = 13 (P < 0.00001); I² = 81%
Test for overall effect: Z = 2.61 (P = 0.009)

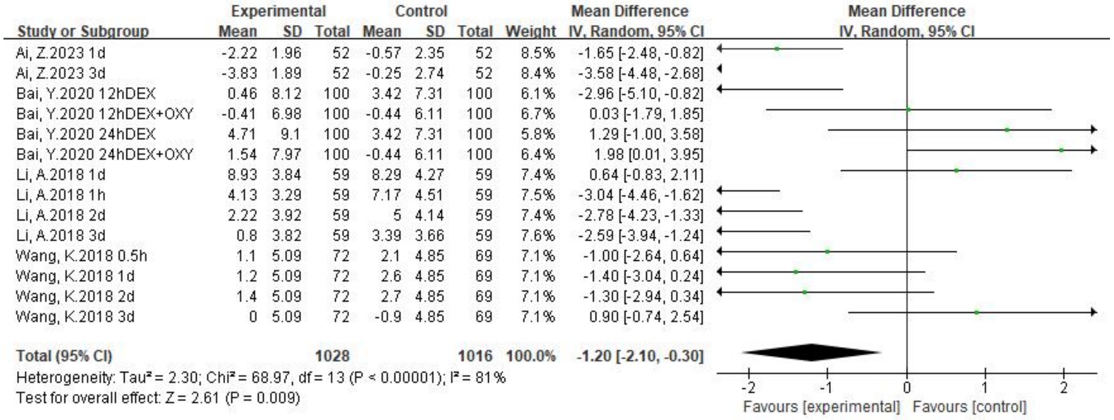

1.13 CD4/CD8(Dexmedetomidine)

Study or Subgroup	Experimental Mean	SD	Total	Control Mean	SD	Total	Weight	Mean Difference IV, Random, 95% CI
Ai, Z.2023 1d	0.09	0.16	52	-0.18	0.15	52	6.0%	0.27 [0.21, 0.33]
Ai, Z.2023 3d	0.28	0.15	52	-0.11	0.17	52	6.0%	0.39 [0.33, 0.45]
Bai, Y.2020 12hDEX	-0.17	0.48	100	-0.32	0.47	100	5.6%	0.15 [0.02, 0.28]
Bai, Y.2020 12hDEX+OXY	-0.12	0.39	100	-0.25	0.77	100	5.3%	0.13 [-0.04, 0.30]
Bai, Y.2020 24hDEX	-0.41	0.85	100	-0.32	0.47	100	5.1%	-0.09 [-0.28, 0.10]
Bai, Y.2020 24hDEX+OXY	-0.35	0.56	100	-0.25	0.77	100	5.1%	-0.10 [-0.29, 0.09]
Li, A.2018 1d	-0.84	0.32	59	-0.78	0.18	59	5.8%	-0.06 [-0.15, 0.03]
Li, A.2018 1h	-0.52	0.028	59	-0.71	0.21	59	6.0%	0.19 [0.14, 0.24]
Li, A.2018 2d	-0.32	0.28	59	-0.52	0.17	59	5.9%	0.20 [0.12, 0.28]
Li, A.2018 3d	-0.08	0.3	59	-0.31	0.24	59	5.8%	0.23 [0.13, 0.33]
Wang, K.2018 0.5h	-0.3	0.35	72	-0.1	0.36	69	5.7%	-0.20 [-0.32, -0.08]
Wang, K.2018 1d	-0.4	0.35	72	-0.4	0.36	69	5.7%	0.00 [-0.12, 0.12]
Wang, K.2018 2d	-0.2	0.35	72	-0.4	0.36	69	5.7%	0.20 [0.08, 0.32]
Wang, K.2018 3d	-0.4	0.35	72	0	0.36	69	5.7%	-0.40 [-0.52, -0.28]
Zong, S.2021 12h	0.19	0.35	30	-0.48	0.36	30	5.2%	0.67 [0.49, 0.85]
Zong, S.2021 1d	0.16	0.35	30	-0.64	0.36	30	5.2%	0.80 [0.62, 0.98]
Zong, S.2021 1w	0.13	0.35	30	-0.33	0.36	30	5.2%	0.46 [0.28, 0.64]
Zong, S.2021 2d	0.17	0.35	30	-0.19	0.36	30	5.2%	0.36 [0.18, 0.54]
**Total (95% CI)**			**1148**			**1136**	**100.0%**	**0.17 [0.07, 0.28]**

Heterogeneity: Tau² = 0.05; Chi² = 327.46, df = 17 (P < 0.00001); I² = 95%
Test for overall effect: Z = 3.17 (P = 0.002)

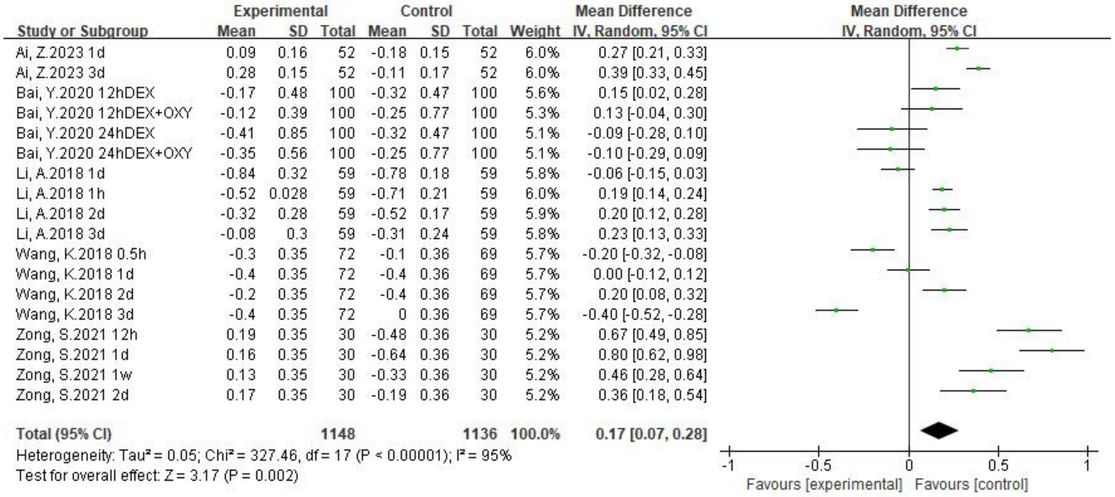

1.14 NK(Dexmedetomidine)

Study or Subgroup	Experimental Mean	SD	Total	Control Mean	SD	Total	Weight	Mean Difference IV, Random, 95% CI
Bai, Y.2020 12hDEX	-1.69	6.67	100	-4.01	6.3	100	23.6%	2.32 [0.52, 4.12]
Bai, Y.2020 12hDEX+OXY	-0.32	5.32	100	-2.72	5.4	100	26.6%	2.40 [0.91, 3.89]
Bai, Y.2020 24hDEX	-4.12	6.51	100	-4.01	6.3	100	23.9%	-0.11 [-1.89, 1.67]
Bai, Y.2020 24hDEX+OXY	-3.02	5.87	100	-2.72	5.4	100	25.9%	-0.30 [-1.86, 1.26]
**Total (95% CI)**			**400**			**400**	**100.0%**	**1.08 [-0.39, 2.56]**

Heterogeneity: Tau² = 1.55; Chi² = 9.57, df = 3 (P = 0.02); I² = 69%
Test for overall effect: Z = 1.44 (P = 0.15)

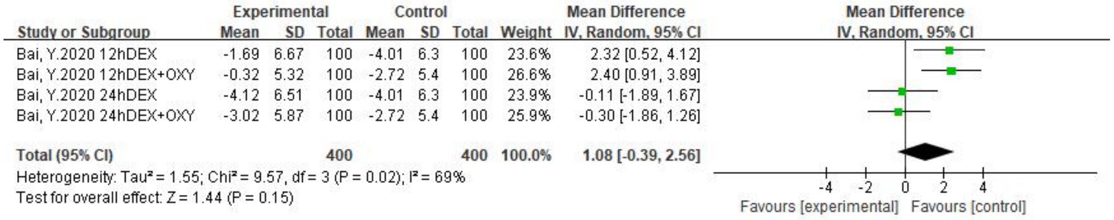

1.15 CD3(ERAS)

Study or Subgroup	Experimental Mean	SD	Total	Control Mean	SD	Total	Weight	Mean Difference IV, Random, 95% CI
Chen, L.2016 1d	-6.07	3.87	128	-11.82	3.87	132	29.3%	5.75 [4.81, 6.69]
Chen, L.2016 3d	-4.16	3.64	128	-9.81	3.64	132	29.6%	5.65 [4.76, 6.54]
Chen, L.2016 7d	-0.94	3.83	128	-4	3.78	132	29.4%	3.06 [2.13, 3.99]
Ding, H.2020	-1.46	9.41	42	-7.84	8.95	58	11.8%	6.38 [2.72, 10.04]
**Total (95% CI)**			**426**			**454**	**100.0%**	**5.00 [3.43, 6.58]**

Heterogeneity: Tau² = 1.96; Chi² = 21.70, df = 3 (P < 0.0001); I² = 86%
Test for overall effect: Z = 6.25 (P < 0.00001)

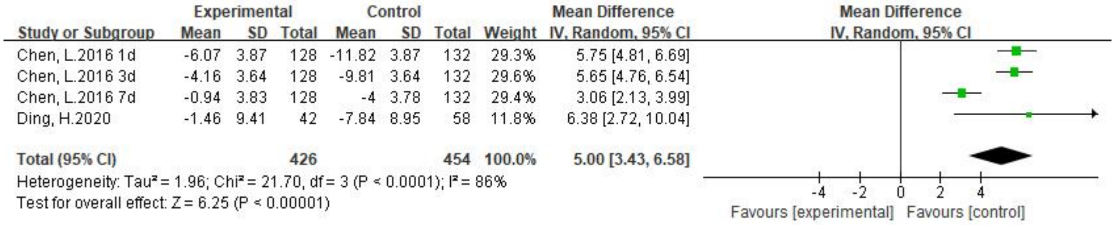

1.16 CD4(ERAS)

Study or Subgroup	Experimental Mean	SD	Total	Control Mean	SD	Total	Weight	Mean Difference IV, Random, 95% CI
Chen, L.2016 1d	-12.32	6.14	128	-14.6	6.77	132	26.2%	2.28 [0.71, 3.85]
Chen, L.2016 3d	-6.19	6.21	128	-10.63	7.17	132	26.0%	4.44 [2.81, 6.07]
Chen, L.2016 7d	-1.82	5.88	128	-2.1	5.87	132	26.7%	0.28 [-1.15, 1.71]
Ding, H.2020	-1.12	7.22	42	-7.38	6.63	58	21.1%	6.26 [3.49, 9.03]
**Total (95% CI)**			**426**			**454**	**100.0%**	**3.14 [0.78, 5.51]**

Heterogeneity: Tau² = 4.91; Chi² = 21.87, df = 3 (P < 0.0001); I² = 86%
Test for overall effect: Z = 2.61 (P = 0.009)

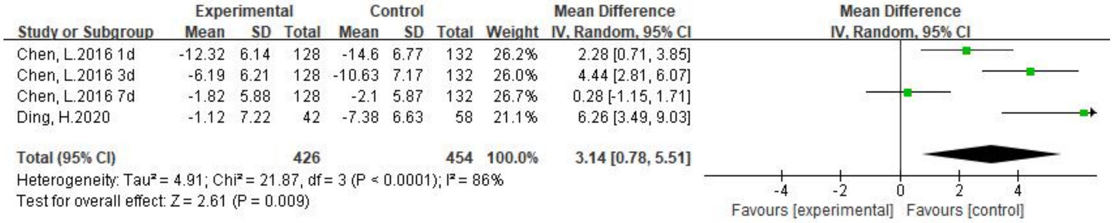

1.17 CD8(ERAS)

Study or Subgroup	Experimental Mean	SD	Total	Control Mean	SD	Total	Weight	Mean Difference IV, Fixed, 95% CI
Chen, L.2016 1d	-3.01	6.5	128	-1.66	6.18	132	31.6%	-1.35 [-2.89, 0.19]
Chen, L.2016 3d	-2.97	6.18	128	-3.35	6.11	132	33.7%	0.38 [-1.11, 1.87]
Chen, L.2016 7d	2	6.54	128	1.54	5.52	132	34.7%	0.46 [-1.01, 1.93]
**Total (95% CI)**			**384**			**396**	**100.0%**	**-0.14 [-1.01, 0.73]**

Heterogeneity: Chi² = 3.47, df = 2 (P = 0.18); I² = 42%
Test for overall effect: Z = 0.31 (P = 0.75)

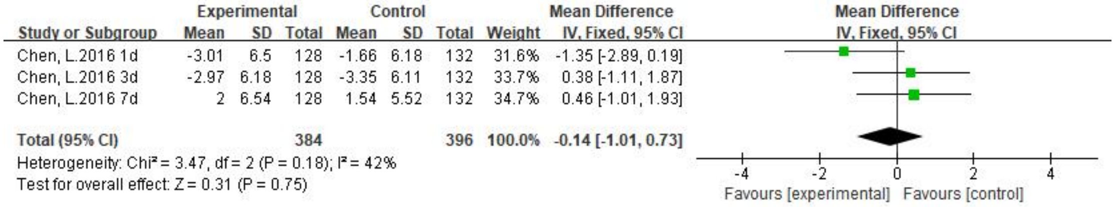

1.18 CD4/CD8(ERAS)

Study or Subgroup	Experimental Mean	Experimental SD	Experimental Total	Control Mean	Control SD	Control Total	Weight	Mean Difference IV, Random, 95% CI
Chen, L.2016 1d	-0.08	0.45	128	-0.26	0.62	132	26.6%	0.18 [0.05, 0.31]
Chen, L.2016 3d	-0.01	0.56	128	-0.09	0.52	132	26.5%	0.08 [-0.05, 0.21]
Chen, L.2016 7d	0.05	0.46	128	0.05	0.68	132	25.3%	0.00 [-0.14, 0.14]
Ding, H.2020	-0.05	0.47	42	-0.35	0.37	58	21.6%	0.30 [0.13, 0.47]
**Total (95% CI)**			**426**			**454**	**100.0%**	**0.13 [0.02, 0.25]**

Heterogeneity: Tau² = 0.01; Chi² = 8.17, df = 3 (P = 0.04); I² = 63%
Test for overall effect: Z = 2.23 (P = 0.03)

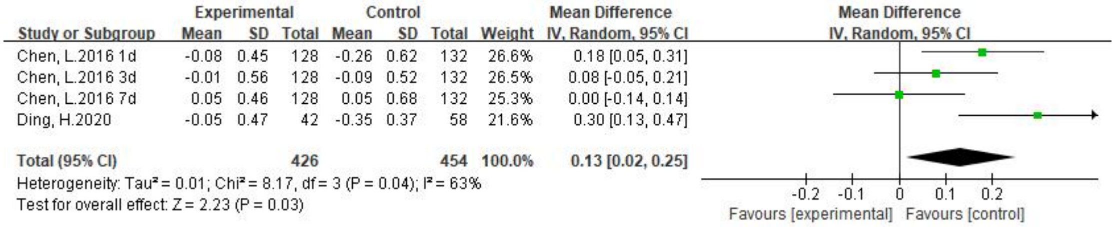

1.19 CD3(Psychotherapy)

Study or Subgroup	Experimental Mean	Experimental SD	Experimental Total	Control Mean	Control SD	Control Total	Weight	Mean Difference IV, Fixed, 95% CI
Lengacher, C. A.2013	37	504.43	40	22	428.03	42	0.1%	15.00 [-187.96, 217.96]
Zhao, X.2016	3	12.92	62	3.93	15.83	62	99.9%	-0.93 [-6.02, 4.16]
**Total (95% CI)**			**102**			**104**	**100.0%**	**-0.92 [-6.00, 4.16]**

Heterogeneity: Chi² = 0.02, df = 1 (P = 0.88); I² = 0%
Test for overall effect: Z = 0.35 (P = 0.72)

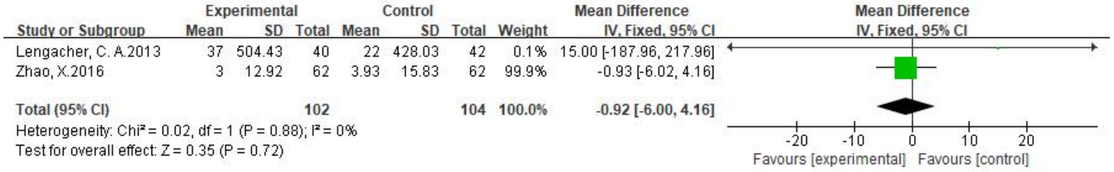

1.20 CD4(Psychotherapy)

Study or Subgroup	Experimental Mean	Experimental SD	Experimental Total	Control Mean	Control SD	Control Total	Weight	Mean Difference IV, Fixed, 95% CI
Lengacher, C. A.2013	75	377.6	40	14	294.24	42	0.1%	61.00 [-86.01, 208.01]
Zhao, X.2016	4.12	11.33	62	2.84	12.57	62	99.9%	1.28 [-2.93, 5.49]
**Total (95% CI)**			**102**			**104**	**100.0%**	**1.33 [-2.88, 5.54]**

Heterogeneity: Chi² = 0.63, df = 1 (P = 0.43); I² = 0%
Test for overall effect: Z = 0.62 (P = 0.54)

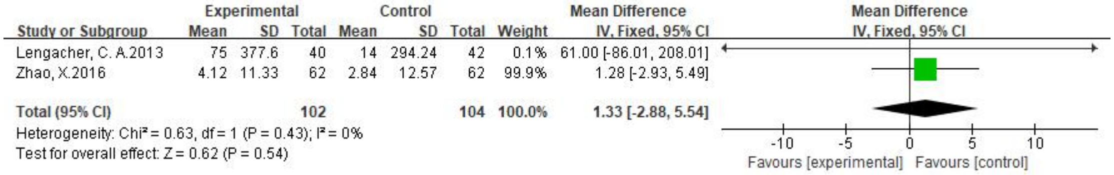

1.21 CD8(Psychotherapy)

Study or Subgroup	Experimental Mean	Experimental SD	Experimental Total	Control Mean	Control SD	Control Total	Weight	Mean Difference IV, Fixed, 95% CI
Lengacher, C. A.2013	-7	231.28	40	18	244.29	42	0.2%	-25.00 [-127.93, 77.93]
Zhao, X.2016	-1.22	12.04	62	0.99	11.85	62	99.8%	-2.21 [-6.42, 2.00]
**Total (95% CI)**			**102**			**104**	**100.0%**	**-2.25 [-6.45, 1.95]**

Heterogeneity: Chi² = 0.19, df = 1 (P = 0.66); I² = 0%
Test for overall effect: Z = 1.05 (P = 0.29)

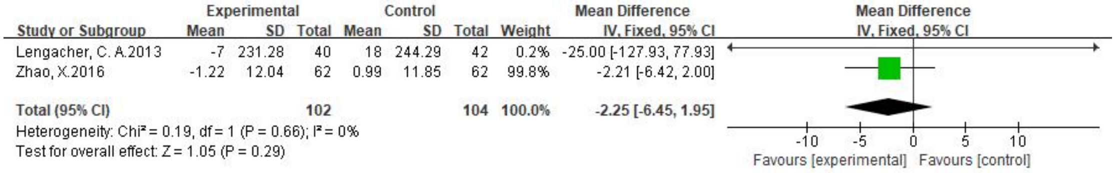

1.22 CD4/CD8(Psychotherapy)

Study or Subgroup	Experimental Mean	Experimental SD	Experimental Total	Control Mean	Control SD	Control Total	Weight	Mean Difference IV, Fixed, 95% CI
Lengacher, C. A.2013	1	24.95	40	0.5	29.33	42	0.1%	0.50 [-11.27, 12.27]
Zhao, X.2016	0.22	1	62	-0.04	1.21	62	99.9%	0.26 [-0.13, 0.65]
**Total (95% CI)**			**102**			**104**	**100.0%**	**0.26 [-0.13, 0.65]**

Heterogeneity: Chi² = 0.00, df = 1 (P = 0.97); I² = 0%
Test for overall effect: Z = 1.31 (P = 0.19)

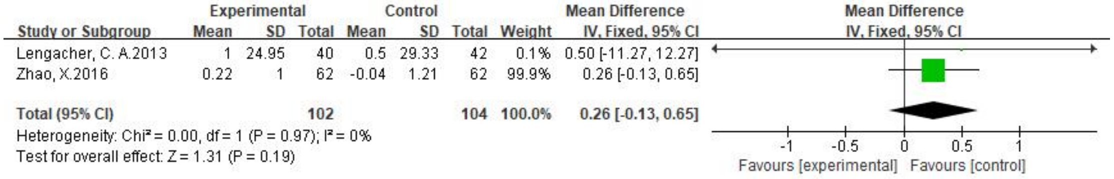

1.23 CD3(TCM)

Study or Subgroup	Experimental Mean	Experimental SD	Experimental Total	Control Mean	Control SD	Control Total	Weight	Mean Difference IV, Random, 95% CI
Cai, J.2008 10d	-14.69	9.82	21	-15.69	11.98	20	10.1%	1.00 [-5.72, 7.72]
Cai, J.2008 1d	-5.51	11.39	21	-64.68	9.58	20	10.1%	59.17 [52.74, 65.60]
Dai, C. M.2021	12.46	9.16	39	7.09	9.95	40	10.3%	5.37 [1.15, 9.59]
Huang, Z. R.2019 14d	-7.69	19.09	30	-7.76	18.48	30	9.7%	0.07 [-9.44, 9.58]
Huang, Z. R.2019 2d	-0.2	19.59	30	-4.88	19.3	30	9.7%	4.68 [-5.16, 14.52]
Wang, J. Y.2007 3d	-1.7	15.19	30	-1.8	16.04	30	9.9%	0.10 [-7.81, 8.01]
Wang, J. Y.2007 9d	4	13.37	30	0.7	13.55	30	10.1%	3.30 [-3.51, 10.11]
Wu, B.2007 10d	-13.05	15.87	20	-14.69	9.82	20	9.9%	1.64 [-6.54, 9.82]
Wu, B.2007 1d	0.28	17.37	20	-5.51	11.39	20	9.8%	5.79 [-3.31, 14.89]
Zhao, S.2017	6.5	9.23	40	3.03	9.12	40	10.3%	3.47 [-0.55, 7.49]
**Total (95% CI)**			**281**			**280**	**100.0%**	**8.53 [-2.68, 19.74]**

Heterogeneity: Tau² = 312.47; Chi² = 264.54, df = 9 (P < 0.00001); I² = 97%
Test for overall effect: Z = 1.49 (P = 0.14)

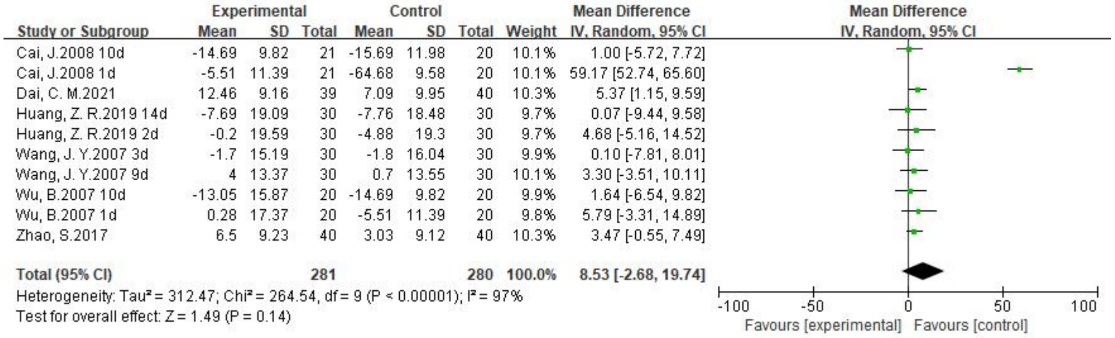

1.24 CD4(Psychotherapy)

Study or Subgroup	Experimental Mean	SD	Total	Control Mean	SD	Total	Weight	Mean Difference IV, Random, 95% CI
Cai, J.2008 10d	-8.01	8.35	21	-9.02	7.37	20	10.4%	1.01 [-3.81, 5.83]
Cai, J.2008 1d	-2.32	8.9	21	-41.11	5.54	20	10.4%	38.79 [34.28, 43.30]
Dai, C. M.2021	9.4	6.18	39	5.11	6.28	40	10.6%	4.29 [1.54, 7.04]
Huang, Z. R.2019 14d	-7.38	17.13	30	-6.57	14.8	30	9.7%	-0.81 [-8.91, 7.29]
Huang, Z. R.2019 2d	-1.05	17.88	30	-4.69	15.22	30	9.7%	3.64 [-4.76, 12.04]
Wang, J. Y.2007 3d	0.1	17.07	30	-7.8	18.53	30	9.5%	7.90 [-1.12, 16.92]
Wang, J. Y.2007 9d	4.7	18.17	30	-1.2	17.7	30	9.5%	5.90 [-3.18, 14.98]
Wu, B.2007 10d	-16.4	17.65	20	-8.01	8.35	20	9.6%	-8.39 [-16.95, 0.17]
Wu, B.2007 1d	3.83	15.75	20	-2.32	8.9	20	9.8%	6.15 [-1.78, 14.08]
Zhao, S.2017	8.76	7.23	40	3.91	6.29	40	10.6%	4.85 [1.88, 7.82]
**Total (95% CI)**			**281**			**280**	**100.0%**	**6.51 [-1.68, 14.69]**

Heterogeneity: Tau² = 161.76; Chi² = 218.04, df = 9 (P < 0.00001); I² = 96%
Test for overall effect: Z = 1.56 (P = 0.12)

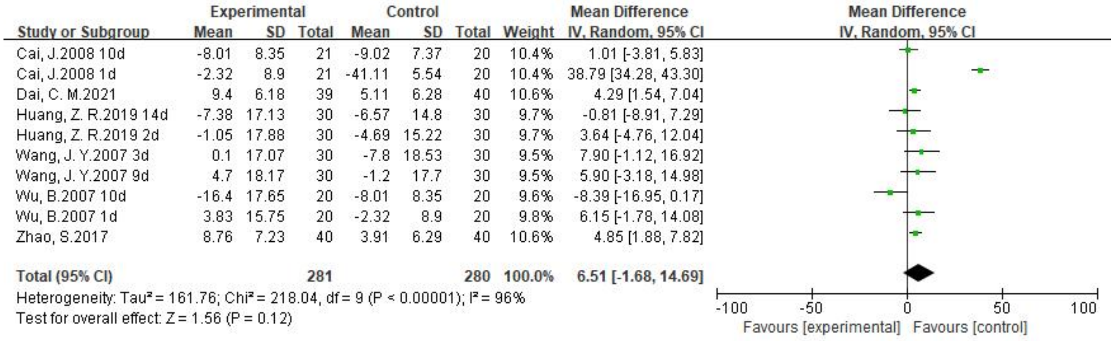

1.25 CD8(Psychotherapy)

Study or Subgroup	Experimental Mean	SD	Total	Control Mean	SD	Total	Weight	Mean Difference IV, Random, 95% CI
Huang, Z. R.2019 14d	-1.87	14.75	30	-1.88	14.02	30	13.2%	0.01 [-7.27, 7.29]
Huang, Z. R.2019 2d	-0.79	15.17	30	-1.02	14.12	30	13.0%	0.23 [-7.19, 7.65]
Wang, J. Y.2007 3d	3.2	13.61	30	3	14.05	30	13.6%	0.20 [-6.80, 7.20]
Wang, J. Y.2007 9d	-16.3	15.18	30	3.8	14.66	30	12.8%	-20.10 [-27.65, -12.55]
Wu, B.2007 10d	0.1	14.29	20	0.65	8.23	20	13.3%	-0.55 [-7.78, 6.68]
Wu, B.2007 1d	-3.21	13.03	20	2.65	8.58	20	13.8%	-5.86 [-12.70, 0.98]
Zhao, S.2017	-5.47	4.15	40	-2.63	4.38	40	20.3%	-2.84 [-4.71, -0.97]
**Total (95% CI)**			**200**			**200**	**100.0%**	**-3.98 [-8.30, 0.34]**

Heterogeneity: Tau² = 22.96; Chi² = 22.95, df = 6 (P = 0.0008); I² = 74%
Test for overall effect: Z = 1.81 (P = 0.07)

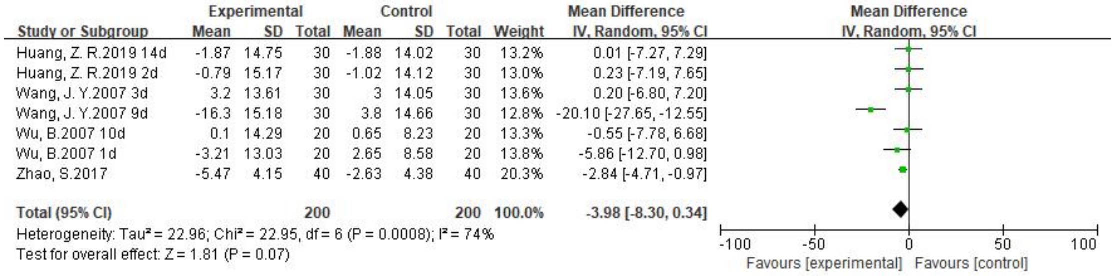

1.26CD4/CD8(Psychotherapy)

Study or Subgroup	Experimental Mean	SD	Total	Control Mean	SD	Total	Weight	Mean Difference IV, Random, 95% CI
Cai, J.2008 10d	-0.3	0.52	21	-0.26	0.54	20	10.3%	-0.04 [-0.36, 0.28]
Cai, J.2008 1d	-0.15	0.66	21	-0.24	0.53	20	9.0%	0.09 [-0.28, 0.46]
Dai, C. M.2021	0.79	0.25	40	0.47	0.23	40	20.3%	0.32 [0.21, 0.43]
Huang, Z. R.2019 14d	-0.23	0.45	30	-0.17	0.45	30	14.3%	-0.06 [-0.29, 0.17]
Huang, Z. R.2019 2d	0.02	0.44	30	-0.09	0.52	30	13.5%	0.11 [-0.13, 0.35]
Wang, J. Y.2007 3d	-0.3	1.7	30	-0.8	1.98	30	2.1%	0.50 [-0.43, 1.43]
Wang, J. Y.2007 9d	0.2	1.84	30	-0.1	1.84	30	2.1%	0.30 [-0.63, 1.23]
Wu, B.2007 10d	-0.86	1.21	20	-0.3	0.52	20	4.7%	-0.56 [-1.14, 0.02]
Wu, B.2007 1d	0.28	1.42	20	-0.15	0.66	20	3.5%	0.43 [-0.26, 1.12]
Zhao, S.2017	0.79	0.25	40	0.47	0.23	40	20.3%	0.32 [0.21, 0.43]
**Total (95% CI)**			**282**			**280**	**100.0%**	**0.15 [0.00, 0.29]**

Heterogeneity: Tau² = 0.02; Chi² = 23.67, df = 9 (P = 0.005); I² = 62%
Test for overall effect: Z = 2.03 (P = 0.04)

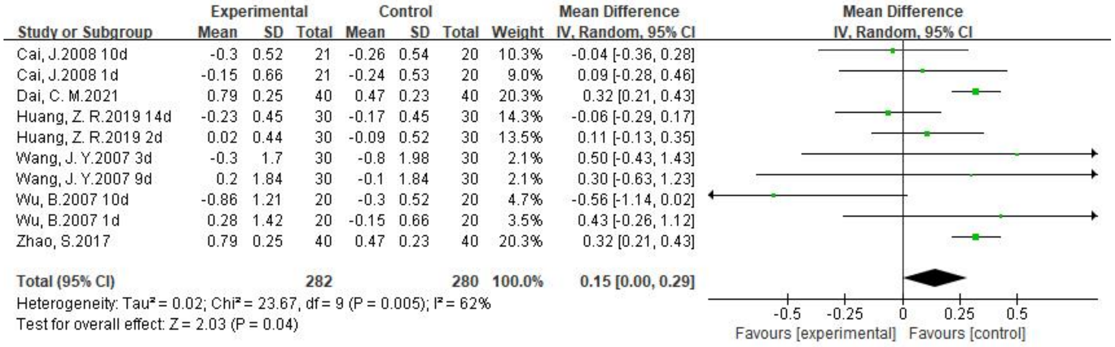

1.27 NK(Psychotherapy)

Study or Subgroup	Experimental Mean	SD	Total	Control Mean	SD	Total	Weight	Mean Difference IV, Fixed, 95% CI
Huang, Z. R.2019 14d	-1.25	3.89	30	-35.85	4.06	30	53.0%	34.60 [32.59, 36.61]
Huang, Z. R.2019 2d	-0.12	4.42	30	-35.16	4.02	30	47.0%	35.04 [32.90, 37.18]
**Total (95% CI)**			**60**			**60**	**100.0%**	**34.81 [33.34, 36.27]**

Heterogeneity: Chi² = 0.09, df = 1 (P = 0.77); I² = 0%
Test for overall effect: Z = 46.56 (P < 0.00001)

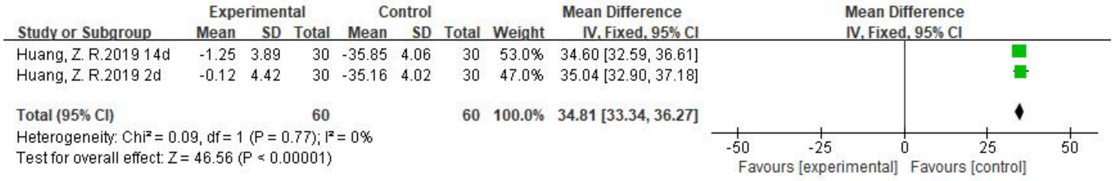

1.28 CD3(TP)

Study or Subgroup	Experimental Mean	SD	Total	Control Mean	SD	Total	Weight	Mean Difference IV, Fixed, 95% CI
Wang, D.2018 1m	11.4	19.68	28	1.6	16.4	28	49.1%	9.80 [0.31, 19.29]
Wang, D.2018 3m	13.1	18.4	28	2.3	17.13	28	50.9%	10.80 [1.49, 20.11]
**Total (95% CI)**			**56**			**56**	**100.0%**	**10.31 [3.66, 16.96]**

Heterogeneity: Chi² = 0.02, df = 1 (P = 0.88); I² = 0%
Test for overall effect: Z = 3.04 (P = 0.002)

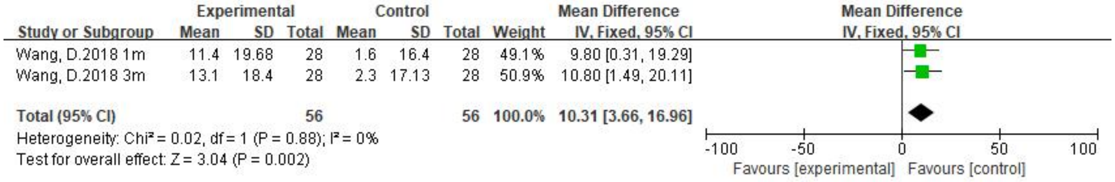

1.29 CD4(TP)

Study or Subgroup	Experimental Mean	Experimental SD	Experimental Total	Control Mean	Control SD	Control Total	Weight	Mean Difference IV, Random, 95% CI
Li，T2007 1w	8.31	12.86	25	-8.76	11.97	25	19.0%	17.07 [10.18, 23.96]
Li，T2007 3w	10.24	10.51	25	-7.05	10.2	25	20.3%	17.29 [11.55, 23.03]
Wang, D.2018 1m	12.1	14.09	28	0.7	10.12	28	19.5%	11.40 [4.97, 17.83]
Wang, D.2018 3m	10	12.43	28	2	12.9	28	19.3%	8.00 [1.36, 14.64]
Zhao, J.2022	-3.06	11.79	60	-4.79	12.13	60	21.8%	1.73 [-2.55, 6.01]
**Total (95% CI)**			**166**			**166**	**100.0%**	**10.91 [4.36, 17.46]**

Heterogeneity: Tau² = 46.37; Chi² = 24.90, df = 4 (P < 0.0001); I² = 84%
Test for overall effect: Z = 3.26 (P = 0.001)

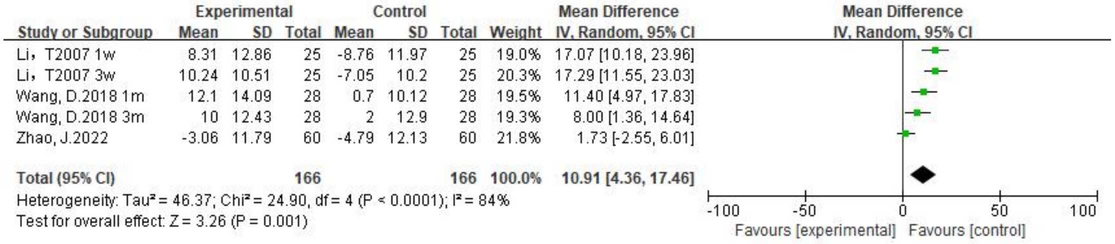

1.30 CD8(TP)

Study or Subgroup	Experimental Mean	Experimental SD	Experimental Total	Control Mean	Control SD	Control Total	Weight	Mean Difference IV, Random, 95% CI
Li，T2007 1w	0.92	12.15	25	-1.77	12.15	25	17.2%	2.69 [-4.05, 9.43]
Li，T2007 3w	3.33	11.65	25	-7.05	9.95	25	19.0%	10.38 [4.37, 16.39]
Wang, D.2018 1m	-0.9	8.49	28	0.3	11.54	28	20.8%	-1.20 [-6.51, 4.11]
Wang, D.2018 3m	-2.3	9.86	28	0.1	12.75	28	19.1%	-2.40 [-8.37, 3.57]
Zhao, J.2022	-3.16	12.01	60	-6.13	11.22	60	24.0%	2.97 [-1.19, 7.13]
**Total (95% CI)**			**166**			**166**	**100.0%**	**2.44 [-1.69, 6.56]**

Heterogeneity: Tau² = 13.99; Chi² = 11.11, df = 4 (P = 0.03); I² = 64%
Test for overall effect: Z = 1.16 (P = 0.25)

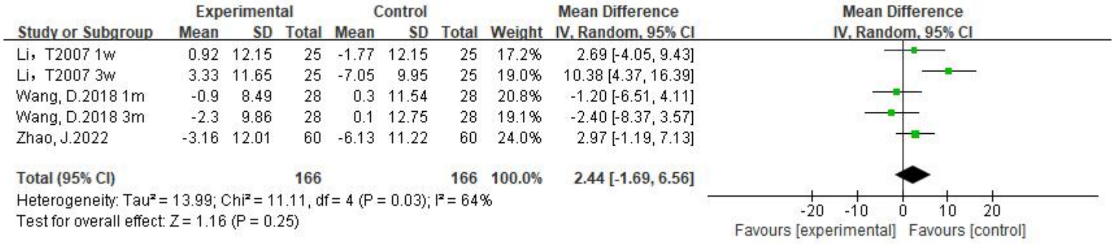

1.31 CD4/CD8(TP)

Study or Subgroup	Experimental Mean	Experimental SD	Experimental Total	Control Mean	Control SD	Control Total	Weight	Mean Difference IV, Random, 95% CI
Wang, D.2018 1m	0.5	0.5	28	0	0.78	28	24.4%	0.50 [0.16, 0.84]
Wang, D.2018 3m	0.5	0.42	28	0	0.71	28	27.7%	0.50 [0.19, 0.81]
Zhao, J.2022	-0.1	0.43	60	-0.31	0.34	60	47.9%	0.21 [0.07, 0.35]
**Total (95% CI)**			**116**			**116**	**100.0%**	**0.36 [0.14, 0.58]**

Heterogeneity: Tau² = 0.02; Chi² = 4.53, df = 2 (P = 0.10); I² = 56%
Test for overall effect: Z = 3.20 (P = 0.001)

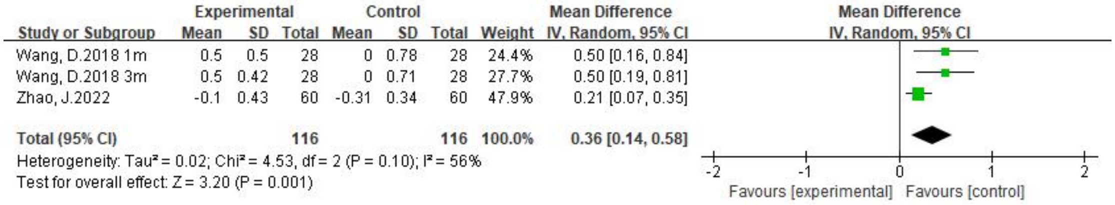

1.32 NK(TP)

Study or Subgroup	Experimental Mean	Experimental SD	Experimental Total	Control Mean	Control SD	Control Total	Weight	Mean Difference IV, Random, 95% CI
Li，T2007 1w	13.22	11.72	25	-1.81	12.88	25	29.4%	15.03 [8.20, 21.86]
Li，T2007 3w	7.25	10.2	25	-1.39	10.98	25	31.6%	8.64 [2.77, 14.51]
Zhao, J.2022	-2.77	6.08	60	-5.86	5.59	60	39.0%	3.09 [1.00, 5.18]
**Total (95% CI)**			**110**			**110**	**100.0%**	**8.35 [1.28, 15.42]**

Heterogeneity: Tau² = 32.24; Chi² = 12.79, df = 2 (P = 0.002); I² = 84%
Test for overall effect: Z = 2.31 (P = 0.02)

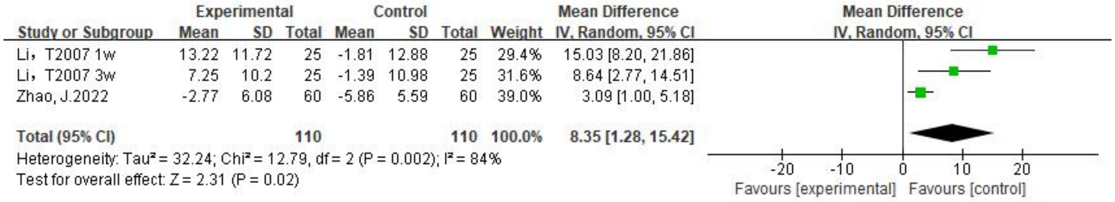

1.33 CD3(TD)

Study or Subgroup	Experimental Mean	Experimental SD	Experimental Total	Control Mean	Control SD	Control Total	Weight	Mean Difference IV, Random, 95% CI
Chen, C.2023	13.46	12.25	75	7.6	10.77	75	48.6%	5.86 [2.17, 9.55]
Li, X.2019	-2.11	3.36	57	-18.47	3.66	57	51.4%	16.36 [15.07, 17.65]
**Total (95% CI)**			**132**			**132**	**100.0%**	**11.26 [0.97, 21.54]**

Heterogeneity: Tau² = 53.13; Chi² = 27.70, df = 1 (P < 0.00001); I² = 96%
Test for overall effect: Z = 2.15 (P = 0.03)

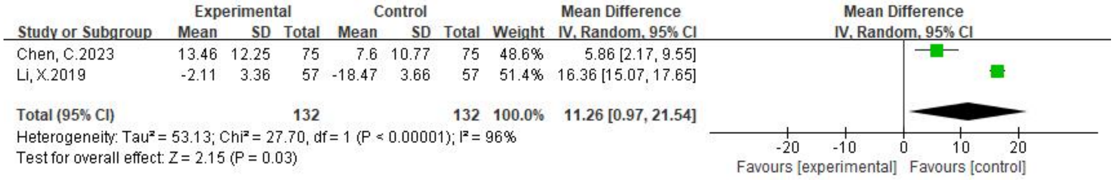

1.34 CD4(TD)

Study or Subgroup	Experimental Mean	Experimental SD	Experimental Total	Control Mean	Control SD	Control Total	Weight	Mean Difference IV, Fixed, 95% CI
Li, X.2019	-3.7	9.42	57	-14.33	8.28	57	34.1%	10.63 [7.37, 13.89]
Lin, S.2022 1w	-11.26	8.64	45	-22.1	7.35	45	32.9%	10.84 [7.53, 14.15]
Lin, S.2022 4w	-0.37	8.48	45	-10.57	7.52	45	33.0%	10.20 [6.89, 13.51]
**Total (95% CI)**			**147**			**147**	**100.0%**	**10.56 [8.66, 12.46]**

Heterogeneity: Chi² = 0.07, df = 2 (P = 0.96); I² = 0%
Test for overall effect: Z = 10.88 (P < 0.00001)

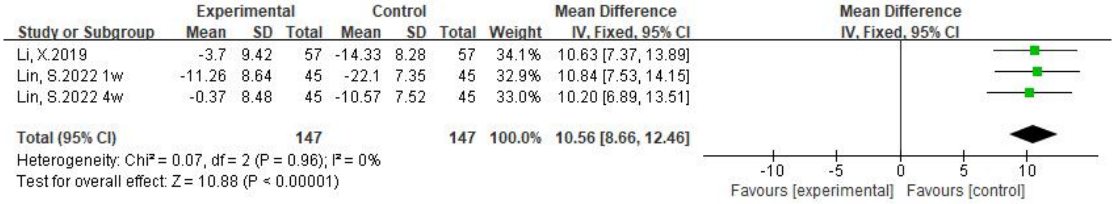

1.35 CD8(TD)

Study or Subgroup	Experimental Mean	Experimental SD	Experimental Total	Control Mean	Control SD	Control Total	Weight	Mean Difference IV, Random, 95% CI
Li, X.2019	2.05	8.06	57	10.63	7.48	57	25.8%	-8.58 [-11.43, -5.73]
Lin, S.2022 1w	9.21	4.37	45	14.37	4.72	45	38.0%	-5.16 [-7.04, -3.28]
Lin, S.2022 4w	4.16	4.69	45	9.24	5	45	36.2%	-5.08 [-7.08, -3.08]
**Total (95% CI)**			**147**			**147**	**100.0%**	**-6.01 [-7.94, -4.09]**

Heterogeneity: Tau² = 1.62; Chi² = 4.58, df = 2 (P = 0.10); I² = 56%
Test for overall effect: Z = 6.12 (P < 0.00001)

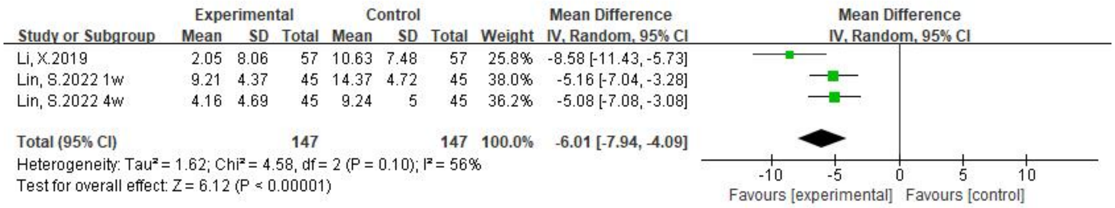

1.36 CD4/CD8(TD)

Study or Subgroup	Experimental Mean	Experimental SD	Experimental Total	Control Mean	Control SD	Control Total	Weight	Mean Difference IV, Fixed, 95% CI
Chen, C.2023	0.63	0.3	75	0.28	0.19	75	51.8%	0.35 [0.27, 0.43]
Li, X.2019	-0.8	2.28	57	-1.63	1.81	57	0.6%	0.83 [0.07, 1.59]
Lin, S.2022 1w	-0.64	0.24	45	-0.98	0.24	45	34.0%	0.34 [0.24, 0.44]
Lin, S.2022 4w	-0.16	0.39	45	-0.63	0.37	45	13.6%	0.47 [0.31, 0.63]
**Total (95% CI)**			**222**			**222**	**100.0%**	**0.37 [0.31, 0.42]**

Heterogeneity: Chi² = 3.55, df = 3 (P = 0.31); I² = 15%
Test for overall effect: Z = 12.39 (P < 0.00001)

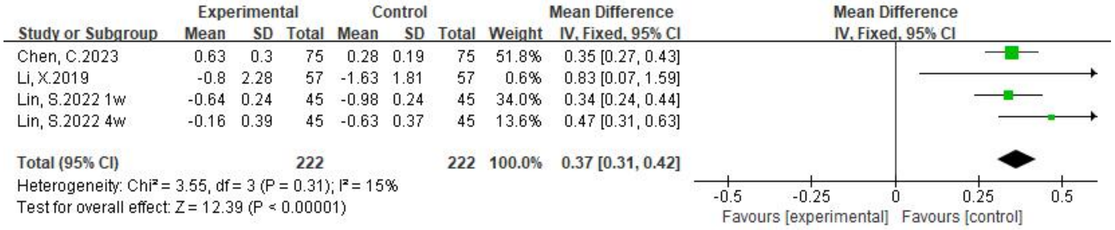

1.37 NK(TD)

Study or Subgroup	Experimental Mean	Experimental SD	Experimental Total	Control Mean	Control SD	Control Total	Weight	Mean Difference IV, Fixed, 95% CI
Lin, S.2022 1w	-3.25	3.8	45	-6	3.72	45	51.7%	2.75 [1.20, 4.30]
Lin, S.2022 4w	-0.13	4.06	45	-3.22	3.72	45	48.3%	3.09 [1.48, 4.70]
**Total (95% CI)**			**90**			**90**	**100.0%**	**2.91 [1.80, 4.03]**

Heterogeneity: Chi² = 0.09, df = 1 (P = 0.77); I² = 0%
Test for overall effect: Z = 5.11 (P < 0.00001)

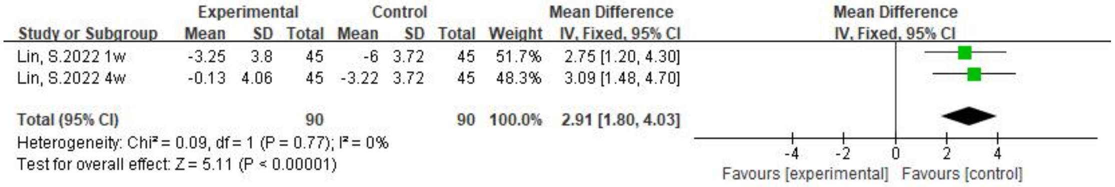

1.38 CD3(CD)

Study or Subgroup	Experimental Mean	Experimental SD	Experimental Total	Control Mean	Control SD	Control Total	Weight	Mean Difference IV, Fixed, 95% CI
Nie, Z.2021	1.73	8.96	126	-1.25	9.34	126	50.8%	2.98 [0.72, 5.24]
Yu, H.2023	5.26	4.82	33	2.7	4.69	33	49.2%	2.56 [0.27, 4.85]
**Total (95% CI)**			**159**			**159**	**100.0%**	**2.77 [1.16, 4.38]**

Heterogeneity: Chi² = 0.07, df = 1 (P = 0.80); I² = 0%
Test for overall effect: Z = 3.38 (P = 0.0007)

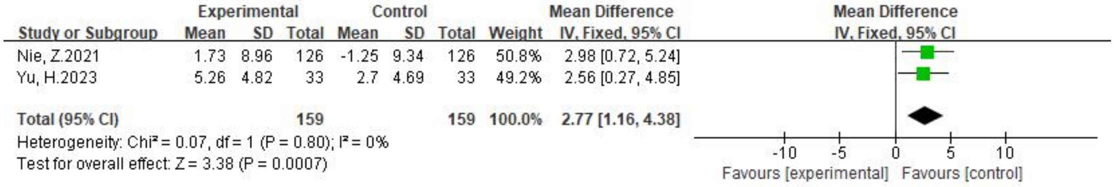

1.39 CD4(CD)

Study or Subgroup	Experimental Mean	Experimental SD	Experimental Total	Control Mean	Control SD	Control Total	Weight	Mean Difference IV, Fixed, 95% CI
Gu, R. M.2012	5.4	9.63	20	3.7	9.62	22	7.3%	1.70 [-4.13, 7.53]
Nie, Z.2021	3.34	10	126	0.87	9.74	126	41.7%	2.47 [0.03, 4.91]
Yu, H.2023	9.44	4.62	33	7.3	4.51	33	51.0%	2.14 [-0.06, 4.34]
**Total (95% CI)**			**179**			**181**	**100.0%**	**2.25 [0.67, 3.82]**

Heterogeneity: Chi² = 0.08, df = 2 (P = 0.96); I² = 0%
Test for overall effect: Z = 2.80 (P = 0.005)

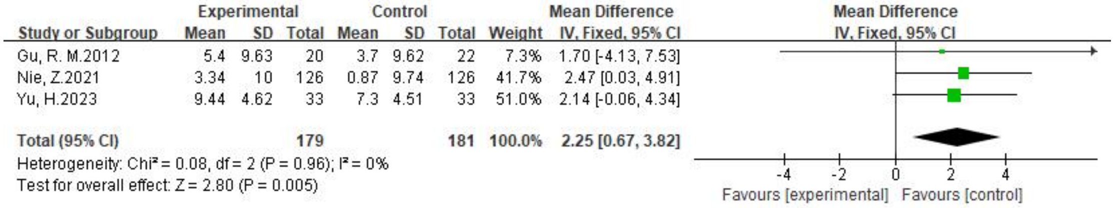

1.40 CD8(CD)

Study or Subgroup	Experimental Mean	Experimental SD	Experimental Total	Control Mean	Control SD	Control Total	Weight	Mean Difference IV, Fixed, 95% CI
Gu, R. M.2012	-2.1	7.51	20	-1.4	7.78	22	17.2%	-0.70 [-5.33, 3.93]
Nie, Z.2021	-3.29	8.51	126	-1.05	8.59	126	82.8%	-2.24 [-4.35, -0.13]
**Total (95% CI)**			**146**			**148**	**100.0%**	**-1.97 [-3.90, -0.05]**

Heterogeneity: Chi² = 0.35, df = 1 (P = 0.55); I² = 0%
Test for overall effect: Z = 2.01 (P = 0.04)

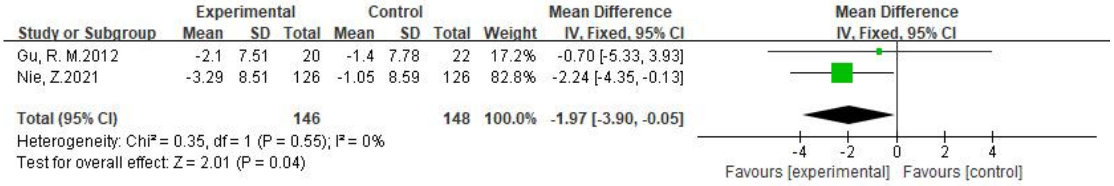

1.41 CD4/CD8(CD)

Study or Subgroup	Experimental Mean	Experimental SD	Experimental Total	Control Mean	Control SD	Control Total	Weight	Mean Difference IV, Random, 95% CI
Gu, R. M.2012	0.53	0.63	20	0.4	0.72	22	100.0%	0.13 [-0.28, 0.54]
**Total (95% CI)**			**20**			**22**	**100.0%**	**0.13 [-0.28, 0.54]**

Heterogeneity: Not applicable
Test for overall effect: Z = 0.62 (P = 0.53)

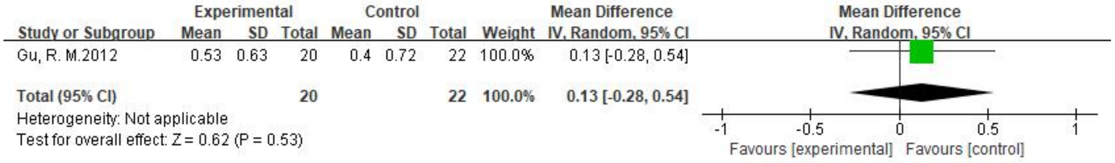

1.42 NK(CD)

Study or Subgroup	Experimental Mean	Experimental SD	Experimental Total	Control Mean	Control SD	Control Total	Weight	Mean Difference IV, Fixed, 95% CI
Gu, R. M.2012	-1.1	6.15	20	-0.1	6.26	22	100.0%	-1.00 [-4.76, 2.76]
**Total (95% CI)**			**20**			**22**	**100.0%**	**-1.00 [-4.76, 2.76]**

Heterogeneity: Not applicable
Test for overall effect: Z = 0.52 (P = 0.60)

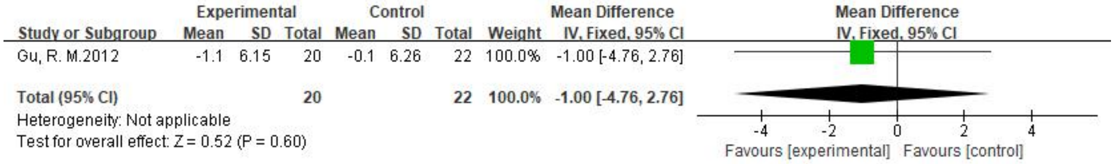

1.43CD3(NIV)

Study or Subgroup	Experimental Mean	Experimental SD	Experimental Total	Control Mean	Control SD	Control Total	Weight	Mean Difference IV, Random, 95% CI
Wang, L.2019 1d	-9.5	5.02	20	-11.8	4.19	20	6.1%	2.30 [-0.57, 5.17]
Wang, L.2019 3d	-7.5	4.65	20	-9.7	4.74	20	6.0%	2.20 [-0.71, 5.11]
Wang, L.2019 7d	-4.4	8.03	20	-2.5	7.56	20	4.9%	-1.90 [-6.73, 2.93]
Xin, L.2019 1d	-11.36	8.49	100	-10.14	8.95	100	6.3%	-1.22 [-3.64, 1.20]
Xin, L.2019 1h	-14.57	11.96	100	-17.63	9.89	100	6.0%	3.06 [0.02, 6.10]
Xin, L.2019 3d	-3.87	12.34	100	-12.98	9.35	100	6.0%	9.11 [6.08, 12.14]
Zhu, J.2017 end	-1.45	12.4	72	-5.62	15.67	72	5.0%	4.17 [-0.45, 8.79]
Zhu, J.2017 1d	-15.93	14.29	72	-21.47	21.68	72	4.2%	5.54 [-0.46, 11.54]
Zhu, J.2017 2h	-3.49	12.35	72	-8.65	16.89	72	4.9%	5.16 [0.33, 9.99]
Zhu, J.2017 3d	-1.73	12.96	72	-24.88	19.25	72	4.6%	23.15 [17.79, 28.51]
Zhu, R.2020 4h	-1.09	6.07	30	-5.05	6.36	30	5.9%	3.96 [0.81, 7.11]
Zhu, R.2020 1d	-5.13	6.26	30	-10.16	7.13	30	5.8%	5.03 [1.63, 8.43]
Zhu, R.2020 2d	-13.82	6.18	30	-18.02	6.32	30	5.9%	4.20 [1.04, 7.36]
Zhu, R.2020 open2h	-6.45	6.64	30	-10.09	7.11	30	5.7%	3.64 [0.16, 7.12]
Zhu, R.2020EA1d	-0.62	8.57	30	-5.05	6.36	30	5.5%	4.43 [0.61, 8.25]
Zhu, R.2020EA2d	-2.82	6.69	30	-10.16	7.13	30	5.7%	7.34 [3.84, 10.84]
Zhu, R.2020EA4h	-10.75	6.5	30	-18.02	6.32	30	5.8%	7.27 [4.03, 10.51]
Zhu, R.2020EAopen2h	-2.65	6.51	30	-10.09	7.11	30	5.7%	7.44 [3.99, 10.89]
**Total (95% CI)**			**888**			**888**	**100.0%**	**5.08 [3.10, 7.07]**

Heterogeneity: Tau² = 14.80; Chi² = 97.89, df = 17 (P < 0.00001); I² = 83%
Test for overall effect: Z = 5.02 (P < 0.00001)

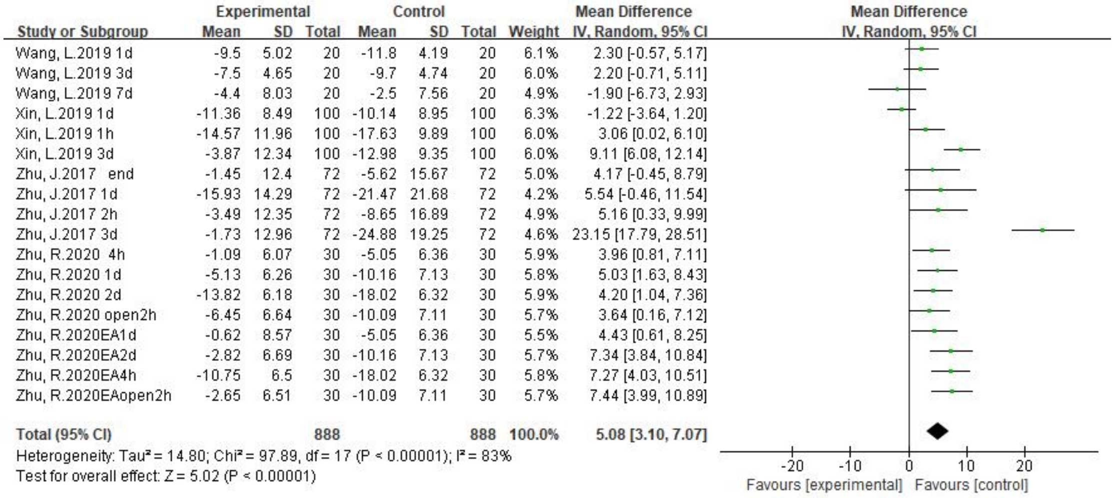

1.44 CD4(NIV)

Study or Subgroup	Experimental Mean	Experimental SD	Experimental Total	Control Mean	Control SD	Control Total	Weight	Mean Difference IV, Random, 95% CI
Wang, L.2019 1d	-11.1	4.95	20	-10.9	5.48	20	5.6%	-0.20 [-3.44, 3.04]
Wang, L.2019 3d	-10	4.08	20	-9.2	4.59	20	6.1%	-0.80 [-3.49, 1.89]
Wang, L.2019 7d	-1.5	4.03	20	-0.7	5.13	20	6.0%	-0.80 [-3.66, 2.06]
Wang, M. Q.2018	6.24	5	20	3.35	5.16	20	5.7%	2.89 [-0.26, 6.04]
Xin, L.2019 1d	-9.95	5.84	100	-9.68	6.34	100	6.8%	-0.27 [-1.96, 1.42]
Xin, L.2019 1h	-6.91	7.54	100	-6.94	7.13	100	6.6%	0.03 [-2.00, 2.06]
Xin, L.2019 3d	1.09	8.37	100	-7.51	9.09	100	6.3%	8.60 [6.18, 11.02]
Zhu, J.2017 end	-4.87	11.53	72	-3.27	10.45	72	5.3%	-1.60 [-5.19, 1.99]
Zhu, J.2017 1d	-9.26	13.45	72	-8.76	12.02	72	4.9%	-0.50 [-4.67, 3.67]
Zhu, J.2017 2h	-18.49	12.57	72	-18.92	11.32	72	5.1%	0.43 [-3.48, 4.34]
Zhu, J.2017 3d	-10.12	12.47	72	-13.13	11.49	72	5.1%	3.01 [-0.91, 6.93]
Zhu, R.2020 4h	-2.86	9.25	30	-4.45	9	30	4.5%	1.59 [-3.03, 6.21]
Zhu, R.2020 1d	-6.82	9.57	30	-9.76	8.47	30	4.6%	2.94 [-1.63, 7.51]
Zhu, R.2020 2d	-9.73	9.99	30	-14.13	7.98	30	4.6%	4.40 [-0.18, 8.98]
Zhu, R.2020 open2h	-6.42	9.76	30	-7.92	8.76	30	4.5%	1.50 [-3.19, 6.19]
Zhu, R.2020EA1d	-1.55	9.02	30	-4.45	9	30	4.6%	2.90 [-1.66, 7.46]
Zhu, R.2020EA2d	-2.71	9.56	30	-9.76	8.47	30	4.6%	7.05 [2.48, 11.62]
Zhu, R.2020EA4h	-4.88	9.66	30	-14.13	7.98	30	4.6%	9.25 [4.77, 13.73]
Zhu, R.2020EAopen2h	-1.41	9.41	30	-7.92	8.76	30	4.5%	6.51 [1.91, 11.11]
**Total (95% CI)**			**908**			**908**	**100.0%**	**2.30 [0.73, 3.87]**

Heterogeneity: Tau² = 8.63; Chi² = 74.50, df = 18 (P < 0.00001); I² = 76%
Test for overall effect: Z = 2.87 (P = 0.004)

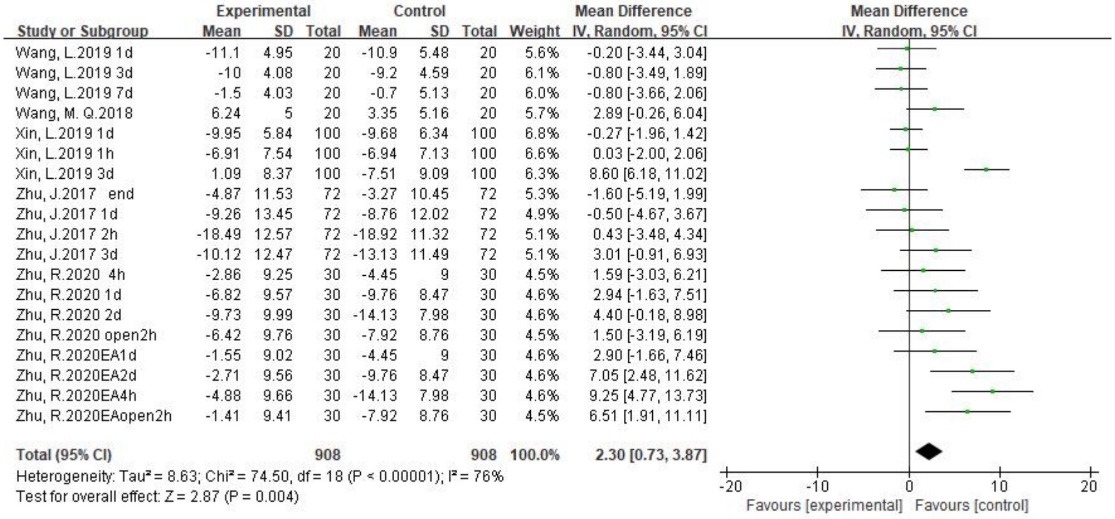

1.45 CD8(NIV)

Study or Subgroup	Experimental Mean	Experimental SD	Experimental Total	Control Mean	Control SD	Control Total	Weight	Mean Difference IV, Random, 95% CI
Wang, L.2019 1d	-0.3	1.63	20	-0.4	2.55	20	6.2%	0.10 [-1.23, 1.43]
Wang, L.2019 3d	1.4	2.33	20	-0.1	2.62	20	6.1%	1.50 [-0.04, 3.04]
Wang, L.2019 7d	-0.2	1.92	20	-0.2	2.22	20	6.3%	0.00 [-1.29, 1.29]
Wang, M. Q.2018	2.56	5.84	20	-5.82	6.22	20	4.7%	8.38 [4.64, 12.12]
Xin, L.2019 1d	-5.57	3.12	100	-3.09	3.32	100	6.4%	-2.48 [-3.37, -1.59]
Xin, L.2019 1h	-3.51	4.32	100	-2.79	4.02	100	6.3%	-0.72 [-1.88, 0.44]
Xin, L.2019 3d	-0.1	4.94	100	-1.8	5.81	100	6.2%	1.70 [0.21, 3.19]
Zhu, J.2017 end	-2.55	10.55	72	-0.86	10.01	72	4.9%	-1.69 [-5.05, 1.67]
Zhu, J.2017 1d	-2.71	11.68	72	-1.82	11.73	72	4.6%	-0.89 [-4.71, 2.93]
Zhu, J.2017 2h	-3.31	11.53	72	0.58	10.48	72	4.8%	-3.89 [-7.49, -0.29]
Zhu, J.2017 3d	0.12	11.1	72	-0.73	11.42	72	4.7%	0.85 [-2.83, 4.53]
Zhu, R.2020 4h	-0.71	7.53	30	-1.42	6.81	30	4.7%	0.71 [-2.92, 4.34]
Zhu, R.2020 1d	-2.22	7	30	-2.21	6.25	30	4.9%	-0.01 [-3.37, 3.35]
Zhu, R.2020 2d	-1.78	6.59	30	-1.43	6.18	30	5.0%	-0.35 [-3.58, 2.88]
Zhu, R.2020 open2h	-0.49	7.43	30	-1.68	6.03	30	4.9%	1.19 [-2.23, 4.61]
Zhu, R.2020EA1d	-1.08	7.48	30	-1.42	6.81	30	4.7%	0.34 [-3.28, 3.96]
Zhu, R.2020EA2d	-21.22	7.53	30	-2.21	6.25	30	4.8%	-19.01 [-22.51, -15.51]
Zhu, R.2020EA4h	-2.39	7.42	30	-1.43	6.18	30	4.9%	-0.96 [-4.42, 2.50]
Zhu, R.2020EAopen2h	-0.53	7.48	30	-1.68	6.03	30	4.9%	1.15 [-2.29, 4.59]
**Total (95% CI)**			**908**			**908**	**100.0%**	**-0.70 [-2.20, 0.80]**

Heterogeneity: Tau² = 8.93; Chi² = 170.69, df = 18 (P < 0.00001); I² = 89%

Test for overall effect: Z = 0.91 (P = 0.36)

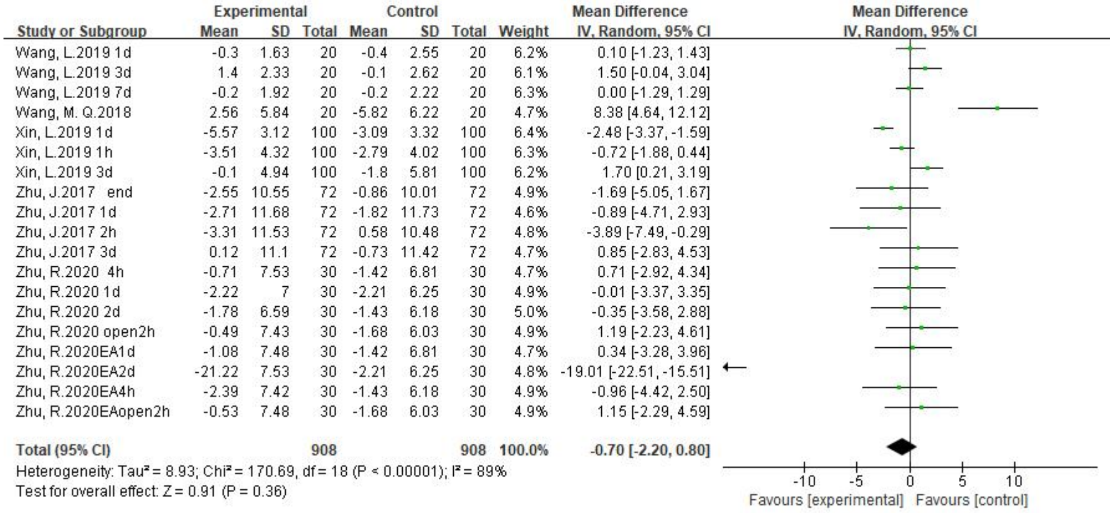

1.46 CD4/CD8(NIV)

Study or Subgroup	Experimental Mean	Experimental SD	Experimental Total	Control Mean	Control SD	Control Total	Weight	Mean Difference IV, Fixed, 95% CI
Wang, L.2019 1d	-0.4	0.54	20	-0.5	0.54	20	1.9%	0.10 [-0.23, 0.43]
Wang, L.2019 3d	-0.4	0.58	20	-0.5	0.51	20	1.8%	0.10 [-0.24, 0.44]
Wang, L.2019 7d	-0.1	0.51	20	0	0.54	20	2.0%	-0.10 [-0.43, 0.23]
Wang, M. Q.2018	-0.05	0.56	20	-0.18	0.52	20	1.9%	0.13 [-0.20, 0.46]
Xin, L.2019 1d	-0.4	0.75	100	-0.34	0.89	100	4.0%	-0.06 [-0.29, 0.17]
Xin, L.2019 1h	-0.21	0.31	100	-0.3	0.39	100	21.8%	0.09 [-0.01, 0.19]
Xin, L.2019 3d	-0.01	0.51	100	-0.28	0.44	100	11.9%	0.27 [0.14, 0.40]
Zhu, J.2017 end	-0.08	0.45	72	-0.07	0.49	72	8.8%	-0.01 [-0.16, 0.14]
Zhu, J.2017 1d	-0.22	0.48	72	-0.21	0.6	72	6.6%	-0.01 [-0.19, 0.17]
Zhu, J.2017 2h	-0.53	0.46	72	-0.68	0.47	72	9.0%	0.15 [-0.00, 0.30]
Zhu, J.2017 3d	-0.39	0.43	72	-0.43	0.54	72	8.2%	0.04 [-0.12, 0.20]
Zhu, R.2020 4h	-0.08	0.65	30	-0.08	0.46	30	2.6%	0.00 [-0.28, 0.28]
Zhu, R.2020 1d	-0.16	0.59	30	-0.26	0.45	30	3.0%	0.10 [-0.17, 0.37]
Zhu, R.2020 2d	-0.3	0.6	30	-0.48	0.41	30	3.1%	0.18 [-0.08, 0.44]
Zhu, R.2020 open2h	-0.22	0.58	30	-0.21	0.5	30	2.8%	-0.01 [-0.28, 0.26]
Zhu, R.2020EA1d	-0.01	0.59	30	-0.08	0.46	30	2.9%	0.07 [-0.20, 0.34]
Zhu, R.2020EA2d	-0.03	0.65	30	-0.26	0.45	30	2.6%	0.23 [-0.05, 0.51]
Zhu, R.2020EA4h	-0.05	0.67	30	-0.48	0.41	30	2.6%	0.43 [0.15, 0.71]
Zhu, R.2020EAopen2h	-0.03	0.62	30	-0.21	0.5	30	2.6%	0.18 [-0.11, 0.47]
**Total (95% CI)**			**908**			**908**	**100.0%**	**0.10 [0.06, 0.15]**

Heterogeneity: Chi² = 22.10, df = 18 (P = 0.23); I² = 19%

Test for overall effect: Z = 4.34 (P < 0.0001)

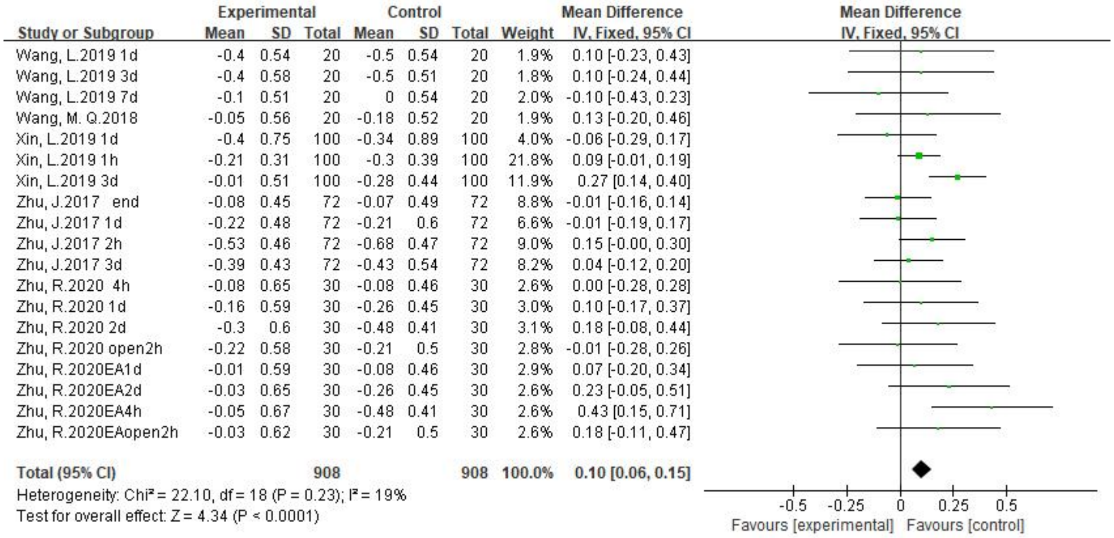

1.47 CD3(PPF)

Study or Subgroup	Experimental Mean	Experimental SD	Experimental Total	Control Mean	Control SD	Control Total	Weight	Mean Difference IV, Fixed, 95% CI
Liu, S.2016 1d	-0.4	0.66	29	-0.82	0.74	29	27.2%	0.42 [0.06, 0.78]
Liu, S.2016 2d	-0.45	0.91	29	-0.89	0.74	29	19.4%	0.44 [0.01, 0.87]
Liu, S.2016 3d	-0.15	0.68	29	-0.48	0.74	29	26.5%	0.33 [-0.04, 0.70]
Liu, S.2016 after operation	-0.07	0.68	29	-0.28	0.74	29	26.5%	0.21 [-0.16, 0.58]
Zhang, T.2014 after operation	-9.7	11.74	19	-10.1	13.59	18	0.1%	0.40 [-7.80, 8.60]
Zhang, T.2014 1d	-10.6	12	19	-15.2	12.4	18	0.1%	4.60 [-3.27, 12.47]
Zhang, T.2014 2d	-10.9	12.39	19	-15.8	12.23	18	0.1%	4.90 [-3.03, 12.83]
Zhang, T.2014 3d	-11	11.38	19	-13.8	13.66	18	0.1%	2.80 [-5.32, 10.92]
Zhang, T.2014 3h	-9.8	11.26	19	-16.6	12.76	18	0.1%	6.80 [-0.97, 14.57]
Zhang, T.2014 5h	-1.9	11.44	19	-13.4	12.46	18	0.1%	11.50 [3.78, 19.22]
Zhang, T.2014 anesthesia1h	1.2	13.15	19	-1.6	14	18	0.0%	2.80 [-5.96, 11.56]
**Total (95% CI)**			**249**			**242**	**100.0%**	**0.36 [0.17, 0.55]**

Heterogeneity: Chi² = 14.57, df = 10 (P = 0.15); I² = 31%

Test for overall effect: Z = 3.77 (P = 0.0002)

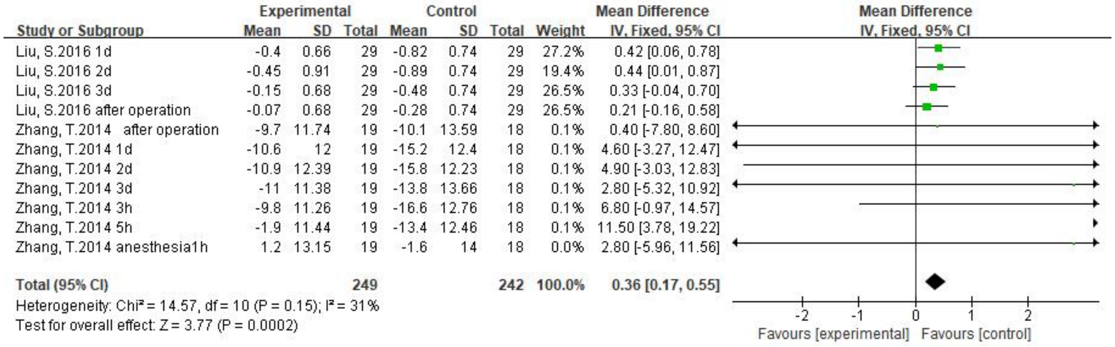

1.48 CD4(PPF)

Study or Subgroup	Experimental Mean	Experimental SD	Experimental Total	Control Mean	Control SD	Control Total	Weight	Mean Difference IV, Random, 95% CI
Liu, S.2016 1d	-0.23	0.42	29	-0.4	0.45	29	24.0%	0.17 [-0.05, 0.39]
Liu, S.2016 2d	-0.24	0.43	29	-0.44	0.42	29	24.1%	0.20 [-0.02, 0.42]
Liu, S.2016 3d	-0.26	0.42	29	-0.38	0.44	29	24.0%	0.12 [-0.10, 0.34]
Liu, S.2016 after operation	-0.09	0.44	29	-0.23	0.45	29	23.9%	0.14 [-0.09, 0.37]
Woo, J. H.2015 1d	1	8.18	20	14	7.84	20	0.8%	-13.00 [-17.97, -8.03]
Woo, J. H.2015 1h	-2	8.18	20	6	7.84	20	0.8%	-8.00 [-12.97, -3.03]
Zhang, T.2014 after operation	-6.7	12.36	19	-11.6	12.17	18	0.3%	4.90 [-3.01, 12.81]
Zhang, T.2014 1d	-5.9	11.68	19	-15.6	12.8	18	0.3%	9.70 [1.79, 17.61]
Zhang, T.2014 2d	-6.3	13.22	19	-15.1	12.17	18	0.3%	8.80 [0.62, 16.98]
Zhang, T.2014 3d	-5.3	12.55	19	-10.4	11.45	18	0.3%	5.10 [-2.63, 12.83]
Zhang, T.2014 3h	-5.9	12.29	19	-10.7	11.26	18	0.4%	4.80 [-2.79, 12.39]
Zhang, T.2014 5h	2.3	13.51	19	-10.6	12.03	18	0.3%	12.90 [4.67, 21.13]
Zhang, T.2014 anesthesia1h	1.1	12.68	19	-1.9	12.59	18	0.3%	3.00 [-5.14, 11.14]
**Total (95% CI)**			**289**			**282**	**100.0%**	**0.14 [-0.32, 0.59]**

Heterogeneity: Tau² = 0.21; Chi² = 61.56, df = 12 (P < 0.00001); I² = 81%
Test for overall effect: Z = 0.59 (P = 0.55)

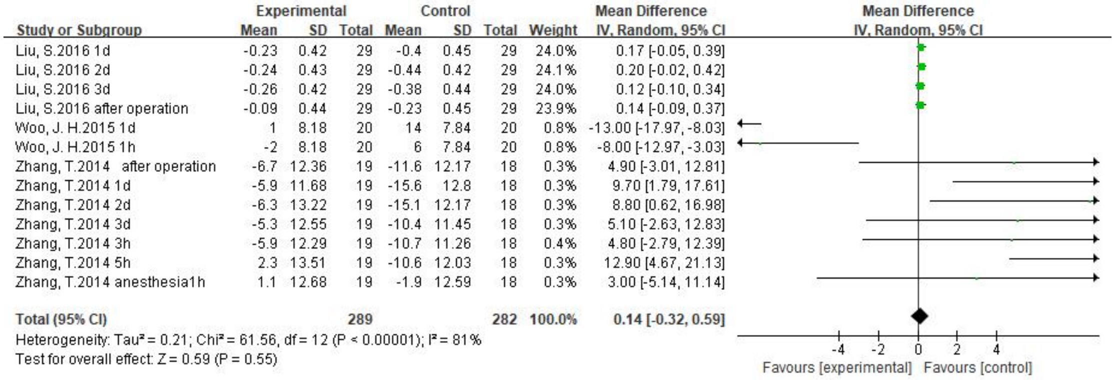

1.49 CD8(PPF)

Study or Subgroup	Experimental Mean	Experimental SD	Experimental Total	Control Mean	Control SD	Control Total	Weight	Mean Difference IV, Fixed, 95% CI
Liu, S.2016 1d	0.15	0.42	29	0.08	0.39	29	25.5%	0.07 [-0.14, 0.28]
Liu, S.2016 2d	-0.06	0.4	29	0.04	0.43	29	24.3%	-0.10 [-0.31, 0.11]
Liu, S.2016 3d	0.1	0.4	29	0.04	0.4	29	26.2%	0.06 [-0.15, 0.27]
Liu, S.2016 after operation	0.06	0.42	29	0.15	0.42	29	23.8%	-0.09 [-0.31, 0.13]
Woo, J. H.2015 1d	-7	7.76	20	3	8.64	20	0.0%	-10.00 [-15.09, -4.91]
Woo, J. H.2015 1h	-7	7.76	20	-7	8.64	20	0.0%	0.00 [-5.09, 5.09]
Zhang, T.2014 after operation	-2.9	11.05	19	1.1	14.21	18	0.0%	-4.00 [-12.23, 4.23]
Zhang, T.2014 1d	0.1	12.17	19	0.2	12.88	18	0.0%	-0.10 [-8.18, 7.98]
Zhang, T.2014 2d	1.1	11.39	19	2	13.8	18	0.0%	-0.90 [-9.08, 7.28]
Zhang, T.2014 3d	2	12.39	19	1.7	12.7	18	0.0%	0.30 [-7.79, 8.39]
Zhang, T.2014 3h	-0.3	11.74	19	2.1	12.35	18	0.0%	-2.40 [-10.17, 5.37]
Zhang, T.2014 5h	-0.4	12.17	19	-2	13.8	18	0.0%	1.60 [-6.80, 10.00]
Zhang, T.2014 anesthesia1h	-0.8	12.84	19	-0.8	13.66	18	0.0%	0.00 [-8.55, 8.55]
**Total (95% CI)**			**289**			**282**	**100.0%**	**-0.02 [-0.12, 0.09]**

Heterogeneity: Chi² = 18.46, df = 12 (P = 0.10); I² = 35%
Test for overall effect: Z = 0.32 (P = 0.75)

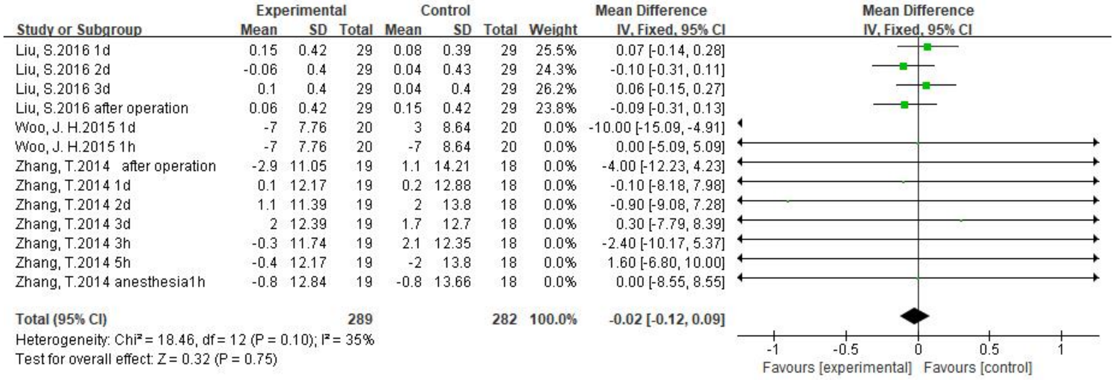

1.50 CD4/CD8(PPF)

Study or Subgroup	Experimental Mean	Experimental SD	Experimental Total	Control Mean	Control SD	Control Total	Weight	Mean Difference IV, Fixed, 95% CI
Liu, S.2016 1d	-0.39	0.98	29	-0.54	0.91	29	10.1%	0.15 [-0.34, 0.64]
Liu, S.2016 2d	-0.58	0.87	29	-0.79	0.7	29	14.5%	0.21 [-0.20, 0.62]
Liu, S.2016 3d	-0.57	0.95	29	-0.82	0.7	29	13.0%	0.25 [-0.18, 0.68]
Liu, S.2016 after operation	-0.06	0.9	29	-0.19	0.95	29	10.6%	0.13 [-0.35, 0.61]
Woo, J. H.2015 1d	0.33	1.04	20	0.04	0.92	20	6.5%	0.29 [-0.32, 0.90]
Woo, J. H.2015 1h	0.62	1.04	20	0.42	0.92	20	6.5%	0.20 [-0.41, 0.81]
Zhang, T.2014 after operation	-0.5	0.94	19	-0.6	0.86	18	7.1%	0.10 [-0.48, 0.68]
Zhang, T.2014 1d	-0.5	1	19	-0.7	0.86	18	6.7%	0.20 [-0.40, 0.80]
Zhang, T.2014 2d	-0.4	0.94	19	-0.8	0.86	18	7.1%	0.40 [-0.18, 0.98]
Zhang, T.2014 3d	-0.5	1.13	19	-0.6	0.99	18	5.1%	0.10 [-0.58, 0.78]
Zhang, T.2014 3h	-0.5	1.3	19	-0.6	1.06	18	4.1%	0.10 [-0.66, 0.86]
Zhang, T.2014 5h	-0.1	1.28	19	-0.5	1.06	18	4.2%	0.40 [-0.36, 1.16]
Zhang, T.2014 anesthesia1h	-0.1	1.13	19	-0.1	1.14	18	4.5%	0.00 [-0.73, 0.73]
**Total (95% CI)**			**289**			**282**	**100.0%**	**0.20 [0.04, 0.35]**

Heterogeneity: Chi² = 1.54, df = 12 (P = 1.00); I² = 0%
Test for overall effect: Z = 2.51 (P = 0.01)

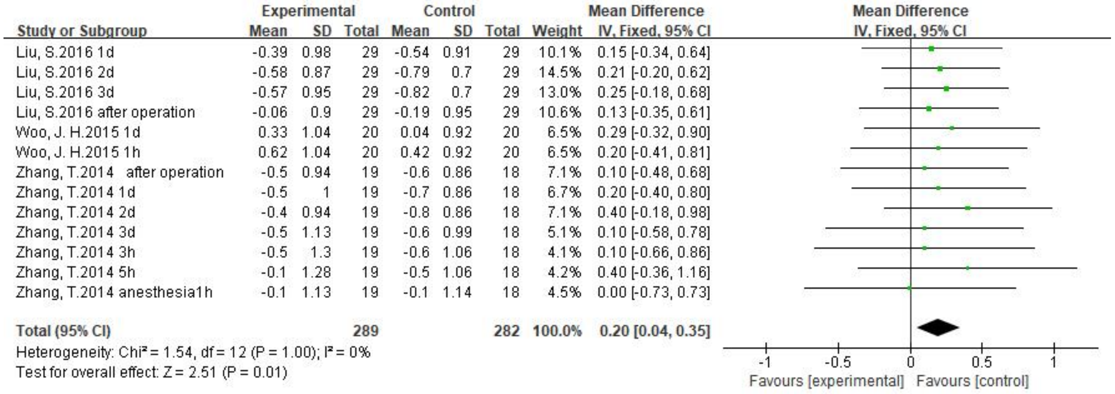

1.51 CD3(SEV)

Study or Subgroup	Experimental Mean	Experimental SD	Experimental Total	Control Mean	Control SD	Control Total	Weight	Mean Difference IV, Fixed, 95% CI
Liu, S.2016 1d	-0.82	0.74	29	-0.4	0.66	29	27.2%	-0.42 [-0.78, -0.06]
Liu, S.2016 2d	-0.89	0.74	29	-0.45	0.91	29	19.4%	-0.44 [-0.87, -0.01]
Liu, S.2016 3d	-0.48	0.74	29	-0.15	0.68	29	26.5%	-0.33 [-0.70, 0.04]
Liu, S.2016 after operation	-0.28	0.74	29	-0.07	0.68	29	26.5%	-0.21 [-0.58, 0.16]
Zhang, T.2014 1d	-10.1	13.59	18	-9.7	11.74	19	0.1%	-0.40 [-8.60, 7.80]
Zhang, T.2014 2d	-15.2	12.4	18	-10.6	12	19	0.1%	-4.60 [-12.47, 3.27]
Zhang, T.2014 3d	-15.8	12.23	18	-10.9	12.39	19	0.1%	-4.90 [-12.83, 3.03]
Zhang, T.2014 3h	-13.8	13.66	18	-11	11.38	19	0.1%	-2.80 [-10.92, 5.32]
Zhang, T.2014 5h	-16.6	12.76	18	-9.8	11.26	19	0.1%	-6.80 [-14.57, 0.97]
Zhang, T.2014 after operation	-13.4	12.46	18	-1.9	11.44	19	0.1%	-11.50 [-19.22, -3.78]
Zhang, T.2014 anesthesia1h	-1.6	14	18	1.2	13.15	19	0.0%	-2.80 [-11.56, 5.96]
**Total (95% CI)**			**242**			**249**	**100.0%**	**-0.36 [-0.55, -0.17]**

Heterogeneity: Chi² = 14.57, df = 10 (P = 0.15); I² = 31%
Test for overall effect: Z = 3.77 (P = 0.0002)

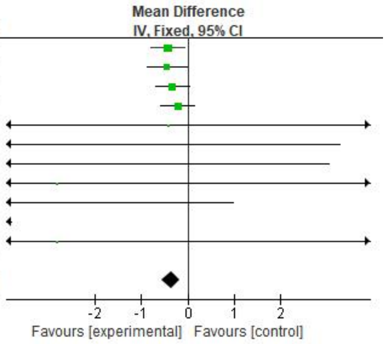

1.52 CD4(SEV)

Study or Subgroup	Experimental Mean	Experimental SD	Experimental Total	Control Mean	Control SD	Control Total	Weight	Mean Difference IV, Random, 95% CI
Liu, S.2016 1d	-0.4	0.45	29	-0.23	0.42	29	24.8%	-0.17 [-0.39, 0.05]
Liu, S.2016 2d	-0.44	0.42	29	-0.24	0.43	29	25.0%	-0.20 [-0.42, 0.02]
Liu, S.2016 3d	-0.38	0.44	29	-0.26	0.42	29	24.9%	-0.12 [-0.34, 0.10]
Liu, S.2016 after operation	-0.23	0.45	29	-0.09	0.44	29	24.6%	-0.14 [-0.37, 0.09]
Zhang, T.2014 1d	-11.6	12.17	18	-6.7	12.36	19	0.1%	-4.90 [-12.81, 3.01]
Zhang, T.2014 2d	-15.6	12.8	18	-5.9	11.68	19	0.1%	-9.70 [-17.61, -1.79]
Zhang, T.2014 3d	-15.1	12.17	18	-6.3	13.22	19	0.1%	-8.80 [-16.98, -0.62]
Zhang, T.2014 3h	-10.4	11.45	18	-5.3	12.55	19	0.1%	-5.10 [-12.83, 2.63]
Zhang, T.2014 5h	-10.7	11.26	18	-5.9	12.29	19	0.1%	-4.80 [-12.39, 2.79]
Zhang, T.2014 after operation	-10.6	12.03	18	2.3	13.51	19	0.1%	-12.90 [-21.13, -4.67]
Zhang, T.2014 anesthesia1h	-1.9	12.59	18	1.1	12.68	19	0.1%	-3.00 [-11.14, 5.14]
**Total (95% CI)**			**242**			**249**	**100.0%**	**-0.21 [-0.48, 0.06]**

Heterogeneity: Tau² = 0.06; Chi² = 24.20, df = 10 (P = 0.007); I² = 59%
Test for overall effect: Z = 1.55 (P = 0.12)

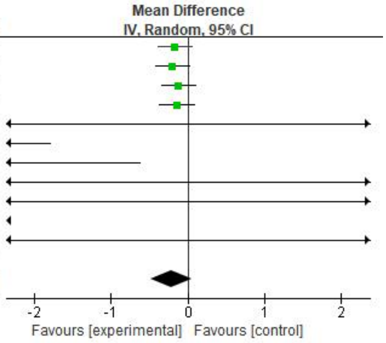

1.53 CD8(SEV)

Study or Subgroup	Experimental Mean	Experimental SD	Experimental Total	Control Mean	Control SD	Control Total	Weight	Mean Difference IV, Fixed, 95% CI
Liu, S.2016 1d	0.08	0.39	29	0.15	0.42	29	25.5%	-0.07 [-0.28, 0.14]
Liu, S.2016 2d	0.04	0.43	29	-0.06	0.4	29	24.3%	0.10 [-0.11, 0.31]
Liu, S.2016 3d	0.04	0.4	29	0.1	0.4	29	26.2%	-0.06 [-0.27, 0.15]
Liu, S.2016 after operation	0.15	0.42	29	0.06	0.42	29	23.8%	0.09 [-0.13, 0.31]
Zhang, T.2014 1d	1.1	14.21	18	-2.9	11.05	19	0.0%	4.00 [-4.23, 12.23]
Zhang, T.2014 2d	0.2	12.88	18	0.1	12.17	19	0.0%	0.10 [-7.98, 8.18]
Zhang, T.2014 3d	2	13.8	18	1.1	11.39	19	0.0%	0.90 [-7.28, 9.08]
Zhang, T.2014 3h	1.7	12.7	18	2	12.39	19	0.0%	-0.30 [-8.39, 7.79]
Zhang, T.2014 5h	2.1	12.35	18	-0.3	11.74	19	0.0%	2.40 [-5.37, 10.17]
Zhang, T.2014 after operation	-2	13.8	18	-0.4	12.17	19	0.0%	-1.60 [-10.00, 6.80]
Zhang, T.2014 anesthesia1h	-0.8	13.66	18	-0.8	12.84	19	0.0%	0.00 [-8.55, 8.55]
**Total (95% CI)**			**242**			**249**	**100.0%**	**0.01 [-0.09, 0.12]**

Heterogeneity: Chi² = 3.67, df = 10 (P = 0.96); I² = 0%
Test for overall effect: Z = 0.24 (P = 0.81)

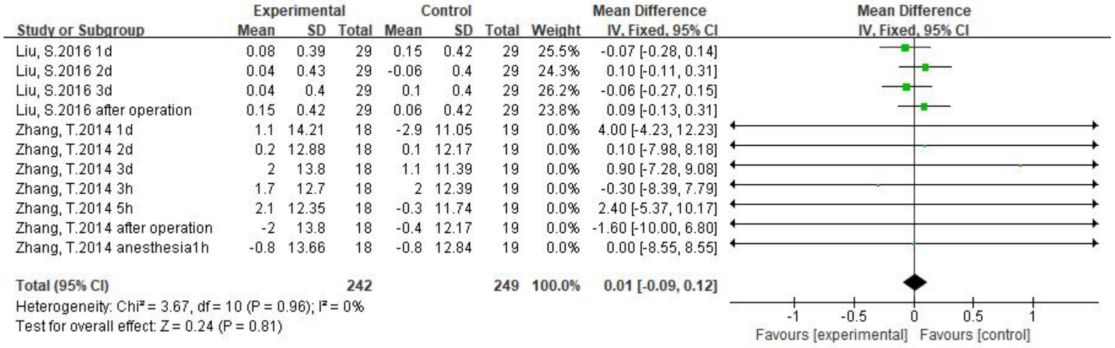

1.54 CD4/CD8(SEV)

Study or Subgroup	Experimental Mean	Experimental SD	Experimental Total	Control Mean	Control SD	Control Total	Weight	Mean Difference IV, Fixed, 95% CI
Liu, S.2016 1d	-0.54	0.91	29	-0.39	0.98	29	11.6%	-0.15 [-0.64, 0.34]
Liu, S.2016 2d	-0.79	0.7	29	-0.58	0.87	29	16.7%	-0.21 [-0.62, 0.20]
Liu, S.2016 3d	-0.82	0.7	29	-0.57	0.95	29	14.9%	-0.25 [-0.68, 0.18]
Liu, S.2016 after operation	-0.19	0.95	29	-0.06	0.9	29	12.1%	-0.13 [-0.61, 0.35]
Zhang, T.2014 1d	-0.6	0.86	18	-0.5	0.94	19	8.2%	-0.10 [-0.68, 0.48]
Zhang, T.2014 2d	-0.7	0.86	18	-0.5	1	19	7.7%	-0.20 [-0.80, 0.40]
Zhang, T.2014 3d	-0.8	0.86	18	-0.4	0.94	19	8.2%	-0.40 [-0.98, 0.18]
Zhang, T.2014 3h	-0.6	0.99	18	-0.5	1.13	19	5.9%	-0.10 [-0.78, 0.58]
Zhang, T.2014 5h	-0.6	1.06	18	-0.5	1.3	19	4.7%	-0.10 [-0.86, 0.66]
Zhang, T.2014 after operation	-0.5	1.06	18	-0.1	1.28	19	4.8%	-0.40 [-1.16, 0.36]
Zhang, T.2014 anesthesia1h	-0.1	1.14	18	-0.1	1.13	19	5.1%	0.00 [-0.73, 0.73]
**Total (95% CI)**			**242**			**249**	**100.0%**	**-0.19 [-0.36, -0.03]**

Heterogeneity: Chi² = 1.44, df = 10 (P = 1.00); I² = 0%
Test for overall effect: Z = 2.26 (P = 0.02)

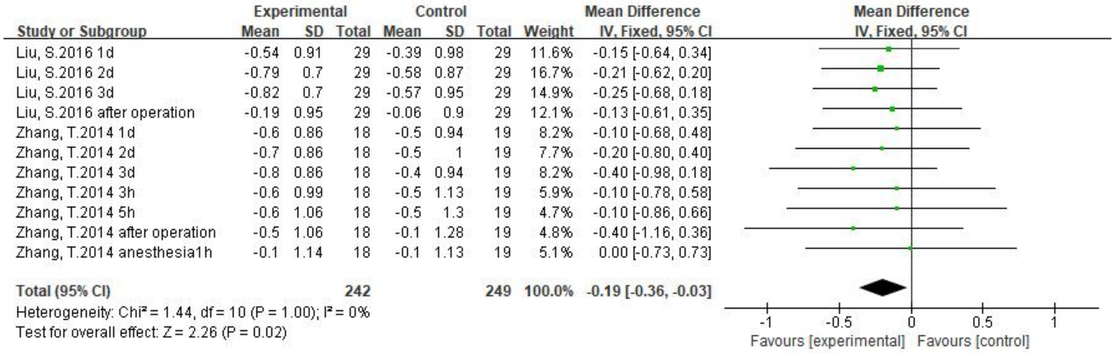

1.55CD3(Fentanyl)

Study or Subgroup	Experimental Mean	Experimental SD	Experimental Total	Control Mean	Control SD	Control Total	Weight	Mean Difference IV, Random, 95% CI
Ai, Z.2023 1d	-6.9	4.58	52	-2.65	4.96	52	11.0%	-4.25 [-6.08, -2.42]
Ai, Z.2023 3d	-4.25	4.59	52	-1.23	4.62	52	11.1%	-3.02 [-4.79, -1.25]
Gao, Y. F.2014 1d	-0.3	9.78	20	-7.82	9.41	20	7.2%	7.52 [1.57, 13.47]
Gao, Y. F.2014 3d	-14.45	9.78	20	-12.73	9.41	20	7.2%	-1.72 [-7.67, 4.23]
Gao, Y. F.2014 5d	-10.68	9.78	20	-0.83	9.41	20	7.2%	-9.85 [-15.80, -3.90]
Gao, Y. F.2014 after operation	0.32	9.78	20	0.89	9.41	20	7.2%	-0.57 [-6.52, 5.38]
Li, W. K.2008 1d	-13.7	8.57	16	-16.2	7.25	16	7.7%	2.50 [-3.00, 8.00]
Li, W. K.2008 3d	-14.9	10.26	16	-21.8	9.27	16	6.5%	6.90 [0.12, 13.68]
Li, W. K.2008 4h	-6.5	9.08	16	-15	8.37	16	7.2%	8.50 [2.45, 14.55]
Li, W. K.2008 after operation	-1.6	10.51	16	-3	9.56	16	6.4%	1.40 [-5.56, 8.36]
Lin, Y.2019 1d	-15.47	13.43	34	-10.72	15.55	35	6.4%	-4.75 [-11.60, 2.10]
Lin, Y.2019 2d	-3.59	13.88	34	-0.77	12.71	35	6.9%	-2.82 [-9.10, 3.46]
Shao, Y. J.2018 1d	-22.18	13.13	44	-12.86	12.36	44	7.8%	-9.32 [-14.65, -3.99]
**Total (95% CI)**			**360**			**362**	**100.0%**	**-1.03 [-3.63, 1.56]**

Heterogeneity: Tau² = 15.03; Chi² = 52.22, df = 12 (P < 0.00001); I² = 77%
Test for overall effect: Z = 0.78 (P = 0.44)

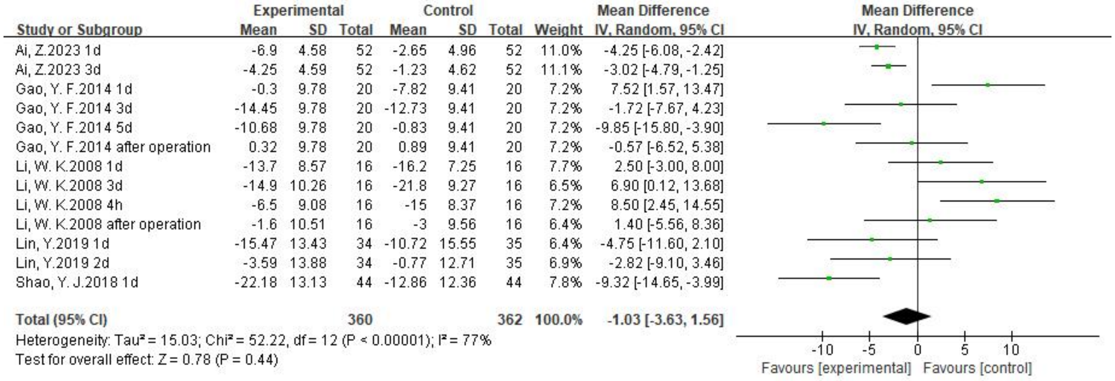

1.56 CD4(Fentanyl)

Study or Subgroup	Experimental Mean	Experimental SD	Experimental Total	Control Mean	Control SD	Control Total	Weight	Mean Difference IV, Random, 95% CI
Ai, Z.2023 1d	-6.38	3.81	52	-0.41	4.37	52	12.2%	-5.97 [-7.55, -4.39]
Ai, Z.2023 3d	-4.02	3.76	52	2.59	3.99	52	12.3%	-6.61 [-8.10, -5.12]
Gao, Y. F.2014 1d	-7.53	8.31	20	-5.34	8.18	20	7.1%	-2.19 [-7.30, 2.92]
Gao, Y. F.2014 3d	-13.59	8.31	20	-11.41	8.18	20	7.1%	-2.18 [-7.29, 2.93]
Gao, Y. F.2014 5d	-9.8	8.31	20	-0.59	8.18	20	7.1%	-9.21 [-14.32, -4.10]
Gao, Y. F.2014 after operation	-0.9	8.31	20	0.05	8.18	20	7.1%	-0.95 [-6.06, 4.16]
Li, W. K.2008 1d	-14.6	8.88	16	-16.9	7.38	16	6.4%	2.30 [-3.36, 7.96]
Li, W. K.2008 3d	-15.2	8.82	16	-17.5	6.6	16	6.7%	2.30 [-3.10, 7.70]
Li, W. K.2008 4h	-4.3	11.06	16	-10.9	10.26	16	4.7%	6.60 [-0.79, 13.99]
Li, W. K.2008 after operation	-0.9	7.96	16	-0.6	7.38	16	6.8%	-0.30 [-5.62, 5.02]
Lin, Y.2019 1d	-11.41	9.56	34	-8.1	11	35	7.4%	-3.31 [-8.17, 1.55]
Lin, Y.2019 2d	-3.7	8.23	34	-0.79	9.42	35	8.4%	-2.91 [-7.08, 1.26]
Shao, Y. J.2018 1d	-11.72	12.69	44	-7.91	13.22	44	6.7%	-3.81 [-9.22, 1.60]
**Total (95% CI)**			**360**			**362**	**100.0%**	**-2.73 [-4.72, -0.73]**

Heterogeneity: Tau² = 7.80; Chi² = 40.68, df = 12 (P < 0.0001); I² = 71%
Test for overall effect: Z = 2.68 (P = 0.007)

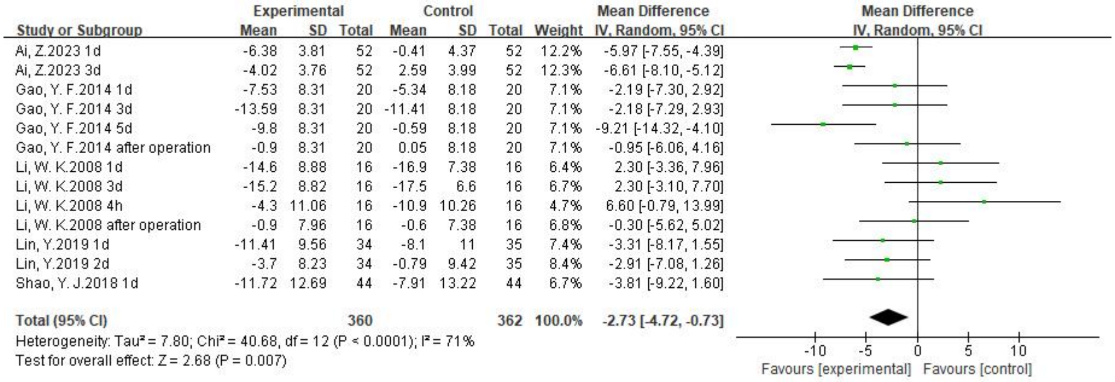

1.57 CD8(Fentanyl)

Study or Subgroup	Experimental Mean	Experimental SD	Experimental Total	Control Mean	Control SD	Control Total	Weight	Mean Difference IV, Fixed, 95% CI
Ai, Z.2023 1d	-0.57	2.35	52	-2.22	1.96	52	44.2%	1.65 [0.82, 2.48]
Ai, Z.2023 3d	-0.25	2.74	52	-3.83	1.89	52	37.4%	3.58 [2.68, 4.48]
Gao, Y. F.2014 1d	0.03	6.73	20	0	6.51	20	1.8%	0.03 [-4.07, 4.13]
Gao, Y. F.2014 3d	-0.09	6.73	20	-0.17	6.51	20	1.8%	0.08 [-4.02, 4.18]
Gao, Y. F.2014 5d	0.38	6.73	20	0.24	6.51	20	1.8%	0.14 [-3.96, 4.24]
Gao, Y. F.2014 after operation	0.08	6.73	20	-0.06	6.51	20	1.8%	0.14 [-3.96, 4.24]
Li, W. K.2008 1d	0.9	7	16	0.3	7.61	16	1.2%	0.60 [-4.47, 5.67]
Li, W. K.2008 3d	1.3	7.28	16	0.7	6.79	16	1.3%	0.60 [-4.28, 5.48]
Li, W. K.2008 4h	0.9	6.19	16	0.8	7.45	16	1.4%	0.10 [-4.65, 4.85]
Li, W. K.2008 after operation	0.3	7.28	16	0.4	7.08	16	1.2%	-0.10 [-5.08, 4.88]
Lin, Y.2019 1d	-4.06	8.29	34	-2.62	8.27	35	2.0%	-1.44 [-5.35, 2.47]
Lin, Y.2019 2d	0.23	8.03	34	0.02	5.99	35	2.7%	0.21 [-3.14, 3.56]
Shao, Y. J.2018 1d	0.96	11.39	44	0.79	11.59	44	1.3%	0.17 [-4.63, 4.97]
**Total (95% CI)**			**360**			**362**	**100.0%**	**2.07 [1.52, 2.62]**

Heterogeneity: Chi² = 22.18, df = 12 (P = 0.04); I² = 46%
Test for overall effect: Z = 7.33 (P < 0.00001)

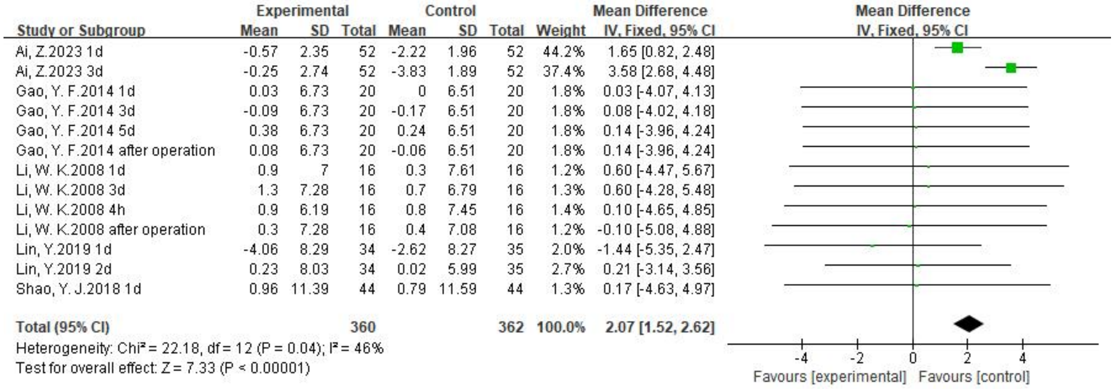

1.58 CD4/CD8(Fentanyl)

Study or Subgroup	Experimental Mean	Experimental SD	Experimental Total	Control Mean	Control SD	Control Total	Weight	Mean Difference IV, Random, 95% CI
Ai, Z.2023 1d	-0.18	0.15	52	0.09	0.16	52	14.1%	-0.27 [-0.33, -0.21]
Ai, Z.2023 3d	-0.11	0.17	52	0.28	0.15	52	14.1%	-0.39 [-0.45, -0.33]
Gao, Y. F.2014 1d	-0.4	0.39	20	-0.3	0.37	20	7.9%	-0.10 [-0.34, 0.14]
Gao, Y. F.2014 3d	-0.58	0.39	20	-0.48	0.37	20	7.9%	-0.10 [-0.34, 0.14]
Gao, Y. F.2014 5d	-0.36	0.39	20	-0.12	0.37	20	7.9%	-0.24 [-0.48, -0.00]
Gao, Y. F.2014 after operation	-0.12	0.39	20	0	0.37	20	7.9%	-0.12 [-0.36, 0.12]
Li, W. K.2008 1d	-0.4	0.45	16	-0.4	0.42	16	6.1%	0.00 [-0.30, 0.30]
Li, W. K.2008 3d	-0.5	0.41	16	-0.5	0.36	16	6.9%	0.00 [-0.27, 0.27]
Li, W. K.2008 4h	-0.2	0.45	16	-0.3	0.32	16	6.9%	0.10 [-0.17, 0.37]
Li, W. K.2008 after operation	-0.1	0.5	16	-0.1	0.5	16	5.1%	0.00 [-0.35, 0.35]
Lin, Y.2019 1d	-0.24	0.6	34	-0.17	0.62	35	6.4%	-0.07 [-0.36, 0.22]
Lin, Y.2019 2d	-0.18	0.42	34	-0.08	0.45	35	8.9%	-0.10 [-0.31, 0.11]
**Total (95% CI)**			**316**			**318**	**100.0%**	**-0.14 [-0.24, -0.05]**

Heterogeneity: Tau² = 0.02; Chi² = 41.17, df = 11 (P < 0.0001); I² = 73%
Test for overall effect: Z = 2.92 (P = 0.004)

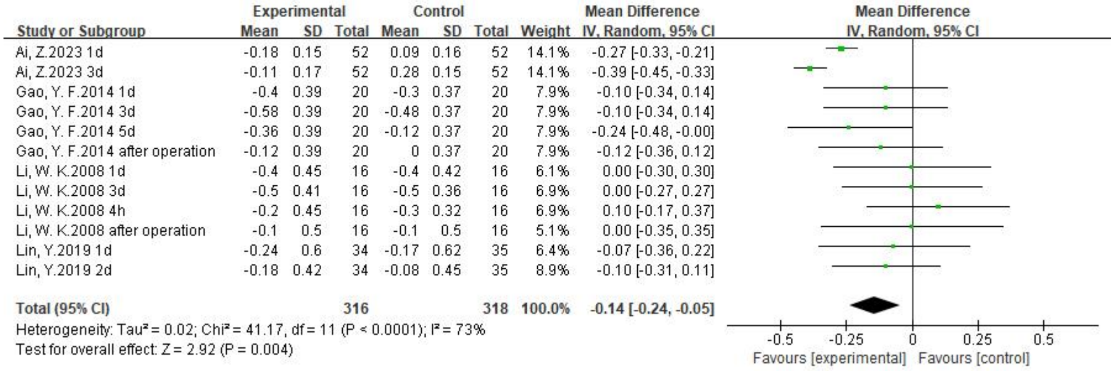

1.59 NK(Fentanyl)

Study or Subgroup	Experimental Mean	Experimental SD	Experimental Total	Control Mean	Control SD	Control Total	Weight	Mean Difference IV, Fixed, 95% CI
Gao, Y. F.2014 1d	-7.08	10.8	20	-6.06	10.6	20	16.1%	-1.02 [-7.65, 5.61]
Gao, Y. F.2014 3d	-8.73	10.8	20	-7.16	10.6	20	16.1%	-1.57 [-8.20, 5.06]
Gao, Y. F.2014 5d	-4.43	10.8	20	-1.21	10.6	20	16.1%	-3.22 [-9.85, 3.41]
Gao, Y. F.2014 after operation	-1.37	10.8	20	-0.3	10.6	20	16.1%	-1.07 [-7.70, 5.56]
Shao, Y. J.2018 1d	-8.94	10.8	44	-5.02	10.6	44	35.5%	-3.92 [-8.39, 0.55]
**Total (95% CI)**			**124**			**124**	**100.0%**	**-2.50 [-5.16, 0.16]**

Heterogeneity: Chi² = 0.88, df = 4 (P = 0.93); I² = 0%
Test for overall effect: Z = 1.84 (P = 0.07)

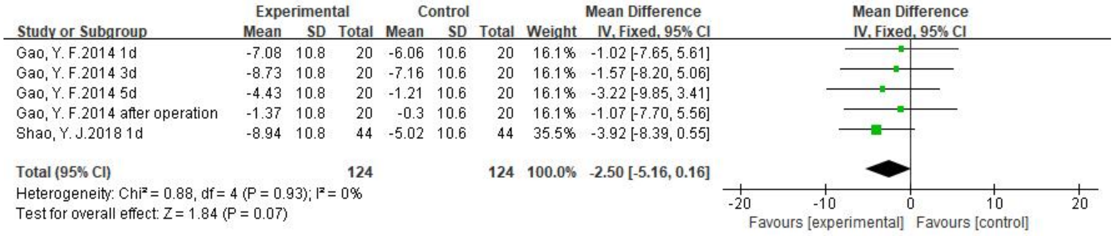

1.60 CD3(Flurbiprofen)

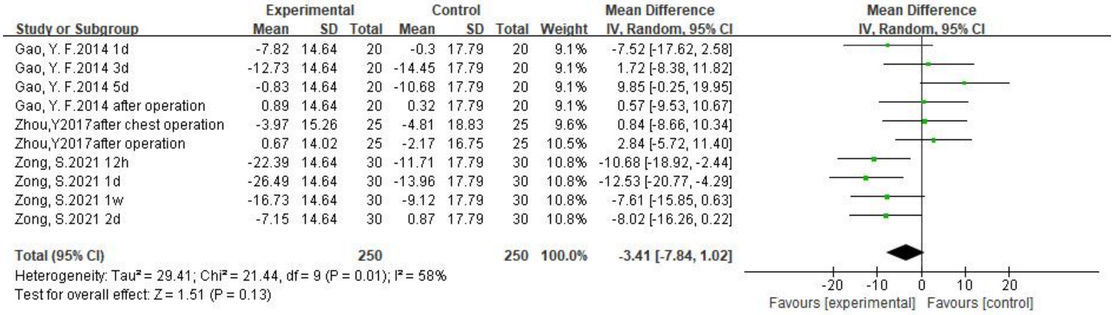

1.61 CD4(Flurbiprofen)

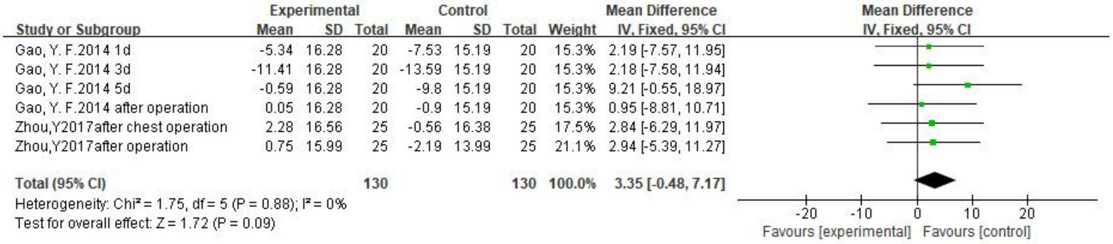

1.62 CD8(Flurbiprofen)

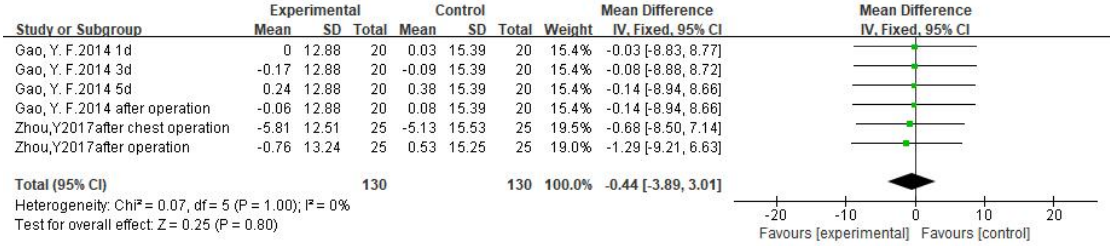

1.63 CD4/CD8(Flurbiprofen)

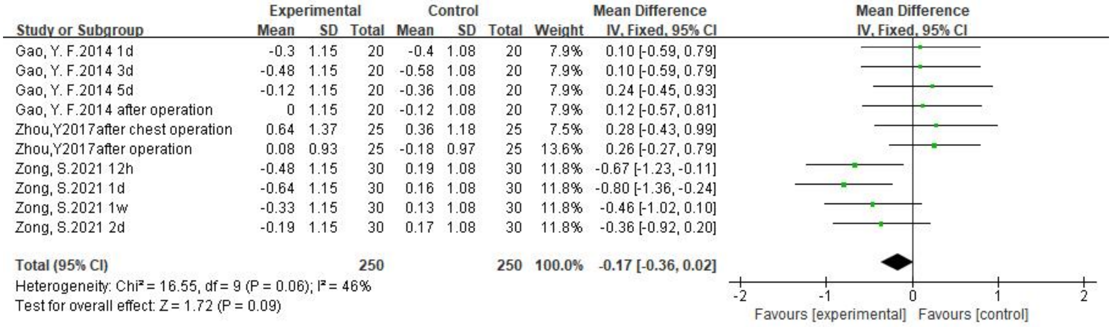

1.64 CD3(Parecoxib)

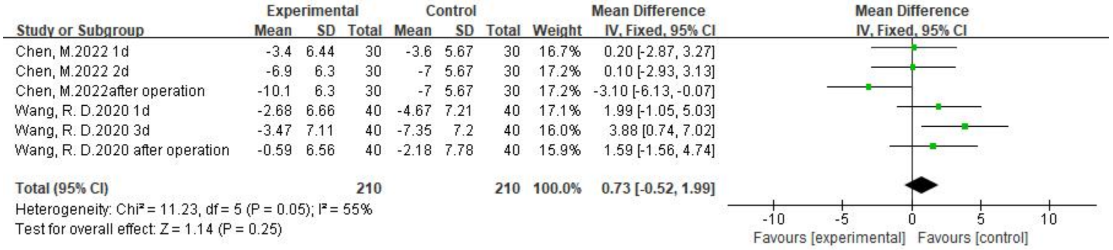

1.65 CD4(Parecoxib)

Study or Subgroup	Experimental Mean	Experimental SD	Experimental Total	Control Mean	Control SD	Control Total	Weight	Mean Difference IV, Random, 95% CI
Chen, M.2022 1d	-3.1	3.33	30	-5.5	3.26	30	20.4%	2.40 [0.73, 4.07]
Chen, M.2022 2d	-5.7	3.2	30	-3.3	3.06	30	20.7%	-2.40 [-3.98, -0.82]
Chen, M.2022after operation	-7.9	3.54	30	-6.7	3.54	30	19.9%	-1.20 [-2.99, 0.59]
Wang, R. D.2020 1d	-2.52	7.57	40	-3.42	7.99	40	13.7%	0.90 [-2.51, 4.31]
Wang, R. D.2020 3d	-4.32	8.01	40	-6.55	8.86	40	12.7%	2.23 [-1.47, 5.93]
Wang, R. D.2020 after operation	-0.15	8.8	40	-1.12	8.28	40	12.6%	0.97 [-2.77, 4.71]
**Total (95% CI)**			**210**			**210**	**100.0%**	**0.28 [-1.65, 2.21]**

Heterogeneity: Tau² = 4.03; Chi² = 20.34, df = 5 (P = 0.001); I² = 75%
Test for overall effect: Z = 0.29 (P = 0.77)

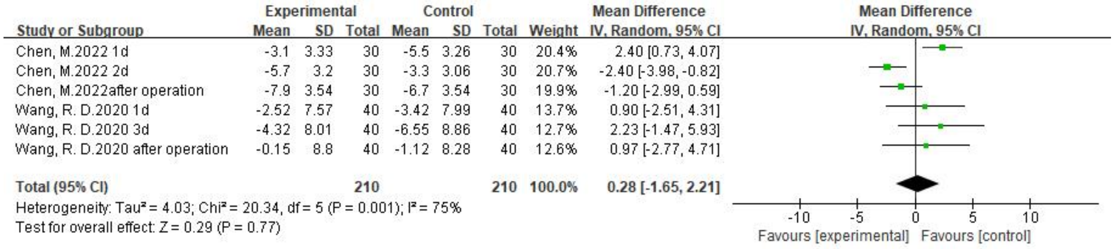

1.66 CD8(Parecoxib)

Study or Subgroup	Experimental Mean	Experimental SD	Experimental Total	Control Mean	Control SD	Control Total	Weight	Mean Difference IV, Fixed, 95% CI
Chen, M.2022 1d	-1.3	2.41	30	-2.4	2.34	30	29.3%	1.10 [-0.10, 2.30]
Chen, M.2022 2d	-2.7	2.26	30	-1.5	2.34	30	31.2%	-1.20 [-2.36, -0.04]
Chen, M.2022after operation	-3.7	2.26	30	-4.9	2.69	30	26.8%	1.20 [-0.06, 2.46]
Wang, R. D.2020 1d	-0.65	7.96	40	-0.23	6.15	40	4.4%	-0.42 [-3.54, 2.70]
Wang, R. D.2020 3d	-1.03	8.2	40	-0.57	5.99	40	4.3%	-0.46 [-3.61, 2.69]
Wang, R. D.2020 after operation	-0.33	8	40	0.57	6.51	40	4.1%	-0.90 [-4.10, 2.30]
**Total (95% CI)**			**210**			**210**	**100.0%**	**0.19 [-0.46, 0.84]**

Heterogeneity: Chi² = 10.92, df = 5 (P = 0.05); I² = 54%
Test for overall effect: Z = 0.58 (P = 0.56)

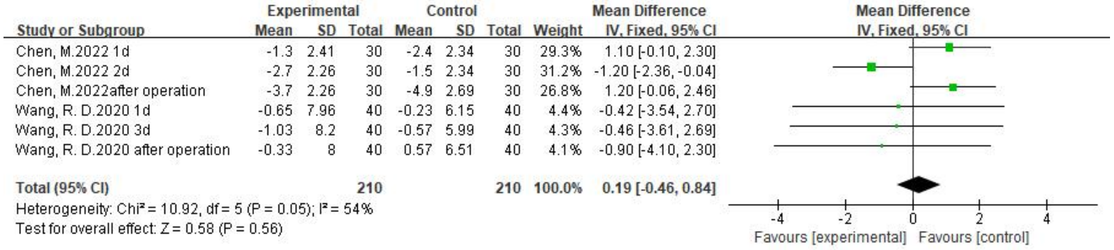

1.67 CD4/CD8(Parecoxib)

Study or Subgroup	Experimental Mean	Experimental SD	Experimental Total	Control Mean	Control SD	Control Total	Weight	Mean Difference IV, Fixed, 95% CI
Wang, R. D.2020 1d	-0.08	0.55	40	-0.14	0.65	40	27.7%	0.06 [-0.20, 0.32]
Wang, R. D.2020 3d	-0.13	0.054	40	-0.25	0.66	40	45.8%	0.12 [-0.09, 0.33]
Wang, R. D.2020 after operation	0	0.59	40	-0.1	0.64	40	26.5%	0.10 [-0.17, 0.37]
**Total (95% CI)**			**120**			**120**	**100.0%**	**0.10 [-0.04, 0.24]**

Heterogeneity: Chi² = 0.12, df = 2 (P = 0.94); I² = 0%
Test for overall effect: Z = 1.38 (P = 0.17)

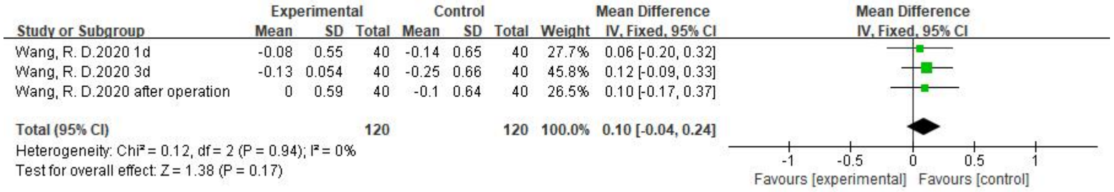

1.68 NK(Parecoxib)

Study or Subgroup	Experimental Mean	Experimental SD	Experimental Total	Control Mean	Control SD	Control Total	Weight	Mean Difference IV, Fixed, 95% CI
Chen, M.2022 1d	-2.6	3.75	30	-1.9	3.25	30	21.9%	-0.70 [-2.48, 1.08]
Chen, M.2022 2d	-3.9	3.34	30	-1.2	3.18	30	25.4%	-2.70 [-4.35, -1.05]
Chen, M.2022after operation	-4.9	3.05	30	-3	3.05	30	29.0%	-1.90 [-3.44, -0.36]
Wang, R. D.2020 1d	-2.57	5.67	40	-2.82	7.26	40	8.5%	0.25 [-2.60, 3.10]
Wang, R. D.2020 3d	0.09	6.22	40	-2.25	7.31	40	7.8%	2.34 [-0.63, 5.31]
Wang, R. D.2020 after operation	0.43	6.46	40	-0.98	7.38	40	7.5%	1.41 [-1.63, 4.45]
**Total (95% CI)**			**210**			**210**	**100.0%**	**-1.08 [-1.91, -0.25]**

Heterogeneity: Chi² = 13.45, df = 5 (P = 0.02); I² = 63%
Test for overall effect: Z = 2.55 (P = 0.01)

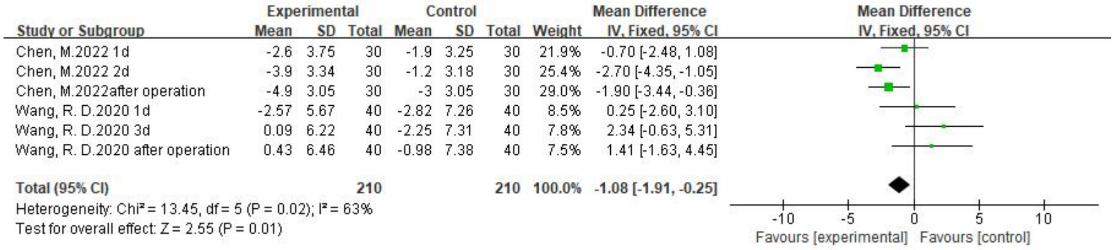

Morphine

1.69 CD3(Morphine)

Study or Subgroup	Experimental Mean	Experimental SD	Experimental Total	Control Mean	Control SD	Control Total	Weight	Mean Difference IV, Fixed, 95% CI
Bakr, M. A. E. M.2016KETOROL1d	-10.98	6.15	20	-8.47	3.63	20	10.2%	-2.51 [-5.64, 0.62]
Bakr, M. A. E. M.2016KETOROL90min	-19.37	6.24	20	-19.17	4.95	20	8.2%	-0.20 [-3.69, 3.29]
Bakr, M. A. E. M.2016TRAM1d	-10.98	6.15	20	-11.89	5.99	20	7.0%	0.91 [-2.85, 4.67]
Bakr, M. A. E. M.2016TRAM90min	-19.37	6.24	20	-21.6	5.1	20	8.0%	2.23 [-1.30, 5.76]
Li, W. K.2008 1d	-16.2	7.25	16	-13.7	8.57	16	3.3%	-2.50 [-8.00, 3.00]
Li, W. K.2008 3d	-21.8	9.27	16	-14.9	10.26	16	2.2%	-6.90 [-13.68, -0.12]
Li, W. K.2008 4h	-15	8.37	16	-6.5	9.08	16	2.7%	-8.50 [-14.55, -2.45]
Li, W. K.2008after operation	-3	9.56	16	-1.6	10.51	16	2.1%	-1.40 [-8.36, 5.56]
Shen, J. C.2014 1d	-6.69	7.4	30	-5.26	7.26	29	7.1%	-1.43 [-5.17, 2.31]
Shen, J. C.2014 2d	-13.26	7.4	30	-8.74	7.26	29	7.1%	-4.52 [-8.26, -0.78]
Shen, J. C.2014 2h	-3.12	7.4	30	-3.1	7.26	29	7.1%	-0.02 [-3.76, 3.72]
Shen, J. C.2014 5d	-9.93	7.4	30	-6.91	7.26	29	7.1%	-3.02 [-6.76, 0.72]
Wang, Z. Y.2006TRAM+LORN1d	-3.44	7.4	14	-5.13	7.26	15	3.5%	1.69 [-3.65, 7.03]
Wang, Z. Y.2006TRAM+LORN2d	-10.69	7.4	14	-8.74	7.26	15	3.5%	-1.95 [-7.29, 3.39]
Wang, Z. Y.2006TRAM+LORN3d	-8.25	7.4	14	-2.48	7.26	15	3.5%	-5.77 [-11.11, -0.43]
Wang, Z. Y.2006TRAM+LORN90min	-1.46	7.4	14	-1.95	7.26	15	3.5%	0.49 [-4.85, 5.83]
Wang, Z. Y.2006TRAM1d	-3.44	7.4	14	-0.04	7.26	15	3.5%	-3.40 [-8.74, 1.94]
Wang, Z. Y.2006TRAM2d	-10.69	7.4	14	-2.35	7.26	15	3.5%	-8.34 [-13.68, -3.00]
Wang, Z. Y.2006TRAM3d	-8.25	7.4	14	-1.45	7.26	15	3.5%	-6.80 [-12.14, -1.46]
Wang, Z. Y.2006TRAM90min	-1.46	7.4	14	-0.61	7.26	15	3.5%	-0.85 [-6.19, 4.49]
**Total (95% CI)**			**376**			**380**	**100.0%**	**-2.03 [-3.03, -1.03]**

Heterogeneity: Chi² = 32.17, df = 19 (P = 0.03); I² = 41%
Test for overall effect: Z = 3.99 (P < 0.0001)

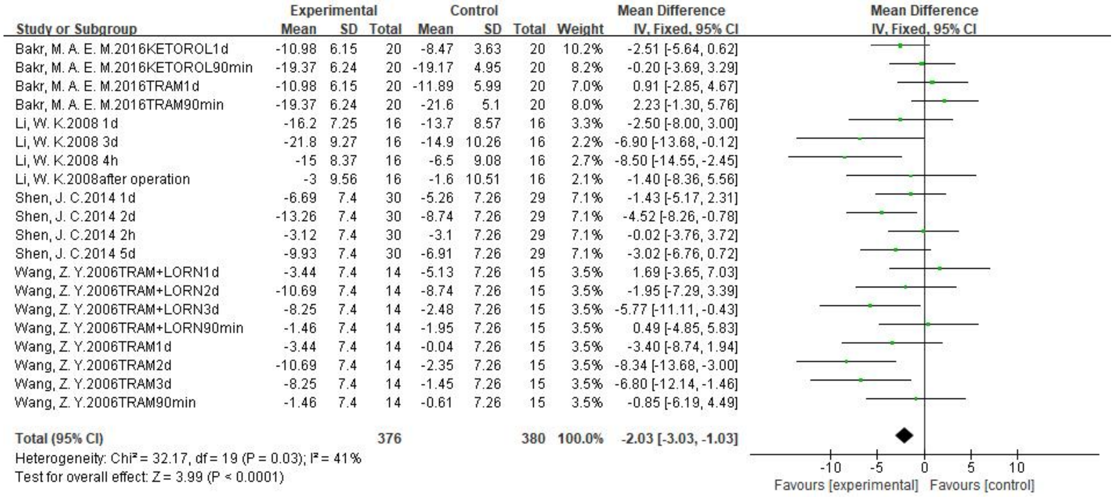

1.70 CD4(Morphine)

Study or Subgroup	Experimental Mean	Experimental SD	Experimental Total	Control Mean	Control SD	Control Total	Weight	Mean Difference IV, Random, 95% CI
Bakr, M. A. E. M.2016KETOROL1d	-21	3.07	20	-6.32	3.15	20	5.5%	-14.68 [-16.61, -12.75]
Bakr, M. A. E. M.2016KETOROL90min	-21.13	2.99	20	-13.54	2.67	20	5.5%	-7.59 [-9.35, -5.83]
Bakr, M. A. E. M.2016TRAM1d	-21	3.07	20	-5.1	5.62	20	5.3%	-15.90 [-18.71, -13.09]
Bakr, M. A. E. M.2016TRAM90min	-21.13	2.99	20	-17.2	4.59	20	5.4%	-3.93 [-6.33, -1.53]
Li, W. K.2008 1d	-16.9	7.38	16	-14.6	8.88	16	4.6%	-2.30 [-7.96, 3.36]
Li, W. K.2008 3d	-17.5	6.6	16	-15.2	8.82	16	4.6%	-2.30 [-7.70, 3.10]
Li, W. K.2008 4h	-10.9	10.26	16	-4.3	11.06	16	4.0%	-6.60 [-13.99, 0.79]
Li, W. K.2008after operation	-0.6	7.38	16	-0.9	7.96	16	4.7%	0.30 [-5.02, 5.62]
Shen, J. C.2014 1d	-4.51	5.47	30	-3.53	6.59	29	5.2%	-0.98 [-4.08, 2.12]
Shen, J. C.2014 2d	-8.95	5.47	30	-5.38	6.59	29	5.2%	-3.57 [-6.67, -0.47]
Shen, J. C.2014 2h	-1.95	5.47	30	-1.99	6.59	29	5.2%	0.04 [-3.06, 3.14]
Shen, J. C.2014 5d	-6.73	5.47	30	-4.94	6.59	29	5.2%	-1.79 [-4.89, 1.31]
Wang, Z. Y.2006TRAM+LORN1d	-5.19	5.47	14	-6.49	6.59	15	4.9%	1.30 [-3.10, 5.70]
Wang, Z. Y.2006TRAM+LORN2d	-4.98	5.47	14	-5.26	6.59	15	4.9%	0.28 [-4.12, 4.68]
Wang, Z. Y.2006TRAM+LORN3d	3.93	5.47	14	-1.09	6.59	15	4.9%	5.02 [0.62, 9.42]
Wang, Z. Y.2006TRAM+LORN90min	-0.29	5.47	14	-0.79	6.59	15	4.9%	0.50 [-3.90, 4.90]
Wang, Z. Y.2006TRAM1d	-5.19	5.47	14	-4.53	6.59	15	4.9%	-0.66 [-5.06, 3.74]
Wang, Z. Y.2006TRAM2d	-4.98	5.47	14	-0.92	6.59	15	4.9%	-4.06 [-8.46, 0.34]
Wang, Z. Y.2006TRAM3d	3.93	5.47	14	-0.86	6.59	15	4.9%	4.79 [0.39, 9.19]
Wang, Z. Y.2006TRAM90min	-0.29	5.47	14	-0.31	6.59	15	4.9%	0.02 [-4.38, 4.42]
**Total (95% CI)**			**376**			**380**	**100.0%**	**-2.71 [-5.53, 0.11]**

Heterogeneity: Tau² = 36.96; Chi² = 258.32, df = 19 (P < 0.00001); I² = 93%
Test for overall effect: Z = 1.89 (P = 0.06)

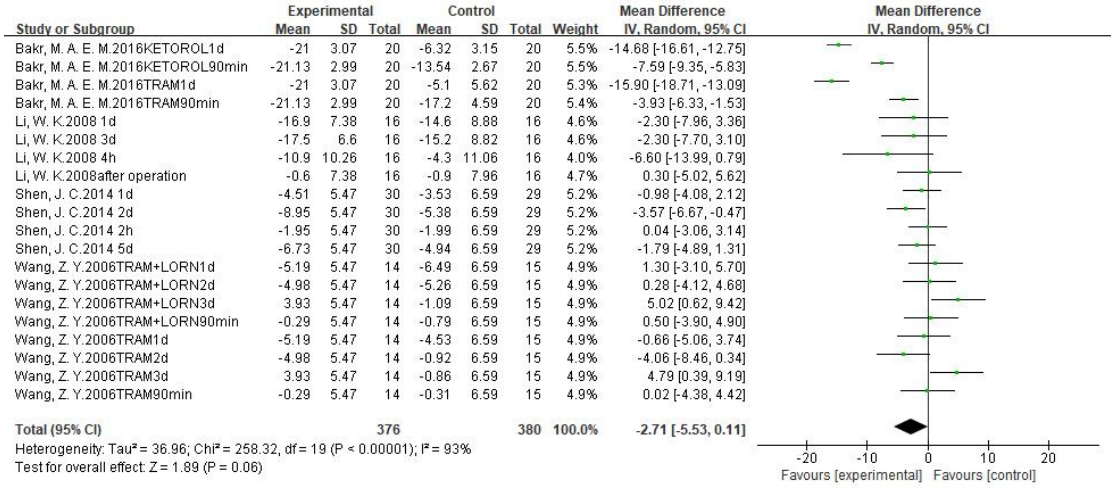

1.71 CD8(Morphine)

Study or Subgroup	Experimental Mean	Experimental SD	Experimental Total	Control Mean	Control SD	Control Total	Weight	Mean Difference IV, Random, 95% CI
Bakr, M. A. E. M.2016KETOROL1d	-14.33	3.44	20	-4.34	3.61	20	5.9%	-9.99 [-12.18, -7.80]
Bakr, M. A. E. M.2016KETOROL90min	-14.39	3.35	20	-13.13	4.86	20	5.6%	-1.26 [-3.85, 1.33]
Bakr, M. A. E. M.2016TRAM1d	-14.33	3.44	20	-6.38	4.27	20	5.8%	-7.95 [-10.35, -5.55]
Bakr, M. A. E. M.2016TRAM90min	-14.39	3.35	20	-12.54	3.45	20	5.9%	-1.85 [-3.96, 0.26]
Li, W. K.2008 1d	0.3	7.61	16	0.9	7	16	4.0%	-0.60 [-5.67, 4.47]
Li, W. K.2008 3d	0.7	6.79	16	1.3	7.28	16	4.1%	-0.60 [-5.48, 4.28]
Li, W. K.2008 4h	0.8	7.45	16	0.9	6.19	16	4.2%	-0.10 [-4.85, 4.65]
Li, W. K.2008after operation	0.4	7.08	16	0.3	7.28	16	4.1%	0.10 [-4.88, 5.08]
Shen, J. C.2014 1d	-0.89	5.31	30	-0.75	5.49	29	5.5%	-0.14 [-2.90, 2.62]
Shen, J. C.2014 2d	-1.82	5.31	30	-1.44	5.49	29	5.5%	-0.38 [-3.14, 2.38]
Shen, J. C.2014 2h	0.41	5.31	30	-0.78	5.49	29	5.5%	1.19 [-1.57, 3.95]
Shen, J. C.2014 5d	-1.5	5.31	30	-1.08	5.49	29	5.5%	-0.42 [-3.18, 2.34]
Wang, Z. Y.2006TRAM+LORN1d	0.21	5.31	14	1.37	5.49	15	4.8%	-1.16 [-5.09, 2.77]
Wang, Z. Y.2006TRAM+LORN2d	0.31	5.31	14	1.72	5.49	15	4.8%	-1.41 [-5.34, 2.52]
Wang, Z. Y.2006TRAM+LORN3d	0.7	5.31	14	0.77	5.49	15	4.8%	-0.07 [-4.00, 3.86]
Wang, Z. Y.2006TRAM+LORN90min	0.36	5.31	14	0.32	5.49	15	4.8%	0.04 [-3.89, 3.97]
Wang, Z. Y.2006TRAM1d	0.21	5.31	14	1.15	5.49	15	4.8%	-0.94 [-4.87, 2.99]
Wang, Z. Y.2006TRAM2d	0.31	5.31	14	0.02	5.49	15	4.8%	0.29 [-3.64, 4.22]
Wang, Z. Y.2006TRAM3d	0.7	5.31	14	0.04	5.49	15	4.8%	0.66 [-3.27, 4.59]
Wang, Z. Y.2006TRAM90min	0.36	5.31	14	0.07	5.49	15	4.8%	0.29 [-3.64, 4.22]
**Total (95% CI)**			**376**			**380**	**100.0%**	**-1.37 [-3.00, 0.26]**

Heterogeneity: Tau² = 10.53; Chi² = 93.30, df = 19 (P < 0.00001); I² = 80%
Test for overall effect: Z = 1.65 (P = 0.10)

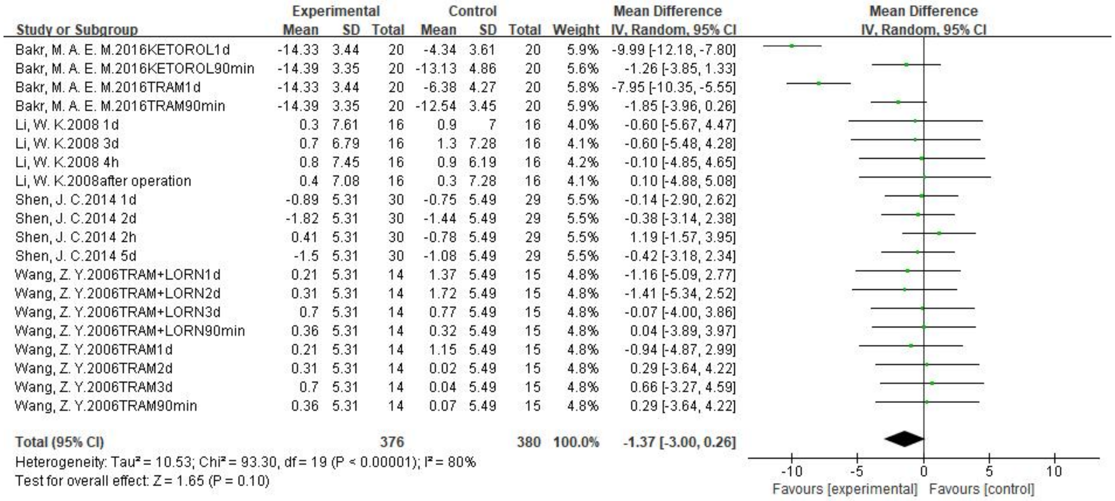

1.72 CD4/CD8(Morphine)

Study or Subgroup	Experimental Mean	Experimental SD	Experimental Total	Control Mean	Control SD	Control Total	Weight	Mean Difference IV, Random, 95% CI
Li, W. K.2008 1d	-0.4	0.42	16	-0.4	0.45	16	5.9%	0.00 [-0.30, 0.30]
Li, W. K.2008 3d	-0.5	0.36	16	-0.5	0.41	16	6.5%	0.00 [-0.27, 0.27]
Li, W. K.2008 4h	-0.3	0.32	16	-0.2	0.45	16	6.4%	-0.10 [-0.37, 0.17]
Li, W. K.2008after operation	-0.1	0.5	16	-0.1	0.5	16	5.2%	0.00 [-0.35, 0.35]
Shen, J. C.2014 1d	-0.11	0.4	30	-0.78	0.45	29	7.4%	0.67 [0.45, 0.89]
Shen, J. C.2014 2d	-0.22	0.4	30	-0.16	0.45	29	7.4%	-0.06 [-0.28, 0.16]
Shen, J. C.2014 2h	-0.1	0.4	30	-0.05	0.45	29	7.4%	-0.05 [-0.27, 0.17]
Shen, J. C.2014 5d	-0.22	0.4	30	-0.15	0.45	29	7.4%	-0.07 [-0.29, 0.15]
Wang, Z. Y.2006TRAM+LORN1d	-0.21	0.4	14	-0.31	0.45	15	5.8%	0.10 [-0.21, 0.41]
Wang, Z. Y.2006TRAM+LORN2d	-0.2	0.4	14	-0.31	0.45	15	5.8%	0.11 [-0.20, 0.42]
Wang, Z. Y.2006TRAM+LORN3d	-0.19	0.4	14	-0.09	0.45	15	5.8%	-0.10 [-0.41, 0.21]
Wang, Z. Y.2006TRAM+LORN90min	-0.02	0.4	14	-0.04	0.45	15	5.8%	0.02 [-0.29, 0.33]
Wang, Z. Y.2006TRAM1d	-0.21	0.4	14	-0.24	0.45	15	5.8%	0.03 [-0.28, 0.34]
Wang, Z. Y.2006TRAM2d	-0.2	0.4	14	-0.04	0.45	15	5.8%	-0.16 [-0.47, 0.15]
Wang, Z. Y.2006TRAM3d	-0.19	0.4	14	-0.03	0.45	15	5.8%	-0.16 [-0.47, 0.15]
Wang, Z. Y.2006TRAM90min	-0.02	0.4	14	-0.01	0.45	15	5.8%	-0.01 [-0.32, 0.30]
**Total (95% CI)**			**296**			**300**	**100.0%**	**0.02 [-0.09, 0.13]**

Heterogeneity: $Tau^2 = 0.03; Chi^2 = 40.33, df = 15 (P = 0.0004); I^2 = 63\%$
Test for overall effect: Z = 0.35 (P = 0.73)

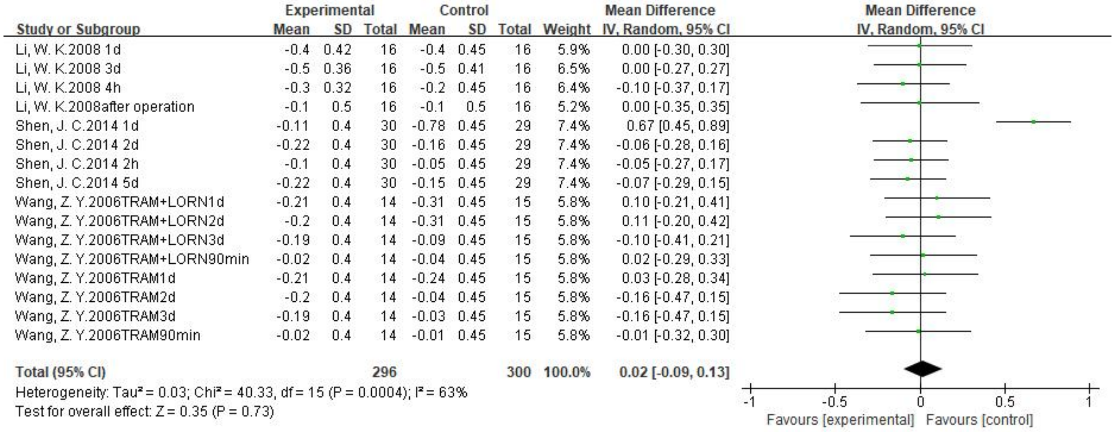

1.73 CD3(Dezocine)

Study or Subgroup	Experimental Mean	Experimental SD	Experimental Total	Control Mean	Control SD	Control Total	Weight	Mean Difference IV, Fixed, 95% CI
Gao, Y. F.2014 1d	-7.82	12.36	20	-8.09	13.13	20	16.1%	0.27 [-7.63, 8.17]
Gao, Y. F.2014 3d	-12.73	12.36	20	-14.45	13.13	20	16.1%	1.72 [-6.18, 9.62]
Gao, Y. F.2014 5d	-0.83	12.36	20	-10.68	13.13	20	16.1%	9.85 [1.95, 17.75]
Gao, Y. F.2014after operation	0.89	12.36	20	0.32	13.13	20	16.1%	0.57 [-7.33, 8.47]
Shao, Y. J.2018	-12.86	12.36	44	-22.18	13.13	44	35.5%	9.32 [3.99, 14.65]
**Total (95% CI)**			**124**			**124**	**100.0%**	**5.31 [2.13, 8.48]**

Heterogeneity: $Chi^2 = 7.18, df = 4 (P = 0.13); I^2 = 44\%$
Test for overall effect: Z = 3.28 (P = 0.001)

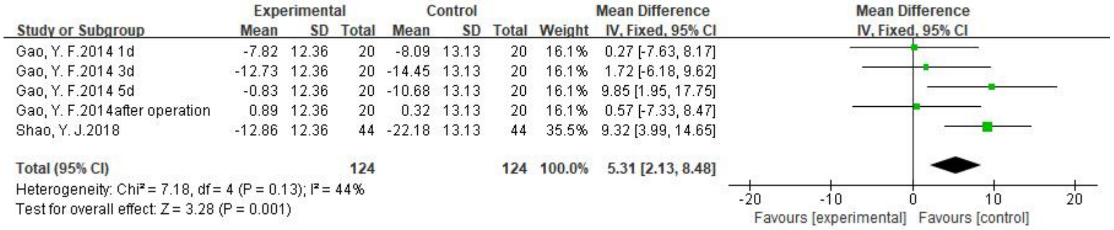

1.74 CD4(Dezocine)

Study or Subgroup	Experimental Mean	Experimental SD	Experimental Total	Control Mean	Control SD	Control Total	Weight	Mean Difference IV, Fixed, 95% CI
Gao, Y. F.2014 1d	-5.34	13.22	20	-7.53	12.69	20	16.1%	2.19 [-5.84, 10.22]
Gao, Y. F.2014 3d	-11.41	13.22	20	-13.59	12.69	20	16.1%	2.18 [-5.85, 10.21]
Gao, Y. F.2014 5d	-0.59	13.22	20	-9.8	12.69	20	16.1%	9.21 [1.18, 17.24]
Gao, Y. F.2014after operation	0.05	13.22	20	-0.9	12.69	20	16.1%	0.95 [-7.08, 8.98]
Shao, Y. J.2018	-7.91	13.22	44	-11.72	12.69	44	35.5%	3.81 [-1.60, 9.22]
**Total (95% CI)**			**124**			**124**	**100.0%**	**3.70 [0.47, 6.92]**

Heterogeneity: $Chi^2 = 2.53, df = 4 (P = 0.64); I^2 = 0\%$
Test for overall effect: Z = 2.25 (P = 0.02)

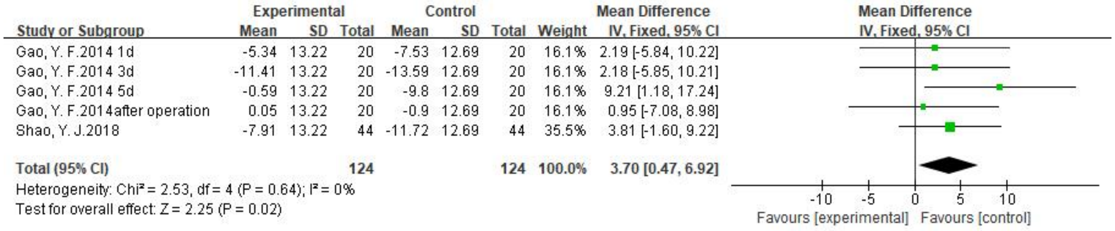

1.75 CD8(Dezocine)

Study or Subgroup	Experimental Mean	Experimental SD	Experimental Total	Control Mean	Control SD	Control Total	Weight	Mean Difference IV, Fixed, 95% CI
Gao, Y. F.2014 1d	0	11.59	20	0.03	11.39	20	16.1%	-0.03 [-7.15, 7.09]
Gao, Y. F.2014 3d	-0.17	11.59	20	-0.09	11.39	20	16.1%	-0.08 [-7.20, 7.04]
Gao, Y. F.2014 5d	0.24	11.59	20	0.38	11.39	20	16.1%	-0.14 [-7.26, 6.98]
Gao, Y. F.2014after operation	-0.06	11.59	20	0.08	11.39	20	16.1%	-0.14 [-7.26, 6.98]
Shao, Y. J.2018	0.79	11.59	44	0.96	11.39	44	35.5%	-0.17 [-4.97, 4.63]
**Total (95% CI)**			**124**			**124**	**100.0%**	**-0.12 [-2.98, 2.74]**

Heterogeneity: $Chi^2 = 0.00, df = 4 (P = 1.00); I^2 = 0\%$
Test for overall effect: Z = 0.08 (P = 0.93)

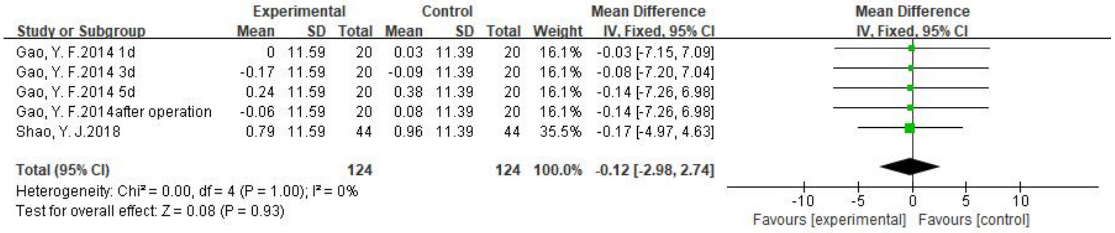

1.76NK(Dezocine)

Study or Subgroup	Experimental Mean	Experimental SD	Experimental Total	Control Mean	Control SD	Control Total	Weight	Mean Difference IV, Fixed, 95% CI
Gao, Y. F.2014 1d	-6.06	10.6	20	-7.08	10.8	20	16.1%	1.02 [-5.61, 7.65]
Gao, Y. F.2014 3d	-7.16	10.6	20	-8.73	10.8	20	16.1%	1.57 [-5.06, 8.20]
Gao, Y. F.2014 5d	-1.21	10.6	20	-4.43	10.8	20	16.1%	3.22 [-3.41, 9.85]
Gao, Y. F.2014after operation	-0.3	10.6	20	-1.37	10.8	20	16.1%	1.07 [-5.56, 7.70]
Shao, Y. J.2018	-5.02	10.6	44	-8.94	10.8	44	35.5%	3.92 [-0.55, 8.39]
**Total (95% CI)**			**124**			**124**	**100.0%**	**2.50 [-0.16, 5.16]**

Heterogeneity: $Chi^2 = 0.88, df = 4 (P = 0.93); I^2 = 0\%$
Test for overall effect: Z = 1.84 (P = 0.07)

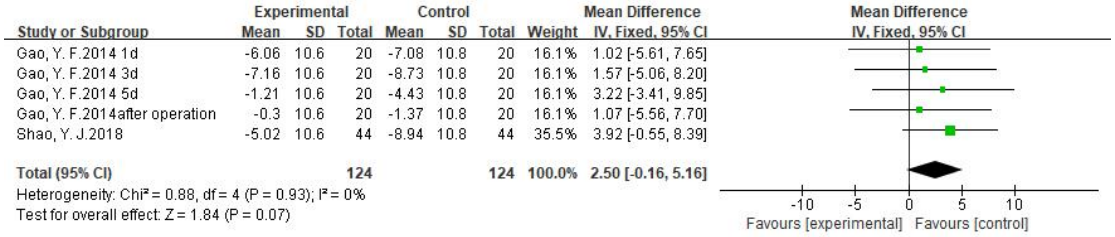

1.77 CD3(Tramadol)

Study or Subgroup	Experimental Mean	Experimental SD	Experimental Total	Control Mean	Control SD	Control Total	Weight	Mean Difference IV, Fixed, 95% CI
Bakr, M. A. E. M.2016KETOROL 90 min	-21.6	5.1	20	-19.17	4.95	20	11.6%	-2.43 [-5.54, 0.68]
Bakr, M. A. E. M.2016KETOROL1d	-11.89	5.99	20	-8.47	3.63	20	12.0%	-3.42 [-6.49, -0.35]
Bakr, M. A. E. M.2016MOR 90 min	-21.6	5.1	20	-19.37	6.24	20	9.0%	-2.23 [-5.76, 1.30]
Bakr, M. A. E. M.2016MOR1d	-11.89	5.99	20	-10.98	6.15	20	8.0%	-0.91 [-4.67, 2.85]
Wang, Z. Y.2006 MOR90min	-5.13	5.55	15	-3.44	5.24	14	7.3%	-1.69 [-5.62, 2.24]
Wang, Z. Y.2006MOR1d	-8.74	5.55	15	-10.69	5.24	14	7.3%	1.95 [-1.98, 5.88]
Wang, Z. Y.2006MOR2d	-2.48	5.55	15	-8.25	5.24	14	7.3%	5.77 [1.84, 9.70]
Wang, Z. Y.2006MOR3d	-1.95	5.55	15	-1.46	5.24	14	7.3%	-0.49 [-4.42, 3.44]
Wang, Z. Y.2006TRAM+LORN1d	-5.13	5.55	15	-5.13	5.24	15	7.6%	0.00 [-3.86, 3.86]
Wang, Z. Y.2006TRAM+LORN2d	-8.74	5.55	15	-8.74	5.24	15	7.6%	0.00 [-3.86, 3.86]
Wang, Z. Y.2006TRAM+LORN3d	-2.48	5.55	15	-2.48	5.24	15	7.6%	0.00 [-3.86, 3.86]
Wang, Z. Y.2006TRAM+LORN90min	-1.95	5.55	15	-1.95	5.24	15	7.6%	0.00 [-3.86, 3.86]
**Total (95% CI)**			**200**			**196**	**100.0%**	**-0.56 [-1.62, 0.50]**

Heterogeneity: Chi² = 17.80, df = 11 (P = 0.09); I² = 38%
Test for overall effect: Z = 1.03 (P = 0.30)

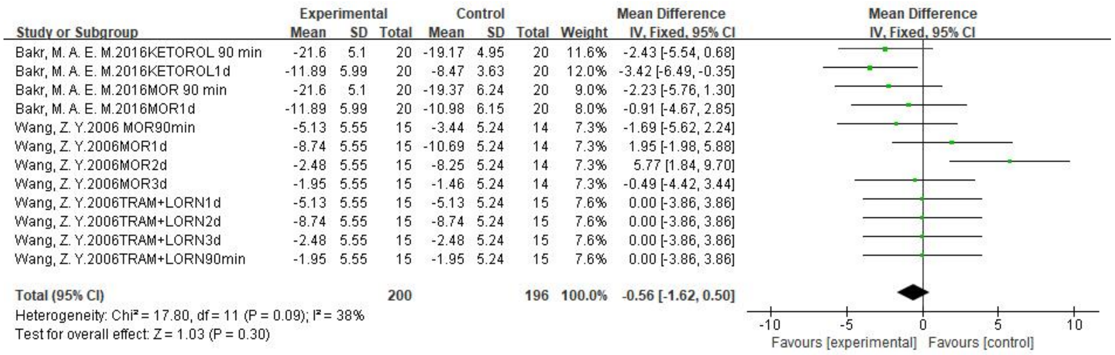

1.78 CD4(Tramadol)

Study or Subgroup	Experimental Mean	Experimental SD	Experimental Total	Control Mean	Control SD	Control Total	Weight	Mean Difference IV, Random, 95% CI
Bakr, M. A. E. M.2016KETOROL 90 min	-17.2	4.59	20	-13.54	2.67	20	8.6%	-3.66 [-5.99, -1.33]
Bakr, M. A. E. M.2016KETOROL1d	-5.1	5.62	20	-6.32	3.15	20	8.4%	1.22 [-1.60, 4.04]
Bakr, M. A. E. M.2016MOR 90 min	-17.2	4.59	20	-21.13	2.99	20	8.5%	3.93 [1.53, 6.33]
Bakr, M. A. E. M.2016MOR1d	-5.1	5.62	20	-21	3.07	20	8.4%	15.90 [13.09, 18.71]
Wang, Z. Y.2006 MOR90min	-6.49	5.11	15	-5.19	2.97	14	8.3%	-1.30 [-4.32, 1.72]
Wang, Z. Y.2006MOR1d	-5.26	5.11	15	-4.98	2.97	14	8.3%	-0.28 [-3.30, 2.74]
Wang, Z. Y.2006MOR2d	-1.09	5.11	15	3.93	2.97	14	8.3%	-5.02 [-8.04, -2.00]
Wang, Z. Y.2006MOR3d	-0.79	5.11	15	-0.29	2.97	14	8.3%	-0.50 [-3.52, 2.52]
Wang, Z. Y.2006TRAM+LORN1d	-6.49	5.11	15	-6.49	2.97	15	8.3%	0.00 [-2.99, 2.99]
Wang, Z. Y.2006TRAM+LORN2d	-5.26	5.11	15	-5.26	2.97	15	8.3%	0.00 [-2.99, 2.99]
Wang, Z. Y.2006TRAM+LORN3d	-1.09	5.11	15	-1.09	2.97	15	8.3%	0.00 [-2.99, 2.99]
Wang, Z. Y.2006TRAM+LORN90min	-0.79	5.11	15	-0.79	2.97	15	8.3%	0.00 [-2.99, 2.99]
**Total (95% CI)**			**200**			**196**	**100.0%**	**0.87 [-2.16, 3.90]**

Heterogeneity: Tau² = 26.52; Chi² = 150.27, df = 11 (P < 0.00001); I² = 93%
Test for overall effect: Z = 0.56 (P = 0.58)

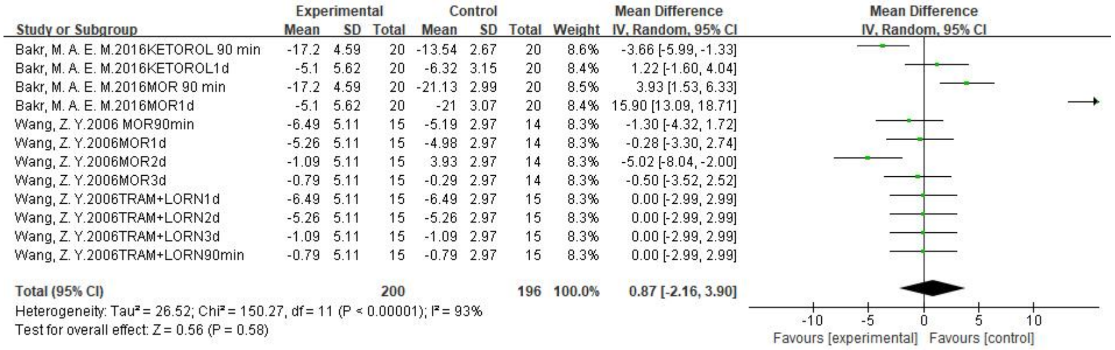

1.79 CD8(Tramadol)

Study or Subgroup	Experimental Mean	Experimental SD	Experimental Total	Control Mean	Control SD	Control Total	Weight	Mean Difference IV, Random, 95% CI
Bakr, M. A. E. M.2016KETOROL 90 min	-12.54	3.45	20	-13.13	4.86	20	8.4%	0.59 [-2.02, 3.20]
Bakr, M. A. E. M.2016KETOROL1d	-6.38	4.27	20	-4.34	3.61	20	8.7%	-2.04 [-4.49, 0.41]
Bakr, M. A. E. M.2016MOR 90 min	-12.54	3.45	20	-14.39	3.35	20	9.2%	1.85 [-0.26, 3.96]
Bakr, M. A. E. M.2016MOR1d	-6.38	4.27	20	-14.33	3.44	20	8.7%	7.95 [5.55, 10.35]
Wang, Z. Y.2006 MOR90min	1.37	3.86	15	0.21	3.815	14	8.1%	1.16 [-1.63, 3.95]
Wang, Z. Y.2006MOR1d	1.72	3.86	15	0.31	3.815	14	8.1%	1.41 [-1.38, 4.20]
Wang, Z. Y.2006MOR2d	0.77	3.86	15	0.7	3.815	14	8.1%	0.07 [-2.72, 2.86]
Wang, Z. Y.2006MOR3d	0.32	3.86	15	0.36	3.815	14	8.1%	-0.04 [-2.83, 2.75]
Wang, Z. Y.2006TRAM+LORN1d	1.37	3.86	15	1.37	3.815	15	8.2%	0.00 [-2.75, 2.75]
Wang, Z. Y.2006TRAM+LORN2d	1.72	3.86	15	1.72	3.815	15	8.2%	0.00 [-2.75, 2.75]
Wang, Z. Y.2006TRAM+LORN3d	0.77	3.86	15	0.77	3.815	15	8.2%	0.00 [-2.75, 2.75]
Wang, Z. Y.2006TRAM+LORN90min	0.32	3.86	15	0.32	3.815	15	8.2%	0.00 [-2.75, 2.75]
**Total (95% CI)**			**200**			**196**	**100.0%**	**0.95 [-0.53, 2.43]**

Heterogeneity: Tau² = 5.01; Chi² = 41.82, df = 11 (P < 0.0001); I² = 74%
Test for overall effect: Z = 1.26 (P = 0.21)

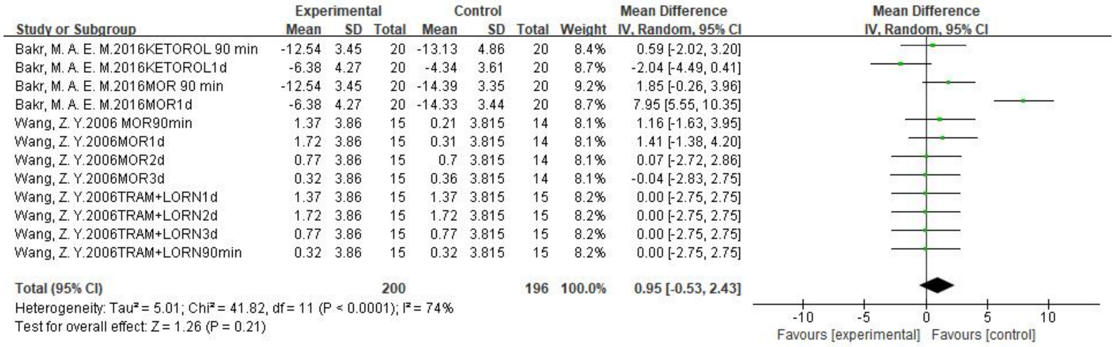

1.80 CD3(EN)

Study or Subgroup	Experimental Mean	Experimental SD	Experimental Total	Control Mean	Control SD	Control Total	Weight	Mean Difference IV, Random, 95% CI
Cai, J.2008 10d	-14.69	9.82	21	-15.69	11.98	20	7.5%	1.00 [-5.72, 7.72]
Cai, J.2008 1d	-5.51	11.39	21	-64.68	9.58	20	7.6%	59.17 [52.74, 65.60]
Li, B.2015 1d	-14.3	5.33	200	-13.8	5.37	200	9.2%	-0.50 [-1.55, 0.55]
Li, B.2015 7d	-1	6.16	200	-10	6.58	200	9.1%	9.00 [7.75, 10.25]
Li, K.2009 3d	-3.45	8.31	310	-3.1	8.81	310	9.1%	-0.35 [-1.70, 1.00]
Li, K.2009 7d	-1.02	9.16	310	-1.45	9.31	310	9.1%	0.43 [-1.02, 1.88]
Mi, L.2012 1d	-14.1	4.85	30	-12.6	5.42	30	8.9%	-1.50 [-4.10, 1.10]
Mi, L.2012 3d	-1.9	6.72	30	-6	6.1	30	8.7%	4.10 [0.85, 7.35]
Mi, L.2012 7d	-0.8	6.79	30	-4.4	6.4	30	8.7%	3.60 [0.26, 6.94]
Tang, J.2019 14d	-7.12	8.98	53	-1.45	9.33	53	8.7%	-5.67 [-9.16, -2.18]
Tang, J.2019 3m	5.62	49.41	53	-0.44	9.51	53	4.7%	6.06 [-7.49, 19.61]
Tang, J.2019 7d	-2.53	9.29	53	-0.14	9.74	53	8.6%	-2.39 [-6.01, 1.23]
**Total (95% CI)**			**1311**			**1309**	**100.0%**	**5.47 [1.27, 9.68]**

Heterogeneity: Tau² = 49.85; Chi² = 496.07, df = 11 (P < 0.00001); I² = 98%
Test for overall effect: Z = 2.55 (P = 0.01)

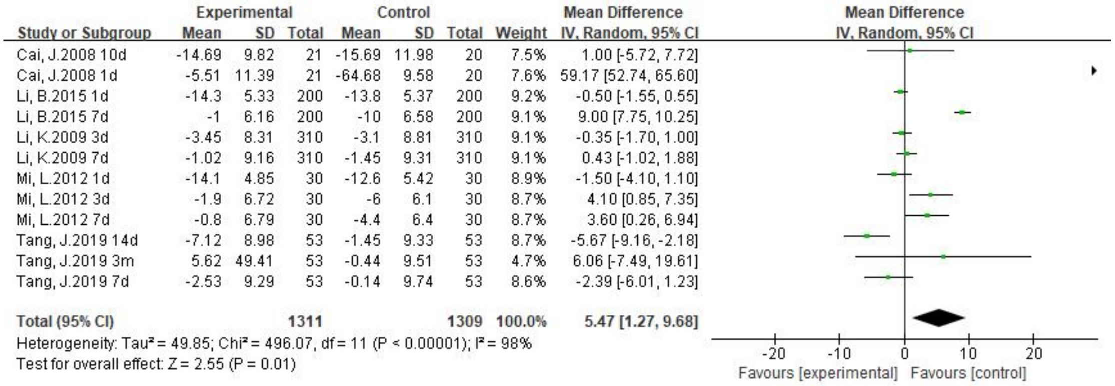

1.81 CD4(EN)

Study or Subgroup	Experimental Mean	Experimental SD	Experimental Total	Control Mean	Control SD	Control Total	Weight	Mean Difference IV, Random, 95% CI
Cai, J.2008 10d	-8.01	8.35	21	-9.02	7.37	20	6.7%	1.01 [-3.81, 5.83]
Cai, J.2008 1d	-2.32	8.9	21	-41.11	5.54	20	6.8%	38.79 [34.28, 43.30]
Li, B.2015 1d	-12	4.72	200	-13	4.28	200	7.6%	1.00 [0.12, 1.88]
Li, B.2015 7d	-1.4	5.23	200	-9.1	4.33	200	7.6%	7.70 [6.76, 8.64]
Li, K.2009 3d	-5.53	9.76	310	-6.29	9.11	310	7.6%	0.76 [-0.73, 2.25]
Li, K.2009 7d	-0.64	10.61	310	-0.6	8.98	310	7.6%	-0.04 [-1.59, 1.51]
Mi, L.2012 1d	-5.8	4.68	30	-6.8	4.62	30	7.4%	1.00 [-1.35, 3.35]
Mi, L.2012 3d	-3.5	4.61	30	-11.6	4.5	30	7.4%	8.10 [5.79, 10.41]
Mi, L.2012 7d	-1.6	5.24	30	-7.7	4.75	30	7.4%	6.10 [3.57, 8.63]
Tang, J.2019 14d	-6.27	6.61	53	-0.31	7.07	53	7.4%	-5.96 [-8.57, -3.35]
Tang, J.2019 3m	4.25	32.06	53	-0.58	7.88	53	5.2%	4.83 [-4.06, 13.72]
Tang, J.2019 7d	-2.8	6.94	53	9.56	7.46	53	7.3%	-12.36 [-15.10, -9.62]
Zong, L.2019 1d	0.56	10.6	48	-10.81	9.37	48	7.0%	11.37 [7.37, 15.37]
Zong, L.2019 7d	0.57	10.62	48	-9.09	11.1	48	6.9%	9.66 [5.31, 14.01]
**Total (95% CI)**			**1407**			**1405**	**100.0%**	**4.93 [1.35, 8.51]**

Heterogeneity: Tau² = 43.54; Chi² = 599.83, df = 13 (P < 0.00001); I² = 98%
Test for overall effect: Z = 2.70 (P = 0.007)

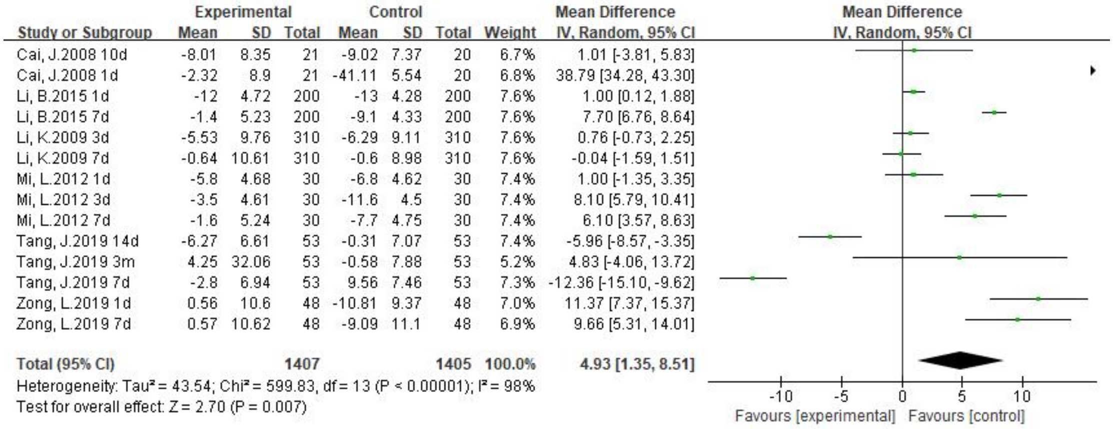

1.82 CD8(EN)

Study or Subgroup	Experimental Mean	Experimental SD	Experimental Total	Control Mean	Control SD	Control Total	Weight	Mean Difference IV, Random, 95% CI
Li, B.2015 1d	-0.4	3.61	200	-2.3	3.96	200	16.8%	1.90 [1.16, 2.64]
Li, B.2015 7d	-0.2	2.79	200	-0.6	3.82	200	16.8%	0.40 [-0.26, 1.06]
Mi, L.2012 1d	0.8	3.76	30	-4.3	4	30	14.9%	5.10 [3.14, 7.06]
Mi, L.2012 3d	-4.4	3.47	30	0	3.26	30	15.4%	-4.40 [-6.10, -2.70]
Mi, L.2012 7d	-6.8	3.84	30	-2.3	3.34	30	15.2%	-4.50 [-6.32, -2.68]
Zong, L.2019 1d	-0.77	10.68	48	-3.62	9.77	48	10.5%	2.85 [-1.24, 6.94]
Zong, L.2019 7d	-0.65	10.73	48	-2.6	10.18	48	10.3%	1.95 [-2.23, 6.13]
**Total (95% CI)**			**586**			**586**	**100.0%**	**0.28 [-1.85, 2.42]**

Heterogeneity: Tau² = 6.93; Chi² = 97.06, df = 6 (P < 0.00001); I² = 94%
Test for overall effect: Z = 0.26 (P = 0.79)

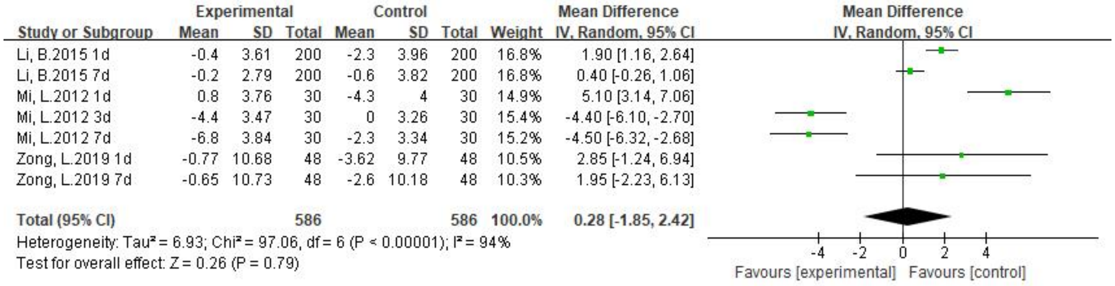

1.83 CD4/CD8(EN)

Study or Subgroup	Experimental Mean	Experimental SD	Experimental Total	Control Mean	Control SD	Control Total	Weight	Mean Difference IV, Random, 95% CI
Cai, J.2008 10d	-0.3	0.52	21	-0.26	0.54	20	5.9%	-0.04 [-0.36, 0.28]
Cai, J.2008 1d	-0.15	0.66	21	-0.24	0.53	20	5.4%	0.09 [-0.28, 0.46]
Li, B.2015 1d	0	0.72	200	-0.4	0.42	200	8.5%	0.40 [0.28, 0.52]
Li, B.2015 7d	0.1	0.5	200	-0.4	0.85	200	8.2%	0.50 [0.36, 0.64]
Li, K.2009 3d	-0.24	0.34	310	-0.3	0.4	310	8.9%	0.06 [0.00, 0.12]
Li, K.2009 7d	-0.08	0.52	310	-0.14	0.44	310	8.8%	0.06 [-0.02, 0.14]
Mi, L.2012 1d	-0.36	0.37	30	-0.13	0.5	30	7.2%	-0.23 [-0.45, -0.01]
Mi, L.2012 3d	-0.07	0.28	30	-0.31	0.28	30	8.2%	0.24 [0.10, 0.38]
Mi, L.2012 7d	0	0.35	30	-0.29	0.32	30	7.9%	0.29 [0.12, 0.46]
Tang, J.2019 14d	-0.48	0.38	53	-0.12	0.45	53	8.0%	-0.36 [-0.52, -0.20]
Tang, J.2019 3m	0.45	1.16	53	-0.05	0.4	53	5.8%	0.50 [0.17, 0.83]
Tang, J.2019 7d	-0.34	0.42	53	-0.03	0.5	53	7.8%	-0.31 [-0.49, -0.13]
Zong, L.2019 1d	-0.08	1.04	48	-0.37	1	48	4.9%	0.29 [-0.12, 0.70]
Zong, L.2019 7d	-0.06	1.08	48	-0.21	1.05	48	4.7%	0.15 [-0.28, 0.58]
**Total (95% CI)**			**1407**			**1405**	**100.0%**	**0.11 [-0.02, 0.24]**

Heterogeneity: Tau² = 0.05; Chi² = 139.06, df = 13 (P < 0.00001); I² = 91%
Test for overall effect: Z = 1.64 (P = 0.10)

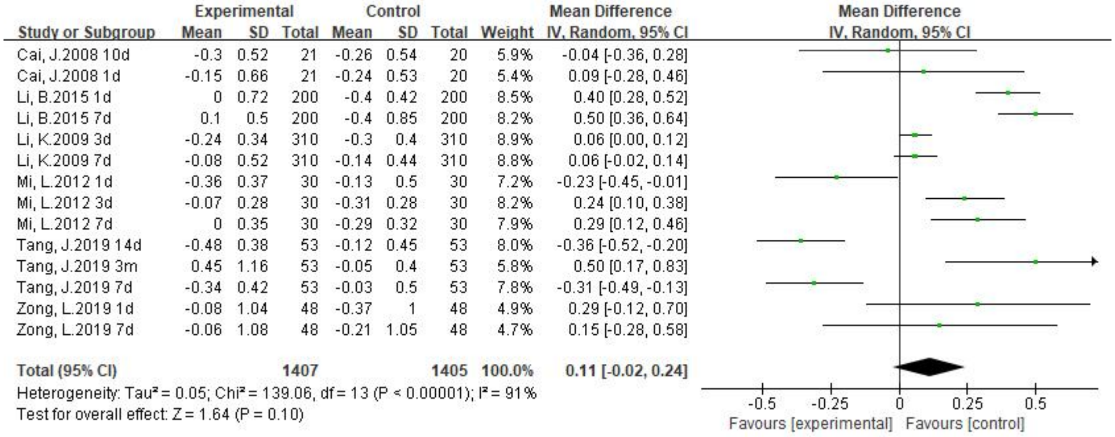

1.84 NK(EN)

Study or Subgroup	Experimental Mean	Experimental SD	Experimental Total	Control Mean	Control SD	Control Total	Weight	Mean Difference IV, Random, 95% CI
Mi, L.2012 1d	-1.9	4.88	30	-1.1	7.17	30	34.1%	-0.80 [-3.90, 2.30]
Mi, L.2012 3d	4	5.74	30	-1.9	7.09	30	33.4%	5.90 [2.64, 9.16]
Mi, L.2012 7d	4.9	6.41	30	0.3	7.25	30	32.5%	4.60 [1.14, 8.06]
**Total (95% CI)**			**90**			**90**	**100.0%**	**3.19 [-0.95, 7.34]**

Heterogeneity: Tau² = 10.61; Chi² = 9.61, df = 2 (P = 0.008); I² = 79%
Test for overall effect: Z = 1.51 (P = 0.13)

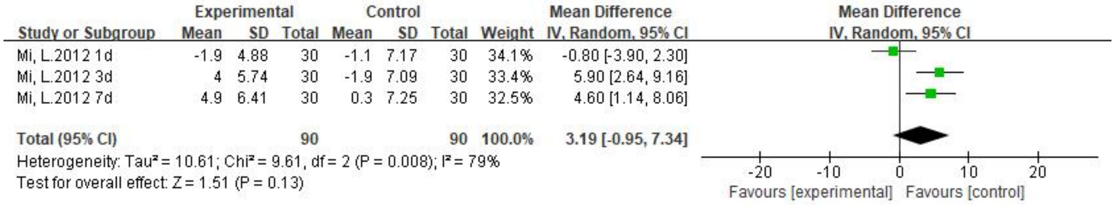

1.85 CD3(EEN)

Study or Subgroup	Experimental Mean	Experimental SD	Experimental Total	Control Mean	Control SD	Control Total	Weight	Std. Mean Difference IV, Random, 95% CI
Chen, H.2006	180.87	756.38	22	-209.34	747.01	24	31.6%	0.51 [-0.08, 1.10]
Li, B.2015 1d	-14.3	5.33	200	-13.8	5.37	200	34.3%	-0.09 [-0.29, 0.10]
Li, B.2015 7d	-1	6.16	200	-10	6.58	200	34.2%	1.41 [1.19, 1.63]
**Total (95% CI)**			**422**			**424**	**100.0%**	**0.61 [-0.51, 1.73]**

Heterogeneity: Tau² = 0.94; Chi² = 100.32, df = 2 (P < 0.00001); I² = 98%
Test for overall effect: Z = 1.07 (P = 0.28)

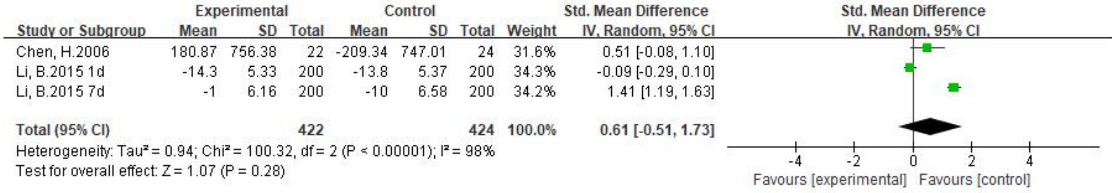

1.86 CD4(EEN)

Study or Subgroup	Experimental Mean	Experimental SD	Experimental Total	Control Mean	Control SD	Control Total	Weight	Std. Mean Difference IV, Random, 95% CI
Chen, H.2006	139.13	447.99	22	-66.08	404.97	24	18.6%	0.47 [-0.11, 1.06]
Li, B.2015 1d	-12	4.72	200	-13	4.28	200	20.7%	0.22 [0.02, 0.42]
Li, B.2015 7d	-1.4	5.23	200	-9.1	4.33	200	20.6%	1.60 [1.38, 1.83]
Wang, H. X.2011 10d	-7.43	10.37	63	-5.6	10.02	63	20.1%	-0.18 [-0.53, 0.17]
Wang, H. X.2011 1d	-3.13	10.2	63	-1.23	6.26	63	20.1%	-0.22 [-0.57, 0.13]
**Total (95% CI)**			**548**			**550**	**100.0%**	**0.38 [-0.37, 1.14]**

Heterogeneity: Tau² = 0.71; Chi² = 129.56, df = 4 (P < 0.00001); I² = 97%
Test for overall effect: Z = 0.99 (P = 0.32)

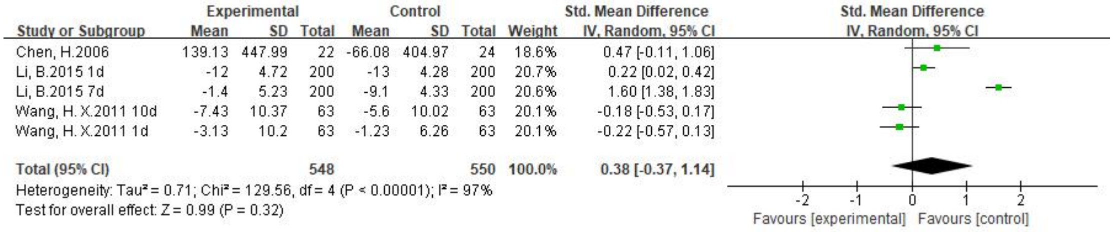

1.87 CD8 (EEN)

Study or Subgroup	Experimental Mean	Experimental SD	Experimental Total	Control Mean	Control SD	Control Total	Weight	Std. Mean Difference IV, Random, 95% CI
Chen, H.2006	-42.59	309.8	22	-123.04	346.71	24	10.2%	0.24 [-0.34, 0.82]
Li, B.2015 1d	-0.4	3.61	200	-2.3	3.96	200	26.5%	0.50 [0.30, 0.70]
Li, B.2015 7d	-0.2	2.79	200	-0.6	3.82	200	26.7%	0.12 [-0.08, 0.32]
Wang, H. X.2011 10d	-0.32	12.17	63	-2.14	12.81	63	18.3%	0.14 [-0.20, 0.49]
Wang, H. X.2011 1d	-0.55	11.19	63	0.29	13.47	63	18.3%	-0.07 [-0.42, 0.28]
**Total (95% CI)**			**548**			**550**	**100.0%**	**0.20 [-0.02, 0.42]**

Heterogeneity: Tau² = 0.04; Chi² = 11.29, df = 4 (P = 0.02); I² = 65%
Test for overall effect: Z = 1.79 (P = 0.07)

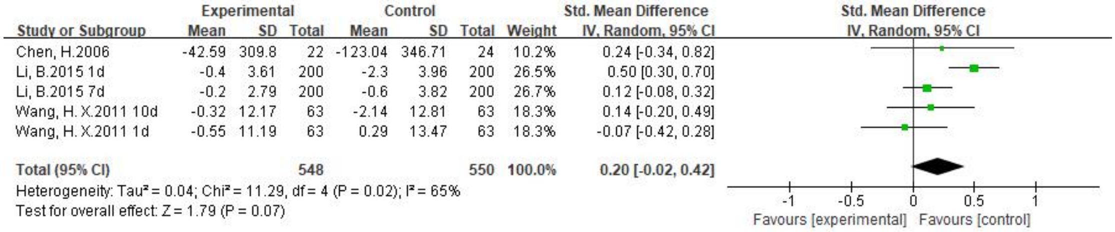

1.88 CD4/CD8(EEN)

Study or Subgroup	Experimental Mean	Experimental SD	Experimental Total	Control Mean	Control SD	Control Total	Weight	Mean Difference IV, Random, 95% CI
Chen, H.2006	0.58	1.27	22	0.29	0.87	24	10.9%	0.29 [-0.34, 0.92]
Li, B.2015 1d	0	0.72	200	-0.4	0.42	200	24.8%	0.40 [0.28, 0.52]
Li, B.2015 7d	0.1	0.5	200	-0.4	0.85	200	24.4%	0.50 [0.36, 0.64]
Wang, H. X.2011 10d	-0.06	0.73	63	-0.02	1.17	63	18.6%	-0.04 [-0.38, 0.30]
Wang, H. X.2011 1d	0.22	0.71	63	0.49	0.76	63	21.2%	-0.27 [-0.53, -0.01]
**Total (95% CI)**			**548**			**550**	**100.0%**	**0.19 [-0.09, 0.46]**

Heterogeneity: Tau² = 0.08; Chi² = 32.78, df = 4 (P < 0.00001); I² = 88%
Test for overall effect: Z = 1.34 (P = 0.18)

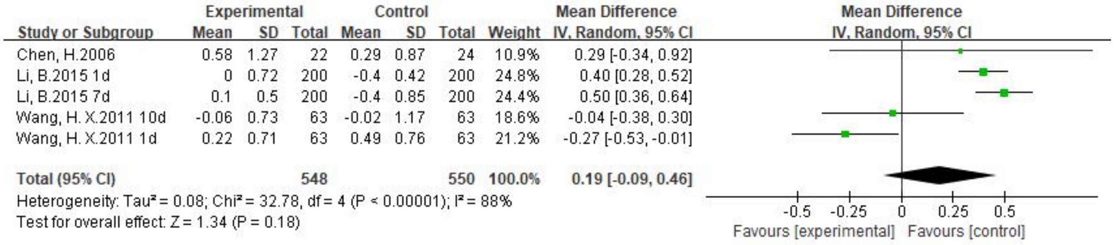

1.89 CD3(EN+PN)

Study or Subgroup	Experimental Mean	Experimental SD	Experimental Total	Control Mean	Control SD	Control Total	Weight	Std. Mean Difference IV, Random, 95% CI
Chen, H.2006	180.87	756.38	22	-209.34	747.01	24	11.4%	0.51 [-0.08, 1.10]
Ma, B. Q.2020 1d	-11.26	9.53	26	-7.86	22.56	30	12.8%	-0.19 [-0.72, 0.34]
Ma, B. Q.2020 3d	-6.23	9.53	26	-15.53	22.56	30	12.6%	0.52 [-0.02, 1.05]
Ma, B. Q.2020 7d	5.38	9.53	26	-4.3	22.56	30	12.6%	0.54 [0.00, 1.07]
Tang, J.2019 14d	-1.45	9.33	53	-7.12	8.98	53	16.7%	0.61 [0.22, 1.00]
Tang, J.2019 3m	-0.44	9.51	53	5.62	49.41	53	17.0%	-0.17 [-0.55, 0.21]
Tang, J.2019 7d	-0.14	9.74	53	-2.53	9.29	53	16.9%	0.25 [-0.13, 0.63]
**Total (95% CI)**			**259**			**273**	**100.0%**	**0.28 [0.02, 0.55]**

Heterogeneity: Tau² = 0.07; Chi² = 13.44, df = 6 (P = 0.04); I² = 55%
Test for overall effect: Z = 2.11 (P = 0.04)

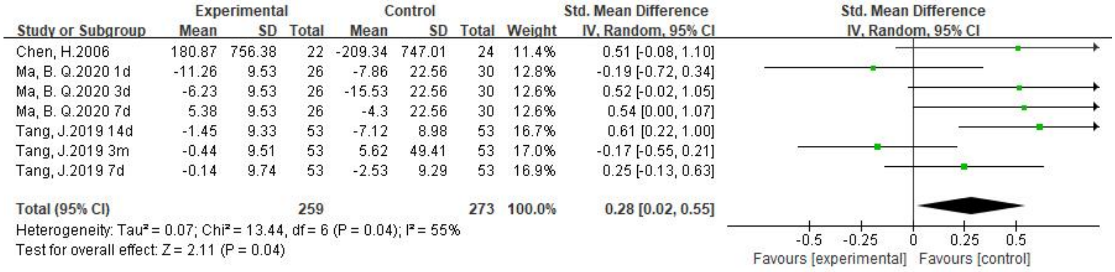

1.90 CD4(EN+PN)

Study or Subgroup	Experimental Mean	Experimental SD	Experimental Total	Control Mean	Control SD	Control Total	Weight	Std. Mean Difference IV, Random, 95% CI
Chen, H.2006	139.13	447.99	22	-66.08	404.97	24	13.5%	0.47 [-0.11, 1.06]
Ma, B. Q.2020 1d	-14.16	7.47	26	-15.34	15.2	30	14.0%	0.10 [-0.43, 0.62]
Ma, B. Q.2020 3d	-5.31	7.47	26	-12.1	15.2	30	13.9%	0.55 [0.01, 1.08]
Ma, B. Q.2020 7d	8.85	7.47	26	-2.69	15.2	30	13.8%	0.93 [0.37, 1.48]
Tang, J.2019 14d	-0.31	7.07	53	-6.27	6.61	53	15.0%	0.86 [0.47, 1.26]
Tang, J.2019 3m	-0.58	7.88	53	4.25	32.06	53	15.1%	-0.21 [-0.59, 0.18]
Tang, J.2019 7d	9.56	7.46	53	-2.8	6.94	53	14.6%	1.70 [1.26, 2.15]
**Total (95% CI)**			**259**			**273**	**100.0%**	**0.63 [0.12, 1.14]**

Heterogeneity: Tau² = 0.41; Chi² = 47.32, df = 6 (P < 0.00001); I² = 87%
Test for overall effect: Z = 2.43 (P = 0.02)

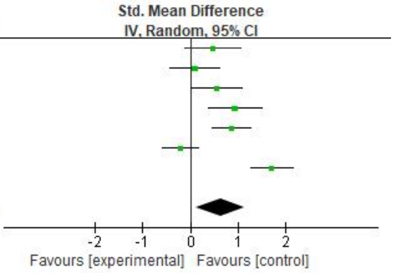

1.91 CD8(EN+PN)

Study or Subgroup	Experimental Mean	Experimental SD	Experimental Total	Control Mean	Control SD	Control Total	Weight	Std. Mean Difference IV, Fixed, 95% CI
Chen, H.2006	-42.59	309.8	22	-123.04	346.71	24	21.4%	0.24 [-0.34, 0.82]
Ma, B. Q.2020 1d	-0.21	309.8	26	-2.41	346.71	30	26.2%	0.01 [-0.52, 0.53]
Ma, B. Q.2020 3d	2.59	309.8	26	-7.79	346.71	30	26.2%	0.03 [-0.49, 0.56]
Ma, B. Q.2020 7d	5.18	309.8	26	-4.02	346.71	30	26.2%	0.03 [-0.50, 0.55]
**Total (95% CI)**			**100**			**114**	**100.0%**	**0.07 [-0.20, 0.34]**

Heterogeneity: Chi² = 0.43, df = 3 (P = 0.93); I² = 0%
Test for overall effect: Z = 0.50 (P = 0.62)

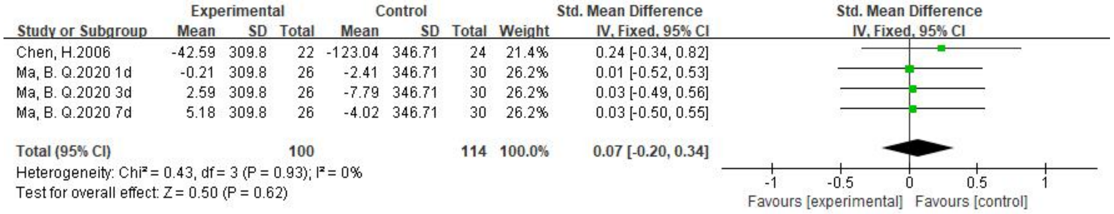

1.92 CD4/CD8(EN+PN)

Study or Subgroup	Experimental Mean	Experimental SD	Experimental Total	Control Mean	Control SD	Control Total	Weight	Mean Difference IV, Random, 95% CI
Chen, H.2006	0.58	1.27	22	0.29	0.87	24	8.3%	0.29 [-0.34, 0.92]
Ma, B. Q.2020 1d	-0.68	0.45	26	-0.38	0.65	30	14.7%	-0.30 [-0.59, -0.01]
Ma, B. Q.2020 3d	0.05	0.45	26	-0.22	0.65	30	14.7%	0.27 [-0.02, 0.56]
Ma, B. Q.2020 7d	0.44	0.45	26	-0.01	0.65	30	14.7%	0.45 [0.16, 0.74]
Tang, J.2019 14d	-0.12	0.45	53	-0.48	0.38	53	17.1%	0.36 [0.20, 0.52]
Tang, J.2019 3m	-0.05	0.4	53	0.45	1.16	53	13.8%	-0.50 [-0.83, -0.17]
Tang, J.2019 7d	-0.03	0.5	53	-0.34	0.42	53	16.8%	0.31 [0.13, 0.49]
**Total (95% CI)**			**259**			**273**	**100.0%**	**0.13 [-0.12, 0.38]**

Heterogeneity: Tau² = 0.09; Chi² = 37.17, df = 6 (P < 0.00001); I² = 84%
Test for overall effect: Z = 1.03 (P = 0.30)

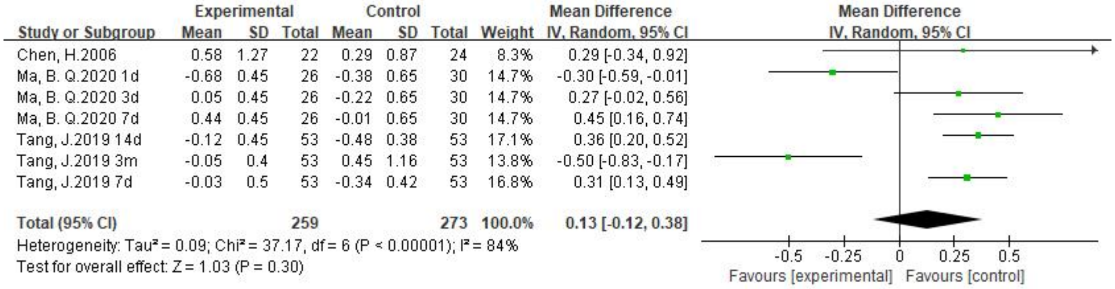

1.93 CD3(Immune+PN)

Study or Subgroup	Experimental Mean	Experimental SD	Experimental Total	Control Mean	Control SD	Control Total	Weight	Mean Difference IV, Random, 95% CI
Liang, B.2008 1d	-9.38	15.77	20	2.32	19.53	21	11.9%	-11.70 [-22.54, -0.86]
Liang, B.2008 8d	3.47	13.39	20	6.16	15.12	21	15.3%	-2.69 [-11.42, 6.04]
Yang,J2022 1d	-6.26	14.43	60	-6.39	13.28	60	23.7%	0.13 [-4.83, 5.09]
Yang,J2022 3d	-0.7	13.41	60	-6.31	12.97	60	24.3%	5.61 [0.89, 10.33]
Yang,J2022 5d	2.05	12.74	60	-3.24	12.65	60	24.8%	5.29 [0.75, 9.83]
**Total (95% CI)**			**220**			**222**	**100.0%**	**0.90 [-3.80, 5.61]**

Heterogeneity: Tau² = 17.91; Chi² = 11.94, df = 4 (P = 0.02); I² = 66%
Test for overall effect: Z = 0.38 (P = 0.71)

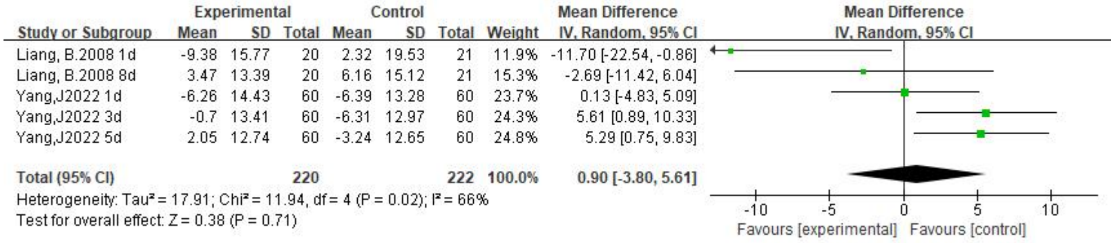

1.94 CD4(Immune+PN)

Study or Subgroup	Experimental Mean	Experimental SD	Experimental Total	Control Mean	Control SD	Control Total	Weight	Mean Difference IV, Random, 95% CI
Liang, B.2008 1d	-12.01	15.69	20	-6.05	18.62	21	10.5%	-5.96 [-16.48, 4.56]
Liang, B.2008 8d	5.79	14.91	20	3.61	14.08	21	13.0%	2.18 [-6.71, 11.07]
Yang,J2022 1d	-7.69	9.76	60	-7.59	9.82	60	25.7%	-0.10 [-3.60, 3.40]
Yang,J2022 3d	-0.2	10.83	60	-7.81	9.82	60	25.2%	7.61 [3.91, 11.31]
Yang,J2022 5d	2.84	10.19	60	-3.52	9.48	60	25.7%	6.36 [2.84, 9.88]
**Total (95% CI)**			**220**			**222**	**100.0%**	**3.18 [-1.00, 7.36]**

Heterogeneity: Tau² = 14.50; Chi² = 14.25, df = 4 (P = 0.007); I² = 72%
Test for overall effect: Z = 1.49 (P = 0.14)

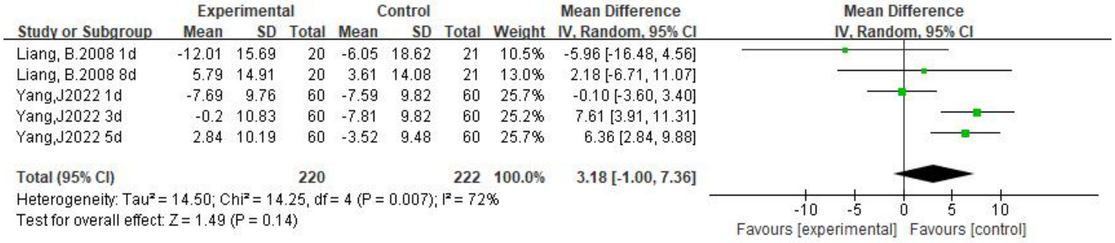

1.95 CD8(Immune+PN)

Study or Subgroup	Experimental Mean	Experimental SD	Experimental Total	Control Mean	Control SD	Control Total	Weight	Mean Difference IV, Fixed, 95% CI
Liang, B.2008 1d	2.98	11.12	20	0.38	16.3	21	7.8%	2.60 [-5.91, 11.11]
Liang, B.2008 8d	-0.43	10.83	20	0.19	14.73	21	9.1%	-0.62 [-8.51, 7.27]
Yang,J2022 1d	1.07	13.38	60	2.2	12.99	60	25.4%	-1.13 [-5.85, 3.59]
Yang,J2022 3d	0.16	13.34	60	0.45	11.94	60	27.5%	-0.29 [-4.82, 4.24]
Yang,J2022 5d	-0.84	12.69	60	-1.49	11.42	60	30.3%	0.65 [-3.67, 4.97]
**Total (95% CI)**			**220**			**222**	**100.0%**	**-0.02 [-2.40, 2.35]**

Heterogeneity: Chi² = 0.71, df = 4 (P = 0.95); I² = 0%
Test for overall effect: Z = 0.02 (P = 0.98)

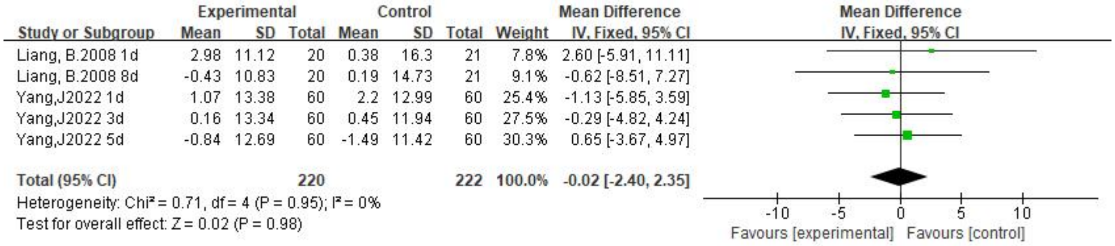

1.96 CD4/CD8(Immune+PN)

Study or Subgroup	Experimental Mean	Experimental SD	Experimental Total	Control Mean	Control SD	Control Total	Weight	Mean Difference IV, Random, 95% CI
Liang, B.2008 1d	-0.65	0.95	20	0.25	1.51	21	12.3%	-0.90 [-1.67, -0.13]
Liang, B.2008 8d	0.26	1.08	20	0.05	1.43	21	12.2%	0.21 [-0.56, 0.98]
Yang,J2022 1d	-0.45	1.07	60	-0.42	1	60	25.7%	-0.03 [-0.40, 0.34]
Yang,J2022 3d	-0.03	1.22	60	-0.38	0.96	60	24.7%	0.35 [-0.04, 0.74]
Yang,J2022 5d	0.06	1.13	60	-0.15	1	60	25.2%	0.21 [-0.17, 0.59]
**Total (95% CI)**			**220**			**222**	**100.0%**	**0.05 [-0.28, 0.37]**

Heterogeneity: Tau² = 0.07; Chi² = 8.93, df = 4 (P = 0.06); I² = 55%
Test for overall effect: Z = 0.28 (P = 0.78)

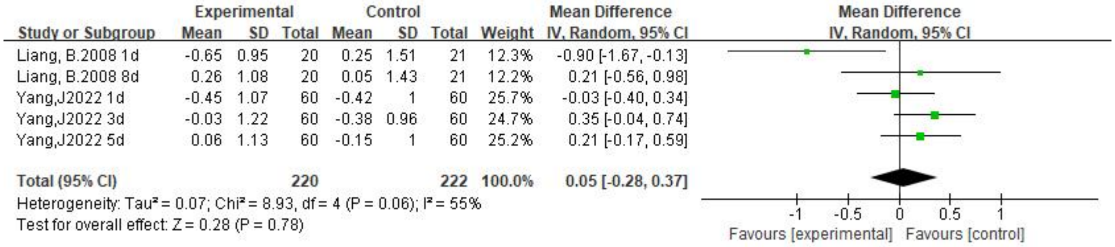

1.97 CD4(EN before)

Study or Subgroup	Experimental Mean	Experimental SD	Experimental Total	Control Mean	Control SD	Control Total	Weight	Mean Difference IV, Random, 95% CI
Wang, H. X.2011 10d	-5.6	10.02	63	-7.43	10.37	63	25.3%	1.83 [-1.73, 5.39]
Wang, H. X.2011 1d	-1.23	6.26	63	-3.13	10.2	63	26.4%	1.90 [-1.06, 4.86]
Zong, L.2019 1d	0.56	10.6	48	-10.81	9.37	48	24.5%	11.37 [7.37, 15.37]
Zong, L.2019 7d	0.57	10.62	48	-9.09	11.1	48	23.8%	9.66 [5.31, 14.01]
**Total (95% CI)**			**222**			**222**	**100.0%**	**6.04 [1.15, 10.94]**

Heterogeneity: $Tau^2 = 21.34$; $Chi^2 = 21.40$, df = 3 (P < 0.0001); $I^2 = 86\%$
Test for overall effect: Z = 2.42 (P = 0.02)

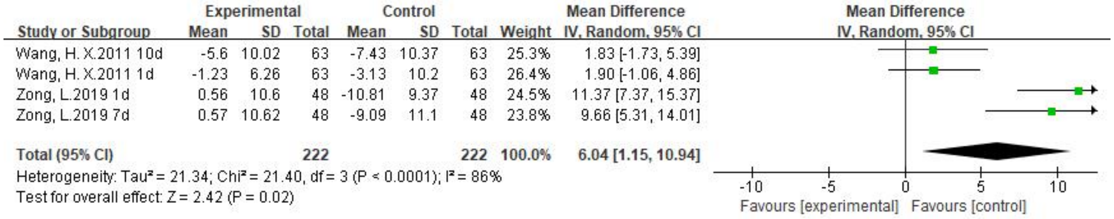

1.98 CD8(EN before)

Study or Subgroup	Experimental Mean	Experimental SD	Experimental Total	Control Mean	Control SD	Control Total	Weight	Mean Difference IV, Fixed, 95% CI
Wang, H. X.2011 10d	-2.14	12.81	63	-0.32	12.17	63	23.6%	-1.82 [-6.18, 2.54]
Wang, H. X.2011 1d	0.29	13.47	63	-0.55	11.19	63	24.0%	0.84 [-3.48, 5.16]
Zong, L.2019 1d	-0.77	10.68	48	-3.62	9.77	48	26.8%	2.85 [-1.24, 6.94]
Zong, L.2019 7d	-0.65	10.73	48	-2.6	10.18	48	25.6%	1.95 [-2.23, 6.13]
**Total (95% CI)**			**222**			**222**	**100.0%**	**1.04 [-1.08, 3.15]**

Heterogeneity: $Chi^2 = 2.59$, df = 3 (P = 0.46); $I^2 = 0\%$
Test for overall effect: Z = 0.96 (P = 0.34)

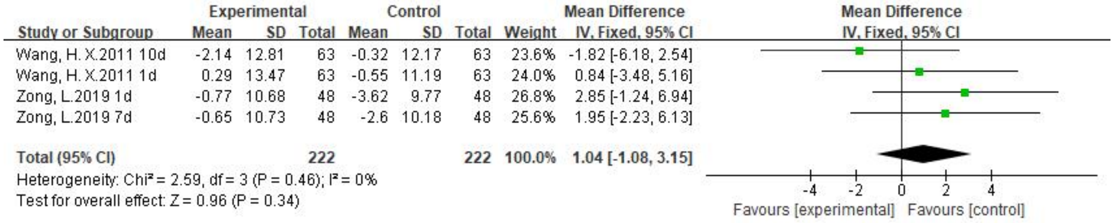

1.99 CD4/CD8(EN before)

Study or Subgroup	Experimental Mean	Experimental SD	Experimental Total	Control Mean	Control SD	Control Total	Weight	Mean Difference IV, Fixed, 95% CI
Wang, H. X.2011 10d	-0.02	1.17	63	-0.06	0.73	63	24.4%	0.04 [-0.30, 0.38]
Wang, H. X.2011 1d	0.49	0.76	63	0.22	0.71	63	43.0%	0.27 [0.01, 0.53]
Zong, L.2019 1d	-0.08	1.04	48	-0.37	1	48	17.0%	0.29 [-0.12, 0.70]
Zong, L.2019 7d	-0.06	1.08	48	-0.21	1.05	48	15.6%	0.15 [-0.28, 0.58]
**Total (95% CI)**			**222**			**222**	**100.0%**	**0.20 [0.03, 0.37]**

Heterogeneity: $Chi^2 = 1.37$, df = 3 (P = 0.71); $I^2 = 0\%$
Test for overall effect: Z = 2.31 (P = 0.02)

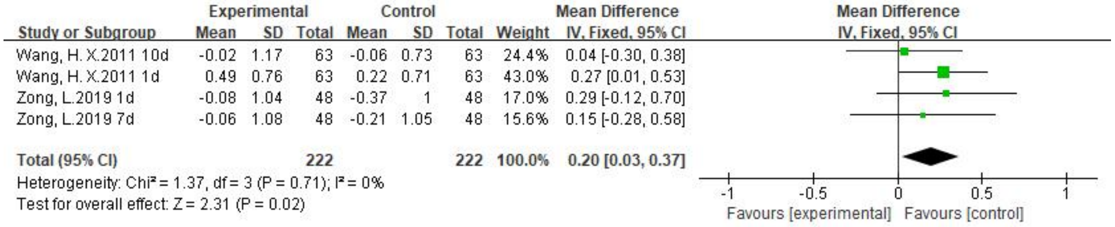

Supplement: Supplementary file 1 [file DataSheet1.pdf]
