# Supplementary material for: What are the beneficial treatment strategies in maintaining T lymphocyte subsets after cancer surgery? A systematic review and network meta-analysis
Source: Front Immunol. 2026 Jul 14;17:1854279. doi: 10.3389/fimmu.2026.1854279 (PMC13408238; doi:10.3389/fimmu.2026.1854279)
Supplement: Supplementary file 2 [file DataSheet2.pdf]

Figure S2 Funnel plots of available comparisons among all included interventions.

2.1 CD3(Hysteroscopy)

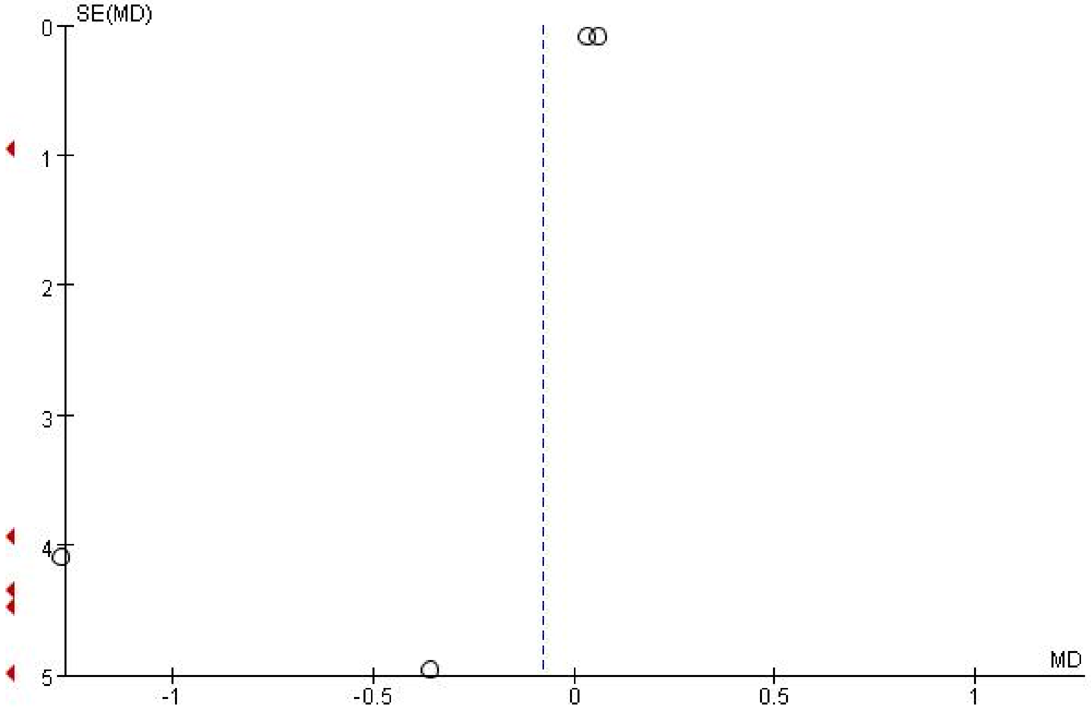

2.2 CD4(Hysteroscopy)

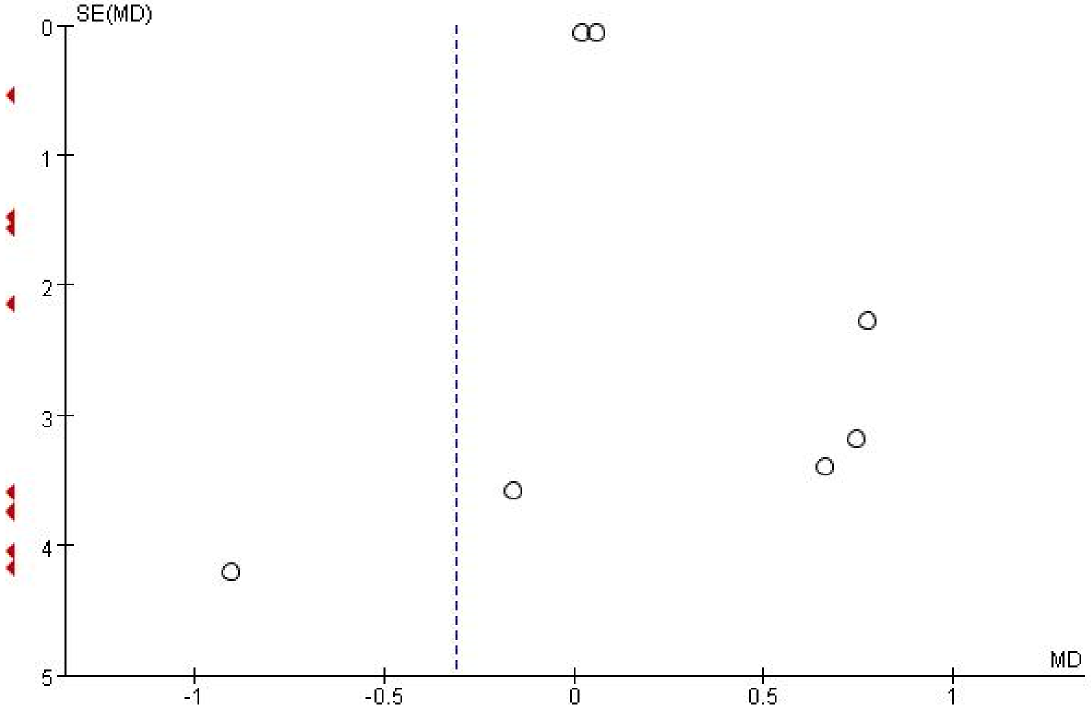

2.3 CD8(Hysteroscopy)

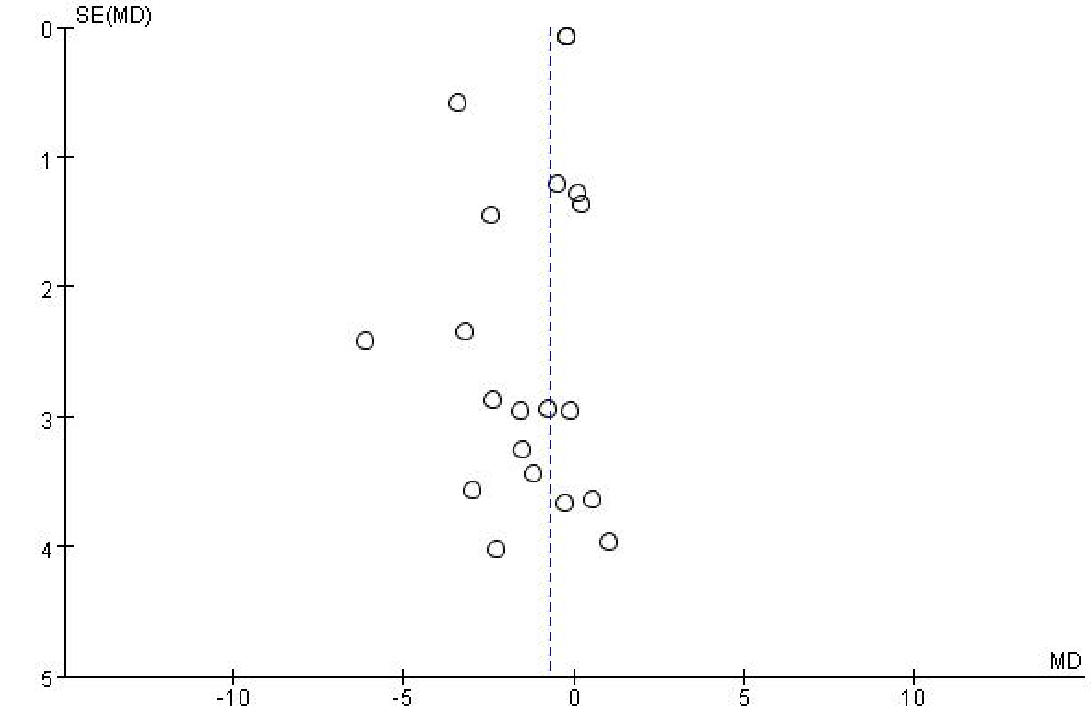

2.4 CD4/CD8(Hysteroscopy)

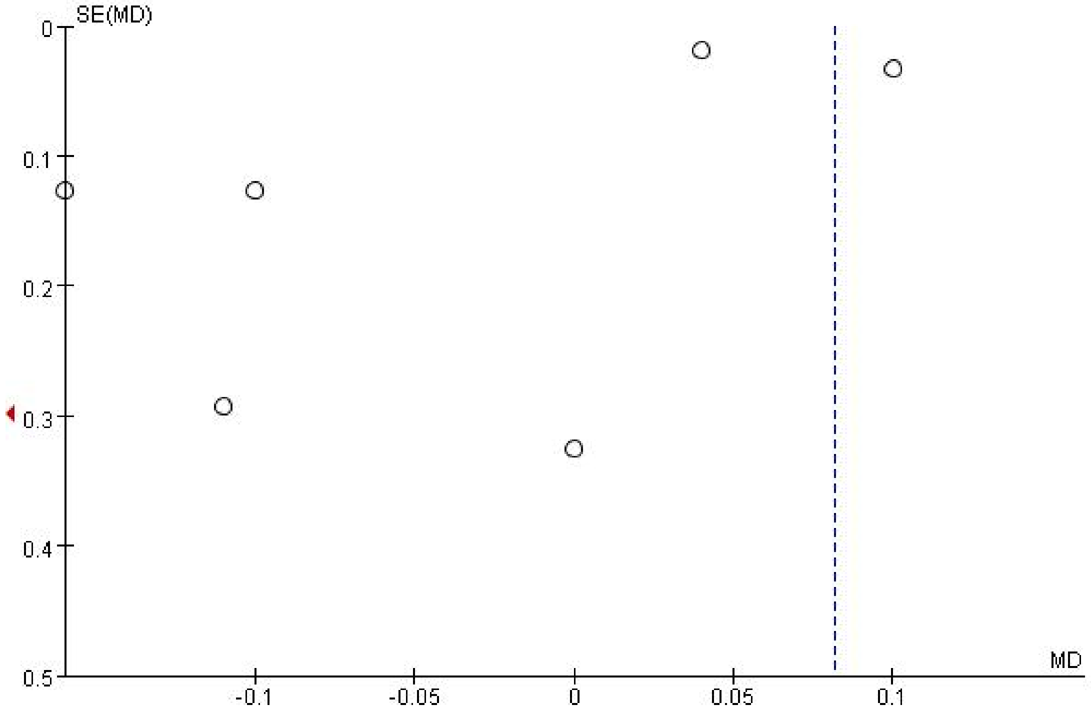

2.5 NK(Hysteroscopy)

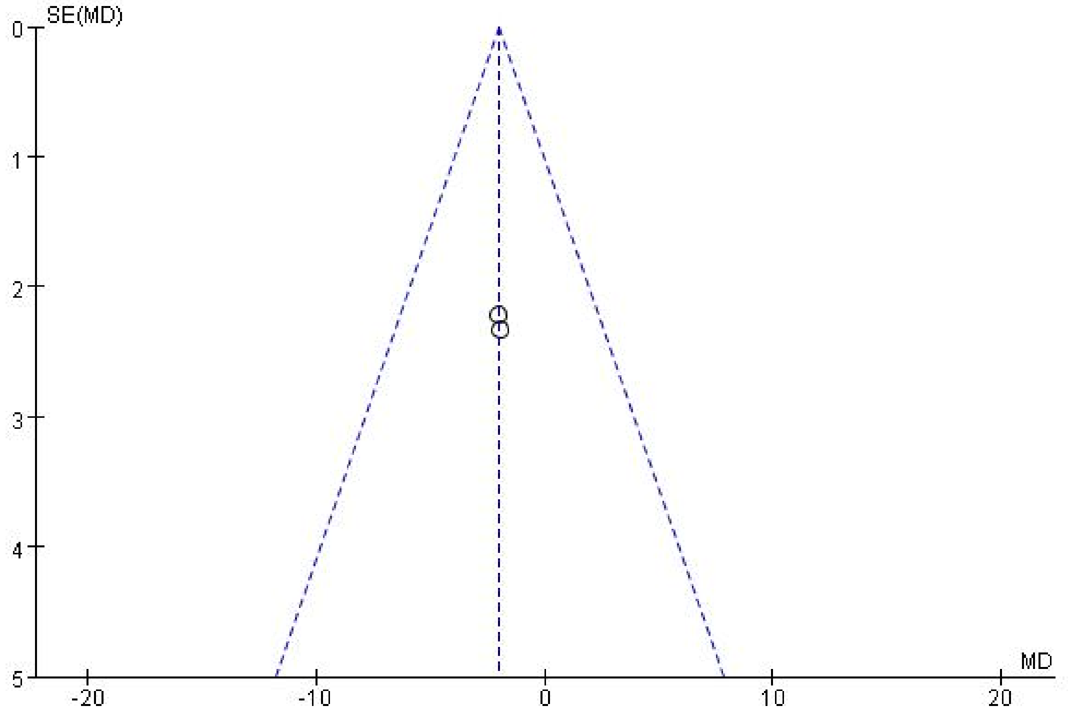

2.6CD3(Immune)

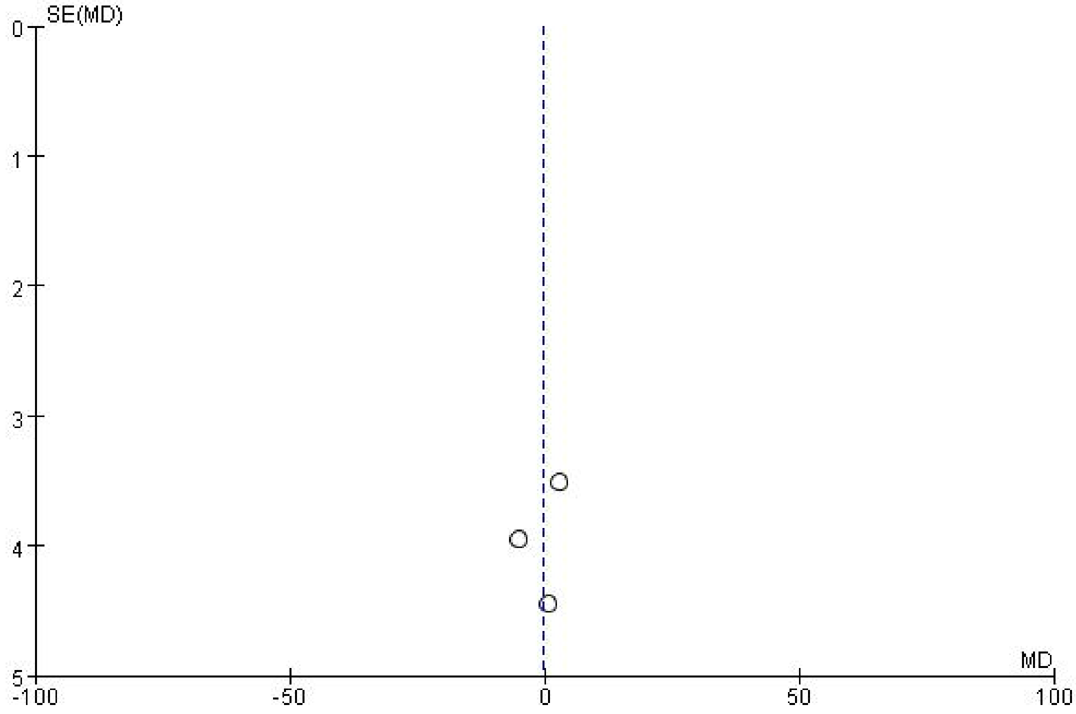

2.7 CD4(Immune)

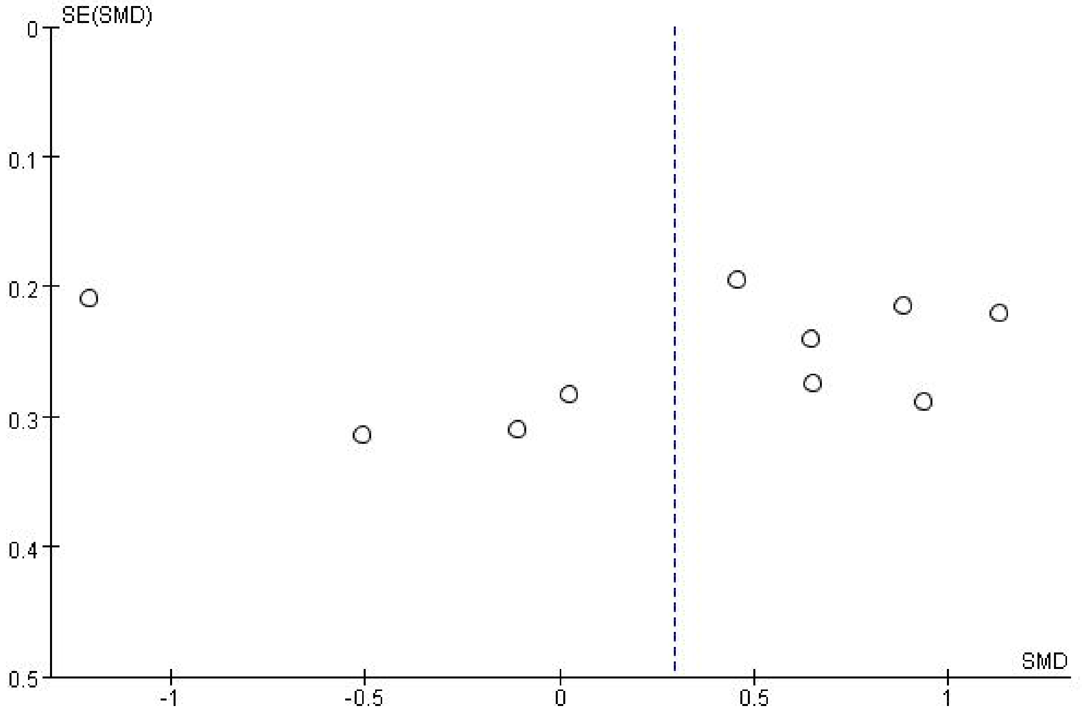

2.8 CD8(Immune)

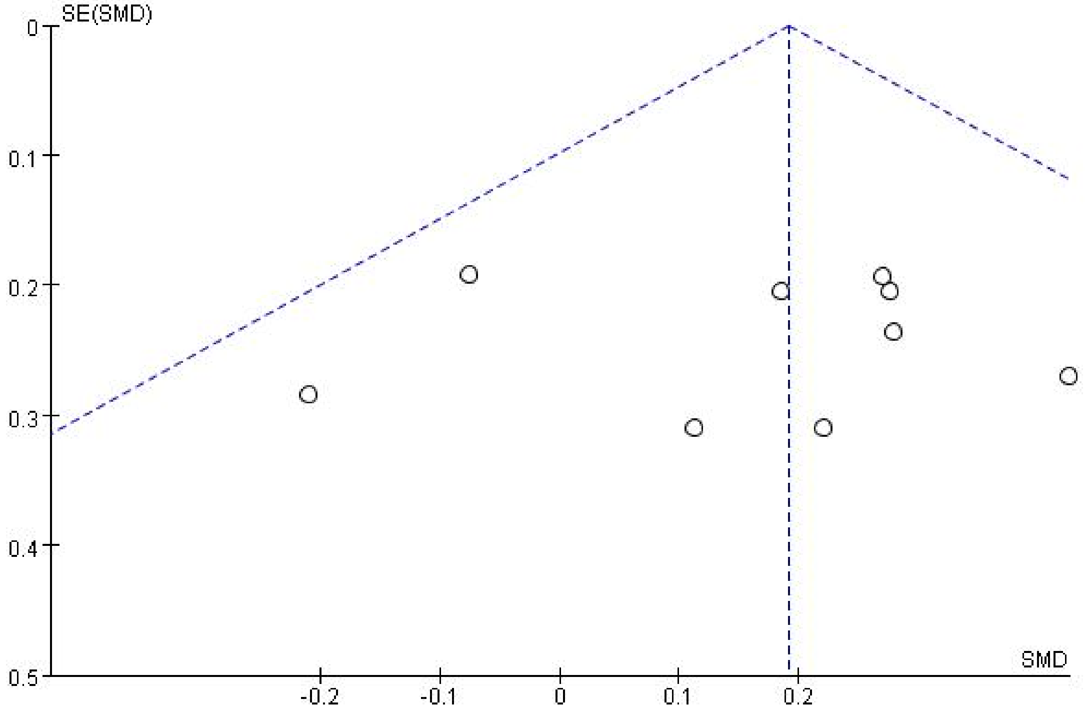

2.9 CD4/CD8(Immune)

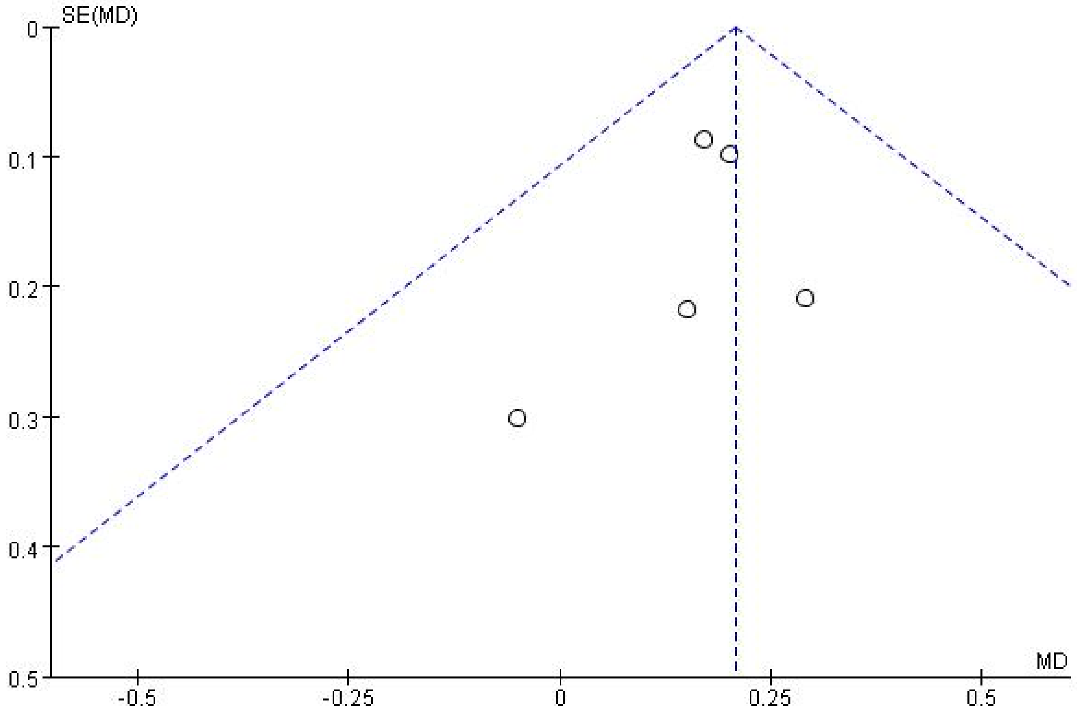

2.10 CD3(Dexmedetomidine)

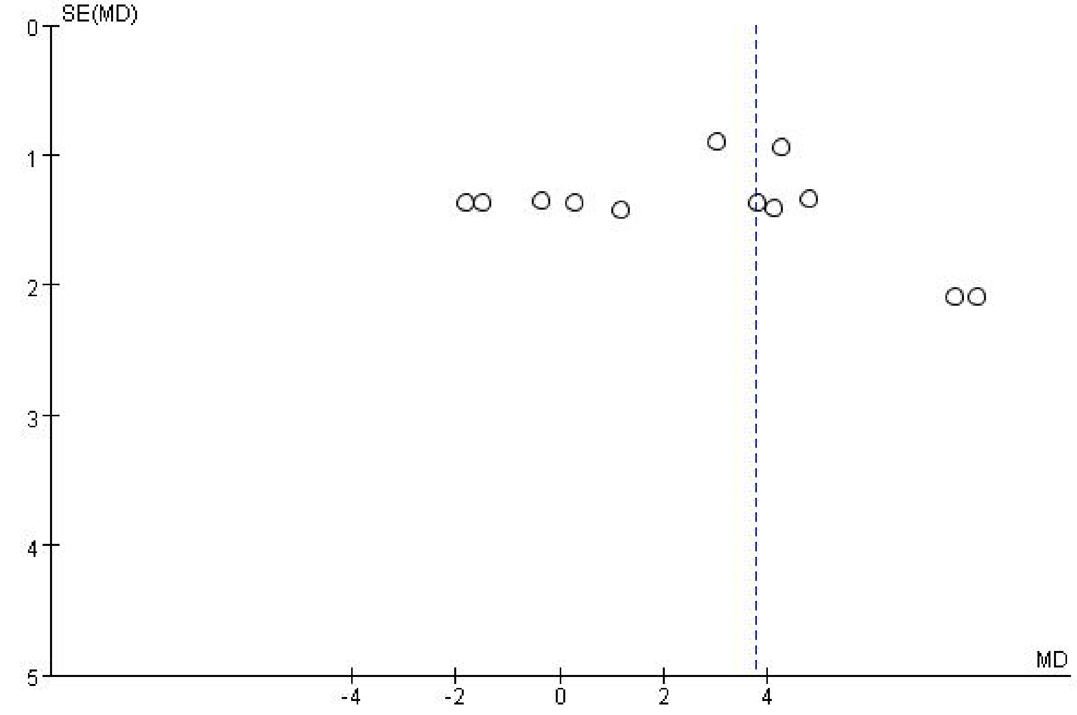

2.11 CD4(Dexmedetomidine)

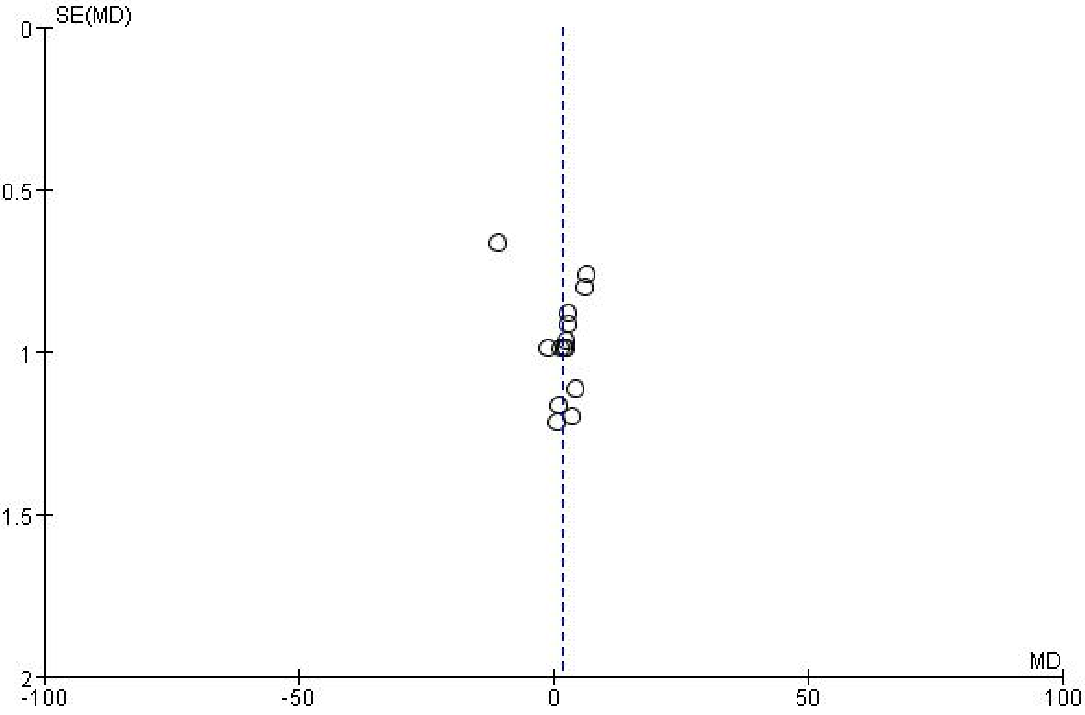

2.12CD8(Dexmedetomidine)

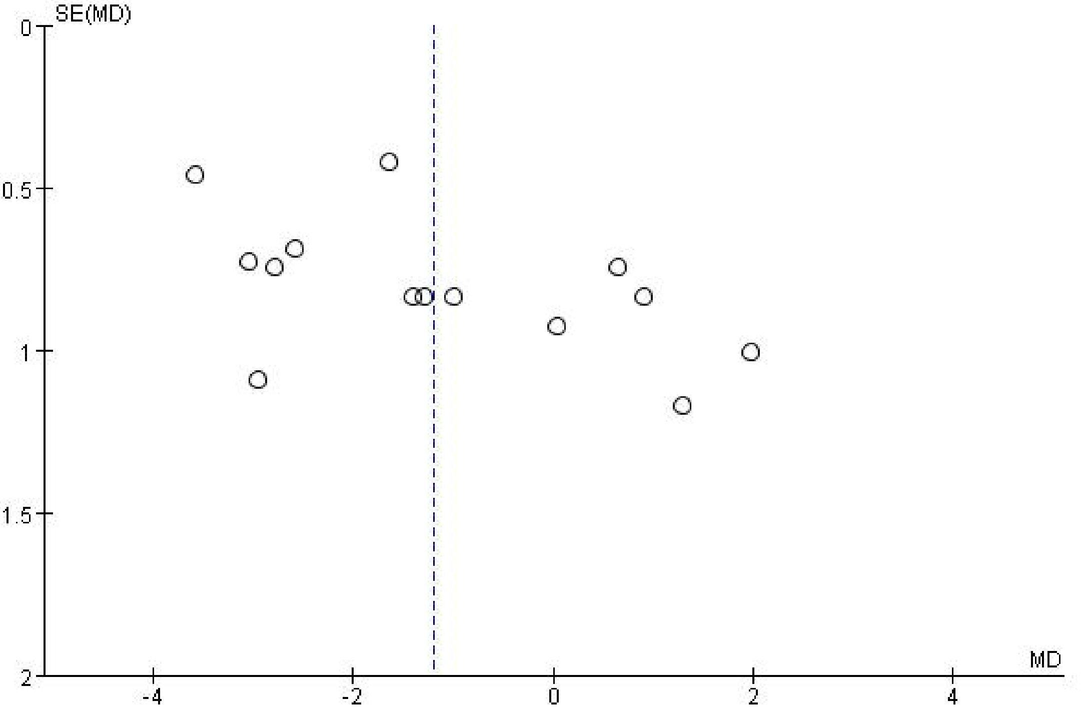

2.13 CD4/CD8(Dexmedetomidine)

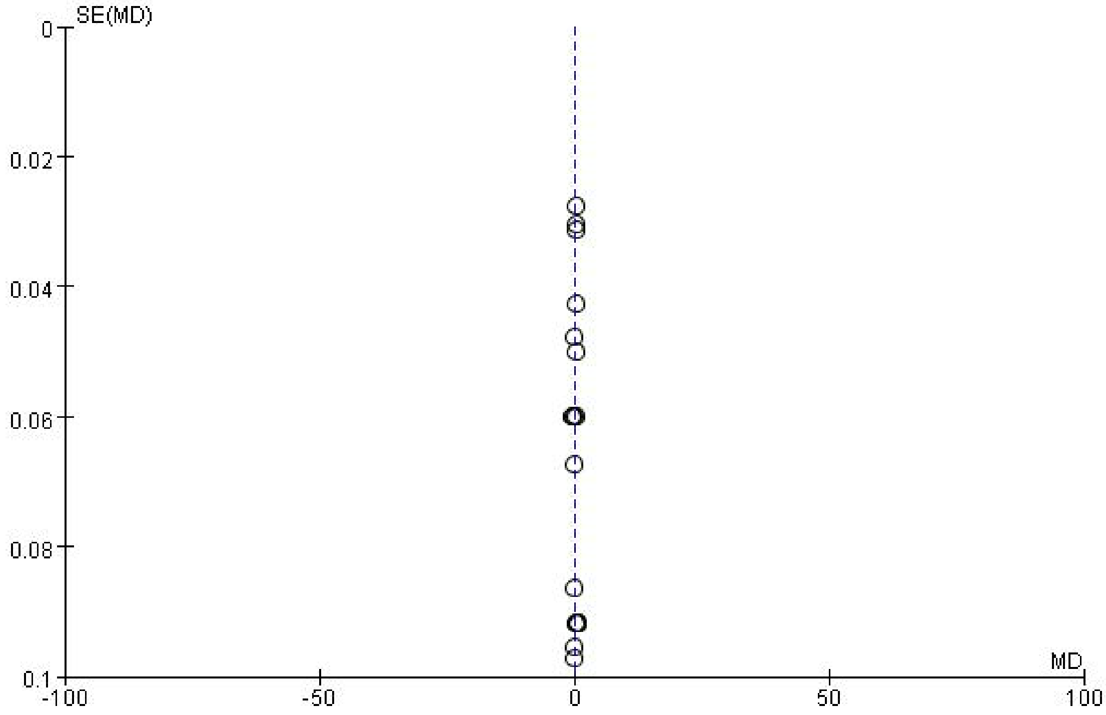

2.14 NK(Dexmedetomidine)

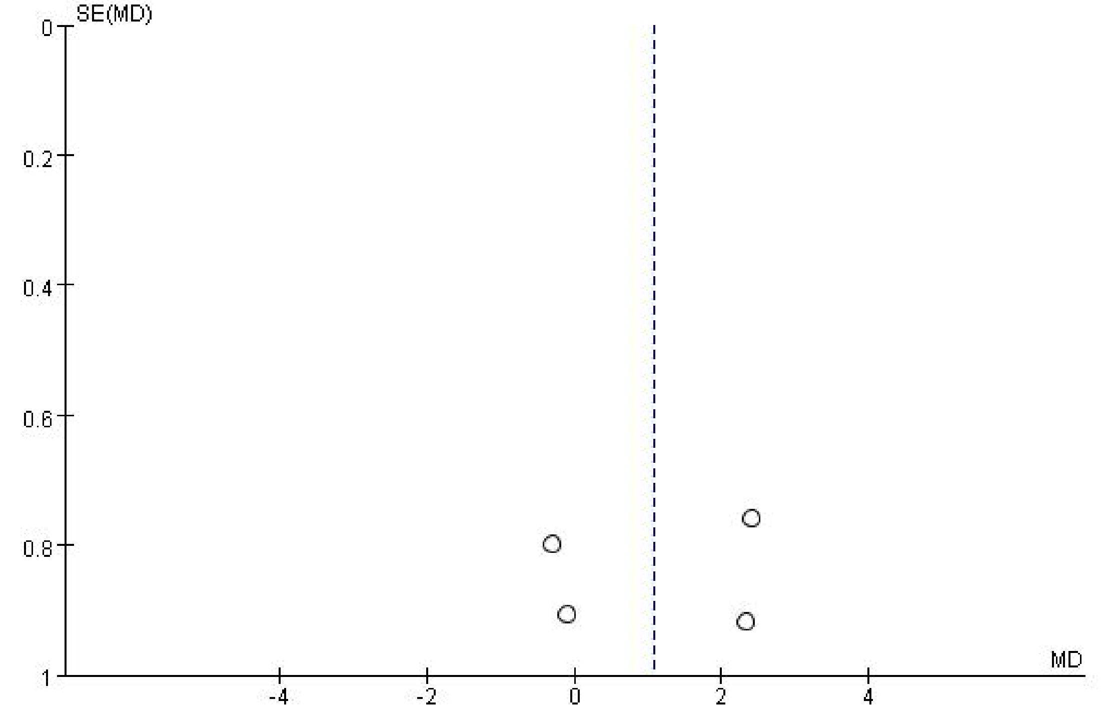

2.15 CD3(ERAS)

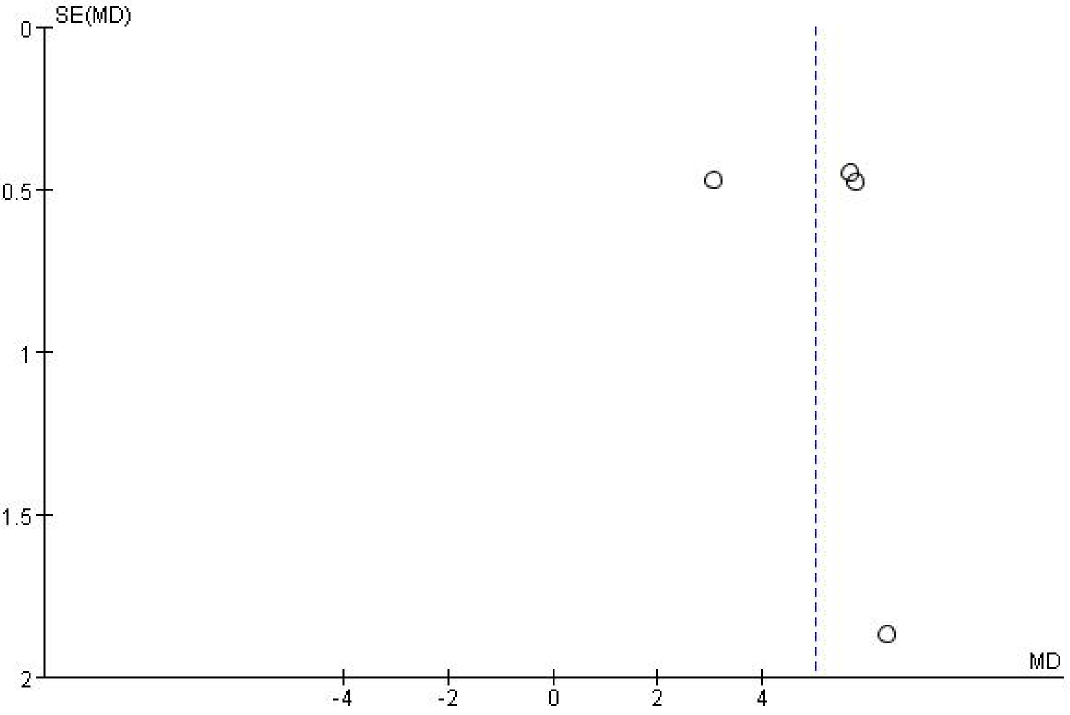

2.16
CD4(ERAS)

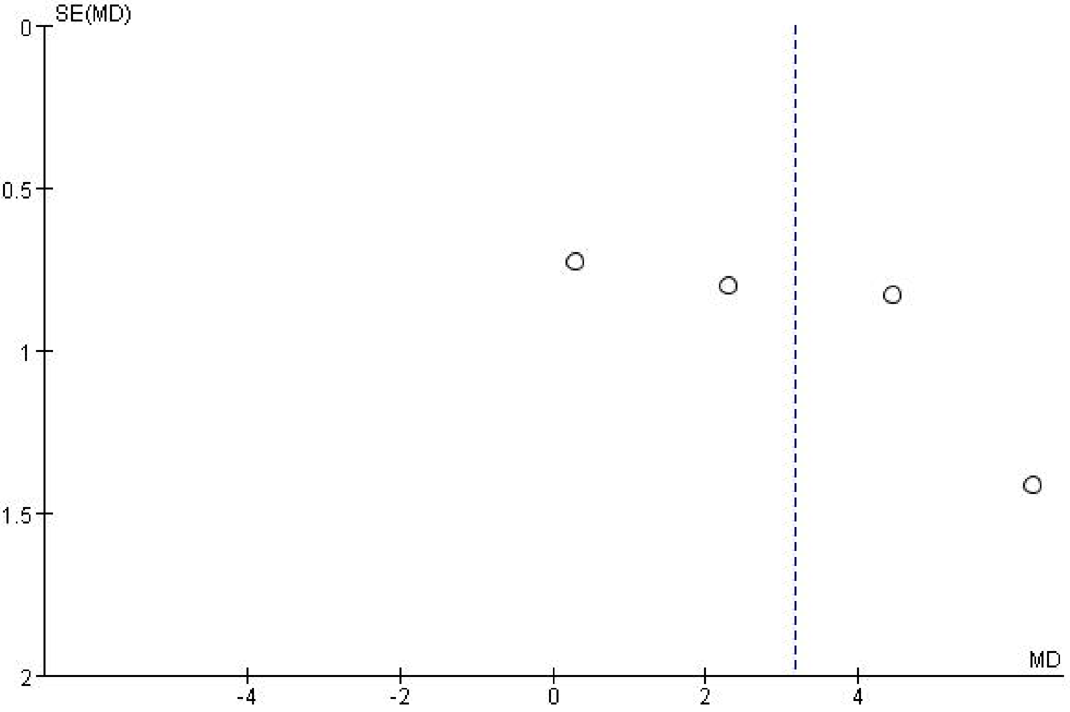

2.17 CD8(ERAS)

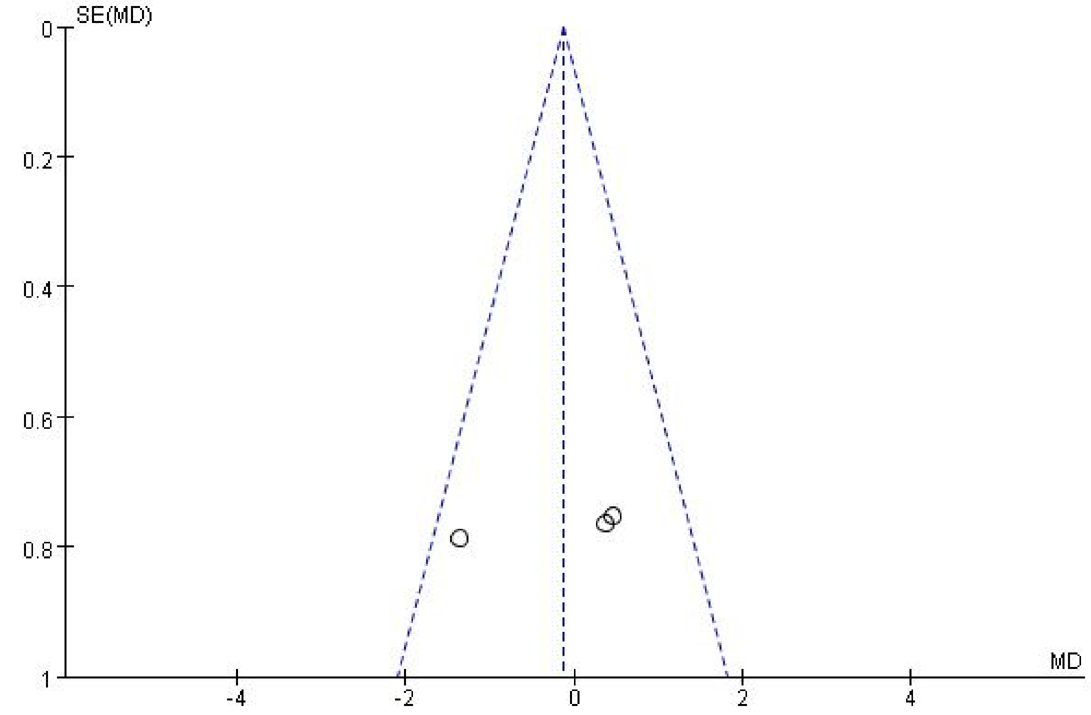

2.18 CD4/CD8(ERAS)

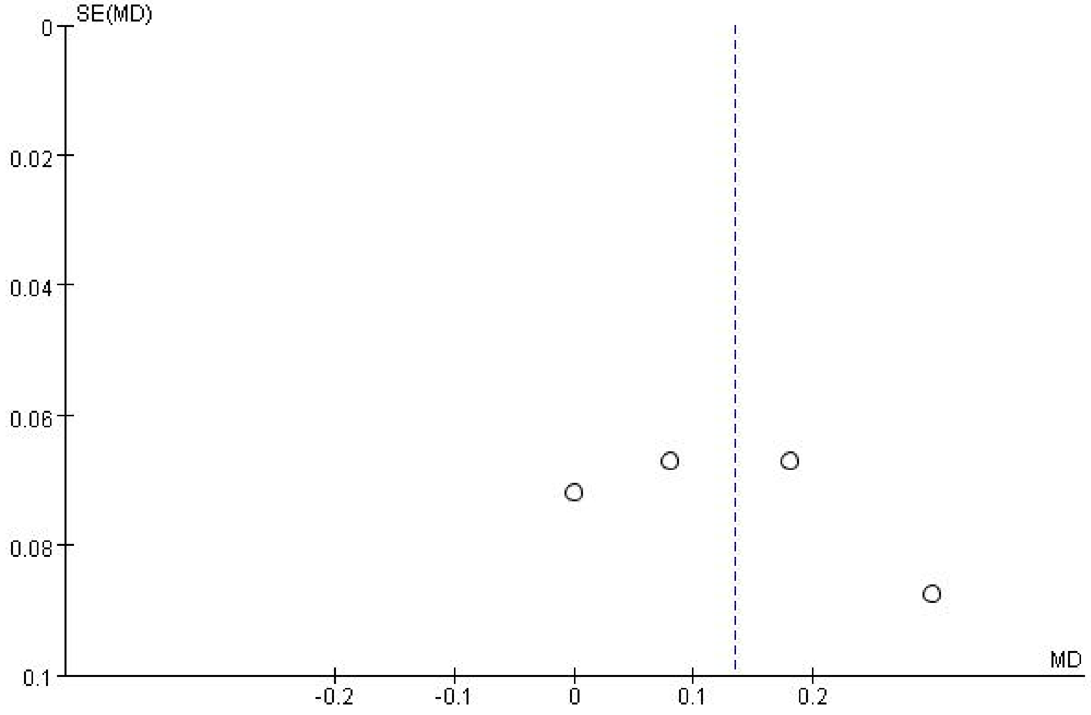

2.19 CD3(Psychotherapy)

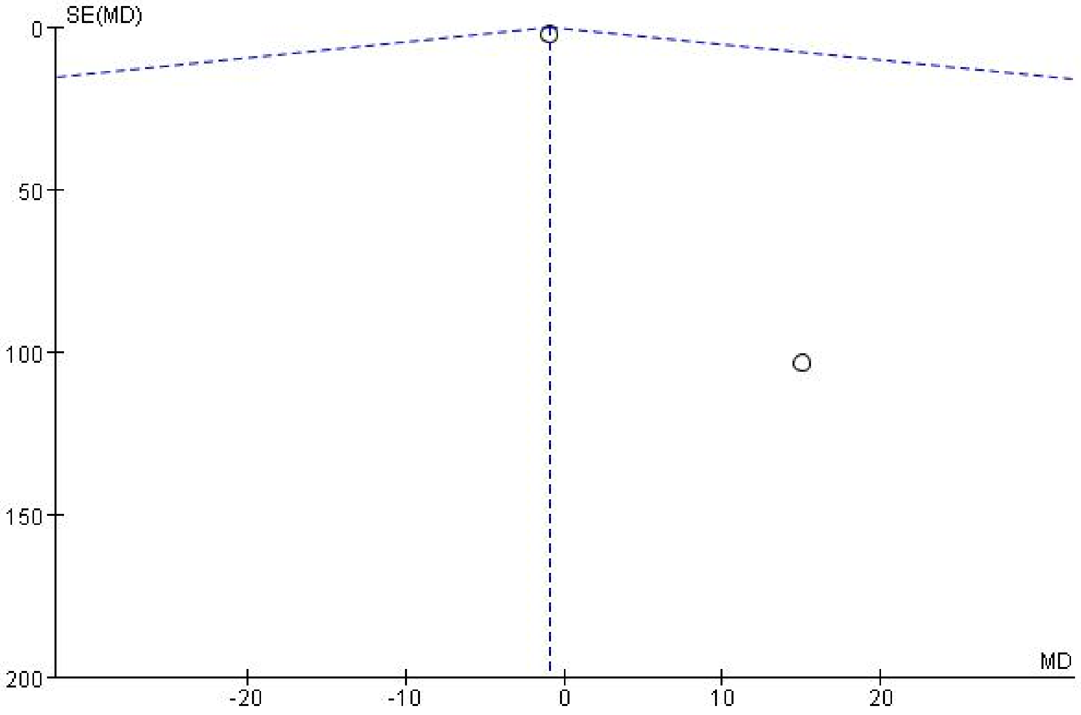

2.20 CD4(Psychotherapy)

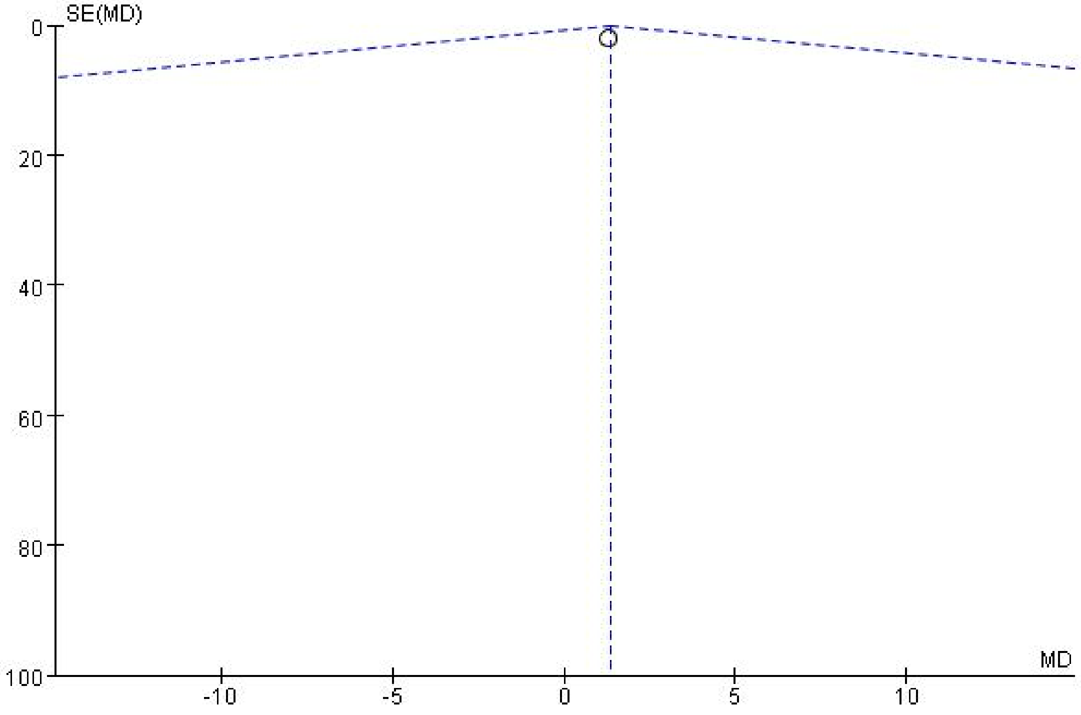

2.21 CD8(Psychotherapy)

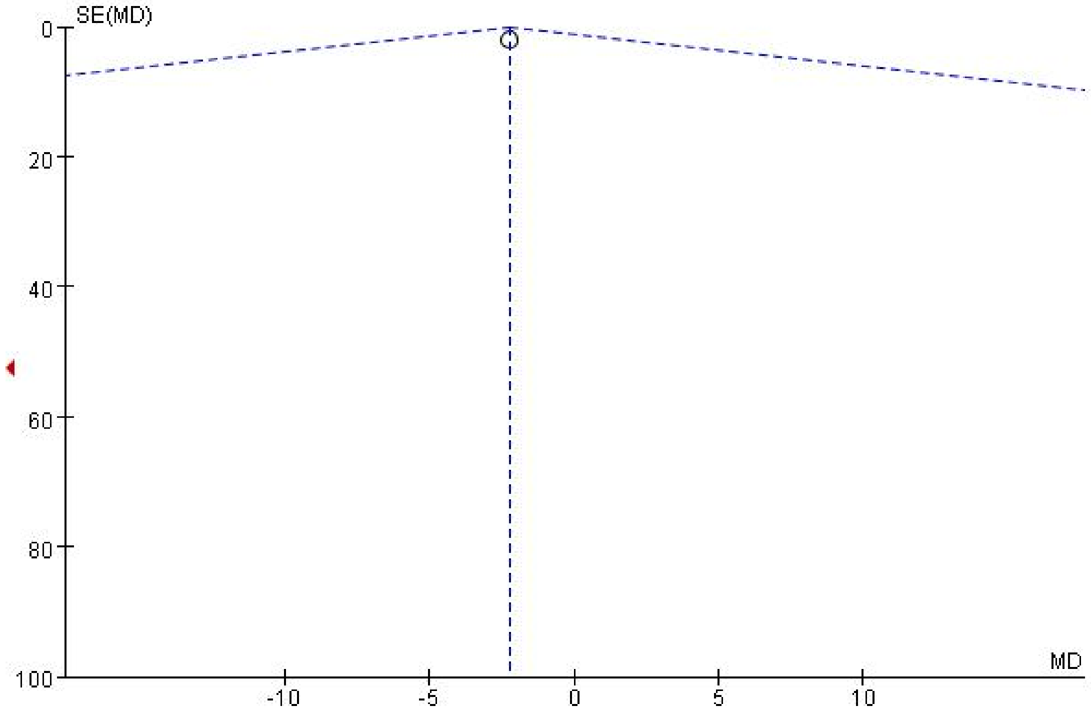

2.22 CD4/CD8(Psychotherapy)

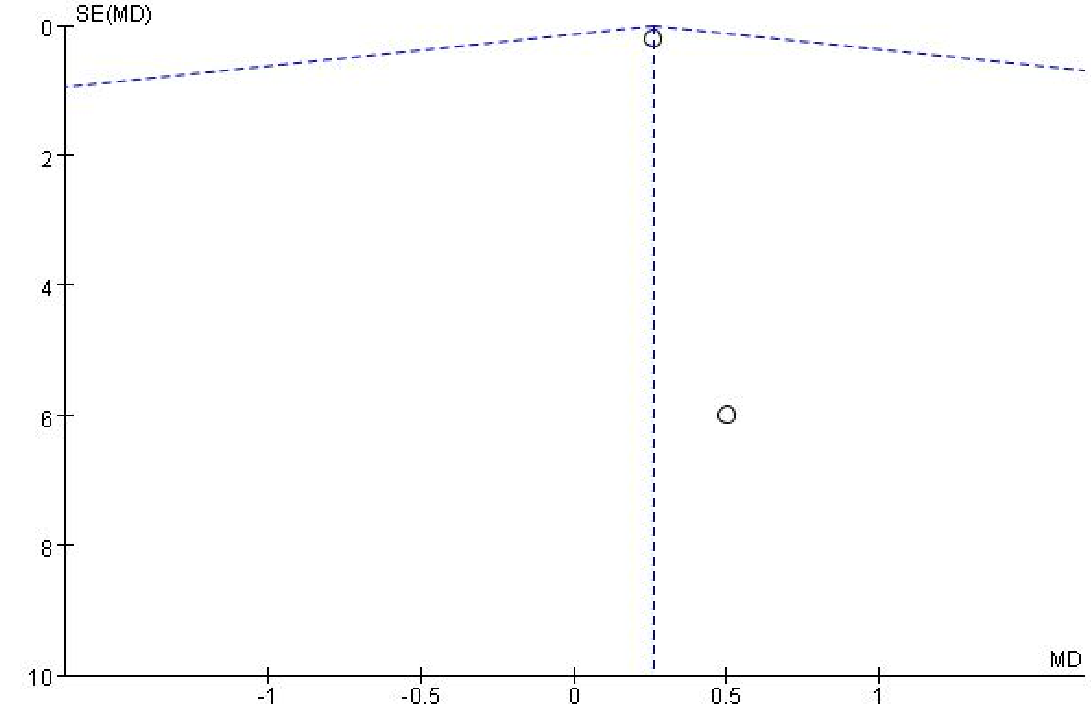

2.23 CD3(TCM)

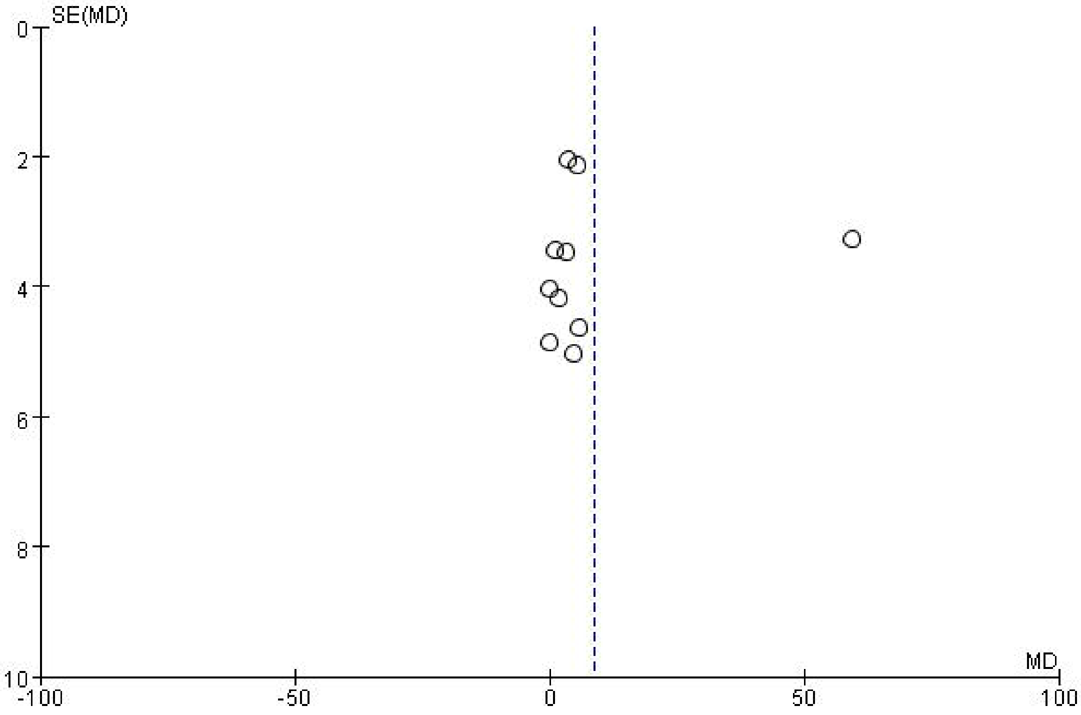

2.24 CD4(Psychotherapy)

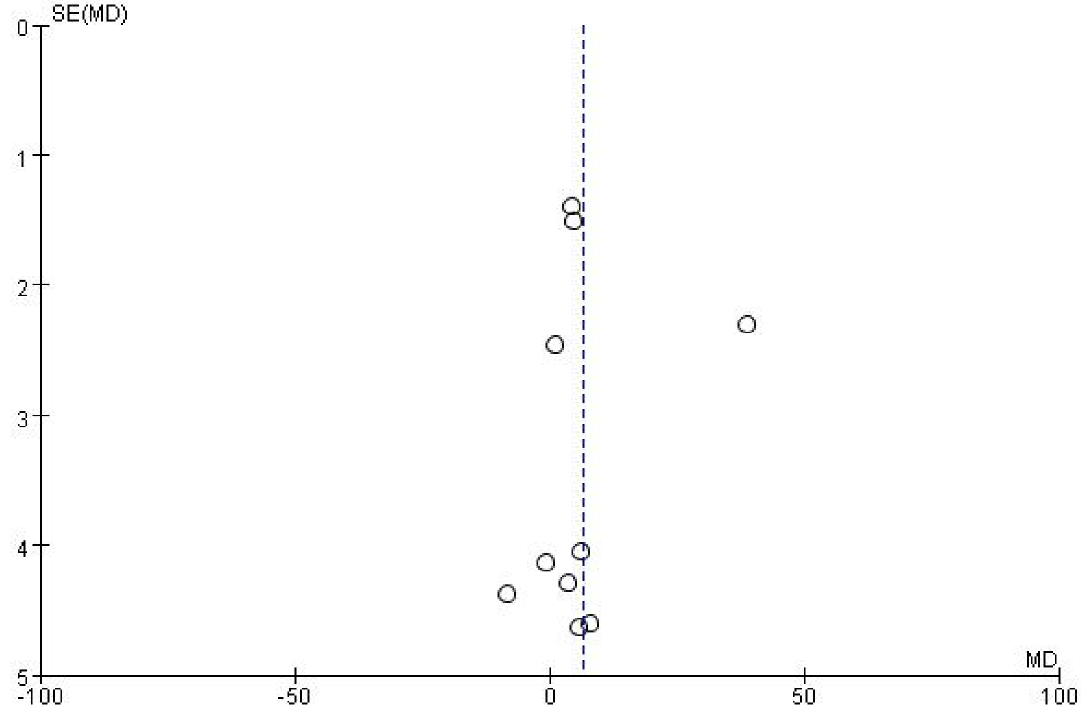

2.25 CD8(Psychotherapy)

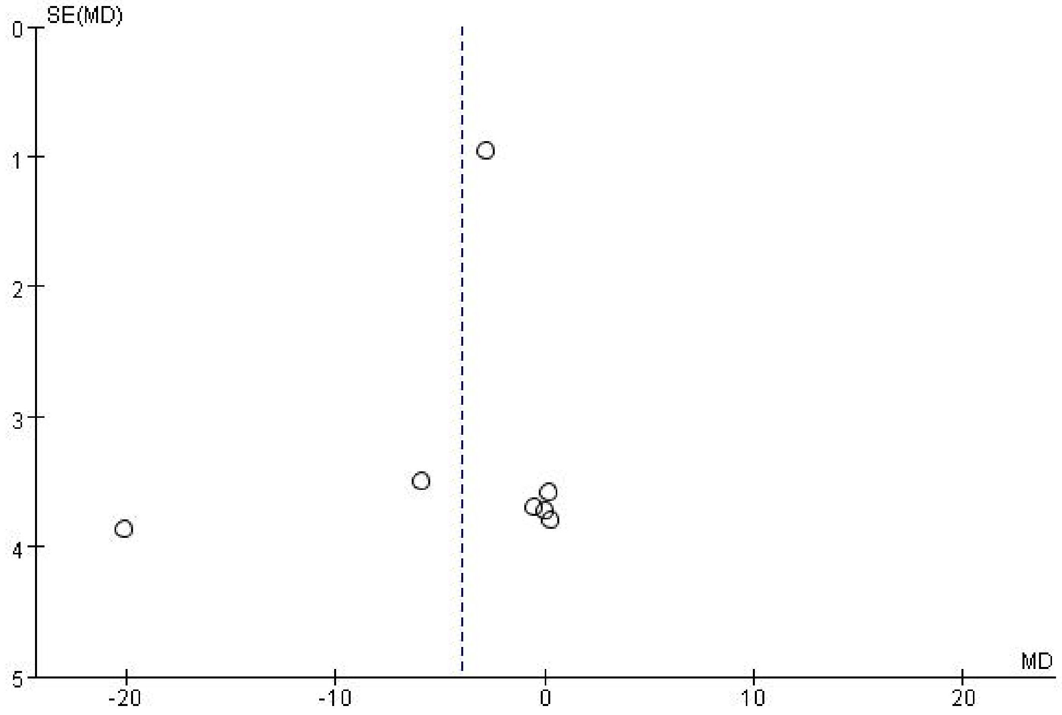

2.26CD4/CD8(Psychotherapy)

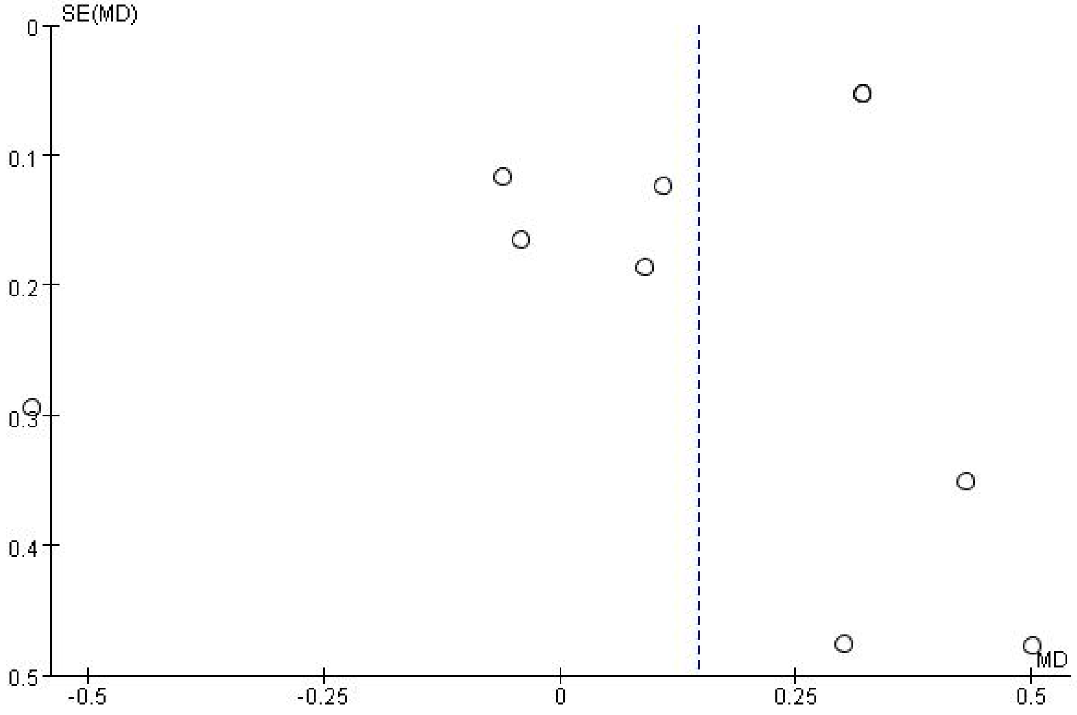

2.27 NK(Psychotherapy)

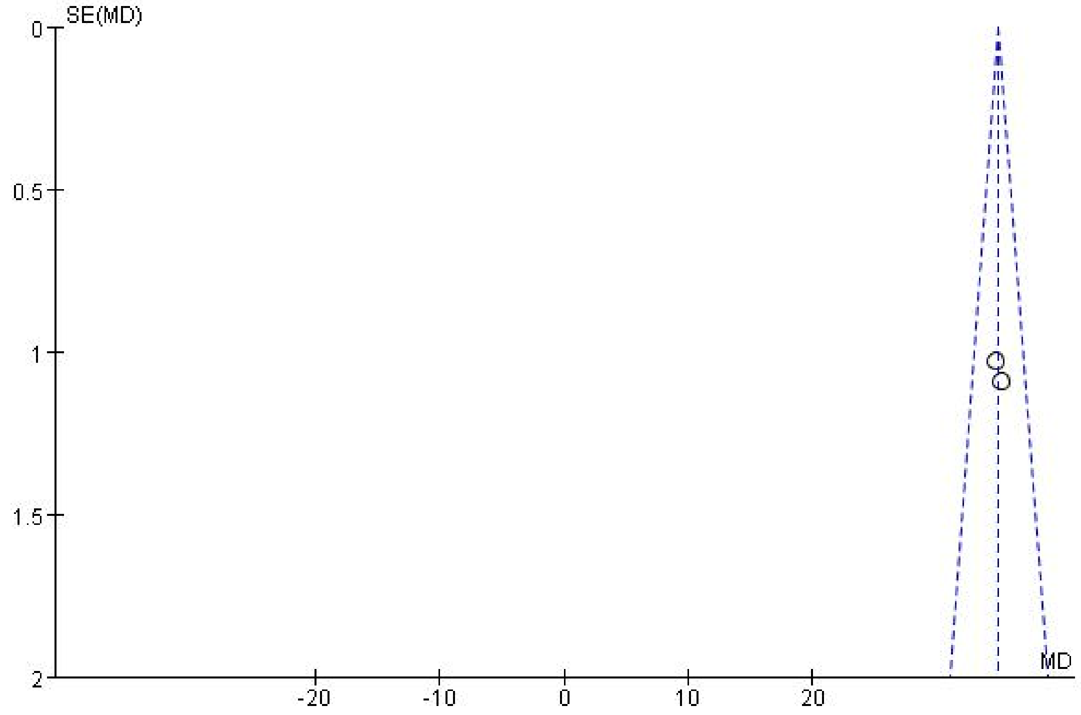

2.28 CD3(TP)

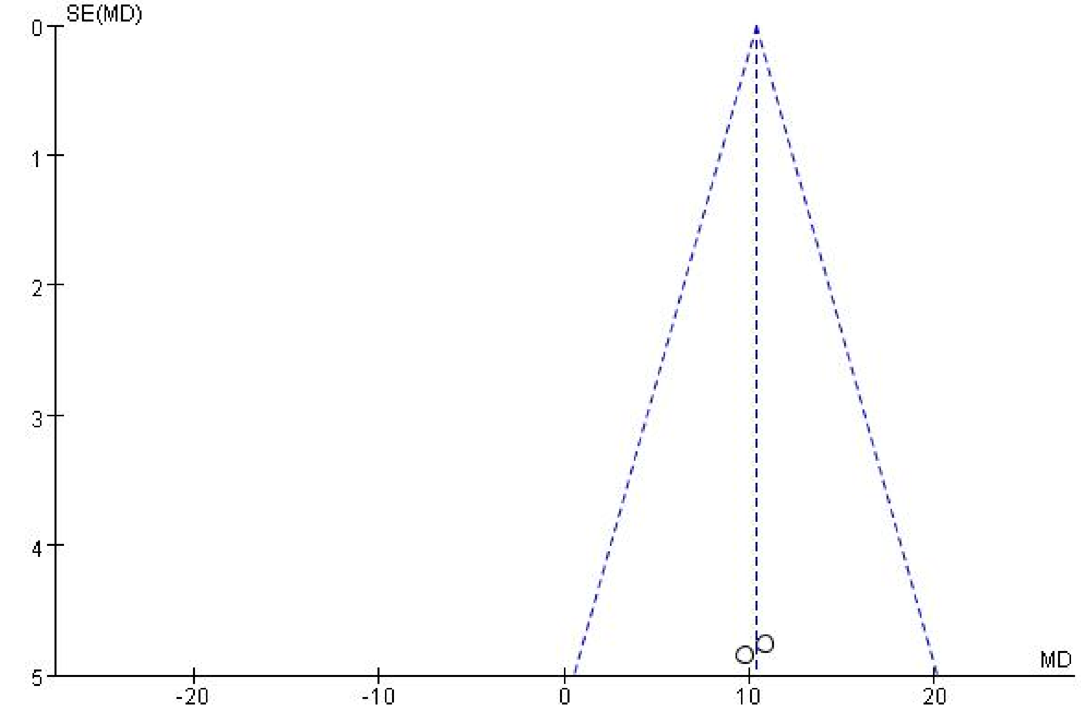

2.29 CD4(TP)

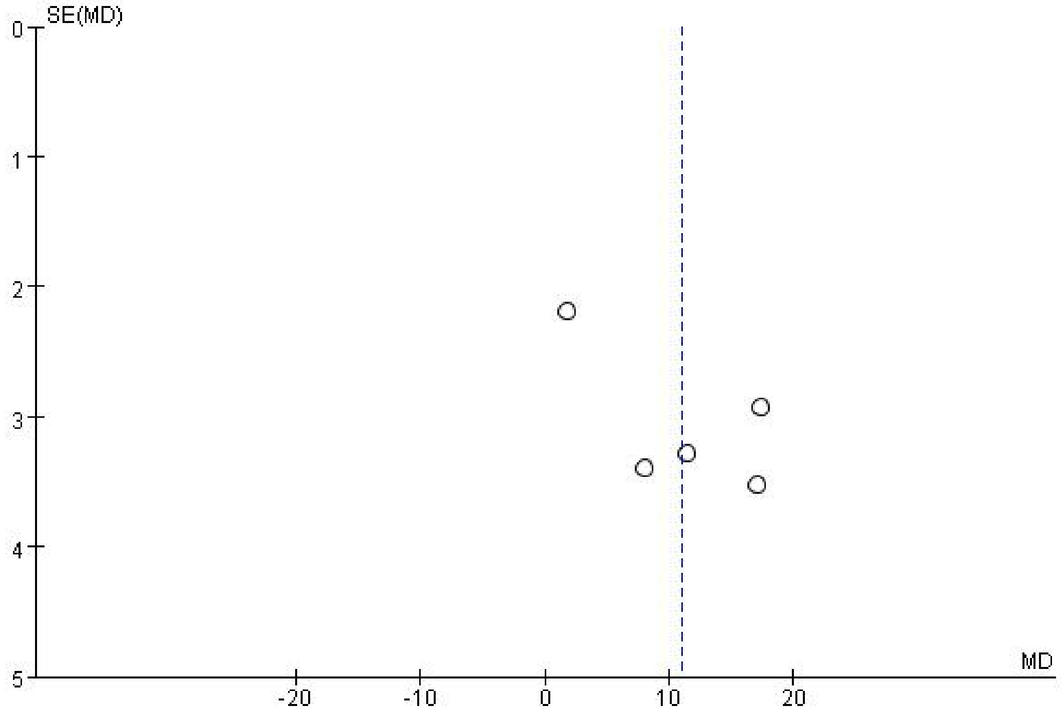

2.30 CD8(TP)

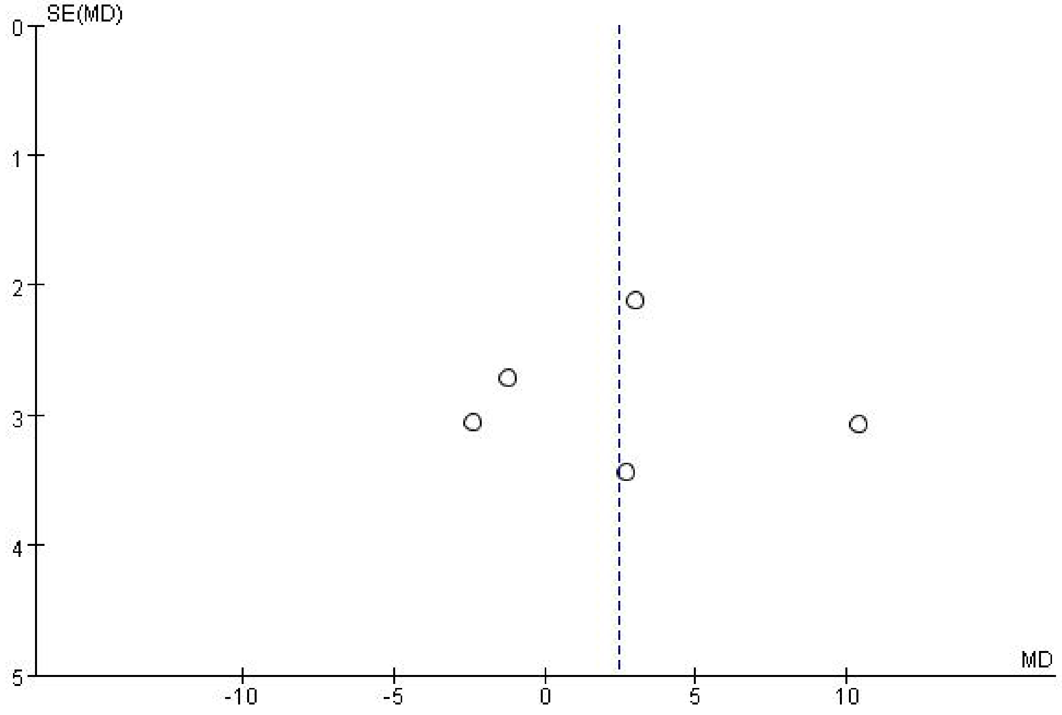

2.31 CD4/CD8(TP)

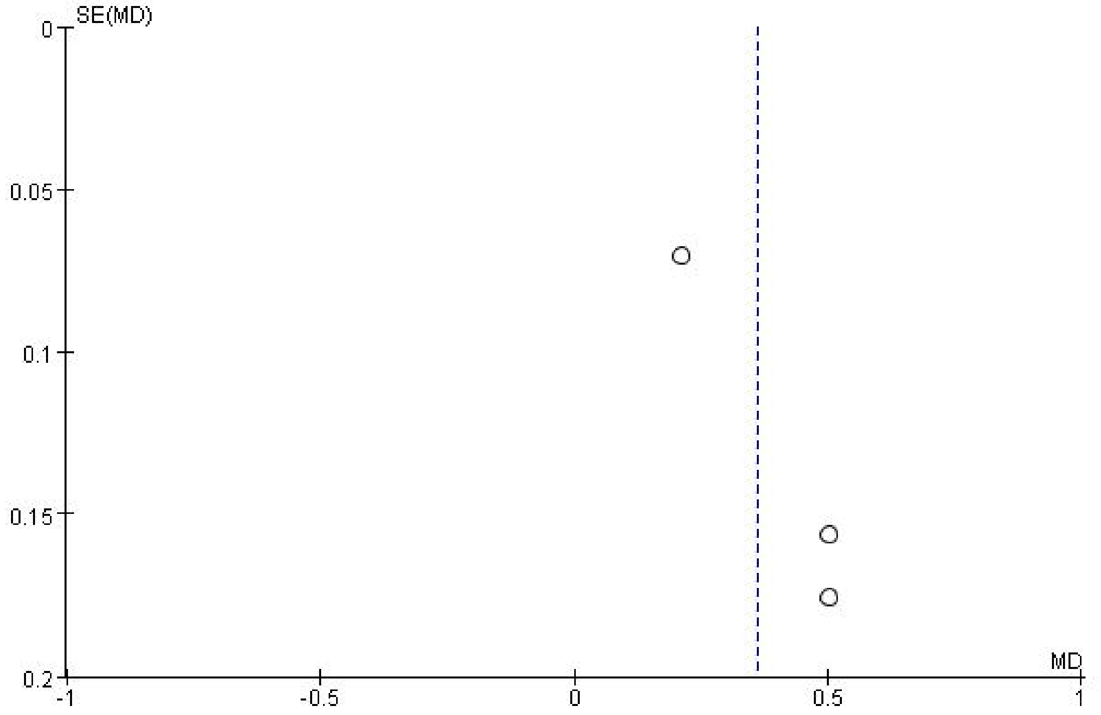

2.32 NK(TP)

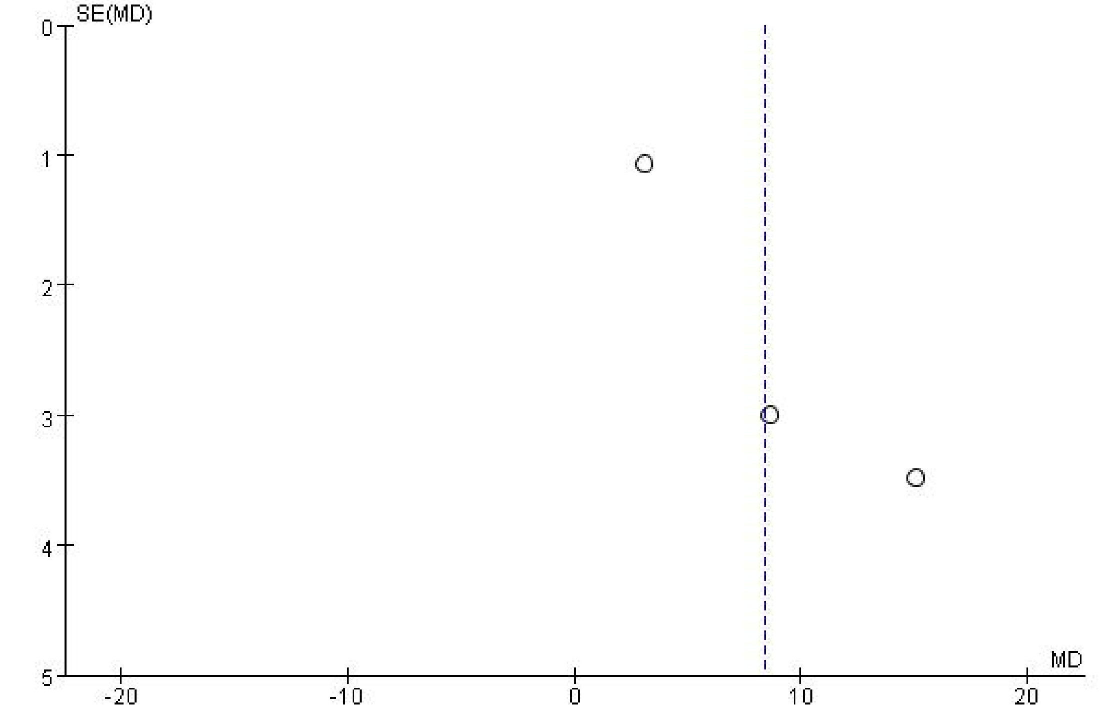

2.33 CD3(TD)

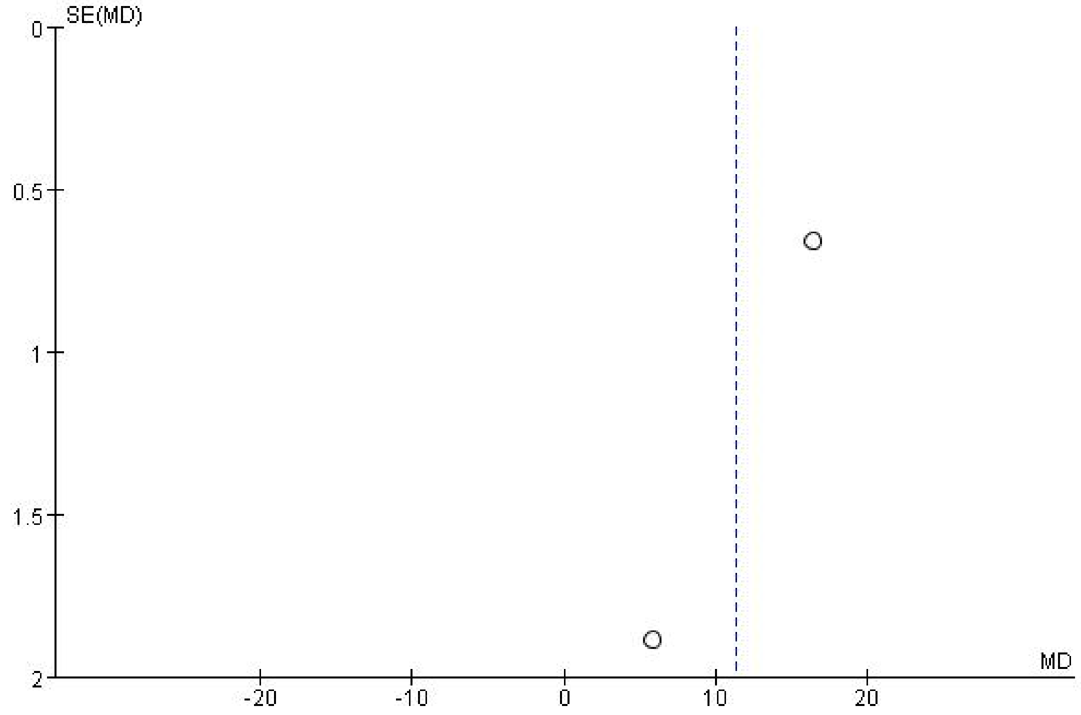

2.34 CD4(TD)

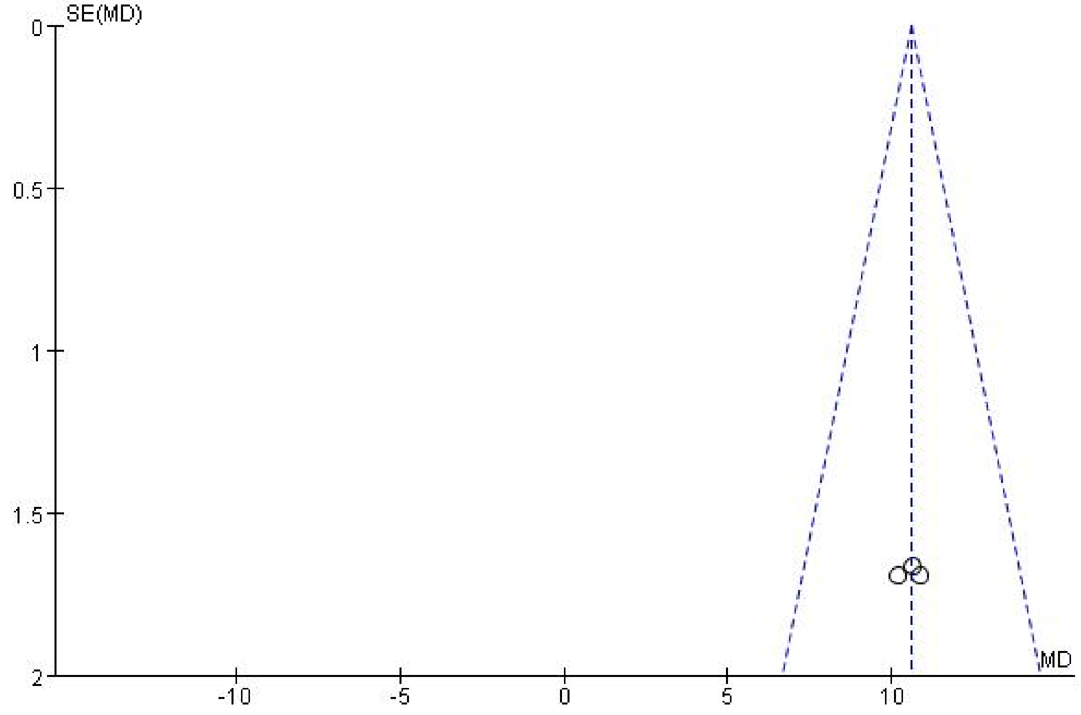

2.35 CD8(TD)

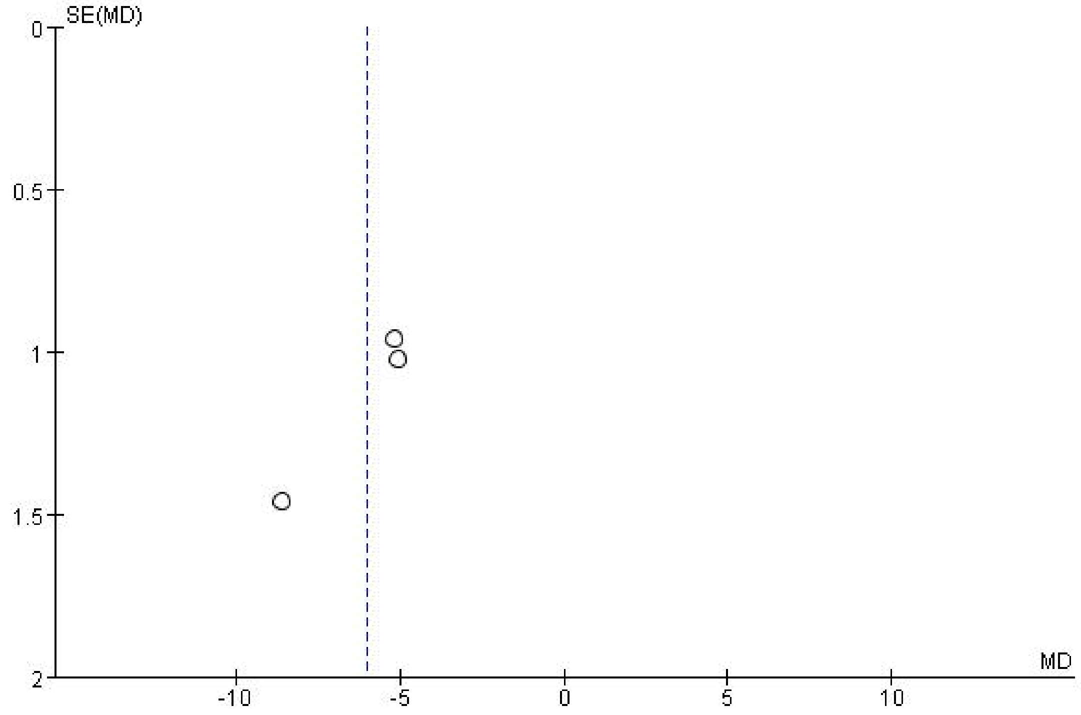

2.36 CD4/CD8(TD)

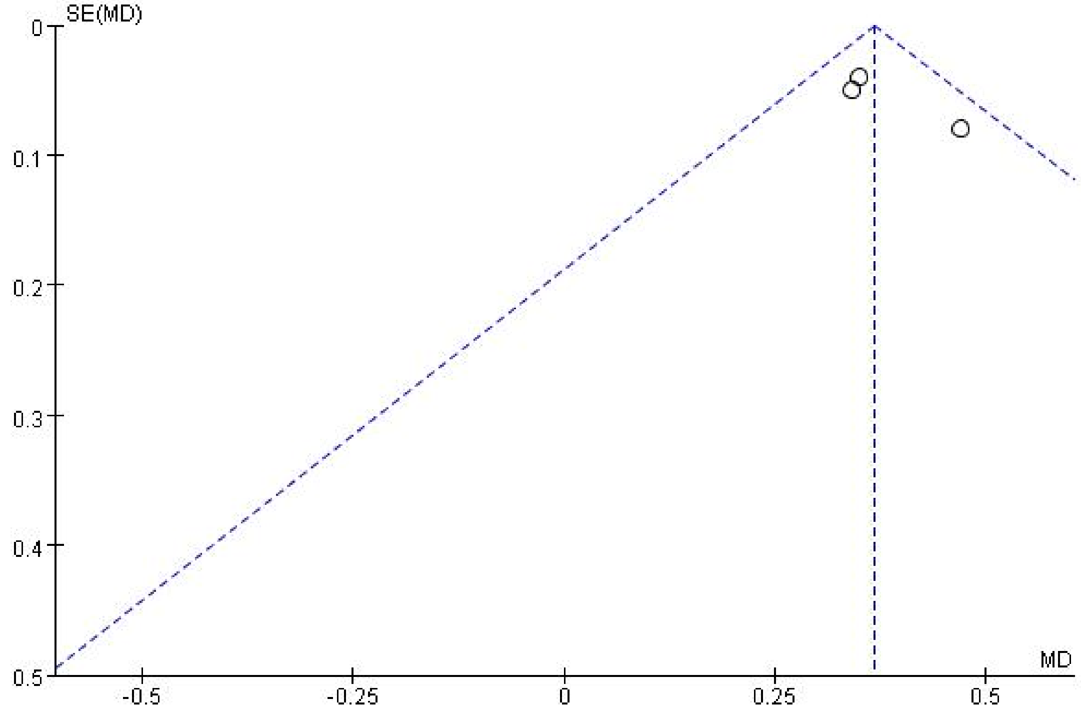

2.37 NK(TD)

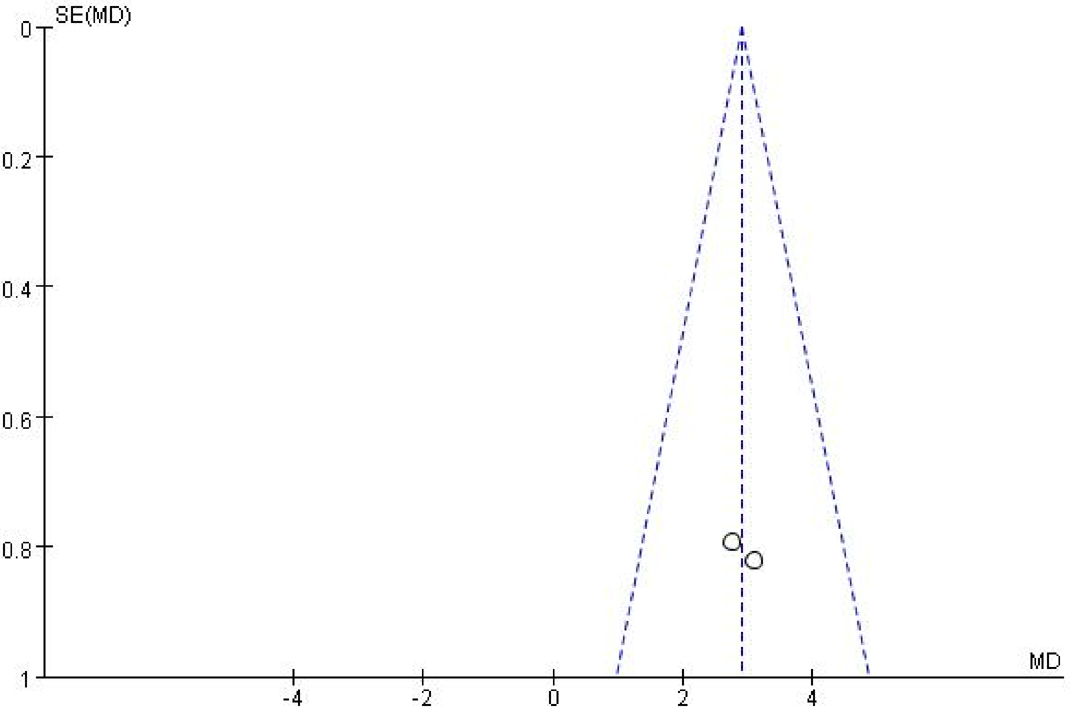

2.38 CD3(CD)

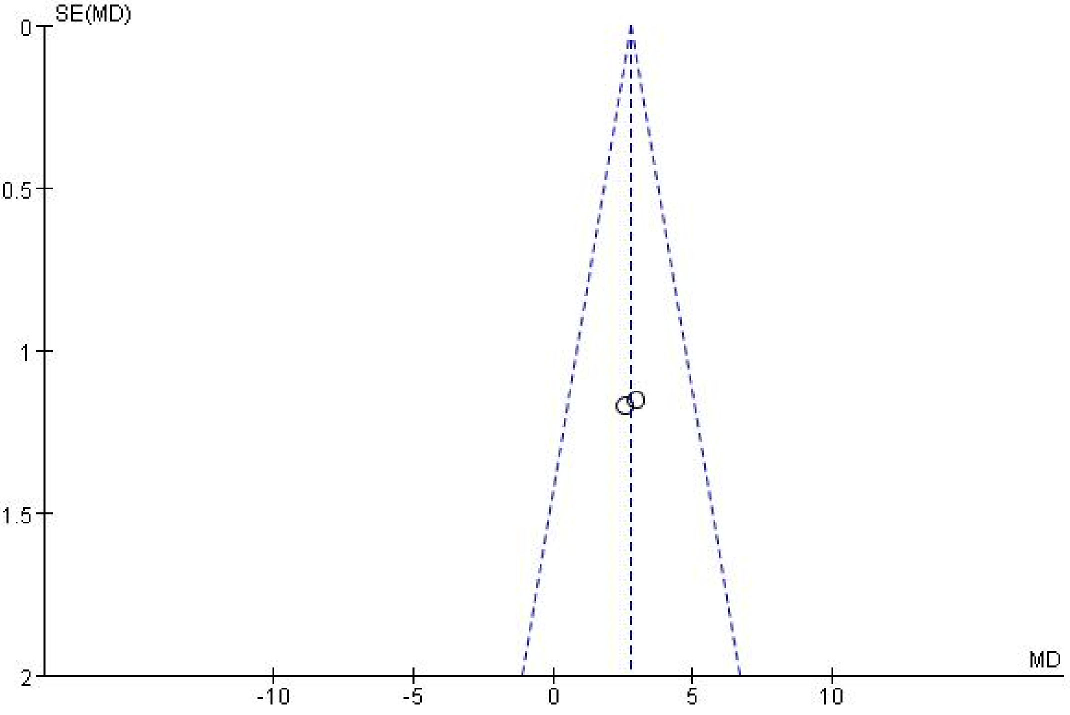

2.39 CD4(CD)

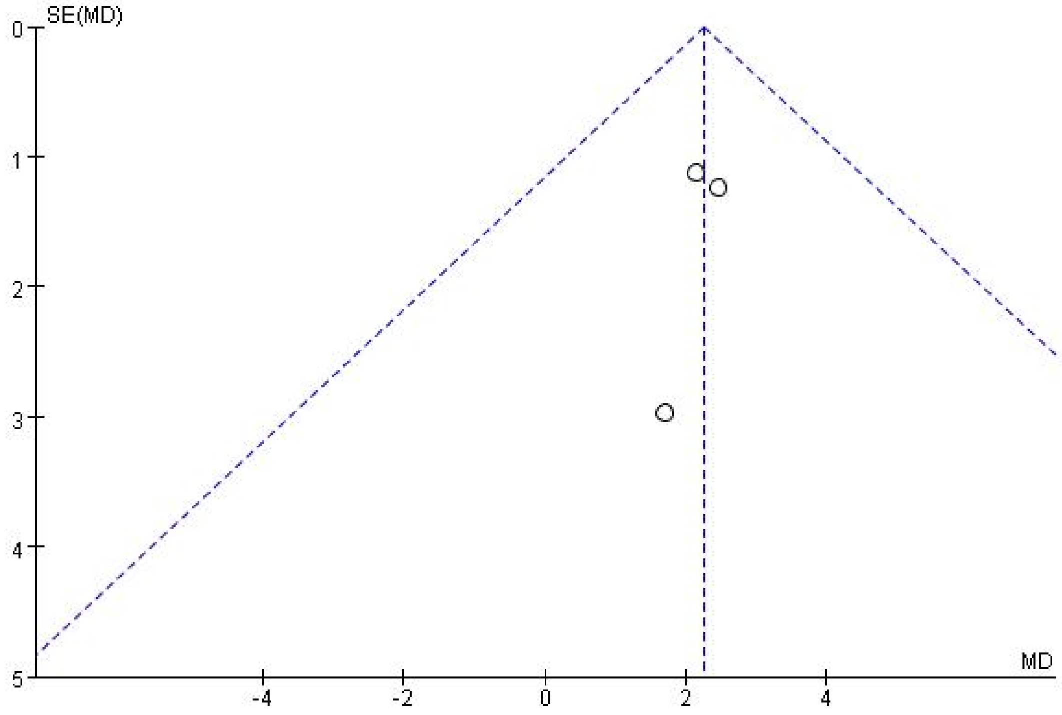

2.40 CD8(CD)

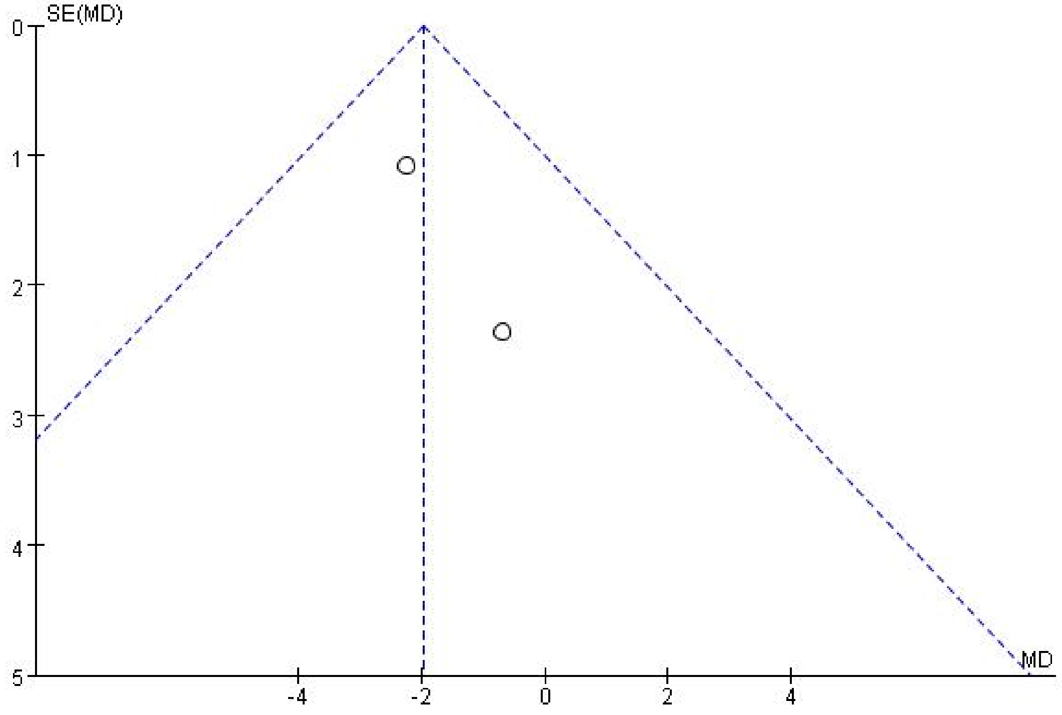

2.41 CD4/CD8(CD)

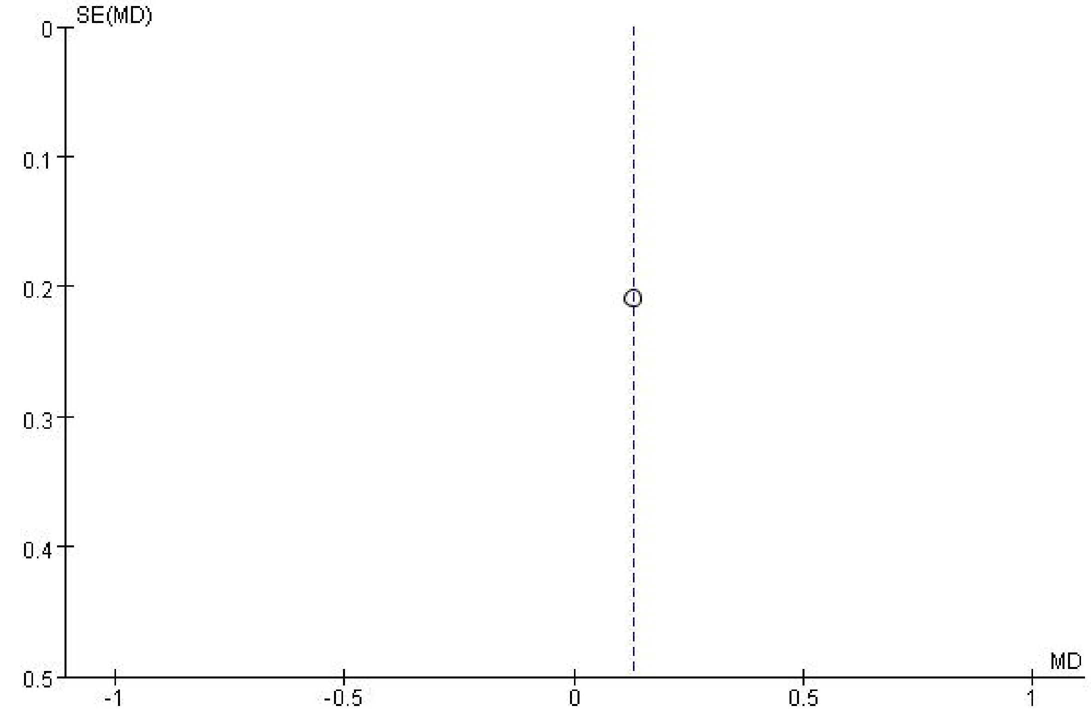

2.42 NK(CD)

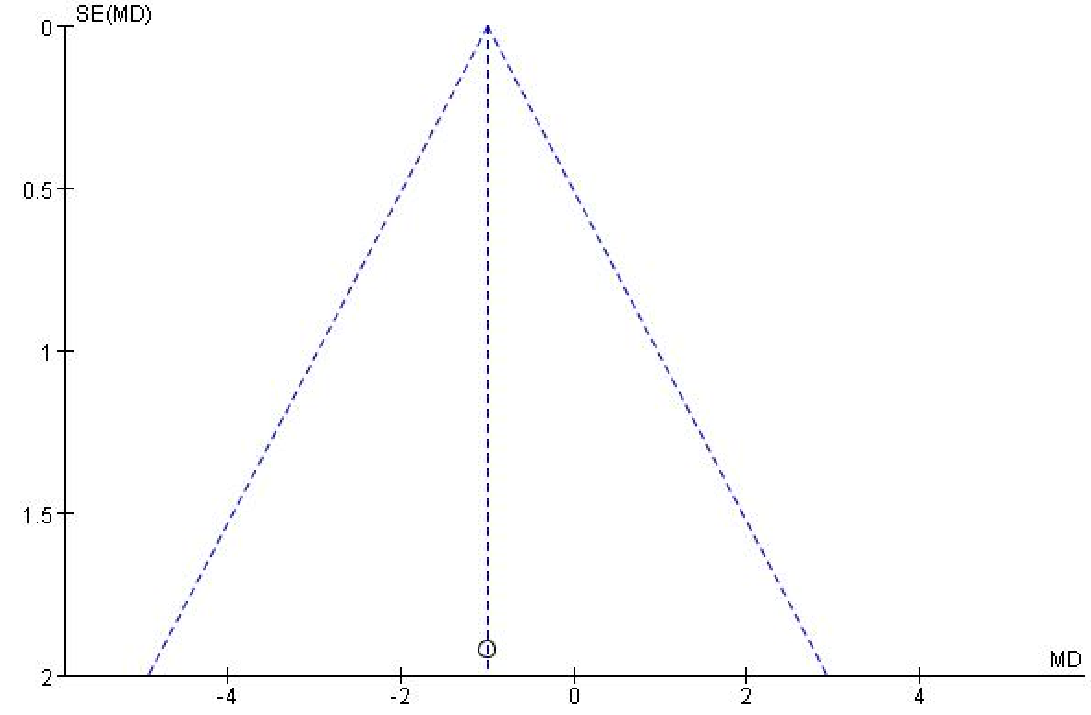

2.43CD3(NIV)

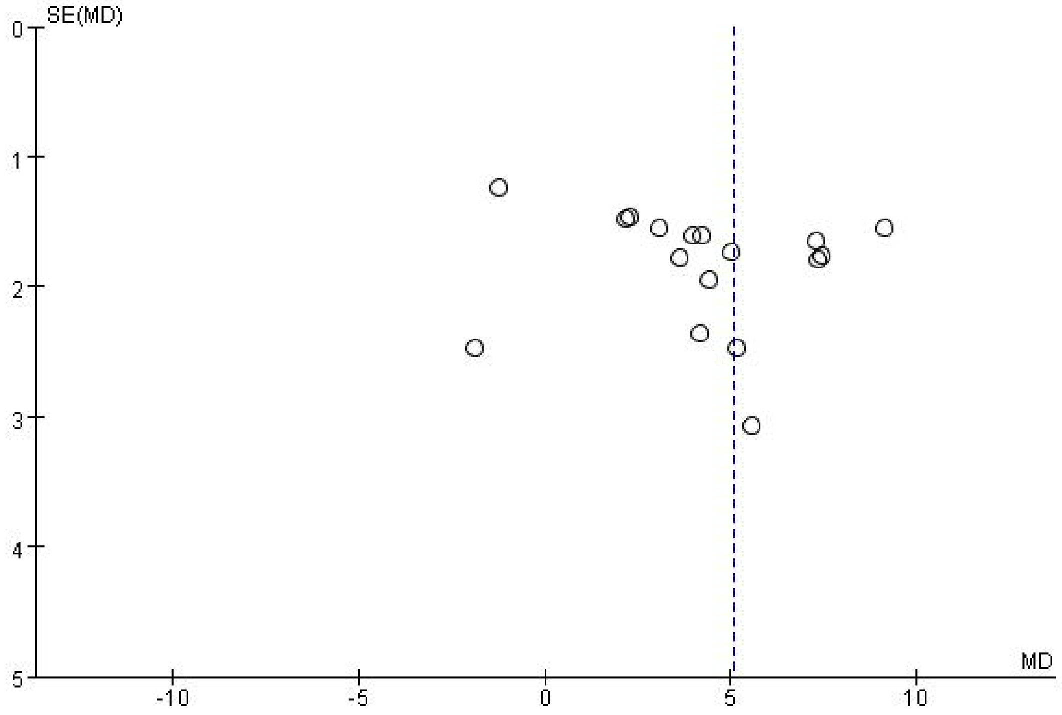

2.44 CD4(NIV)

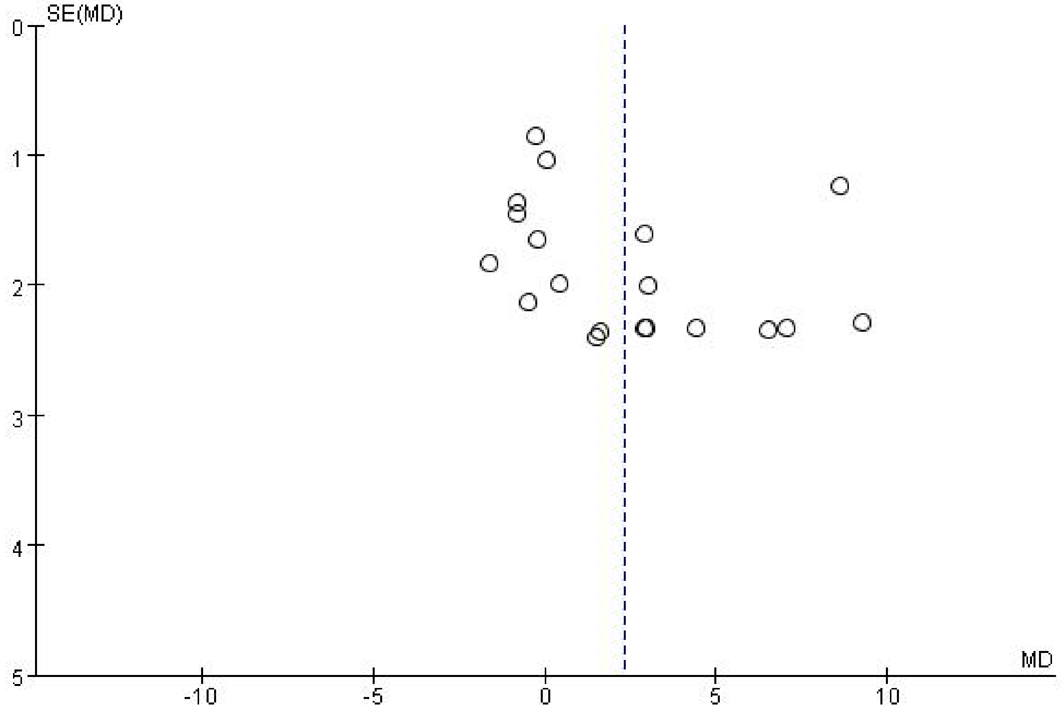

2.45 CD8(NIV)

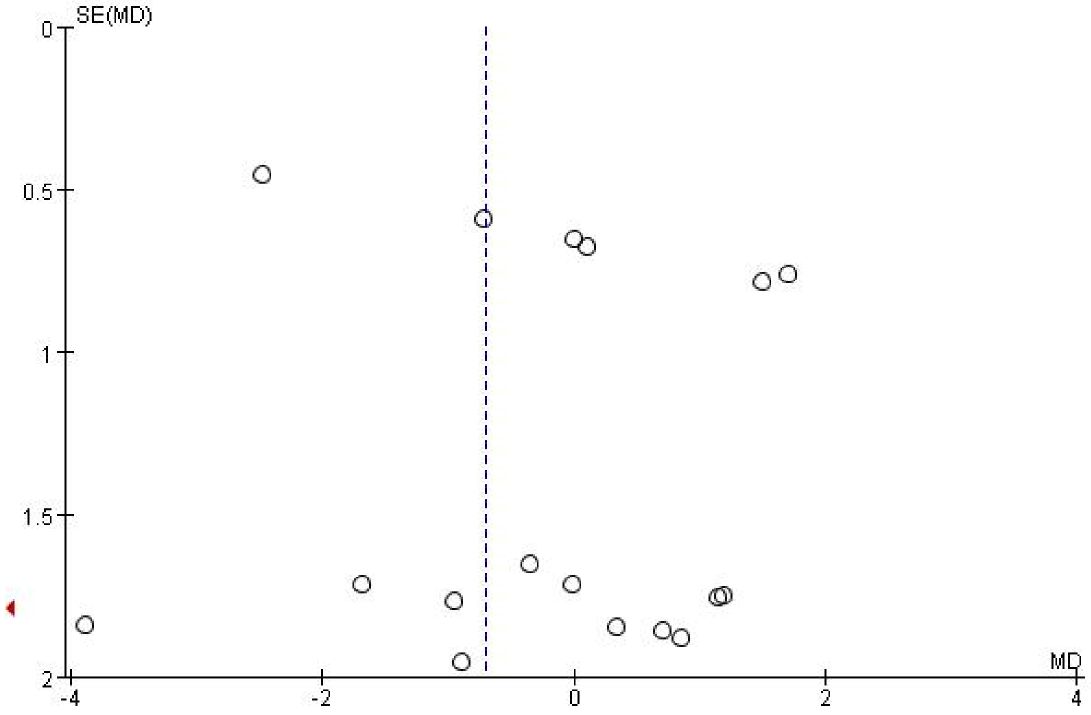

2.46 CD4/CD8(NIV)

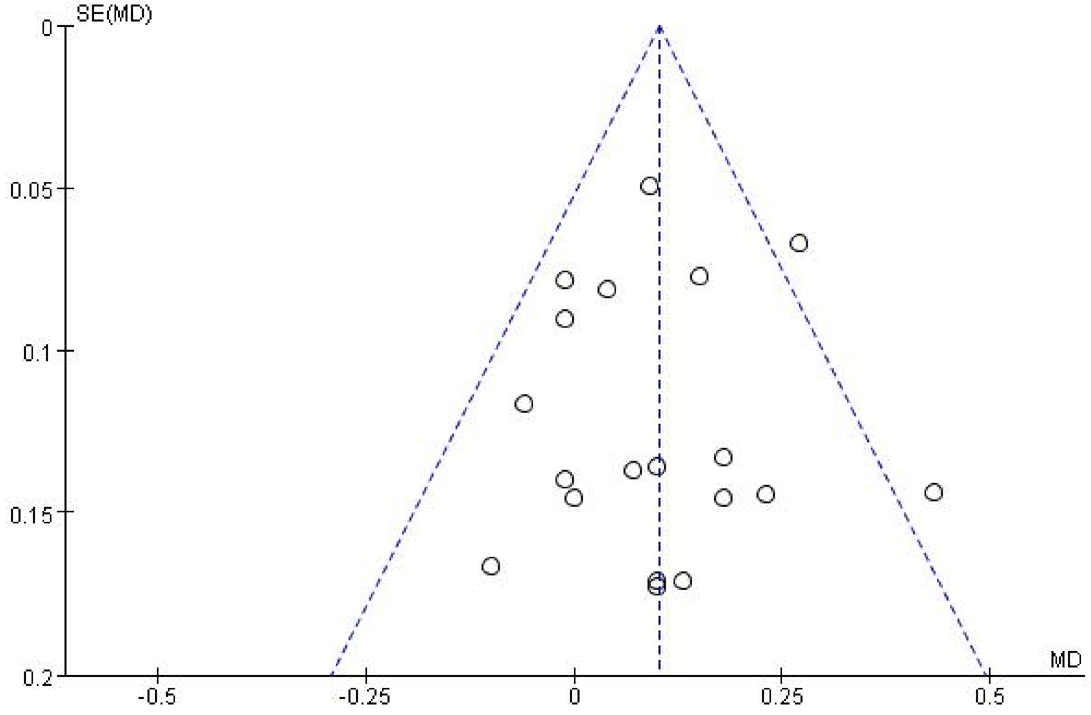

2.47 CD3(PPF)

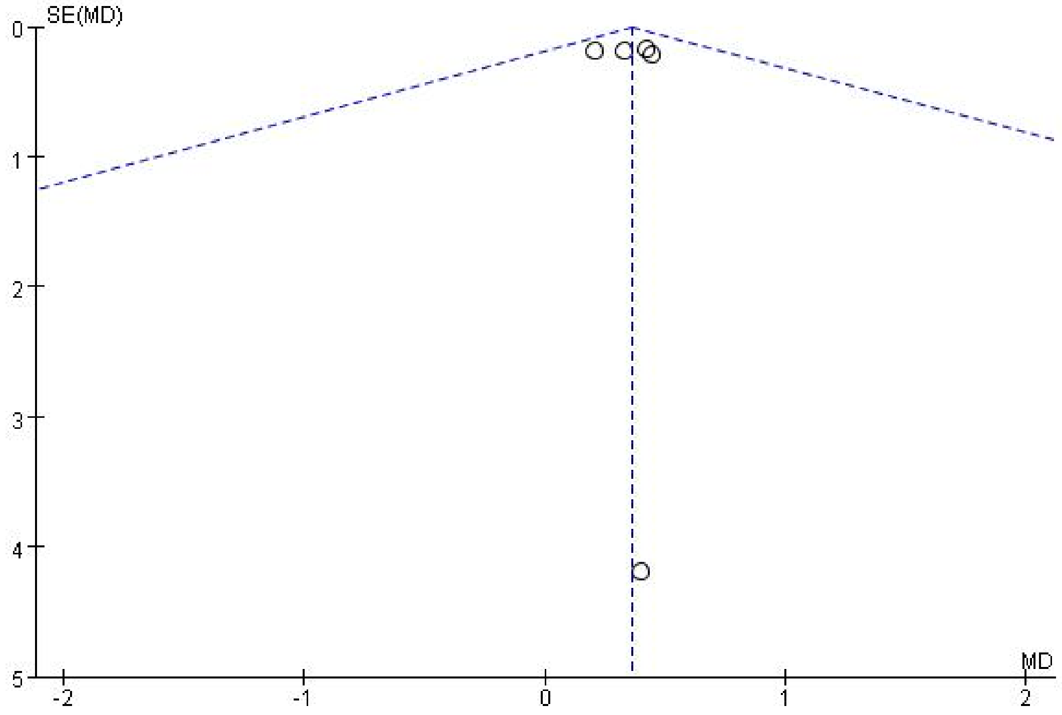

2.48 CD4(PPF)

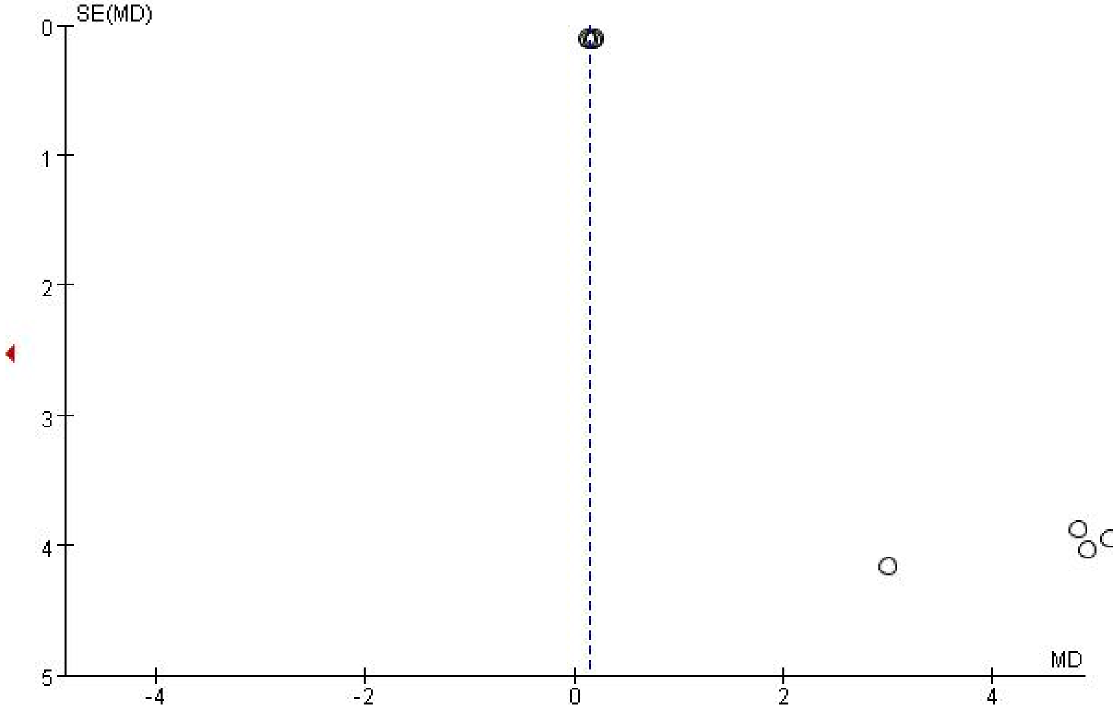

2.49 CD8(PPF)

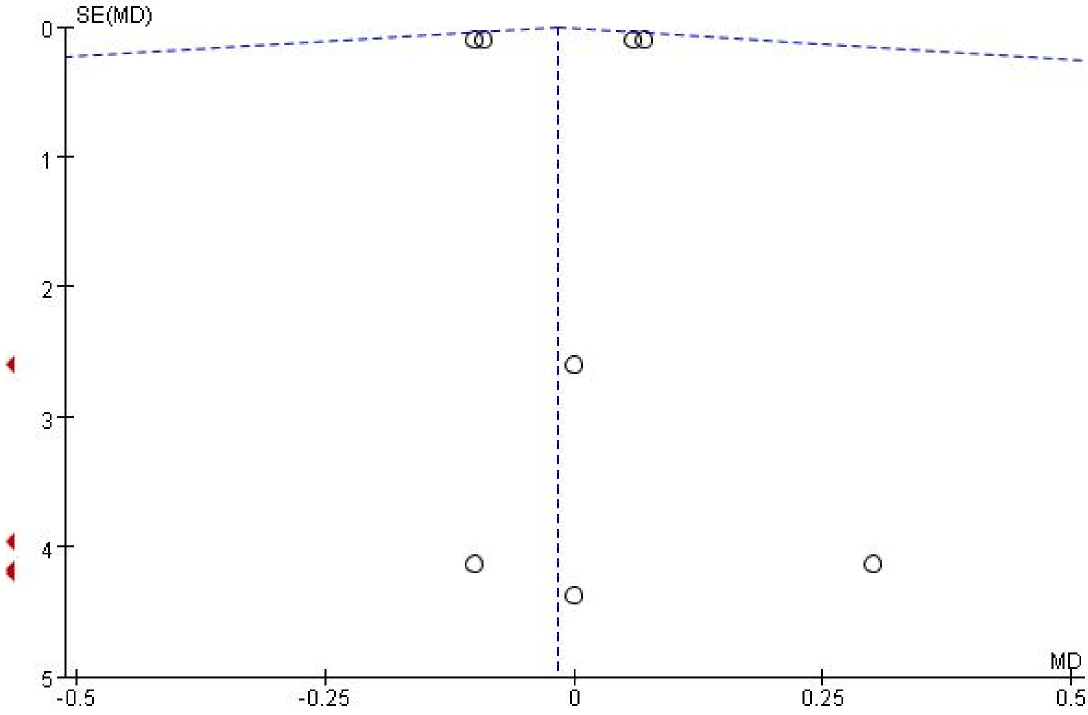

2.50 CD4/CD8(PPF)

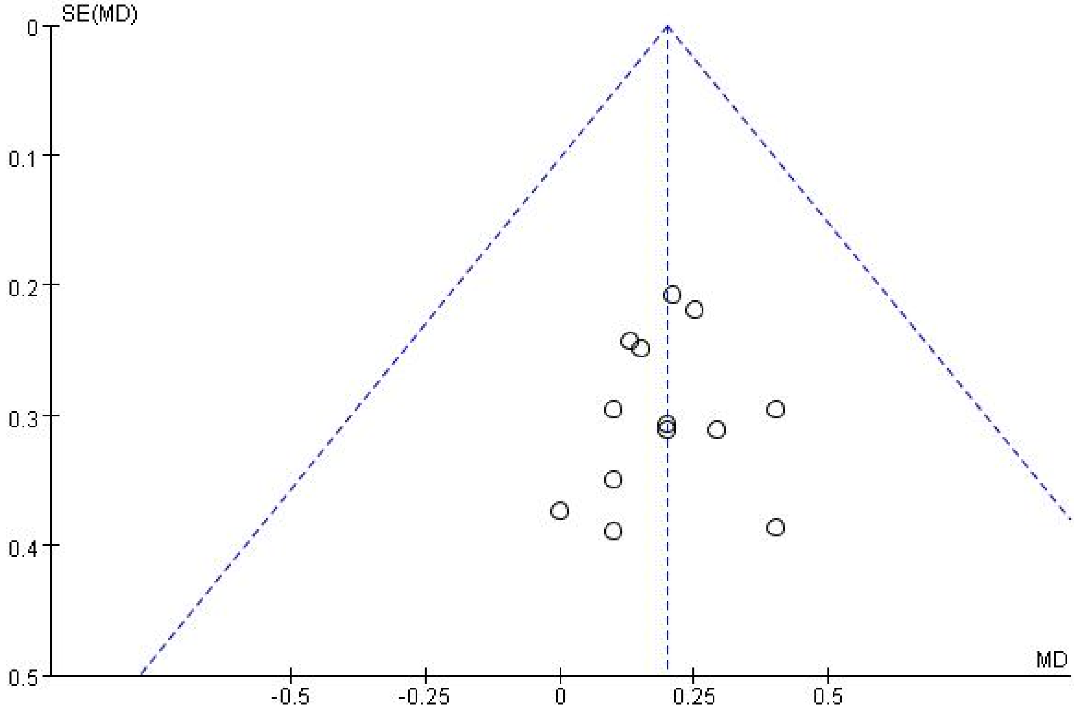

2.51 CD3(SEV)

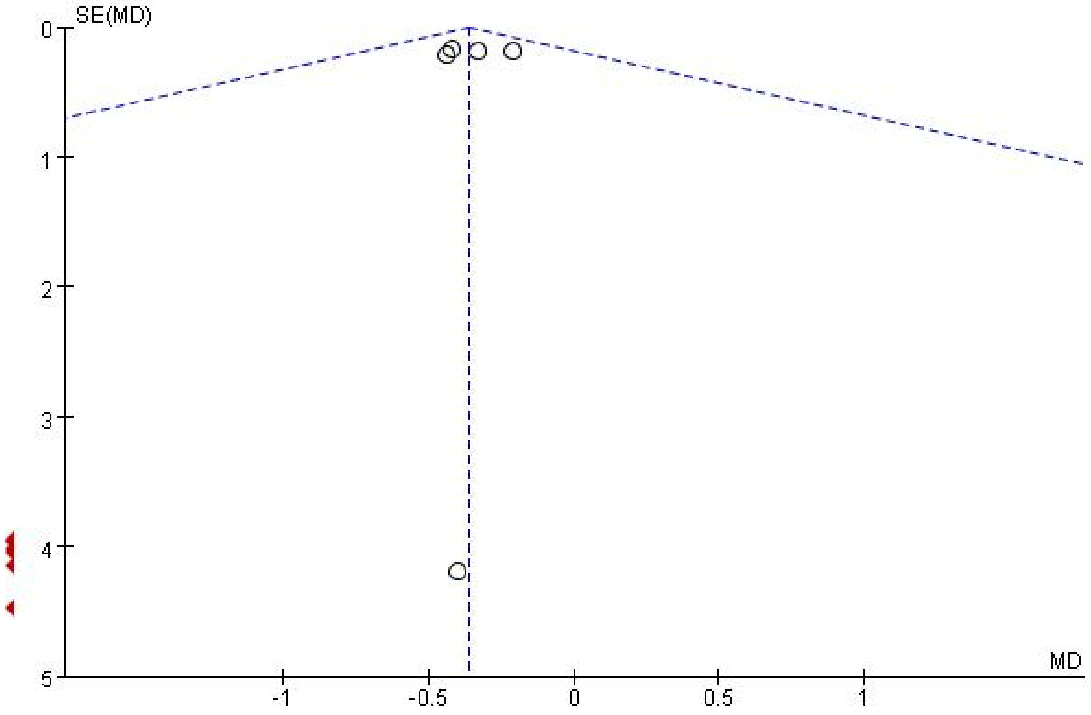

2.52 CD4(SEV)

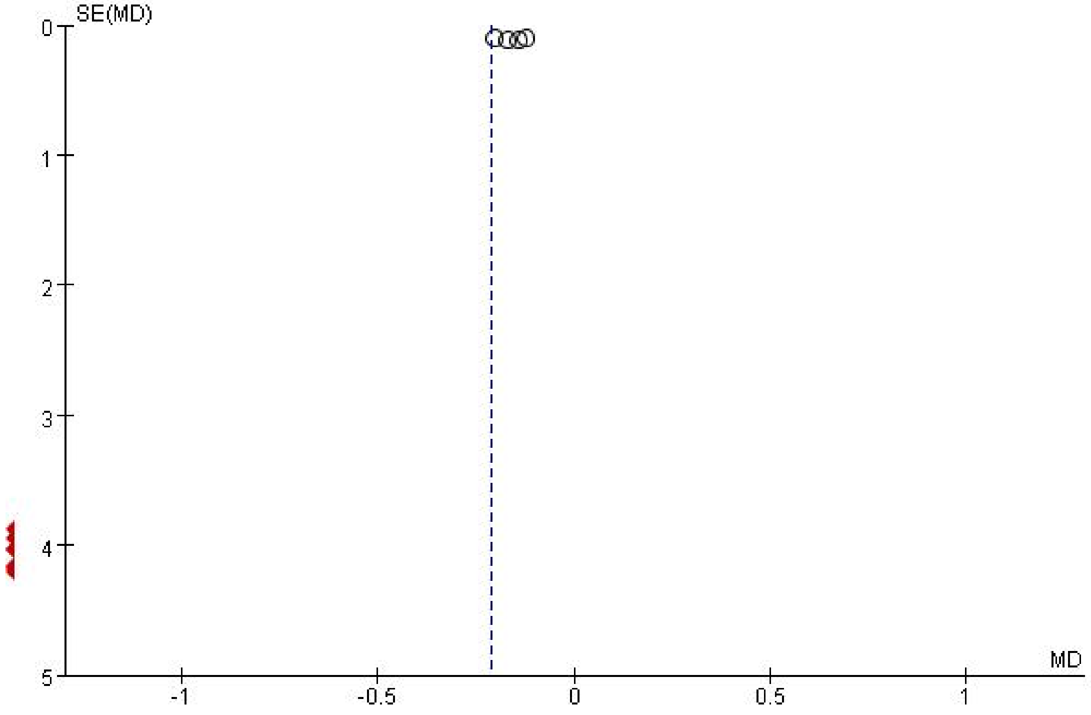

2.53 CD8(SEV)

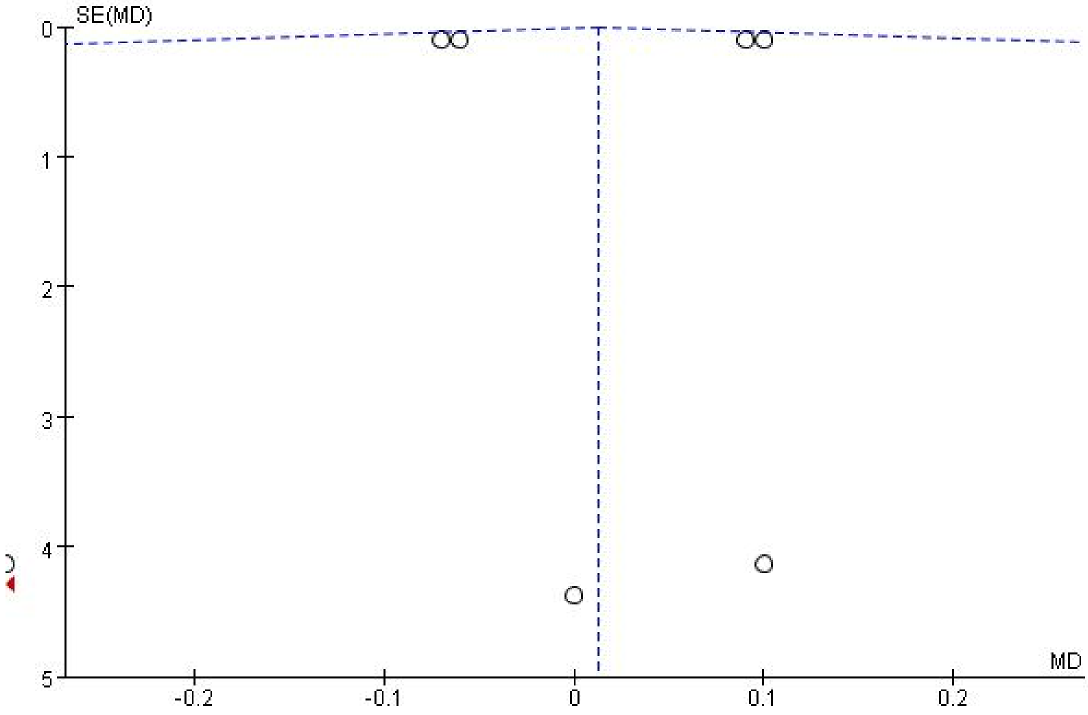

2.54 CD4/CD8(SEV)

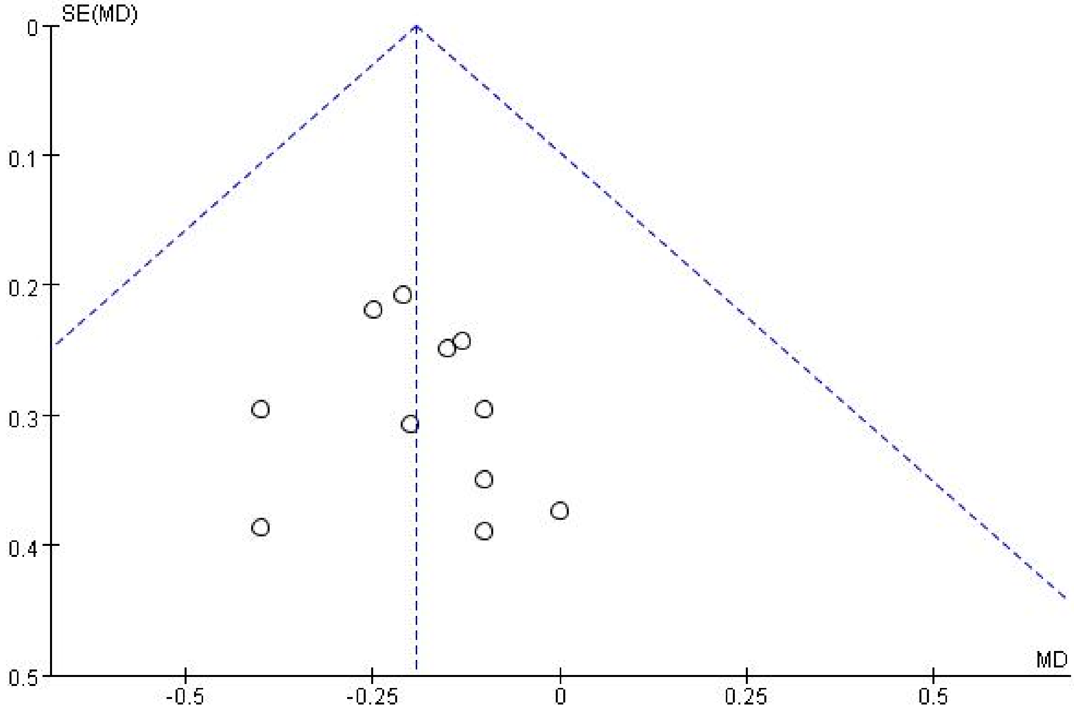

2.55CD3(Fentanyl)

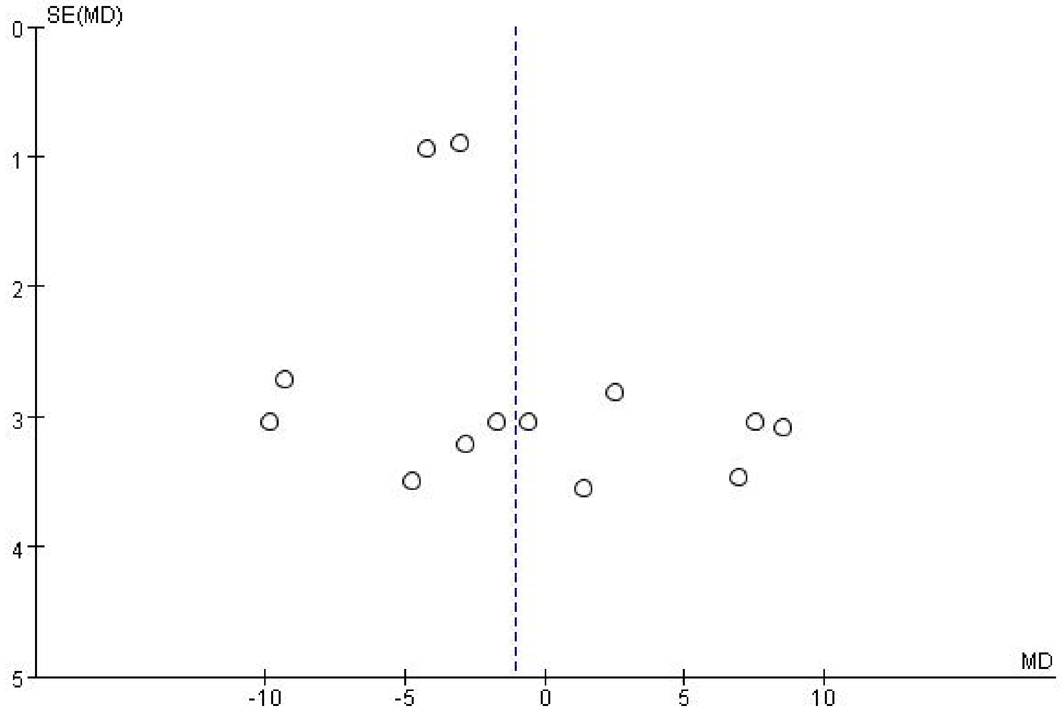

2.56 CD4(Fentanyl)

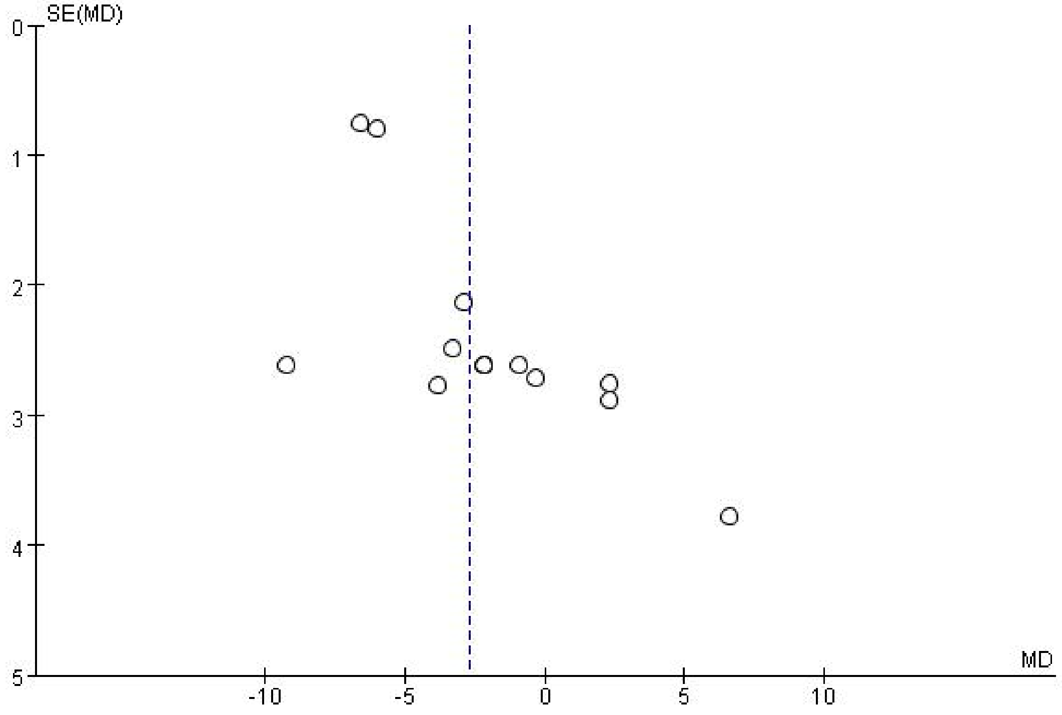

2.57 CD8(Fentanyl)

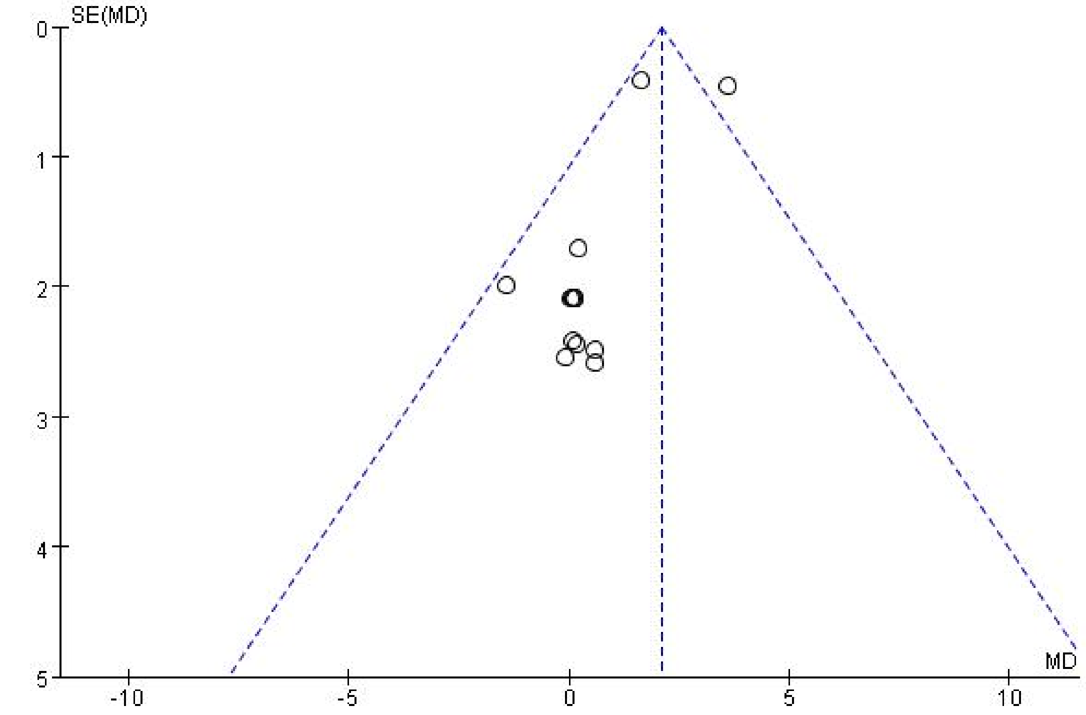

2.58 CD4/CD8(Fentanyl)

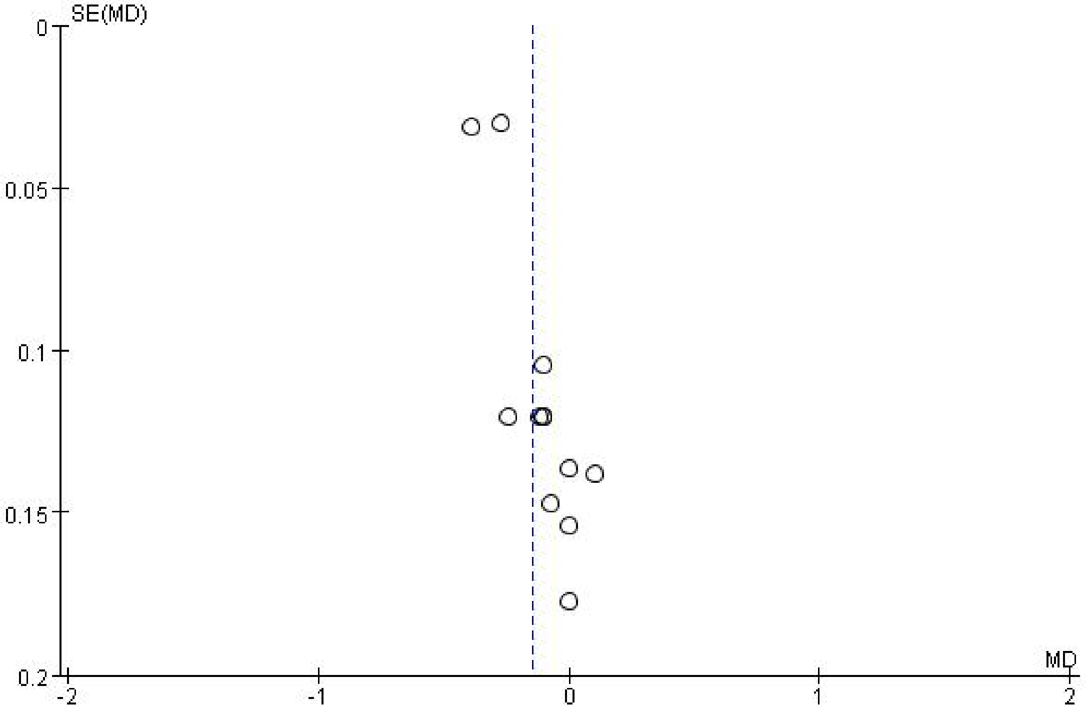

2.59 NK(Fentanyl)

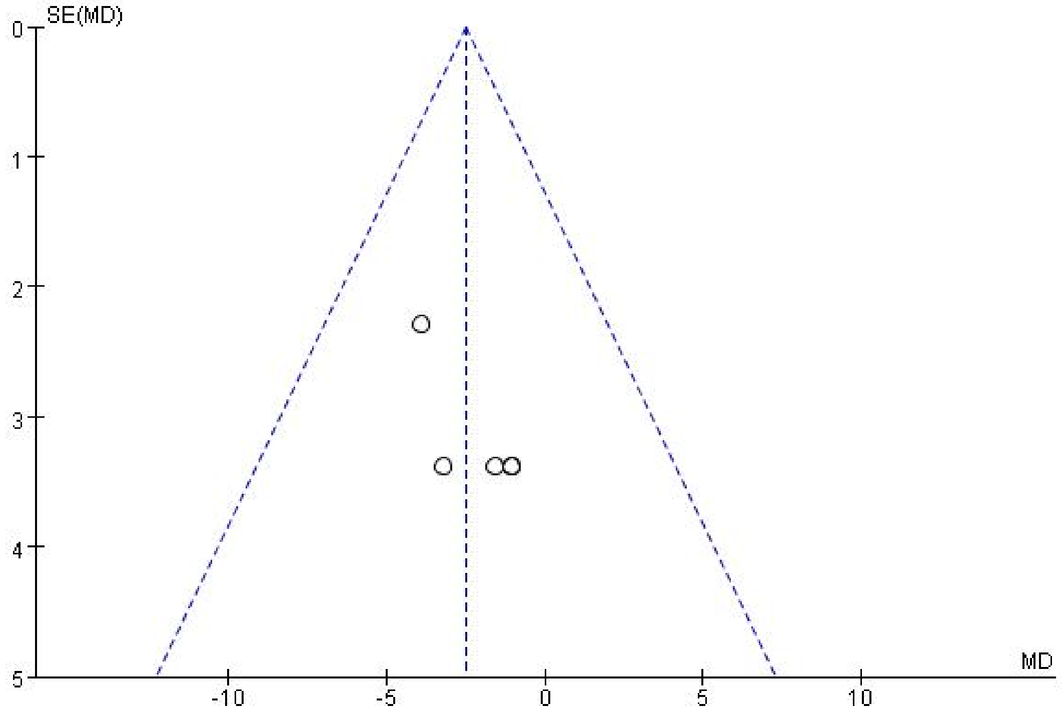

2.60 CD3(Flurbiprofen)

CD3

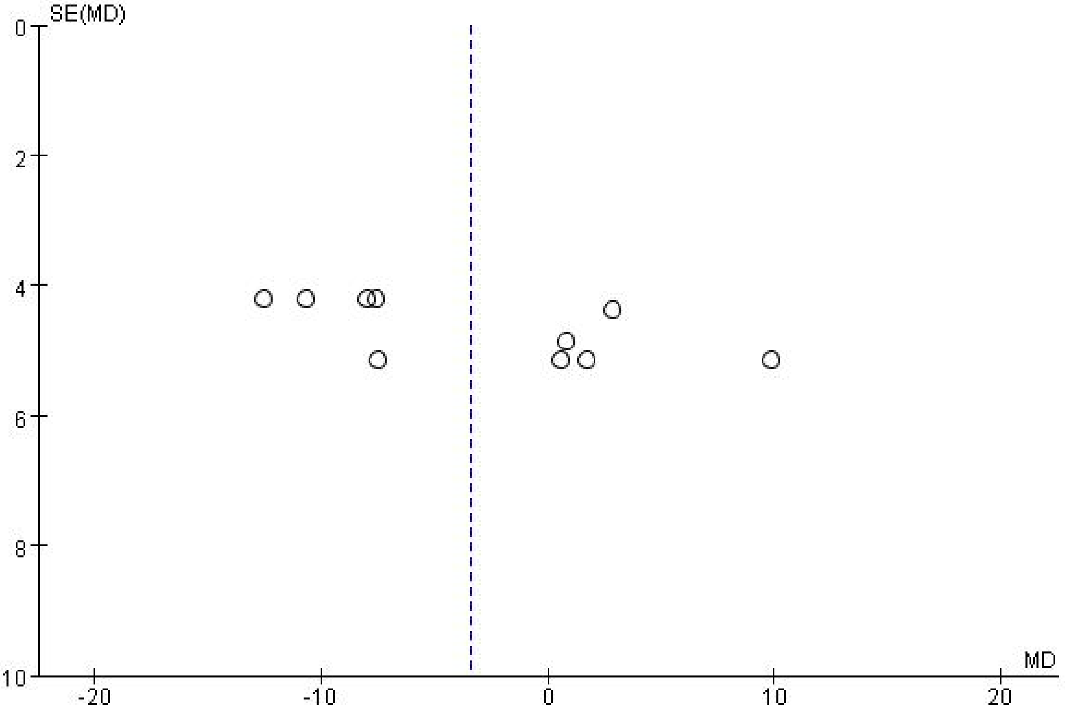

2.61 CD4(Flurbiprofen)

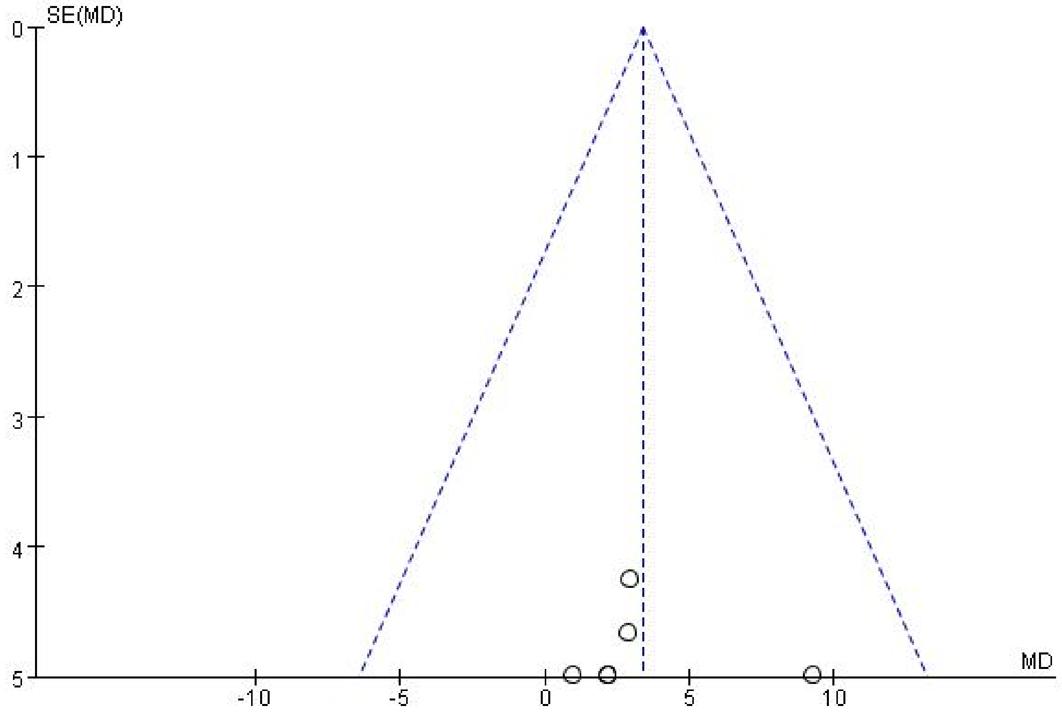

2.62 CD8(Flurbiprofen)

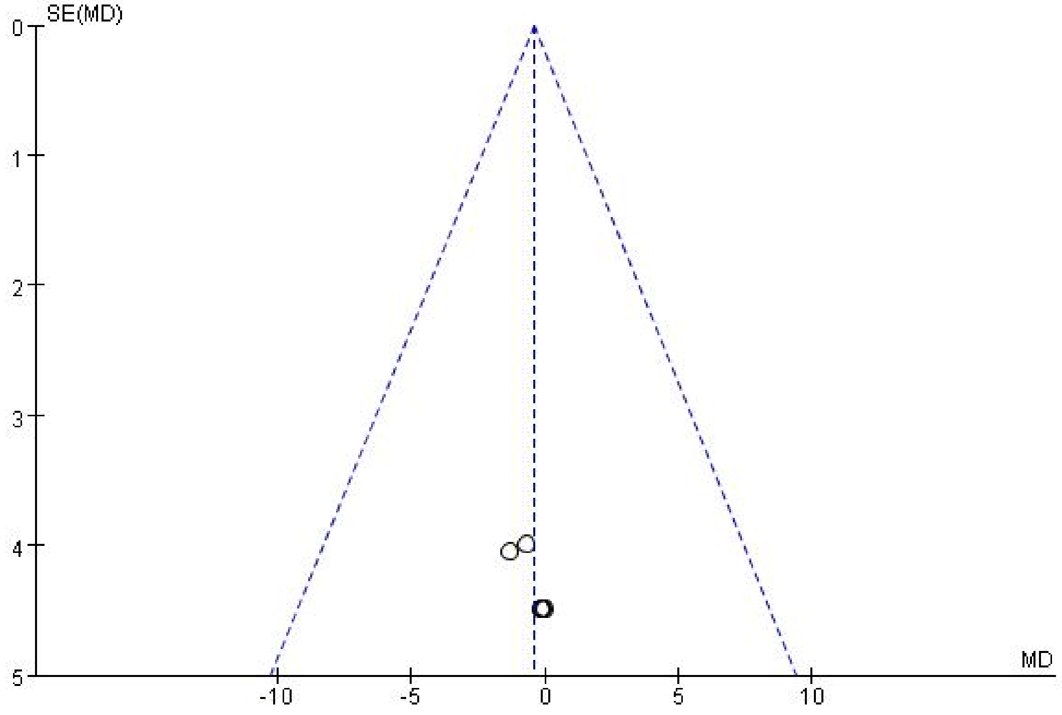

2.63 CD4/CD8(Flurbiprofen)

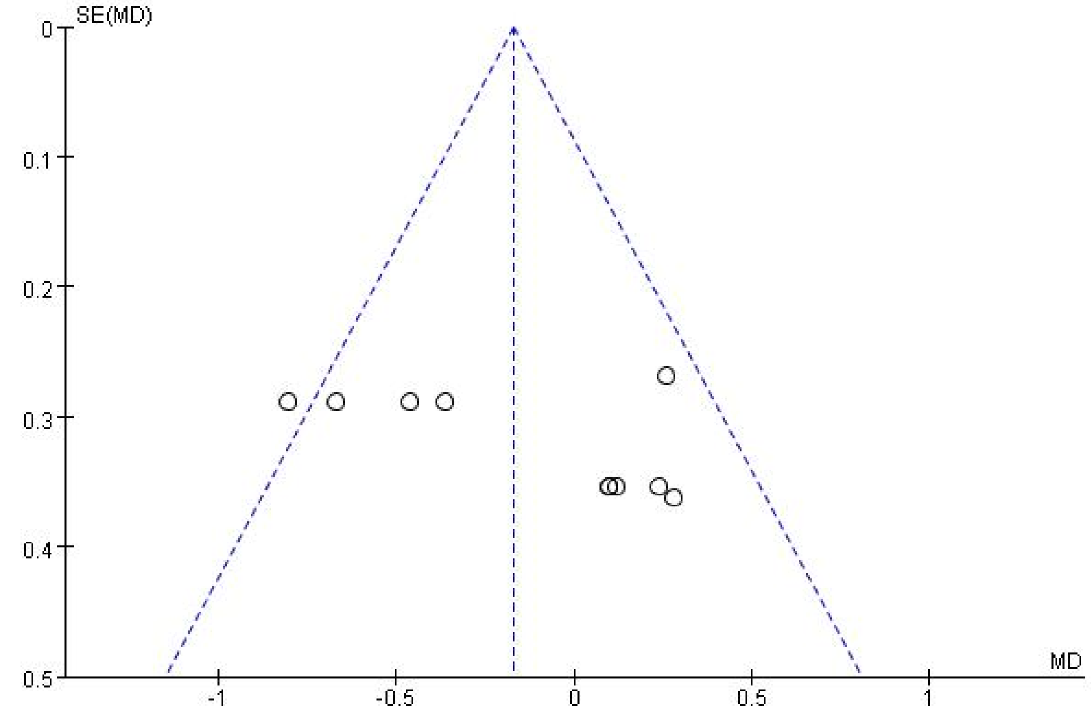

2.64 CD3(Parecoxib)

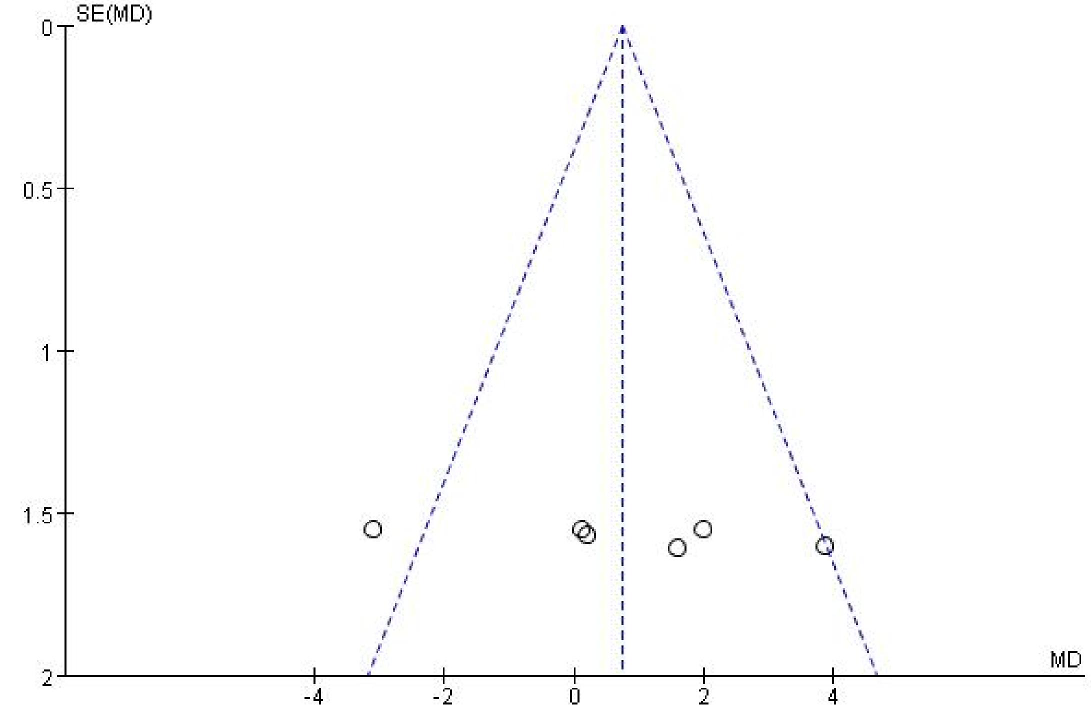

2.65 CD4(Parecoxib)

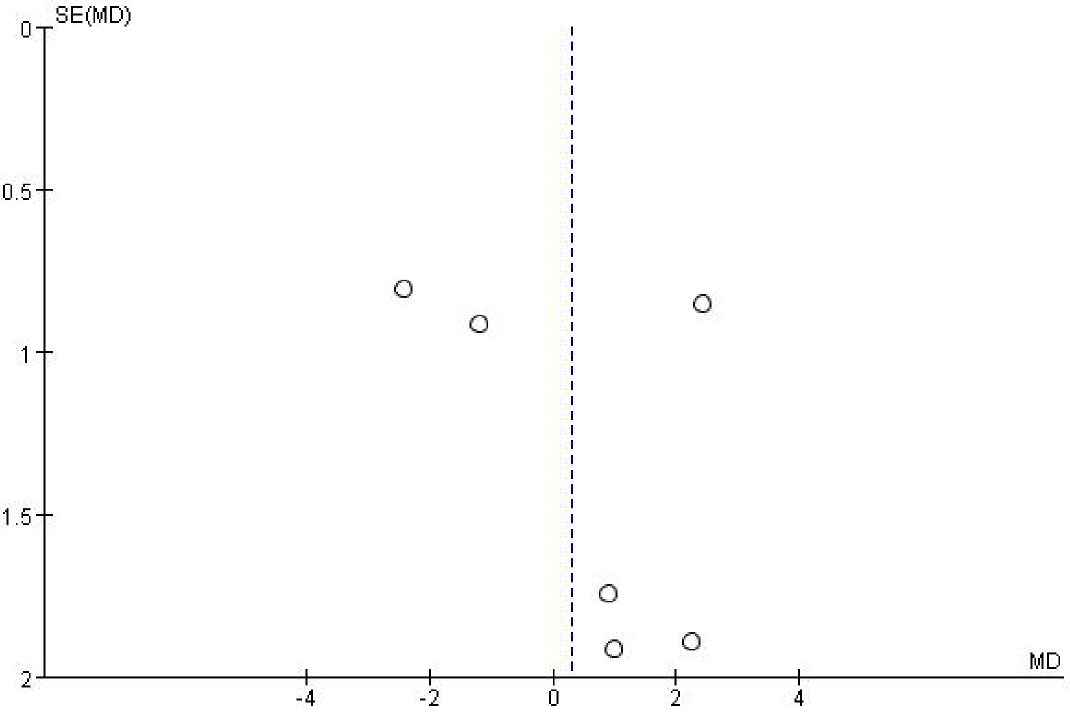

2.66 CD8(Parecoxib)

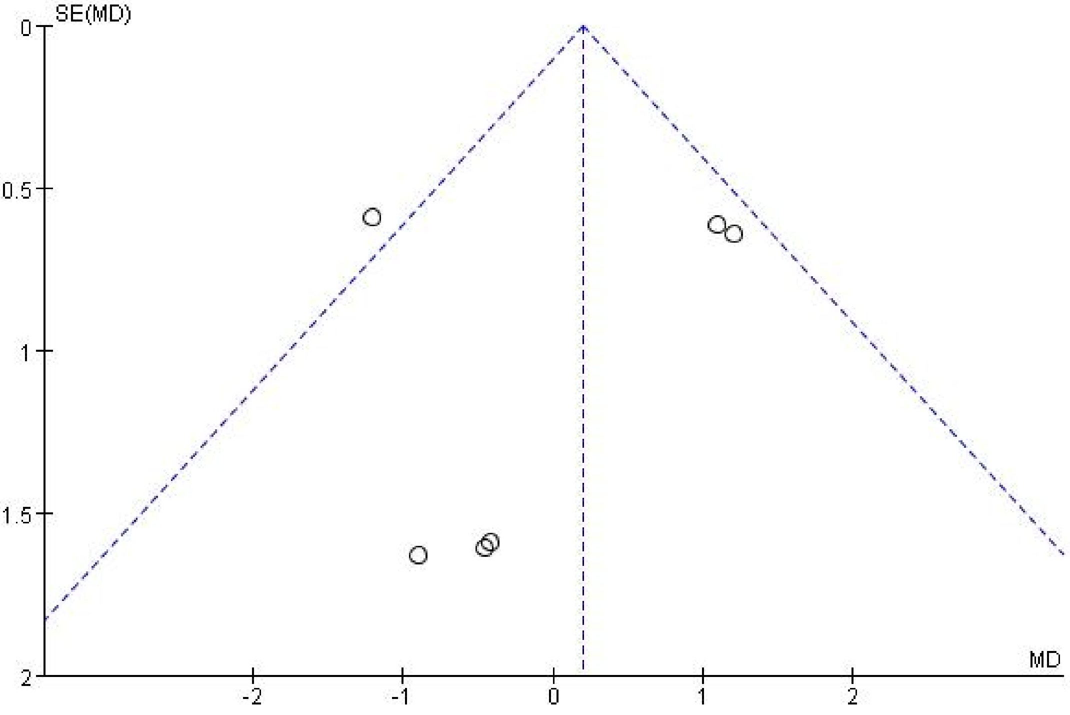

2.67 CD4/CD8(Parecoxib)

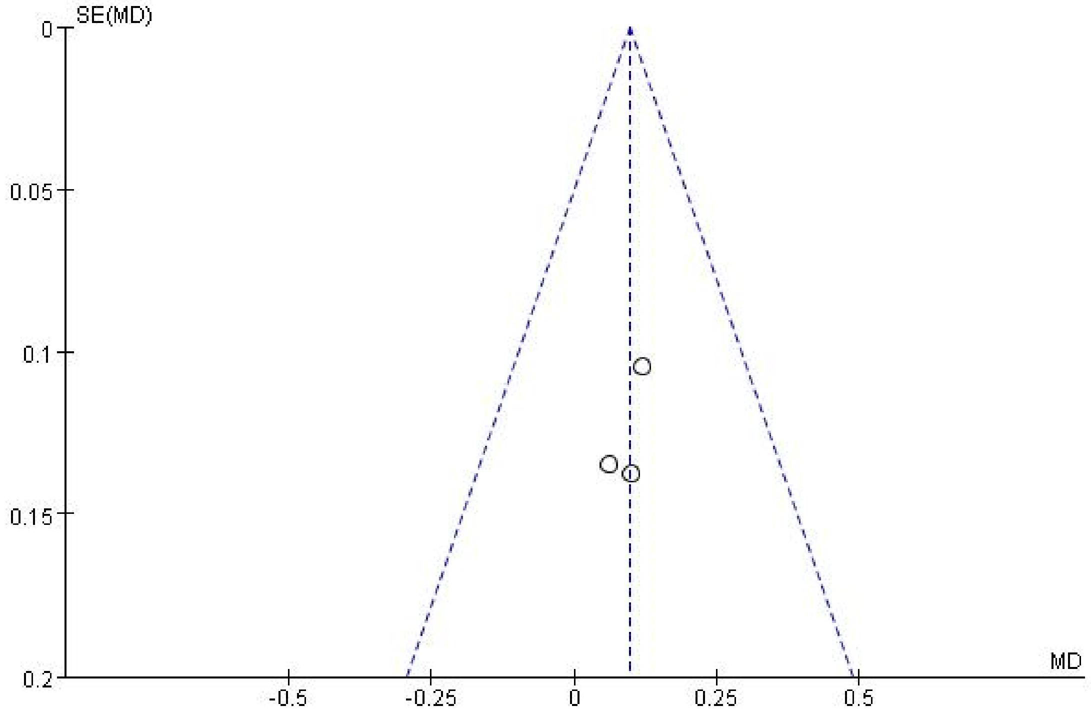

2.68 NK(Parecoxib)

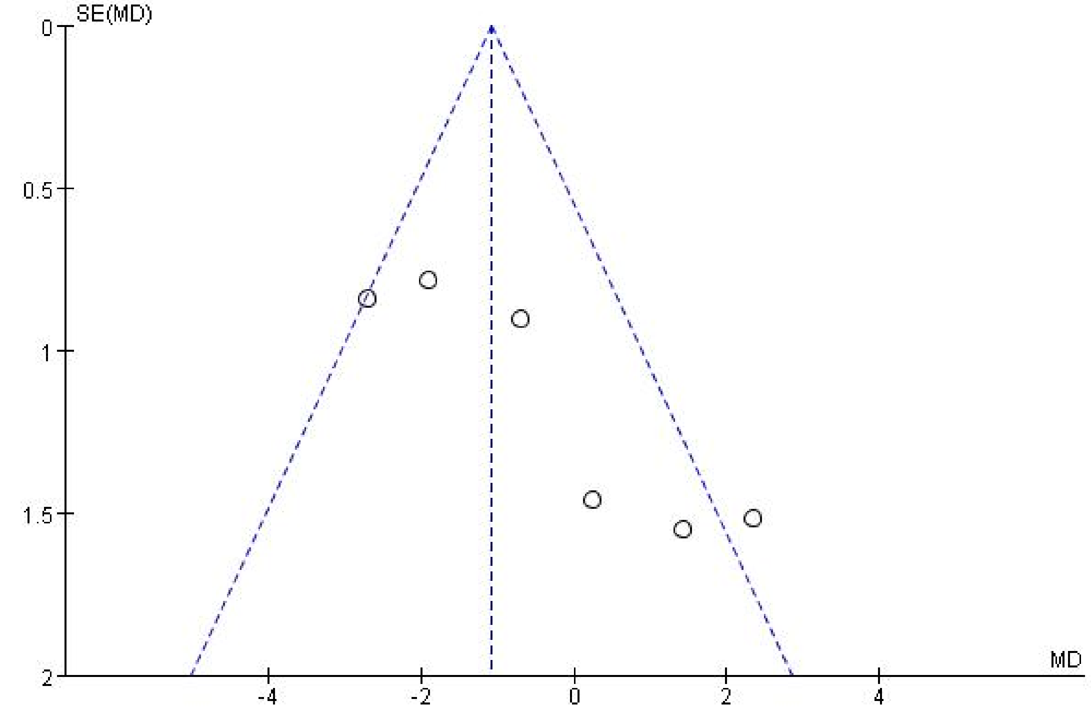

2.69 CD3(Morphine)

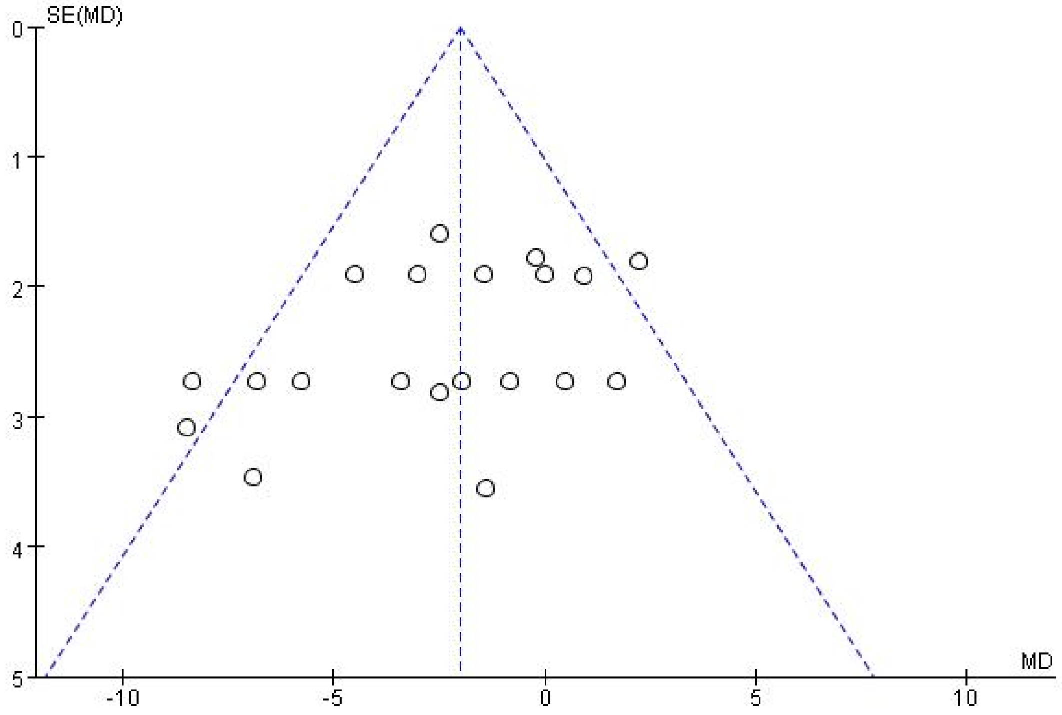

2.70 CD4(Morphine)

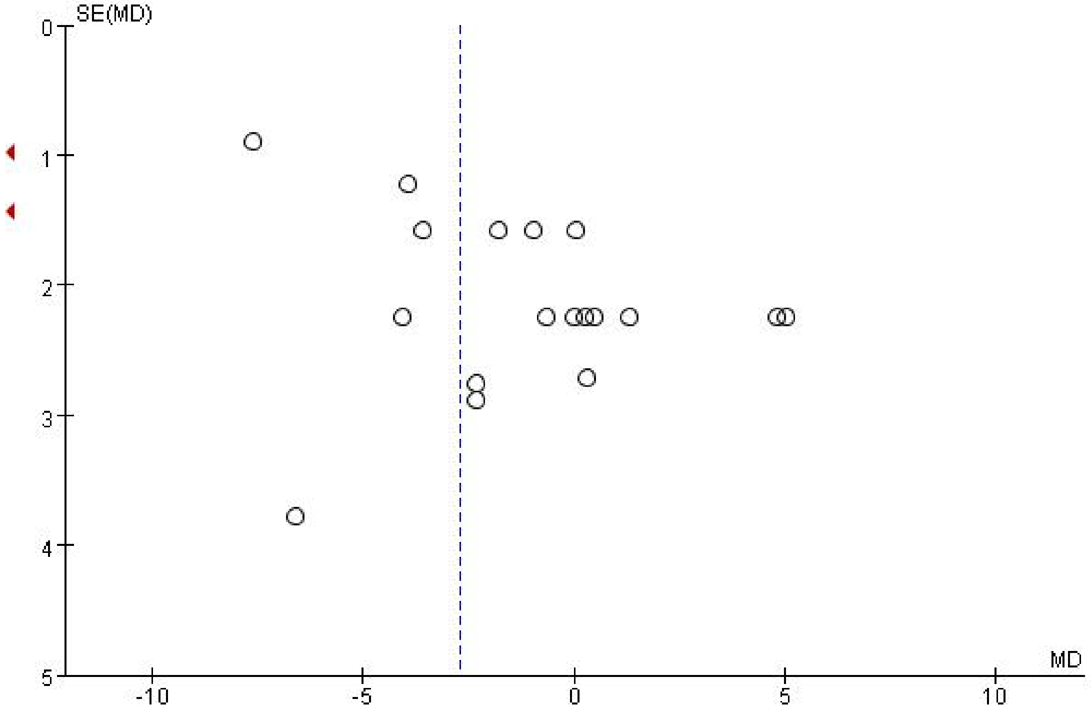

2.71 CD8(Morphine)

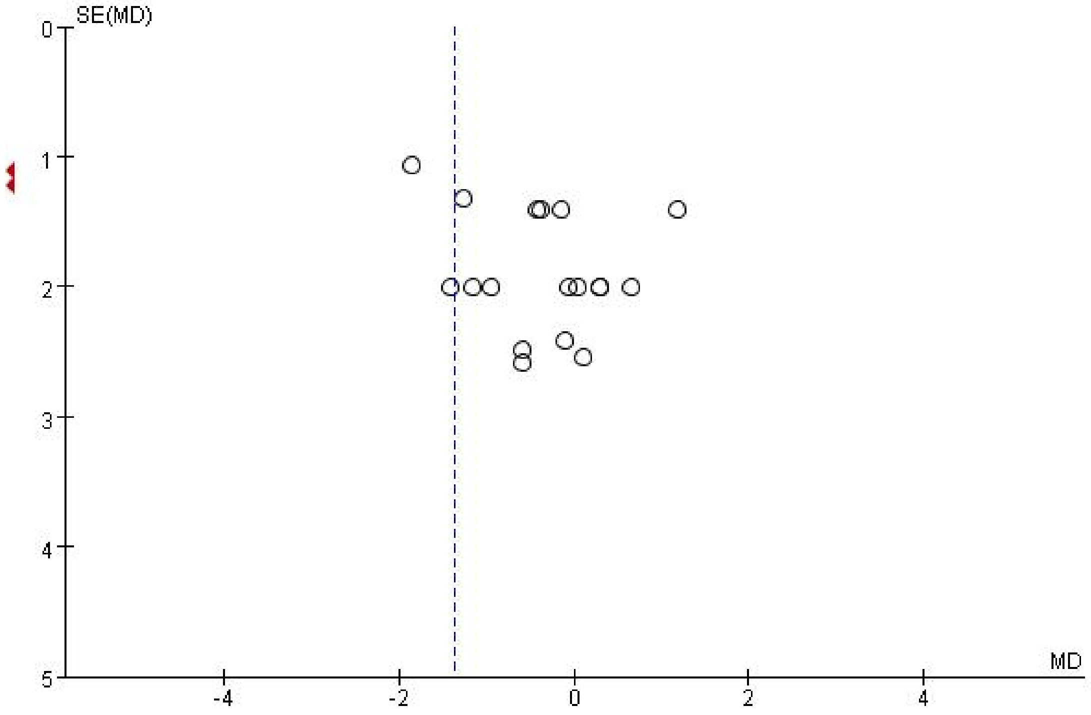

2.72 CD4/CD8(Morphine)

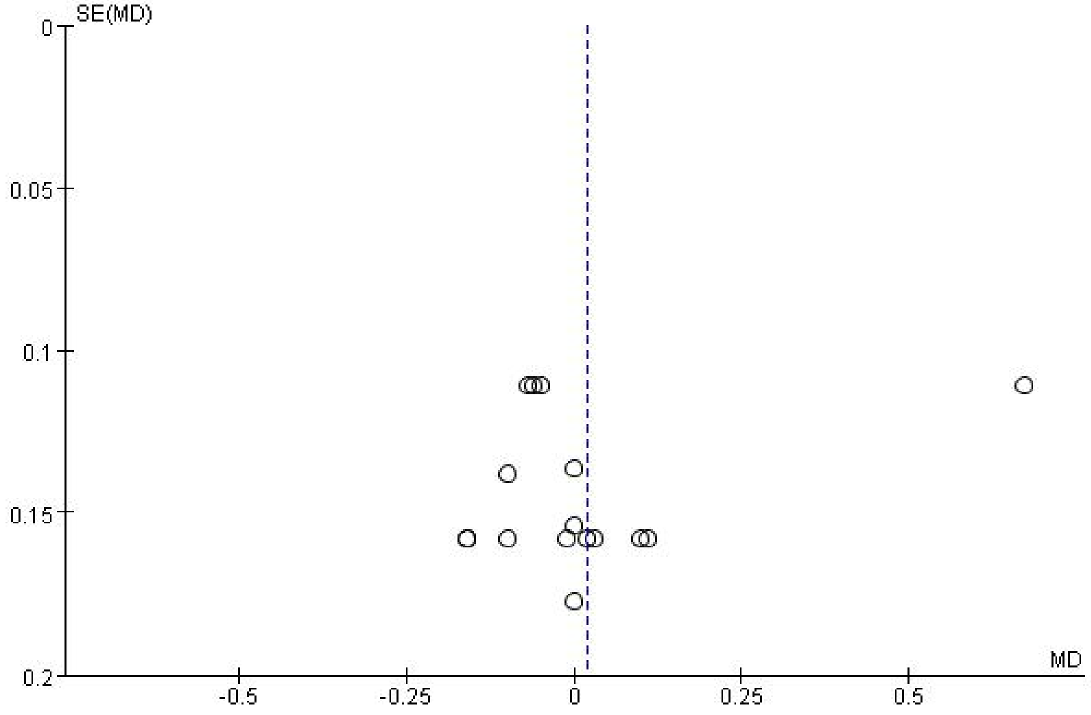

2.73 CD3(Dezocine)

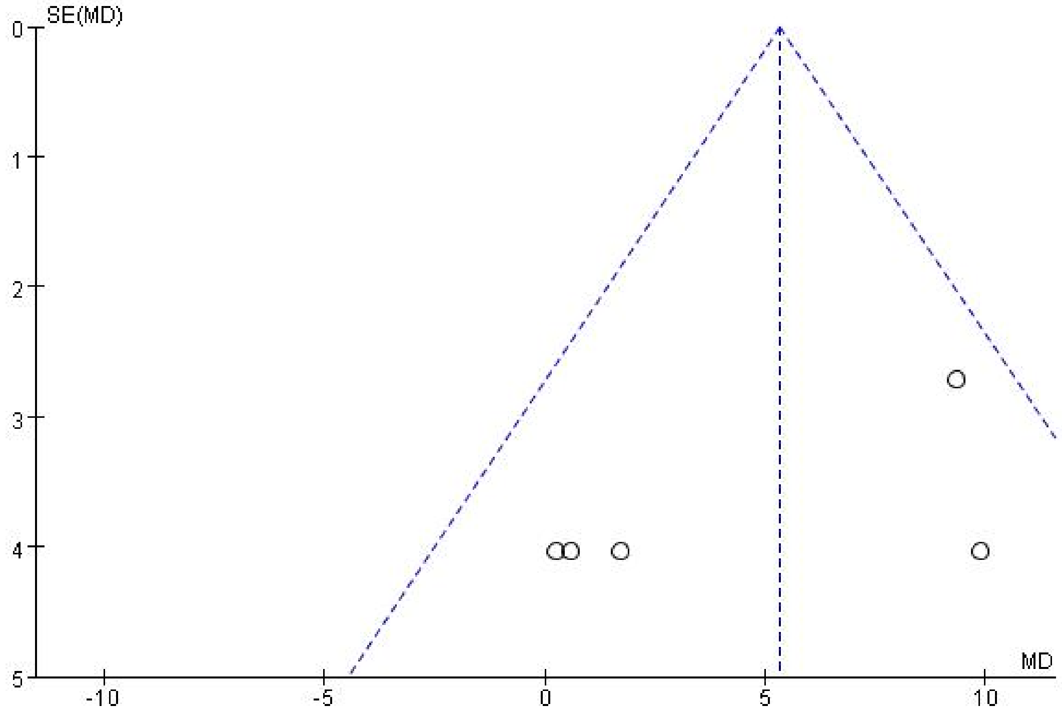

2.74 CD4(Dezocine)

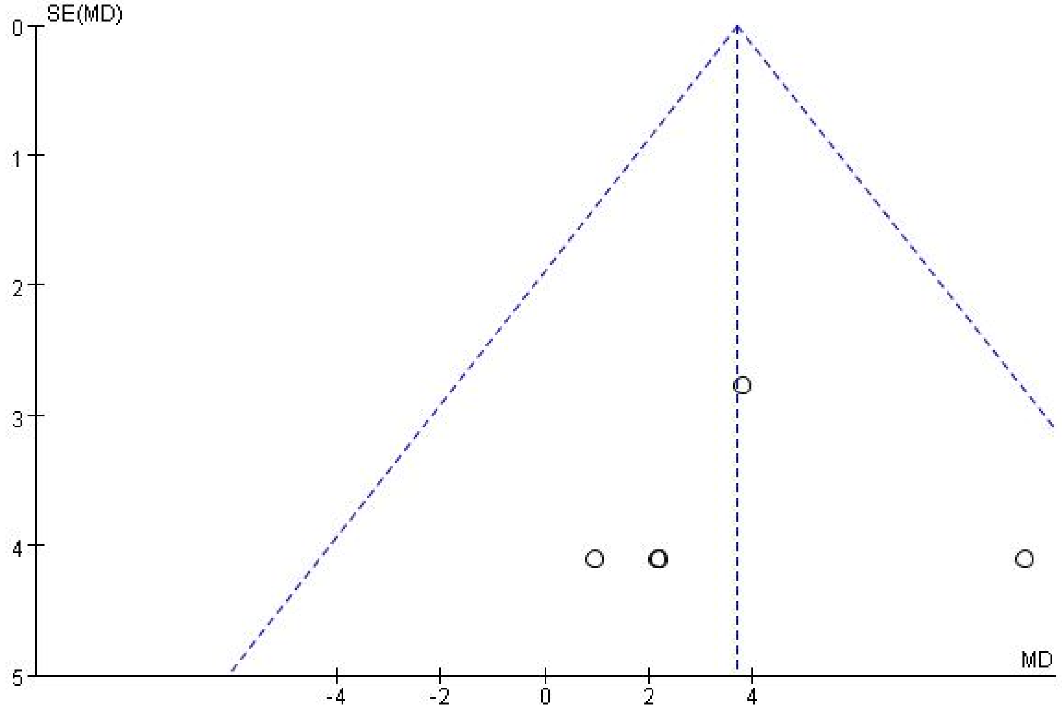

2.75 CD8(Dezocine)

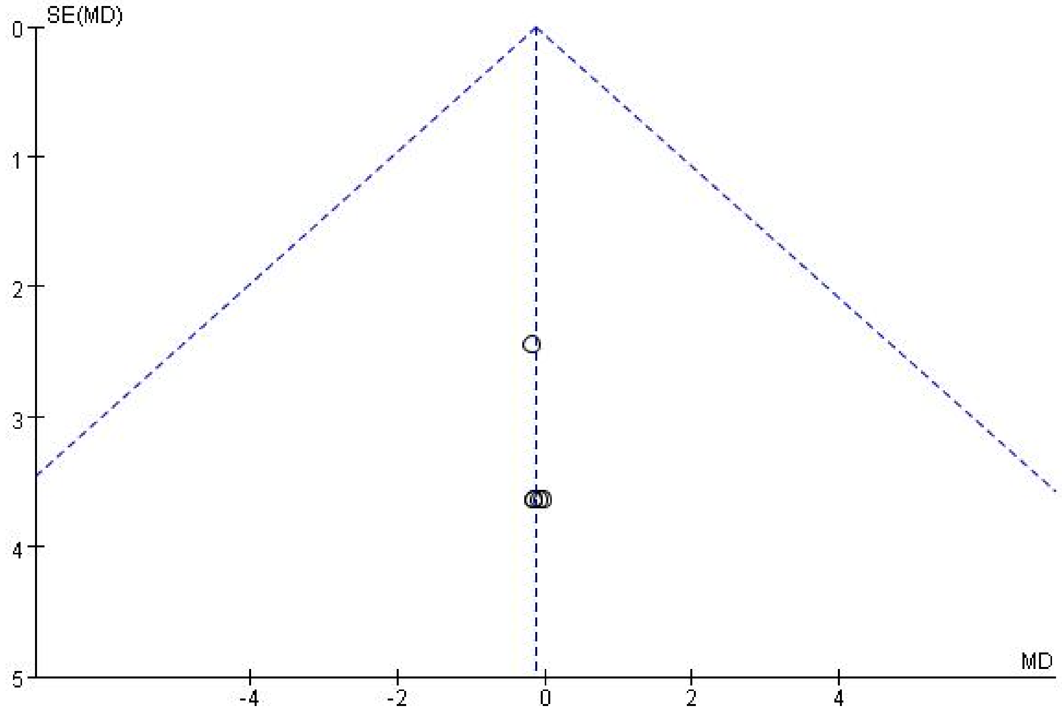

2.76NK(Dezocine)

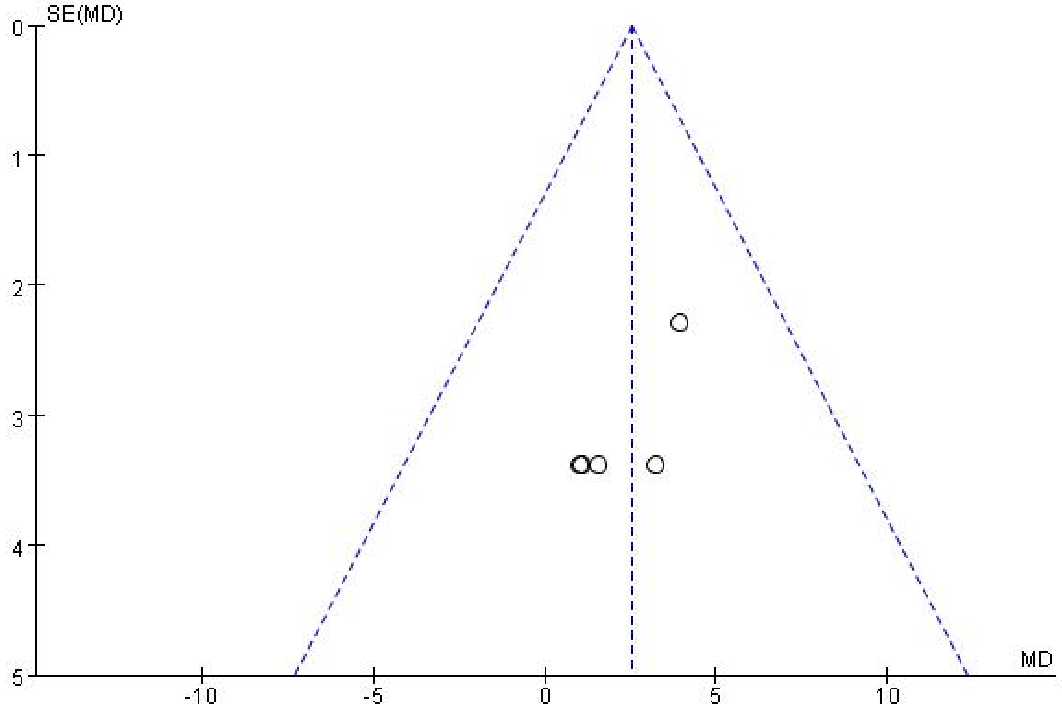

2.77 CD3(Tramadol)

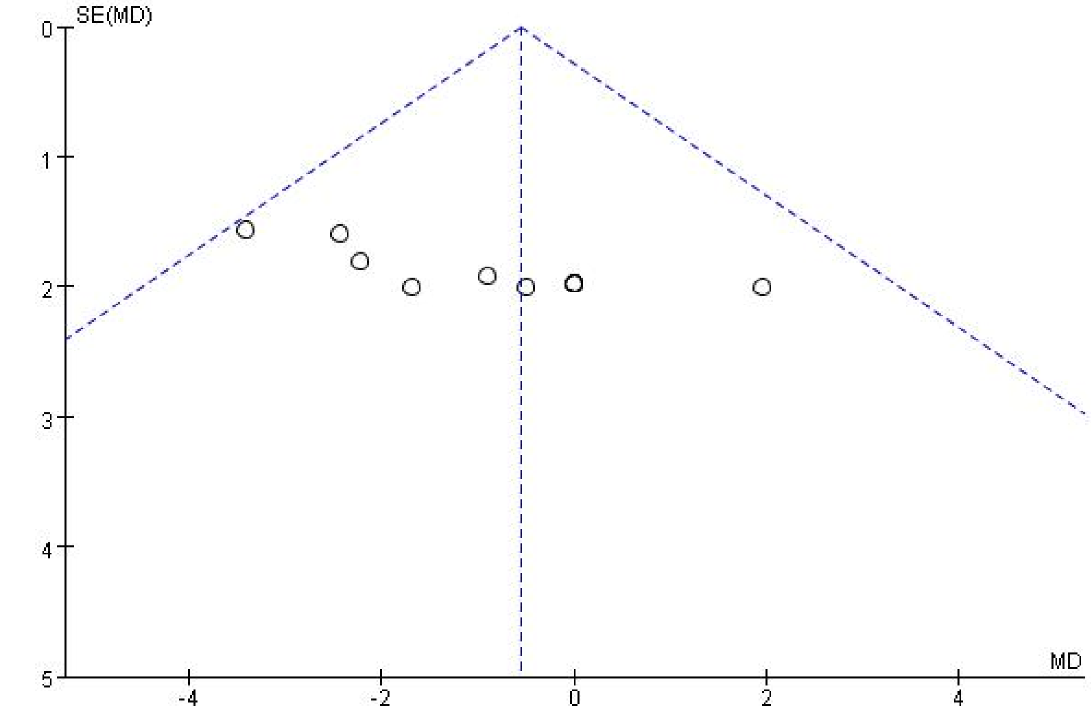

2.78 CD4(Tramadol)

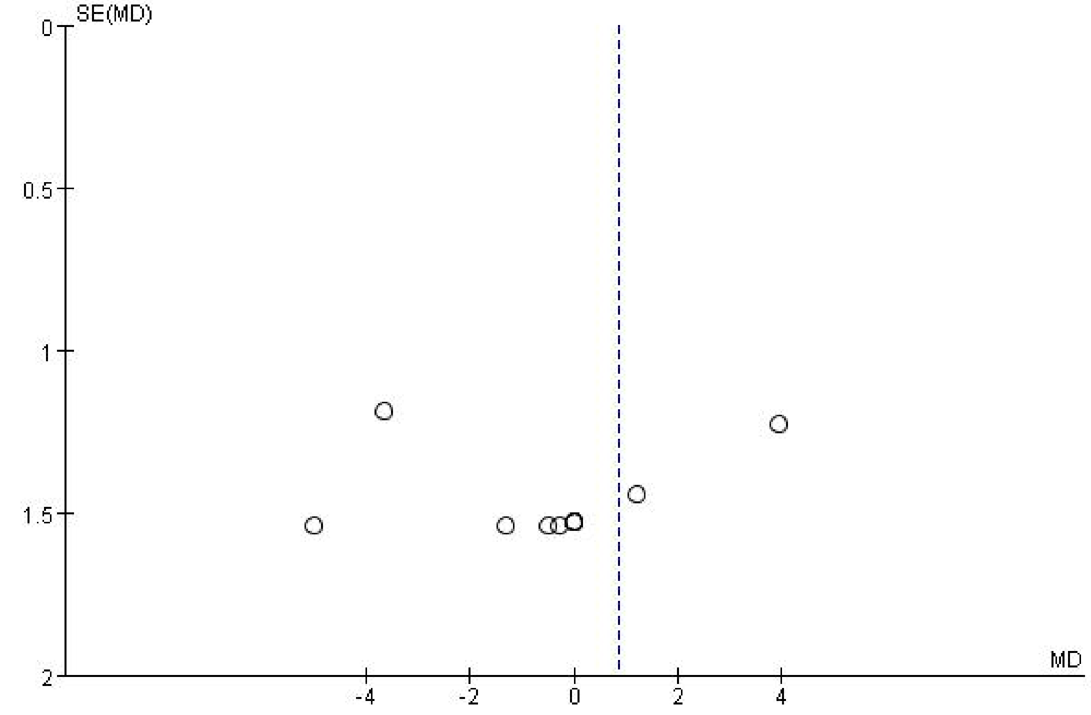

2.79 CD8(Tramadol)

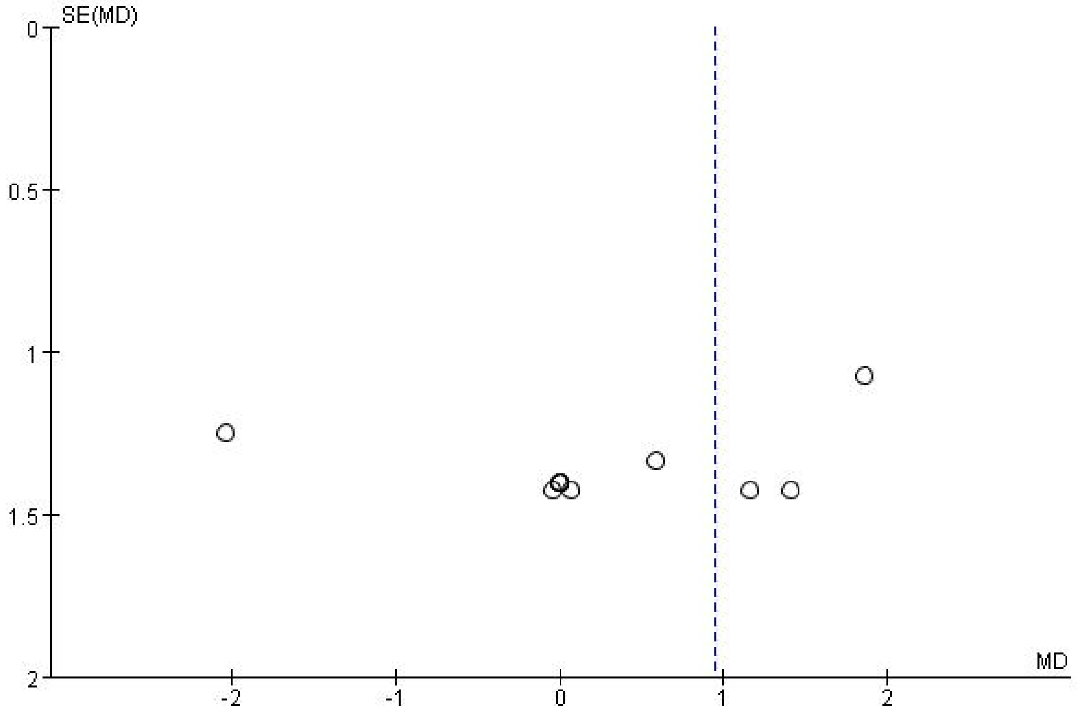

2.80 CD3(EN)

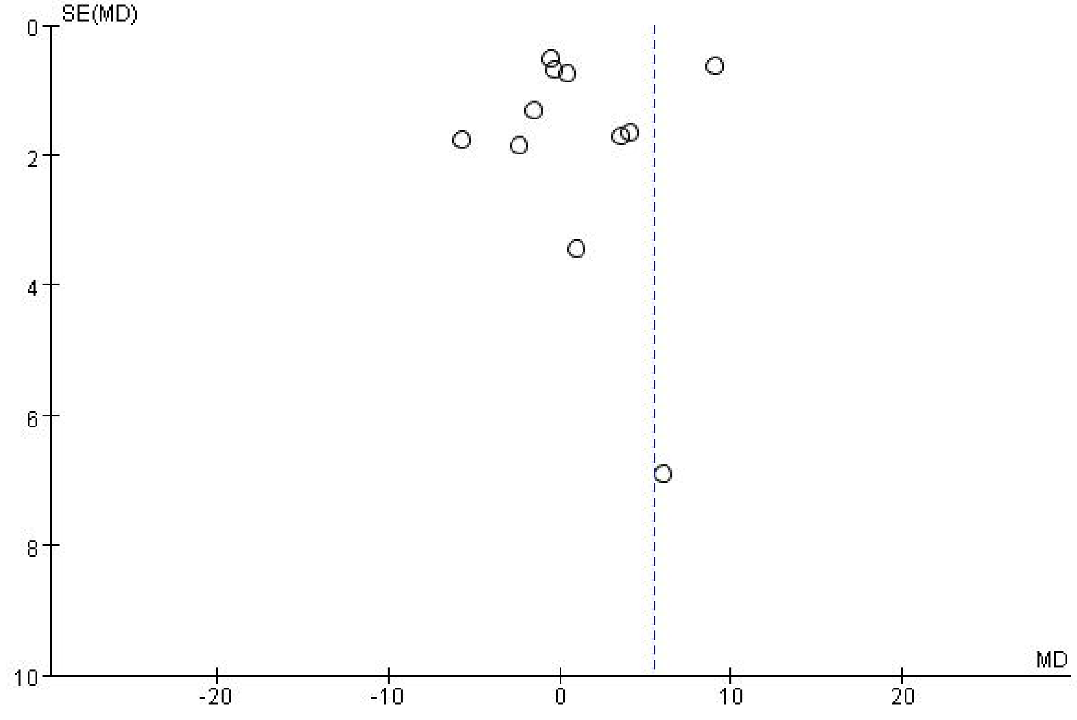

2.81 CD4(EN)

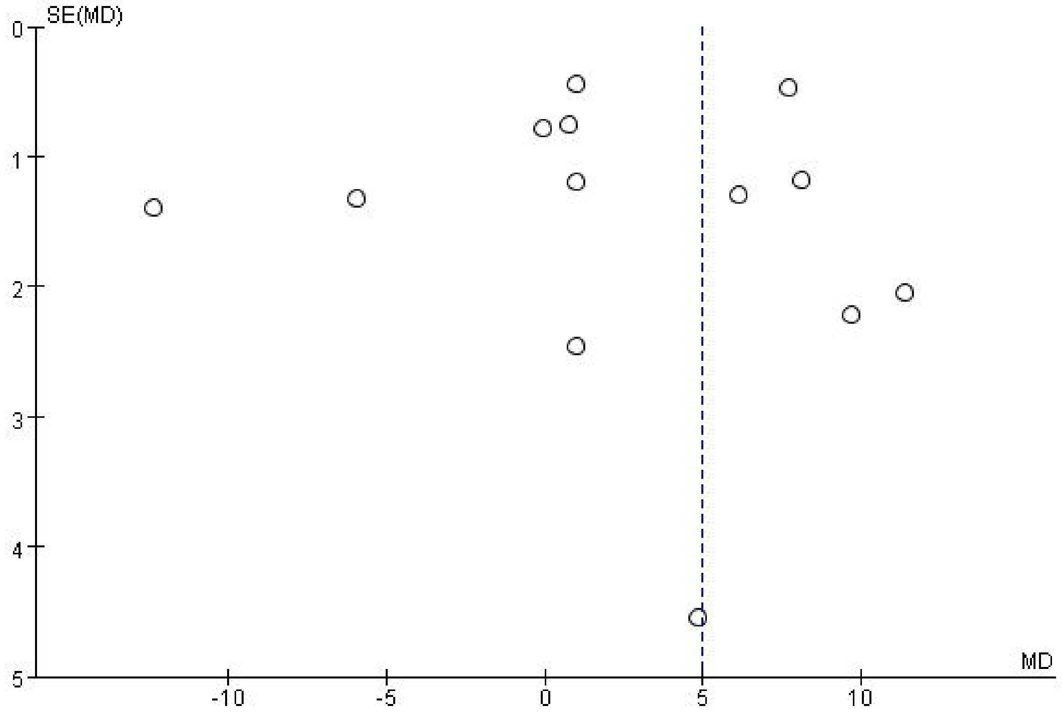

2.82 CD8(EN)

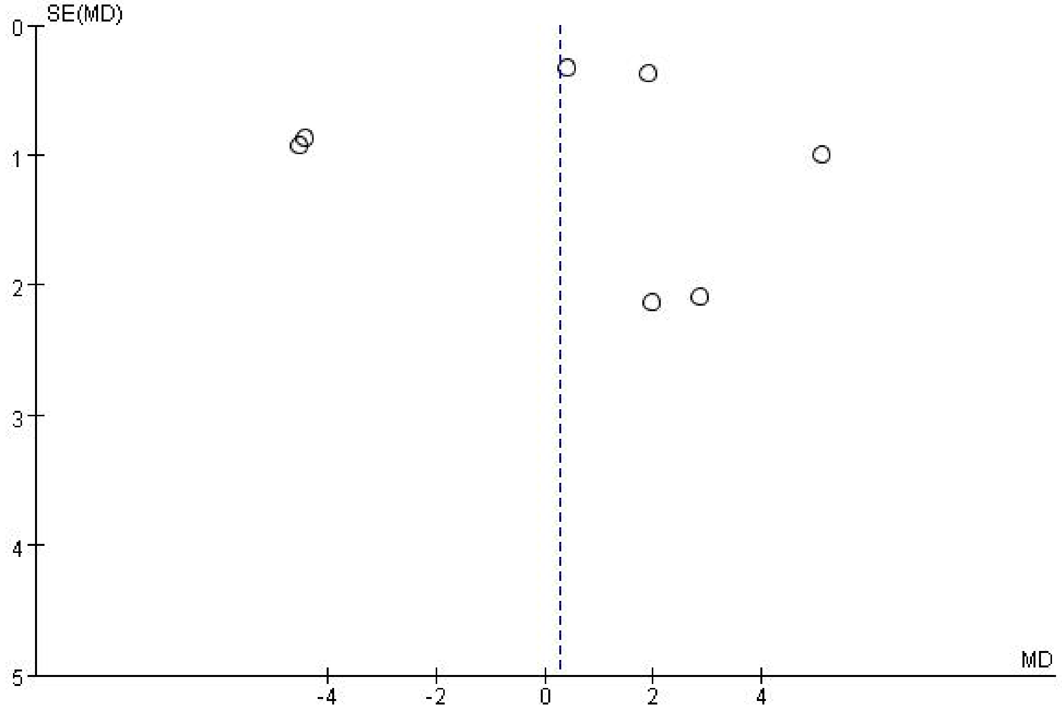

2.83 CD4/CD8(EN)

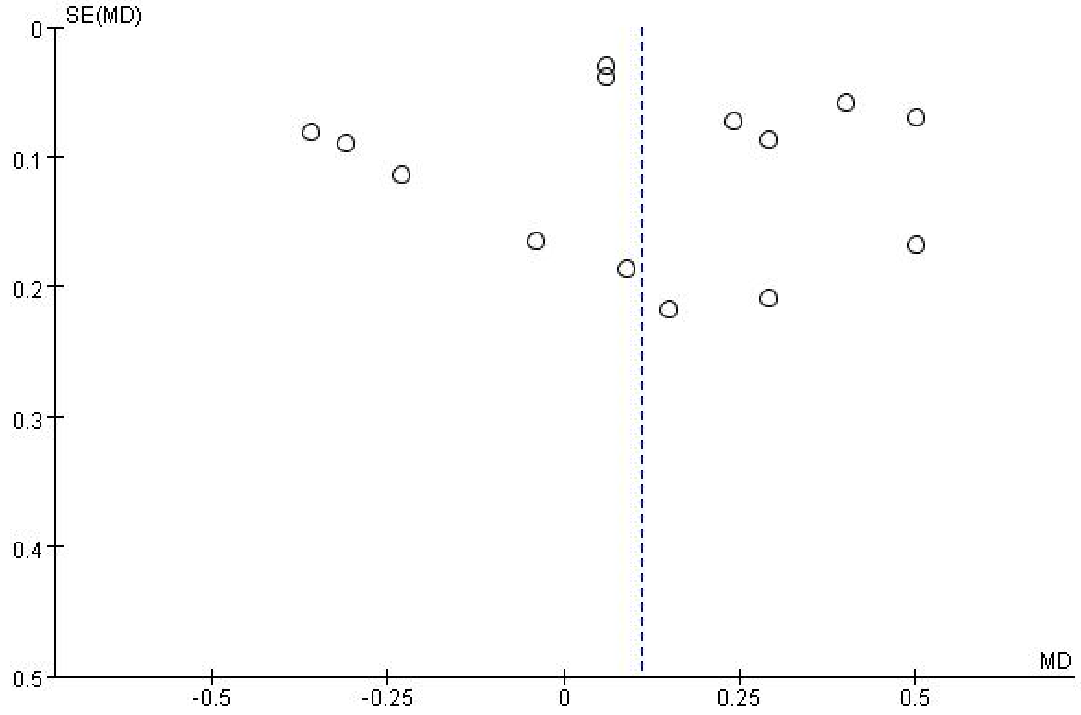

2.84 NK(EN)

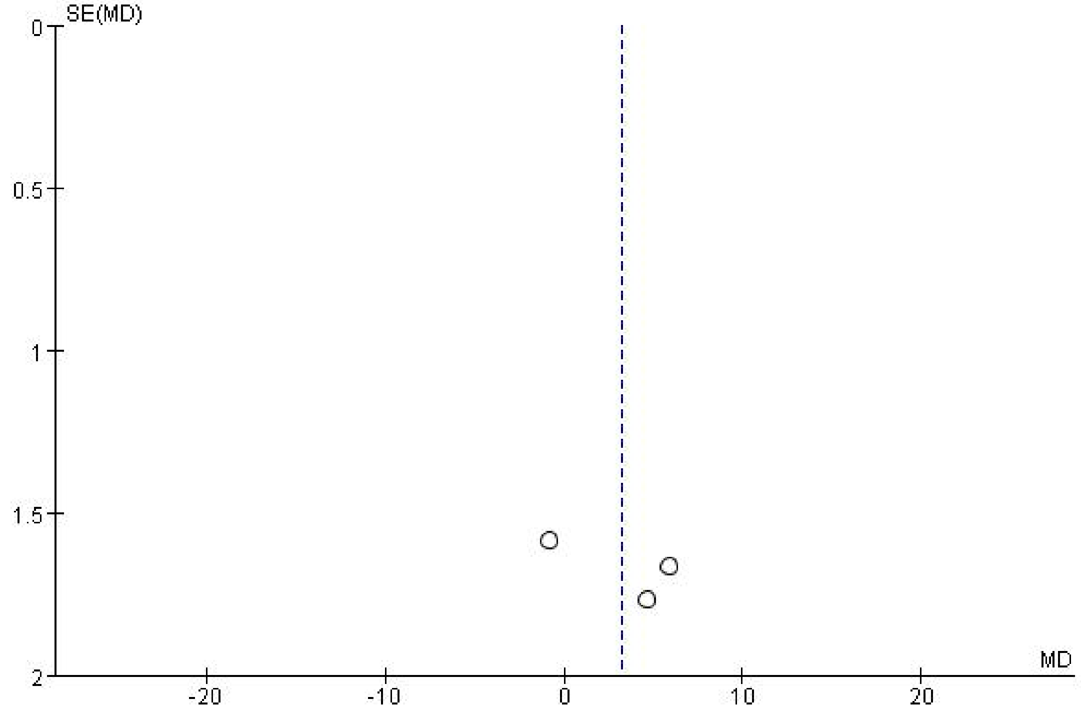

2.85 CD3(EEN)

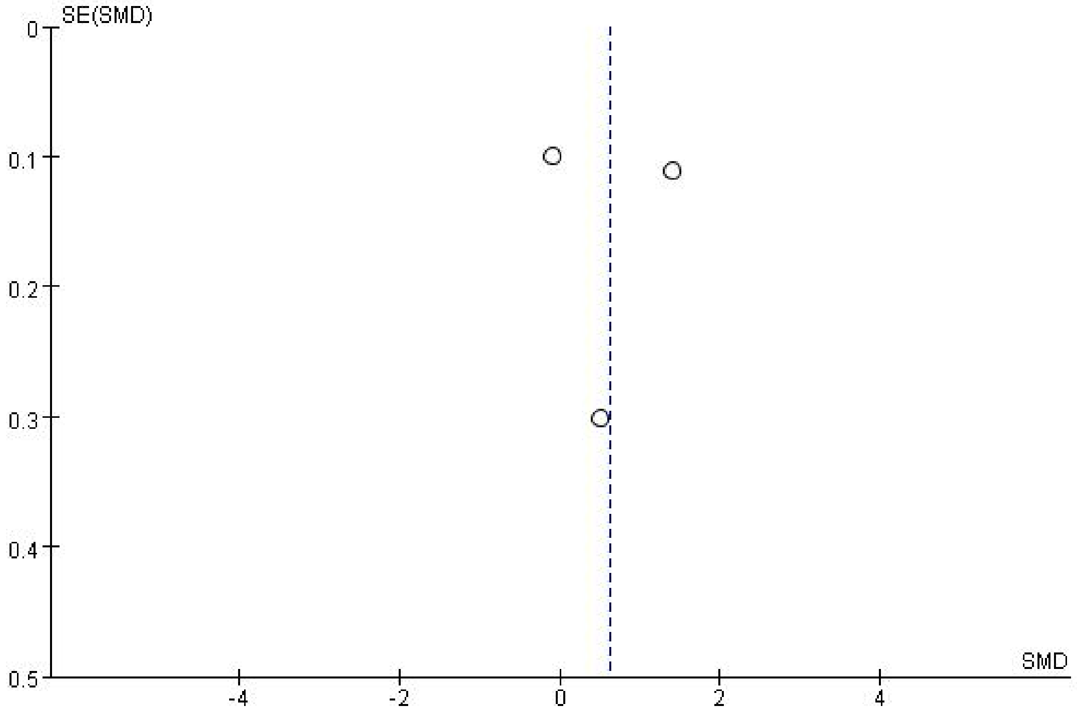

2.86 CD4(EEN)

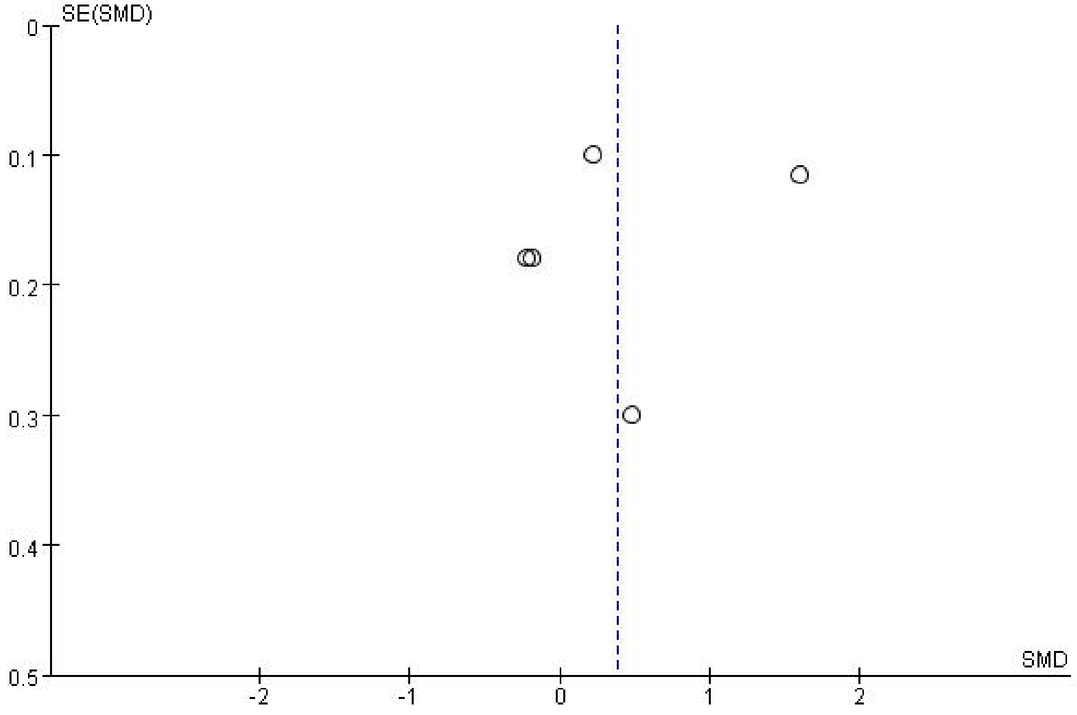

2.87 CD8 (EEN)

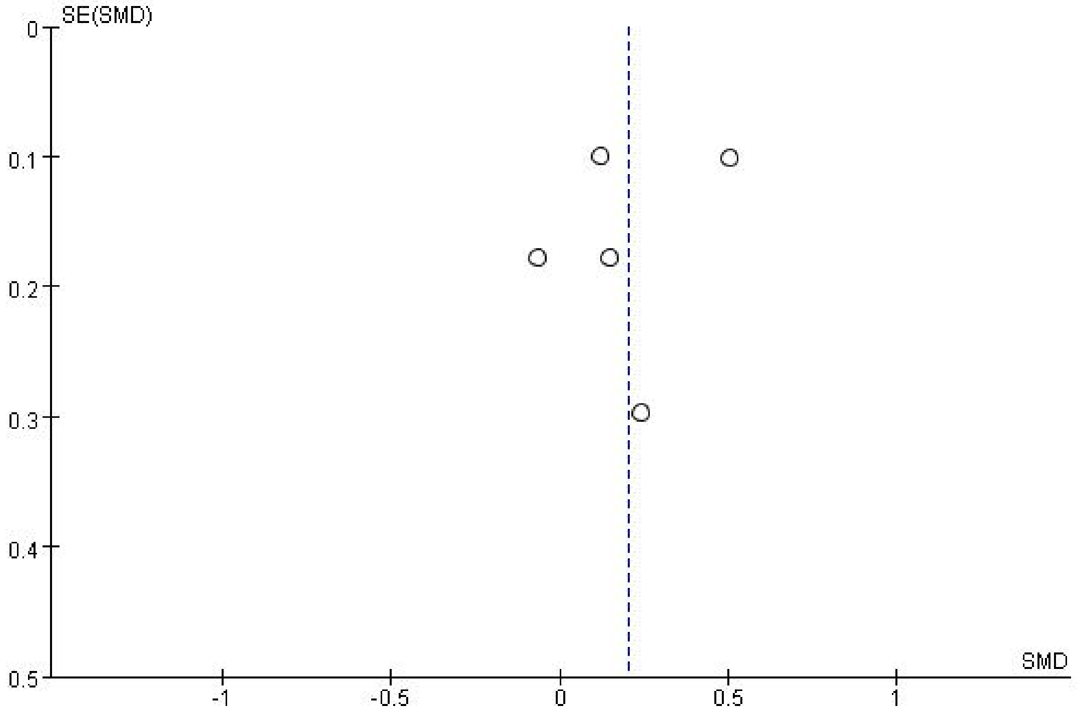

2.88 CD4/CD8(EEN)

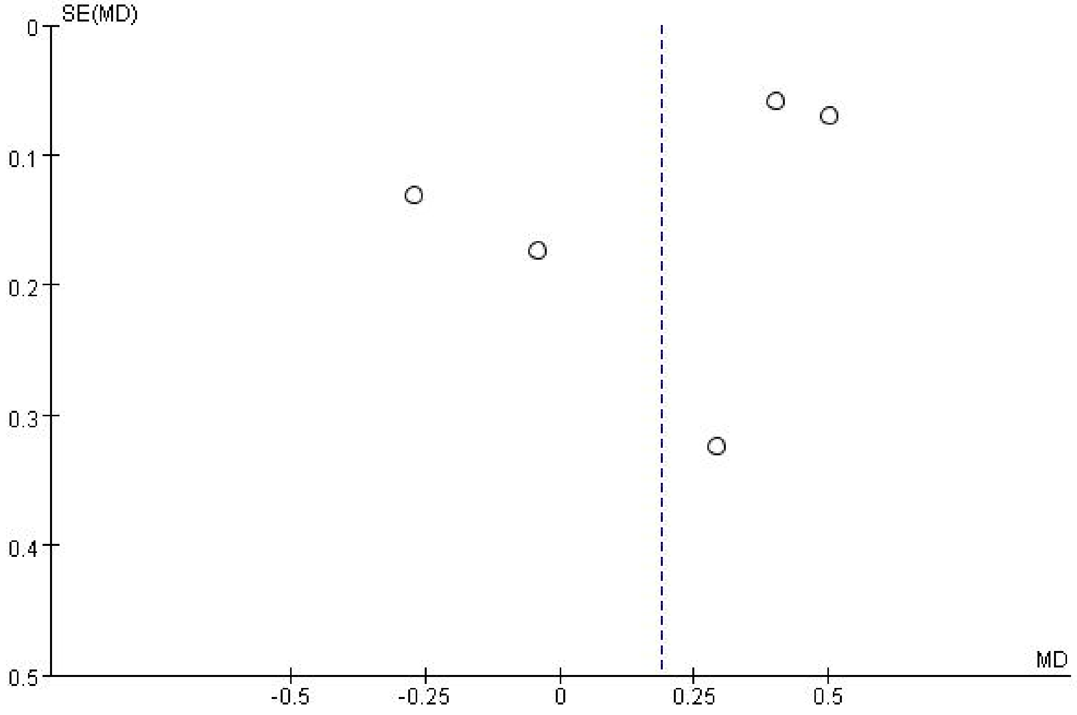

2.89 CD3(EN+PN)

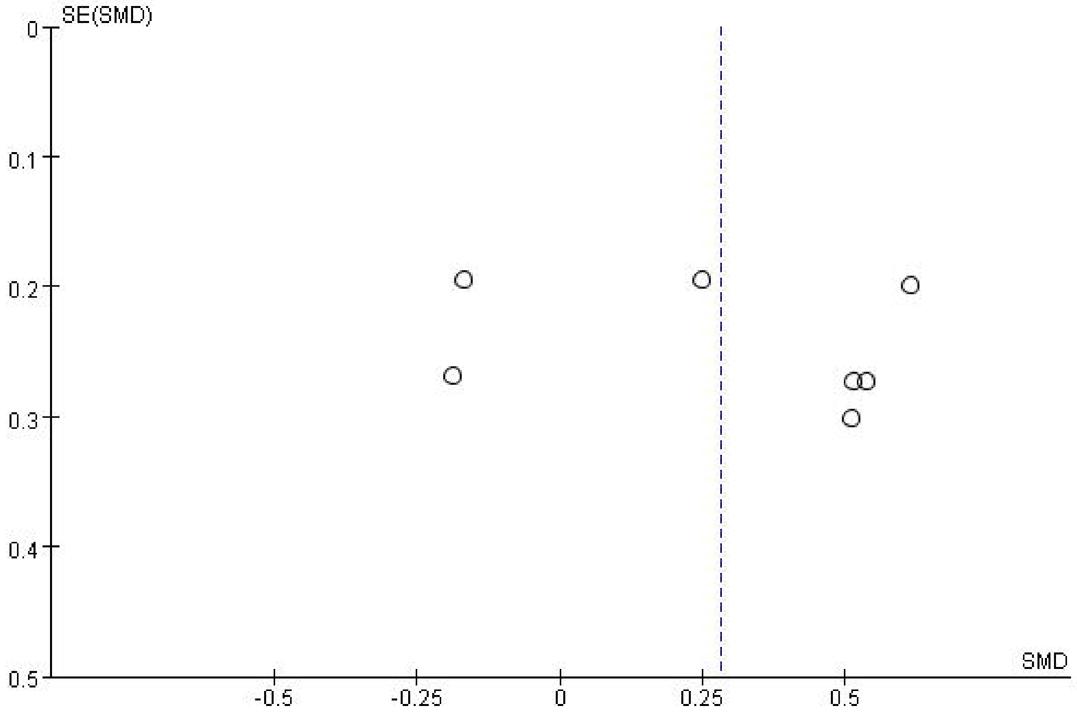

2.90 CD4(EN+PN)

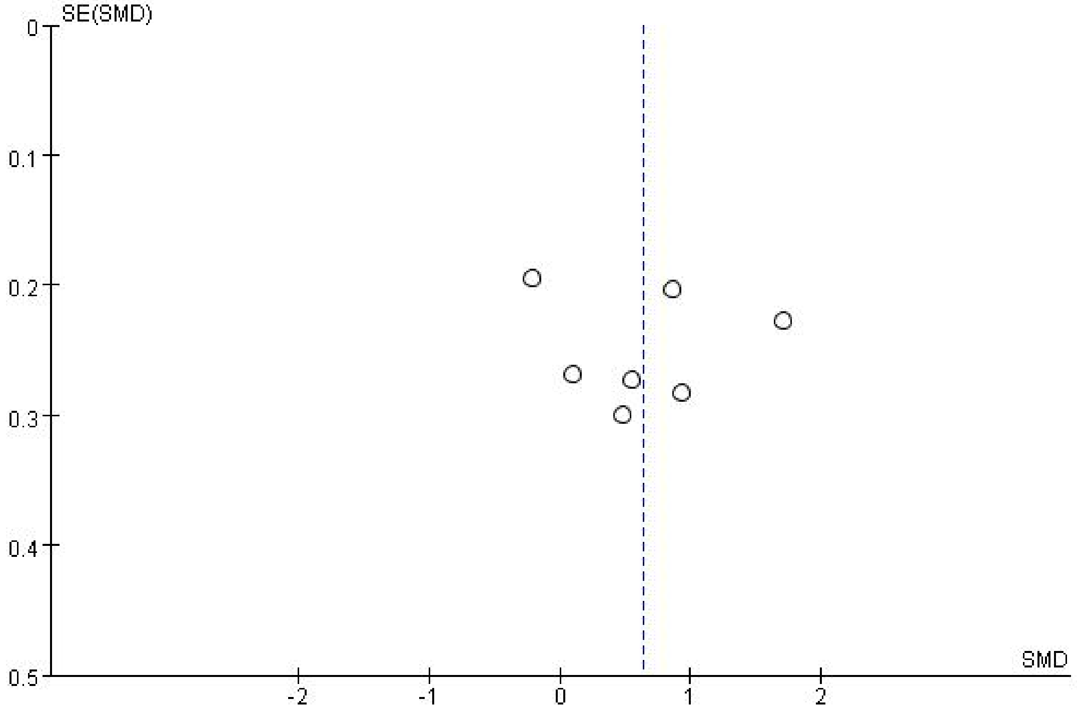

2.91 CD8(EN+PN)

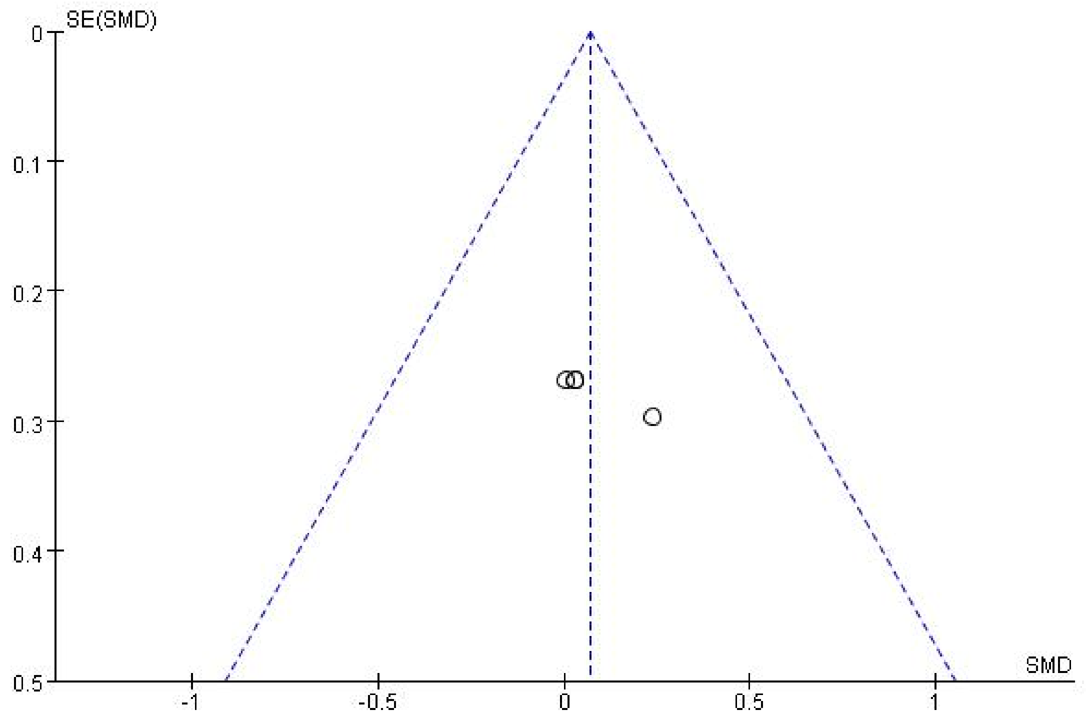

2.92 CD4/CD8(EN+PN)

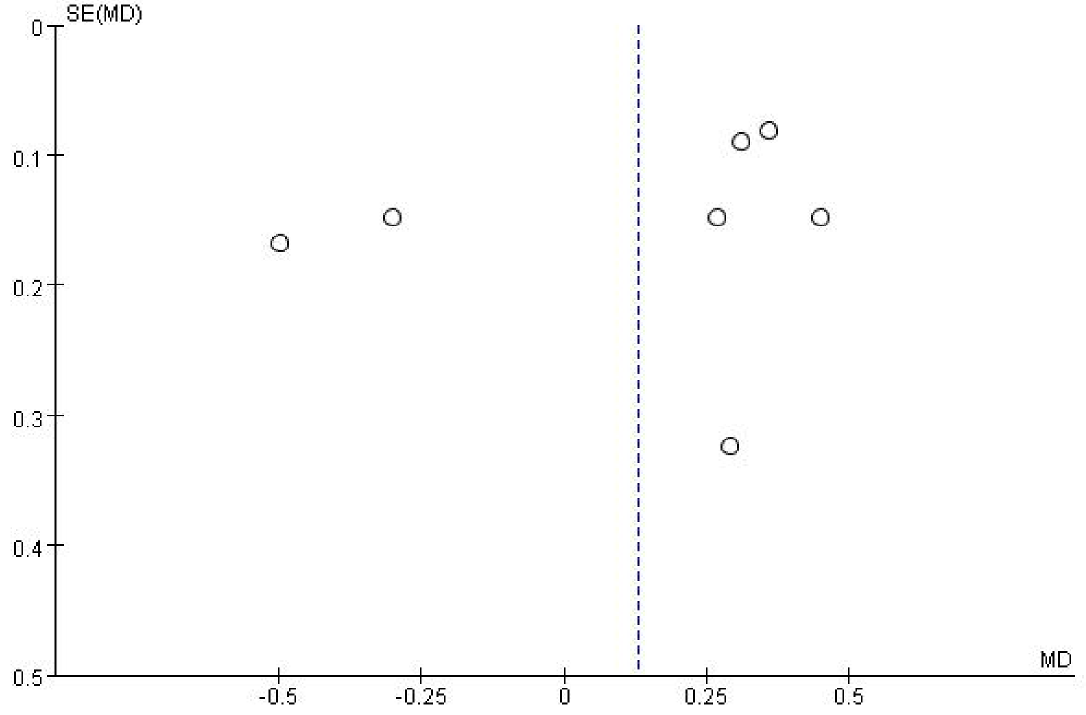

2.93 CD3(Immune+PN)

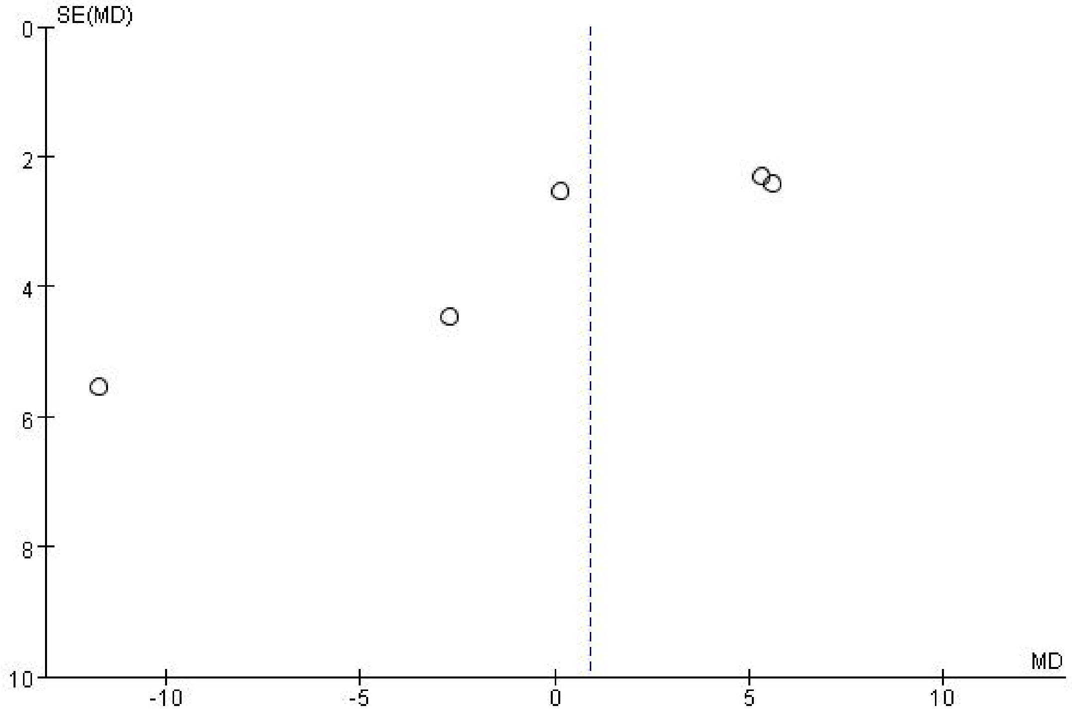

2.94 CD4(Immune+PN)

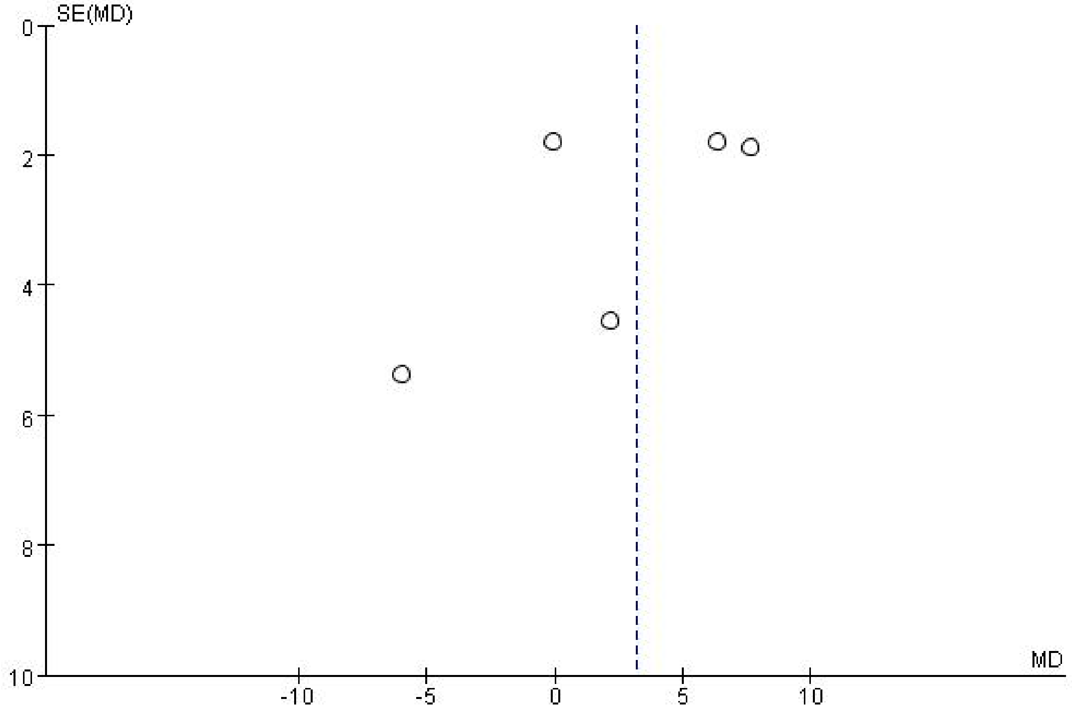

2.95 CD8(Immune+PN)

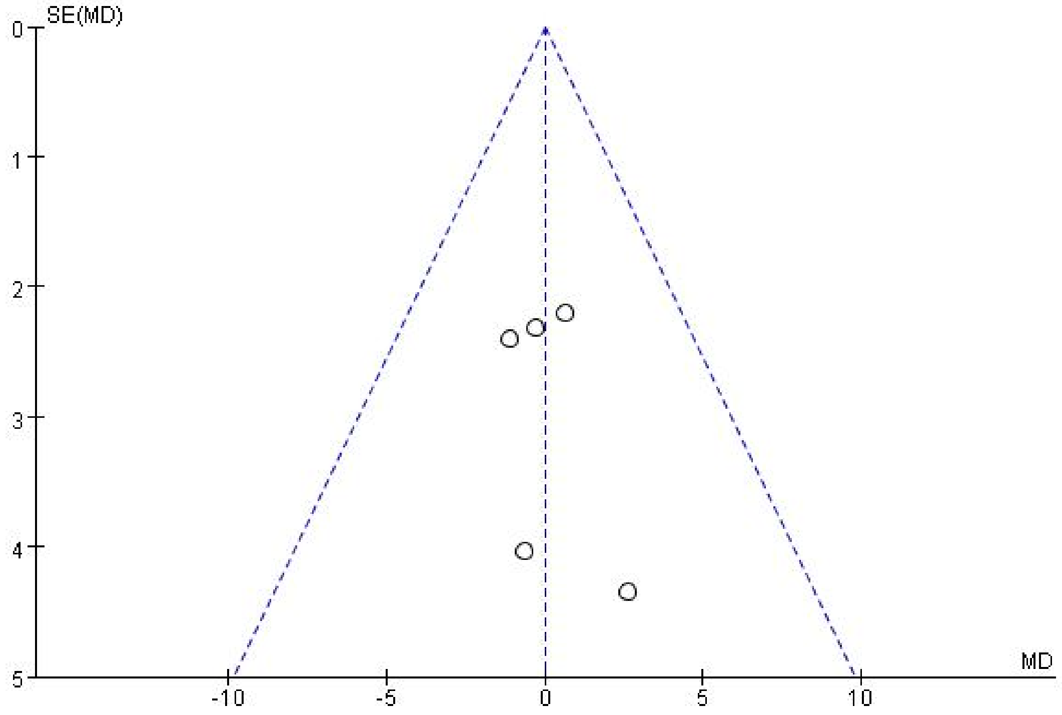

2.96 CD4/CD8(Immune+PN)

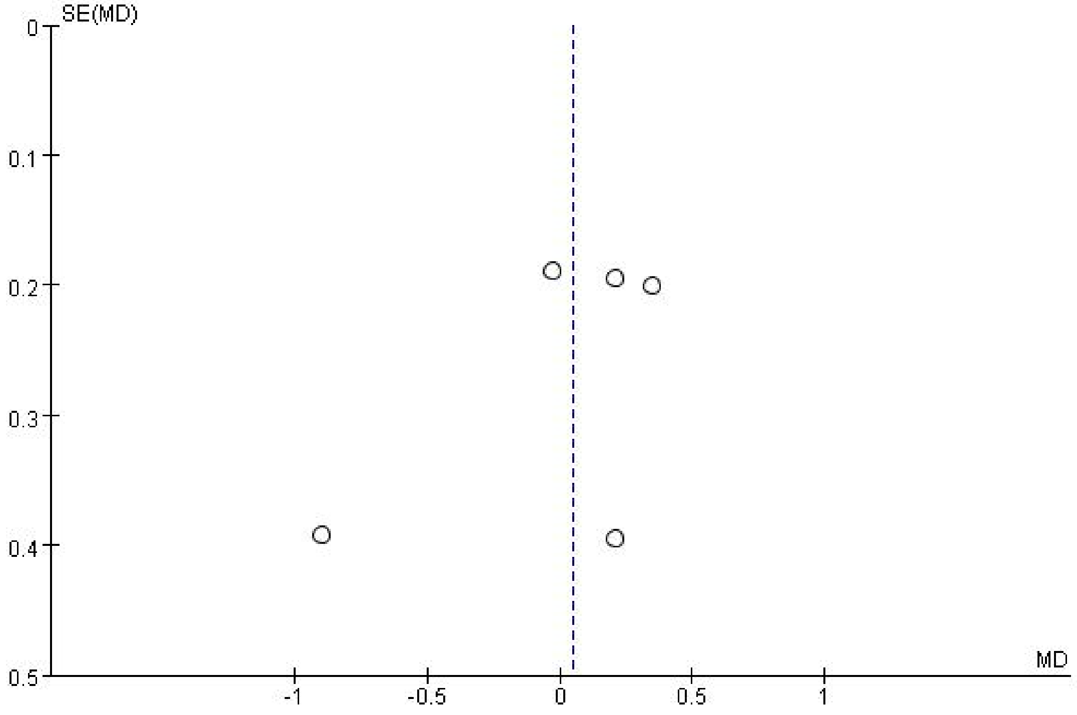

2.97 CD4(EN before)

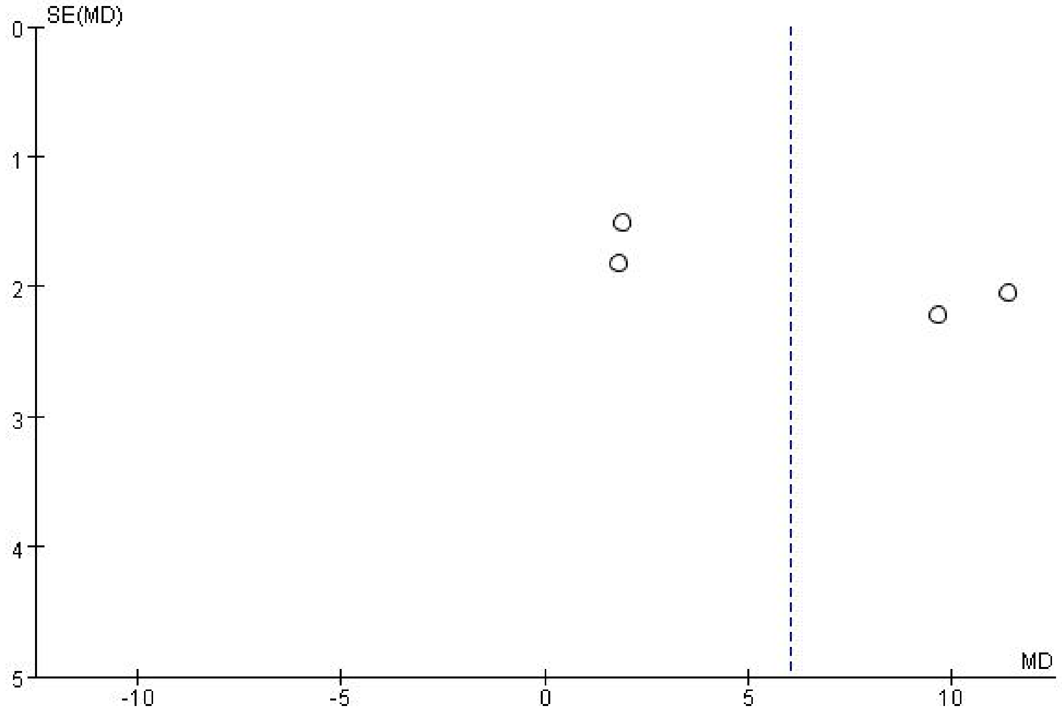

2.98 CD8(EN before)

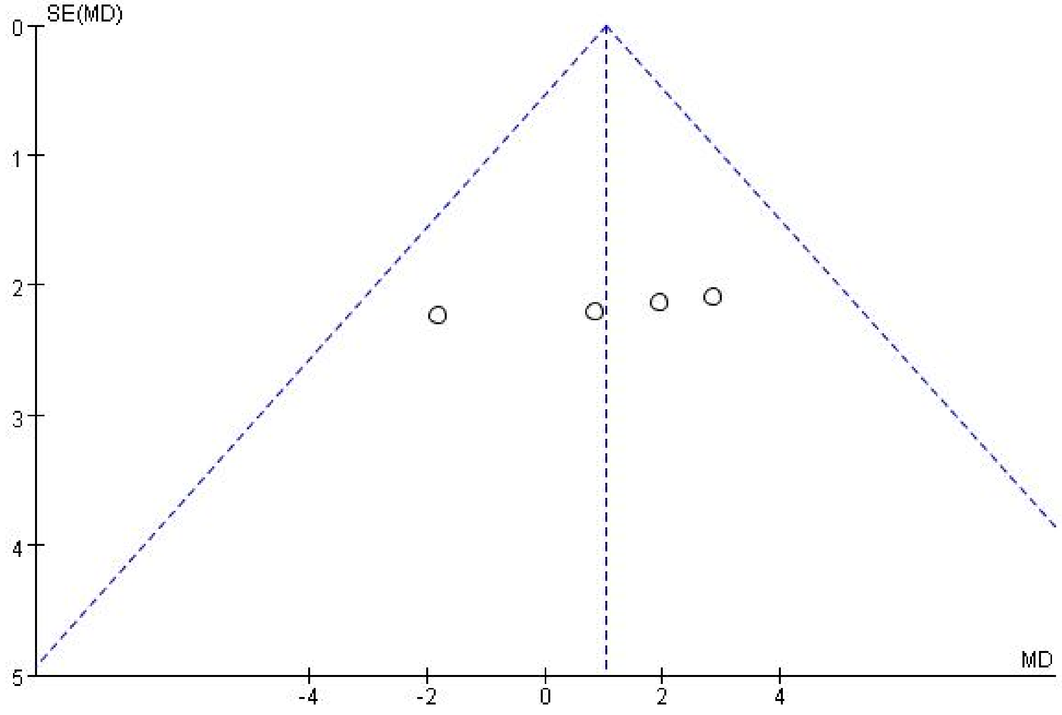

2.99 CD4/CD8(EN before)

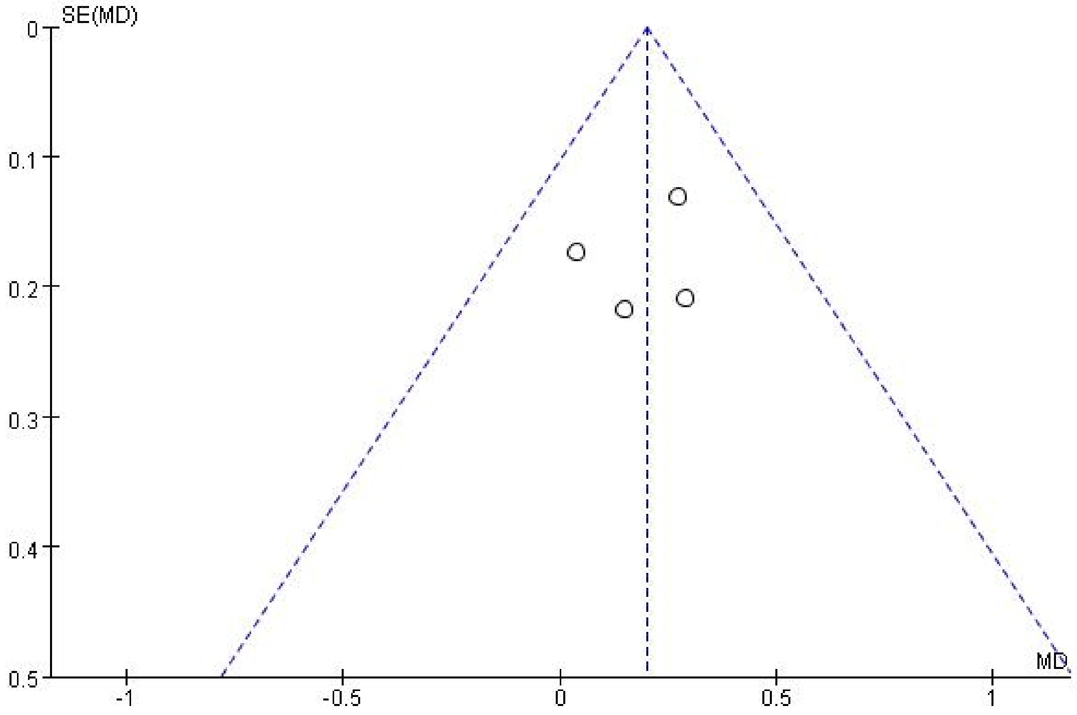

SE(MD)
0
0.1
0.2
0.3
0.4
0.5
-1
-0.5
0
0.5
1
MD
