# Supplementary material for: What are the beneficial treatment strategies in maintaining T lymphocyte subsets after cancer surgery? A systematic review and network meta-analysis
Source: Front Immunol. 2026 Jul 14;17:1854279. doi: 10.3389/fimmu.2026.1854279 (PMC13408238; doi:10.3389/fimmu.2026.1854279)

Figure S3 Network plots of available comparisons among all included interventions for different time.

3.1 CD3(SACT,1D)

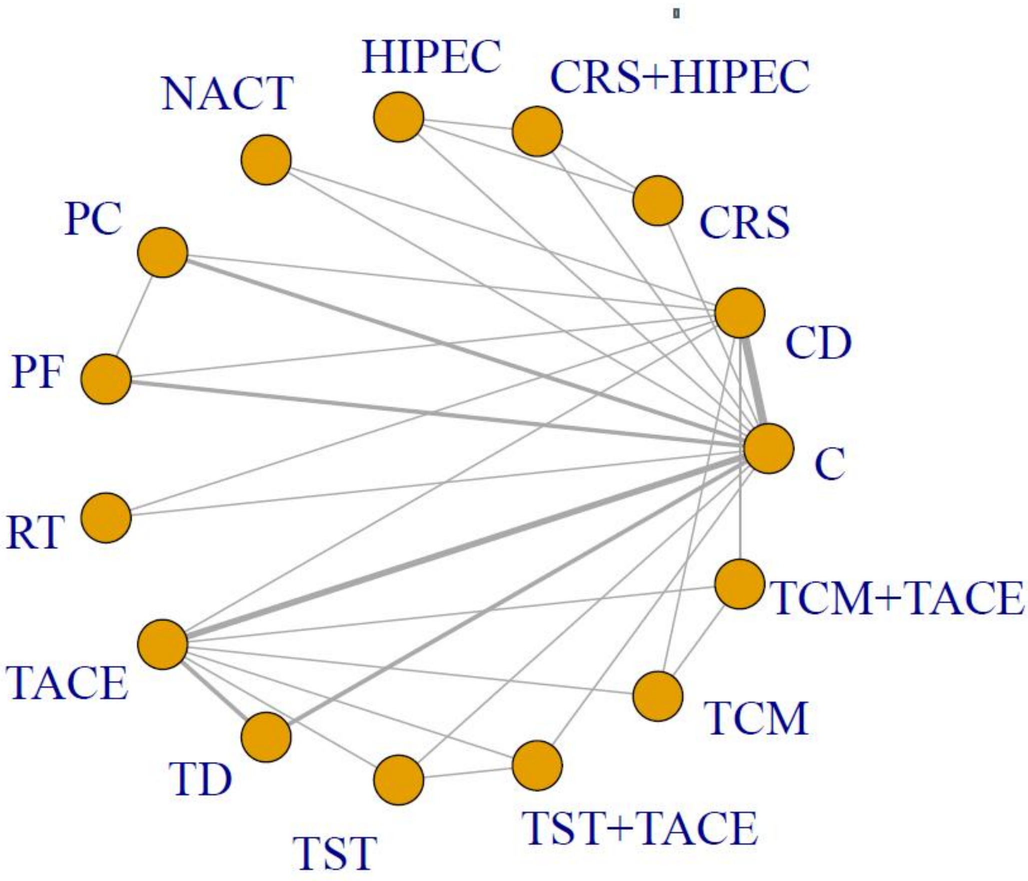

3.2 CD3(SACT,14D)

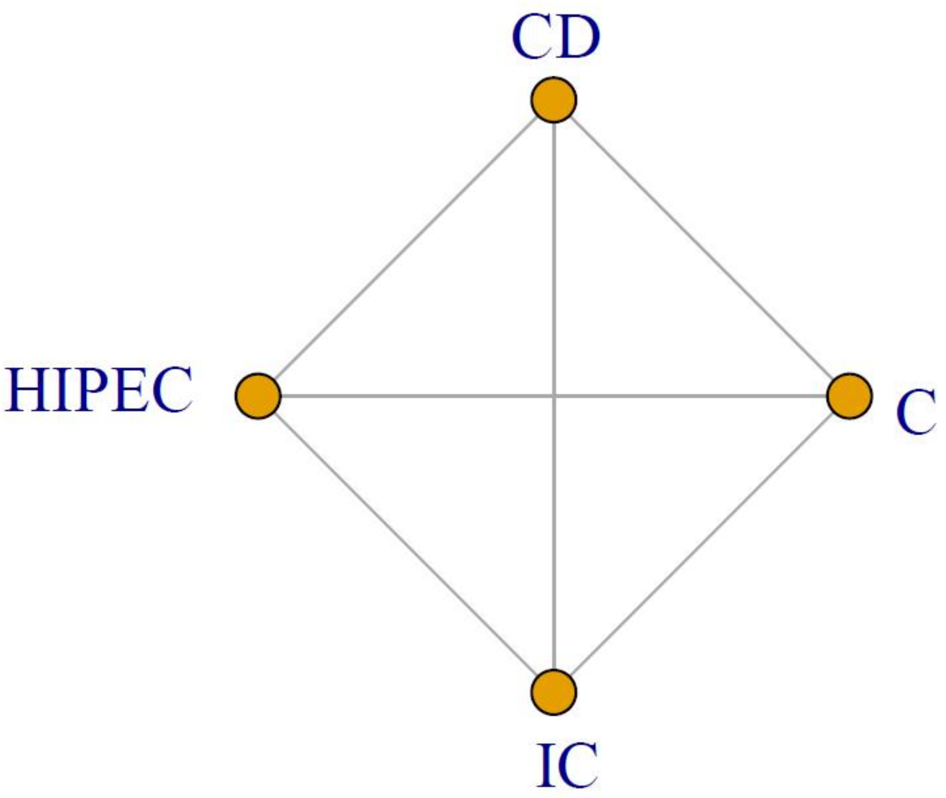

3.3 CD3(SACT,3M)

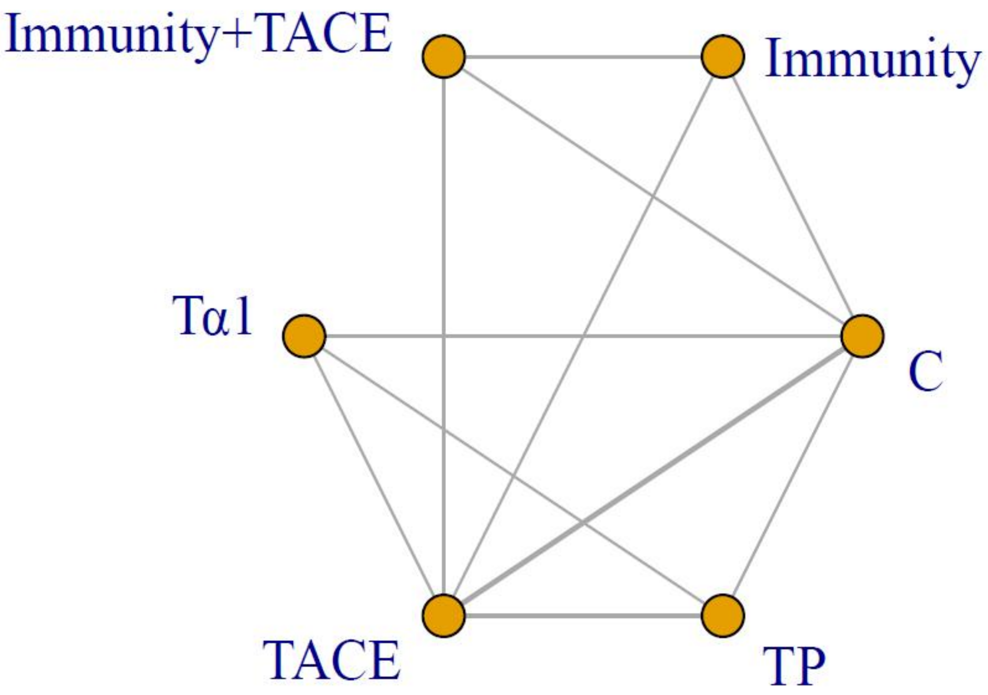

3.4 CD4(SACT,1D)

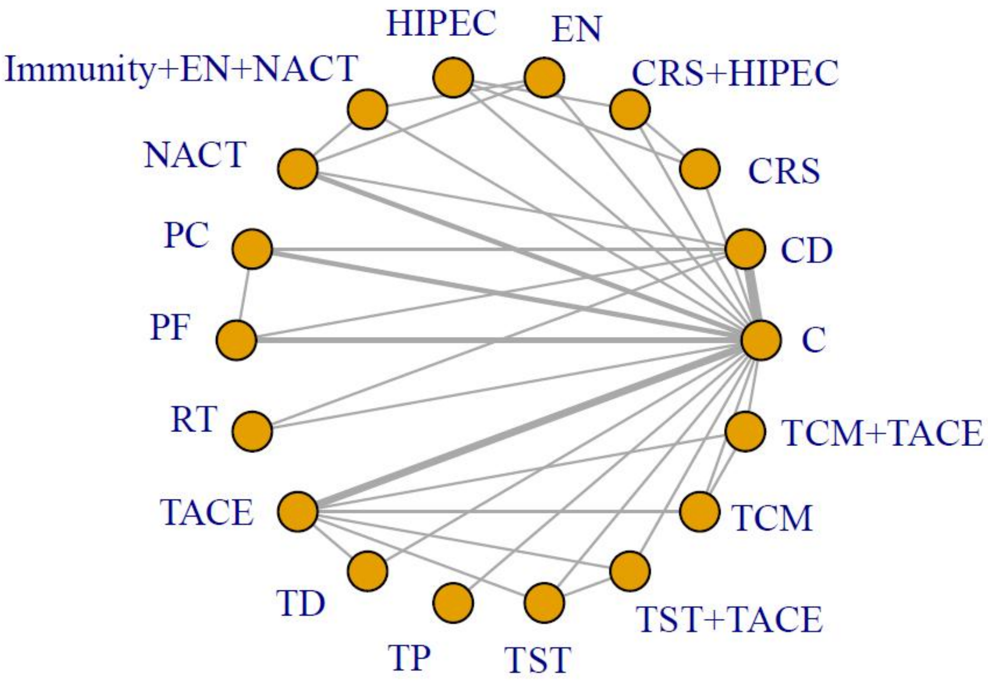

3.5 CD4(SACT,14D)

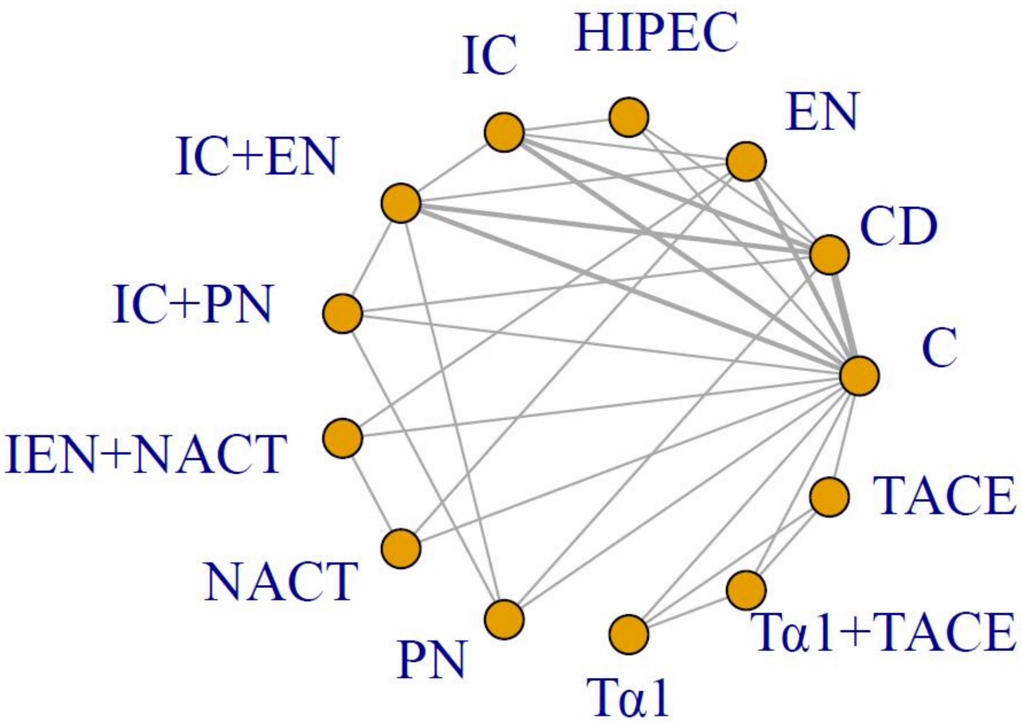

3.6 CD4(SACT,3M)

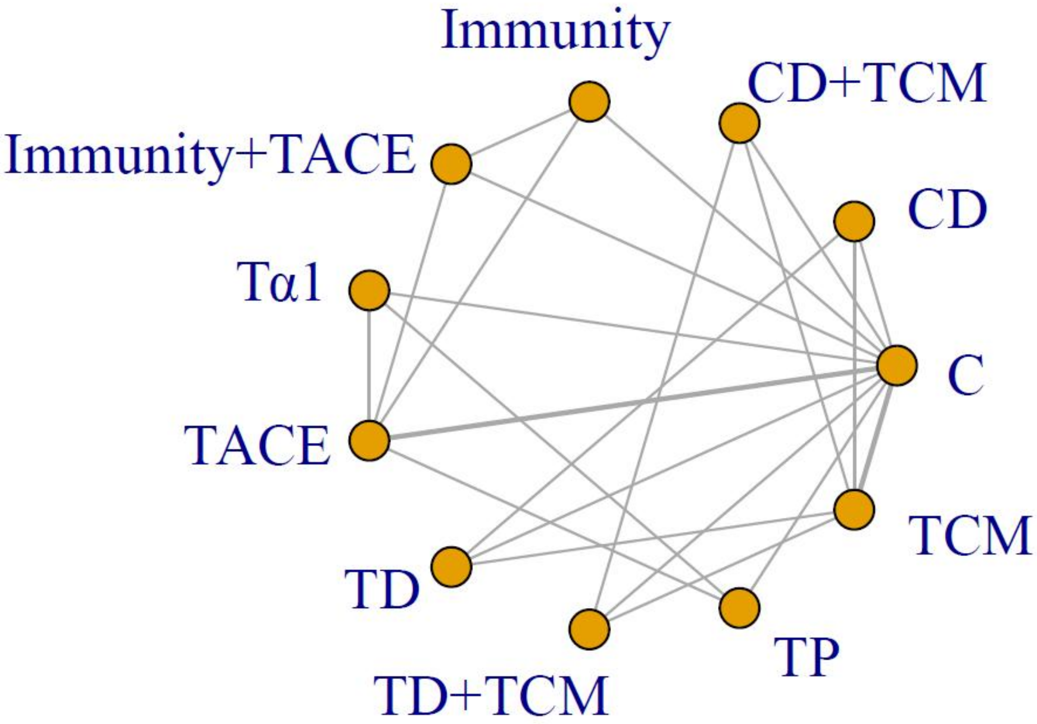

3.7 CD8(SACT,1D)

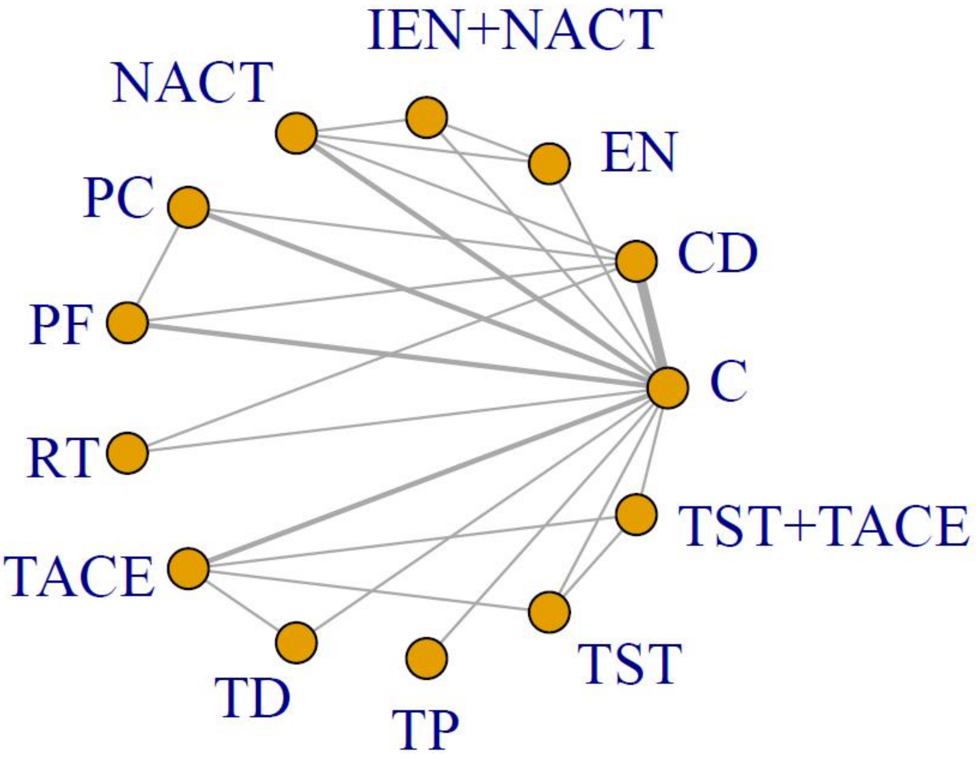

3.8 CD8(SACT,14D)

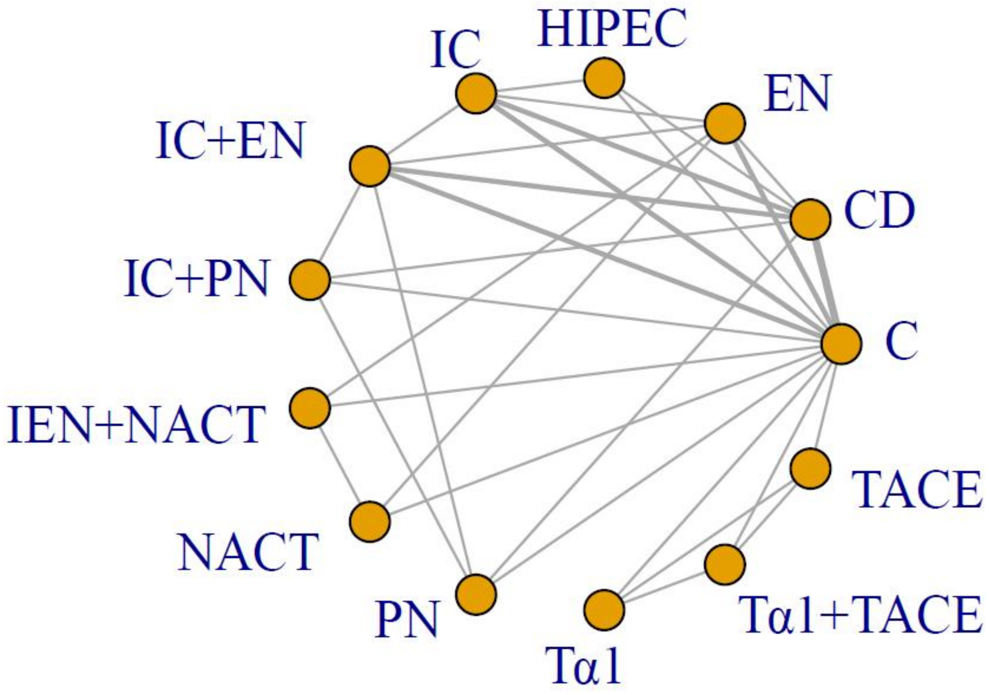

3.9 CD8(SACT,3M)

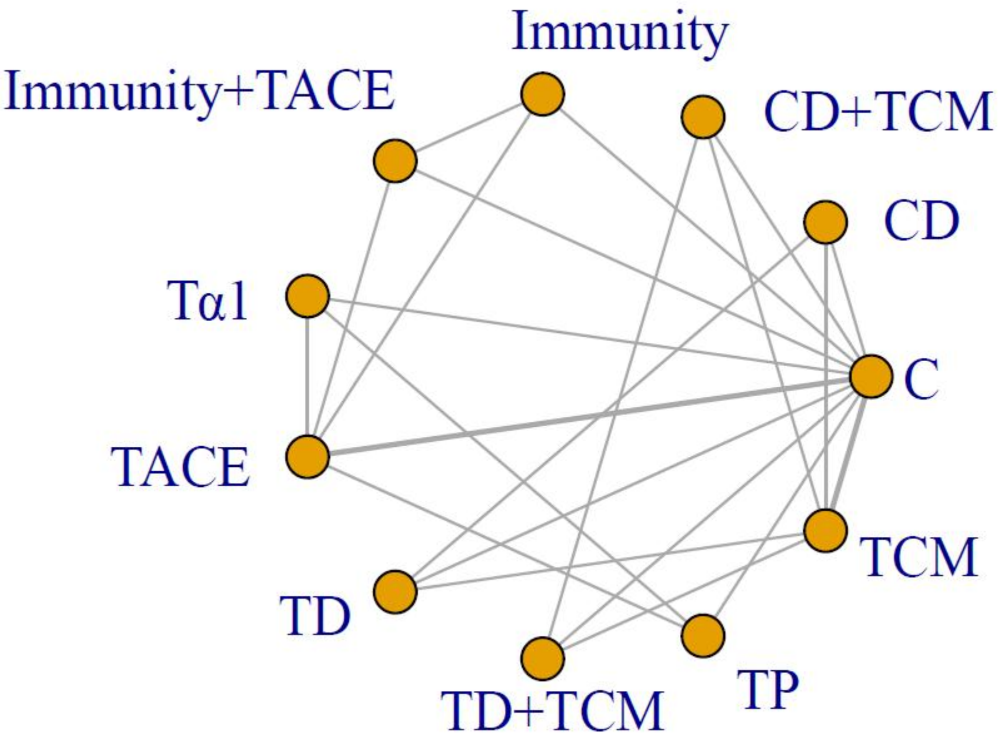

3.10 CD4/CD8(SACT,1D)

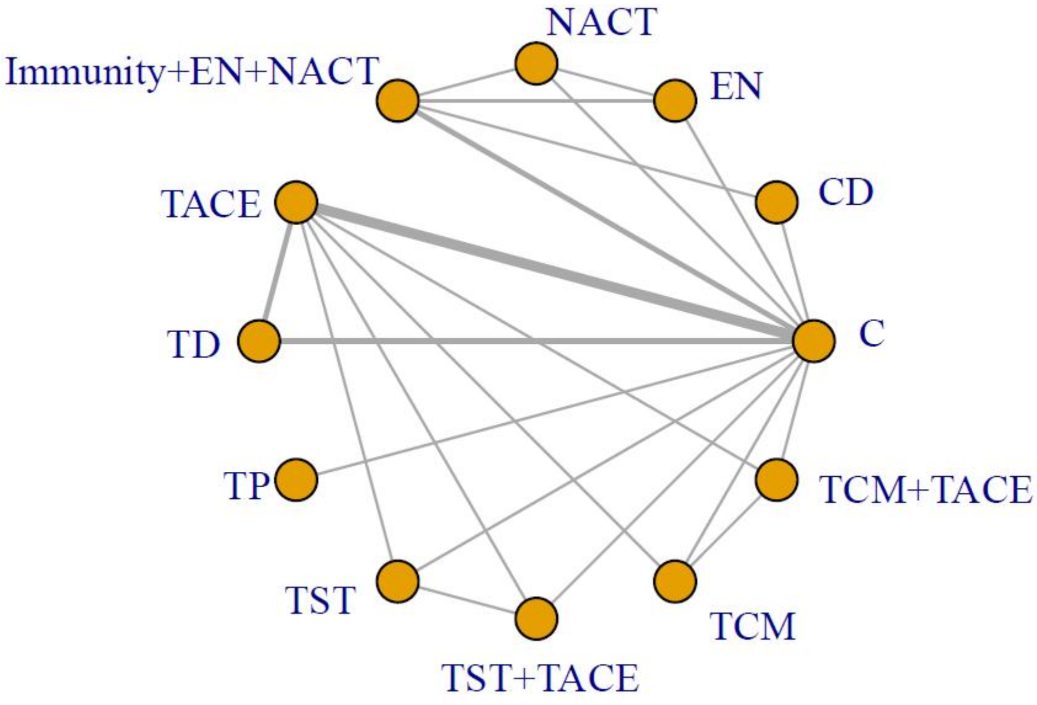

3.11 CD4/CD8(SACT,14D)

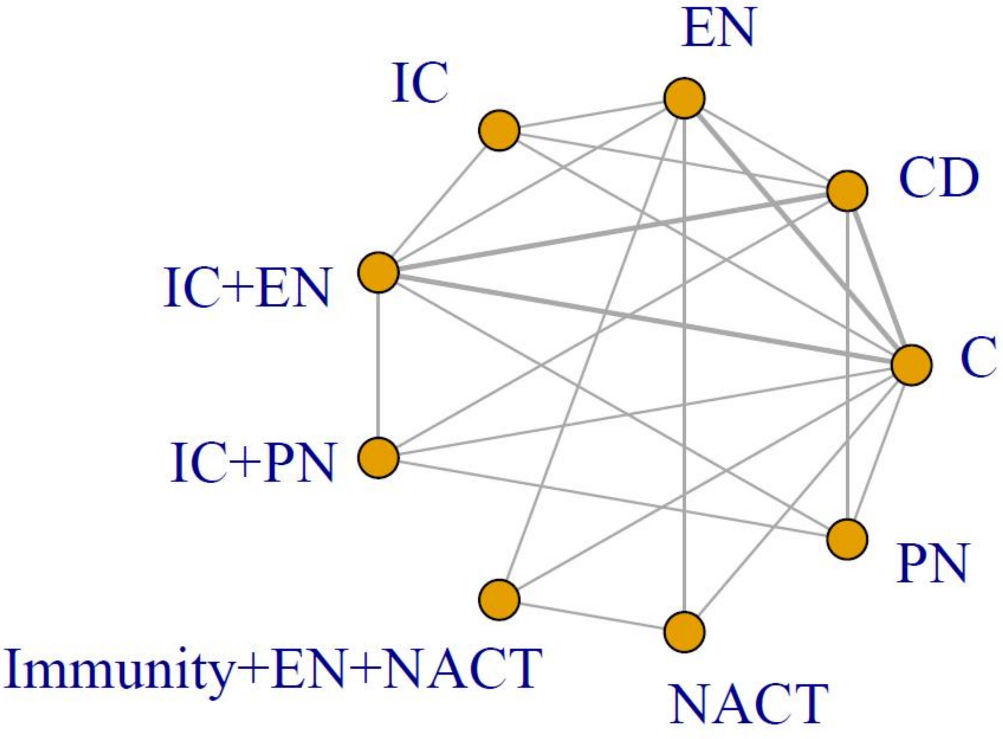

3.12 CD4/CD8(SACT,3M)

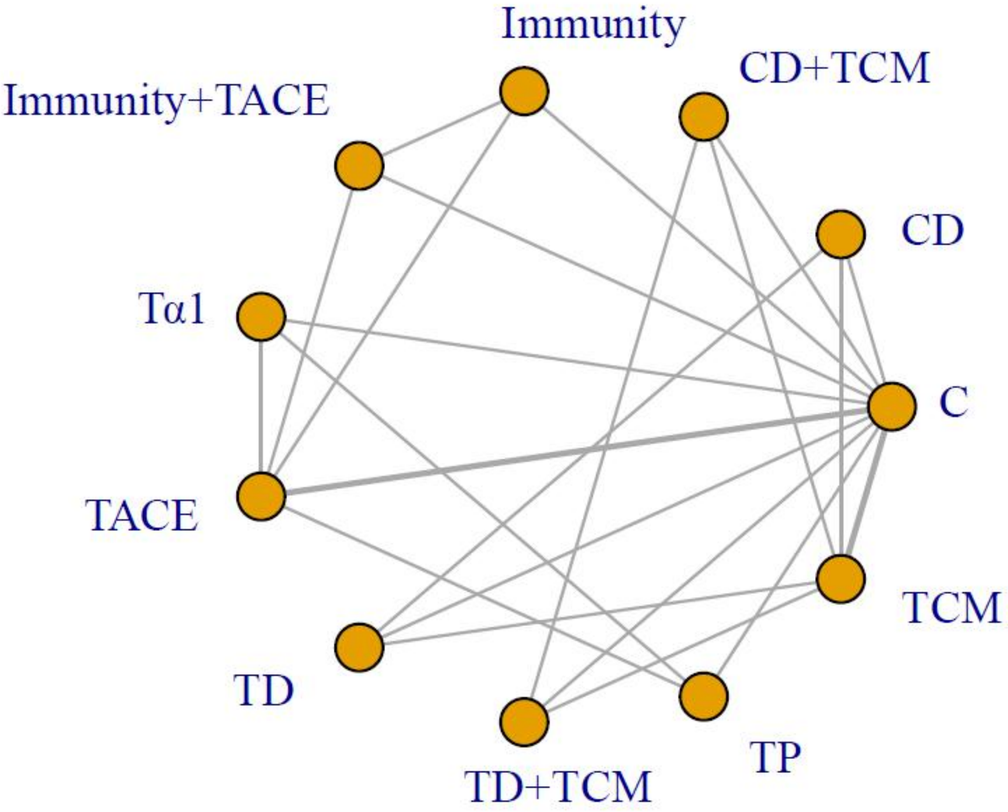

3.13 CD3(Nutrition,1D)

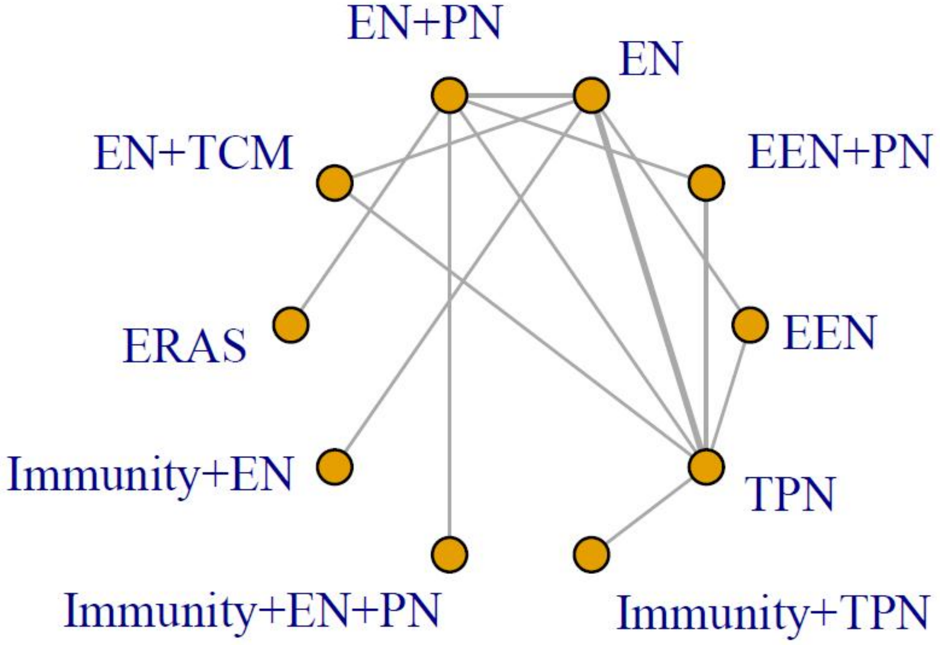

3.15 CD3(Nutrition,<7D)

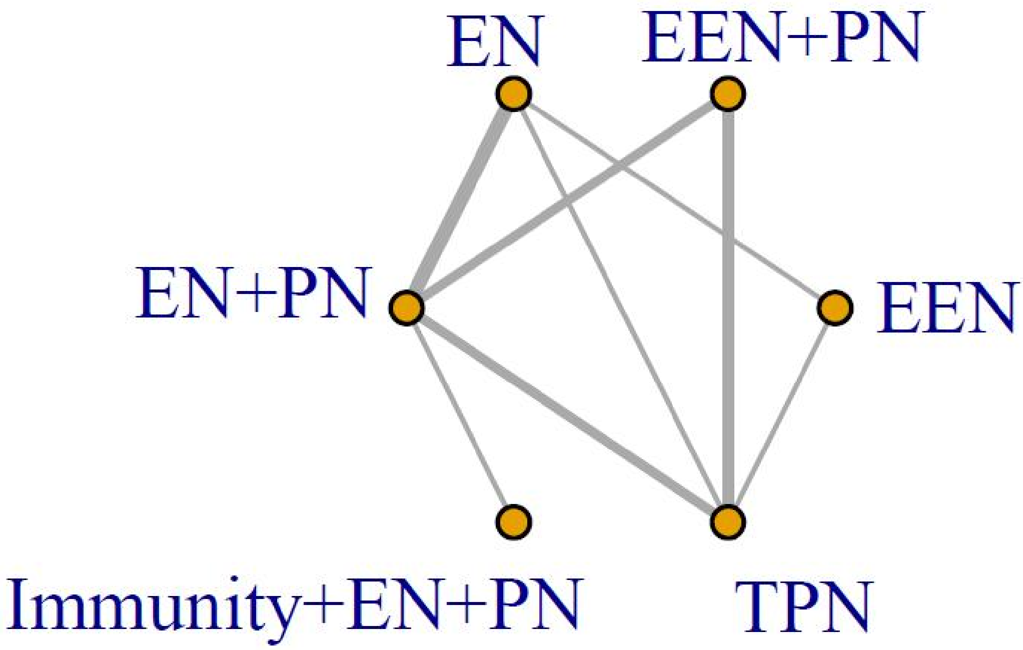

3.14 CD3(Nutrition,>7D)

CD3>7D

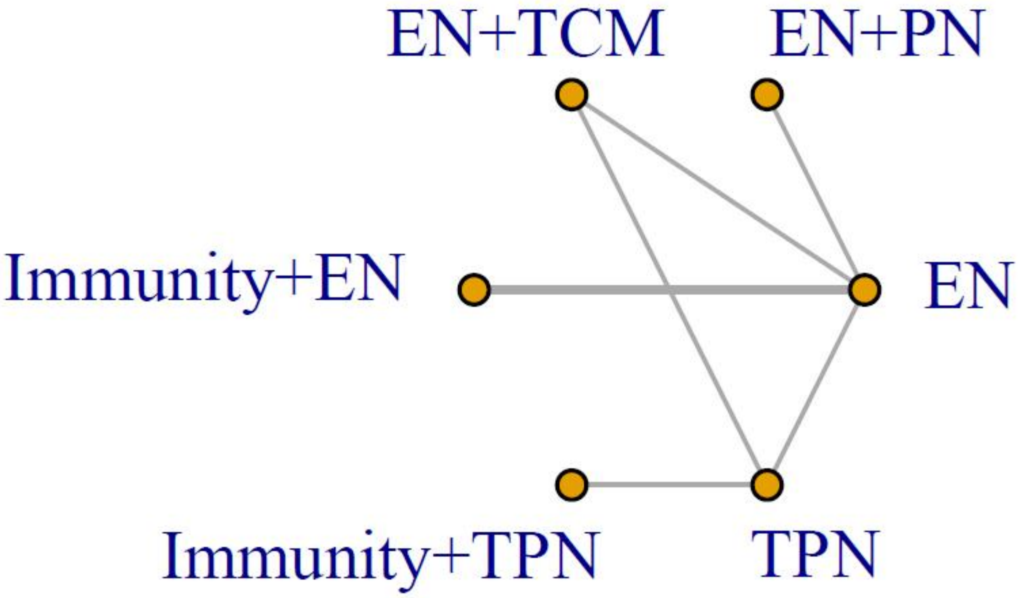

3.16 CD4(Nutrition,1D)

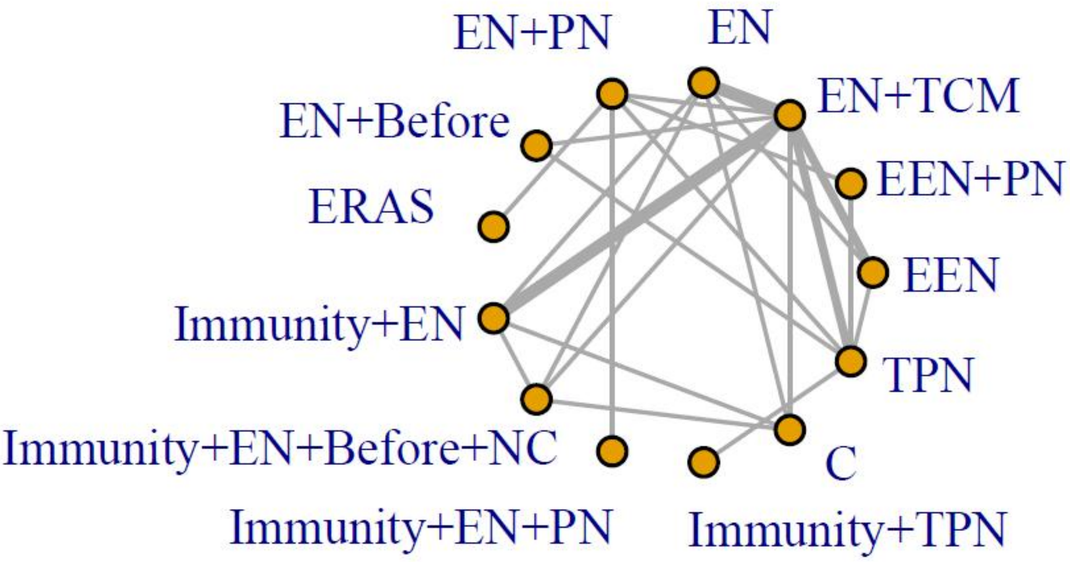

3.17 CD4(Nutrition,<7D)

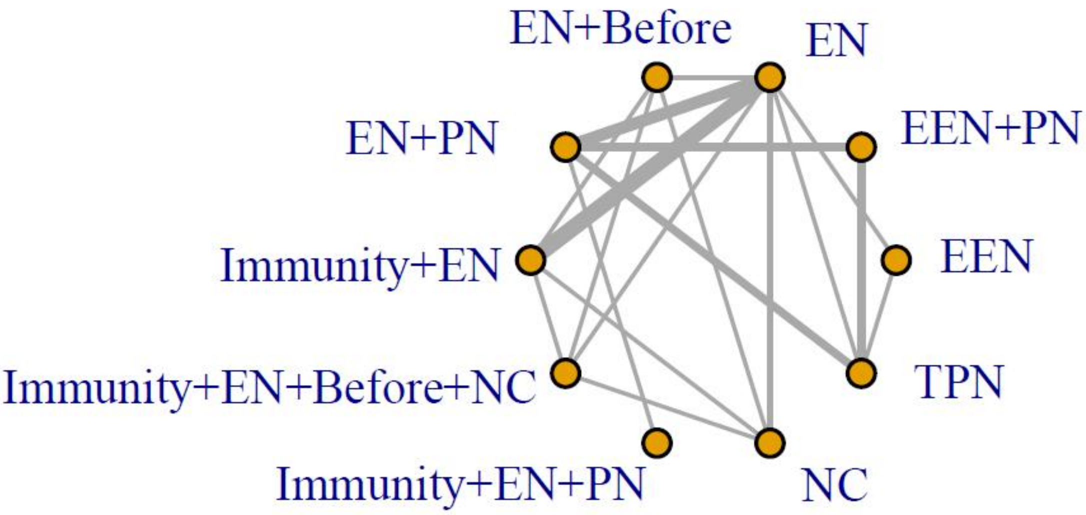

3.19 CD4(Nutrition,>7D)

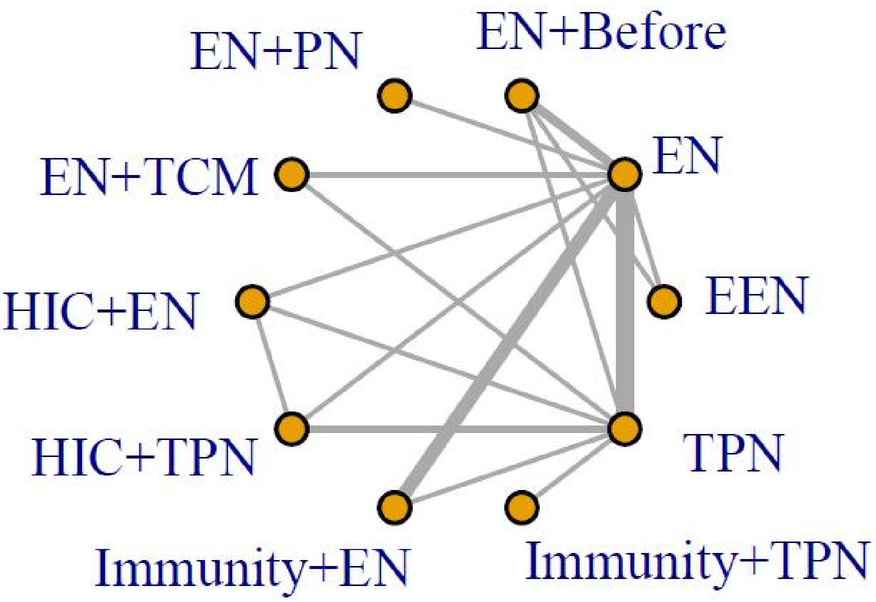

3.18 CD8(Nutrition,1D)

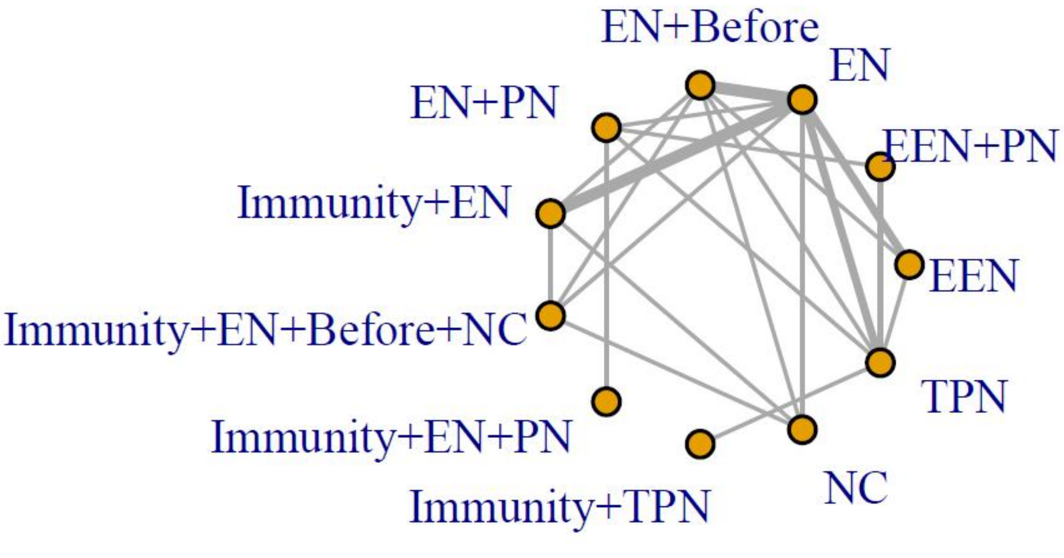

3.20 CD8(Nutrition,<7D)

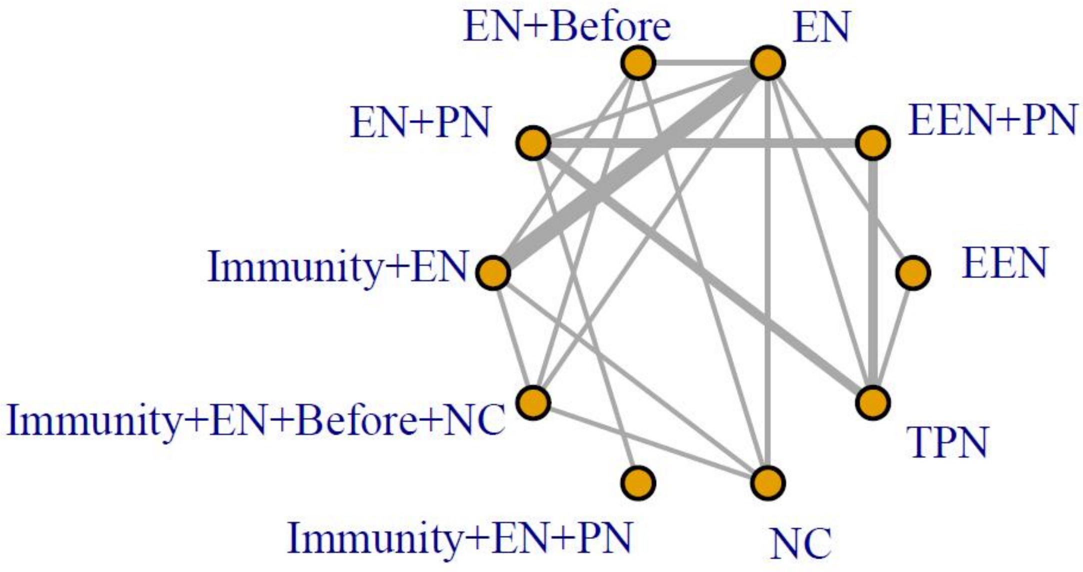

3.27 CD8(Nutrition,>7D)

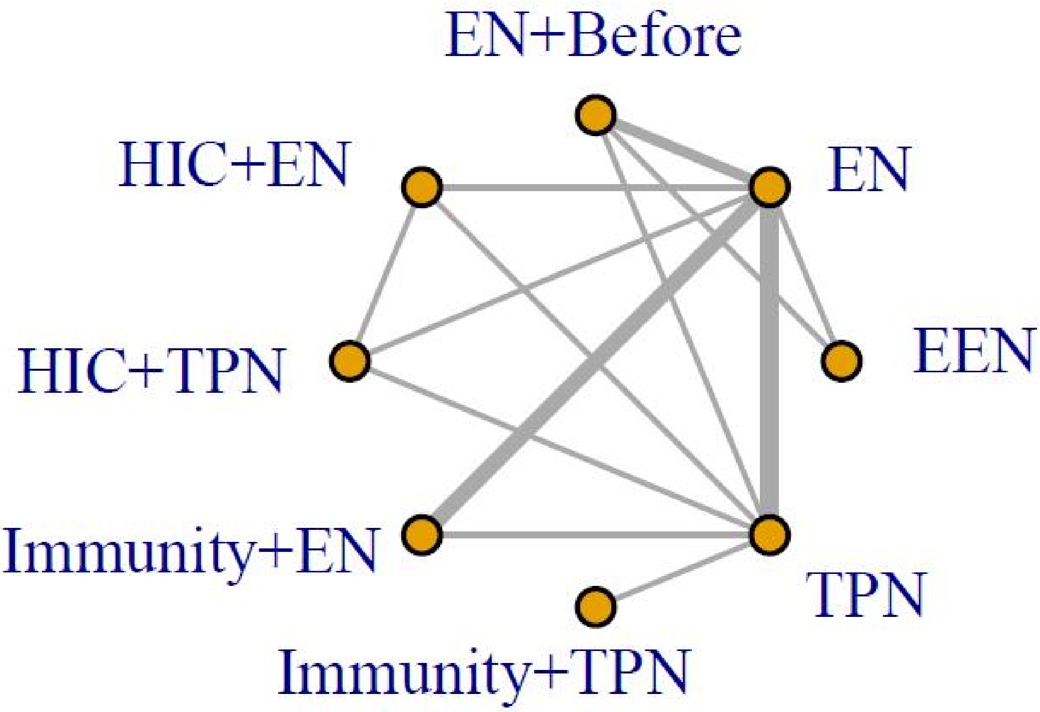

3.29 CD4/CD8(Nutrition,1D)

CD4/CD831D

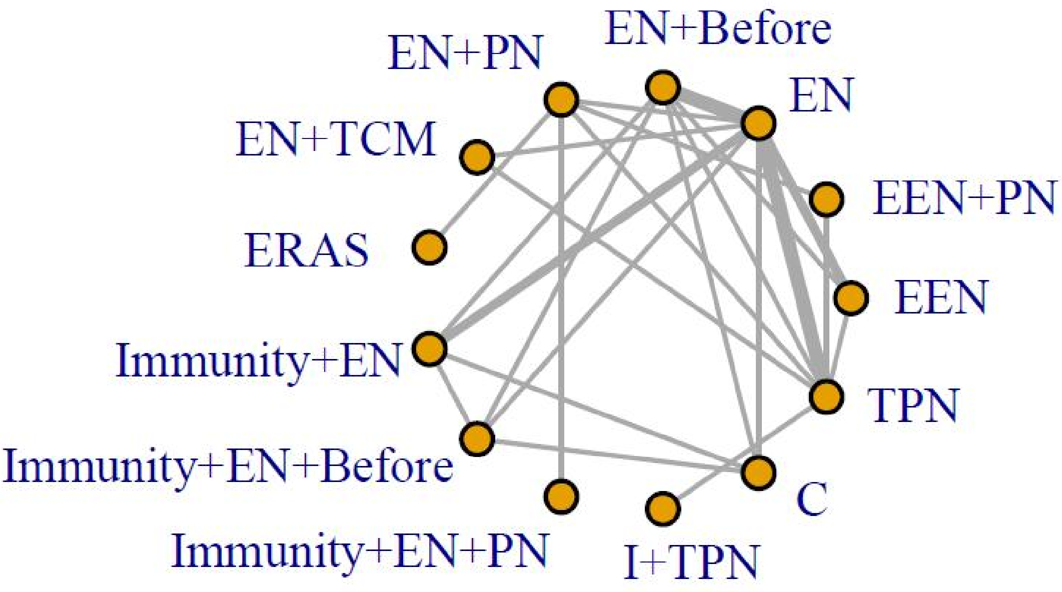

3.28 CD4/CD8(Nutrition,<7D)

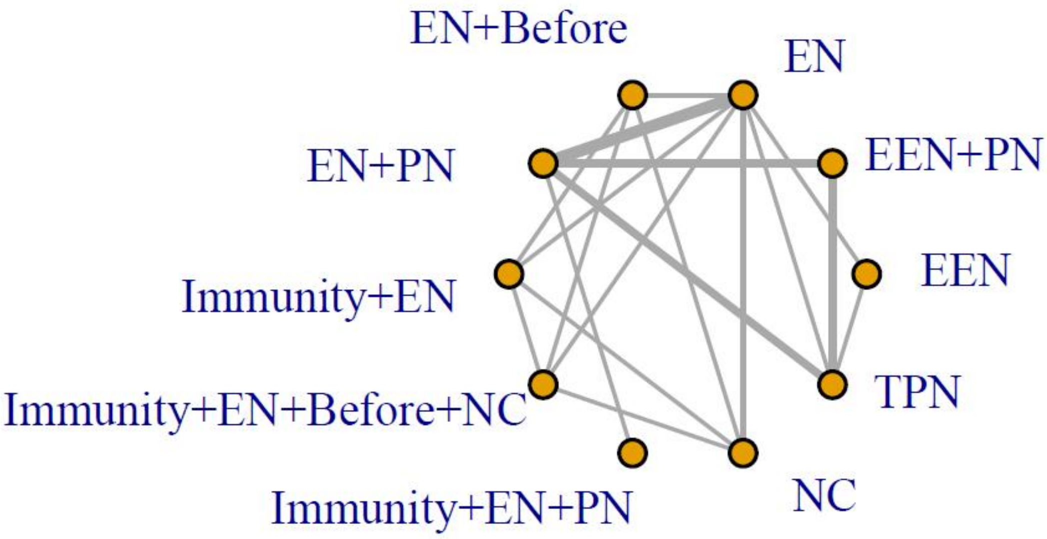

3.30 CD4/CD8(Nutrition,7D)

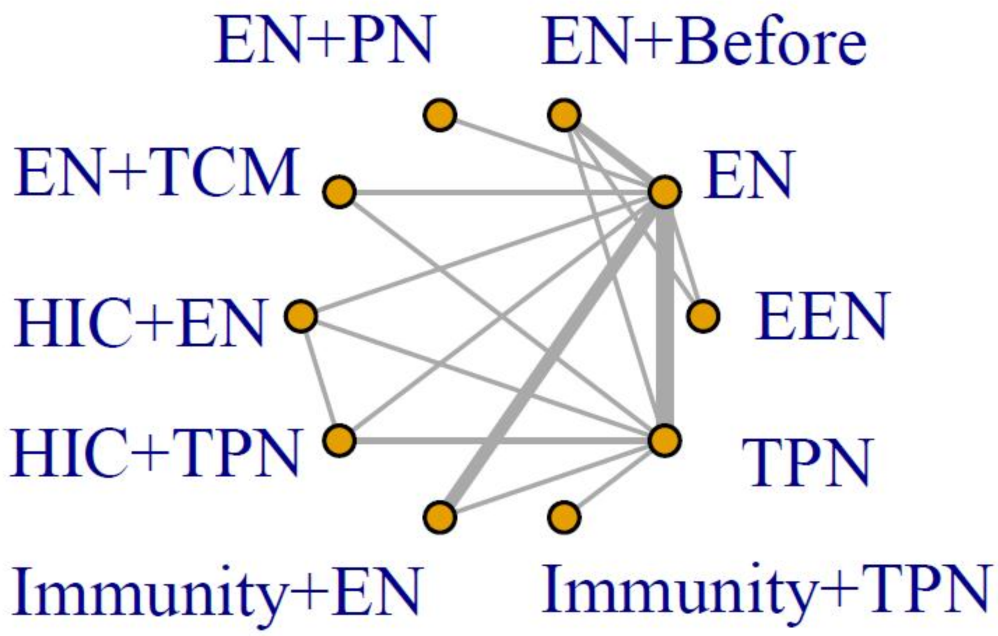

3.31 CD3(Anesthesia,before over)

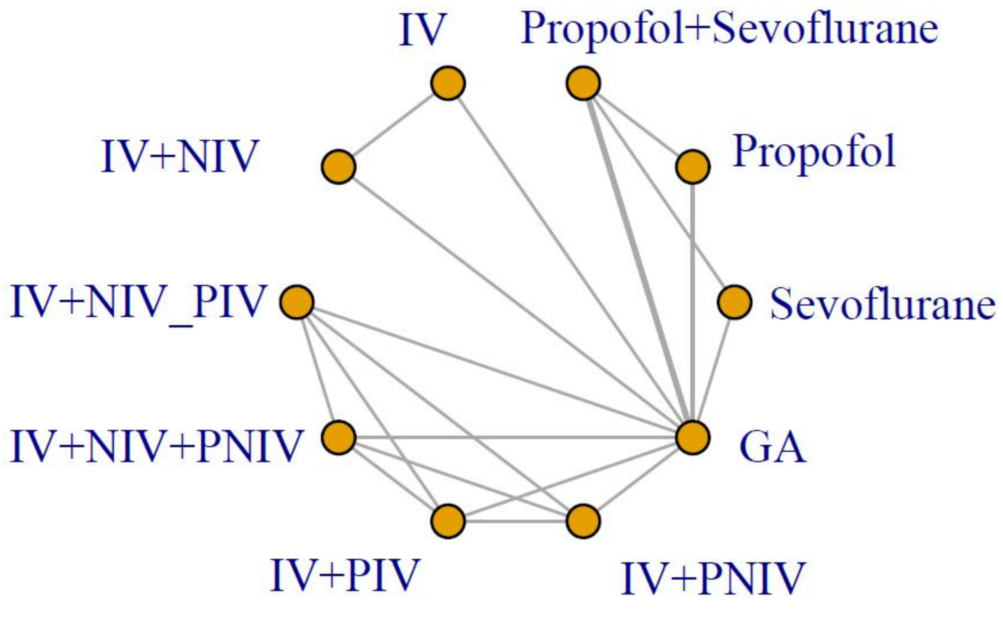

3.33 CD3(Anesthesia,over)

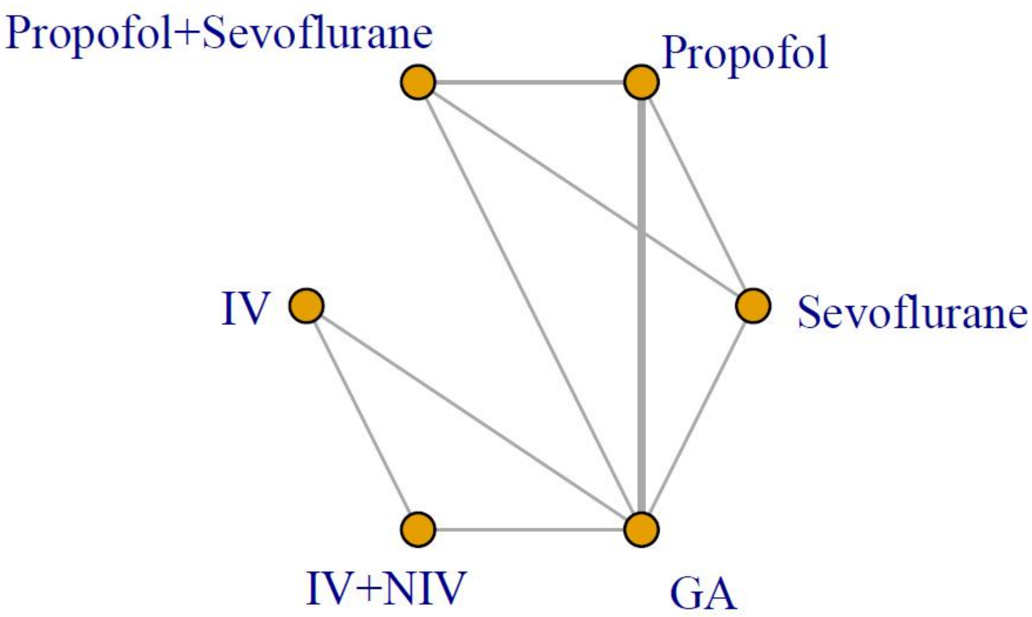

3.32 CD3(Anesthesia,12h)

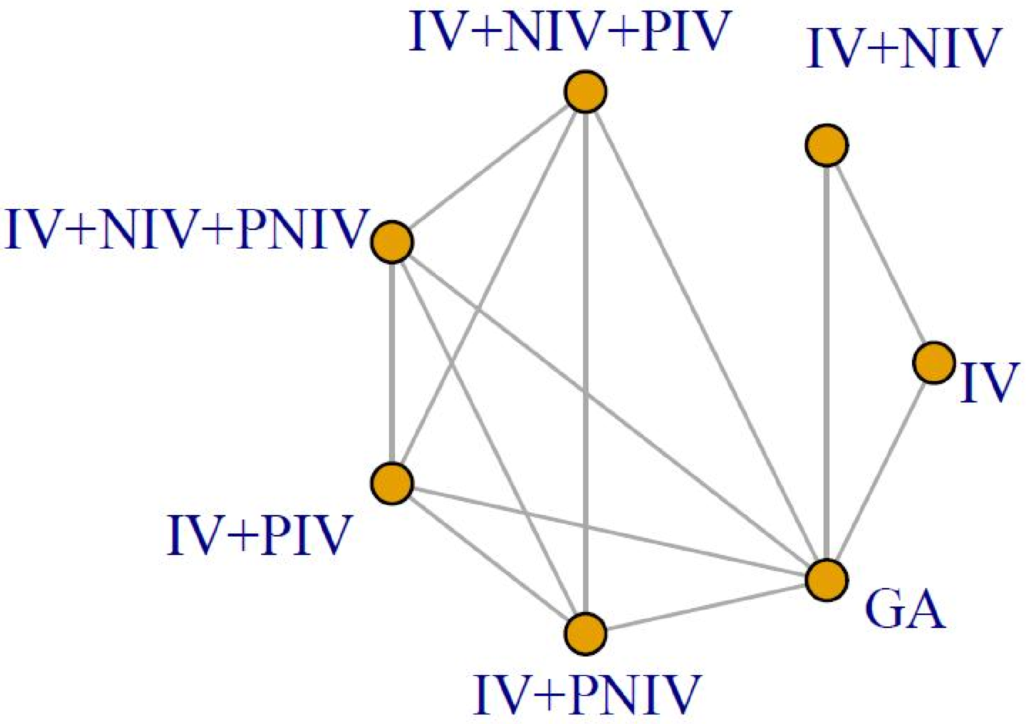

3.34 CD3(Anesthesia,1D)

CD31D

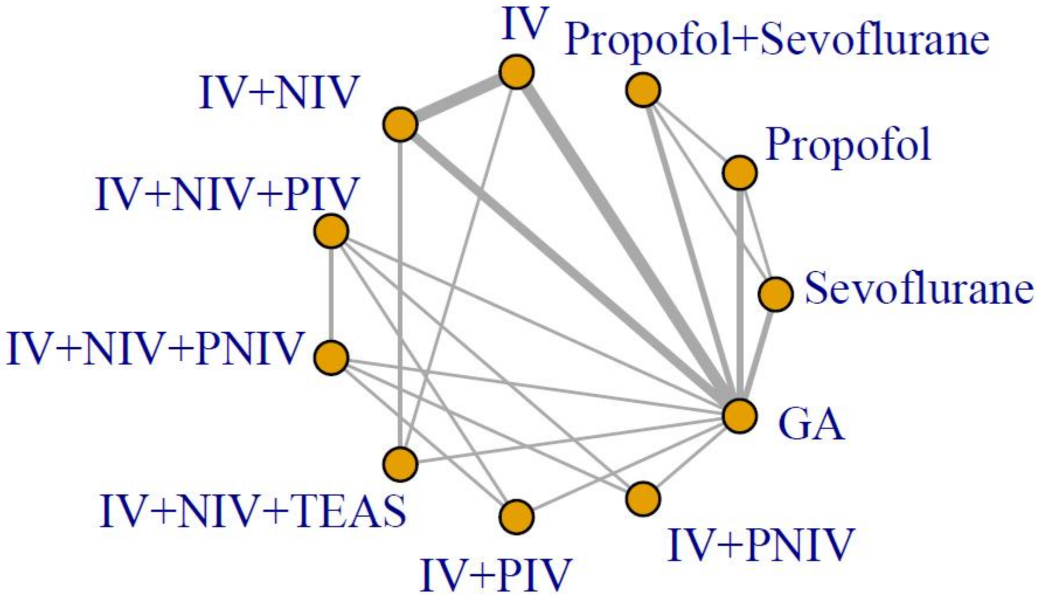

3.35 CD3(Anesthesia,2D)

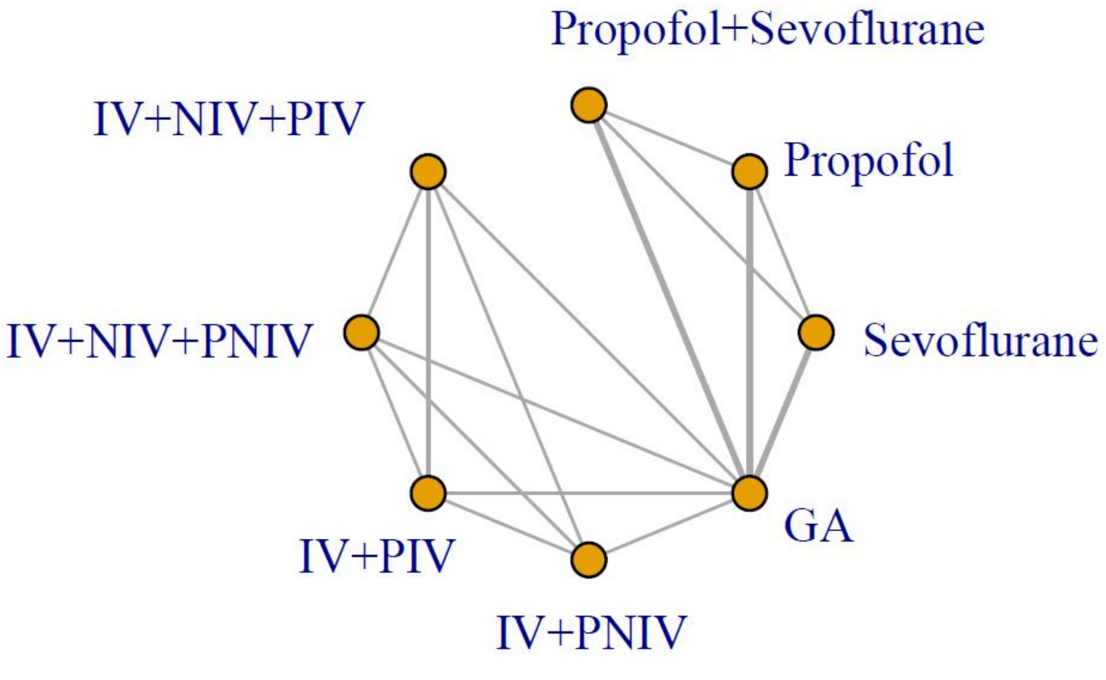

3.14 CD3(Anesthesia,3D)

CD33D

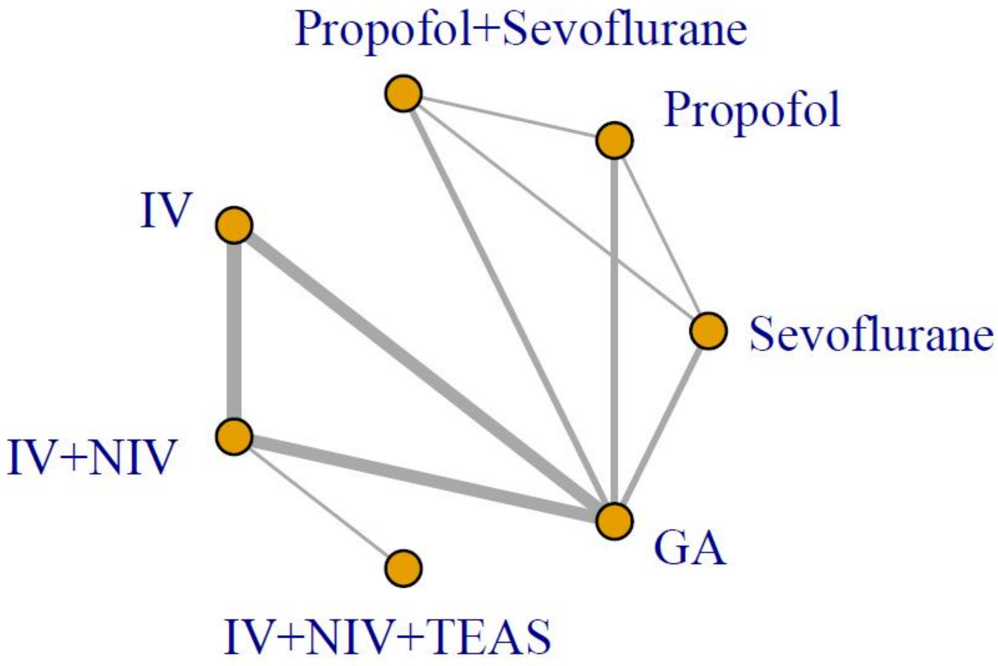

3.36CD3(Anesthesia,5-7D)

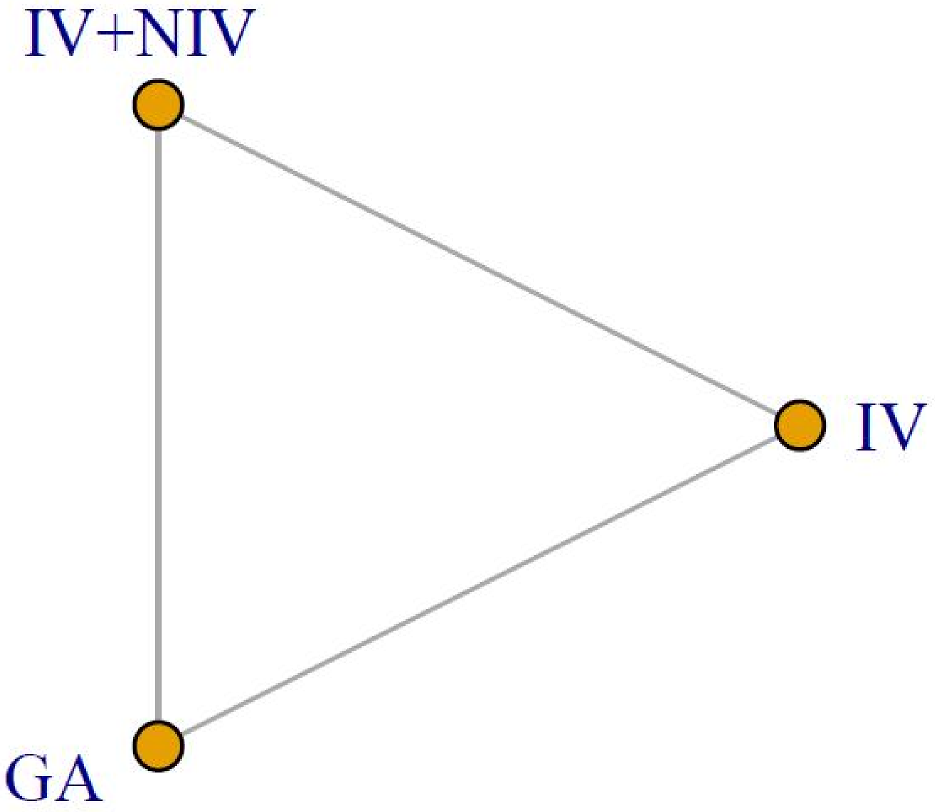

3.37 CD4(Anesthesia,before over)

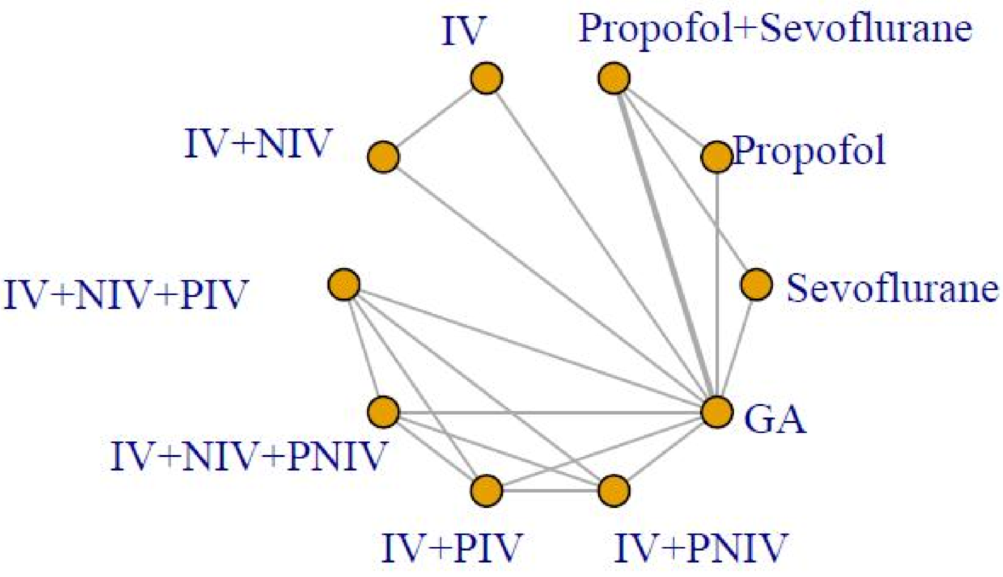

3.38 CD4(Anesthesia,over)

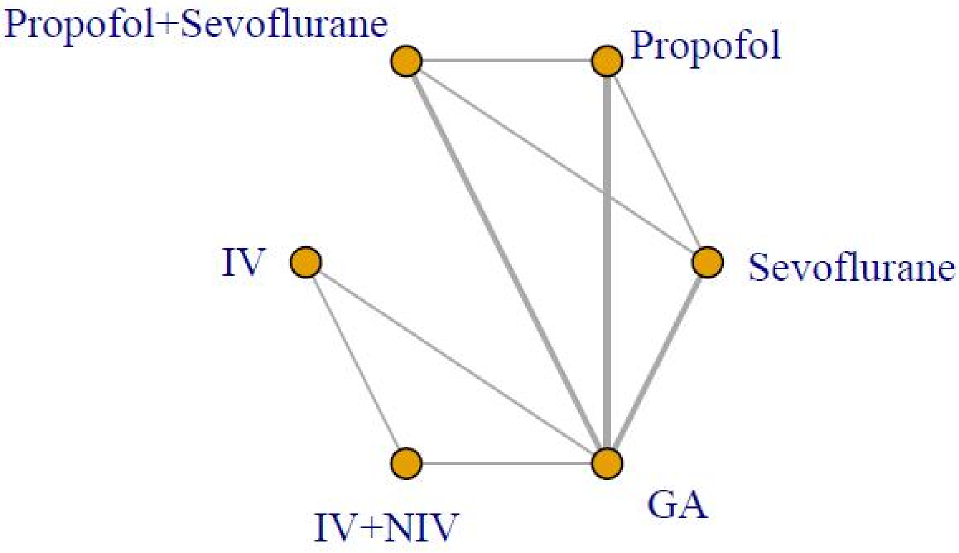

3.39 CD4(Anesthesia,12h)

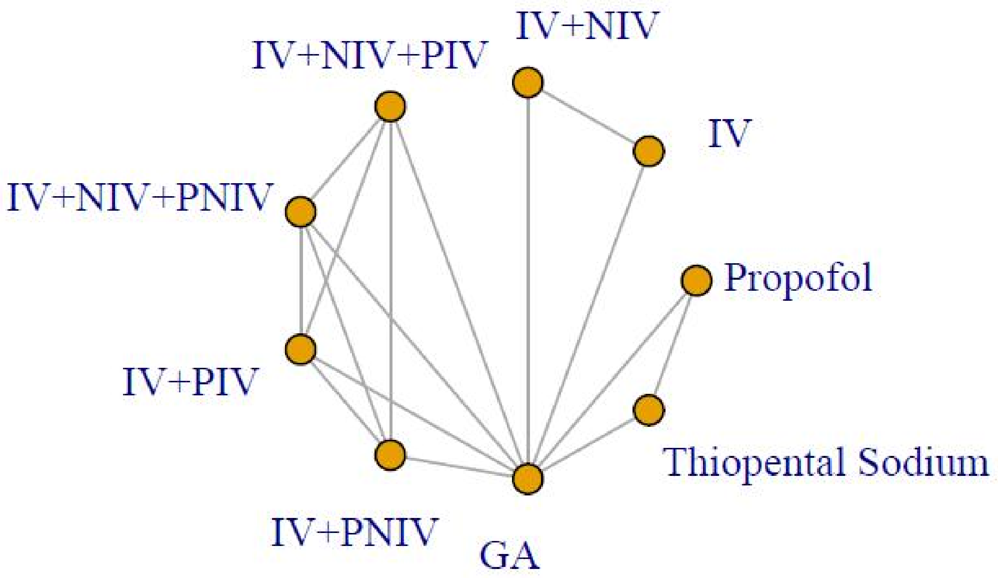

3.40 CD4(Anesthesia,1D)

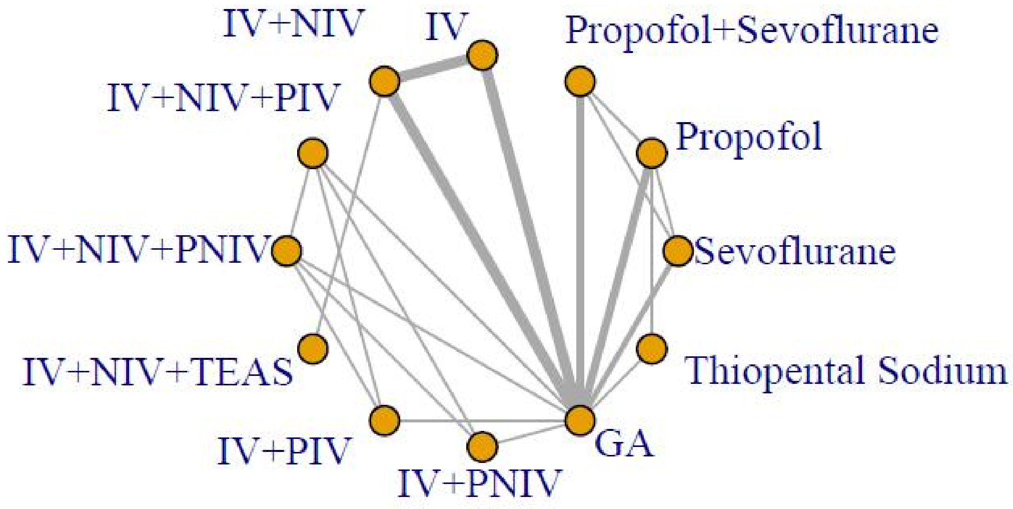

3.41 CD4(Anesthesia,2D)

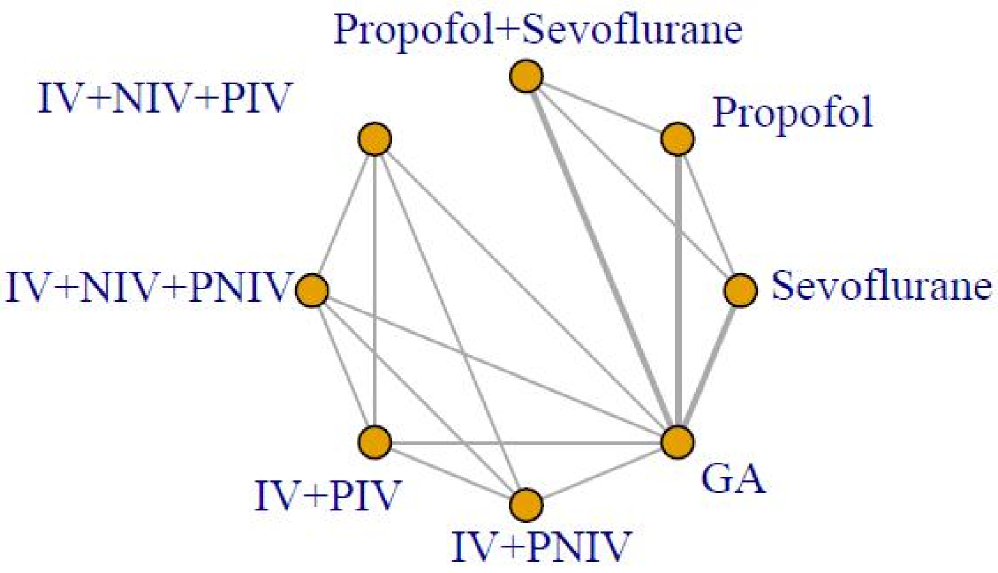

3.42 CD4(Anesthesia,3D)

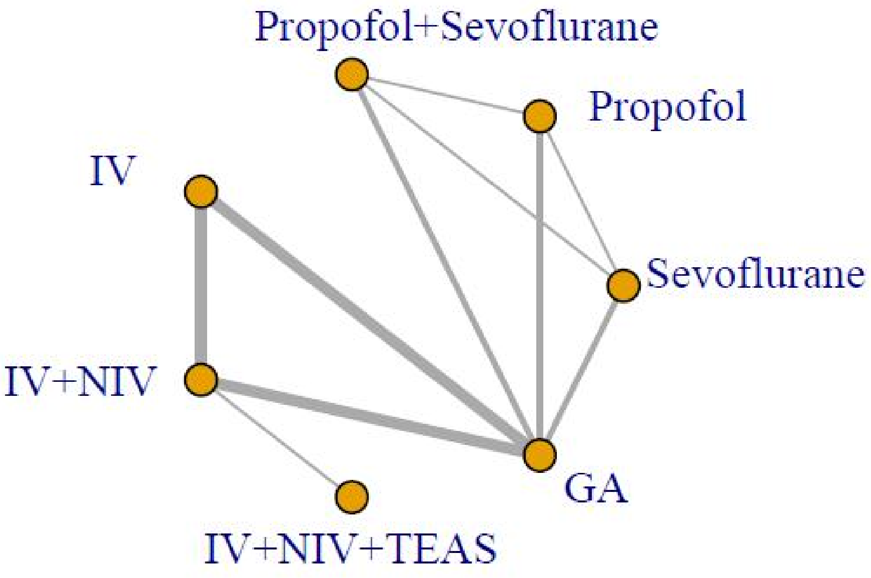

3.43 CD4(Anesthesia,5-7D)

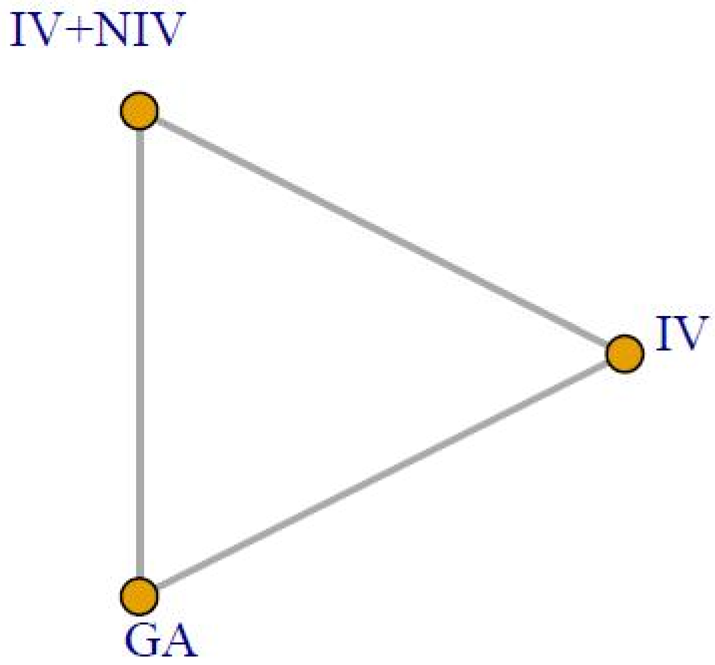

3.44 CD6(Anesthesia, before over)

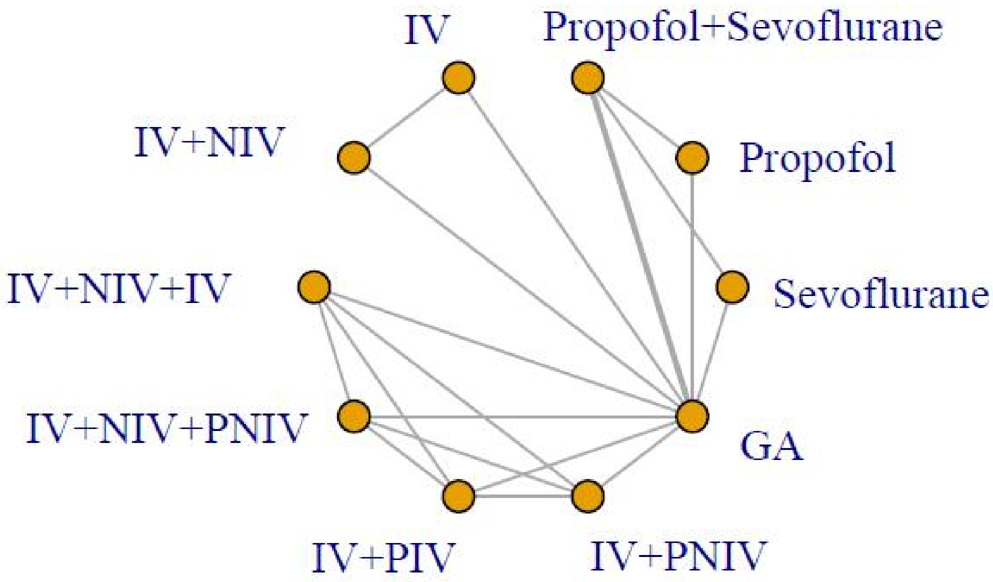

3.45 CD8(Anesthesia,over)

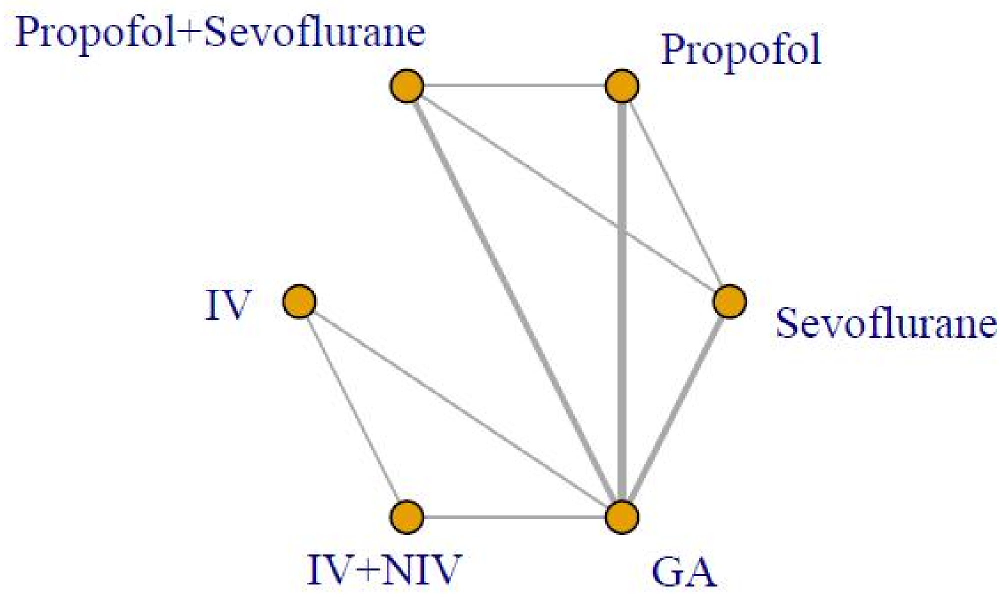

3.46CD8(Anesthesia,12h)

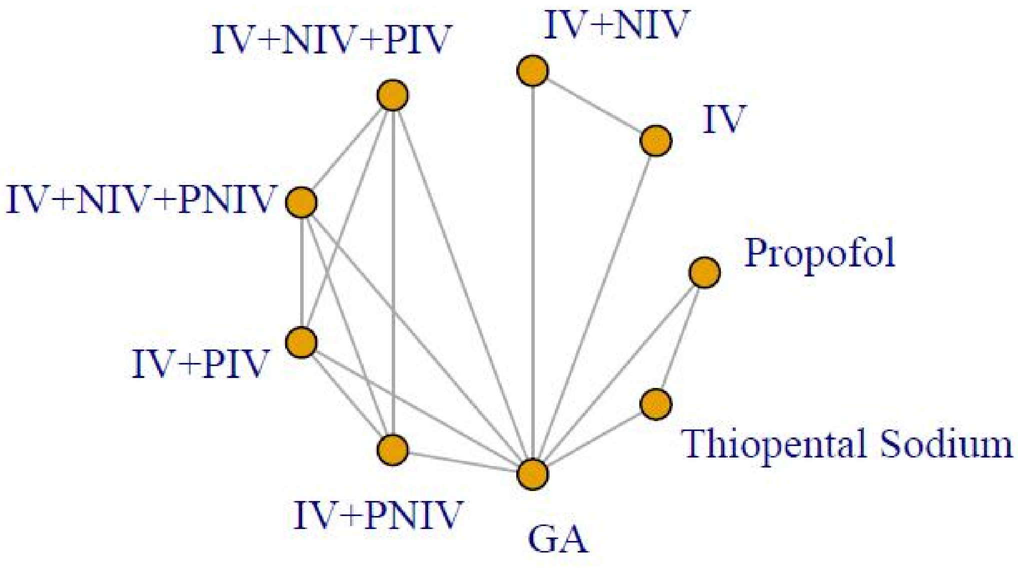

3.47CD8(Anesthesia,1D)

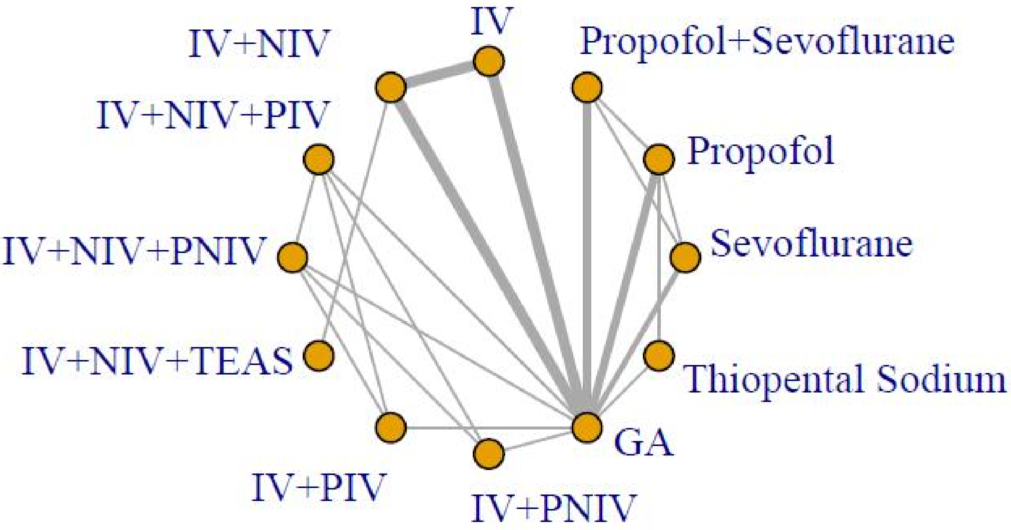

3.48CD8(Anesthesia,2D)

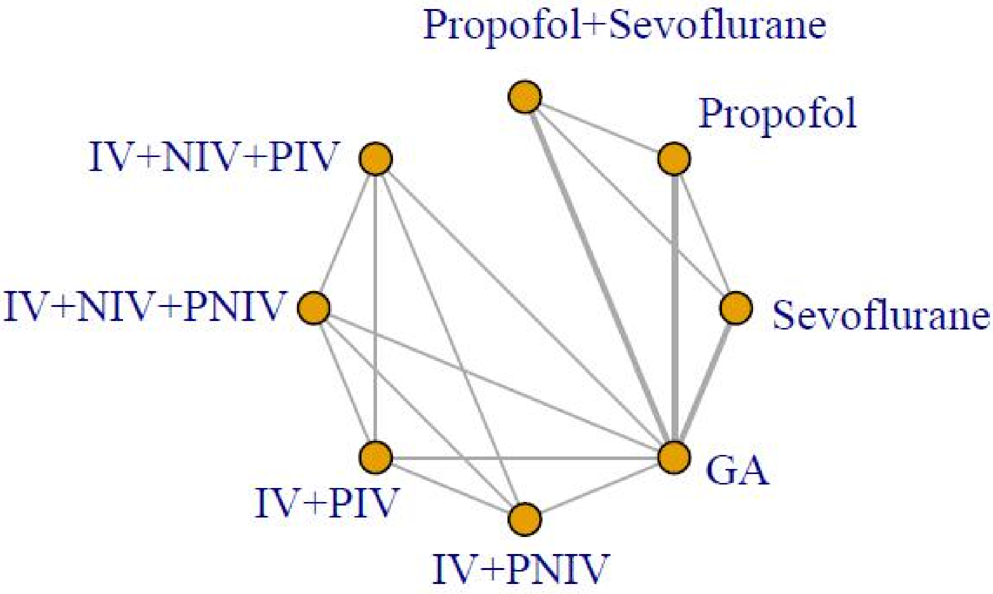

3.49CD8(Anesthesia,3D)

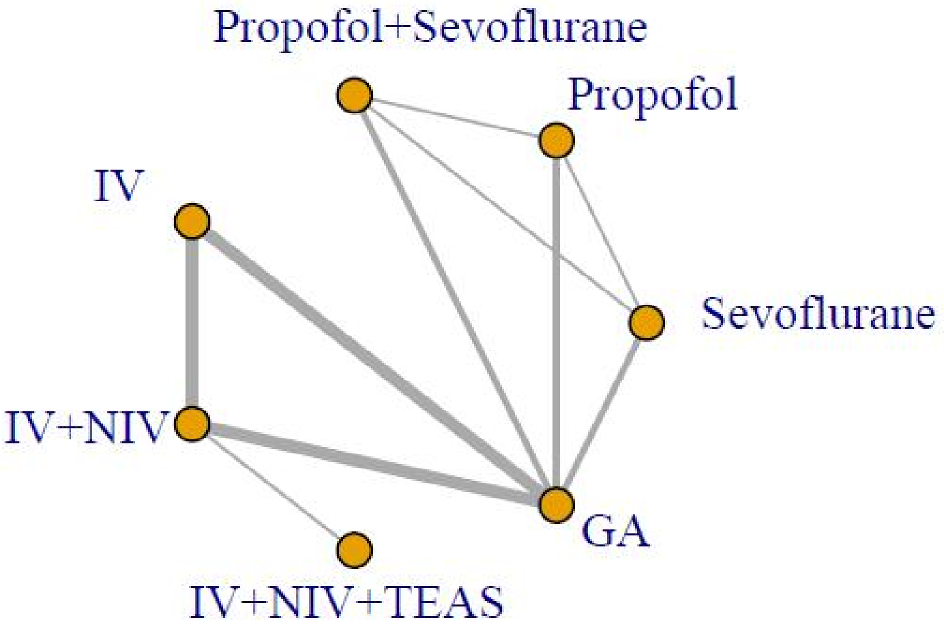

3.50 CD8(Anesthesia,5-7D)

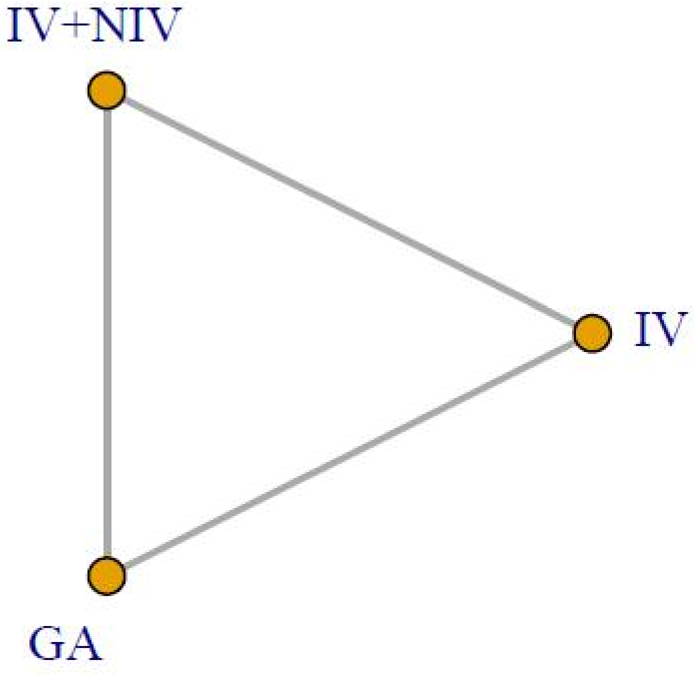

3.51 CD4/CD8(Anesthesia,before over)

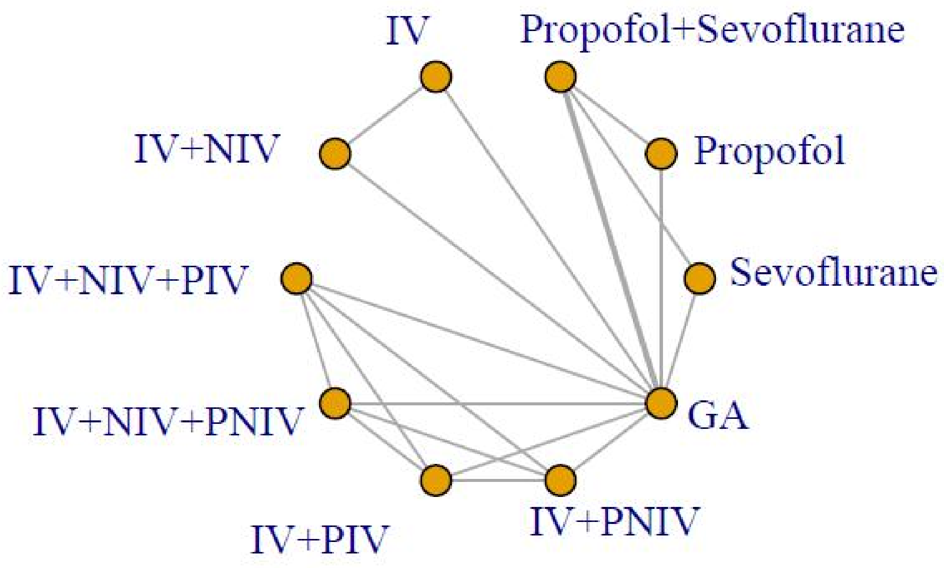

3.52 CD4/CD8(Anesthesia,over)

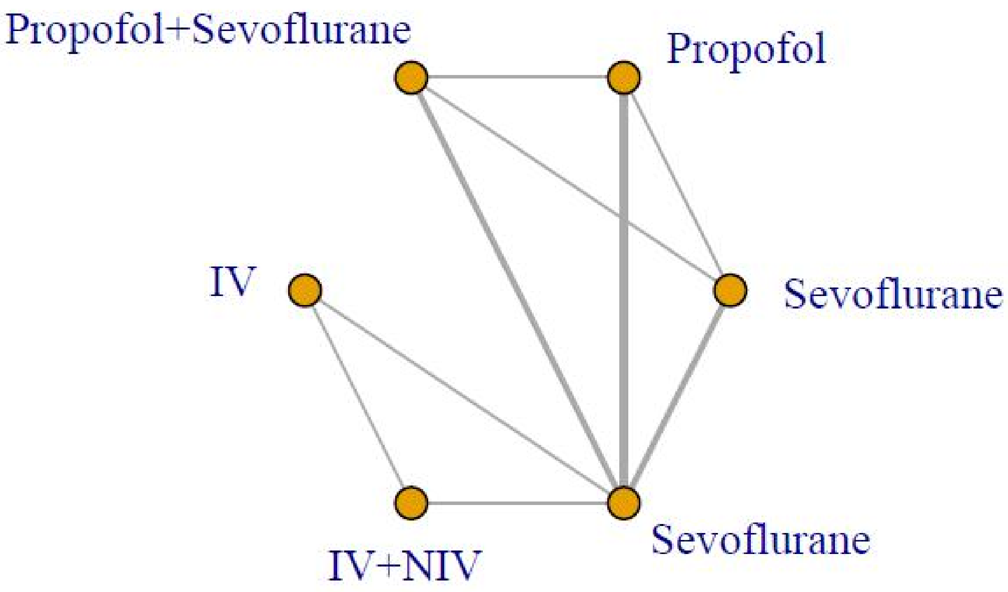

3.53 CD4/CD8(Anesthesia,12h)

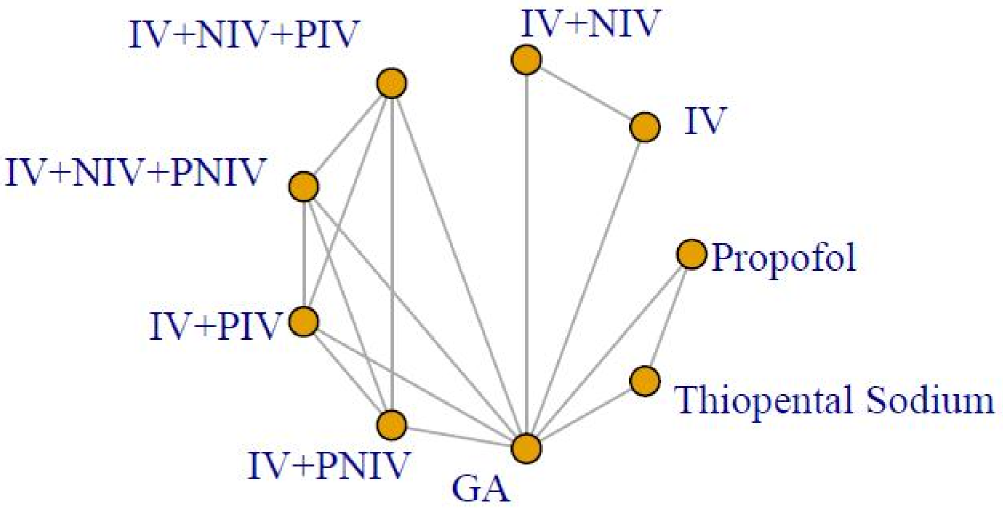

3.54 CD4/CD8(Anesthesia,1D)

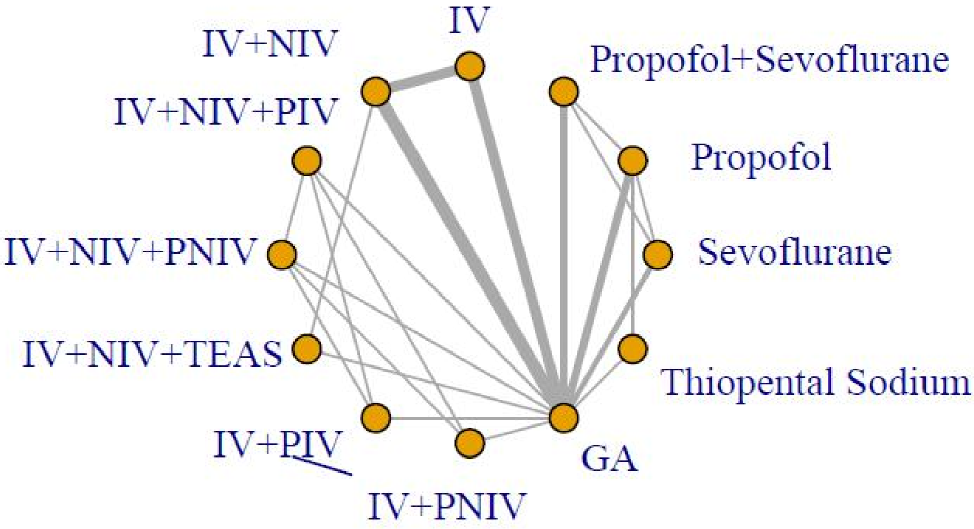

3.55 CD4/CD8(Anesthesia,2D)

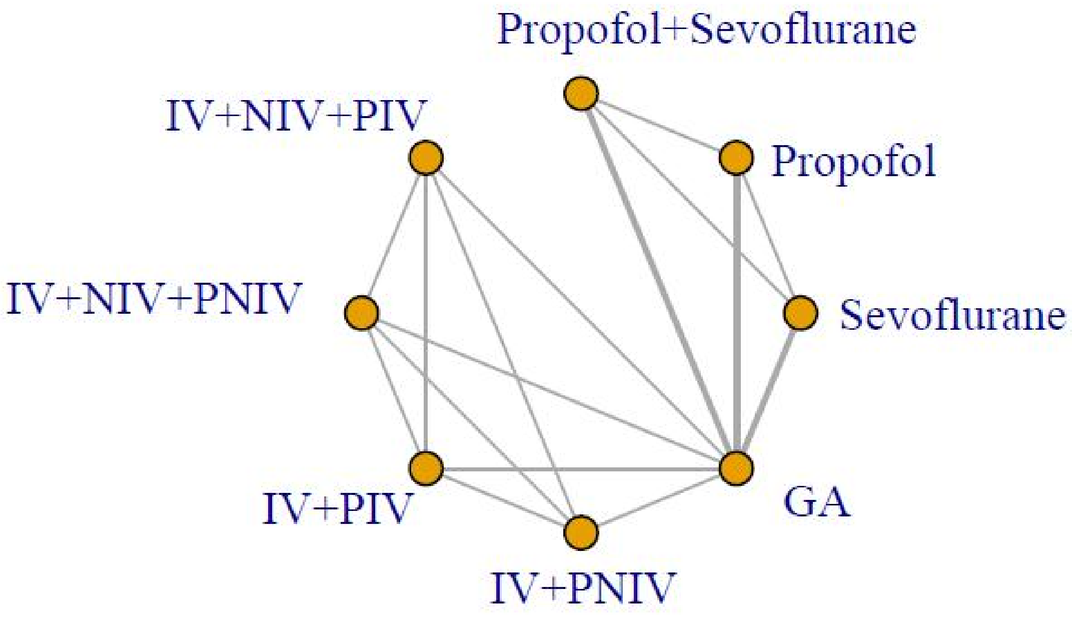

3.56 CD4/CD8(Anesthesia,3D)

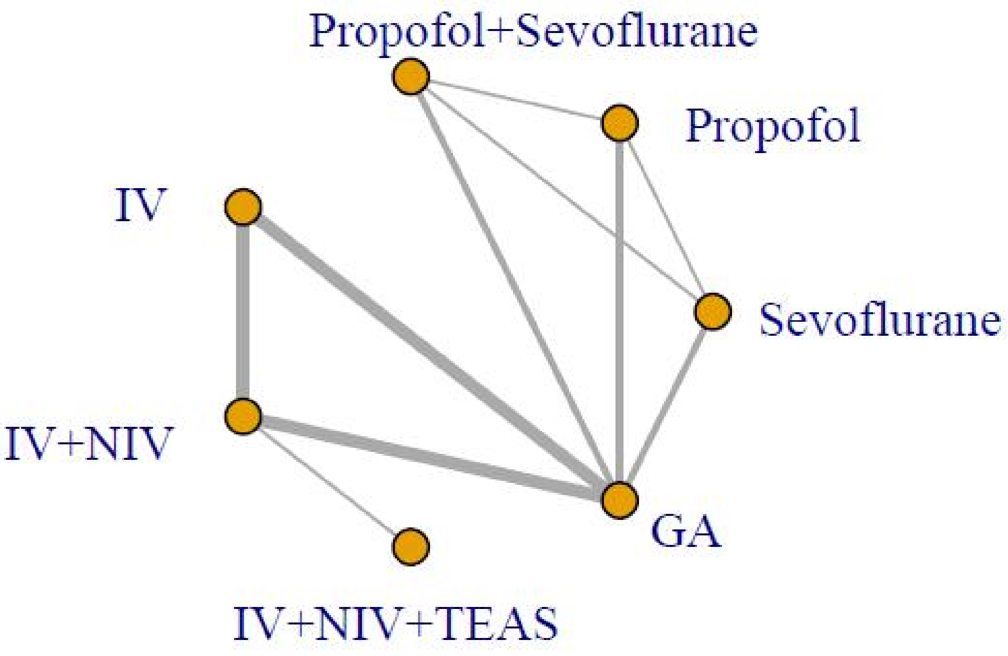

3.57 CD4/CD8(Anesthesia,5-7D)

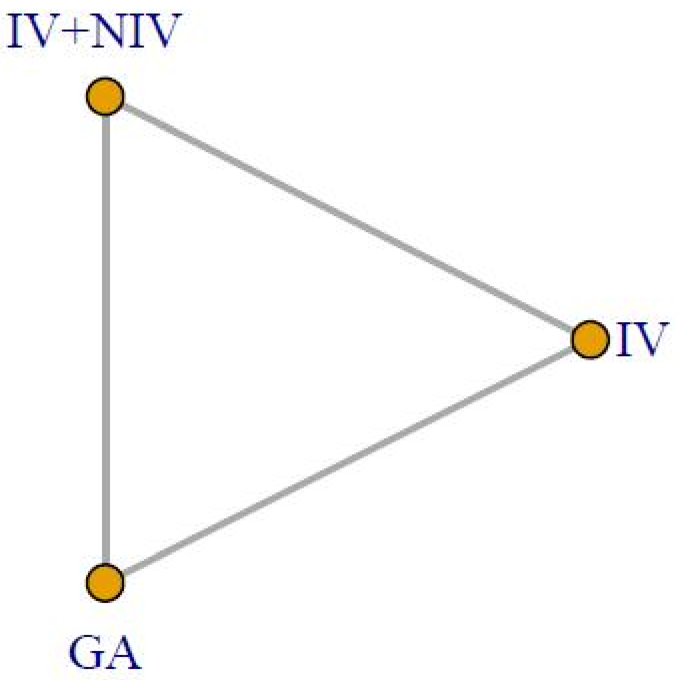

3.58 CD3(Analgesia,before over)

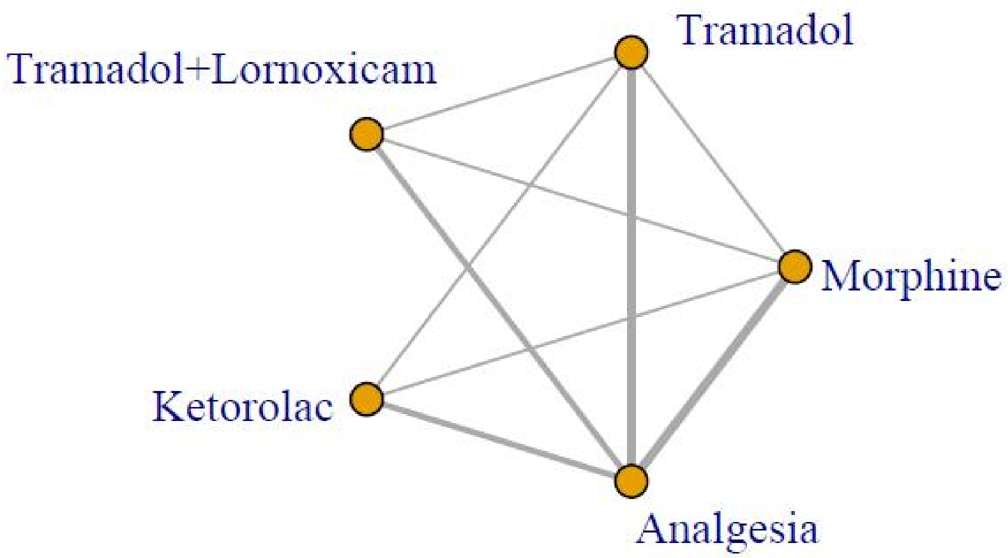

3.59 CD3(Analgesia,over)

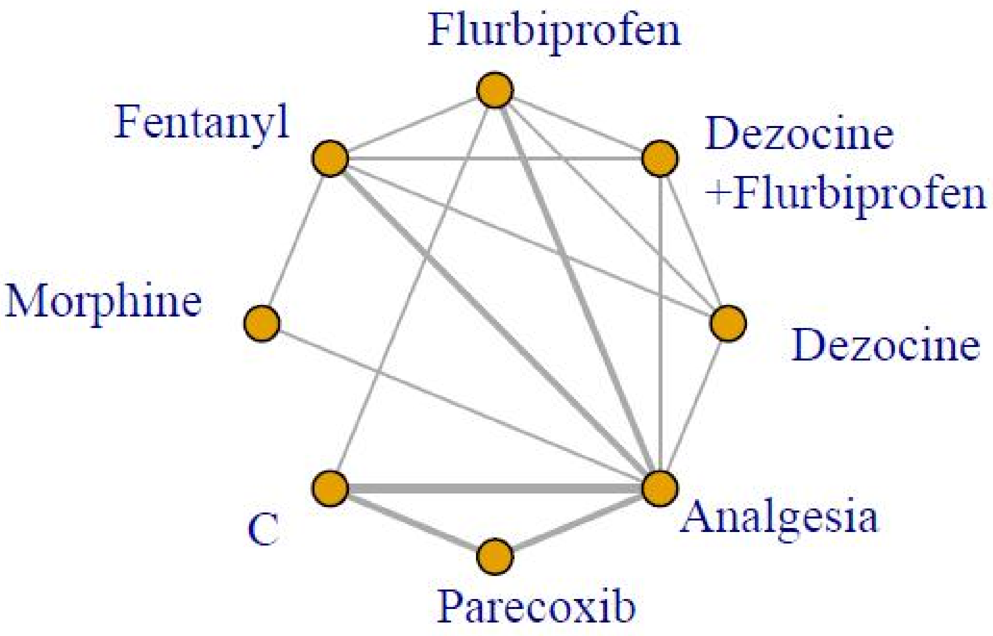

3.60 CD3(Analgesia,12h)

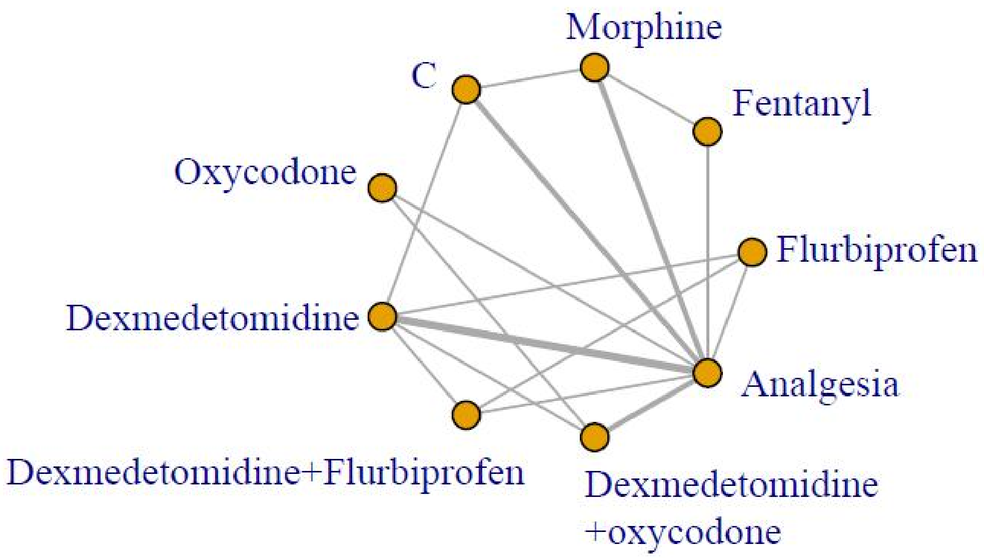

3.61 CD3(Analgesia,1D)

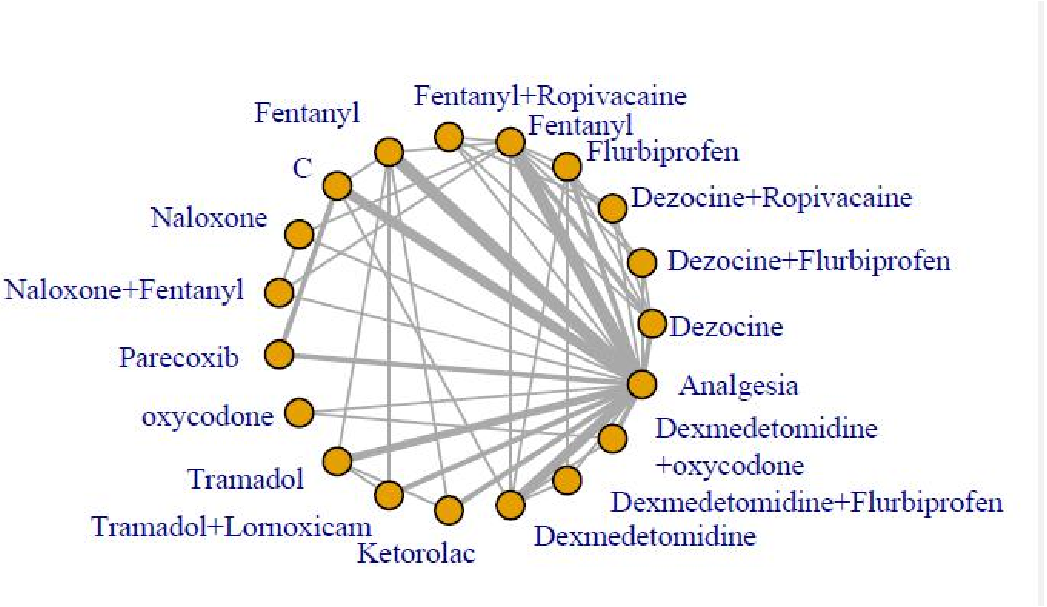

3.62 CD3(Analgesia,2D)

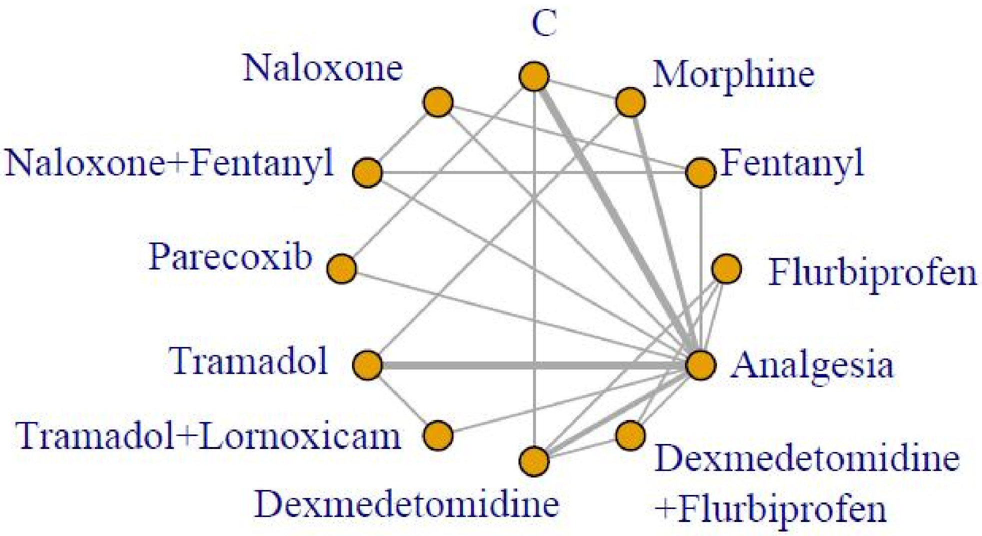

3.63 CD3(Analgesia,3D)

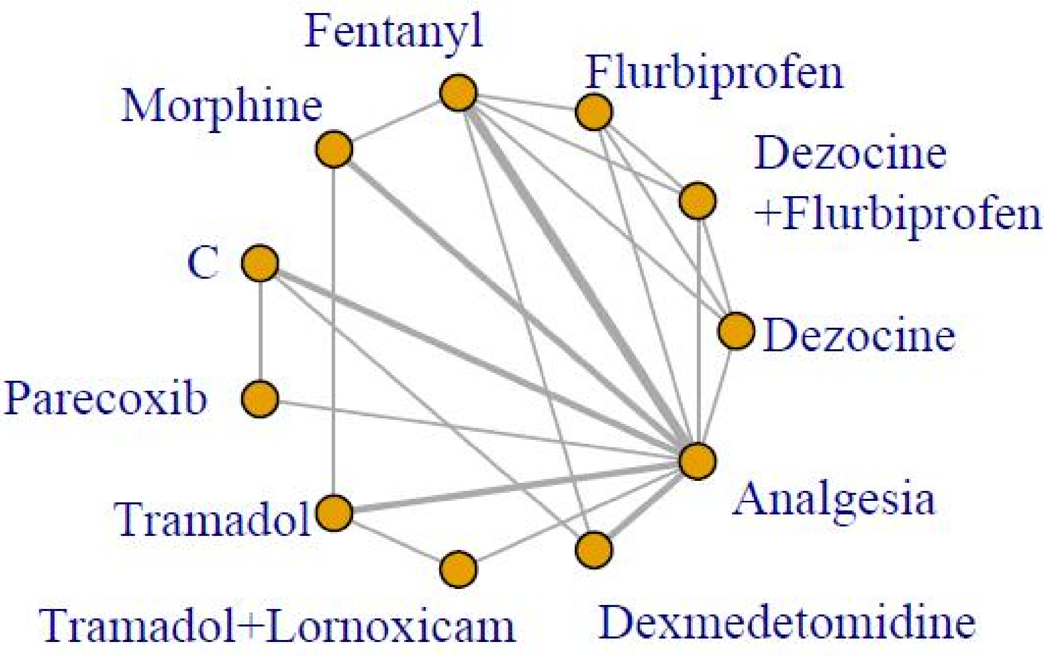

3.64 CD3(Analgesia,5-7D)

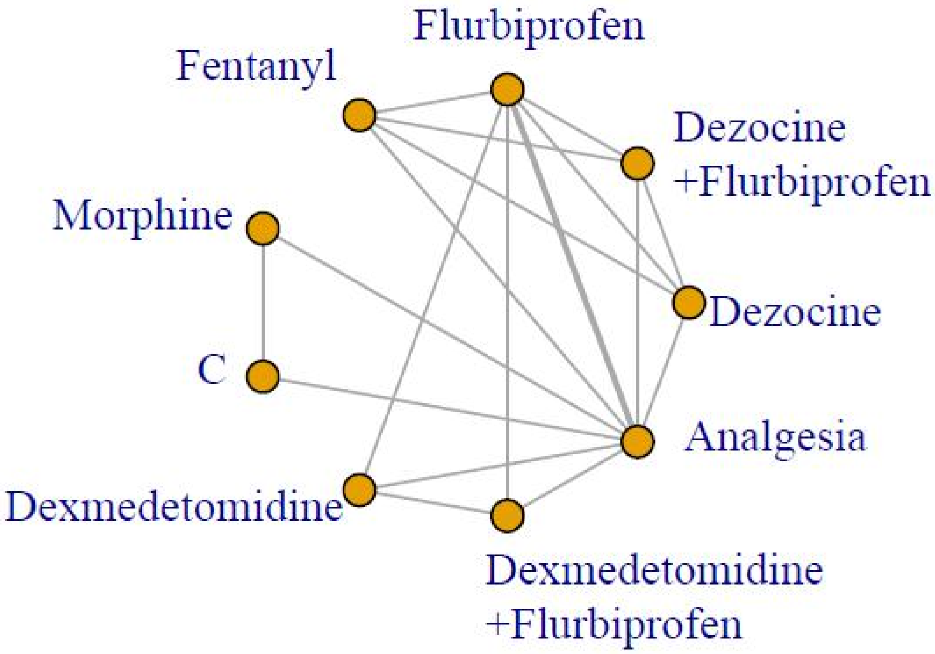

3.65 CD4(Analgesia,before over)

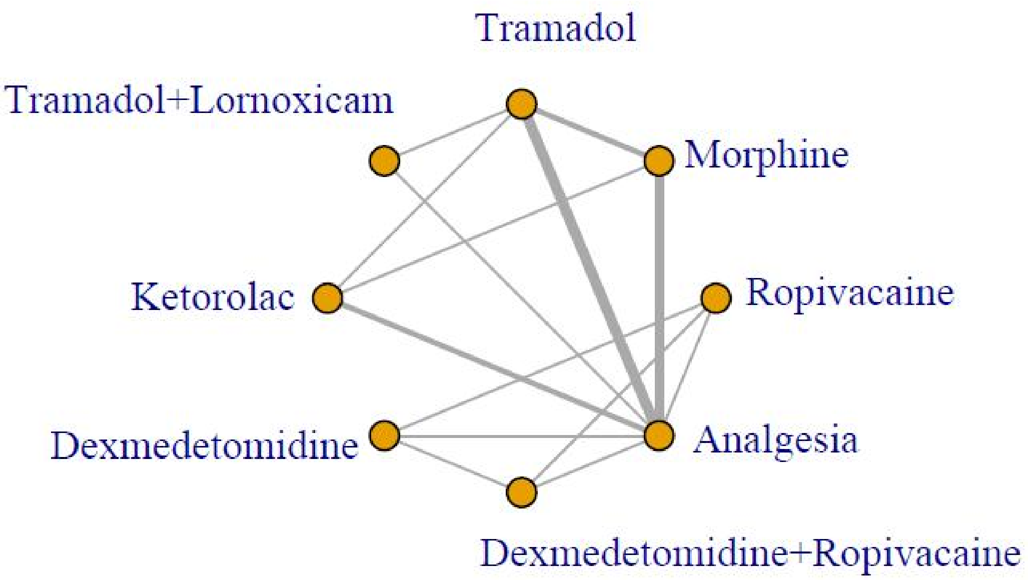

3.66 CD4(Analgesia,over)

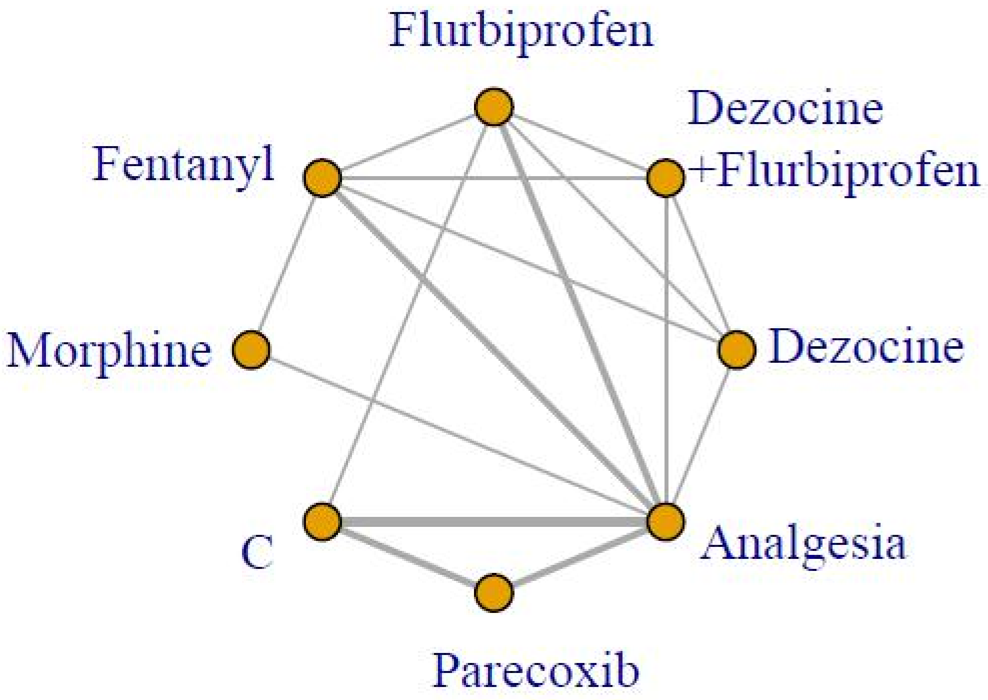

3.67 CD4(Analgesia,12h)

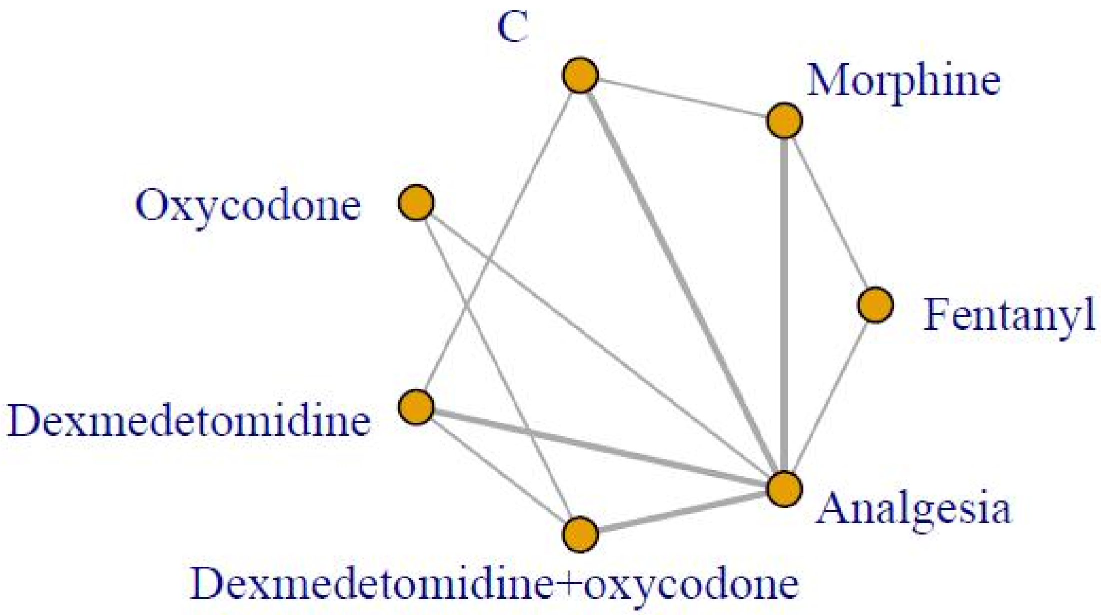

3.68 CD4(Analgesia,1D)

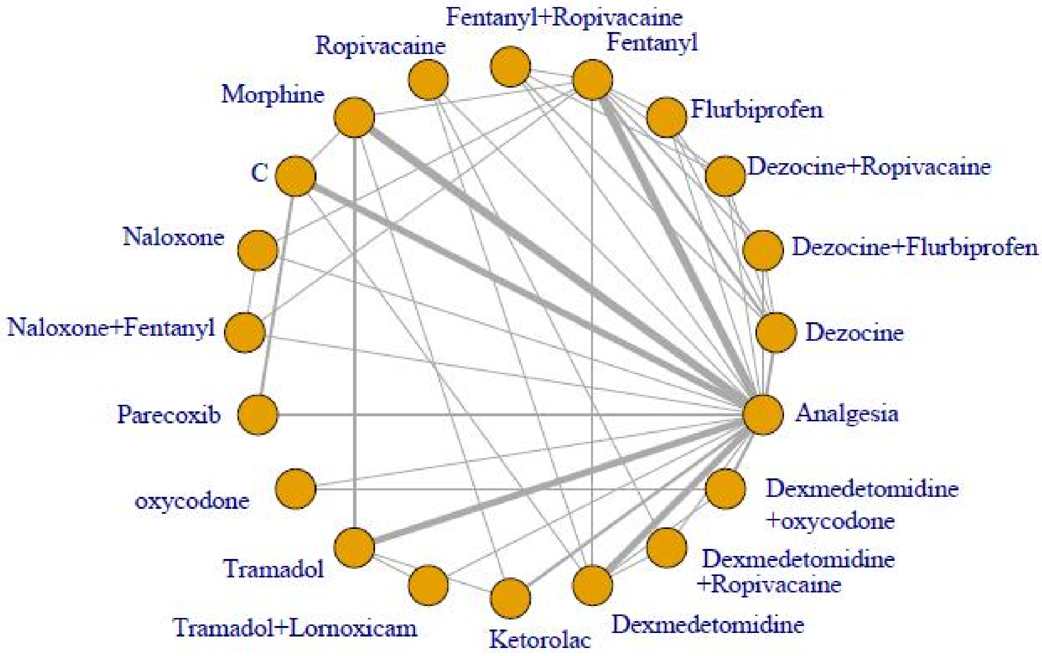

3.69 CD4(Analgesia,2D)

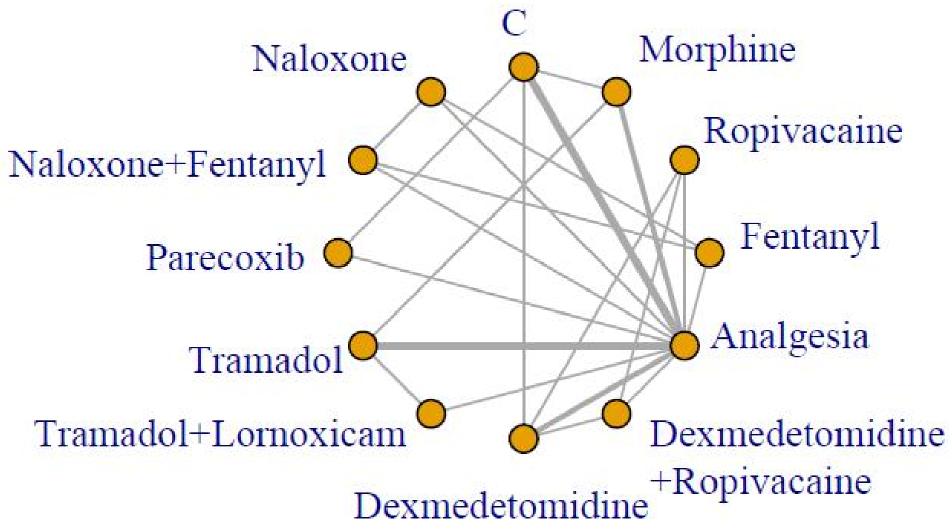

3.70 CD4(Analgesia,3D)

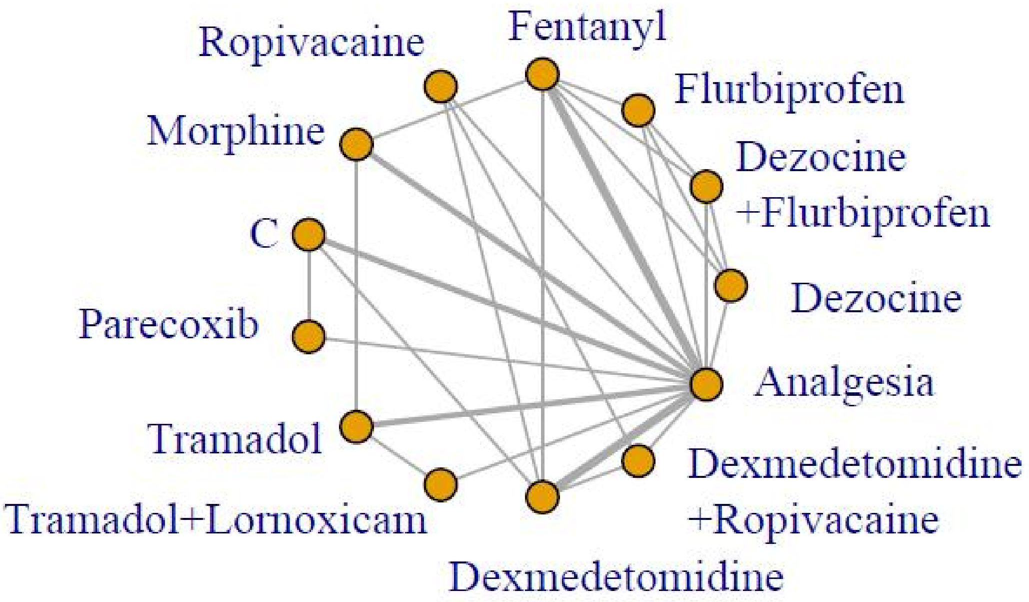

3.71 CD4(Analgesia,5-7D)

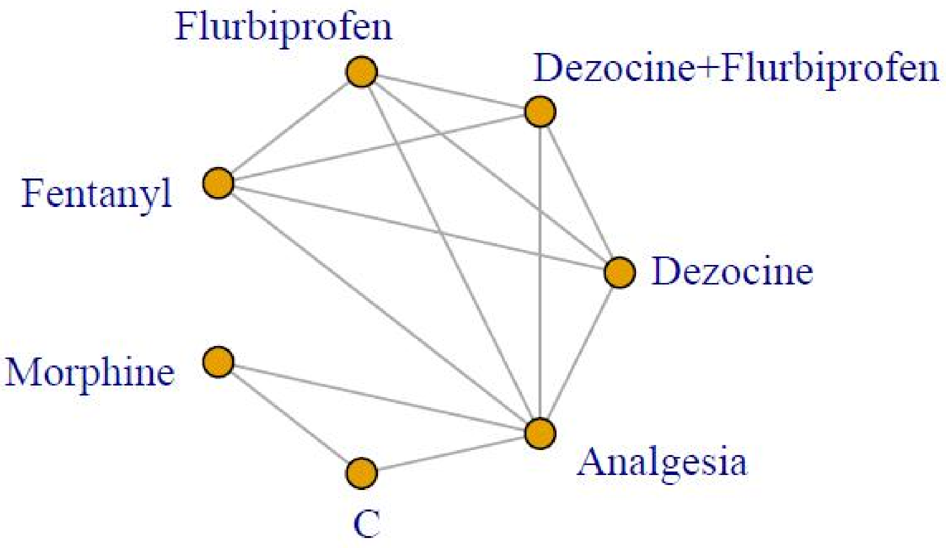

3.72 CD8(Analgesia,before over)

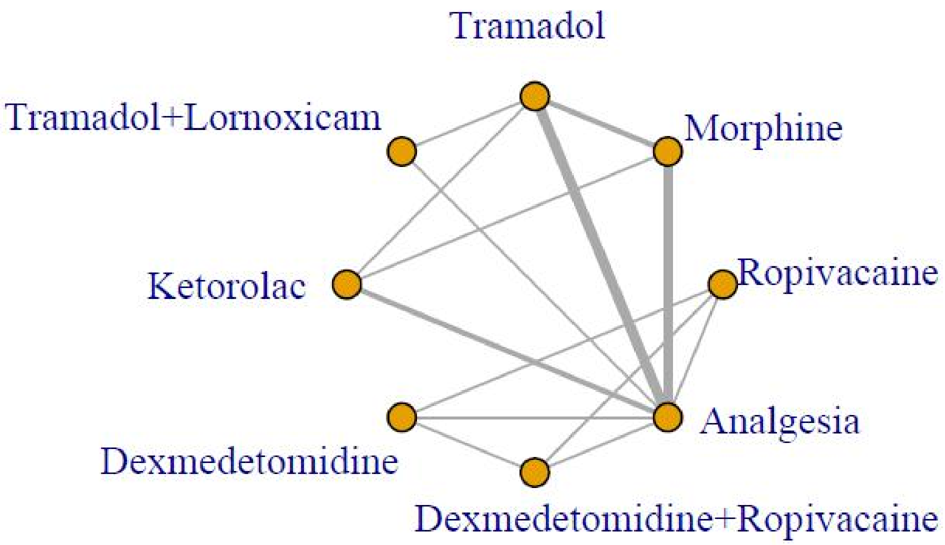

3.73 CD8(Analgesia,over)

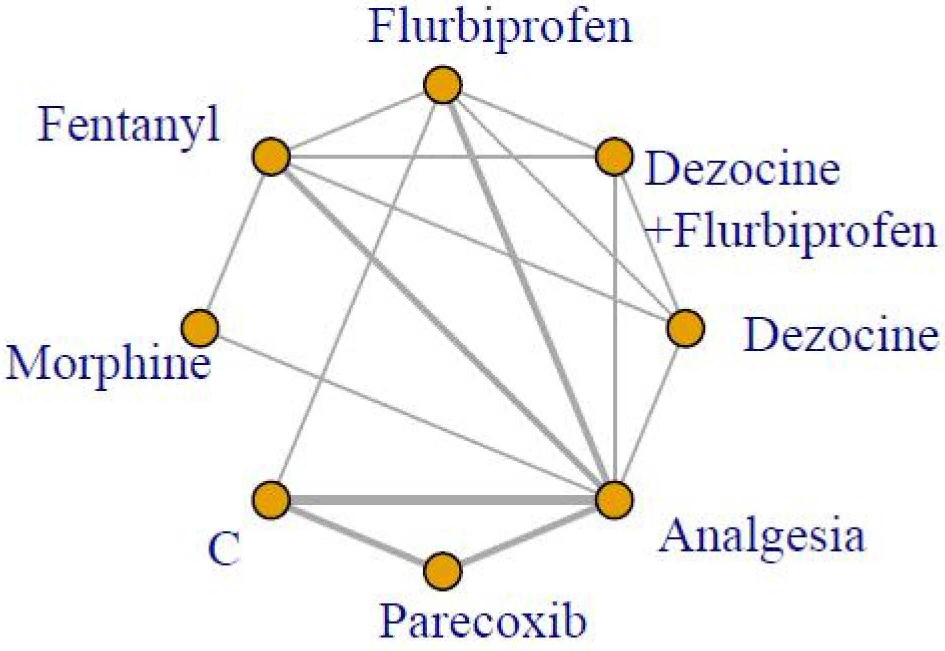

3.74 CD8(Analgesia,12h)

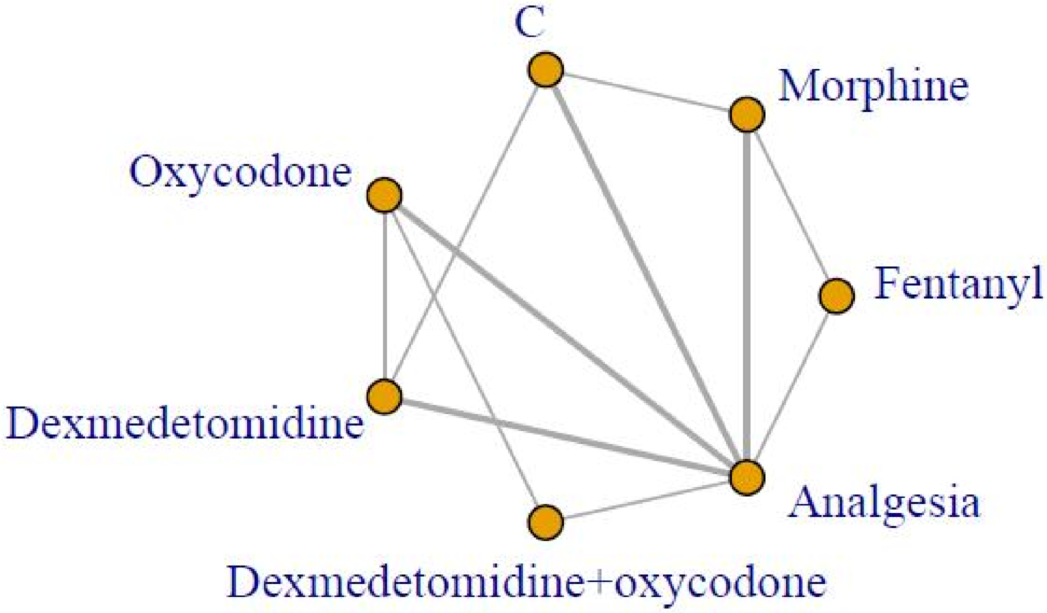

3.75 CD8(Analgesia,1D)

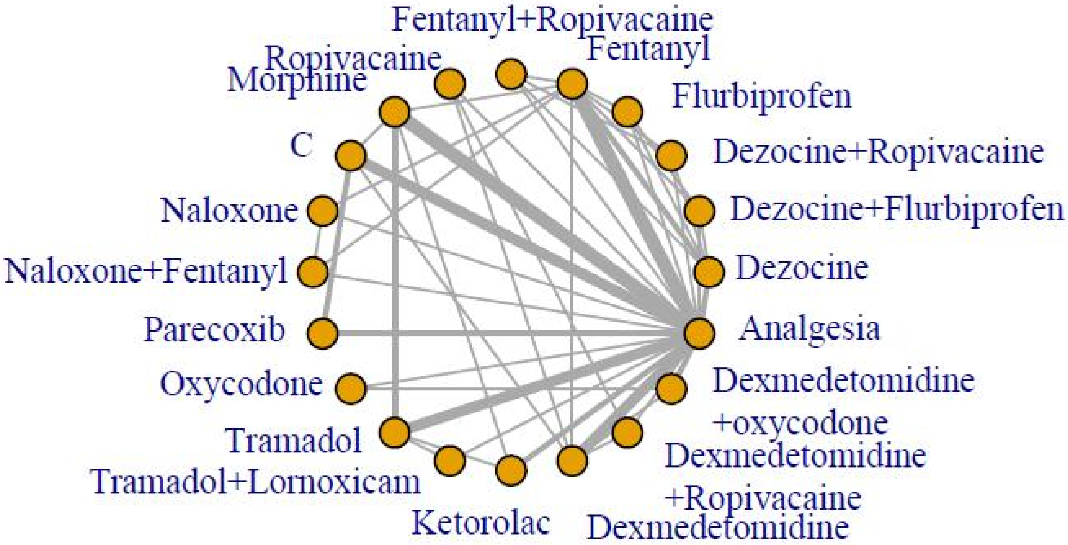

3.76 CD8(Analgesia,2D)

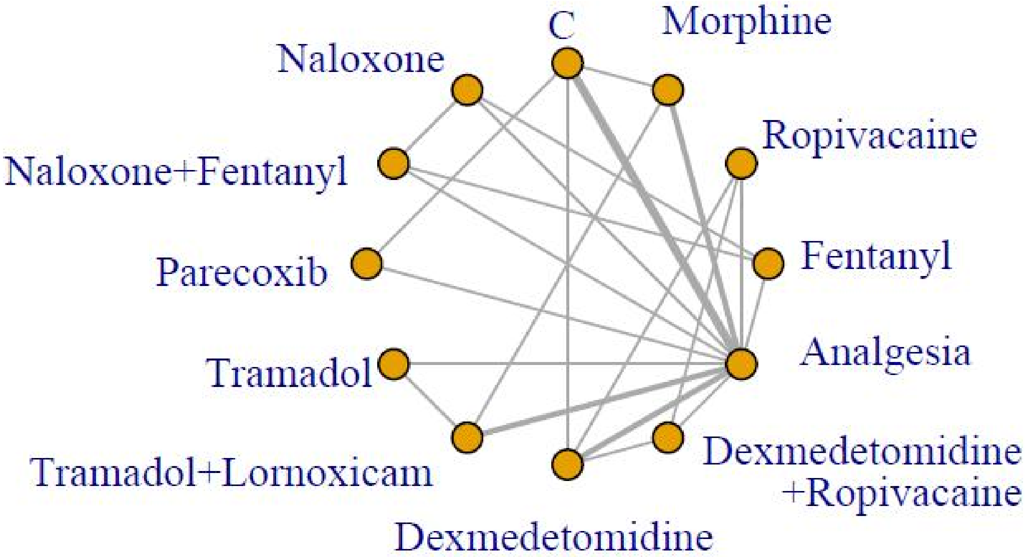

3.77 CD8(Analgesia,3D)

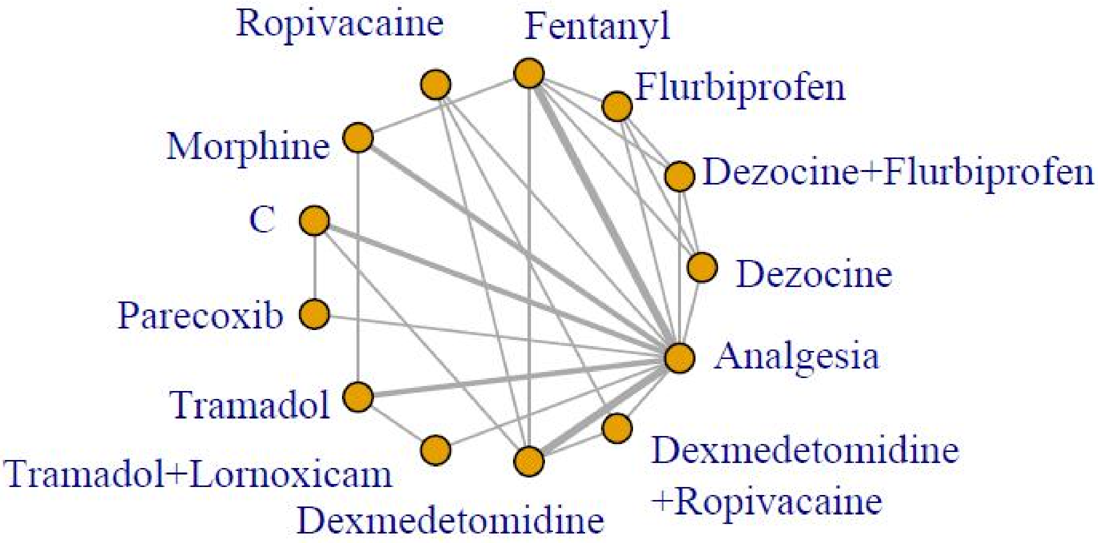

3.78 CD8(Analgesia,5-7D)

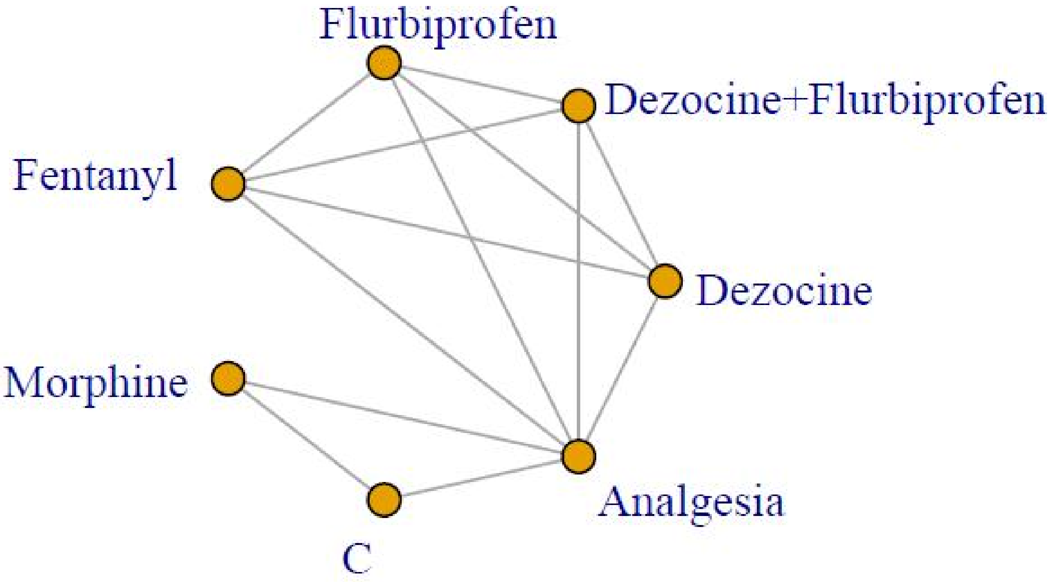

3.79 CD4/CD8(Analgesia,before over)

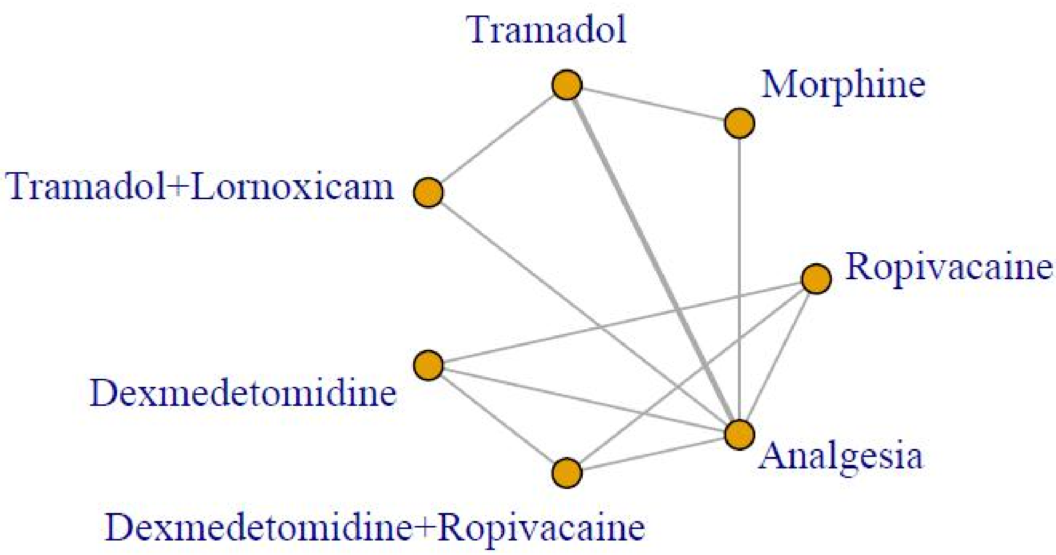

3.80 CD4/CD8(Analgesia,over)

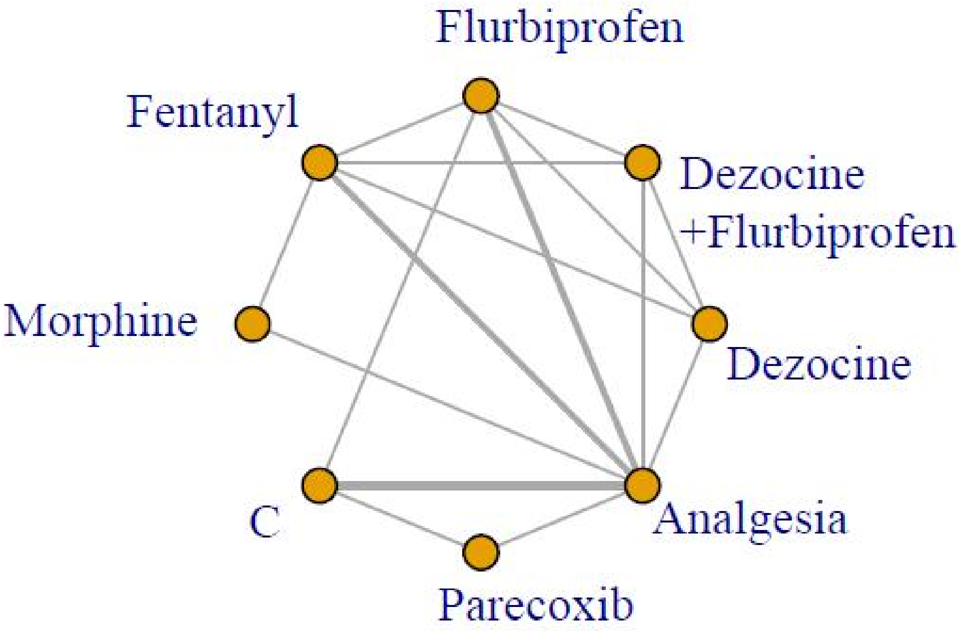

3.81 CD4/CD8(Analgesia,12h)

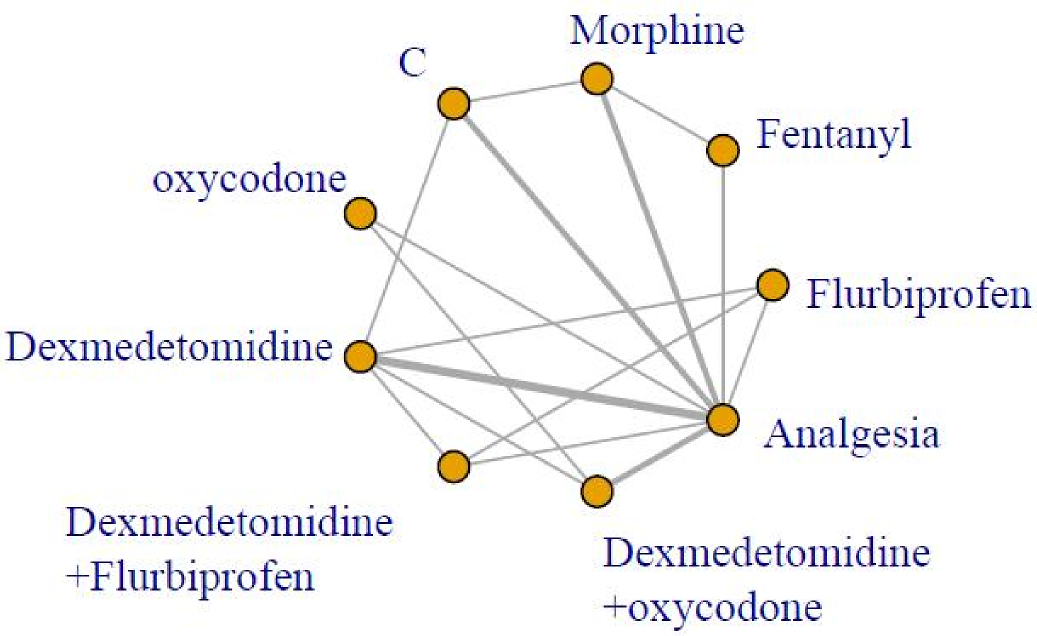

3.82 CD4/CD8(Analgesia,1D)

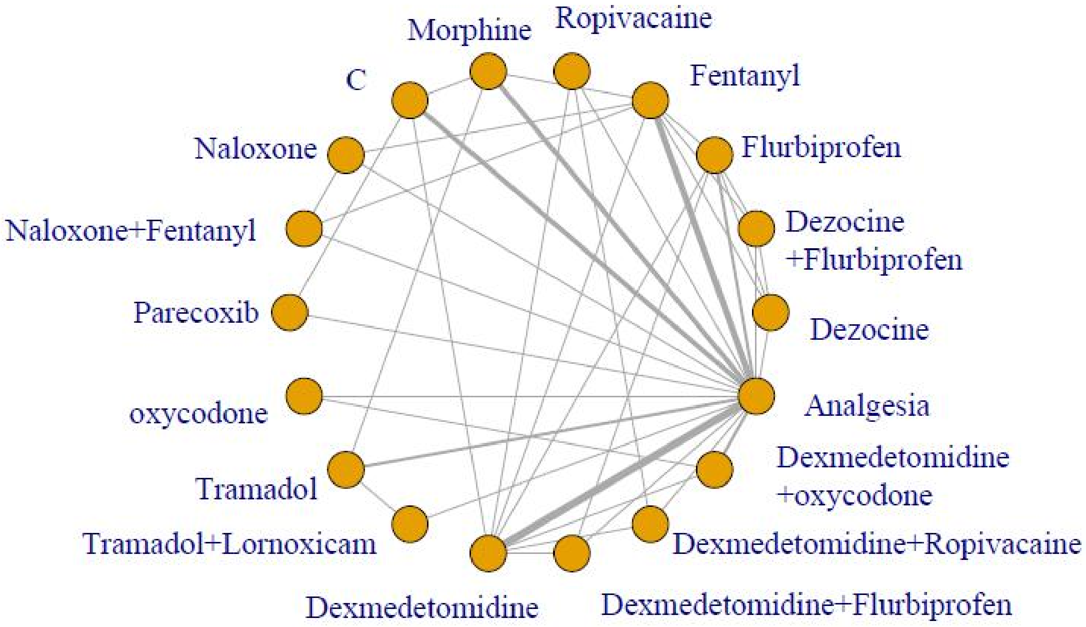

3.83 CD4/CD8(Analgesia,2D)

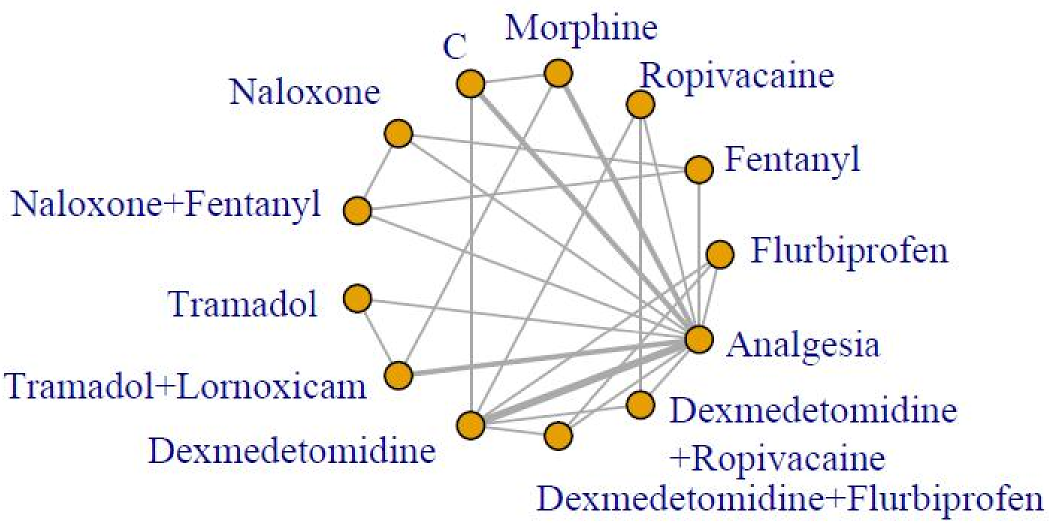

3.84 CD4/CD8(Analgesia,3D)

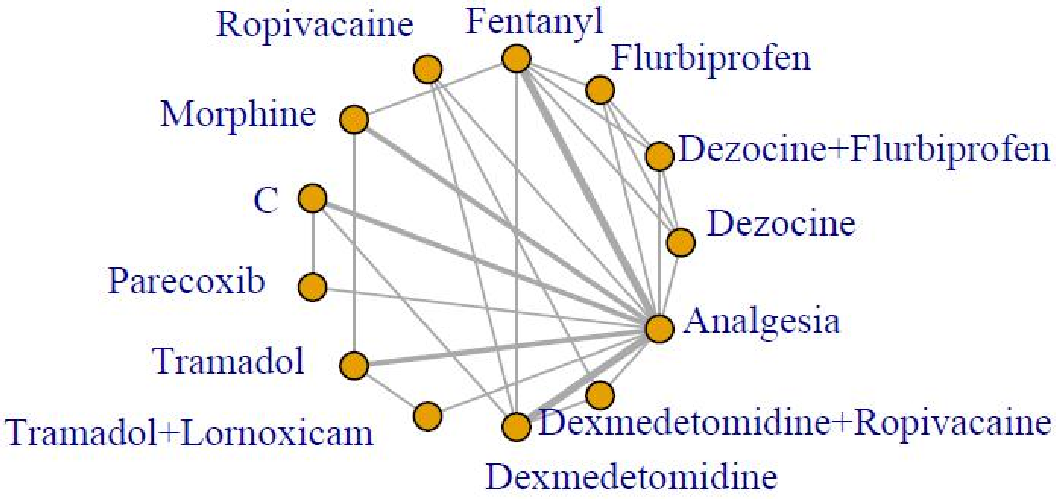

3.85 CD4/CD8(Analgesia,5-7D)

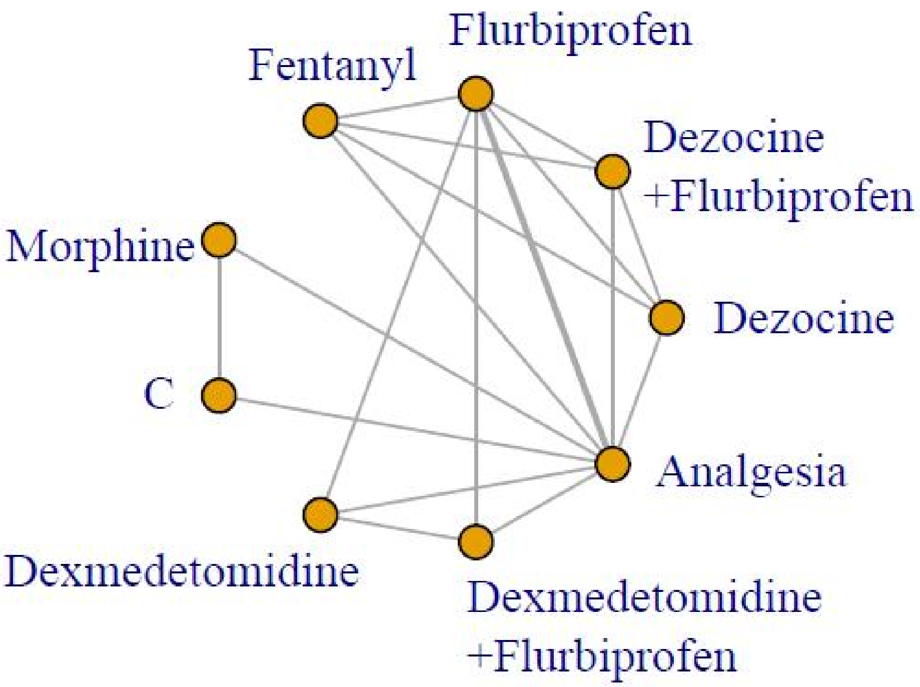

3.86 CD3(Others,1D)

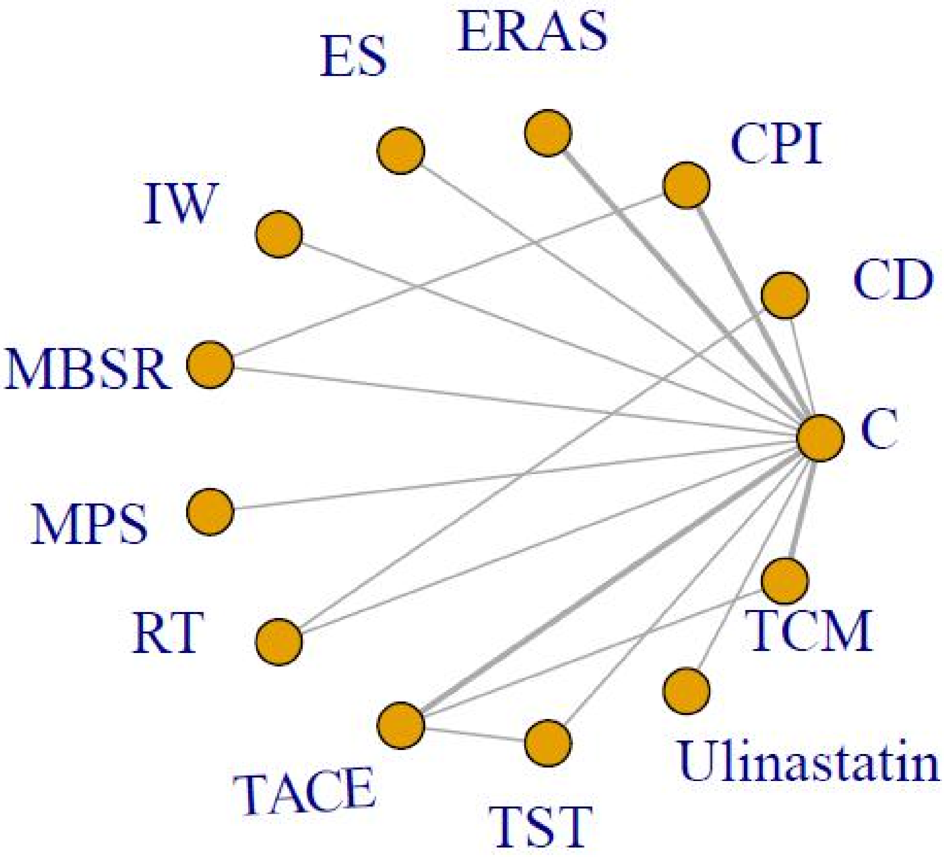

3.87 CD3(Others,3D)

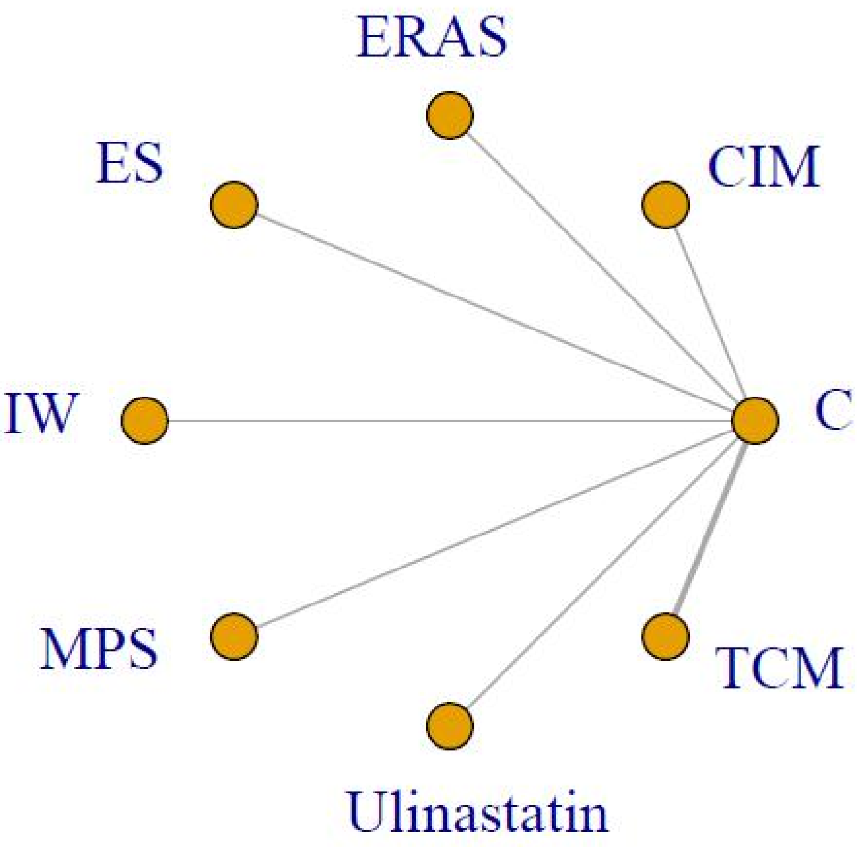

3.88 CD3(Others,>3D)

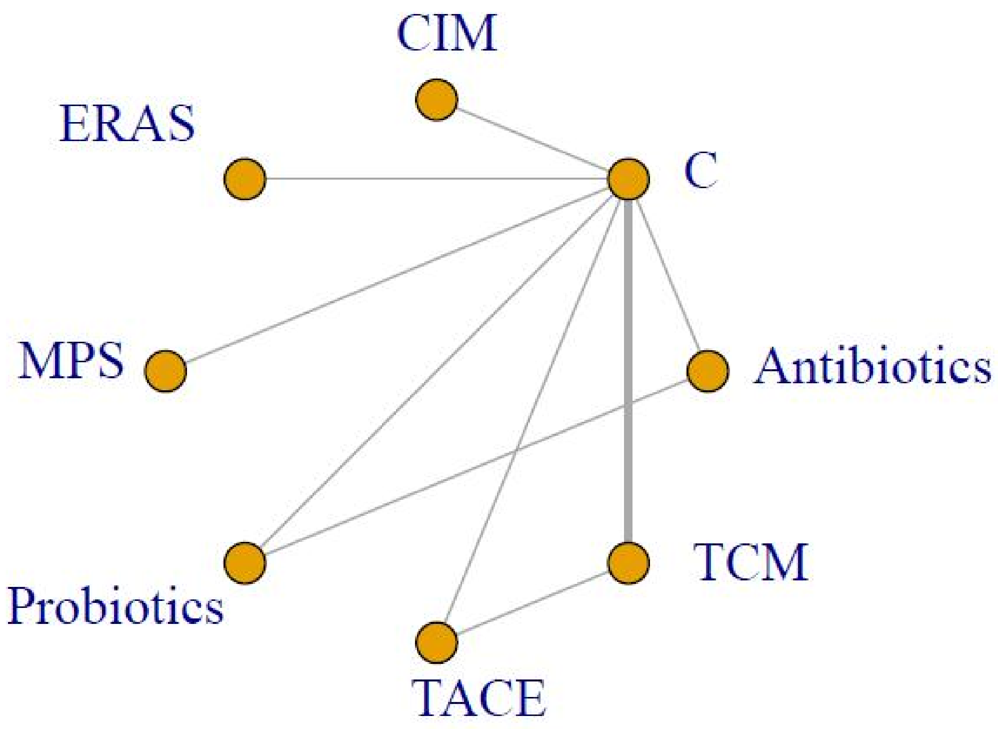

3.89 CD4(Others,1D)

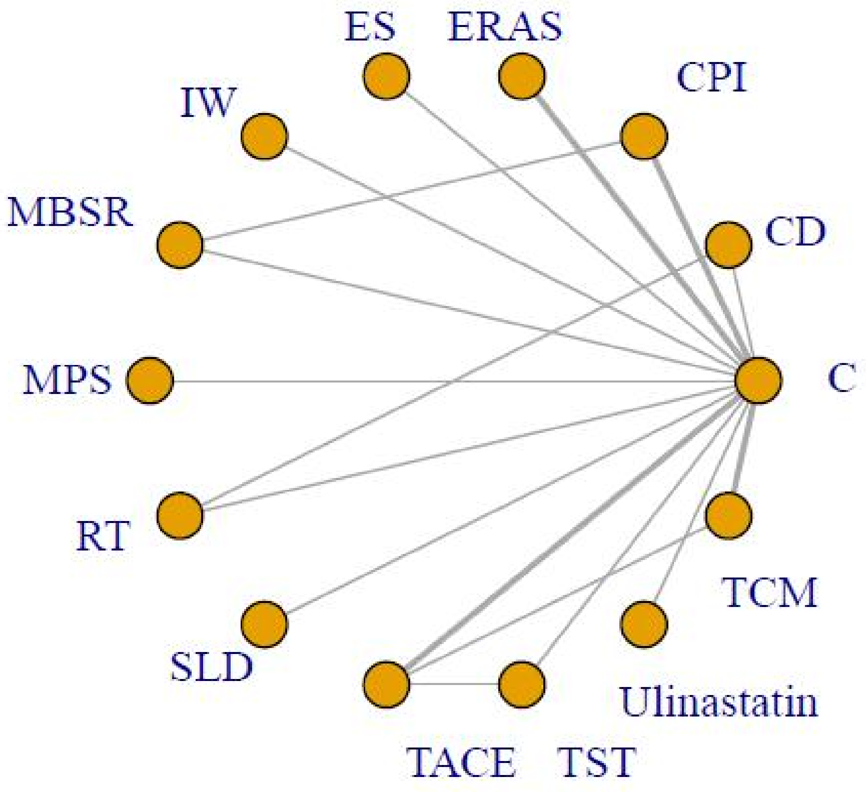

3.90 CD4(Others,3D)

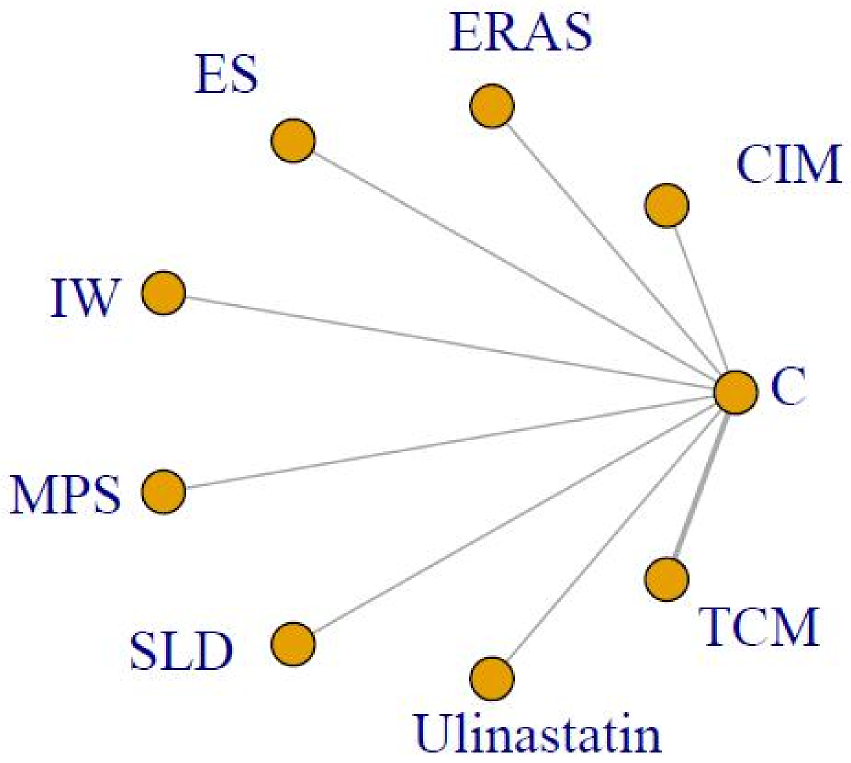

3.91 CD4(Others,>3D)

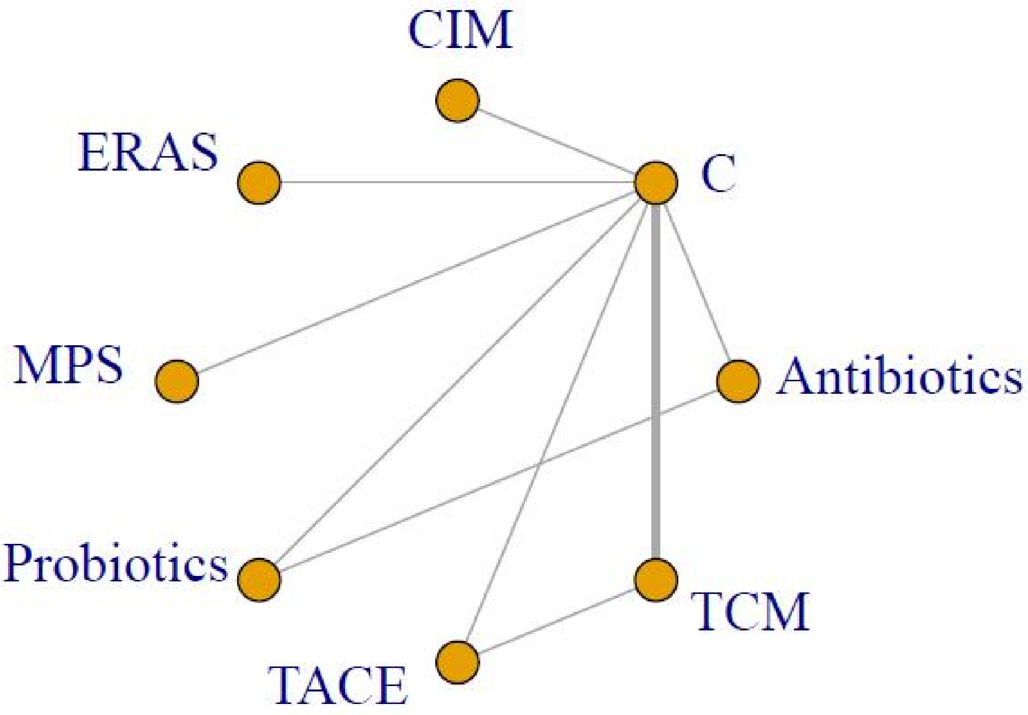

3.92 CD8(Others,1D)

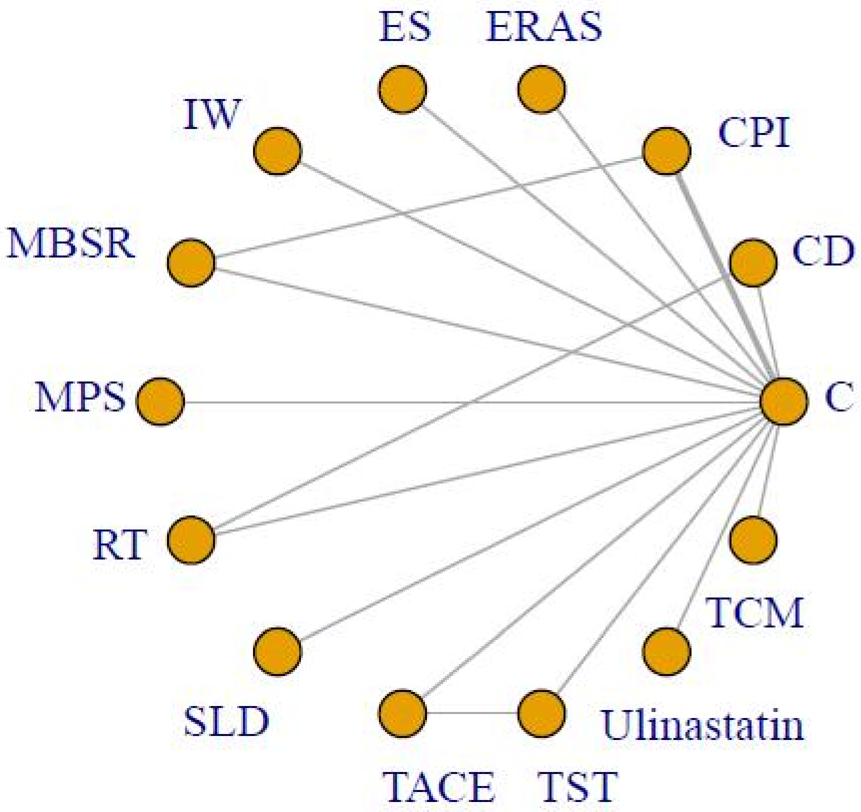

3.93 CD8(Others,3D)

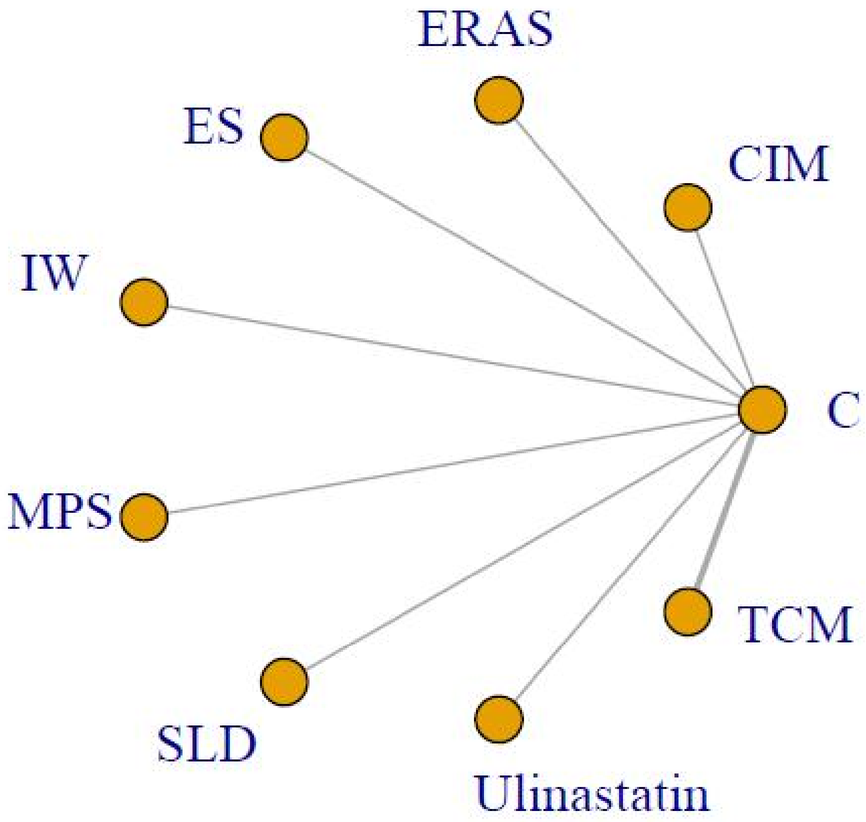

3.94 CD8(Others,>3D)

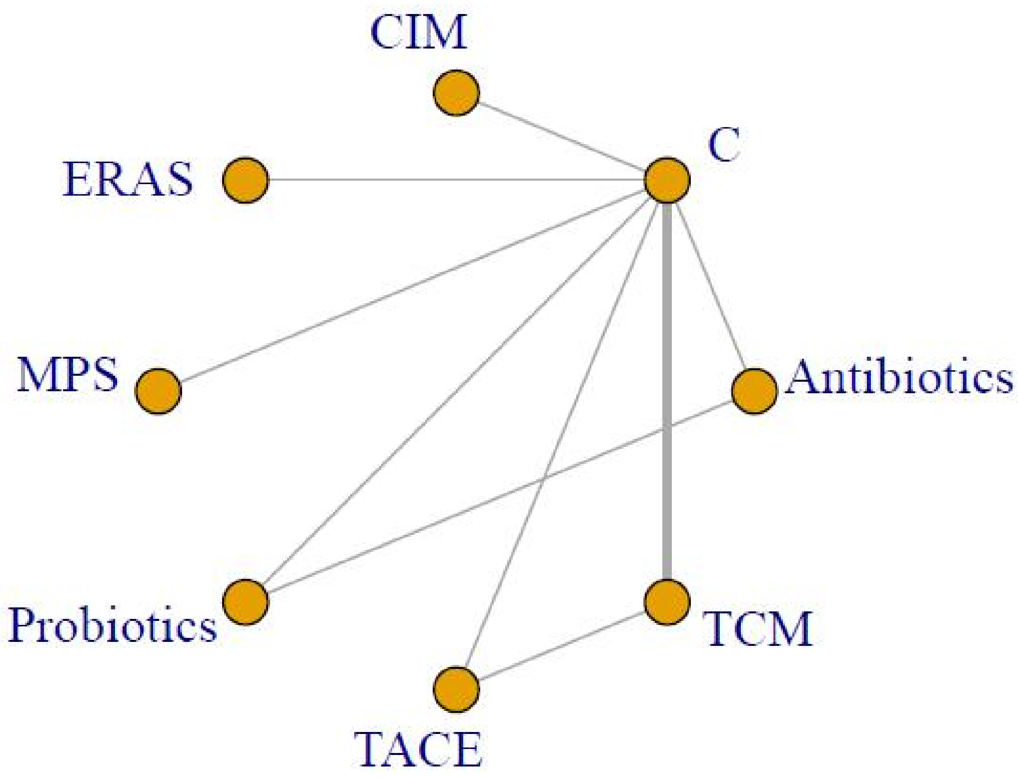

3.95 CD4/CD8(Others,1D)

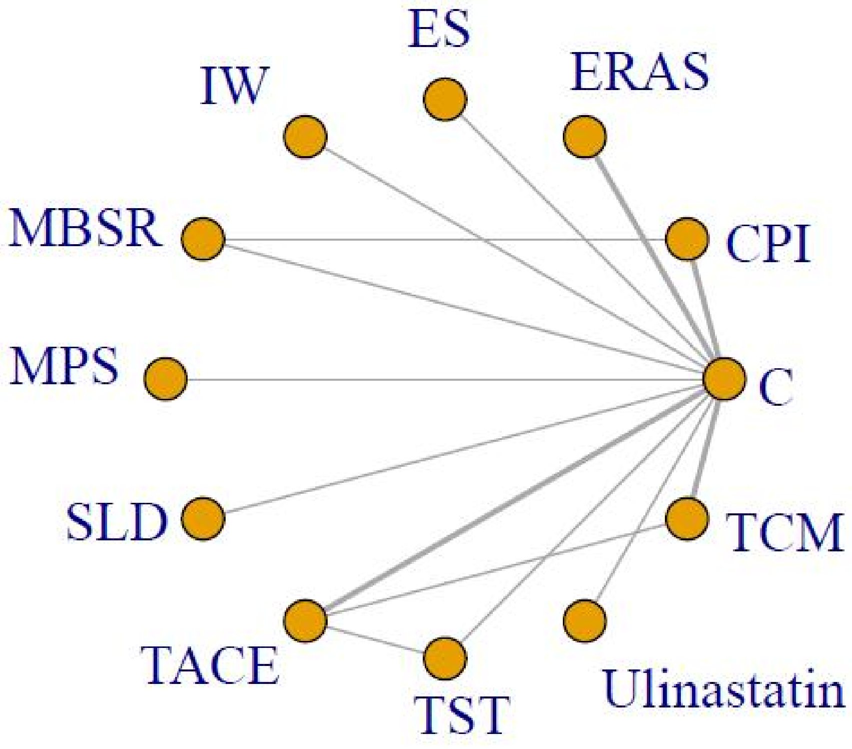

3.96 CD4/CD8(Others,3D)

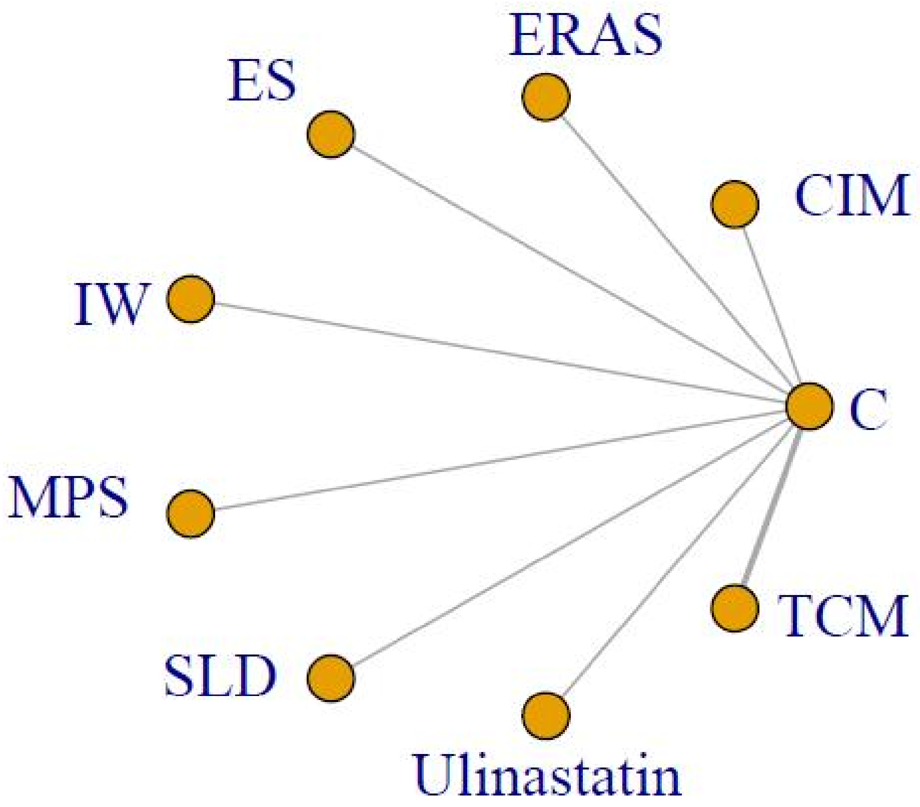

3.97 CD4/CD8(Others,>3D)

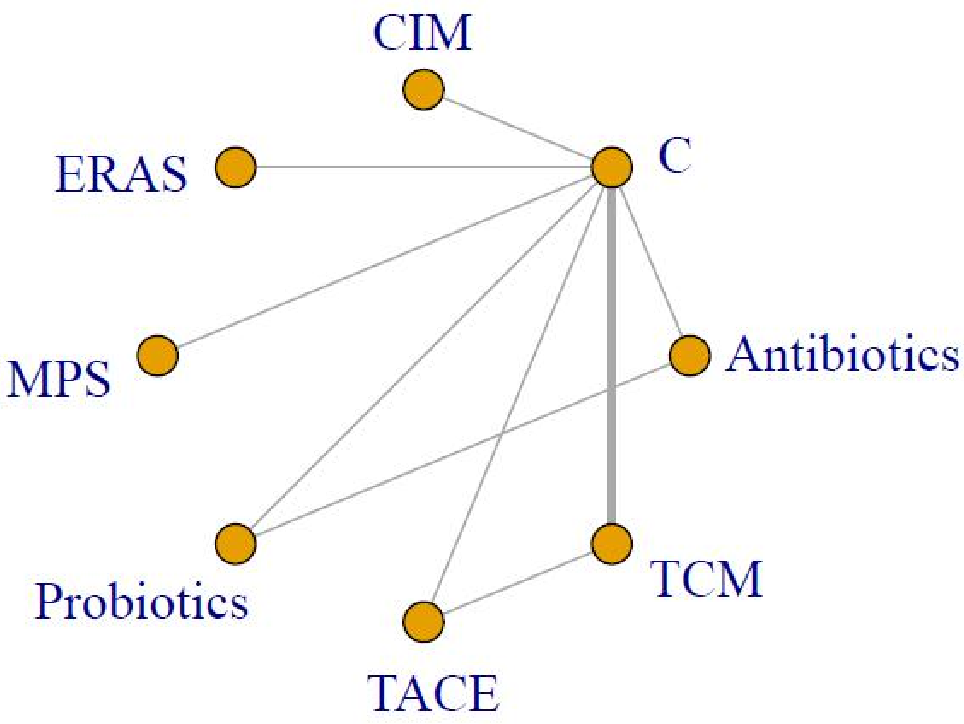

Supplement: Supplementary file 3 [file DataSheet3.pdf]
