# Supplementary material for: What are the beneficial treatment strategies in maintaining T lymphocyte subsets after cancer surgery? A systematic review and network meta-analysis
Source: Front Immunol. 2026 Jul 14;17:1854279. doi: 10.3389/fimmu.2026.1854279 (PMC13408238; doi:10.3389/fimmu.2026.1854279)
Supplement: Supplementary file 4 [file DataSheet4.pdf]

**Figure S4 Summary risk of bias**

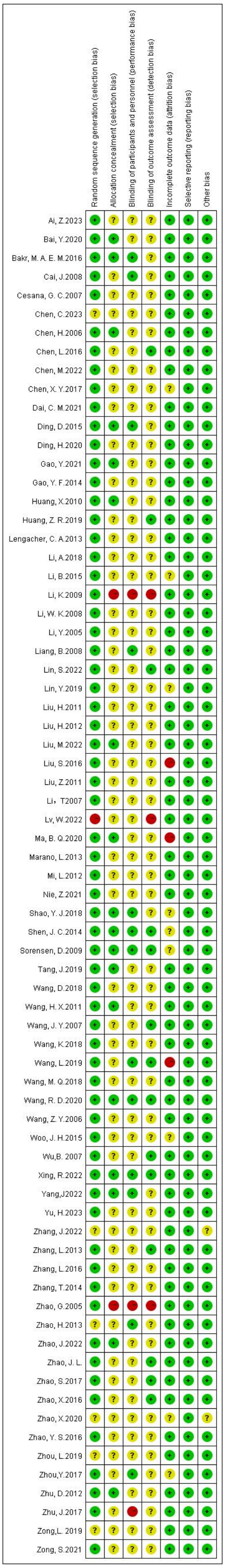
Random sequence generation (selection bias)
Allocation concealment (selection bias)
Blinding of participants and personnel (performance bias)
Blinding of outcome assessment (detection bias)
Incomplete outcome data (attrition bias)
Selective reporting (reporting bias)
Other bias
Ai, Z.2023
Bai, Y.2020
Bakr, M. A. E. M.2016
Cai, J.2008
Cesana, G. C.2007
Chen, C.2023
Chen, H.2006
Chen, L.2016
Chen, M.2022
Chen, X. Y.2017
Dai, C. M.2021
Ding, D.2015
Ding, H.2020
Gao, Y.2021
Gao, Y. F.2014
Huang, X.2010
Huang, Z. R.2019
Lengacher, C. A.2013
Li, A.2018
Li, B.2015
Li, K.2009
Li, W. K.2008
Li, Y.2005
Liang, B.2008
Lin, S.2022
Lin, Y.2019
Liu, H.2011
Liu, H.2012
Liu, M.2022
Liu, S.2016
Liu, Z.2011
Li，T2007
Lv, W.2022
Ma, B. Q.2020
Marano, L.2013
Mi, L.2012
Nie, Z.2021
Shao, Y. J.2018
Shen, J. C.2014
Sorensen, D.2009
Tang, J.2019
Wang, D.2018
Wang, H. X.2011
Wang, J. Y.2007
Wang, K.2018
Wang, L.2019
Wang, M. Q.2018
Wang, R. D.2020
Wang, Z. Y.2006
Woo, J. H.2015
Wu,B. 2007
Xing, R.2022
Yang,J2022
Yu, H.2023
Zhang, J.2022
Zhang, L.2013
Zhang, L.2016
Zhang, T.2014
Zhao, G.2005
Zhao, H.2013
Zhao, J.2022
Zhao, J. L.
Zhao, S.2017
Zhao, X.2016
Zhao, X.2020
Zhao, Y. S.2016
Zhou, L.2019
Zhou,Y.2017
Zhu, D.2012
Zhu, J.2017
Zong,L. 2019
Zong, S.2021

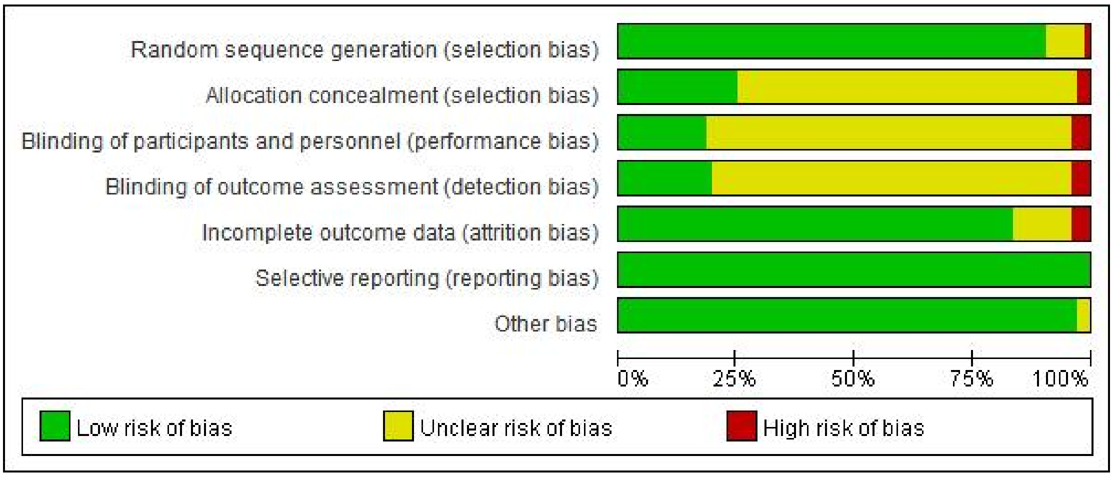

Random sequence generation (selection bias)
Allocation concealment (selection bias)
Blinding of participants and personnel (performance bias)
Blinding of outcome assessment (detection bias)
Incomplete outcome data (attrition bias)
Selective reporting (reporting bias)
Other bias
0%
25%
50%
75%
100%
Low risk of bias
Unclear risk of bias
High risk of bias
