# Supplementary material for: What are the beneficial treatment strategies in maintaining T lymphocyte subsets after cancer surgery? A systematic review and network meta-analysis
Source: Front Immunol. 2026 Jul 14;17:1854279. doi: 10.3389/fimmu.2026.1854279 (PMC13408238; doi:10.3389/fimmu.2026.1854279)

Figure S5 Forest plots of available comparisons among adverse reactions of the optimal intervention measures.

5.1 Total(Gastrointestinal disorders)

Study or Subgroup	Experimental Events	Experimental Total	Control Events	Control Total	Weight	Odds Ratio M-H, Fixed, 95% CI
Chen, C.2023	40	75	32	75	25.5%	1.54 [0.81, 2.93]
Li, X.2019	18	57	15	57	17.5%	1.29 [0.57, 2.91]
Liu, H.2011EN	8	28	6	28	7.3%	1.47 [0.43, 4.97]
Liu, H.2011PN	8	28	4	28	4.9%	2.40 [0.63, 9.16]
Liu, H.2012	2	28	3	24	5.1%	0.54 [0.08, 3.53]
Sorensen, D.2009	3	8	3	7	3.4%	0.80 [0.10, 6.35]
Wang, L.2019	1	20	8	20	13.0%	0.08 [0.01, 0.71]
Wang, M. Q.2018	1	20	1	20	1.6%	1.00 [0.06, 17.18]
Xin, L.2019	4	100	6	100	9.8%	0.65 [0.18, 2.39]
Yang,J2022	0	60	1	60	2.5%	0.33 [0.01, 8.21]
Zhao, H.2013	1	37	2	36	3.4%	0.47 [0.04, 5.45]
Zhu, J.2017	0	72	2	72	4.2%	0.19 [0.01, 4.12]
Zong, S.2021	2	30	1	30	1.6%	2.07 [0.18, 24.15]
**Total (95% CI)**		**563**		**557**	**100.0%**	**1.05 [0.74, 1.51]**
Total events	88		84			

Heterogeneity: Chi² = 12.08, df = 12 (P = 0.44); I² = 1%
Test for overall effect: Z = 0.29 (P = 0.77)

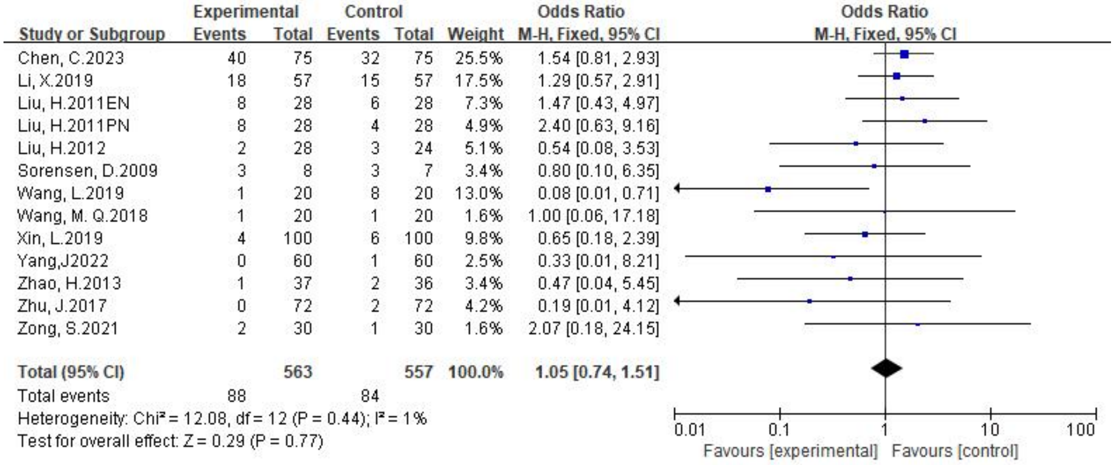

5.2 Diarrhea(Gastrointestinal disorders)

Study or Subgroup	Experimental Events	Experimental Total	Control Events	Control Total	Weight	Odds Ratio M-H, Fixed, 95% CI
Chen, C.2023	40	75	32	75	48.2%	1.54 [0.81, 2.93]
Li, X.2019	18	57	15	57	33.2%	1.29 [0.57, 2.91]
Xin, L.2019	4	100	6	100	18.6%	0.65 [0.18, 2.39]
**Total (95% CI)**		**232**		**232**	**100.0%**	**1.29 [0.81, 2.06]**
Total events	62		53			

Heterogeneity: Chi² = 1.34, df = 2 (P = 0.51); I² = 0%
Test for overall effect: Z = 1.07 (P = 0.28)

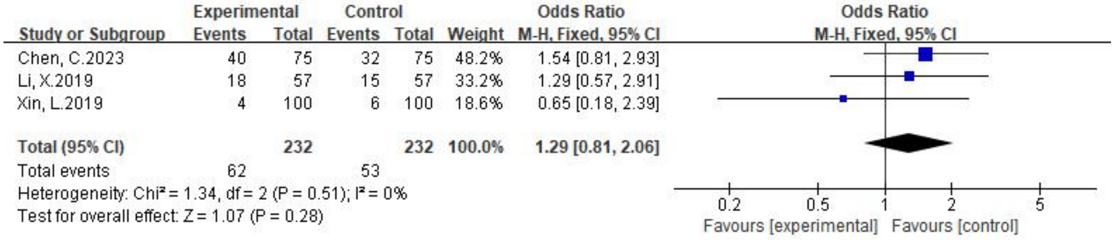

5.3 Gastrointestinal symptoms(Gastrointestinal disorders)

Study or Subgroup	Experimental Events	Experimental Total	Control Events	Control Total	Weight	Odds Ratio M-H, Fixed, 95% CI
Liu, H.2011EN	8	28	6	28	35.4%	1.47 [0.43, 4.97]
Liu, H.2011PN	8	28	4	28	23.6%	2.40 [0.63, 9.16]
Liu, H.2012	2	28	3	24	24.8%	0.54 [0.08, 3.53]
Zhao, H.2013	1	37	2	36	16.3%	0.47 [0.04, 5.45]
**Total (95% CI)**		**121**		**116**	**100.0%**	**1.30 [0.61, 2.73]**
Total events	19		15			

Heterogeneity: Chi² = 2.35, df = 3 (P = 0.50); I² = 0%
Test for overall effect: Z = 0.68 (P = 0.50)

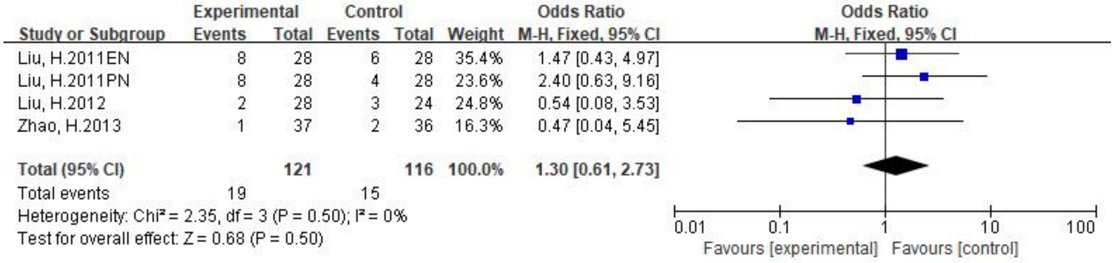

5.4 Nausea and vomiting(Gastrointestinal disorders)

Study or Subgroup	Experimental Events	Experimental Total	Control Events	Control Total	Weight	Odds Ratio M-H, Fixed, 95% CI
Wang, L.2019	1	20	8	20	63.5%	0.08 [0.01, 0.71]
Wang, M. Q.2018	1	20	1	20	7.9%	1.00 [0.06, 17.18]
Zhu, J.2017	0	72	2	72	20.7%	0.19 [0.01, 4.12]
Zong, S.2021	2	30	1	30	7.8%	2.07 [0.18, 24.15]
**Total (95% CI)**		**142**		**142**	**100.0%**	**0.33 [0.11, 0.99]**
Total events	4		12			

Heterogeneity: Chi² = 4.47, df = 3 (P = 0.22); I² = 33%
Test for overall effect: Z = 1.97 (P = 0.05)

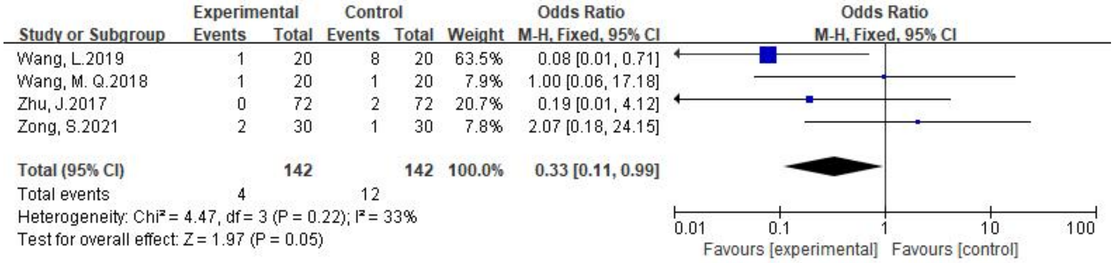

5.5 Dysphagia(Gastrointestinal disorders)

Study or Subgroup	Experimental Events	Experimental Total	Control Events	Control Total	Weight	Odds Ratio M-H, Fixed, 95% CI
Sorensen, D.2009	3	8	3	7	100.0%	0.80 [0.10, 6.35]
**Total (95% CI)**		**8**		**7**	**100.0%**	**0.80 [0.10, 6.35]**
Total events	3		3			
Heterogeneity: Not applicable						
Test for overall effect: Z = 0.21 (P = 0.83)						

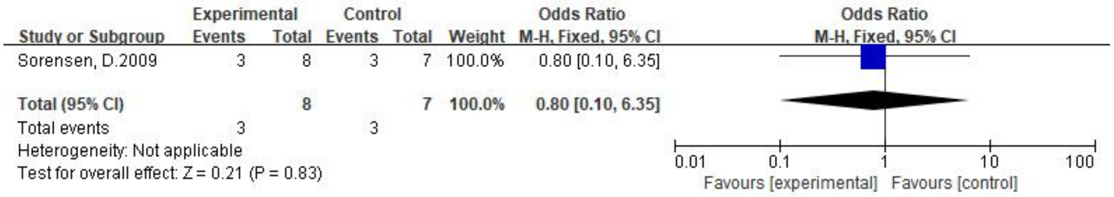

5.6 Chylous fistula(Gastrointestinal disorders)

Study or Subgroup	Experimental Events	Experimental Total	Control Events	Control Total	Weight	Odds Ratio M-H, Fixed, 95% CI
Yang,J2022	0	60	1	60	100.0%	0.33 [0.01, 8.21]
**Total (95% CI)**		**60**		**60**	**100.0%**	**0.33 [0.01, 8.21]**
Total events	0		1			
Heterogeneity: Not applicable						
Test for overall effect: Z = 0.68 (P = 0.50)						

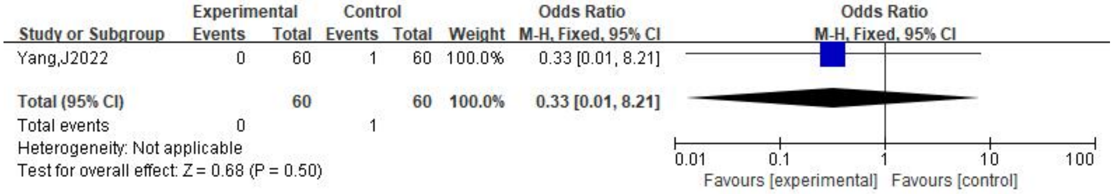

5.7 Total(Infection)

Study or Subgroup	Experimental Events	Experimental Total	Control Events	Control Total	Weight	Odds Ratio M-H, Fixed, 95% CI
Chen, C.2023	12	75	15	75	14.8%	0.76 [0.33, 1.76]
Liu, H.2011.EN.1	4	28	3	28	3.0%	1.39 [0.28, 6.87]
Liu, H.2011.EN.2	0	28	0	28		Not estimable
Liu, H.2011.EN.3	6	28	8	28	7.4%	0.68 [0.20, 2.31]
Liu, H.2011.PN.1	4	28	6	28	6.0%	0.61 [0.15, 2.46]
Liu, H.2011.PN.2	0	28	1	28	1.7%	0.32 [0.01, 8.24]
Liu, H.2011.PN.3	6	28	3	28	2.8%	2.27 [0.51, 10.18]
Liu, H.2012.1	3	28	2	24	2.3%	1.32 [0.20, 8.64]
Liu, H.2012.2	0	28	1	24	1.9%	0.27 [0.01, 7.07]
Liu, H.2012.3	5	28	6	24	6.2%	0.65 [0.17, 2.48]
Marano, L.2013.1	4	54	11	55	11.8%	0.32 [0.10, 1.08]
Marano, L.2013.2	1	54	3	55	3.4%	0.33 [0.03, 3.25]
Marano, L.2013.3	2	54	5	55	5.6%	0.38 [0.07, 2.07]
Marano, L.2013.4	1	54	2	55	2.3%	0.50 [0.04, 5.68]
Marano, L.2013.5	0	54	1	55	1.7%	0.33 [0.01, 8.36]
Marano, L.2013.6	2	54	4	55	4.5%	0.49 [0.09, 2.80]
Sorensen, D.2009.1	2	8	0	7	0.4%	5.77 [0.23, 143.37]
Sorensen, D.2009.2	0	8	2	7	2.9%	0.13 [0.01, 3.24]
Sorensen, D.2009.3	2	8	2	7	1.9%	0.83 [0.08, 8.24]
Sorensen, D.2009.4	2	8	2	7	1.9%	0.83 [0.08, 8.24]
Xin, L.2019.1	1	100	2	100	2.3%	0.49 [0.04, 5.55]
Xin, L.2019.2	0	100	0	100		Not estimable
Yang,J2022.1	3	60	5	60	5.6%	0.58 [0.13, 2.54]
Yang,J2022.2	1	60	2	60	2.3%	0.49 [0.04, 5.57]
Yang,J2022.3	0	60	1	60	1.7%	0.33 [0.01, 8.21]
Yang,J2022.4	0	60	0	60		Not estimable
Zhao, H.2013.1	2	37	2	36	2.2%	0.97 [0.13, 7.29]
Zhao, H.2013.2	2	37	3	36	3.4%	0.63 [0.10, 4.00]
Zhao, H.2013.3	0	37	0	36		Not estimable
**Total (95% CI)**		**1234**		**1221**	**100.0%**	**0.66 [0.47, 0.92]**
Total events	65		92			
Heterogeneity: $Chi^2 = 10.30$, df = 24 (P = 0.99); $I^2 = 0\%$						
Test for overall effect: Z = 2.43 (P = 0.01)						

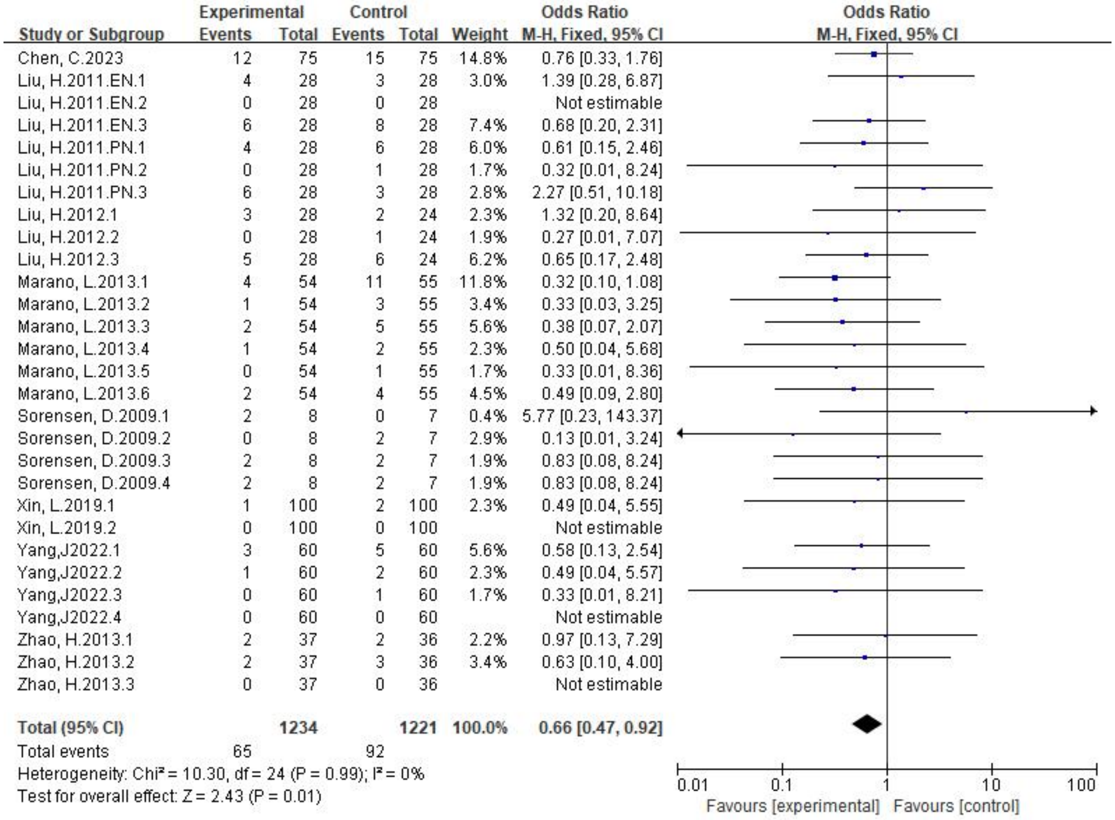

5.8 Infectious complications(Infection)

Study or Subgroup	Experimental Events	Experimental Total	Control Events	Control Total	Weight	Odds Ratio M-H, Fixed, 95% CI
Marano, L.2013.1	4	54	11	55	100.0%	0.32 [0.10, 1.08]
**Total (95% CI)**		**54**		**55**	**100.0%**	**0.32 [0.10, 1.08]**
Total events	4		11			
Heterogeneity: Not applicable						
Test for overall effect: Z = 1.84 (P = 0.07)						

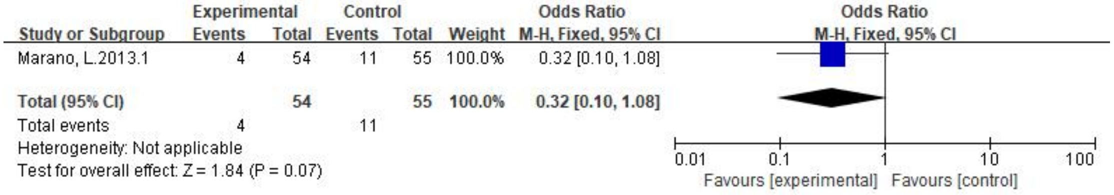

5.9 Incision infection(Infection)

Study or Subgroup	Experimental Events	Experimental Total	Control Events	Control Total	Weight	Odds Ratio M-H, Fixed, 95% CI
Liu, H.2011.EN.1	4	28	3	28	14.0%	1.39 [0.28, 6.87]
Liu, H.2011.PN.1	4	28	6	28	28.1%	0.61 [0.15, 2.46]
Liu, H.2012.1	3	28	2	24	10.5%	1.32 [0.20, 8.64]
Marano, L.2013.2	1	54	3	55	15.9%	0.33 [0.03, 3.25]
Sorensen, D.2009.1	2	8	0	7	2.1%	5.77 [0.23, 143.37]
Xin, L.2019.1	1	100	2	100	10.8%	0.49 [0.04, 5.55]
Yang,J2022.3	0	60	1	60	8.1%	0.33 [0.01, 8.21]
Zhao, H.2013.1	2	37	2	36	10.5%	0.97 [0.13, 7.29]
**Total (95% CI)**		**343**		**338**	**100.0%**	**0.86 [0.44, 1.68]**
Total events	17		19			

Heterogeneity: Chi² = 3.37, df = 7 (P = 0.85); I² = 0%
Test for overall effect: Z = 0.44 (P = 0.66)

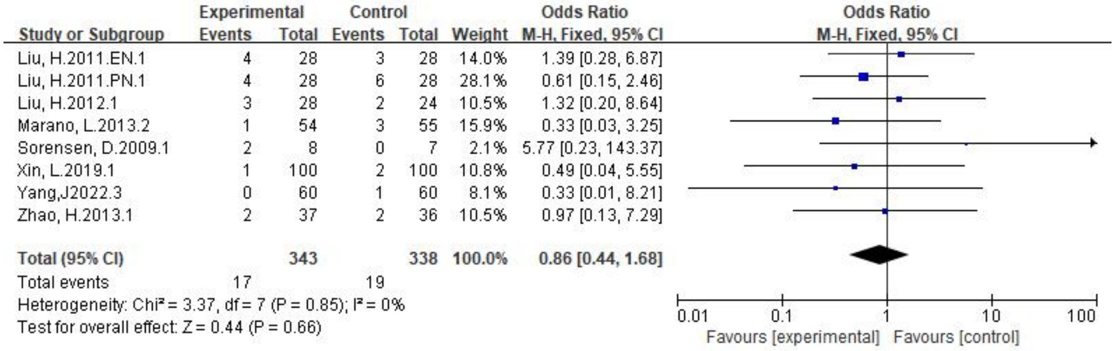

5.10 Respiratory tract infection(Infection)

Study or Subgroup	Experimental Events	Experimental Total	Control Events	Control Total	Weight	Odds Ratio M-H, Fixed, 95% CI
Liu, H.2011.EN.3	6	28	8	28	22.5%	0.68 [0.20, 2.31]
Liu, H.2011.PN.3	6	28	3	28	8.4%	2.27 [0.51, 10.18]
Liu, H.2012.3	5	28	6	24	19.0%	0.65 [0.17, 2.48]
Marano, L.2013.3	2	54	5	55	17.1%	0.38 [0.07, 2.07]
Sorensen, D.2009.3	2	8	2	7	5.7%	0.83 [0.08, 8.24]
Xin, L.2019.2	0	100	0	100		Not estimable
Yang,J2022.1	3	60	5	60	17.0%	0.58 [0.13, 2.54]
Zhao, H.2013.2	2	37	3	36	10.3%	0.63 [0.10, 4.00]
**Total (95% CI)**		**343**		**338**	**100.0%**	**0.75 [0.42, 1.31]**
Total events	26		32			

Heterogeneity: Chi² = 2.93, df = 6 (P = 0.82); I² = 0%
Test for overall effect: Z = 1.02 (P = 0.31)

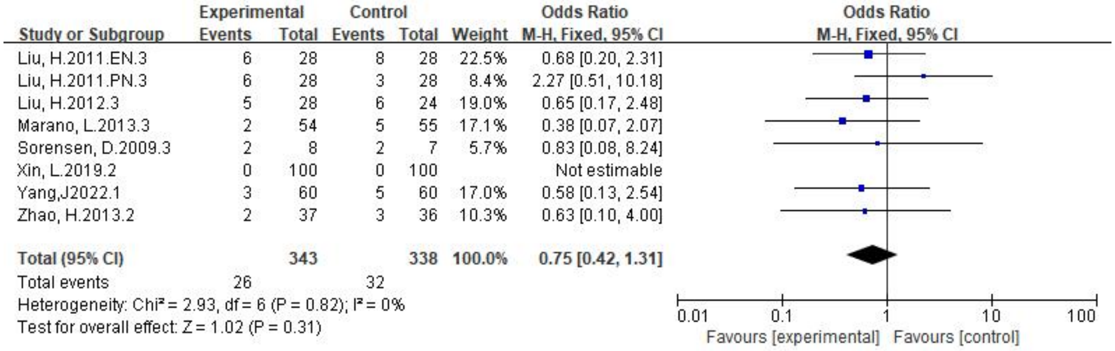

5.11 Urinary tract infection(Infection)

Study or Subgroup	Experimental Events	Experimental Total	Control Events	Control Total	Weight	Odds Ratio M-H, Fixed, 95% CI
Marano, L.2013.4	1	54	2	55	54.9%	0.50 [0.04, 5.68]
Sorensen, D.2009.4	2	8	2	7	45.1%	0.83 [0.08, 8.24]
**Total (95% CI)**		**62**		**62**	**100.0%**	**0.65 [0.12, 3.39]**
Total events	3		4			

Heterogeneity: Chi² = 0.09, df = 1 (P = 0.76); I² = 0%
Test for overall effect: Z = 0.51 (P = 0.61)

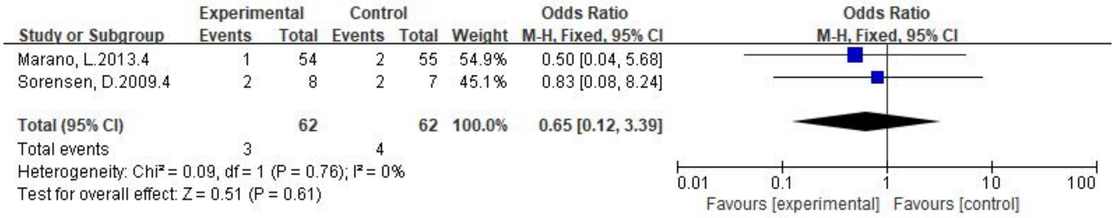

5.12 Abdominal cavity infection(Infection)

Study or Subgroup	Experimental Events	Experimental Total	Control Events	Control Total	Weight	Odds Ratio M-H, Fixed, 95% CI
Yang,J2022.2	1	60	2	60	100.0%	0.49 [0.04, 5.57]
**Total (95% CI)**		**60**		**60**	**100.0%**	**0.49 [0.04, 5.57]**
Total events	1		2			

Heterogeneity: Not applicable
Test for overall effect: Z = 0.57 (P = 0.57)

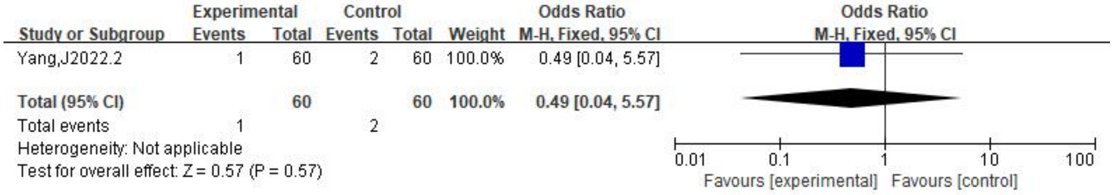

5.13 Sepsis(Infection)

Study or Subgroup	Experimental Events	Experimental Total	Control Events	Control Total	Weight	Odds Ratio M-H, Fixed, 95% CI
Marano, L.2013.5	0	54	1	55	100.0%	0.33 [0.01, 8.36]
**Total (95% CI)**		**54**		**55**	**100.0%**	**0.33 [0.01, 8.36]**
Total events	0		1			

Heterogeneity: Not applicable
Test for overall effect: Z = 0.67 (P = 0.50)

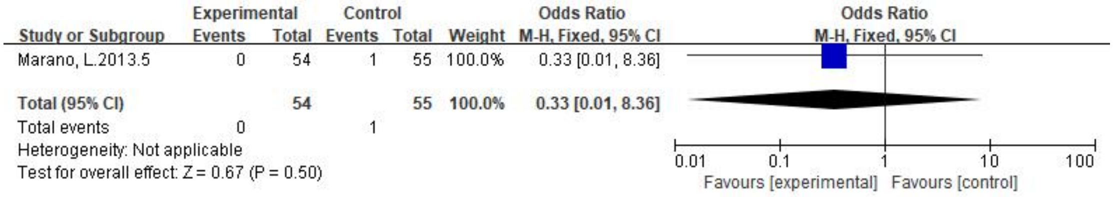

5.14 Abscess(Infection)

Study or Subgroup	Experimental Events	Experimental Total	Control Events	Control Total	Weight	Odds Ratio M-H, Fixed, 95% CI
Marano, L.2013.6	2	54	4	55	100.0%	0.49 [0.09, 2.80]
**Total (95% CI)**		**54**		**55**	**100.0%**	**0.49 [0.09, 2.80]**
Total events	2		4			
Heterogeneity: Not applicable						
Test for overall effect: Z = 0.80 (P = 0.42)						

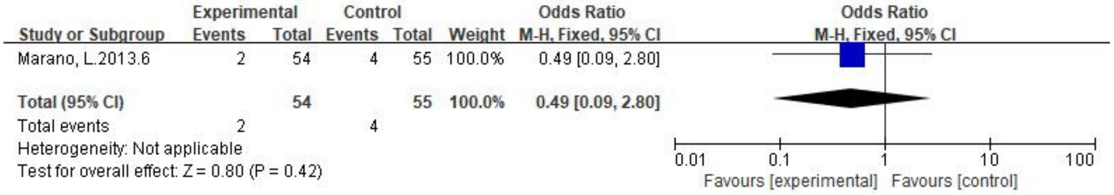

5.15 Anastomosis leakage(Infection)

Study or Subgroup	Experimental Events	Experimental Total	Control Events	Control Total	Weight	Odds Ratio M-H, Fixed, 95% CI
Liu, H.2011.EN.2	0	28	0	28		Not estimable
Liu, H.2011.PN.2	0	28	1	28	26.5%	0.32 [0.01, 8.24]
Liu, H.2012.2	0	28	1	24	28.5%	0.27 [0.01, 7.07]
Sorensen, D.2009.2	0	8	2	7	45.0%	0.13 [0.01, 3.24]
Yang,J2022.4	0	60	0	60		Not estimable
Zhao, H.2013.3	0	37	0	36		Not estimable
**Total (95% CI)**		**189**		**183**	**100.0%**	**0.22 [0.03, 1.42]**
Total events	0		4			
Heterogeneity: Chi² = 0.17, df = 2 (P = 0.92); I² = 0%						
Test for overall effect: Z = 1.59 (P = 0.11)						

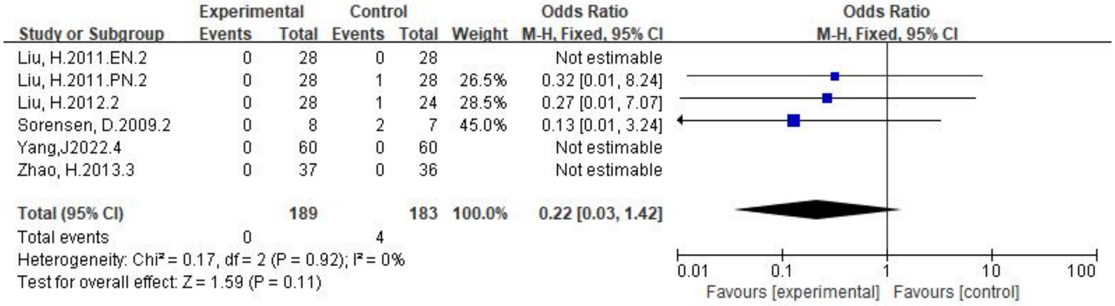

5.16 Fever(Infection)

Study or Subgroup	Experimental Events	Experimental Total	Control Events	Control Total	Weight	Odds Ratio M-H, Fixed, 95% CI
Chen, C.2023	12	75	15	75	100.0%	0.76 [0.33, 1.76]
**Total (95% CI)**		**75**		**75**	**100.0%**	**0.76 [0.33, 1.76]**
Total events	12		15			
Heterogeneity: Not applicable						
Test for overall effect: Z = 0.64 (P = 0.52)						

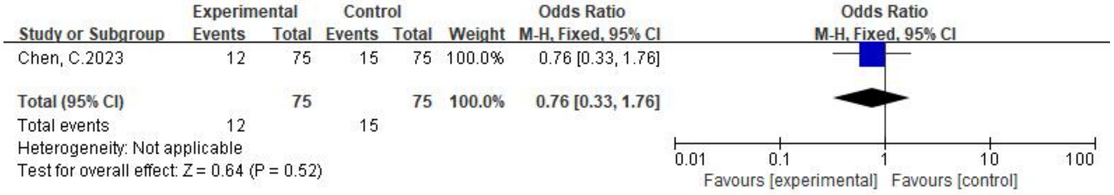

5.17 Total(Skin disorders)

Study or Subgroup	Experimental Events	Experimental Total	Control Events	Control Total	Weight	Odds Ratio M-H, Fixed, 95% CI
Chen, C.2023	52	75	47	75	51.7%	1.35 [0.68, 2.65]
Li, X.2019	22	57	8	57	17.6%	3.85 [1.54, 9.64]
Li, X.2019.1	12	57	9	57	25.5%	1.42 [0.55, 3.70]
Wang, M. Q.2018	0	20	1	20	5.2%	0.32 [0.01, 8.26]
**Total (95% CI)**		**209**		**209**	**100.0%**	**1.75 [1.11, 2.77]**
Total events	86		65			
Heterogeneity: Chi² = 4.64, df = 3 (P = 0.20); I² = 35%						
Test for overall effect: Z = 2.40 (P = 0.02)						

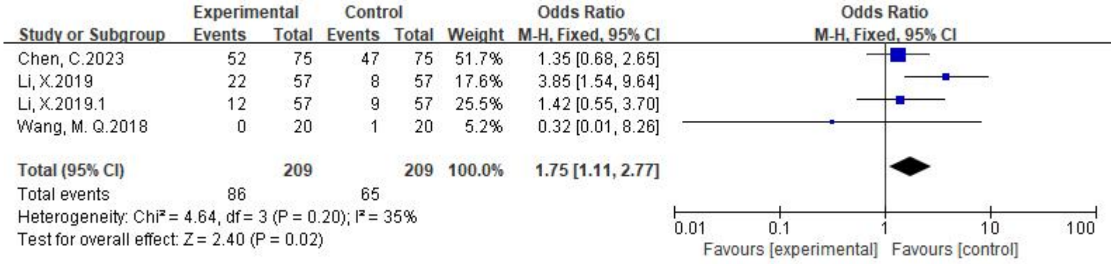

5.18 Hand-foot syndrome(Skin disorders)

Study or Subgroup	Experimental Events	Experimental Total	Control Events	Control Total	Weight	Odds Ratio M-H, Random, 95% CI
Chen, C.2023	52	75	47	75	54.5%	1.35 [0.68, 2.65]
Li, X.2019	22	57	8	57	45.5%	3.85 [1.54, 9.64]
**Total (95% CI)**		**132**		**132**	**100.0%**	**2.17 [0.78, 6.06]**
Total events	74		55			
Heterogeneity: Tau² = 0.38; Chi² = 3.26, df = 1 (P = 0.07); I² = 69%						
Test for overall effect: Z = 1.48 (P = 0.14)						

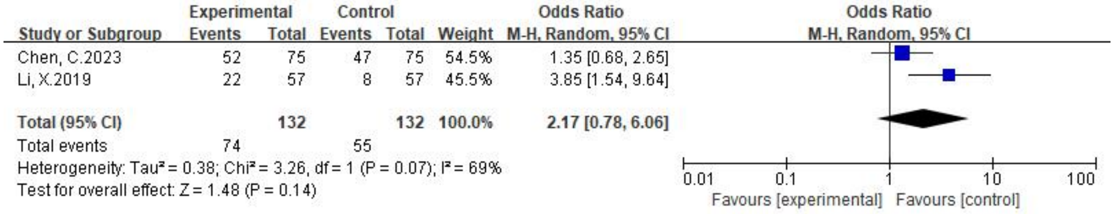

5.19 Itchy skin(Skin disorders)

Study or Subgroup	Experimental Events	Experimental Total	Control Events	Control Total	Weight	Odds Ratio M-H, Fixed, 95% CI
Wang, M. Q.2018	0	20	1	20	100.0%	0.32 [0.01, 8.26]
**Total (95% CI)**		**20**		**20**	**100.0%**	**0.32 [0.01, 8.26]**
Total events	0		1			
Heterogeneity: Not applicable						
Test for overall effect: Z = 0.69 (P = 0.49)						

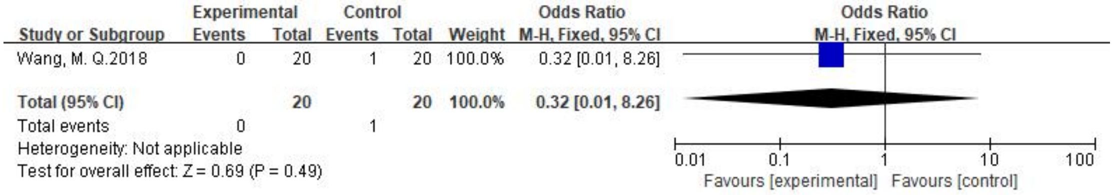

5.20 Hair loss(Skin disorders)

Study or Subgroup	Experimental Events	Experimental Total	Control Events	Control Total	Weight	Odds Ratio M-H, Fixed, 95% CI
Li, X.2019.1	12	57	9	57	100.0%	1.42 [0.55, 3.70]
**Total (95% CI)**		**57**		**57**	**100.0%**	**1.42 [0.55, 3.70]**
Total events	12		9			
Heterogeneity: Not applicable						
Test for overall effect: Z = 0.72 (P = 0.47)						

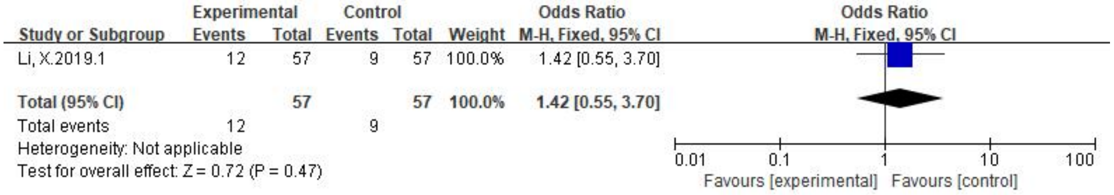

5.21 Total(Abnormal blood pressure)

Study or Subgroup	Experimental Events	Experimental Total	Control Events	Control Total	Weight	Odds Ratio M-H, Fixed, 95% CI
Li, X.2019	16	57	12	57	85.8%	1.46 [0.62, 3.46]
Wang, M. Q.2018	1	20	0	20	4.6%	3.15 [0.12, 82.16]
Zong, S.2021	1	30	1	30	9.6%	1.00 [0.06, 16.76]
**Total (95% CI)**		**107**		**107**	**100.0%**	**1.50 [0.68, 3.31]**
Total events	18		13			
Heterogeneity: Chi² = 0.28, df = 2 (P = 0.87); I² = 0%						
Test for overall effect: Z = 1.00 (P = 0.32)						

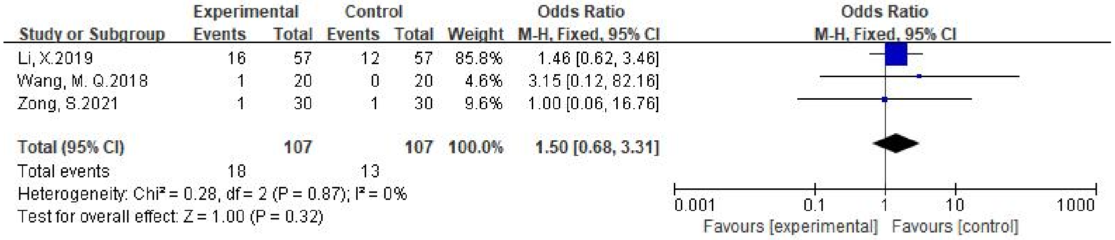

5.22 Hypertension(Abnormal blood pressure)

Study or Subgroup	Experimental Events	Experimental Total	Control Events	Control Total	Weight	Odds Ratio M-H, Fixed, 95% CI
Li, X.2019	16	57	12	57	100.0%	1.46 [0.62, 3.46]
**Total (95% CI)**		**57**		**57**	**100.0%**	**1.46 [0.62, 3.46]**
Total events	16		12			
Heterogeneity: Not applicable						
Test for overall effect: Z = 0.87 (P = 0.39)						

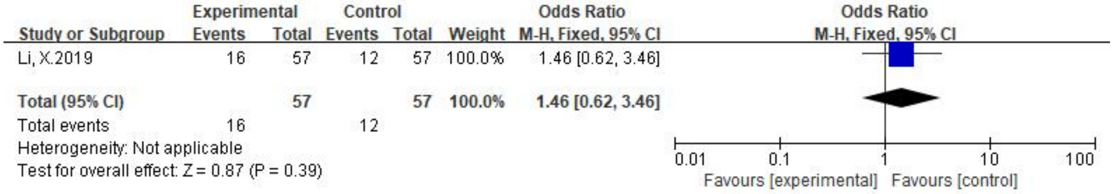

5.23 Hypotension(Abnormal blood pressure)

Study or Subgroup	Experimental Events	Experimental Total	Control Events	Control Total	Weight	Odds Ratio M-H, Fixed, 95% CI
Wang, M. Q.2018	1	20	0	20	32.4%	3.15 [0.12, 82.16]
Zong, S.2021	1	30	1	30	67.6%	1.00 [0.06, 16.76]
**Total (95% CI)**		**50**		**50**	**100.0%**	**1.70 [0.22, 13.32]**
Total events	2		1			
Heterogeneity: Chi² = 0.27, df = 1 (P = 0.60); I² = 0%						
Test for overall effect: Z = 0.50 (P = 0.61)						

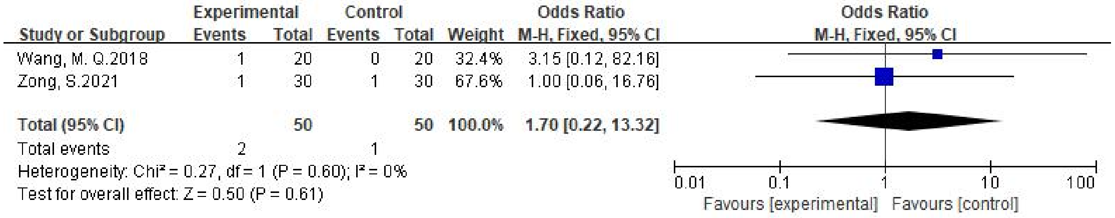

5.24 Total(Nervous system disorders)

Study or Subgroup	Experimental Events	Experimental Total	Control Events	Control Total	Weight	Odds Ratio M-H, Fixed, 95% CI
Chen, C.2023	29	75	32	75	71.6%	0.85 [0.44, 1.63]
Sorensen, D.2009	1	8	1	7	3.4%	0.86 [0.04, 16.85]
Wang, M. Q.2018	0	20	2	20	8.9%	0.18 [0.01, 4.01]
Zhu, J.2017	0	72	2	72	9.1%	0.19 [0.01, 4.12]
Zong, S.2021	1	30	2	30	7.1%	0.48 [0.04, 5.63]
**Total (95% CI)**		**205**		**204**	**100.0%**	**0.70 [0.39, 1.26]**
Total events	31		39			
Heterogeneity: Chi² = 1.84, df = 4 (P = 0.77); I² = 0%						
Test for overall effect: Z = 1.19 (P = 0.23)						

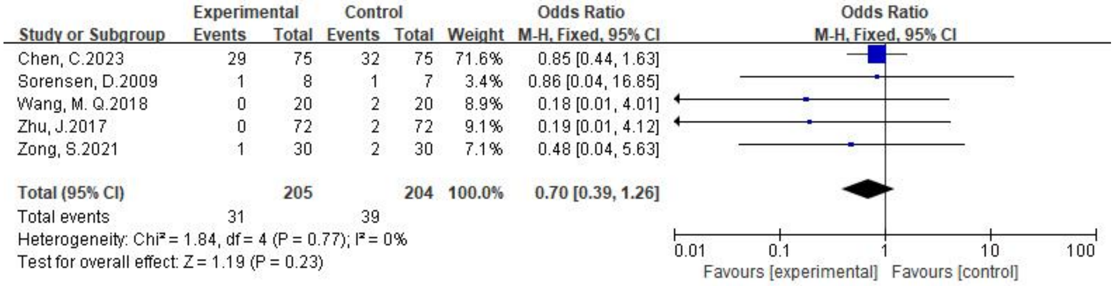

5.25 Pain(Nervous system disorders)

Study or Subgroup	Experimental Events	Experimental Total	Control Events	Control Total	Weight	Odds Ratio M-H, Fixed, 95% CI
Chen, C.2023	29	75	32	75	100.0%	0.85 [0.44, 1.63]
**Total (95% CI)**		**75**		**75**	**100.0%**	**0.85 [0.44, 1.63]**
Total events	29		32			
Heterogeneity: Not applicable						
Test for overall effect: Z = 0.50 (P = 0.62)						

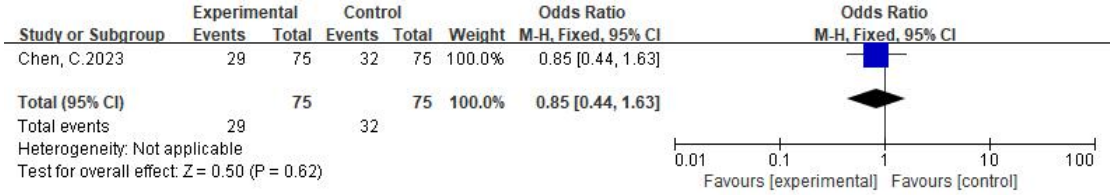

5.26 Neurological dysfunction(Nervous system disorders)

Study or Subgroup	Experimental Events	Experimental Total	Control Events	Control Total	Weight	Odds Ratio M-H, Fixed, 95% CI
Sorensen, D.2009	1	8	1	7	100.0%	0.86 [0.04, 16.85]
**Total (95% CI)**		**8**		**7**	**100.0%**	**0.86 [0.04, 16.85]**
Total events	1		1			
Heterogeneity: Not applicable						
Test for overall effect: Z = 0.10 (P = 0.92)						

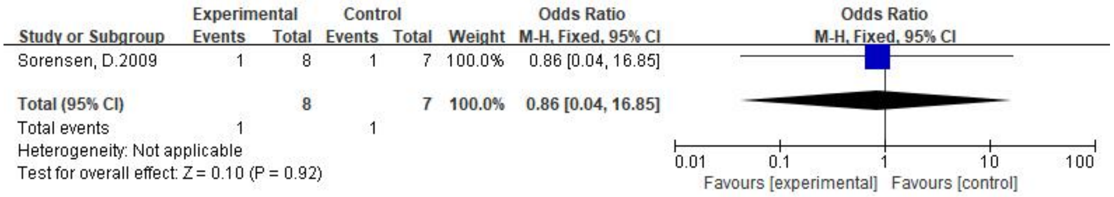

5.27 Dizziness(Nervous system disorders)

Study or Subgroup	Experimental Events	Experimental Total	Control Events	Control Total	Weight	Odds Ratio M-H, Fixed, 95% CI
Zhu, J.2017	0	72	2	72	56.2%	0.19 [0.01, 4.12]
Zong, S.2021	1	30	2	30	43.8%	0.48 [0.04, 5.63]
**Total (95% CI)**		**102**		**102**	**100.0%**	**0.32 [0.05, 2.10]**
Total events	1		4			
Heterogeneity: Chi² = 0.21, df = 1 (P = 0.65); I² = 0%						
Test for overall effect: Z = 1.19 (P = 0.24)						

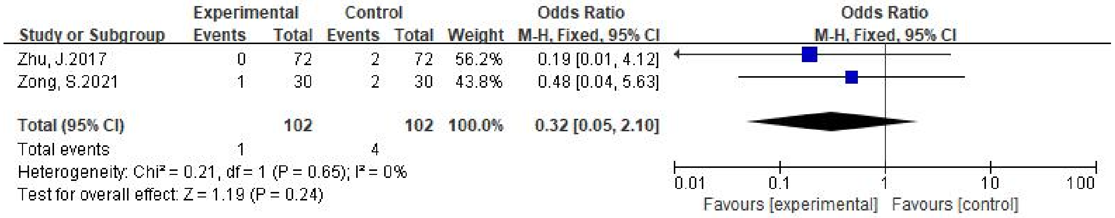

5.28 Respiratory depression(Nervous system disorders)

Study or Subgroup	Experimental Events	Experimental Total	Control Events	Control Total	Weight	Odds Ratio M-H, Fixed, 95% CI
Wang, M. Q.2018	0	20	2	20	100.0%	0.18 [0.01, 4.01]
**Total (95% CI)**		**20**		**20**	**100.0%**	**0.18 [0.01, 4.01]**
Total events	0		2			
Heterogeneity: Not applicable						
Test for overall effect: Z = 1.08 (P = 0.28)						

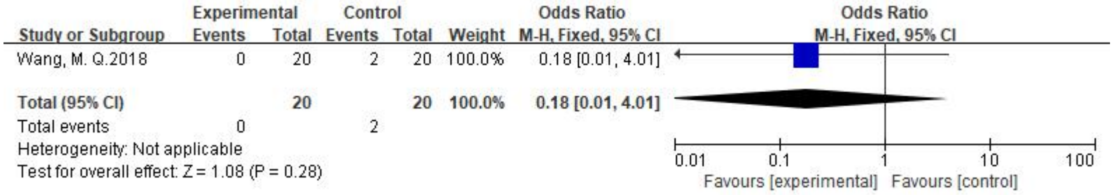

5.29 Total(Other disorders)

Study or Subgroup	Experimental Events	Experimental Total	Control Events	Control Total	Weight	Odds Ratio M-H, Fixed, 95% CI
Chen, C.2023.1	50	75	51	75	21.5%	0.94 [0.48, 1.86]
Chen, C.2023.2	14	75	38	75	39.1%	0.22 [0.11, 0.47]
Li, X.2019	19	57	14	57	11.8%	1.54 [0.68, 3.48]
Sorensen, D.2009	1	8	0	7	0.6%	3.00 [0.10, 86.09]
Xin, L.2019	8	100	12	100	14.0%	0.64 [0.25, 1.63]
Yang,J2022.1	0	60	2	60	3.1%	0.19 [0.01, 4.11]
Yang,J2022.2	0	60	1	60	1.9%	0.33 [0.01, 8.21]
Zhu, J.2017.1	0	72	2	72	3.1%	0.19 [0.01, 4.12]
Zhu, J.2017.2	0	72	3	72	4.4%	0.14 [0.01, 2.70]
Zong, S.2021	1	30	0	30	0.6%	3.10 [0.12, 79.23]
**Total (95% CI)**		**609**		**608**	**100.0%**	**0.62 [0.44, 0.88]**
Total events	93		123			
Heterogeneity: Chi² = 17.60, df = 9 (P = 0.04); I² = 49%						
Test for overall effect: Z = 2.66 (P = 0.008)						

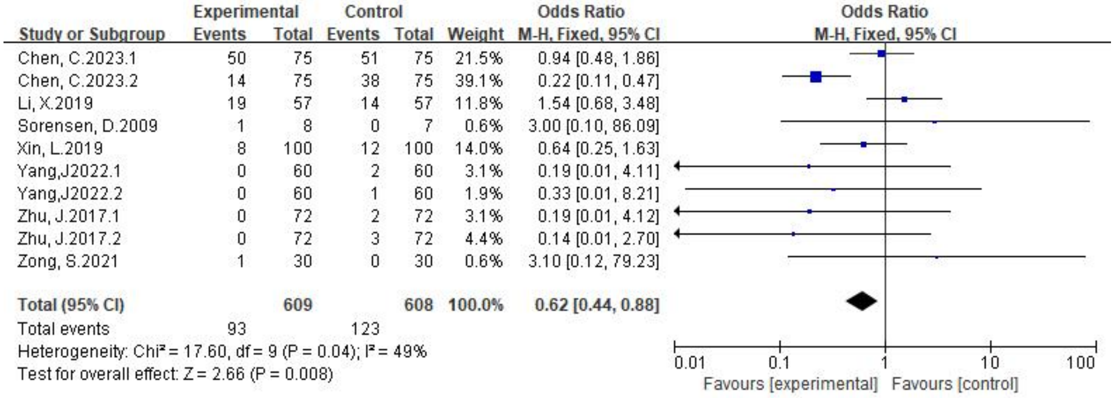

Supplement: Supplementary file 5 [file DataSheet5.pdf]
