# Supplementary material for: What are the beneficial treatment strategies in maintaining T lymphocyte subsets after cancer surgery? A systematic review and network meta-analysis
Source: Front Immunol. 2026 Jul 14;17:1854279. doi: 10.3389/fimmu.2026.1854279 (PMC13408238; doi:10.3389/fimmu.2026.1854279)
Supplement: Supplementary file 6 [file DataSheet6.pdf]

Figure S6 Funnel plots of available comparisons among adverse reactions of the optimal intervention measures.

6.1 Total(Gastrointestinal disorders)

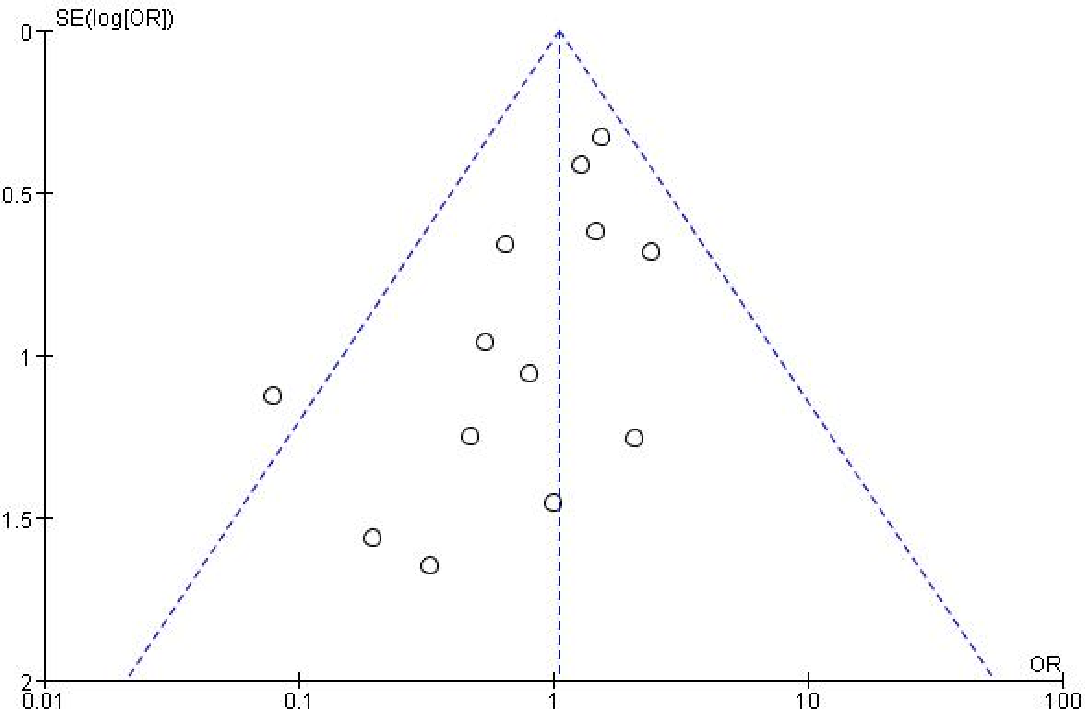

6.2 Diarrhea(Gastrointestinal disorders)

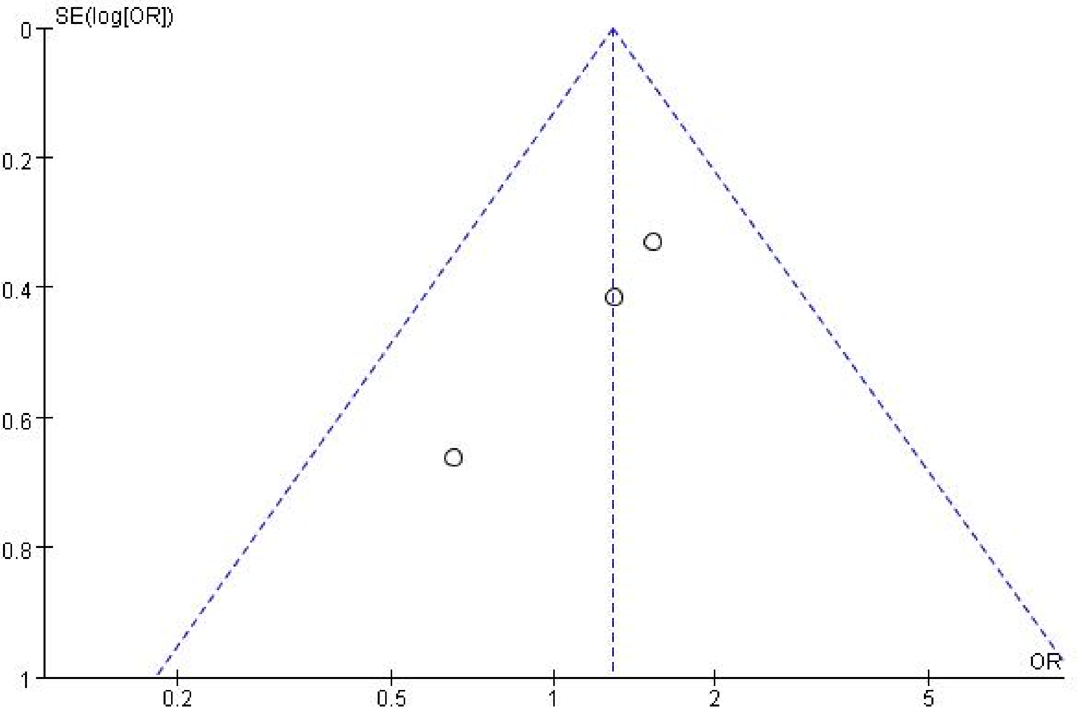

6.3 Gastrointestinal symptoms(Gastrointestinal disorders)

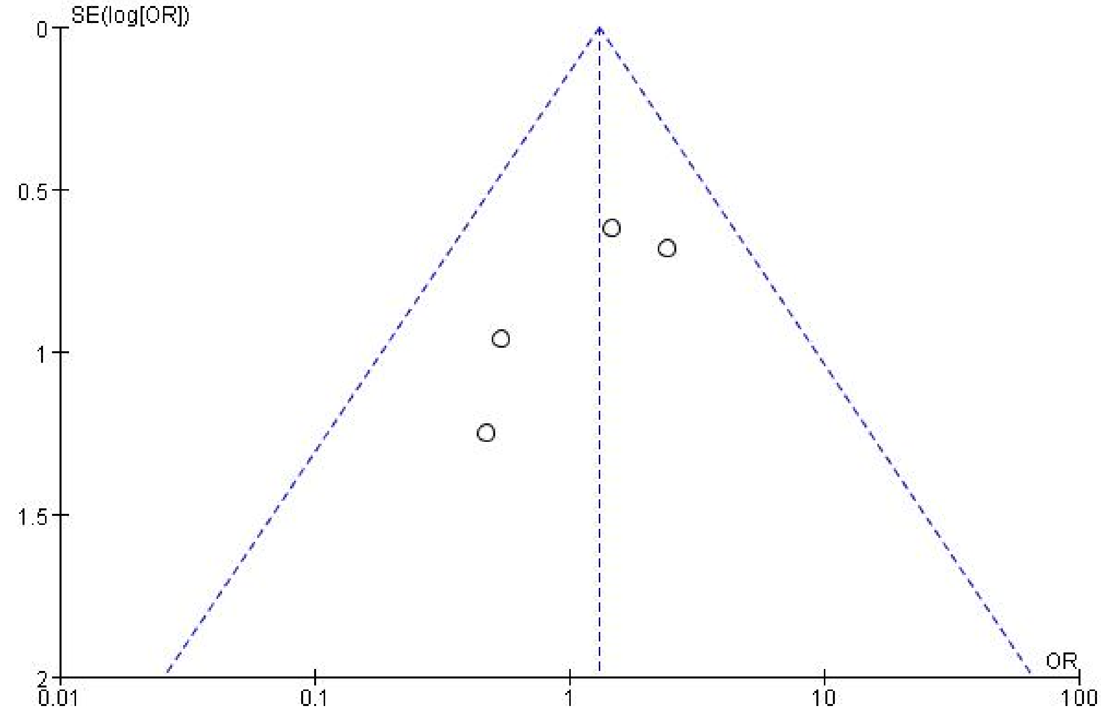

6.4 Nausea and vomiting(Gastrointestinal disorders)

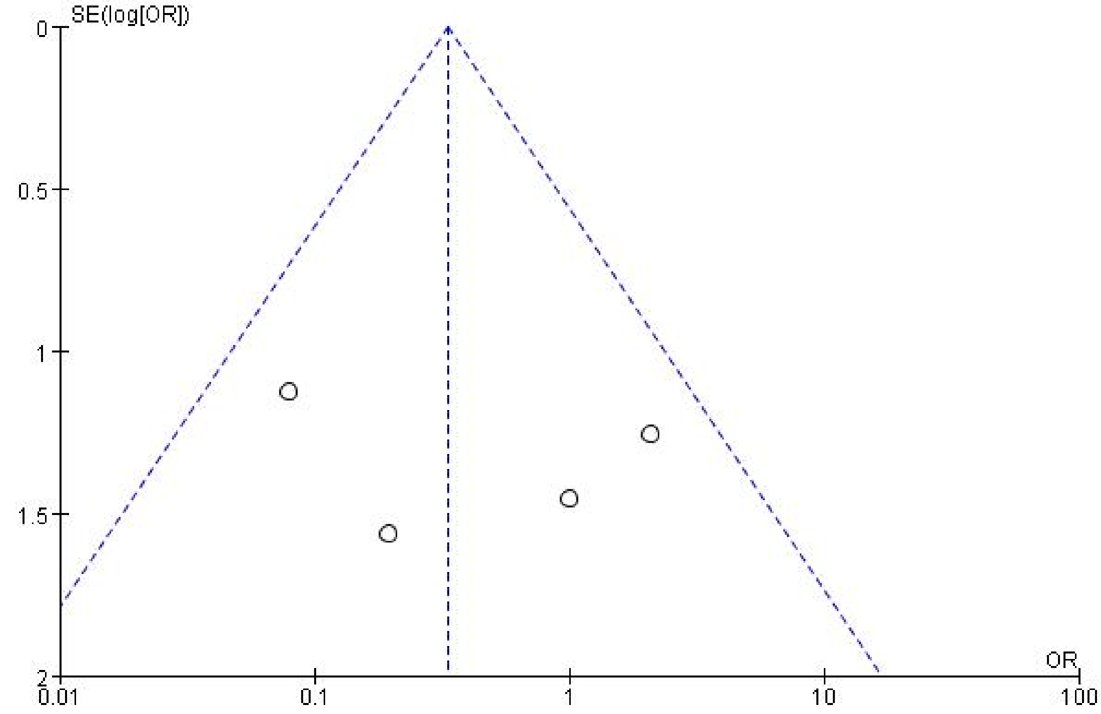

6.5 Dysphagia(Gastrointestinal disorders)

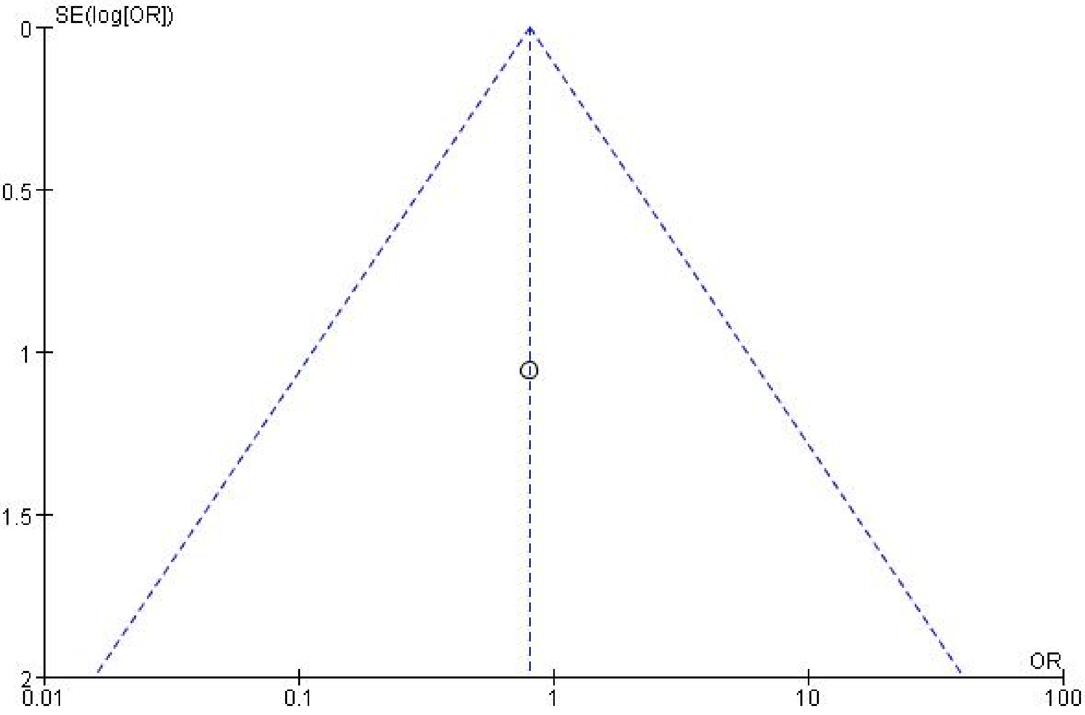

6.6 Chylous fistula(Gastrointestinal disorders)

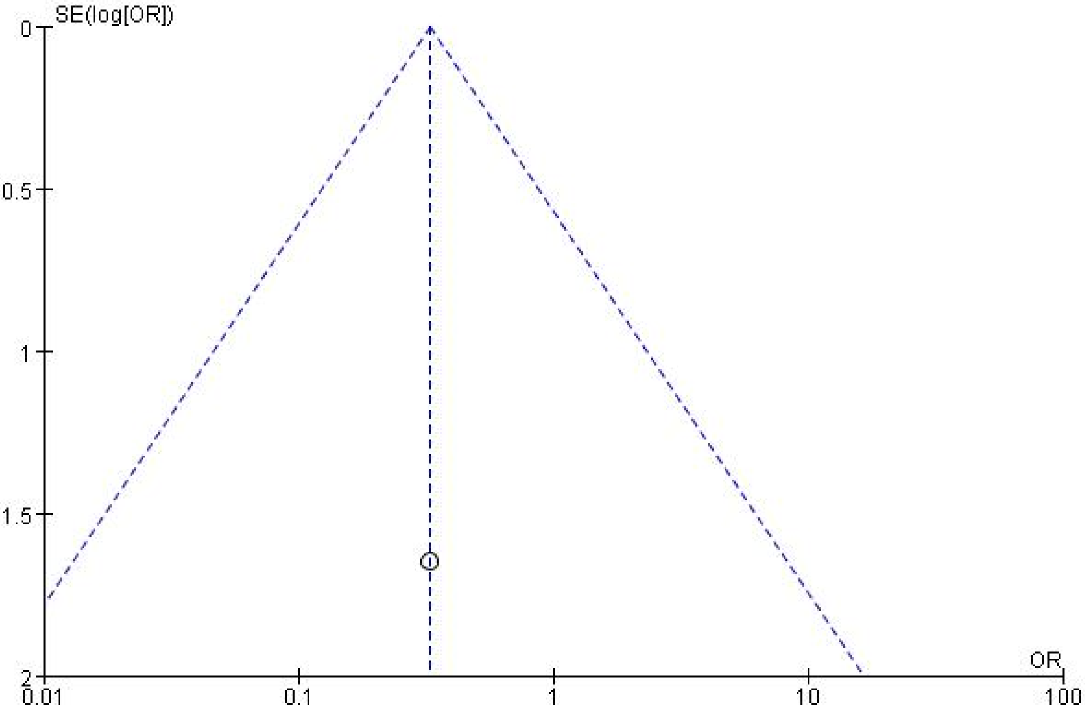

6.7 Total(Infection)

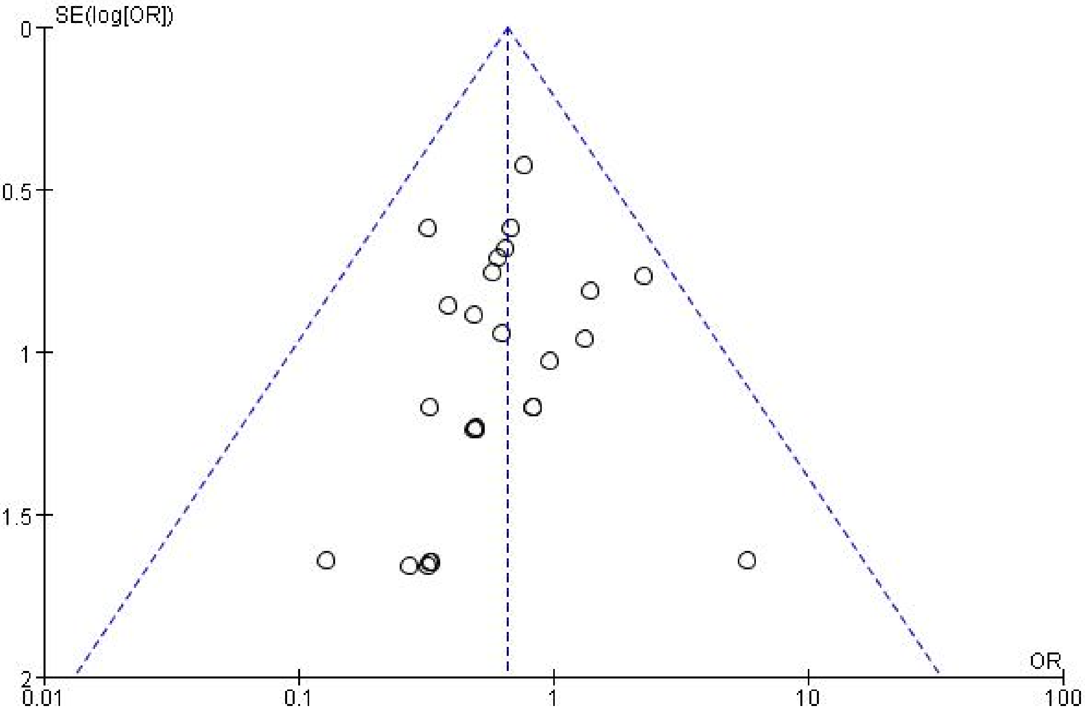

6.8 Infectious complications(Infection)

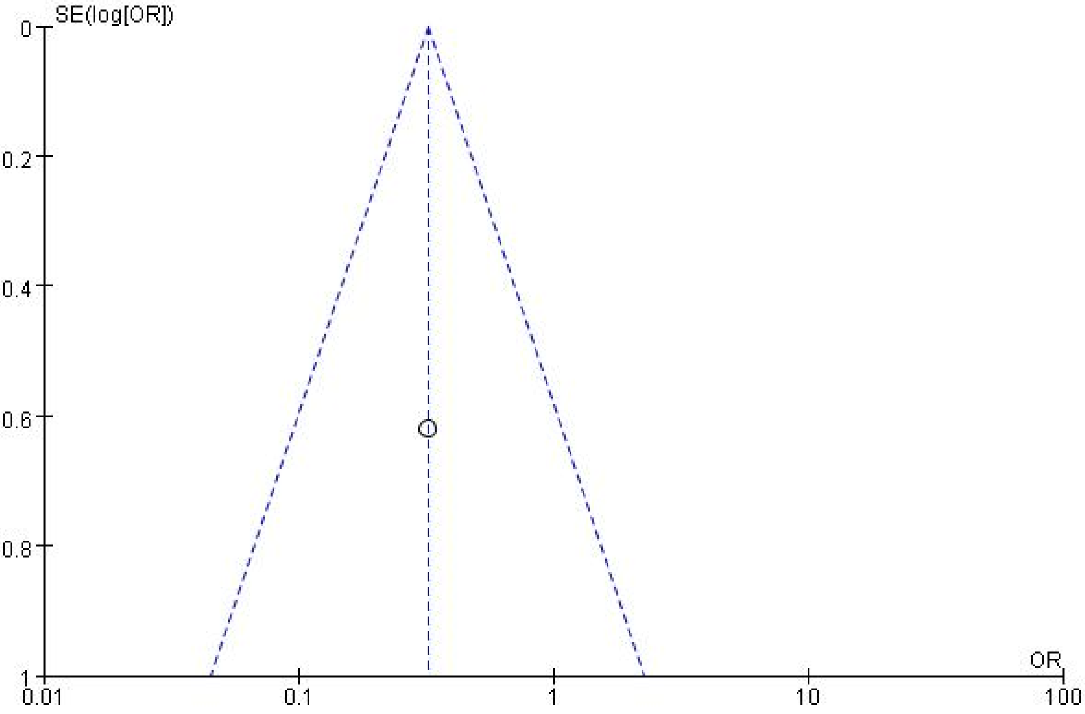

6.9 Incision infection(Infection)

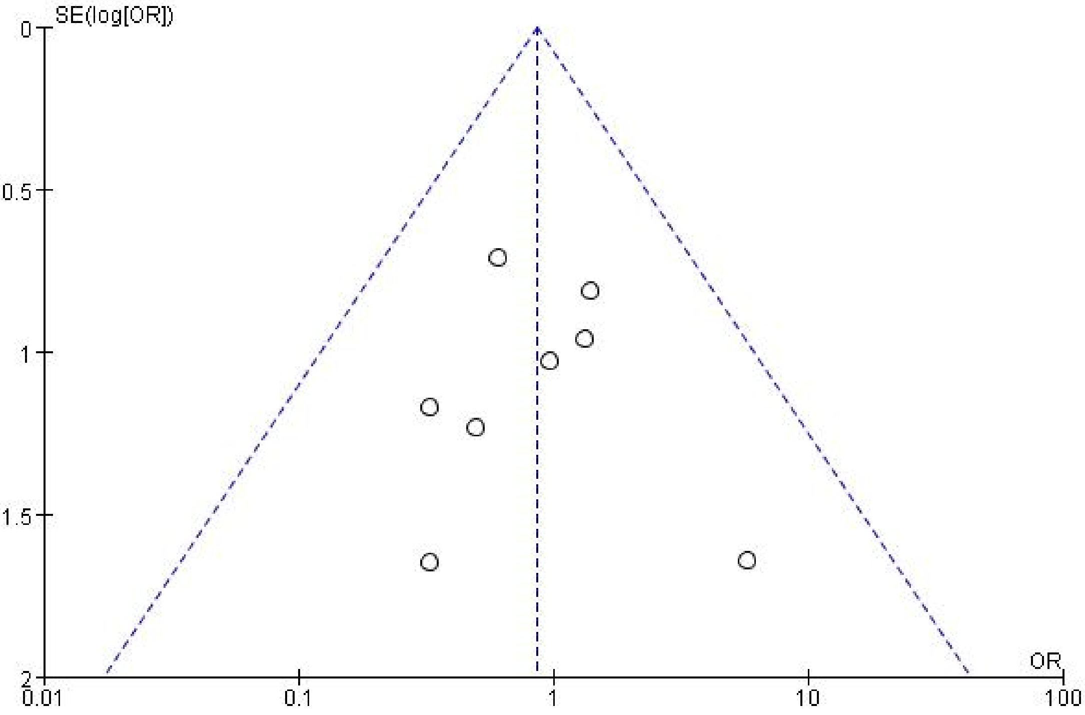

6.10 Respiratory tract infection(Infection)

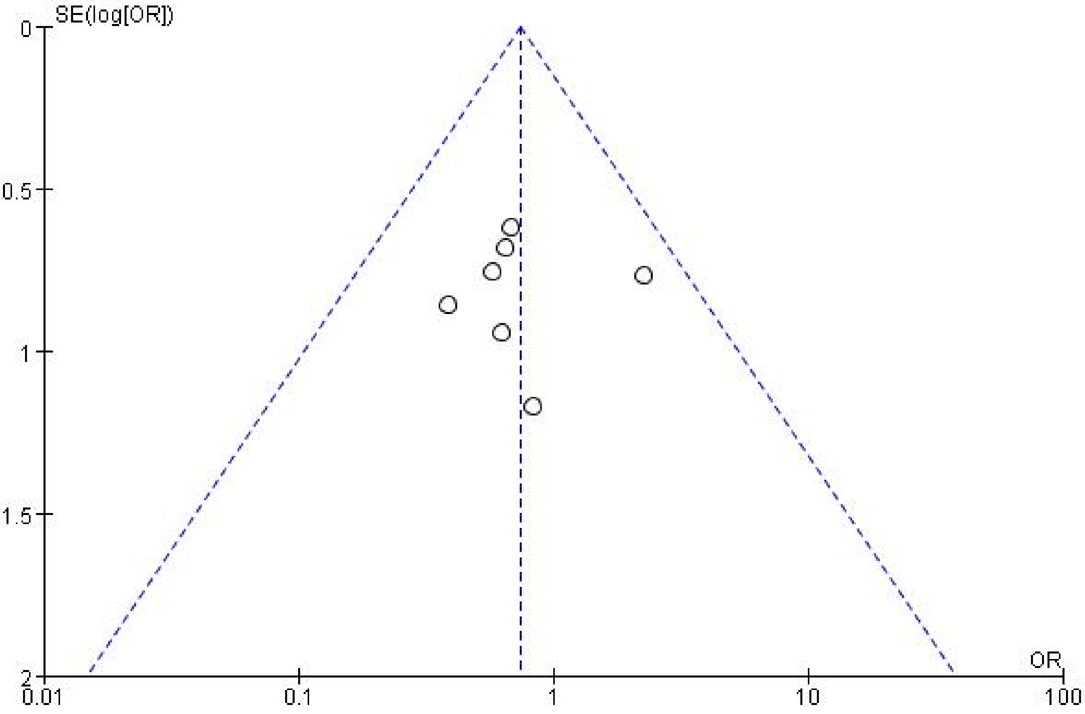

6.11 Urinary tract infection(Infection)

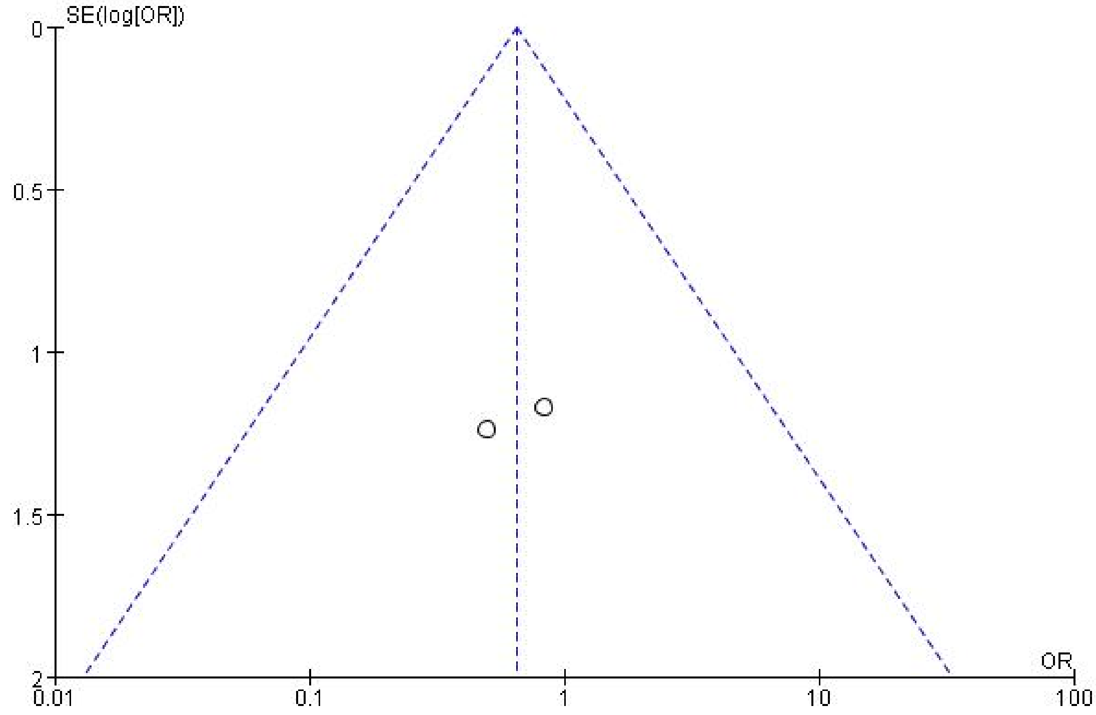

6.12 Abdominal cavity infection(Infection)

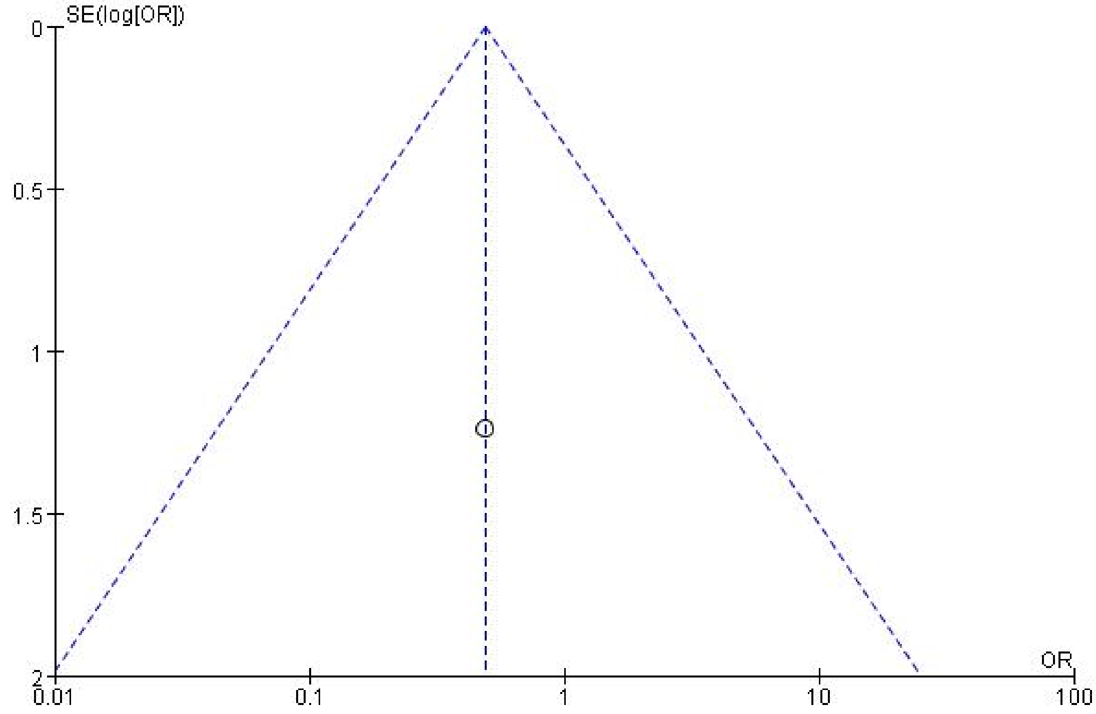

6.13 Sepsis(Infection)

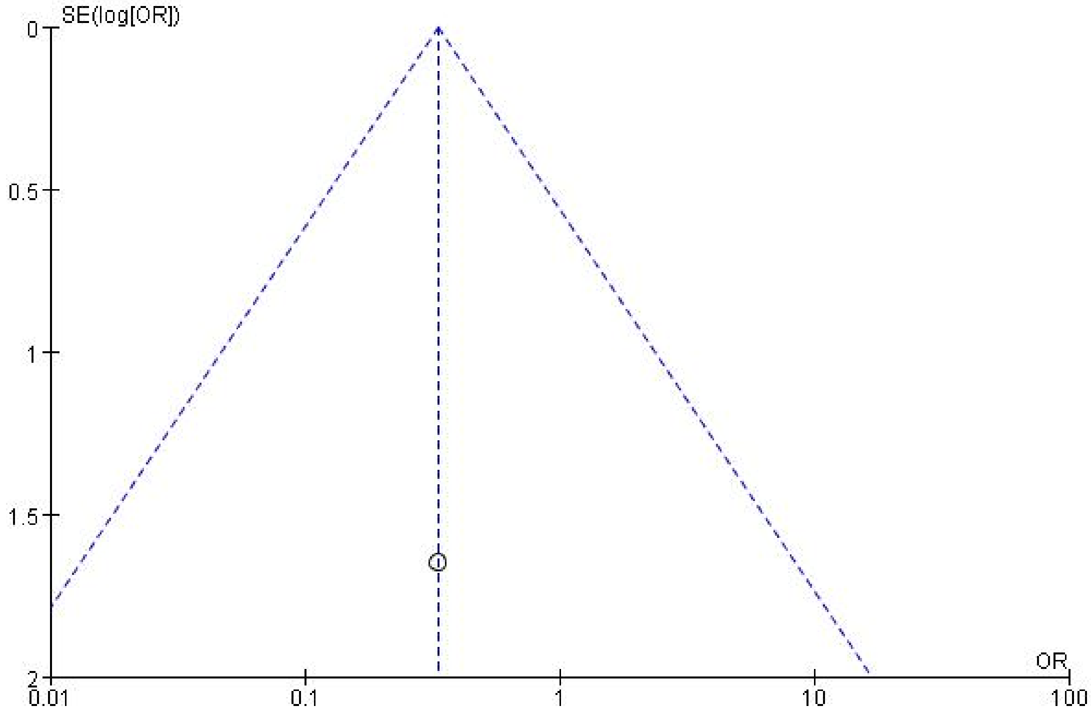

6.14 Abscess(Infection)

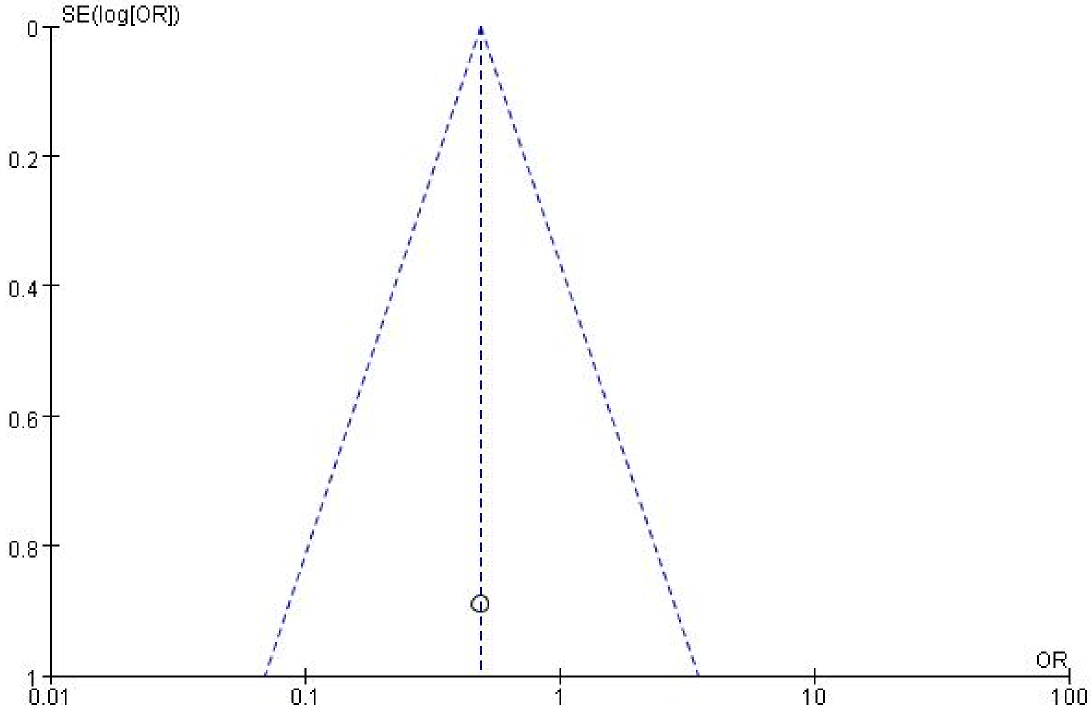

6.15 Anastomosis leakage(Infection)

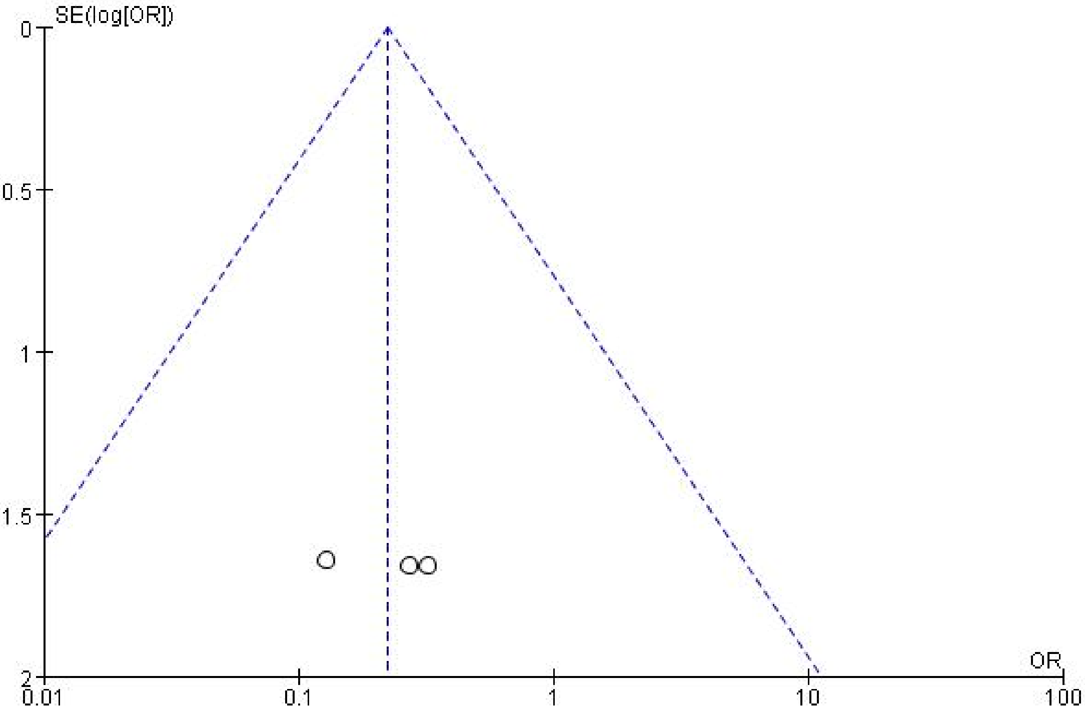

6.16 Fever(Infection)

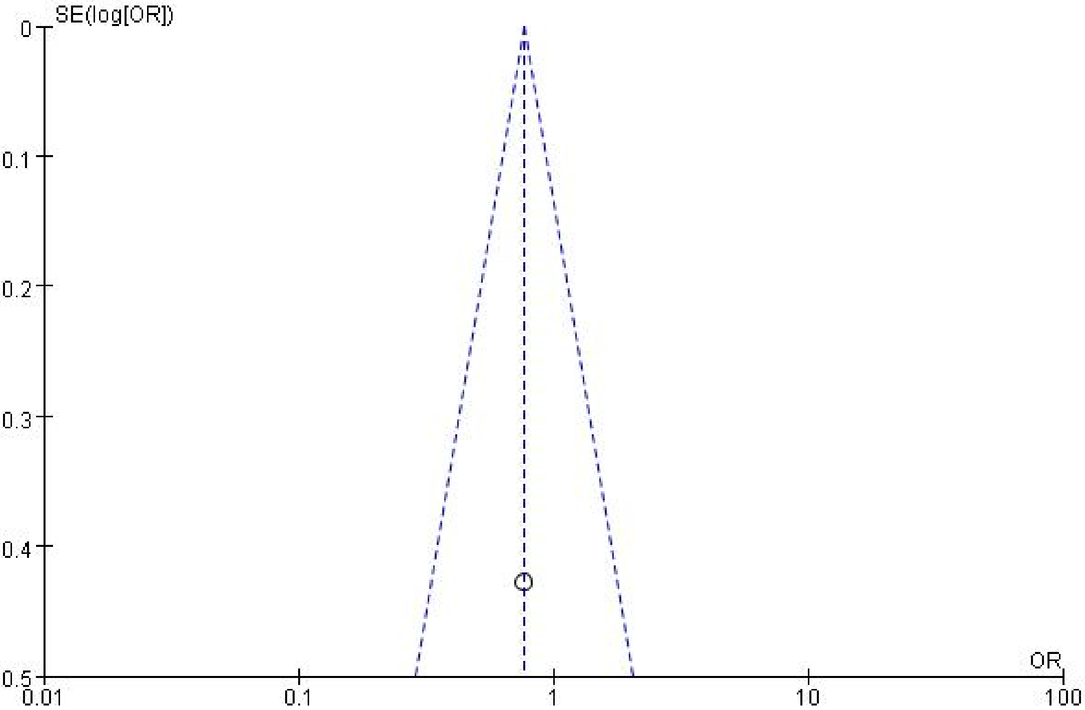

6.17 Total(Skin disorders)

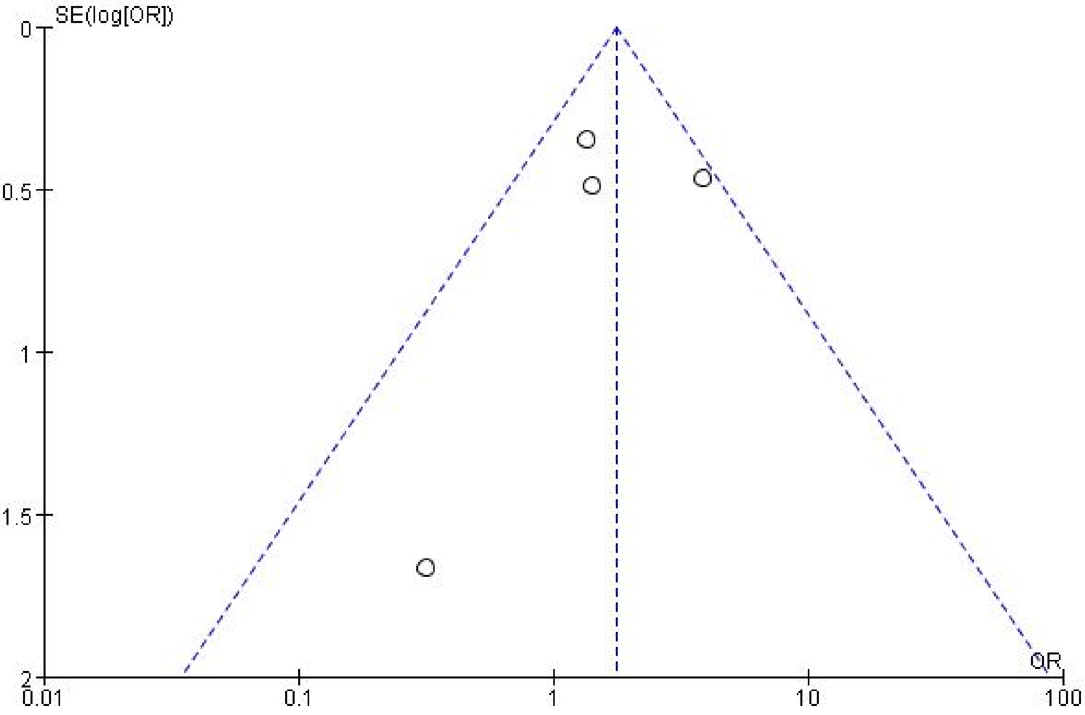

6.18 Hand-foot syndrome(Skin disorders)

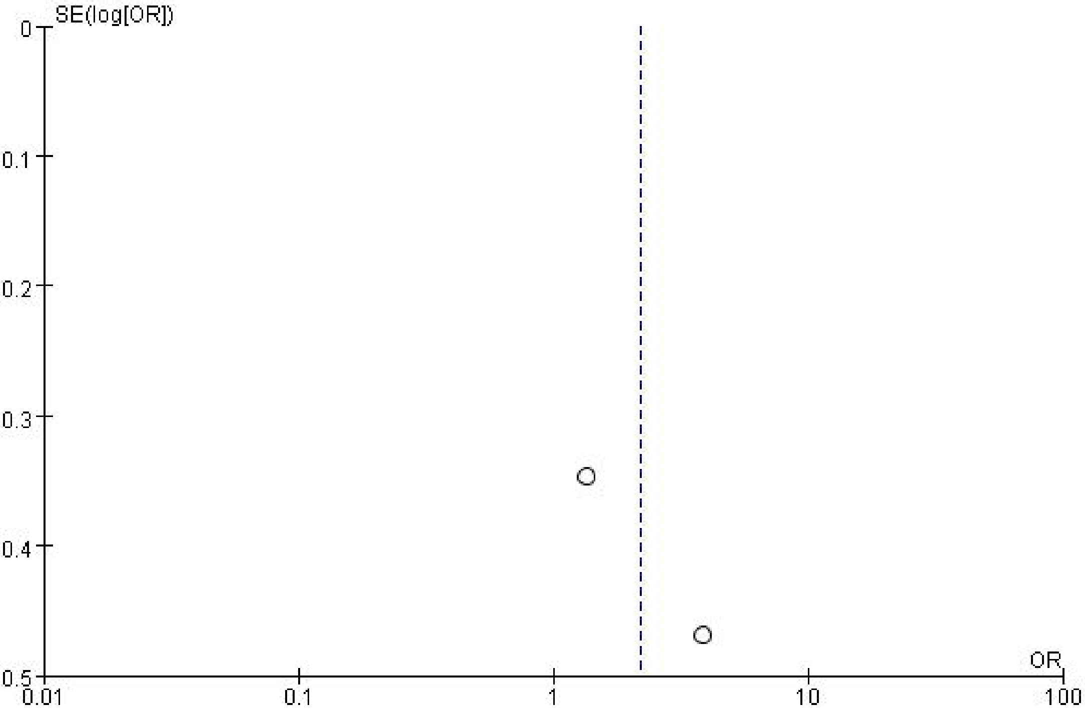

6.19 Itchy skin(Skin disorders)

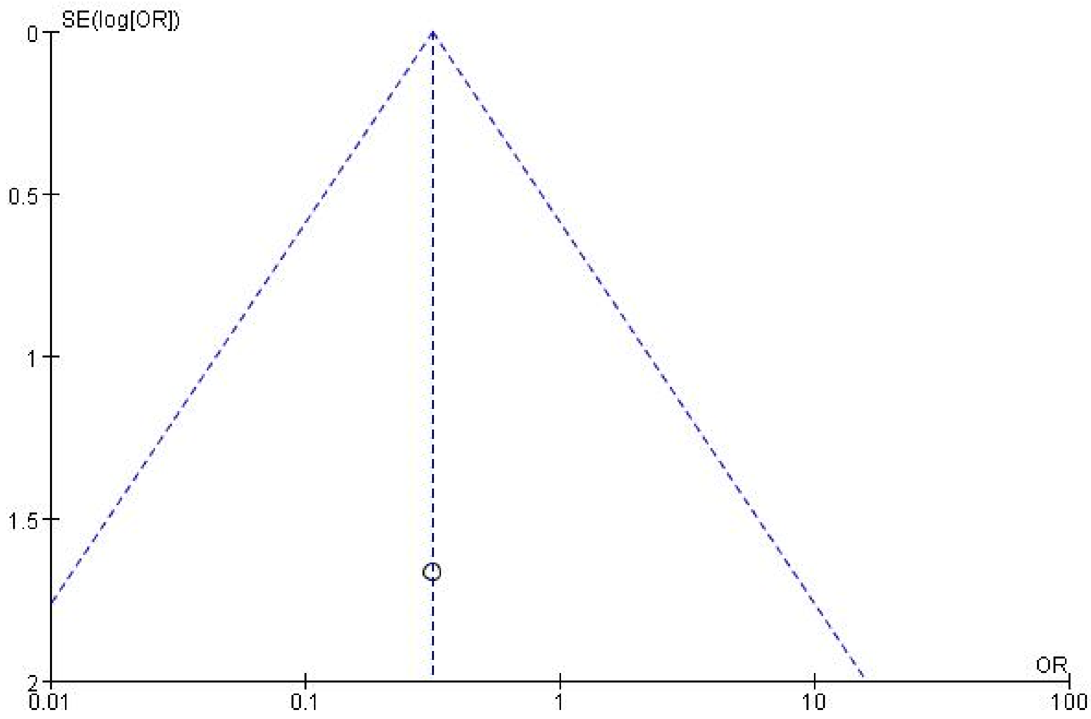

6.20 Hair loss(Skin disorders)

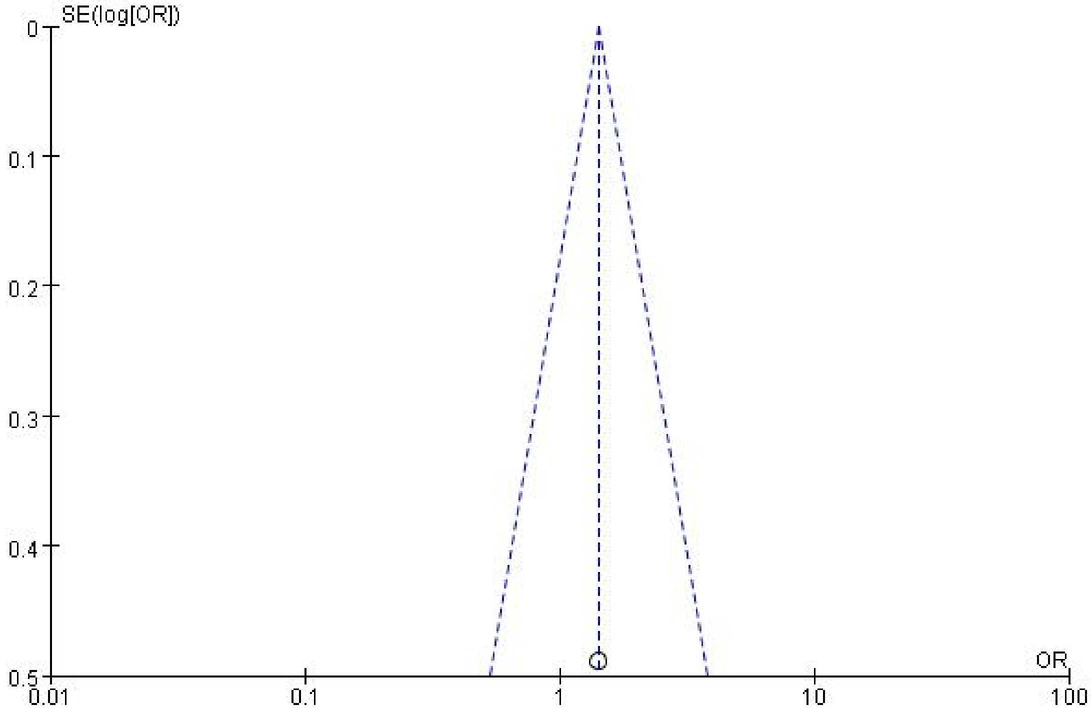

6.21 Total(Abnormal blood pressure)

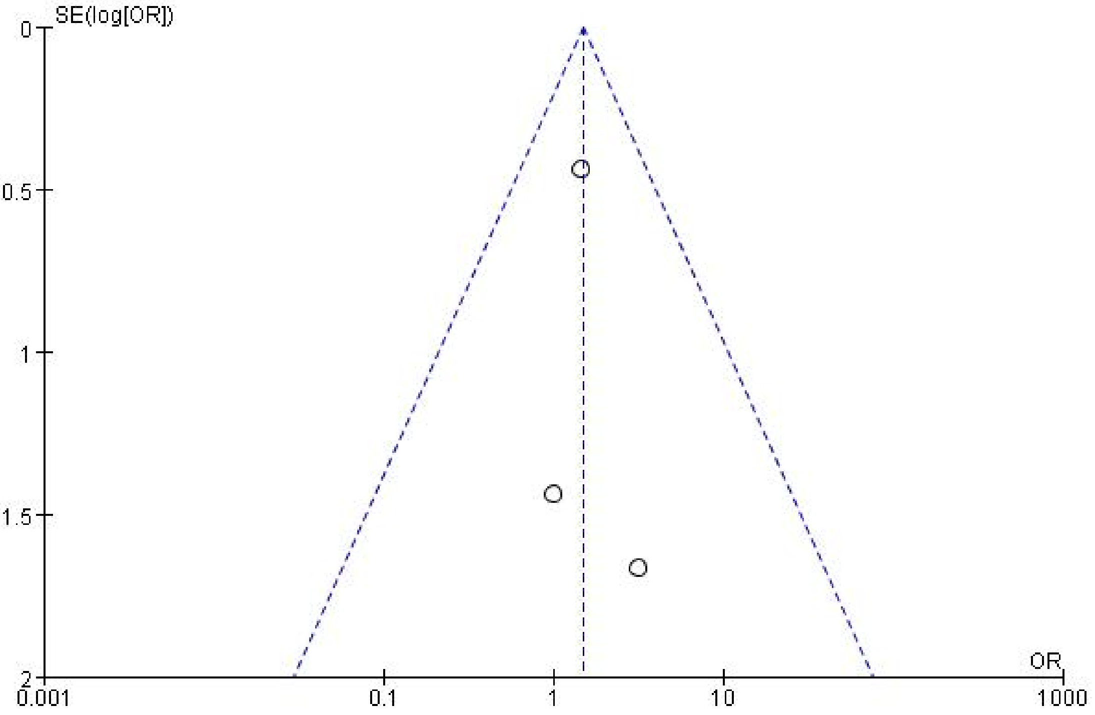

6.22 Hypertension(Abnormal blood pressure)

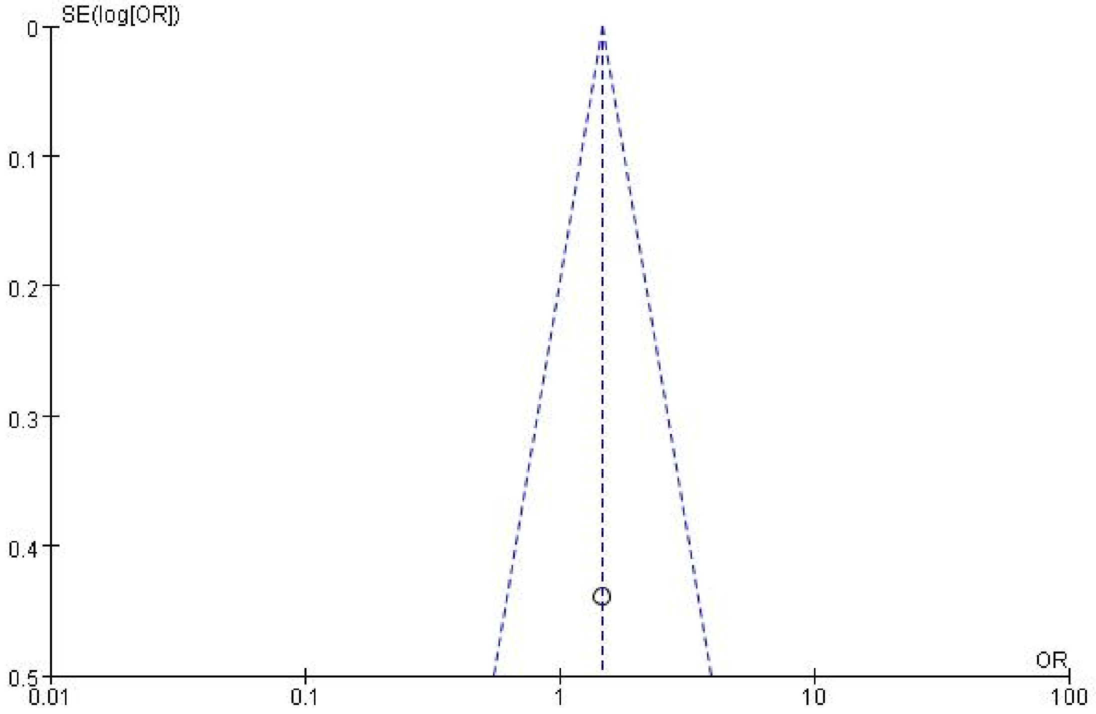

6.23 Hypotension(Abnormal blood pressure)

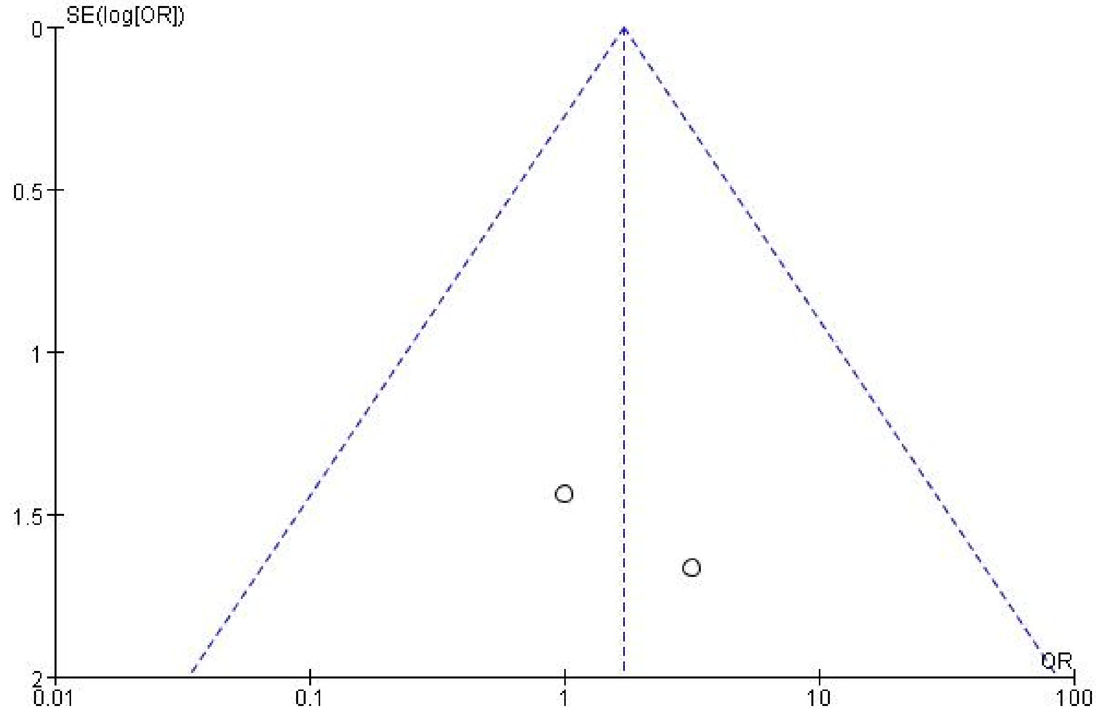

6.24 Total(Nervous system disorders)

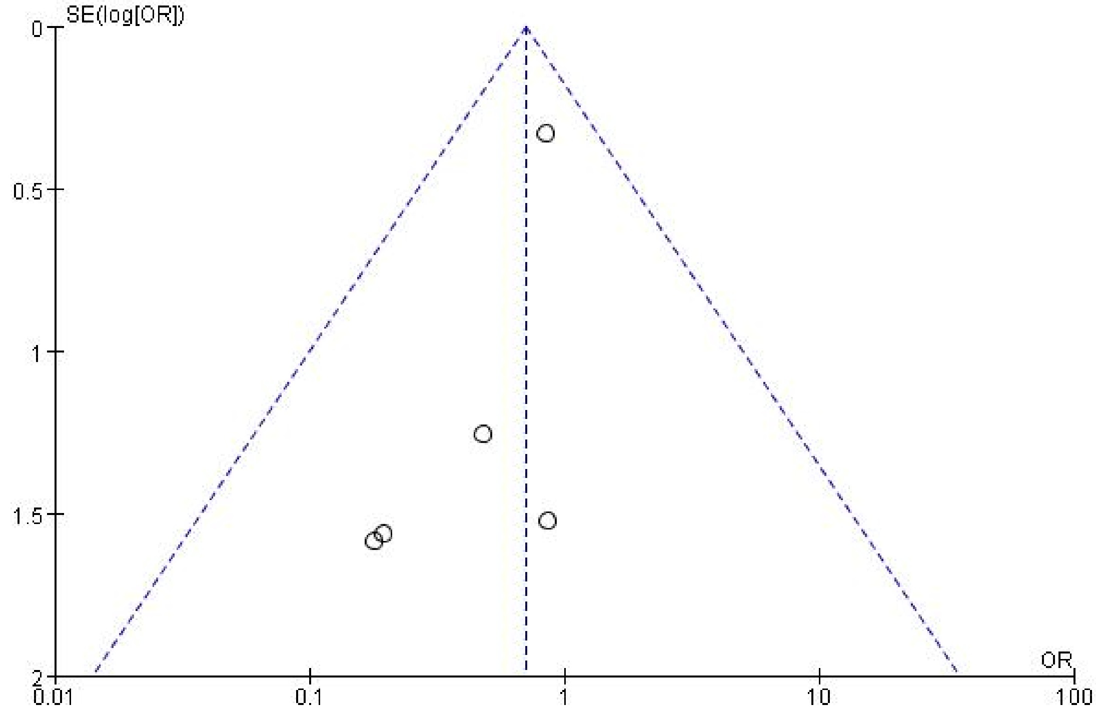

6.25 Pain(Nervous system disorders)

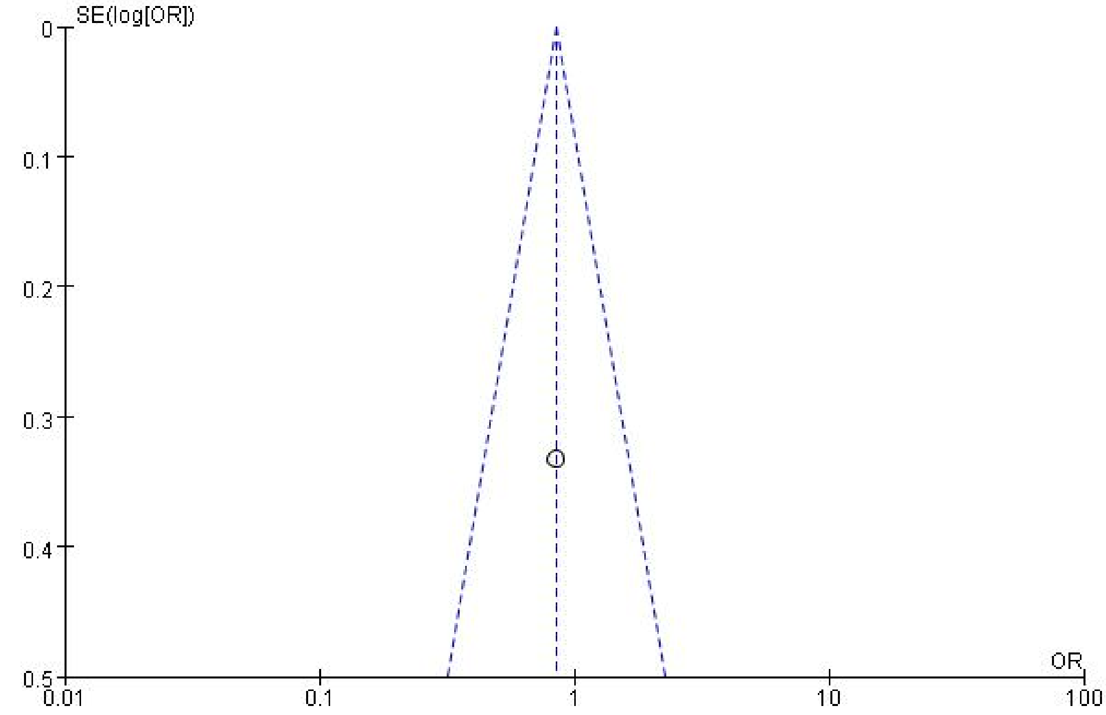

6.26 Neurological dysfunction(Nervous system disorders)

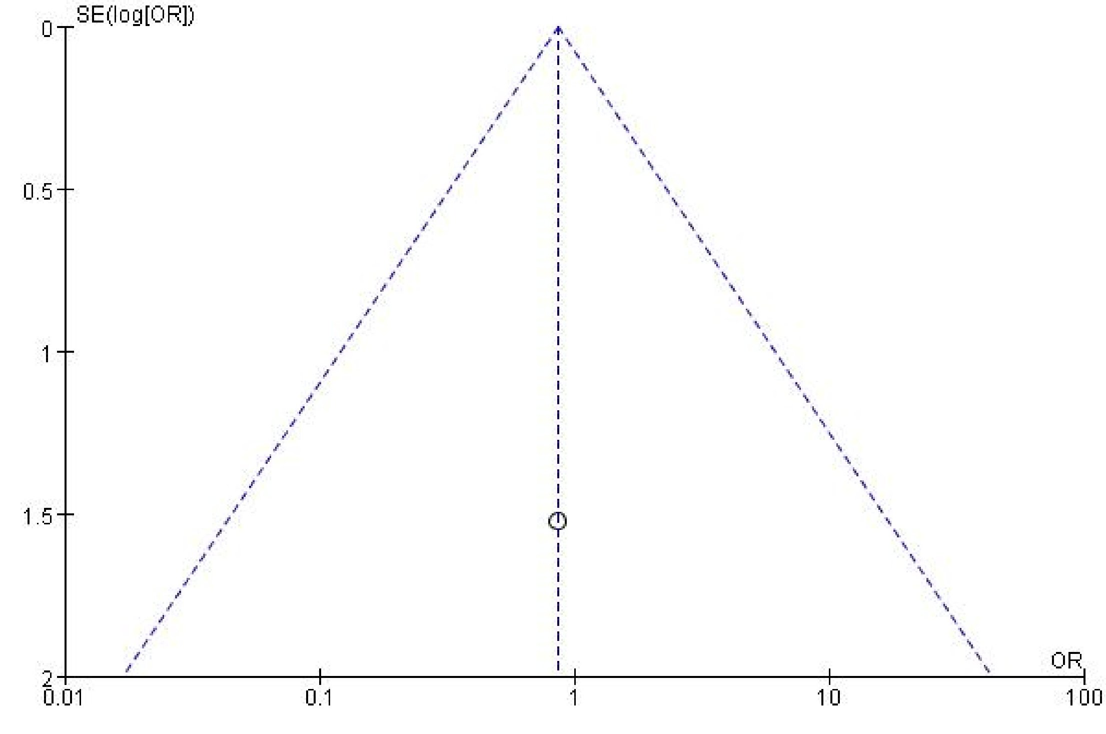

6.27 Dizziness(Nervous system disorders)

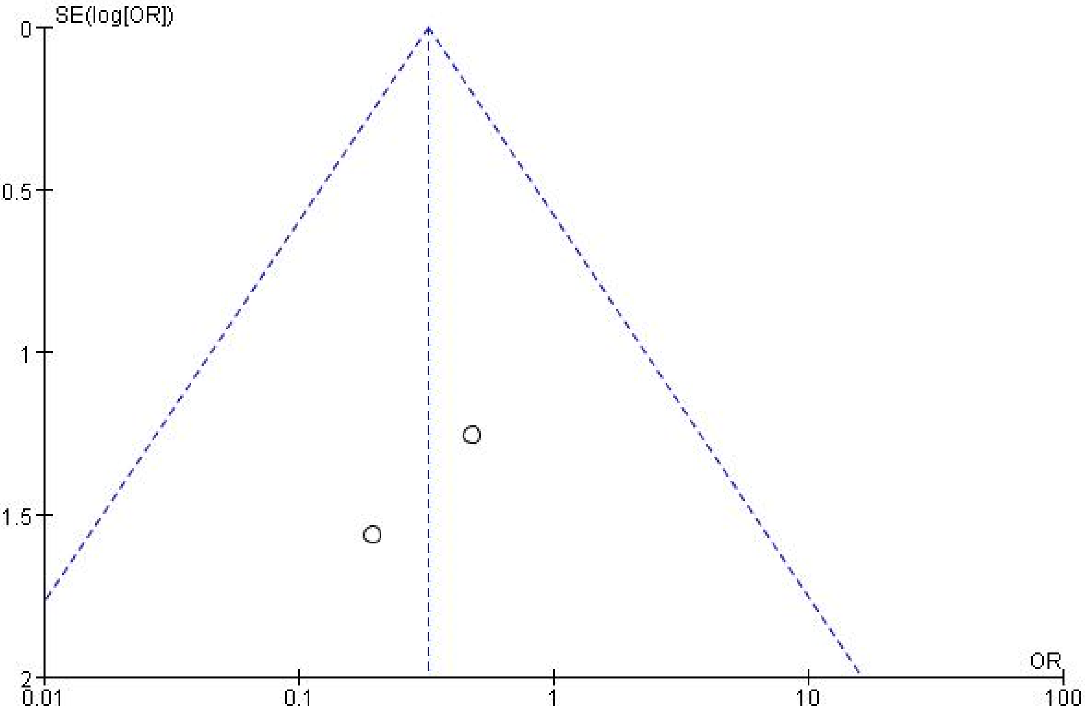

6.28 Respiratory depression(Nervous system disorders)

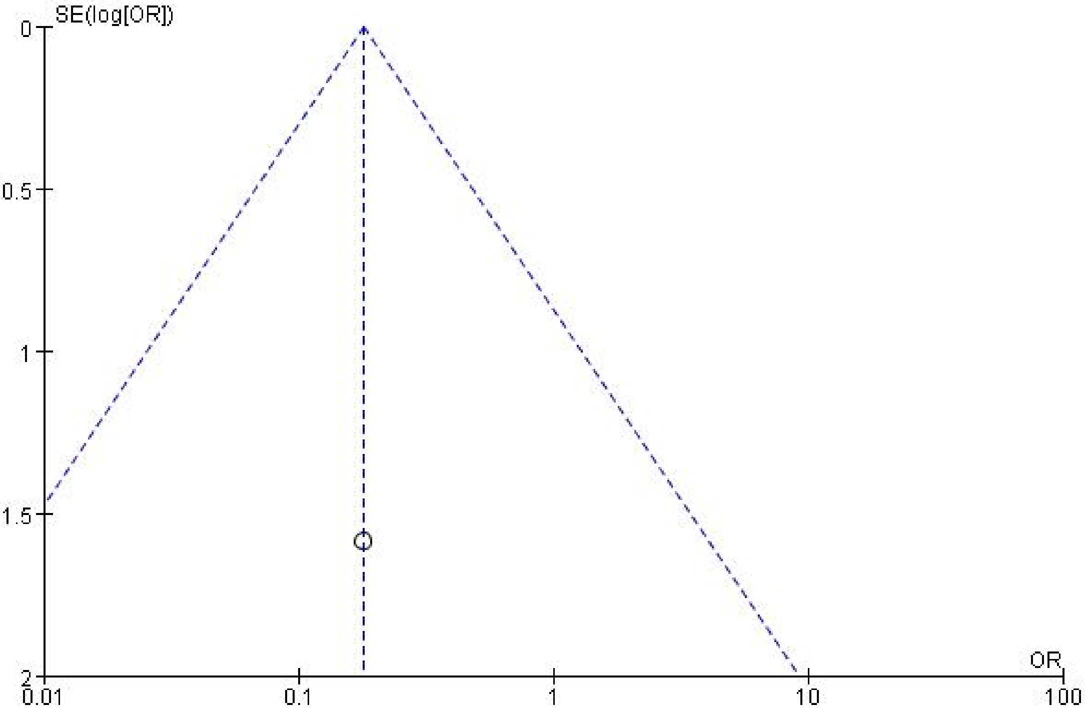

6.29 Total(Other disorders)

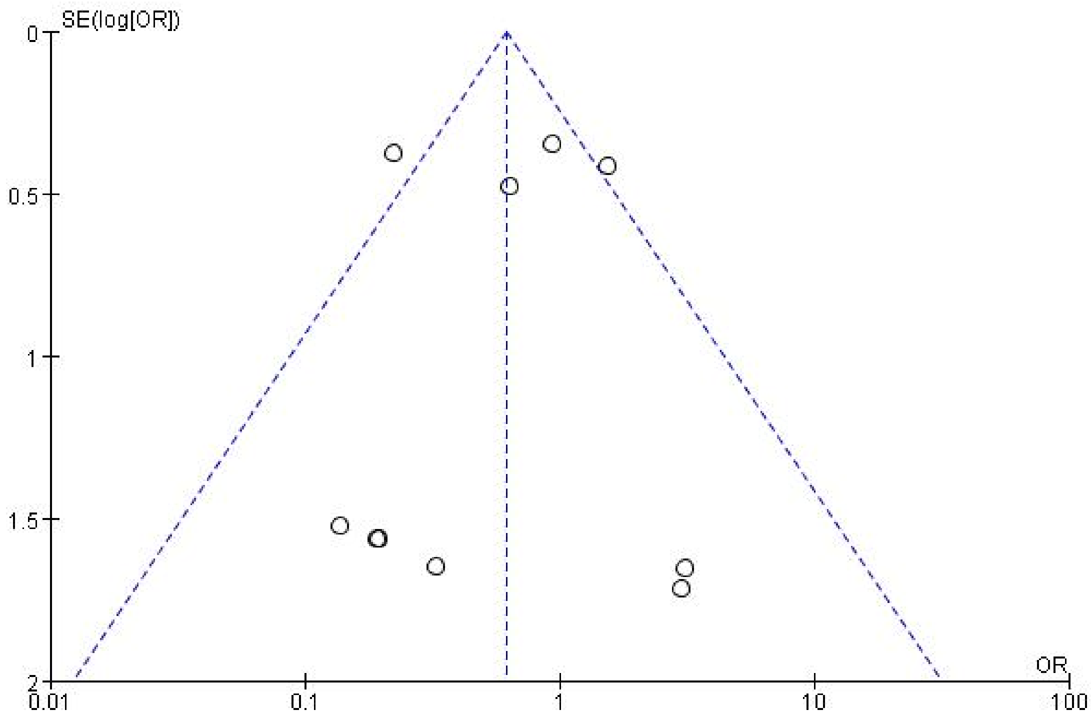

SE(log[OR])
0
0.5
1
1.5
2
OR
0.01
0.1
1
10
100
