# Supplementary material for: What are the beneficial treatment strategies in maintaining T lymphocyte subsets after cancer surgery? A systematic review and network meta-analysis
Source: Front Immunol. 2026 Jul 14;17:1854279. doi: 10.3389/fimmu.2026.1854279 (PMC13408238; doi:10.3389/fimmu.2026.1854279)

Figure S7. Forest plot of dexmedetomidine in the time subgroup.

Dexmedetomidine

CD3≤1day，CD3＞1day，≤1week

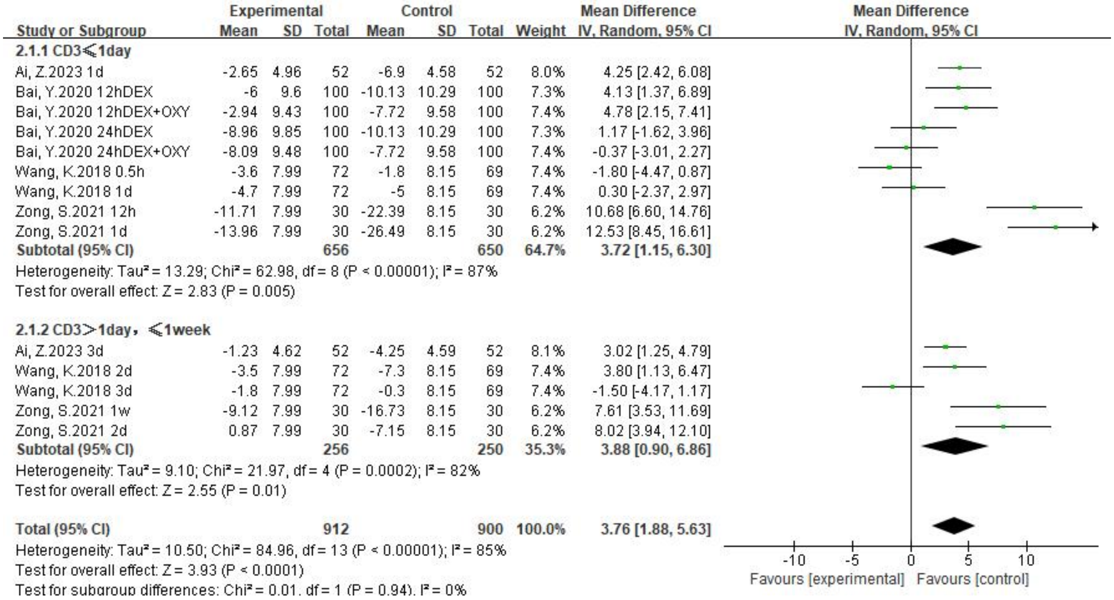

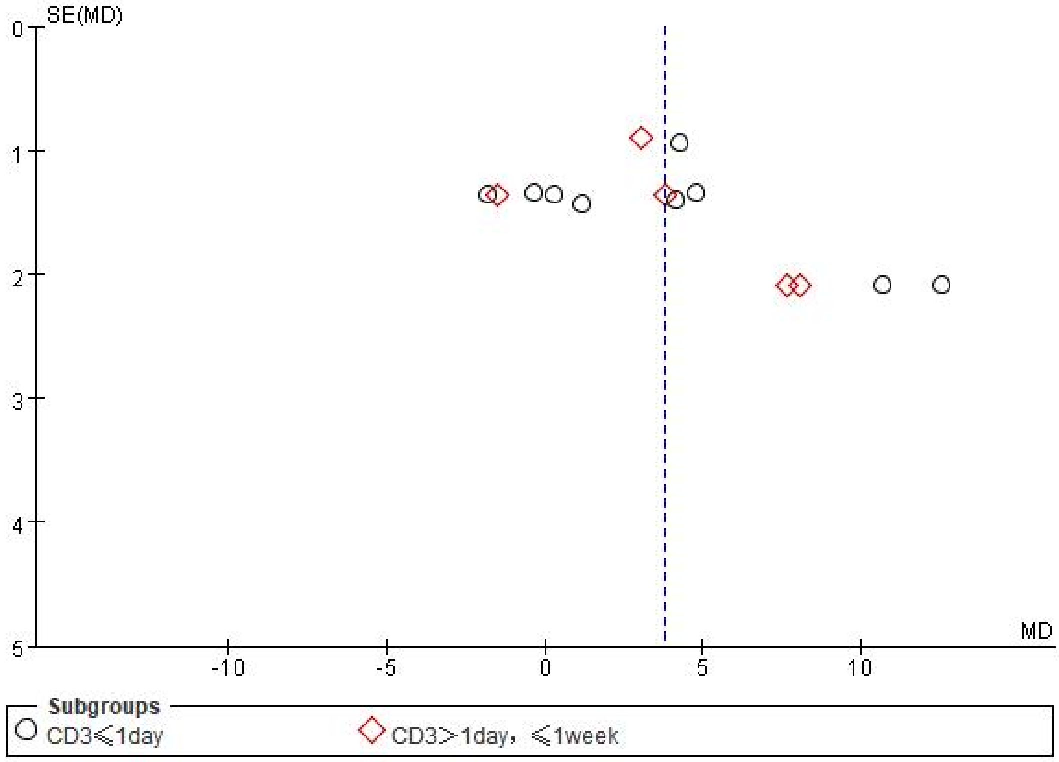

CD4≤1day，CD4＞1day，≤1week

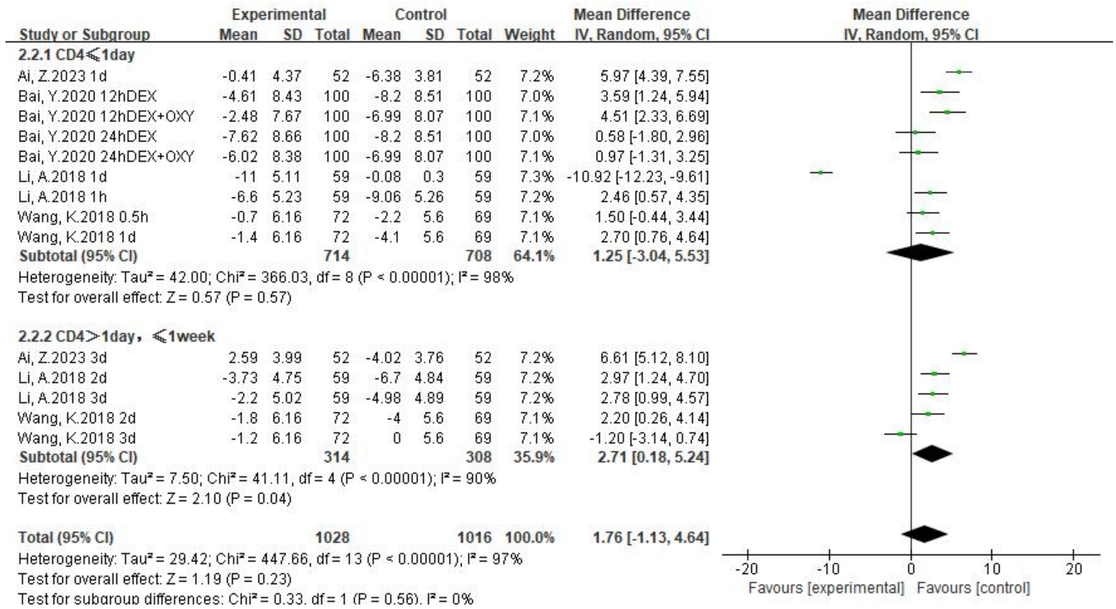

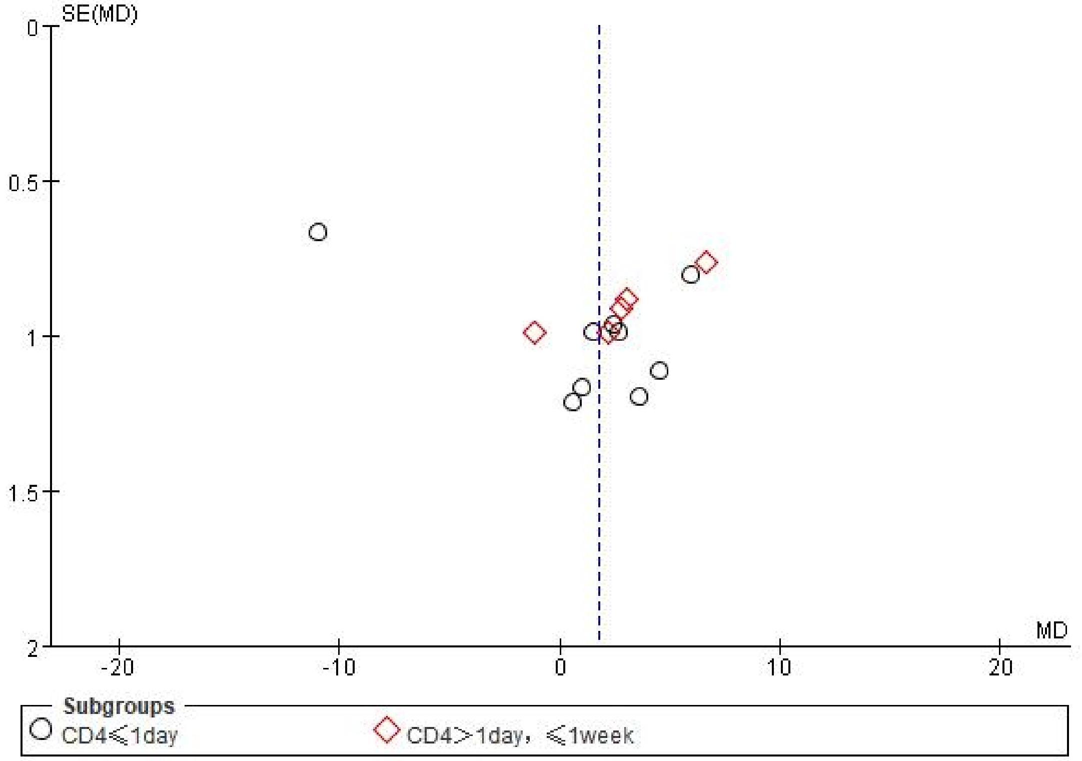

CD8≤1day，CD8＞1day，≤1week

Study or Subgroup	Experimental Mean	Experimental SD	Experimental Total	Control Mean	Control SD	Control Total	Weight	Mean Difference IV, Random, 95% CI
**2.3.1 CD8≤1day**								
Ai, Z.2023 1d	-2.22	1.96	52	-0.57	2.35	52	8.5%	-1.65 [-2.48, -0.82]
Bai, Y.2020 12hDEX	0.46	8.12	100	3.42	7.31	100	6.1%	-2.96 [-5.10, -0.82]
Bai, Y.2020 12hDEX+OXY	-0.41	6.98	100	-0.44	6.11	100	6.7%	0.03 [-1.79, 1.85]
Bai, Y.2020 24hDEX	4.71	9.1	100	3.42	7.31	100	5.8%	1.29 [-1.00, 3.58]
Bai, Y.2020 24hDEX+OXY	1.54	7.97	100	-0.44	6.11	100	6.4%	1.98 [0.01, 3.95]
Li, A.2018 1d	8.93	3.84	59	8.29	4.27	59	7.4%	0.64 [-0.83, 2.11]
Li, A.2018 1h	4.13	3.29	59	7.17	4.51	59	7.5%	-3.04 [-4.46, -1.62]
Wang, K.2018 0.5h	1.1	5.09	72	2.1	4.85	69	7.1%	-1.00 [-2.64, 0.64]
Wang, K.2018 1d	1.2	5.09	72	2.6	4.85	69	7.1%	-1.40 [-3.04, 0.24]
**Subtotal (95% CI)**			**714**			**708**	**62.4%**	**-0.76 [-1.82, 0.30]**
Heterogeneity: Tau² = 1.88; Chi² = 32.32, df = 8 (P < 0.0001); I² = 75%								
Test for overall effect: Z = 1.40 (P = 0.16)								
**2.3.2 CD8>1day，≤1week**								
Ai, Z.2023 3d	-3.83	1.89	52	-0.25	2.74	52	8.4%	-3.58 [-4.48, -2.68]
Li, A.2018 2d	2.22	3.92	59	5	4.14	59	7.4%	-2.78 [-4.23, -1.33]
Li, A.2018 3d	0.8	3.82	59	3.39	3.66	59	7.6%	-2.59 [-3.94, -1.24]
Wang, K.2018 2d	1.4	5.09	72	2.7	4.85	69	7.1%	-1.30 [-2.94, 0.34]
Wang, K.2018 3d	0	5.09	72	-0.9	4.85	69	7.1%	0.90 [-0.74, 2.54]
**Subtotal (95% CI)**			**314**			**308**	**37.6%**	**-1.95 [-3.44, -0.46]**
Heterogeneity: Tau² = 2.36; Chi² = 24.13, df = 4 (P < 0.0001); I² = 83%								
Test for overall effect: Z = 2.57 (P = 0.01)								
**Total (95% CI)**			**1028**			**1016**	**100.0%**	**-1.20 [-2.10, -0.30]**
Heterogeneity: Tau² = 2.30; Chi² = 68.97, df = 13 (P < 0.00001); I² = 81%								
Test for overall effect: Z = 2.61 (P = 0.009)								
Test for subgroup differences: Chi² = 1.64, df = 1 (P = 0.20), I² = 39.0%								

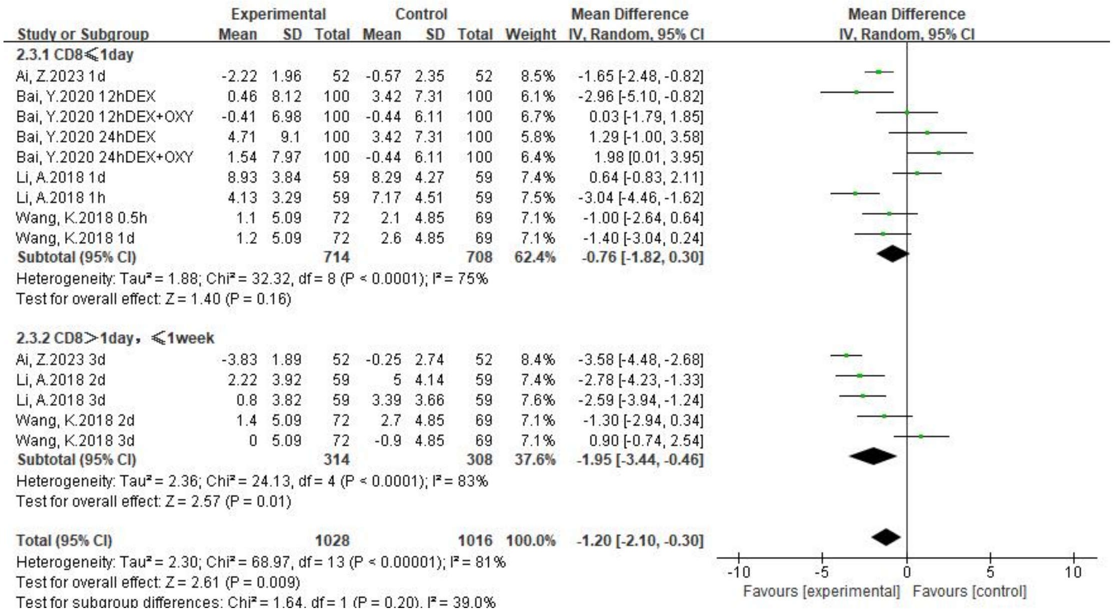

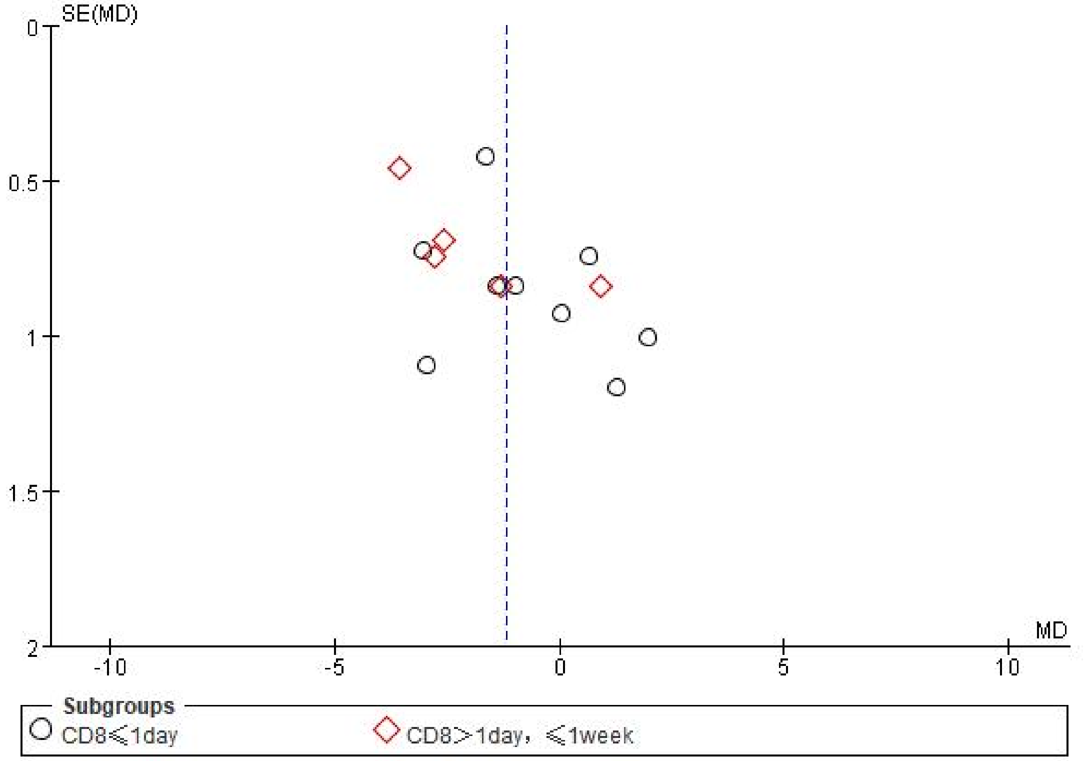

CD4/CD8≤1day，CD4/CD8>1day，≤1week

Study or Subgroup	Experimental Mean	Experimental SD	Experimental Total	Control Mean	Control SD	Control Total	Weight	Mean Difference IV, Random, 95% CI
**2.4.1 CD4/CD8≤1day**								
Ai, Z.2023 1d	0.09	0.16	52	-0.18	0.15	52	6.0%	0.27 [0.21, 0.33]
Bai, Y.2020 12hDEX	-0.17	0.48	100	-0.32	0.47	100	5.6%	0.15 [0.02, 0.28]
Bai, Y.2020 12hDEX+OXY	-0.12	0.39	100	-0.25	0.77	100	5.3%	0.13 [-0.04, 0.30]
Bai, Y.2020 24hDEX	-0.41	0.85	100	-0.32	0.47	100	5.1%	-0.09 [-0.28, 0.10]
Bai, Y.2020 24hDEX+OXY	-0.35	0.56	100	-0.25	0.77	100	5.1%	-0.10 [-0.29, 0.09]
Li, A.2018 1d	-0.84	0.32	59	-0.78	0.18	59	5.8%	-0.06 [-0.15, 0.03]
Li, A.2018 1h	-0.52	0.028	59	-0.71	0.21	59	6.0%	0.19 [0.14, 0.24]
Wang, K.2018 0.5h	-0.3	0.35	72	-0.1	0.36	69	5.7%	-0.20 [-0.32, -0.08]
Wang, K.2018 1d	-0.4	0.35	72	-0.4	0.36	69	5.7%	0.00 [-0.12, 0.12]
Zong, S.2021 12h	0.19	0.35	30	-0.48	0.36	30	5.2%	0.67 [0.49, 0.85]
Zong, S.2021 1d	0.16	0.35	30	-0.64	0.36	30	5.2%	0.80 [0.62, 0.98]
**Subtotal (95% CI)**			**774**			**768**	**60.6%**	**0.16 [0.02, 0.29]**
Heterogeneity: Tau² = 0.05; Chi² = 172.71, df = 10 (P < 0.00001); I² = 94%								
Test for overall effect: Z = 2.25 (P = 0.02)								
**2.4.2 CD4/CD8＞1day，≤1week**								
Ai, Z.2023 3d	0.28	0.15	52	-0.11	0.17	52	6.0%	0.39 [0.33, 0.45]
Li, A.2018 2d	-0.32	0.28	59	-0.52	0.17	59	5.9%	0.20 [0.12, 0.28]
Li, A.2018 3d	-0.08	0.3	59	-0.31	0.24	59	5.8%	0.23 [0.13, 0.33]
Wang, K.2018 2d	-0.2	0.35	72	-0.4	0.36	69	5.7%	0.20 [0.08, 0.32]
Wang, K.2018 3d	-0.4	0.35	72	0	0.36	69	5.7%	-0.40 [-0.52, -0.28]
Zong, S.2021 1w	0.13	0.35	30	-0.33	0.36	30	5.2%	0.46 [0.28, 0.64]
Zong, S.2021 2d	0.17	0.35	30	-0.19	0.36	30	5.2%	0.36 [0.18, 0.54]
**Subtotal (95% CI)**			**374**			**368**	**39.4%**	**0.20 [0.01, 0.40]**
Heterogeneity: Tau² = 0.06; Chi² = 145.82, df = 6 (P < 0.00001); I² = 96%								
Test for overall effect: Z = 2.04 (P = 0.04)								
**Total (95% CI)**			**1148**			**1136**	**100.0%**	**0.17 [0.07, 0.28]**
Heterogeneity: Tau² = 0.05; Chi² = 327.46, df = 17 (P < 0.00001); I² = 95%								
Test for overall effect: Z = 3.17 (P = 0.002)								
Test for subgroup differences: Chi² = 0.15, df = 1 (P = 0.70), I² = 0%								

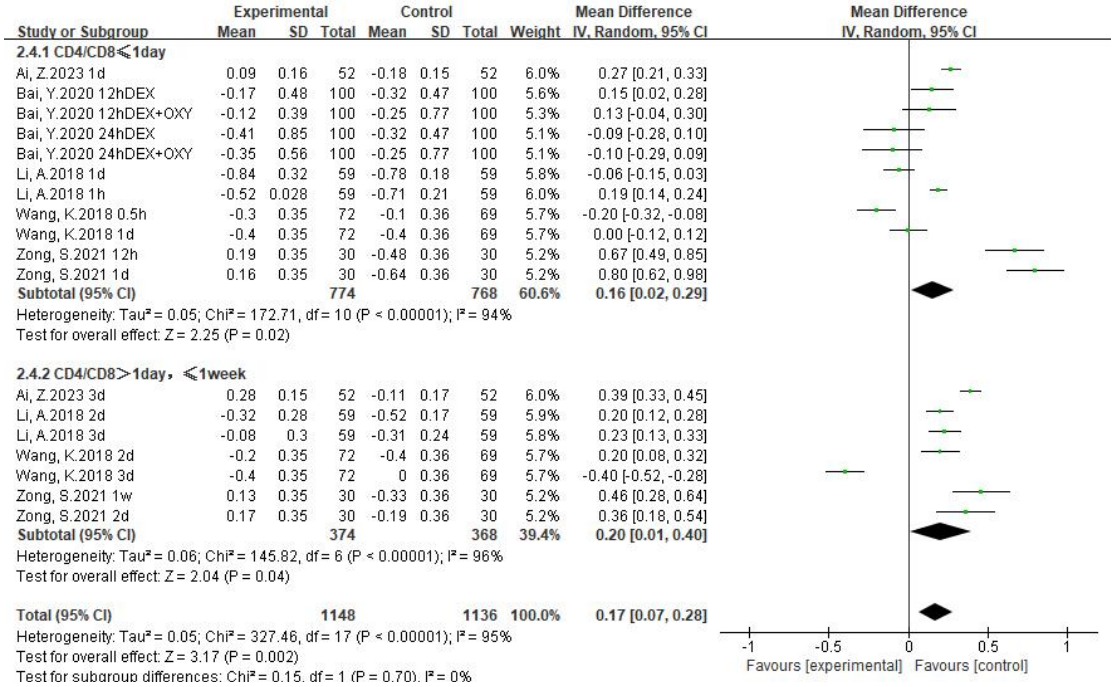

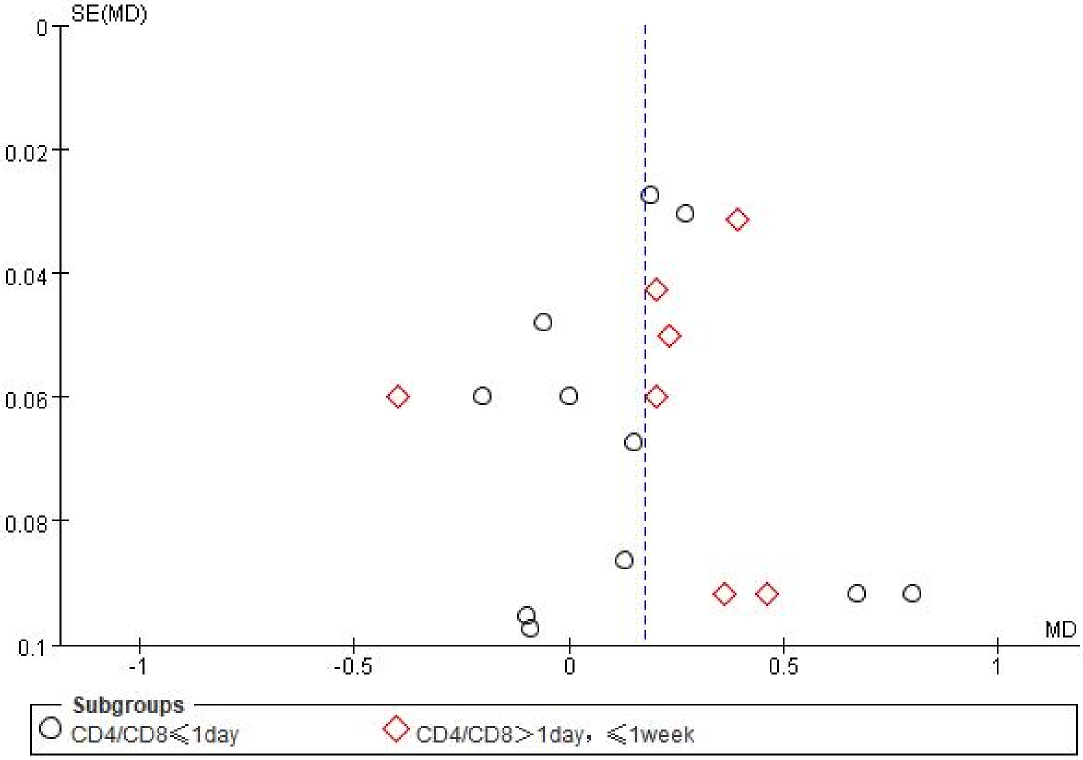

Supplement: Supplementary file 7 [file DataSheet7.pdf]
