# Supplementary material for: What are the beneficial treatment strategies in maintaining T lymphocyte subsets after cancer surgery? A systematic review and network meta-analysis
Source: Front Immunol. 2026 Jul 14;17:1854279. doi: 10.3389/fimmu.2026.1854279 (PMC13408238; doi:10.3389/fimmu.2026.1854279)
Supplement: Supplementary file 8 [file DataSheet8.pdf]

NIHR | National Institute for Health and Care Research

PROSPERO
International prospective register of systematic reviews

What are the benificial treatment strategies of maintaining T lymphocyte subsets after cancer surgery? A systematic review and network meta-analysis

Ziang Xu, Yingshi Zhang, Qingchun Zhao

Citation
Ziang Xu, Yingshi Zhang, Qingchun Zhao. What are the benificial treatment strategies of maintaining T lymphocyte subsets after cancer surgery? A systematic review and network meta-analysis. PROSPERO 2025 CRD420251233455. Available from https://www.crd.york.ac.uk/PROSPERO/view/CRD420251233455.

REVIEW TITLE AND BASIC DETAILS

Review title
What are the benificial treatment strategies of maintaining T lymphocyte subsets after cancer surgery? A systematic review and network meta-analysis

Condition or domain being studied
Cancer; Lymphocytes

Rationale for the review

- This systematic review and network meta-analysis is conducted because the current understandng of how diverse cancer treatments comparatively affect immune function remains fragmented. While individual interventions are studied in isolation, clinicians lack a unified hierarchy to guide immune-protective strategy selection across treatment modalities.This review will synthesize all available direct and indirect evidence to rank the efficacy of systemic therapies, nutritional support, anesthesia, and analgesia on key immune parameters like CD4+ counts and CD4/CD8 ratio in solid cancer patients. It will thereby provide the first comprehensive, cross-comparative assessment, identifying the most immunologically favorable strategies. The findings will fill a critical knowledge gap, offering an evidence-based framework to integrate immune preservation into multidisciplinary cancer care, potentially improving treatment tolerance and patient outcomes.

Review objectives

1. What is the comparative efficacy of different Systemic Anti-Cancer Therapies (SACT)—such as chemotherapy, targeted therapy, and immunotherapy—on preserving or enhancing immune parameters (CD3+, CD4+, CD8+ counts, CD4/CD8 ratio, and NK cells) in solid cancer patients?
2. How do various perioperative nutritional support strategies—including enteral nutrition, parenteral nutrition, and immunonutrition—compare in their ability to mitigate treatment-induced immunosuppression?
3. What is the relative impact of different anaesthetic techniques (e.g., total intravenous anaesthesia vs. regional blocks) and analgesic protocols (e.g., dexmedetomidine-based vs. opioid-based regimens) on postoperative immune competence?
4. Among all evaluated interventions across therapeutic domains, which are the most effective for maintaining or improving CD4+ T-cell counts and the CD4/CD8 ratio?
5. What is the safety profile, in terms of specific adverse events, of the interventions identified as most efficacious for immune potentiation?

Keywords
Solid tumors; T-Lymphocytes; Nutritional support; Anesthesia; Analgesia; Immunomodulation

Country
China

ELIGIBILITY CRITERIA

Population

Included
Inclusion: Adult patients (⩾18 years of age) with a confirmed diagnosis of any solid tumor malignancy (e.g., gastric cancer, non-small cell lung cancer, colorectal cancer), regardless of cancer stage, who are receiving active oncologic treatment or perioperative care.

Exclusion: Pediatric or adolescent populations (<18 years), individuals with known active autoimmune diseases, and those with concurrent HIV infection or other congenital immunodeficiency syndromes.

Intervention(s) or exposure(s)

Included
Enteral nutrition; Parenteral nutrition; Chemotherapy; Targeted Therapy; Transarterial chemoembolization of hepatic artery; Dexmedetomidine

Comparator(s) or control(s)

Included
PICO tags selected: Placebo; Surgery; Usual Care

Study design
Both randomized and nonrandomized study types will be included.

Excluded
Narrative reviews

Editorials

Commentaries

In vitro studies

Animal studies

Conference abstracts

Context

Timing of Outcome Measurement: To be included, studies must report immune outcomes measured during or after the intervention. Studies reporting only baseline (pre-intervention) immune data will be excluded.

Geographic Location: Studies from all geographic locations and income-level countries will be eligible for inclusion.

TIMELINE OF THE REVIEW

Date of first submission to PROSPERO

17 November 2025

Review timeline

Start date: 1 March 2025. End date: 17 December 2025.

Date of registration in PROSPERO

20 November 2025

AVAILABILITY OF FULL PROTOCOL

Availability of full protocol

A full protocol has been written but is not available because:

Making the current version publicly available at this stage could lead to confusion if minor administrative adjustments are made during the project's execution.

SEARCHING AND SCREENING

Search for unpublished studies

Both published and unpublished studies will be sought.

Main bibliographic databases that will be searched

The main databases to be searched are *CENTRAL - Cochrane Central Register of Controlled Trials*, *Embase - Embase via Ovid*, *Embase.com* and *PubMed*.

Search language restrictions

There are no language restrictions.

Search date restrictions

Databases will be searched for articles published from 1 January 2003, there are no search end date restrictions.

Other methods of identifying studies

Other studies will be identified by: *contacting authors or experts* and *searching trial or study registers*.

Link to search strategy

A full search strategy is available in the full protocol as described in the *Availability of full protocol* section

Selection process

Studies will be screened independently by at least two people (or person/machine combination) with a process to resolve differences.

Other relevant information about searching and screening

None

DATA COLLECTION PROCESS

Data extraction from published articles and reports

Data will be extracted independently by at least two people (or person/machine combination) with a process to resolve differences.

Authors will be asked to provide any required data not available in published reports.

Study datasets/IPD will be obtained from study investigators or via a data repository

Study risk of bias or quality assessment

Risk of bias will be assessed using: *Cochrane RoB-2*

Data will be assessed independently by at least two people (or person/machine combination) with a process to resolve differences.

Additional information will be sought from study investigators if required information is unclear or unavailable in the study publications/reports.

Reporting bias assessment

Risk of bias due to missing results will be assessed

Certainty assessment

The certainty (or confidence) in the body of evidence for each outcome will be assessed using the Grading of Recommendations, Assessment, Development, and Evaluations (GRADE) framework.

OUTCOMES TO BE ANALYSED

Main outcomes

1. CD4+ T cells

Definition: The absolute count or percentage of CD4+ T lymphocytes in peripheral blood

Measurement: Flow cytometry

Time points: baseline, during treatment, at the end of treatment

Effect size: Mean Difference (MD) or standardized mean difference (SMD)

2. CD3+ T cells

Definition: Total T lymphocyte count in peripheral blood

Measurement: Flow cytometry

Time points: baseline, during treatment, at the end of treatment

Effect size: Mean Difference (MD) or standardized mean difference (SMD)

3. CD8+ T cells

Definition: Count of CD8+ T lymphocytes in peripheral blood

Measurement: Flow cytometry

Time points: baseline, during treatment, at the end of treatment

Effect size: Mean Difference (MD) or standardized mean difference (SMD)

4. CD4+/CD8+ ratio

Definition: The ratio of CD4+ to CD8+ T cells

Measurement: Calculated by flow cytometry

Time points: baseline, during treatment, at the end of treatment

Effect size: Mean Difference (MD) or standardized mean difference (SMD)

5. NK cell count

Definition: Count of natural killer cells in peripheral blood

Measurement: Flow cytometry (CD3-CD56+/CD16+)

Time points: baseline, during treatment, at the end of treatment

Effect size: Mean Difference (MD) or standardized mean difference (SMD)

6. Treat related adverse events

Definition: The incidence of adverse events classified by organ systems

Measurement: Extracted from the research report

Time points: During the treatment period and the follow-up period

Effect size: Odds ratio (OR)

Additional outcomes

There are no additional outcomes.

PLANNED DATA SYNTHESIS

Strategy for data synthesis

The data will be combined using an integrated statistical framework that incorporates both pairwise meta-analysis and network meta-analysis (NMA):

Pairwise Meta-Analysis:

For continuous outcomes (T-cell counts, ratios), data will be pooled using Mean Difference (MD) or Standardized Mean Difference (SMD) with 95% confidence intervals, depending on the consistency of measurement units across studies.

For dichotomous outcomes (adverse events), data will be pooled using the Mantel-Haenszel method to calculate Odds Ratios (OR) with 95% confidence intervals.

Random-effects models will be used as the primary analysis to account for expected clinical and methodological heterogeneity.

Network Meta-Analysis (NMA):

A frequentist approach will be implemented using R software (v4.5.1) with the netmeta package.

A multivariate random-effects model will be fitted under the consistency assumption to incorporate both direct and indirect evidence across the treatment network.

The geometry of the evidence network will be visualized using network plots, where connection thickness corresponds to the number of direct comparisons.

Treatment Ranking:

Interventions will be ranked for each outcome using the Surface Under the Cumulative Ranking Curve (SUCRA) and mean ranks, with uncertainty presented via rankograms.

Handling of Heterogeneity & Inconsistency:

Heterogeneity in pairwise comparisons will be quantified using the I^2 statistic.

Global and local approaches (e.g., side-splitting method) will be used to evaluate inconsistency between direct and indirect evidence.

Missing Data:

When necessary, multiple imputation methods will be employed to handle missing data, following attempts to contact original study authors.

CURRENT REVIEW STAGE

Stage of the review at this submission

Review stage	Started	Completed
Pilot work	✓	
Formal searching/study identification	✓	
Screening search results against inclusion criteria	✓	
Data extraction or receipt of IPD		
Risk of bias/quality assessment		
Data synthesis		

Review status

The review is currently planned or ongoing.

Publication of review results

Results of the review will be published in English.

REVIEW AFFILIATION, FUNDING AND PEER REVIEW

Review team members

Ziang Xu (review guarantor and contact) ORCID: 0009-0001-5617-7273. Shenyang Pharmaceutical University. China.

No conflict of interest declared.

Dr Yingshi Zhang. Shenyang Pharmaceutical University. China.

No conflict of interest declared.

Professor Qingchun Zhao. General Hospital of Northern Theater Command. China.

No conflict of interest declared.

Named contact

Ziang Xu (Xza13842350393@163.com). ORCID: 0009-0001-5617-7273. Shenyang Pharmaceutical University. China.

Review affiliation

General Hospital of Northern Theater Command.100016

Funding source

Review has no funding and no agreed support from an academic institution and is done in authors' own time.

Peer review

There has been no peer review of this planned review.

ADDITIONAL INFORMATION

Review conflict of interest

Declared individual interests are recorded under team member details.. No additional interests are recorded for this review.

Medical Subject Headings

Analgesics, Opioid; Anesthesia, Intravenous; Anesthetics; CD4-CD8 Ratio; CD4-Positive T-Lymphocytes; CD8-Positive T-Lymphocytes; Dexmedetomidine; Enteral Nutrition; Humans; Immunonutrition Diet; Immunosuppression Therapy; Immunotherapy; Killer Cells, Natural; Meta-Analysis as Topic; Network Meta-Analysis; Nutritional Support; Parenteral Nutrition; Systematic Reviews as Topic; T-Lymphocyte Subsets

SIMILAR REVIEWS

Check for similar records already in PROSPERO

PROSPERO identified a number of existing PROSPERO records that were similar to this one (last check made on 17 November 2025). These are shown below along with the reasons given by that the review team for the reviews being different and/or proceeding.

- Role of CD4+ and CD8+ T Cell Counts and CD4/CD8 Ratio in Predicting Non-AIDS Events Following Successful ART: A Systematic Review and Meta-Analysis [published 12 November 2023] [CRD42023477945]. The review was judged **not to be similar**

- Changes of T lymphocyte subsets in hepatocellular carcinoma before and after locoregional treatments (LRTs)： A Meta-analysis [published 21 January 2022] [CRD42022298307]. The review was judged **not to be similar**
- Efficacy of Brucea javanica oil emulsion injection Combined Chemotherapy in Non-Small Cell Lung Cancer: A Systematic Review and Meta-Analysis [published 8 September 2022] [CRD42022356479]. The review was judged **not to be similar**
- Effect of Compound Kushen Injection on Immune Function in Patients with Colorectal Cancer: A Systematic Review and Meta-Analysis [published 16 January 2025] [CRD42025632516]. The review was judged **not to be similar**
- Effect of Chinese herbal medicine combined with enteral nutrition on critically ill patients: a systematic review and network meta-analysis [published 8 September 2024] [CRD42024580469]. The review was judged **not to be similar**
- The Deviations of CD4+ T Cells in Peripheral Blood and Peritoneal Fluid in Endometriosis: A systematic review and meta-analysis [published 21 December 2022] [CRD42022383153]. The review was judged **not to be similar**
- Efficacy and safety of different traditional Chinese medicine injections combined with radiotherapy and chemotherapy in the treatment of patients with intermediate and advanced pancreatic cancer: systematic reviews and network meta. [published 23 May 2024] [CRD42024545327]. The review was judged **not to be similar**
- T-cell subsets and interleukin-10 levels are predictors of severity and mortality in COVID-19: a systematic review and meta-analysis [published 6 November 2020] [CRD42020218918]. The review was judged **not to be similar**
- Treatment of Non-Small Cell Lung Cancer with Yiqi Buxue Prescriptions Combined with Adjuvant Chemotherapy on Cancer Therapy-Related Cardiovascular ToxicityA Systematic Review and Meta-Analysis [published 18 March 2025] [CRD420251013560]. The review was judged **not to be similar**
- Safety and Efficacy of Cytokine-Induced Killer Cell for Gastric Cancer: A Systematic Review and Meta-analysis [published 2 October 2025] [CRD420251160258]. The review was judged **not to be similar**
- The efficacy of combined use of Huaier granules in the treatment of primary liver cancer: An updated systematic review and meta-analysis [published 29 May 2025] [CRD420251063132]. The review was judged **not to be similar**
- Effects of Oral TCM Compounds Based on "Fuzheng Quxie" Strategy on Quality of Life and Immune parameters in Postoperative Breast Cancer Patients: A Systematic Review and Meta-Analysis [published 10 October 2025] [CRD420251165226]. The review was judged **not to be similar**
- The Effect of Sphingosine-1-phosphate Receptor Modulator Treatment on Leukocyte Subsets: A Systematic Review [published 15 January 2024] [CRD42024495528]. The review was judged **not to be similar**
- The prognostic role of CD8+ and CD4+ T cells in patients with esophageal cancer: a systematic review and meta-analysis [published 11 March 2020] [CRD42020151224]. The review was judged **not to be similar**
- Analysis of the Prognostic Value of the CD4/CD8+ T Cell Ratio in Patients with Cervical Cancer Undergoing Radiochemotherapy: A Meta-analysis [published 9 April 2025] [CRD420251022032]. The review was judged **not to be similar**

- The effects of an exercise program on the CD4+ and CD8+ T lymphocyte response in people living with HIV (PLHIV): Systematic Review [published 18 July 2021] [CRD42021261512]. The review was judged **not to be similar**
- Effects of TCM Based on "Fuzheng Quxie" Strategy on Quality of Life and Immune parameters combined with chemotherapy for NSCLC patients: A Systematic Review and Network meta-analysis [published 25 October 2025] [CRD420251175273]. The review was judged **not to be similar**
- The role of T cells in cervical progression [published 21 November 2023] [CRD42023481919]. The review was judged **not to be similar**
- The Effects of Acupoint Stimulation on T Lymphocyte Subgroups and NK Cell in Peripheral Blood of Patients with Malignant Tumor [published 28 April 2020] [CRD42020167842]. The review was judged **not to be similar**
- Systematic evaluation of the effects of propofol on T cell function and clarification of its immune regulatory role. [published 23 October 2025] [CRD420251167548]. The review was judged **not to be similar**

PROSPERO version history

- Version 1.0, published 20 Nov 2025

Disclaimer

The content of this record displays the information provided by the review team. PROSPERO does not peer review registration records or endorse their content.

PROSPERO accepts and posts the information provided in good faith; responsibility for record content rests with the review team. The guarantor for this record has affirmed that the information provided is truthful and that they understand that deliberate provision of inaccurate information may be construed as scientific misconduct.

PROSPERO does not accept any liability for the content provided in this record or for its use. Readers use the information provided in this record at their own risk.

Any enquiries about the record should be referred to the named review contact
