# Supplementary material for: What are the beneficial treatment strategies in maintaining T lymphocyte subsets after cancer surgery? A systematic review and network meta-analysis
Source: Front Immunol. 2026 Jul 14;17:1854279. doi: 10.3389/fimmu.2026.1854279 (PMC13408238; doi:10.3389/fimmu.2026.1854279)

Figure S9. Forest plot of anesthesia intervention measures in the time subgroup.
Sevoflurane
9.1CD3≤1day，CD3＞1day，≤1week

Study or Subgroup	Experimental Mean	Experimental SD	Experimental Total	Control Mean	Control SD	Control Total	Weight	Mean Difference IV, Fixed, 95% CI
**2.1.1 CD3≤1day**								
Liu, S.2016 1d	-0.82	0.74	29	-0.4	0.66	29	27.2%	-0.42 [-0.78, -0.06]
Liu, S.2016 after operation	-0.28	0.74	29	-0.07	0.68	29	26.5%	-0.21 [-0.58, 0.16]
Zhang, T.2014 1d	-10.1	13.59	18	-9.7	11.74	19	0.1%	-0.40 [-8.60, 7.80]
Zhang, T.2014 3h	-13.8	13.66	18	-11	11.38	19	0.1%	-2.80 [-10.92, 5.32]
Zhang, T.2014 5h	-16.6	12.76	18	-9.8	11.26	19	0.1%	-6.80 [-14.57, 0.97]
Zhang, T.2014 after operation	-13.4	12.46	18	-1.9	11.44	19	0.1%	-11.50 [-19.22, -3.78]
Zhang, T.2014 anesthesia1h	-1.6	14	18	1.2	13.15	19	0.0%	-2.80 [-11.56, 5.96]
**Subtotal (95% CI)**			**148**			**153**	**54.0%**	**-0.34 [-0.60, -0.08]**
Heterogeneity: Chi² = 12.01, df = 6 (P = 0.06); I² = 50%								
Test for overall effect: Z = 2.60 (P = 0.009)								
**2.1.2 CD3＞1day，≤1week**								
Liu, S.2016 2d	-0.89	0.74	29	-0.45	0.91	29	19.4%	-0.44 [-0.87, -0.01]
Liu, S.2016 3d	-0.48	0.74	29	-0.15	0.68	29	26.5%	-0.33 [-0.70, 0.04]
Zhang, T.2014 2d	-15.2	12.4	18	-10.6	12	19	0.1%	-4.60 [-12.47, 3.27]
Zhang, T.2014 3d	-15.8	12.23	18	-10.9	12.39	19	0.1%	-4.90 [-12.83, 3.03]
**Subtotal (95% CI)**			**94**			**96**	**46.0%**	**-0.39 [-0.66, -0.11]**
Heterogeneity: Chi² = 2.50, df = 3 (P = 0.48); I² = 0%								
Test for overall effect: Z = 2.74 (P = 0.006)								
**Total (95% CI)**			**242**			**249**	**100.0%**	**-0.36 [-0.55, -0.17]**
Heterogeneity: Chi² = 14.57, df = 10 (P = 0.15); I² = 31%								
Test for overall effect: Z = 3.77 (P = 0.0002)								
Test for subgroup differences: Chi² = 0.06, df = 1 (P = 0.81), I² = 0%								

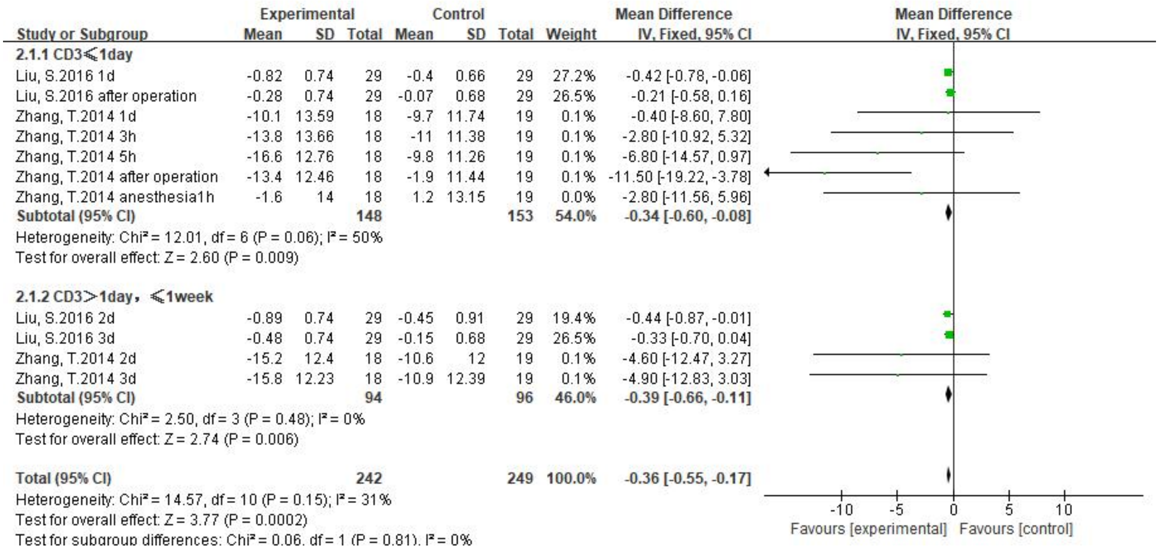

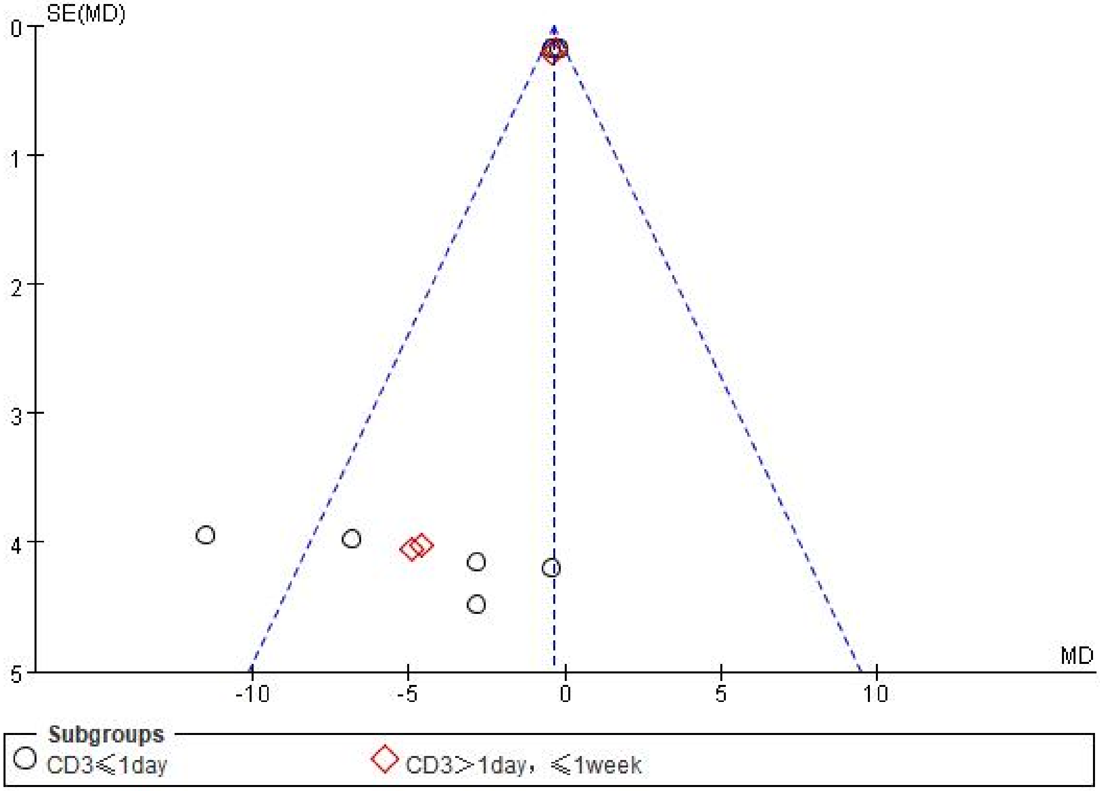

9.2CD4≤1day，CD4＞1day，≤1week

Study or Subgroup	Experimental Mean	Experimental SD	Experimental Total	Control Mean	Control SD	Control Total	Weight	Mean Difference IV, Random, 95% CI
**2.2.1 CD4≤1day**								
Liu, S.2016 1d	-0.4	0.45	29	-0.23	0.42	29	24.8%	-0.17 [-0.39, 0.05]
Liu, S.2016 after operation	-0.23	0.45	29	-0.09	0.44	29	24.6%	-0.14 [-0.37, 0.09]
Zhang, T.2014 1d	-11.6	12.17	18	-6.7	12.36	19	0.1%	-4.90 [-12.81, 3.01]
Zhang, T.2014 3h	-10.4	11.45	18	-5.3	12.55	19	0.1%	-5.10 [-12.83, 2.63]
Zhang, T.2014 5h	-10.7	11.26	18	-5.9	12.29	19	0.1%	-4.80 [-12.39, 2.79]
Zhang, T.2014 after operation	-10.6	12.03	18	2.3	13.51	19	0.1%	-12.90 [-21.13, -4.67]
Zhang, T.2014 anesthesia1h	-1.9	12.59	18	1.1	12.68	19	0.1%	-3.00 [-11.14, 5.14]
**Subtotal (95% CI)**			**148**			**153**	**49.9%**	**-0.26 [-0.74, 0.22]**
Heterogeneity: Tau² = 0.11; Chi² = 14.08, df = 6 (P = 0.03); I² = 57%								
Test for overall effect: Z = 1.07 (P = 0.28)								
**2.2.2 CD4＞1day，≤1week**								
Liu, S.2016 2d	-0.44	0.42	29	-0.24	0.43	29	25.0%	-0.20 [-0.42, 0.02]
Liu, S.2016 3d	-0.38	0.44	29	-0.26	0.42	29	24.9%	-0.12 [-0.34, 0.10]
Zhang, T.2014 2d	-15.6	12.8	18	-5.9	11.68	19	0.1%	-9.70 [-17.61, -1.79]
Zhang, T.2014 3d	-15.1	12.17	18	-6.3	13.22	19	0.1%	-8.80 [-16.98, -0.62]
**Subtotal (95% CI)**			**94**			**96**	**50.1%**	**-0.21 [-0.66, 0.23]**
Heterogeneity: Tau² = 0.09; Chi² = 10.12, df = 3 (P = 0.02); I² = 70%								
Test for overall effect: Z = 0.95 (P = 0.34)								
**Total (95% CI)**			**242**			**249**	**100.0%**	**-0.21 [-0.48, 0.06]**
Heterogeneity: Tau² = 0.06; Chi² = 24.20, df = 10 (P = 0.007); I² = 59%								
Test for overall effect: Z = 1.55 (P = 0.12)								
Test for subgroup differences: Chi² = 0.02, df = 1 (P = 0.89), I² = 0%								

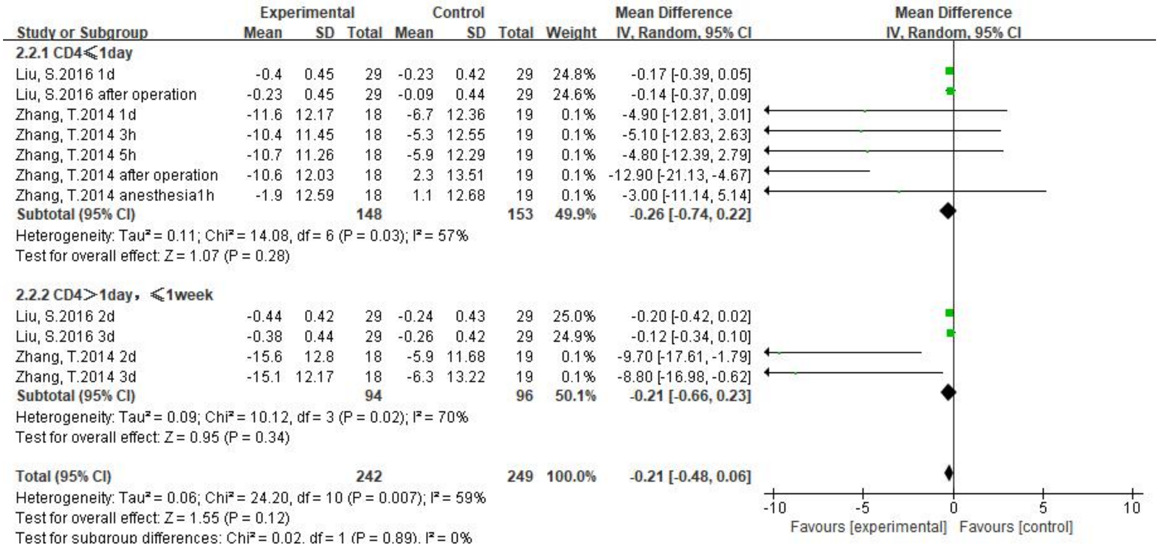

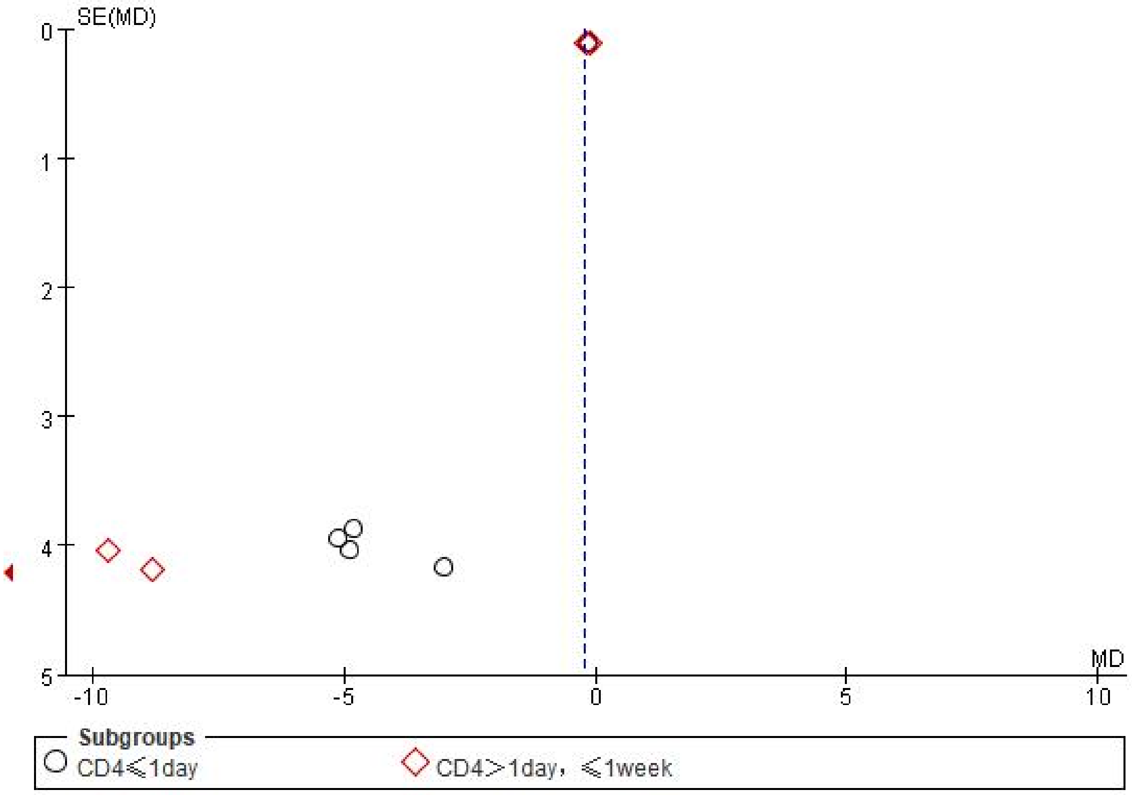

9.3CD8≤1day，CD8＞1day，≤1week

Study or Subgroup	Experimental Mean	Experimental SD	Experimental Total	Control Mean	Control SD	Control Total	Weight	Mean Difference IV, Fixed, 95% CI
**2.3.1 CD8≤1day**								
Liu, S.2016 1d	0.08	0.39	29	0.15	0.42	29	25.5%	-0.07 [-0.28, 0.14]
Liu, S.2016 after operation	0.15	0.42	29	0.06	0.42	29	23.8%	0.09 [-0.13, 0.31]
Zhang, T.2014 1d	1.1	14.21	18	-2.9	11.05	19	0.0%	4.00 [-4.23, 12.23]
Zhang, T.2014 3h	1.7	12.7	18	2	12.39	19	0.0%	-0.30 [-8.39, 7.79]
Zhang, T.2014 5h	2.1	12.35	18	-0.3	11.74	19	0.0%	2.40 [-5.37, 10.17]
Zhang, T.2014 after operation	-2	13.8	18	-0.4	12.17	19	0.0%	-1.60 [-10.00, 6.80]
Zhang, T.2014 anesthesia1h	-0.8	13.66	18	-0.8	12.84	19	0.0%	0.00 [-8.55, 8.55]
**Subtotal (95% CI)**			**148**			**153**	**49.4%**	**0.01 [-0.14, 0.16]**
Heterogeneity: Chi² = 2.50, df = 6 (P = 0.87); I² = 0%								
Test for overall effect: Z = 0.11 (P = 0.91)								
**2.3.2 CD8＞1day，≤1week**								
Liu, S.2016 2d	0.04	0.43	29	-0.06	0.4	29	24.3%	0.10 [-0.11, 0.31]
Liu, S.2016 3d	0.04	0.4	29	0.1	0.4	29	26.2%	-0.06 [-0.27, 0.15]
Zhang, T.2014 2d	0.2	12.88	18	0.1	12.17	19	0.0%	0.10 [-7.98, 8.18]
Zhang, T.2014 3d	2	13.8	18	1.1	11.39	19	0.0%	0.90 [-7.28, 9.08]
**Subtotal (95% CI)**			**94**			**96**	**50.6%**	**0.02 [-0.13, 0.17]**
Heterogeneity: Chi² = 1.16, df = 3 (P = 0.76); I² = 0%								
Test for overall effect: Z = 0.23 (P = 0.82)								
**Total (95% CI)**			**242**			**249**	**100.0%**	**0.01 [-0.09, 0.12]**
Heterogeneity: Chi² = 3.67, df = 10 (P = 0.96); I² = 0%								
Test for overall effect: Z = 0.24 (P = 0.81)								
Test for subgroup differences: Chi² = 0.01, df = 1 (P = 0.94), I² = 0%								

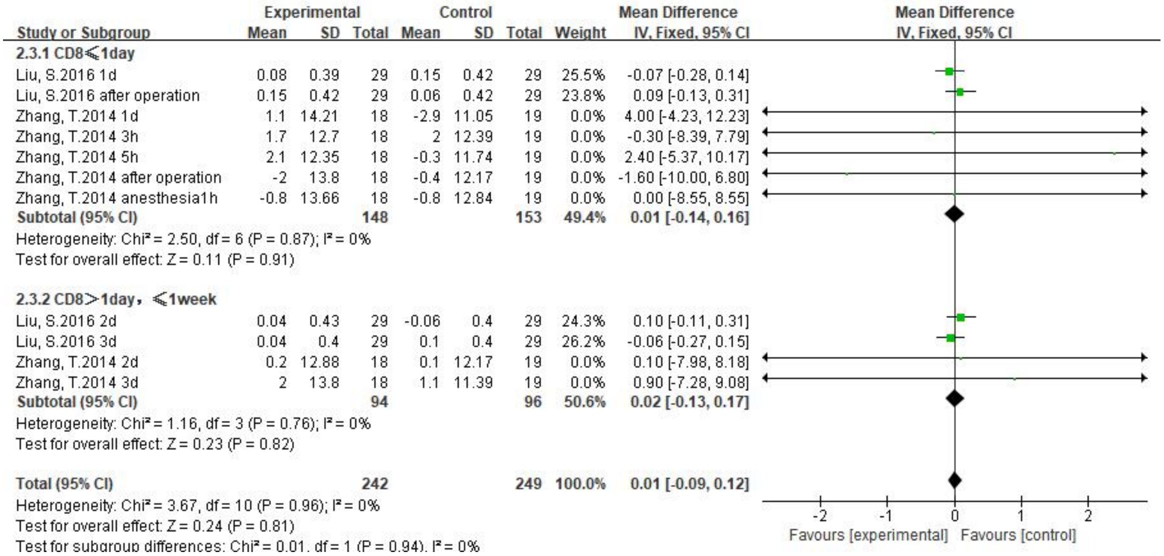

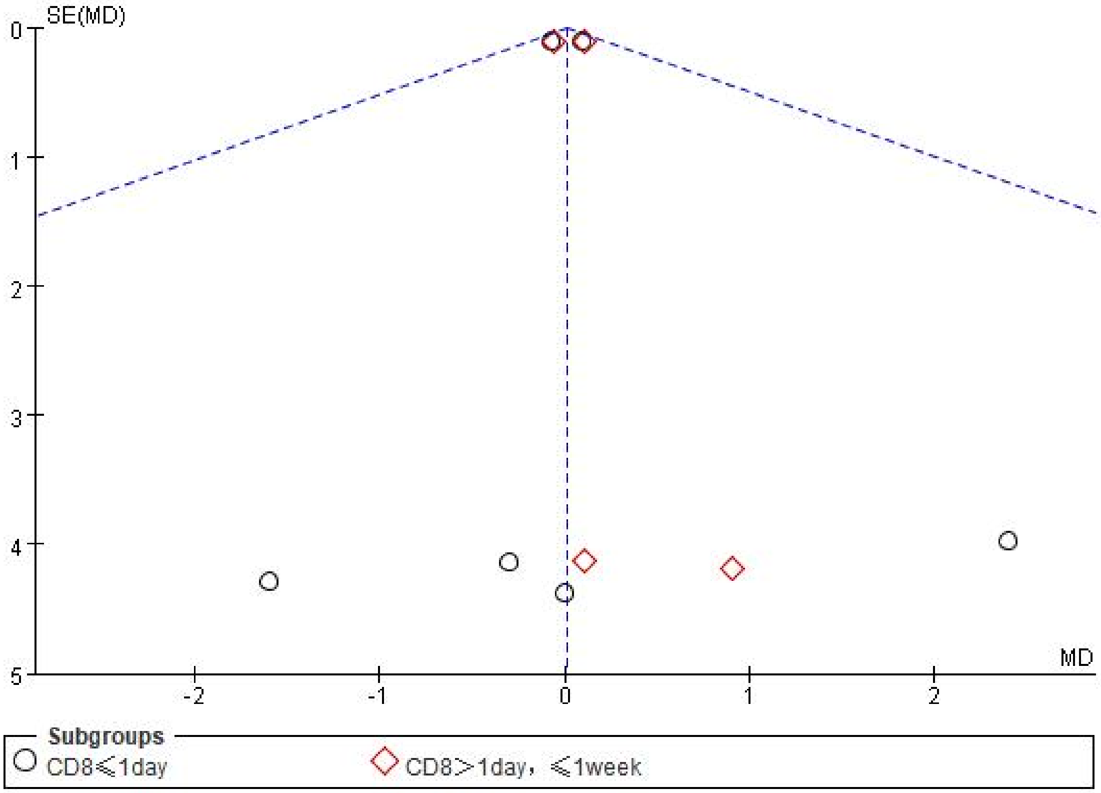

9.4CD4/CD8≤1day，CD4/CD8＞1day，≤1week

Study or Subgroup	Experimental Mean	Experimental SD	Experimental Total	Control Mean	Control SD	Control Total	Weight	Mean Difference IV, Fixed, 95% CI
**2.4.1 CD4/CD8≤1day**								
Liu, S.2016 1d	-0.54	0.91	29	-0.39	0.98	29	11.6%	-0.15 [-0.64, 0.34]
Liu, S.2016 after operation	-0.19	0.95	29	-0.06	0.9	29	12.1%	-0.13 [-0.61, 0.35]
Zhang, T.2014 1d	-0.6	0.86	18	-0.5	0.94	19	8.2%	-0.10 [-0.68, 0.48]
Zhang, T.2014 3h	-0.6	0.99	18	-0.5	1.13	19	5.9%	-0.10 [-0.78, 0.58]
Zhang, T.2014 5h	-0.6	1.06	18	-0.5	1.3	19	4.7%	-0.10 [-0.86, 0.66]
Zhang, T.2014 after operation	-0.5	1.06	18	-0.1	1.28	19	4.8%	-0.40 [-1.16, 0.36]
Zhang, T.2014 anesthesia1h	-0.1	1.14	18	-0.1	1.13	19	5.1%	0.00 [-0.73, 0.73]
**Subtotal (95% CI)**			**148**			**153**	**52.6%**	**-0.14 [-0.36, 0.09]**
Heterogeneity: Chi² = 0.64, df = 6 (P = 1.00); I² = 0%								
Test for overall effect: Z = 1.16 (P = 0.25)								
**2.4.2 CD4/CD8＞1day，≤1week**								
Liu, S.2016 2d	-0.79	0.7	29	-0.58	0.87	29	16.7%	-0.21 [-0.62, 0.20]
Liu, S.2016 3d	-0.82	0.7	29	-0.57	0.95	29	14.9%	-0.25 [-0.68, 0.18]
Zhang, T.2014 2d	-0.7	0.86	18	-0.5	1	19	7.7%	-0.20 [-0.80, 0.40]
Zhang, T.2014 3d	-0.8	0.86	18	-0.4	0.94	19	8.2%	-0.40 [-0.98, 0.18]
**Subtotal (95% CI)**			**94**			**96**	**47.4%**	**-0.25 [-0.49, -0.01]**
Heterogeneity: Chi² = 0.32, df = 3 (P = 0.96); I² = 0%								
Test for overall effect: Z = 2.06 (P = 0.04)								
**Total (95% CI)**			**242**			**249**	**100.0%**	**-0.19 [-0.36, -0.03]**
Heterogeneity: Chi² = 1.44, df = 10 (P = 1.00); I² = 0%								
Test for overall effect: Z = 2.26 (P = 0.02)								
Test for subgroup differences: Chi² = 0.48, df = 1 (P = 0.49), I² = 0%								

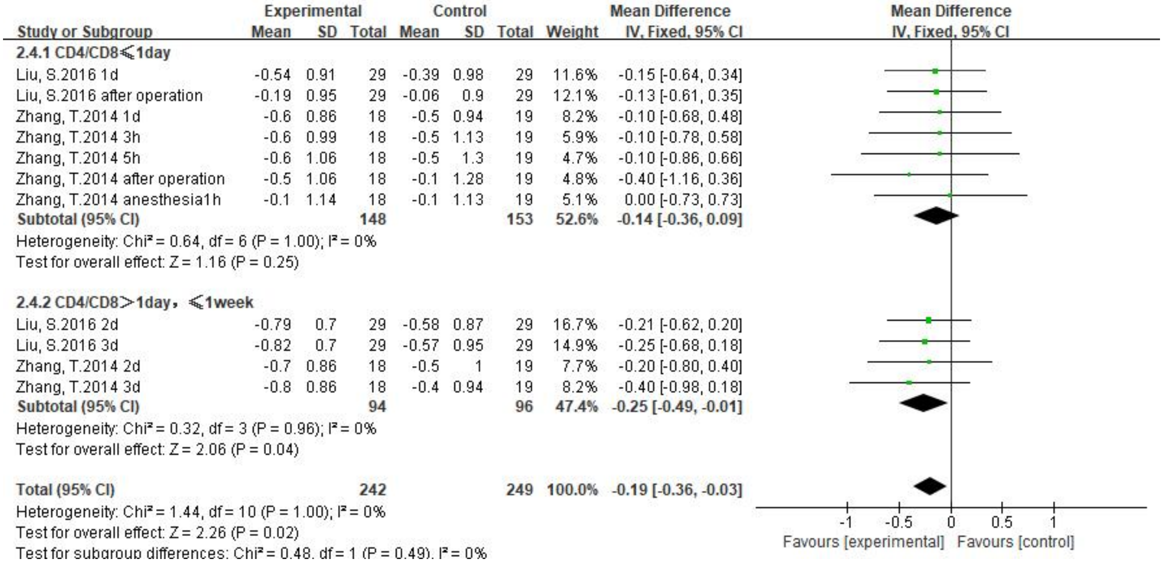

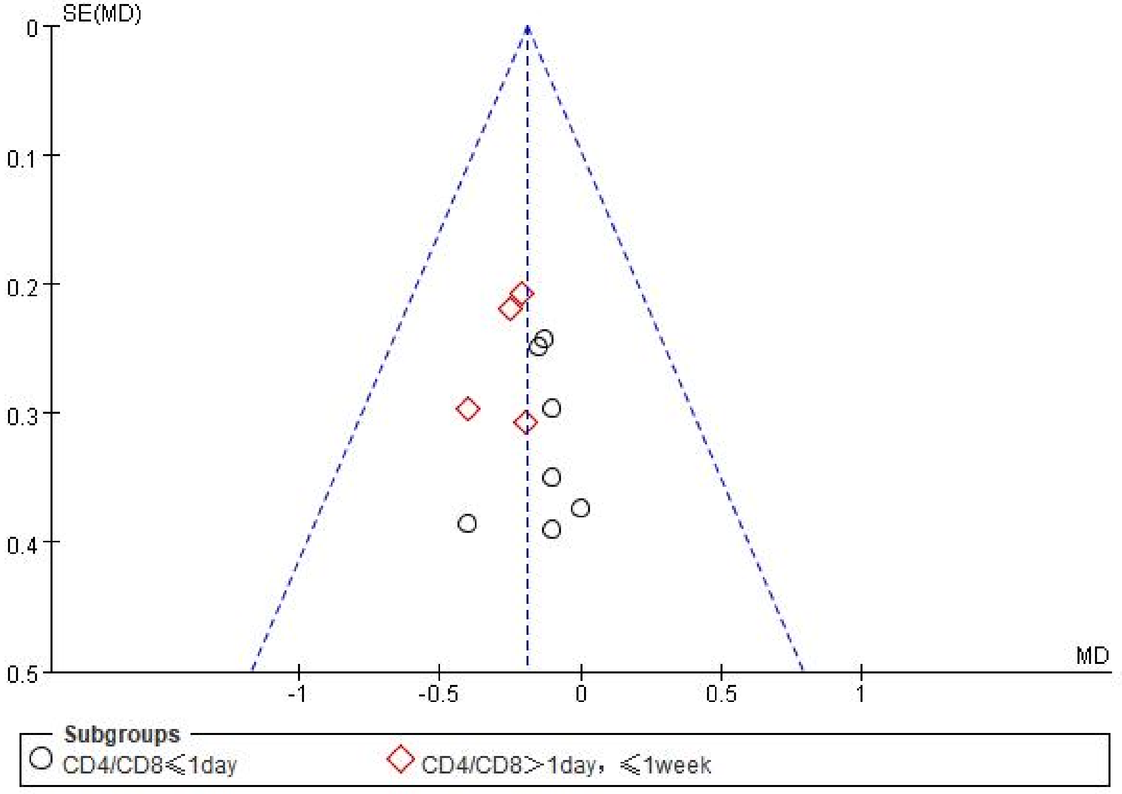

Propofol

9.5CD3≤1day，CD3＞1day，≤1week

Study or Subgroup	Experimental Mean	Experimental SD	Experimental Total	Control Mean	Control SD	Control Total	Weight	Mean Difference IV, Fixed, 95% CI
**2.1.1 CD3⩽1day**								
Liu, S.2016 1d	-0.4	0.66	29	-0.82	0.74	29	27.2%	0.42 [0.06, 0.78]
Liu, S.2016 after operation	-0.07	0.68	29	-0.28	0.74	29	26.5%	0.21 [-0.16, 0.58]
Zhang, T.2014 after operation	-9.7	11.74	19	-10.1	13.59	18	0.1%	0.40 [-7.80, 8.60]
Zhang, T.2014 1d	-10.6	12	19	-15.2	12.4	18	0.1%	4.60 [-3.27, 12.47]
Zhang, T.2014 3h	-9.8	11.26	19	-16.6	12.76	18	0.1%	6.80 [-0.97, 14.57]
Zhang, T.2014 5h	-1.9	11.44	19	-13.4	12.46	18	0.1%	11.50 [3.78, 19.22]
Zhang, T.2014 anesthesia1h	1.2	13.15	19	-1.6	14	18	0.0%	2.80 [-5.96, 11.56]
**Subtotal (95% CI)**			**153**			**148**	**54.0%**	**0.34 [0.09, 0.60]**
Heterogeneity: Chi² = 12.79, df = 6 (P = 0.05); I² = 53%								
Test for overall effect: Z = 2.62 (P = 0.009)								
**2.1.2 CD3>1day，⩽1week**								
Liu, S.2016 2d	-0.45	0.91	29	-0.89	0.74	29	19.4%	0.44 [0.01, 0.87]
Liu, S.2016 3d	-0.15	0.68	29	-0.48	0.74	29	26.5%	0.33 [-0.04, 0.70]
Zhang, T.2014 2d	-10.9	12.39	19	-15.8	12.23	18	0.1%	4.90 [-3.03, 12.83]
Zhang, T.2014 3d	-11	11.38	19	-13.8	13.66	18	0.1%	2.80 [-5.32, 10.92]
**Subtotal (95% CI)**			**96**			**94**	**46.0%**	**0.38 [0.11, 0.66]**
Heterogeneity: Chi² = 1.73, df = 3 (P = 0.63); I² = 0%								
Test for overall effect: Z = 2.72 (P = 0.007)								
**Total (95% CI)**			**249**			**242**	**100.0%**	**0.36 [0.17, 0.55]**
Heterogeneity: Chi² = 14.57, df = 10 (P = 0.15); I² = 31%								
Test for overall effect: Z = 3.77 (P = 0.0002)								
Test for subgroup differences: Chi² = 0.05, df = 1 (P = 0.83), I² = 0%								

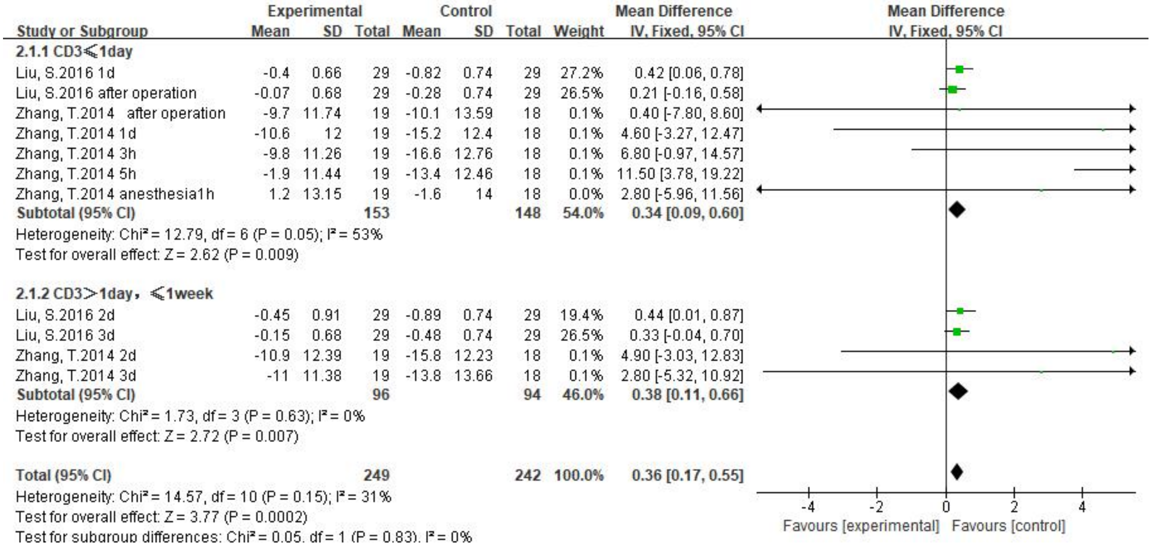

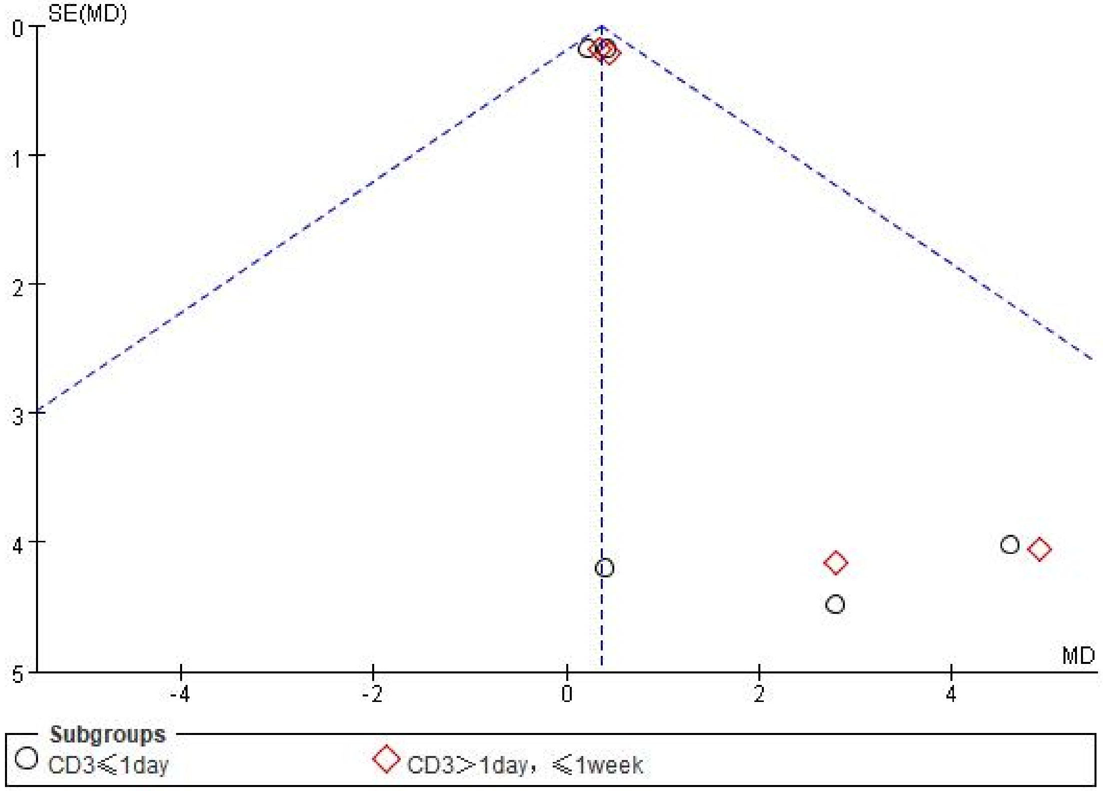

9.6CD4≤1day，CD4＞1day，≤1week

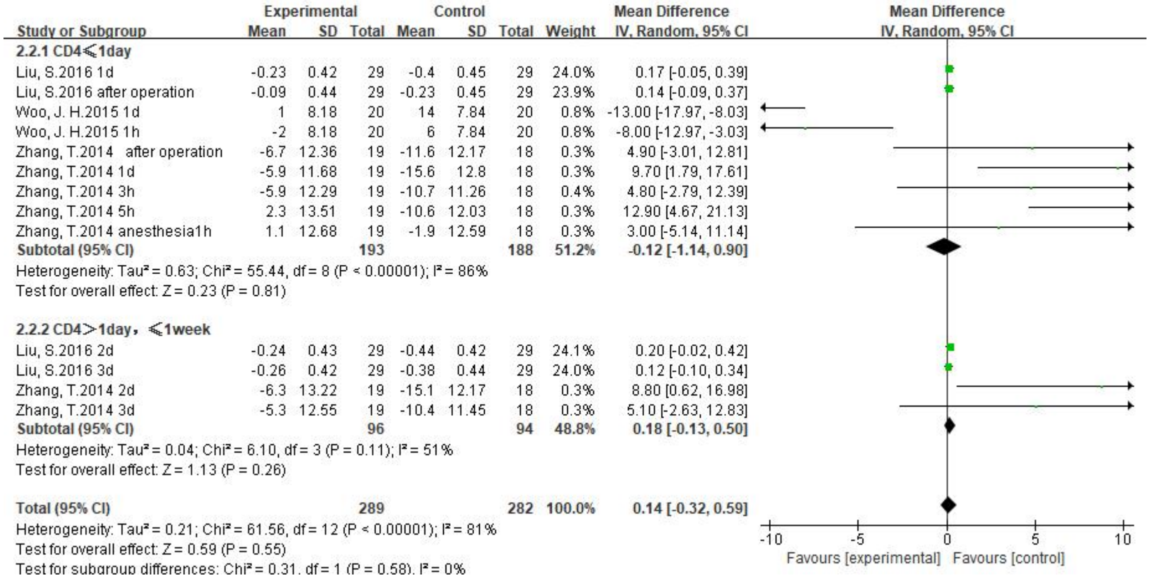

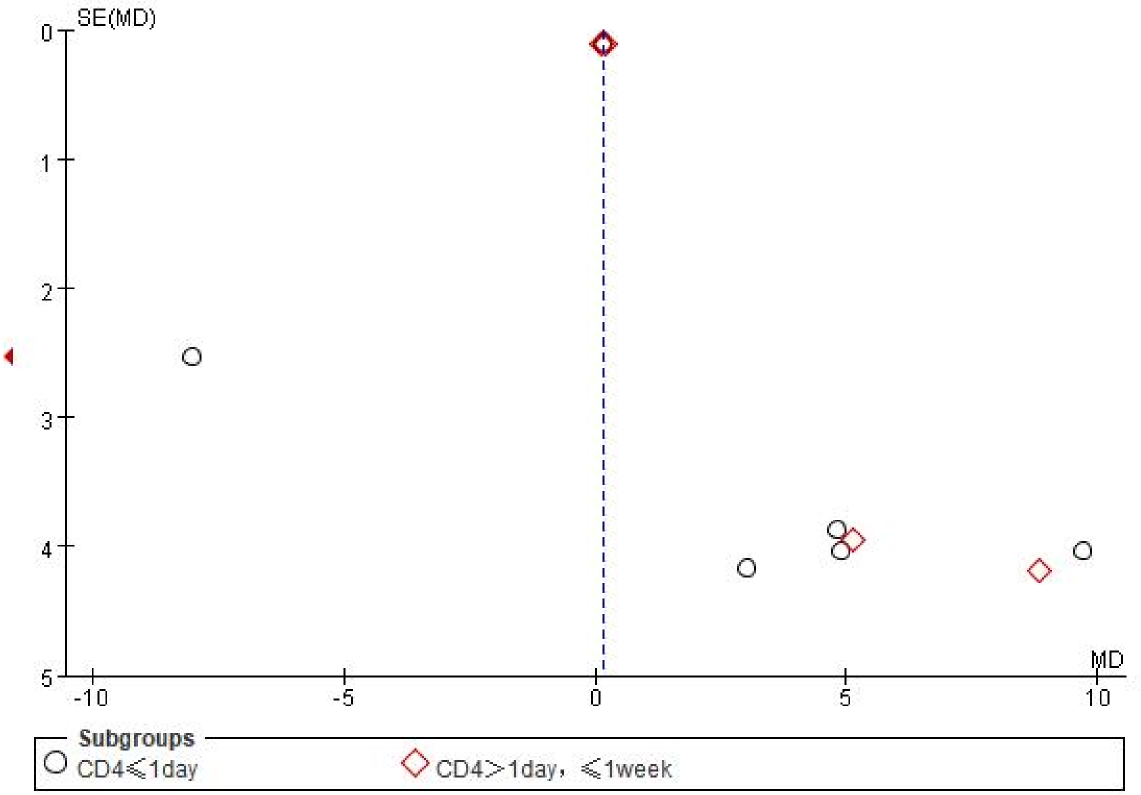

9.7CD8≤1day，CD8＞1day，≤1week

Study or Subgroup	Experimental Mean	Experimental SD	Experimental Total	Control Mean	Control SD	Control Total	Weight	Mean Difference IV, Fixed, 95% CI
**2.3.1 CD8⩽1day**								
Liu, S.2016 1d	0.15	0.42	29	0.08	0.39	29	25.5%	0.07 [-0.14, 0.28]
Liu, S.2016 after operation	0.06	0.42	29	0.15	0.42	29	23.8%	-0.09 [-0.31, 0.13]
Woo, J. H.2015 1d	-7	7.76	20	3	8.64	20	0.0%	-10.00 [-15.09, -4.91]
Woo, J. H.2015 1h	-7	7.76	20	-7	8.64	20	0.0%	0.00 [-5.09, 5.09]
Zhang, T.2014 after operation	-2.9	11.05	19	1.1	14.21	18	0.0%	-4.00 [-12.23, 4.23]
Zhang, T.2014 1d	0.1	12.17	19	0.2	12.88	18	0.0%	-0.10 [-8.18, 7.98]
Zhang, T.2014 3h	-0.3	11.74	19	2.1	12.35	18	0.0%	-2.40 [-10.17, 5.37]
Zhang, T.2014 5h	-0.4	12.17	19	-2	13.8	18	0.0%	1.60 [-6.80, 10.00]
Zhang, T.2014 anesthesia1h	-0.8	12.84	19	-0.8	13.66	18	0.0%	0.00 [-8.55, 8.55]
**Subtotal (95% CI)**			**193**			**188**	**49.5%**	**-0.02 [-0.17, 0.13]**
Heterogeneity: Chi² = 17.29, df = 8 (P = 0.03); I² = 54%								
Test for overall effect: Z = 0.23 (P = 0.82)								
**2.3.2 CD8＞1day，⩽1week**								
Liu, S.2016 2d	-0.06	0.4	29	0.04	0.43	29	24.3%	-0.10 [-0.31, 0.11]
Liu, S.2016 3d	0.1	0.4	29	0.04	0.4	29	26.2%	0.06 [-0.15, 0.27]
Zhang, T.2014 2d	1.1	11.39	19	2	13.8	18	0.0%	-0.90 [-9.08, 7.28]
Zhang, T.2014 3d	2	12.39	19	1.7	12.7	18	0.0%	0.30 [-7.79, 8.39]
**Subtotal (95% CI)**			**96**			**94**	**50.5%**	**-0.02 [-0.17, 0.13]**
Heterogeneity: Chi² = 1.17, df = 3 (P = 0.76); I² = 0%								
Test for overall effect: Z = 0.23 (P = 0.82)								
**Total (95% CI)**			**289**			**282**	**100.0%**	**-0.02 [-0.12, 0.09]**
Heterogeneity: Chi² = 18.46, df = 12 (P = 0.10); I² = 35%								
Test for overall effect: Z = 0.32 (P = 0.75)								
Test for subgroup differences: Chi² = 0.00, df = 1 (P = 1.00), I² = 0%								

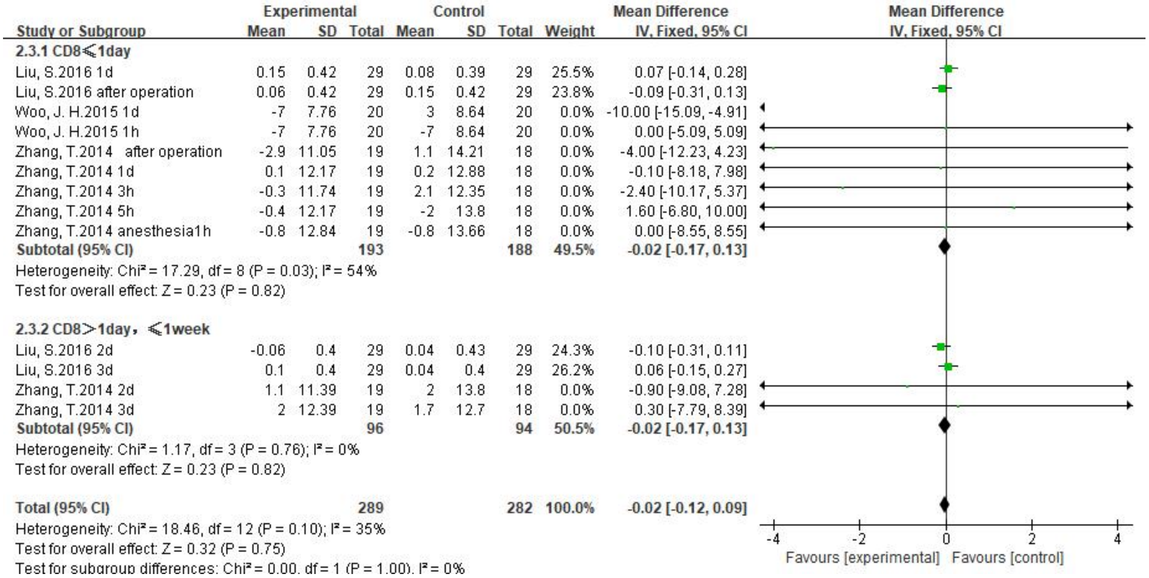

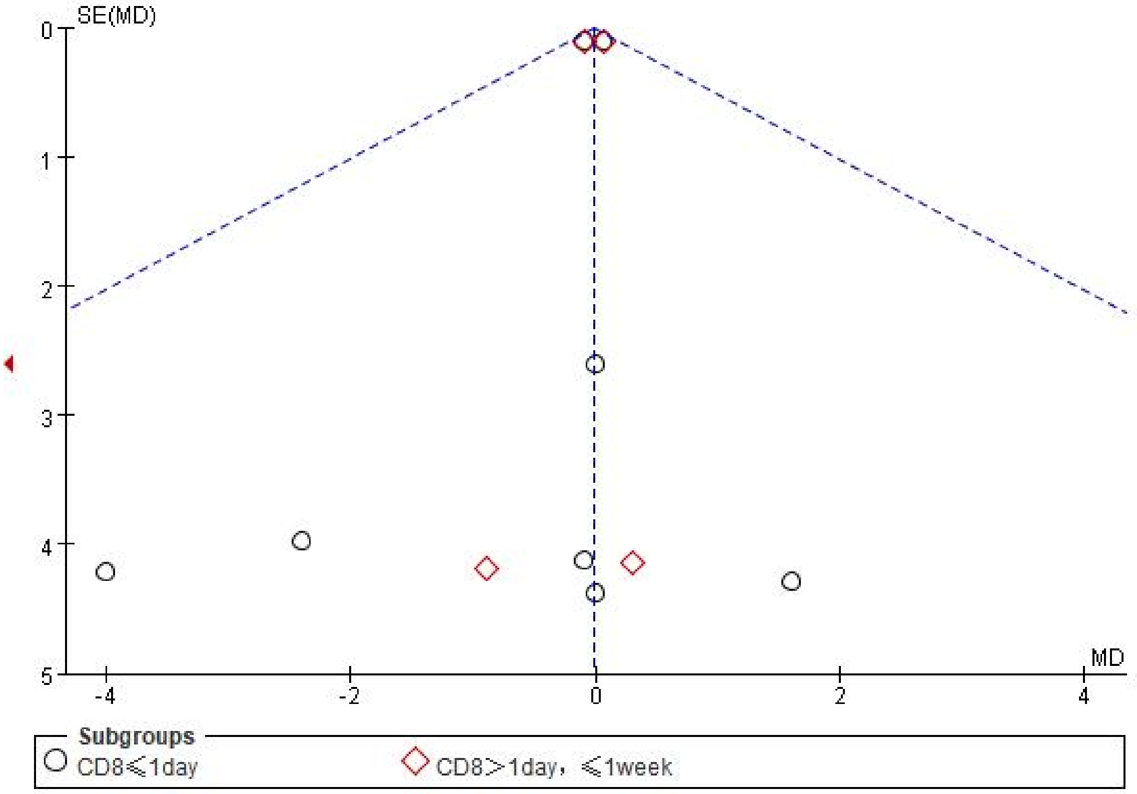

9.8CD4/CD8≤1day，CD4/CD8＞1day，≤1week

Study or Subgroup	Experimental Mean	Experimental SD	Experimental Total	Control Mean	Control SD	Control Total	Weight	Mean Difference IV, Fixed, 95% CI
**2.4.1 CD4/CD8≤1day**								
Liu, S.2016 1d	-0.39	0.98	29	-0.54	0.91	29	10.1%	0.15 [-0.34, 0.64]
Liu, S.2016 after operation	-0.06	0.9	29	-0.19	0.95	29	10.6%	0.13 [-0.35, 0.61]
Woo, J. H.2015 1d	0.33	1.04	20	0.04	0.92	20	6.5%	0.29 [-0.32, 0.90]
Woo, J. H.2015 1h	0.62	1.04	20	0.42	0.92	20	6.5%	0.20 [-0.41, 0.81]
Zhang, T.2014 after operation	-0.5	0.94	19	-0.6	0.86	18	7.1%	0.10 [-0.48, 0.68]
Zhang, T.2014 1d	-0.5	1	19	-0.7	0.86	18	6.7%	0.20 [-0.40, 0.80]
Zhang, T.2014 3h	-0.5	1.3	19	-0.6	1.06	18	4.1%	0.10 [-0.66, 0.86]
Zhang, T.2014 5h	-0.1	1.28	19	-0.5	1.06	18	4.2%	0.40 [-0.36, 1.16]
Zhang, T.2014 anesthesia1h	-0.1	1.13	19	-0.1	1.14	18	4.5%	0.00 [-0.73, 0.73]
**Subtotal (95% CI)**			**193**			**188**	**60.2%**	**0.17 [-0.03, 0.37]**
Heterogeneity: Chi² = 0.85, df = 8 (P = 1.00); I² = 0%								
Test for overall effect: Z = 1.66 (P = 0.10)								
**2.4.2 CD4/CD8＞1day，≤1week**								
Liu, S.2016 2d	-0.58	0.87	29	-0.79	0.7	29	14.5%	0.21 [-0.20, 0.62]
Liu, S.2016 3d	-0.57	0.95	29	-0.82	0.7	29	13.0%	0.25 [-0.18, 0.68]
Zhang, T.2014 2d	-0.4	0.94	19	-0.8	0.86	18	7.1%	0.40 [-0.18, 0.98]
Zhang, T.2014 3d	-0.5	1.13	19	-0.6	0.99	18	5.1%	0.10 [-0.58, 0.78]
**Subtotal (95% CI)**			**96**			**94**	**39.8%**	**0.24 [-0.00, 0.49]**
Heterogeneity: Chi² = 0.48, df = 3 (P = 0.92); I² = 0%								
Test for overall effect: Z = 1.94 (P = 0.05)								
**Total (95% CI)**			**289**			**282**	**100.0%**	**0.20 [0.04, 0.35]**
Heterogeneity: Chi² = 1.54, df = 12 (P = 1.00); I² = 0%								
Test for overall effect: Z = 2.51 (P = 0.01)								
Test for subgroup differences: Chi² = 0.21, df = 1 (P = 0.65), I² = 0%								

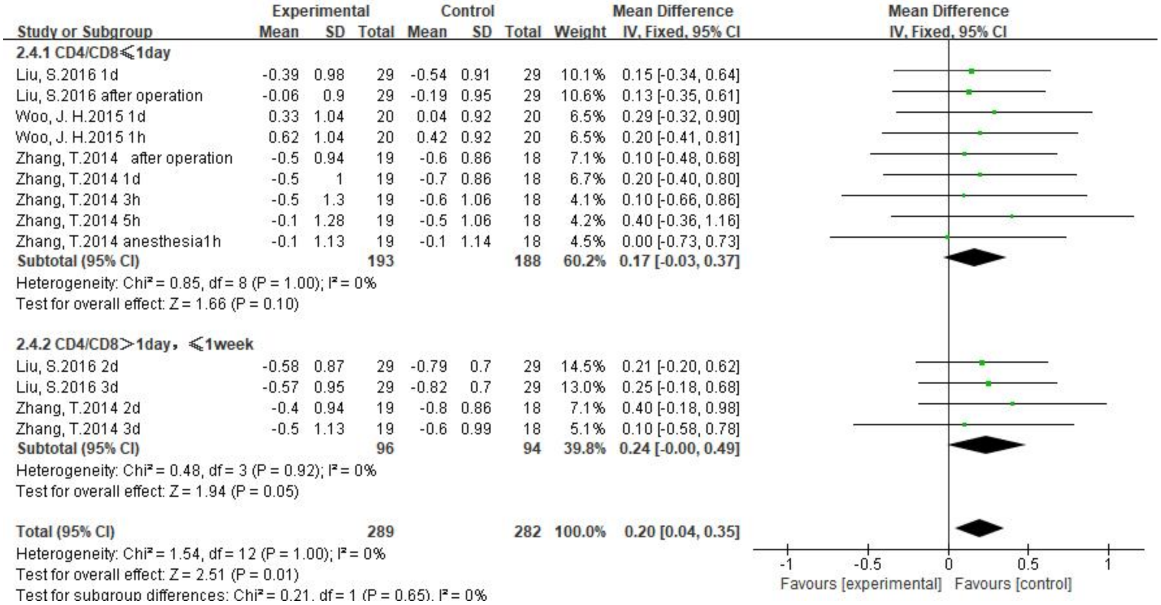

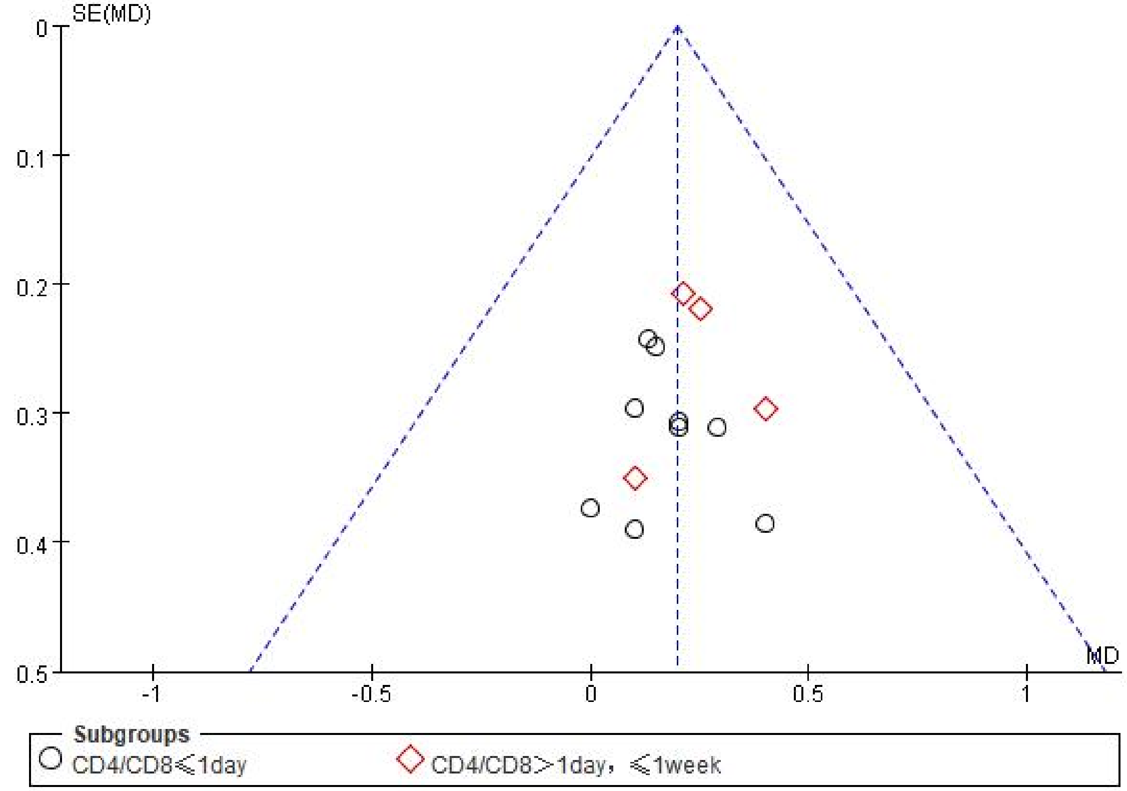

Flurbiprofen

9.9CD3≤1day，CD3＞1day，≤1week

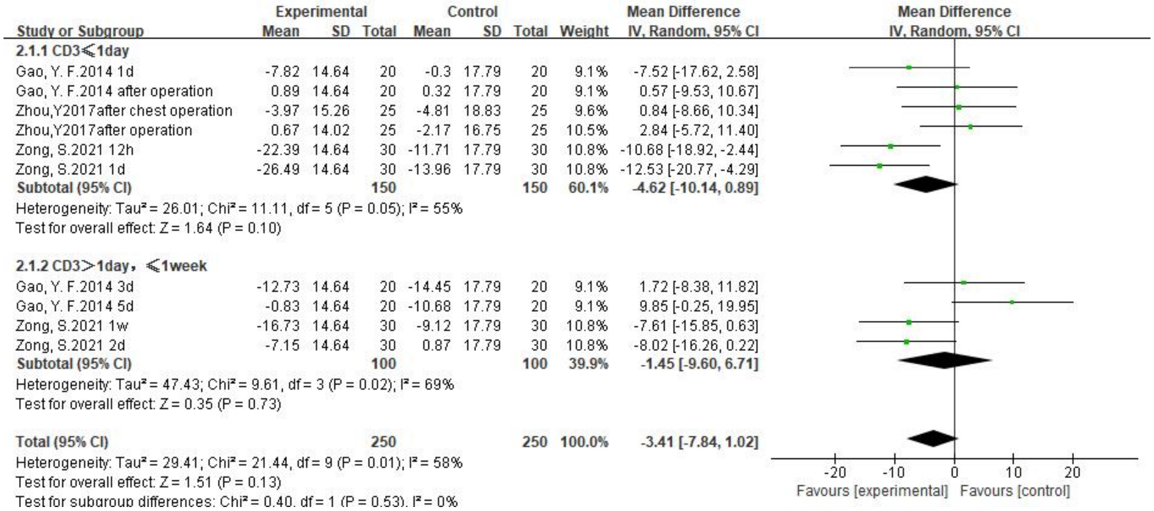

Study or Subgroup	Experimental Mean	Experimental SD	Experimental Total	Control Mean	Control SD	Control Total	Weight	Mean Difference IV, Random, 95% CI
**2.1.1 CD3≤1day**								
Gao, Y. F.2014 1d	-7.82	14.64	20	-0.3	17.79	20	9.1%	-7.52 [-17.62, 2.58]
Gao, Y. F.2014 after operation	0.89	14.64	20	0.32	17.79	20	9.1%	0.57 [-9.53, 10.67]
Zhou,Y2017after chest operation	-3.97	15.26	25	-4.81	18.83	25	9.6%	0.84 [-8.66, 10.34]
Zhou,Y2017after operation	0.67	14.02	25	-2.17	16.75	25	10.5%	2.84 [-5.72, 11.40]
Zong, S.2021 12h	-22.39	14.64	30	-11.71	17.79	30	10.8%	-10.68 [-18.92, -2.44]
Zong, S.2021 1d	-26.49	14.64	30	-13.96	17.79	30	10.8%	-12.53 [-20.77, -4.29]
**Subtotal (95% CI)**			**150**			**150**	**60.1%**	**-4.62 [-10.14, 0.89]**
Heterogeneity: Tau² = 26.01; Chi² = 11.11, df = 5 (P = 0.05); I² = 55%								
Test for overall effect: Z = 1.64 (P = 0.10)								
**2.1.2 CD3>1day，≤1week**								
Gao, Y. F.2014 3d	-12.73	14.64	20	-14.45	17.79	20	9.1%	1.72 [-8.38, 11.82]
Gao, Y. F.2014 5d	-0.83	14.64	20	-10.68	17.79	20	9.1%	9.85 [-0.25, 19.95]
Zong, S.2021 1w	-16.73	14.64	30	-9.12	17.79	30	10.8%	-7.61 [-15.85, 0.63]
Zong, S.2021 2d	-7.15	14.64	30	0.87	17.79	30	10.8%	-8.02 [-16.26, 0.22]
**Subtotal (95% CI)**			**100**			**100**	**39.9%**	**-1.45 [-9.60, 6.71]**
Heterogeneity: Tau² = 47.43; Chi² = 9.61, df = 3 (P = 0.02); I² = 69%								
Test for overall effect: Z = 0.35 (P = 0.73)								
**Total (95% CI)**			**250**			**250**	**100.0%**	**-3.41 [-7.84, 1.02]**
Heterogeneity: Tau² = 29.41; Chi² = 21.44, df = 9 (P = 0.01); I² = 58%								
Test for overall effect: Z = 1.51 (P = 0.13)								
Test for subgroup differences: Chi² = 0.40, df = 1 (P = 0.53), I² = 0%								

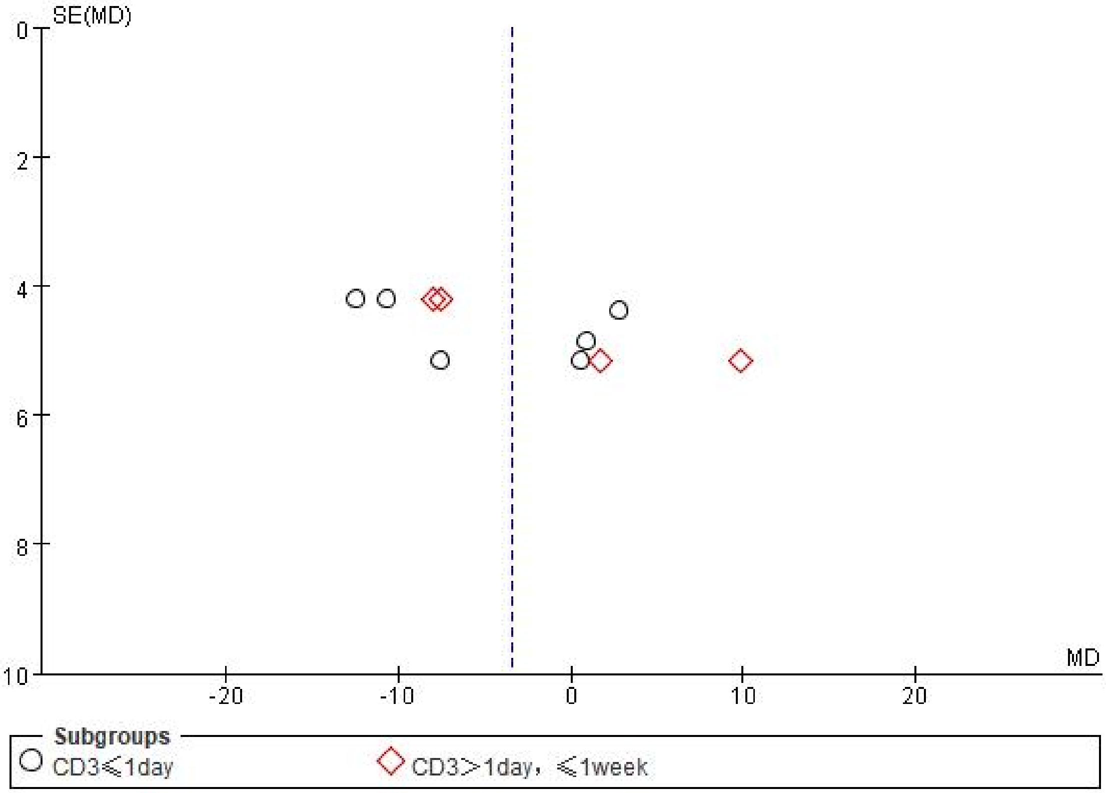

9.10CD4≤1day，CD4＞1day，≤1week

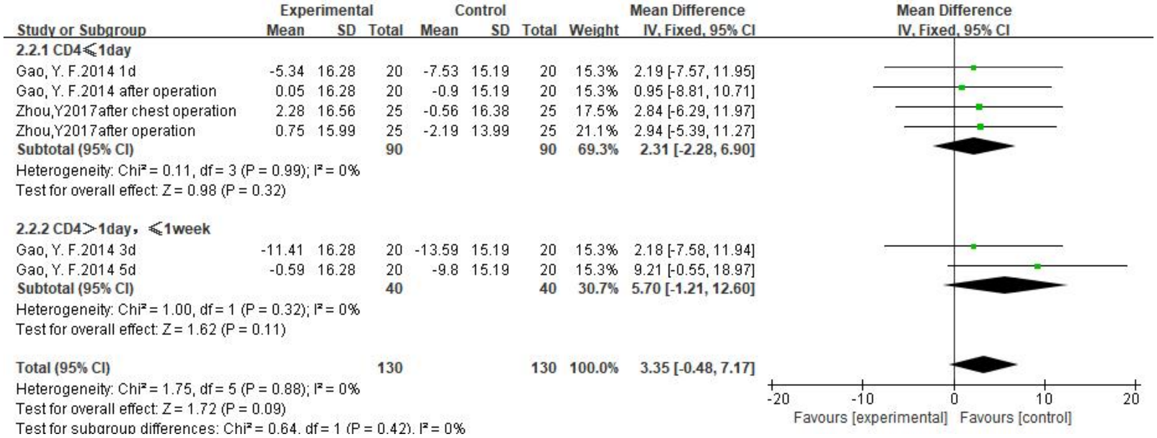

Study or Subgroup	Experimental Mean	Experimental SD	Experimental Total	Control Mean	Control SD	Control Total	Weight	Mean Difference IV, Fixed, 95% CI
**2.2.1 CD4≤1day**								
Gao, Y. F.2014 1d	-5.34	16.28	20	-7.53	15.19	20	15.3%	2.19 [-7.57, 11.95]
Gao, Y. F.2014 after operation	0.05	16.28	20	-0.9	15.19	20	15.3%	0.95 [-8.81, 10.71]
Zhou,Y2017after chest operation	2.28	16.56	25	-0.56	16.38	25	17.5%	2.84 [-6.29, 11.97]
Zhou,Y2017after operation	0.75	15.99	25	-2.19	13.99	25	21.1%	2.94 [-5.39, 11.27]
**Subtotal (95% CI)**			**90**			**90**	**69.3%**	**2.31 [-2.28, 6.90]**
Heterogeneity: Chi² = 0.11, df = 3 (P = 0.99); I² = 0%								
Test for overall effect: Z = 0.98 (P = 0.32)								
**2.2.2 CD4>1day，≤1week**								
Gao, Y. F.2014 3d	-11.41	16.28	20	-13.59	15.19	20	15.3%	2.18 [-7.58, 11.94]
Gao, Y. F.2014 5d	-0.59	16.28	20	-9.8	15.19	20	15.3%	9.21 [-0.55, 18.97]
**Subtotal (95% CI)**			**40**			**40**	**30.7%**	**5.70 [-1.21, 12.60]**
Heterogeneity: Chi² = 1.00, df = 1 (P = 0.32); I² = 0%								
Test for overall effect: Z = 1.62 (P = 0.11)								
**Total (95% CI)**			**130**			**130**	**100.0%**	**3.35 [-0.48, 7.17]**
Heterogeneity: Chi² = 1.75, df = 5 (P = 0.88); I² = 0%								
Test for overall effect: Z = 1.72 (P = 0.09)								
Test for subgroup differences: Chi² = 0.64, df = 1 (P = 0.42), I² = 0%								

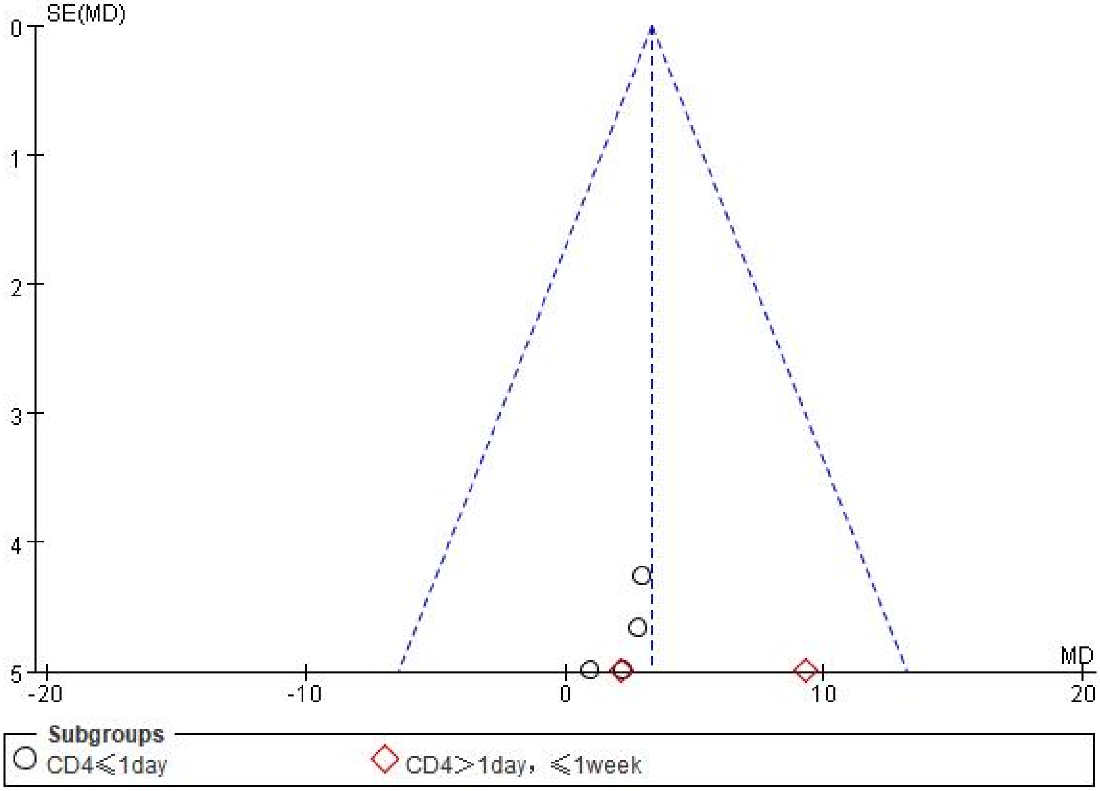

9.11CD8≤1day，CD8＞1day，≤1week

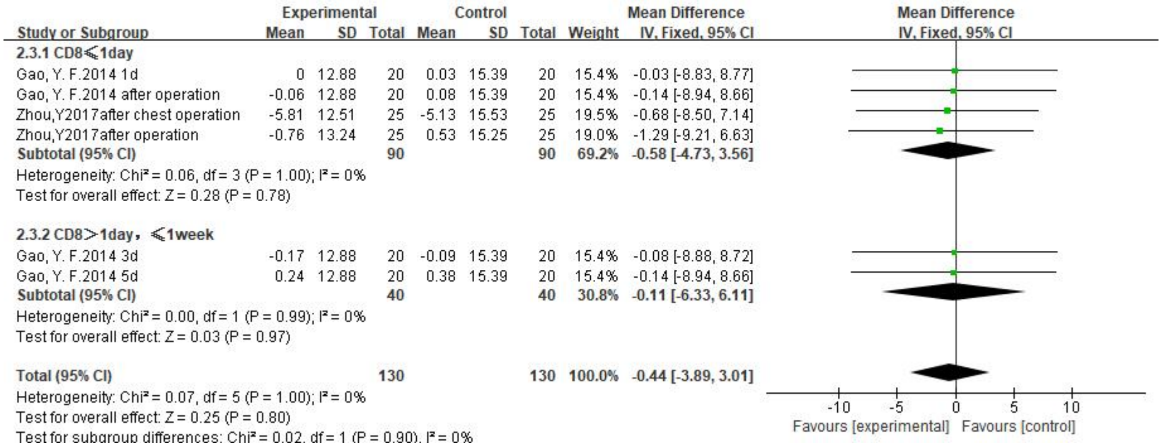

Study or Subgroup	Experimental Mean	Experimental SD	Experimental Total	Control Mean	Control SD	Control Total	Weight	Mean Difference IV, Fixed, 95% CI
**2.3.1 CD8≤1day**								
Gao, Y. F.2014 1d	0	12.88	20	0.03	15.39	20	15.4%	-0.03 [-8.83, 8.77]
Gao, Y. F.2014 after operation	-0.06	12.88	20	0.08	15.39	20	15.4%	-0.14 [-8.94, 8.66]
Zhou,Y2017after chest operation	-5.81	12.51	25	-5.13	15.53	25	19.5%	-0.68 [-8.50, 7.14]
Zhou,Y2017after operation	-0.76	13.24	25	0.53	15.25	25	19.0%	-1.29 [-9.21, 6.63]
**Subtotal (95% CI)**			**90**			**90**	**69.2%**	**-0.58 [-4.73, 3.56]**
Heterogeneity: Chi² = 0.06, df = 3 (P = 1.00); I² = 0%								
Test for overall effect: Z = 0.28 (P = 0.78)								
**2.3.2 CD8>1day，≤1week**								
Gao, Y. F.2014 3d	-0.17	12.88	20	-0.09	15.39	20	15.4%	-0.08 [-8.88, 8.72]
Gao, Y. F.2014 5d	0.24	12.88	20	0.38	15.39	20	15.4%	-0.14 [-8.94, 8.66]
**Subtotal (95% CI)**			**40**			**40**	**30.8%**	**-0.11 [-6.33, 6.11]**
Heterogeneity: Chi² = 0.00, df = 1 (P = 0.99); I² = 0%								
Test for overall effect: Z = 0.03 (P = 0.97)								
**Total (95% CI)**			**130**			**130**	**100.0%**	**-0.44 [-3.89, 3.01]**
Heterogeneity: Chi² = 0.07, df = 5 (P = 1.00); I² = 0%								
Test for overall effect: Z = 0.25 (P = 0.80)								
Test for subgroup differences: Chi² = 0.02, df = 1 (P = 0.90), I² = 0%								

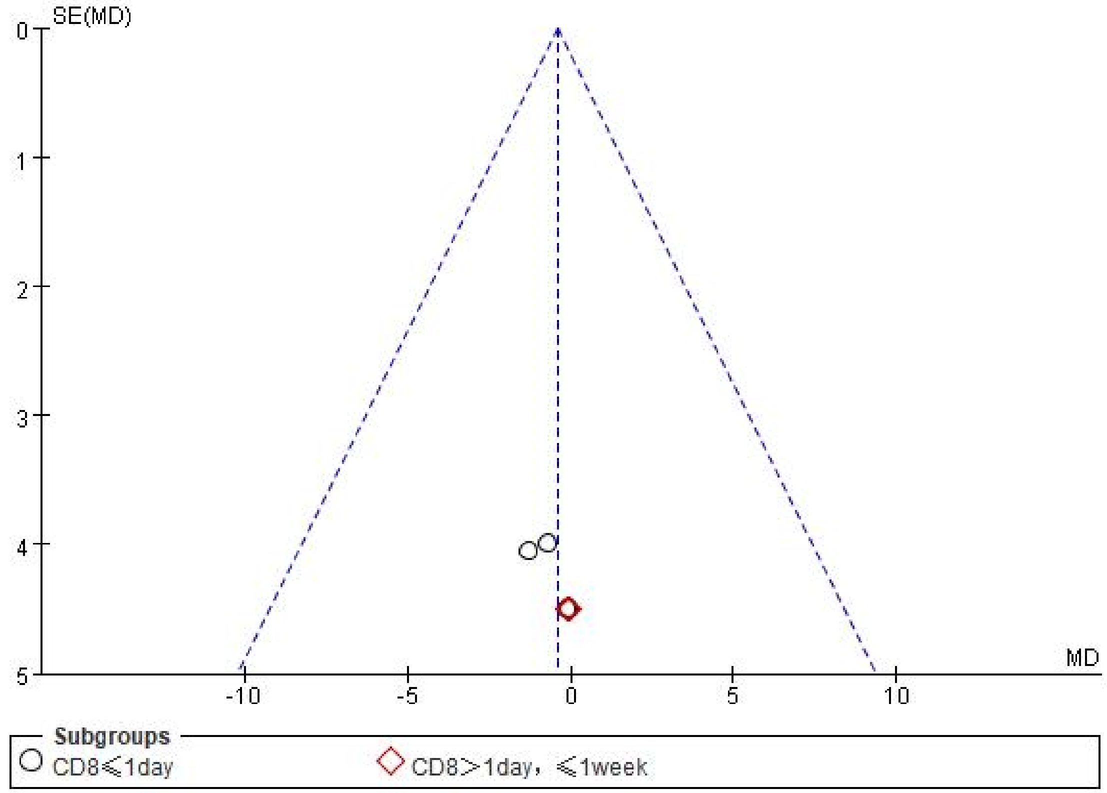

9.12CD4/CD8≤1day，CD4/CD8＞1day，≤1week

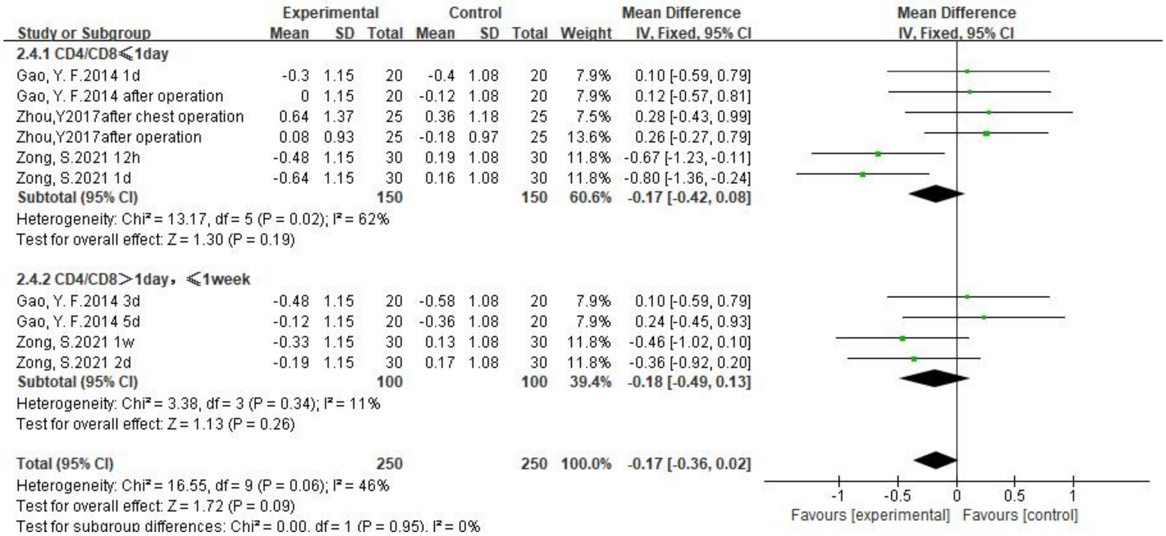

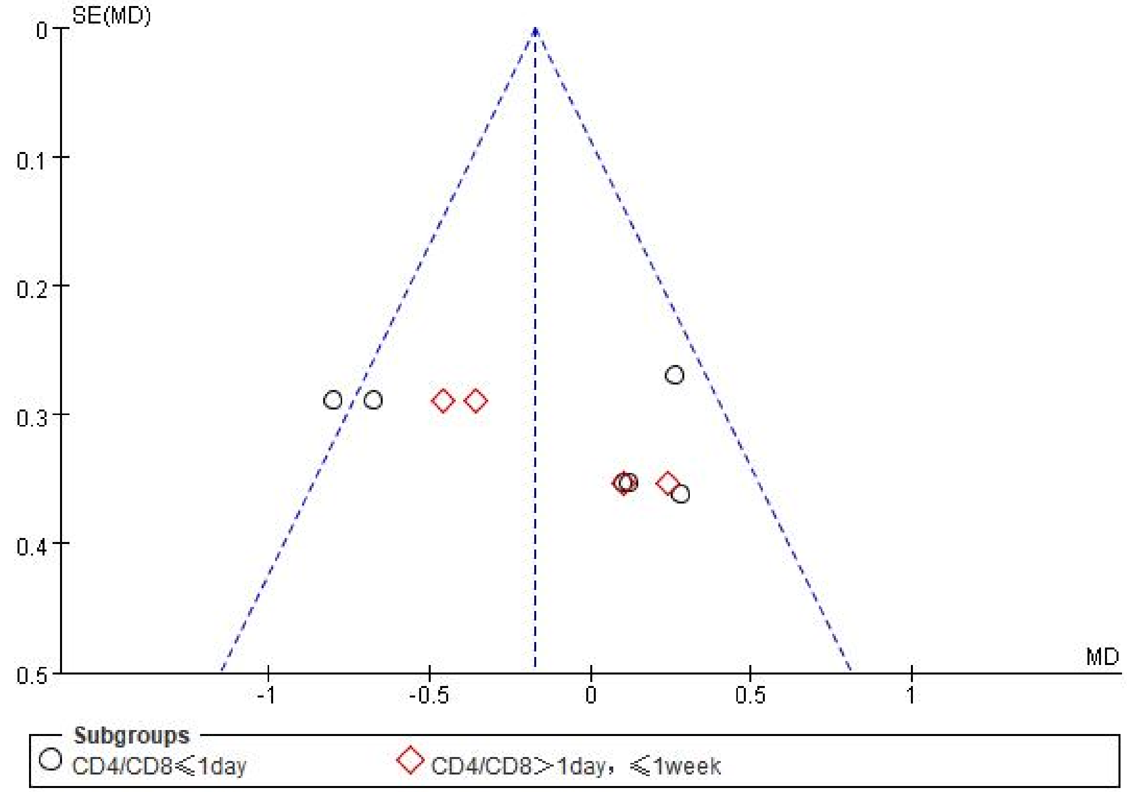

Parecoxib

9.13CD3≤1day，CD3＞1day，≤1week

Study or Subgroup	Experimental Mean	Experimental SD	Experimental Total	Control Mean	Control SD	Control Total	Weight	Mean Difference IV, Fixed, 95% CI
**2.1.1 CD3≤1day**								
Chen, M.2022 1d	-3.4	6.44	30	-3.6	5.67	30	16.7%	0.20 [-2.87, 3.27]
Chen, M.2022after operation	-10.1	6.3	30	-7	5.67	30	17.2%	-3.10 [-6.13, -0.07]
Wang, R. D.2020 1d	-2.68	6.66	40	-4.67	7.21	40	17.1%	1.99 [-1.05, 5.03]
Wang, R. D.2020 after operation	-0.59	6.56	40	-2.18	7.78	40	15.9%	1.59 [-1.56, 4.74]
**Subtotal (95% CI)**			**140**			**140**	**66.8%**	**0.14 [-1.40, 1.68]**
Heterogeneity: Chi² = 6.62, df = 3 (P = 0.09); I² = 55%								
Test for overall effect: Z = 0.18 (P = 0.86)								
**2.1.2 CD3＞1day，≤1week**								
Chen, M.2022 2d	-6.9	6.3	30	-7	5.67	30	17.2%	0.10 [-2.93, 3.13]
Wang, R. D.2020 3d	-3.47	7.11	40	-7.35	7.2	40	16.0%	3.88 [0.74, 7.02]
**Subtotal (95% CI)**			**70**			**70**	**33.2%**	**1.93 [-0.25, 4.11]**
Heterogeneity: Chi² = 2.88, df = 1 (P = 0.09); I² = 65%								
Test for overall effect: Z = 1.73 (P = 0.08)								
**Total (95% CI)**			**210**			**210**	**100.0%**	**0.73 [-0.52, 1.99]**
Heterogeneity: Chi² = 11.23, df = 5 (P = 0.05); I² = 55%								
Test for overall effect: Z = 1.14 (P = 0.25)								
Test for subgroup differences: Chi² = 1.72, df = 1 (P = 0.19), I² = 42.0%								

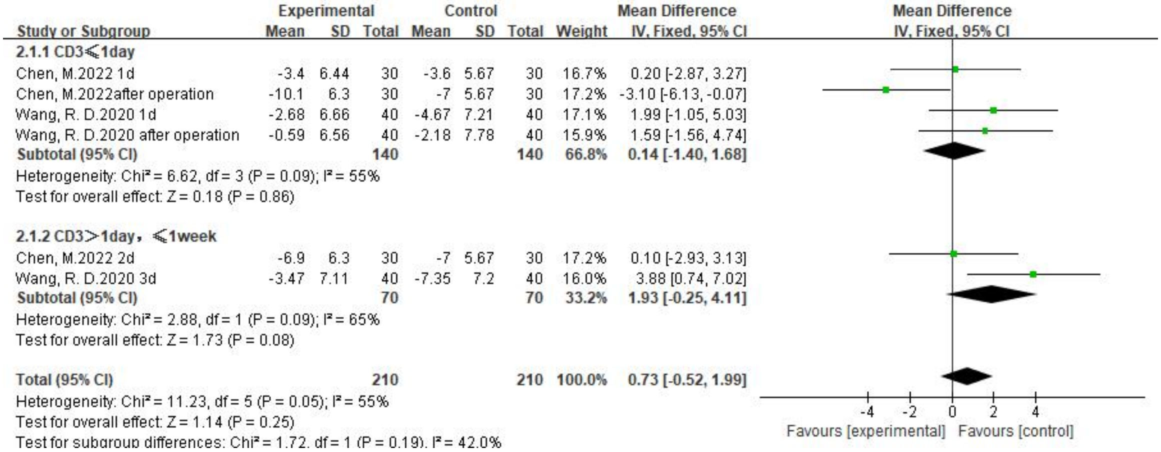

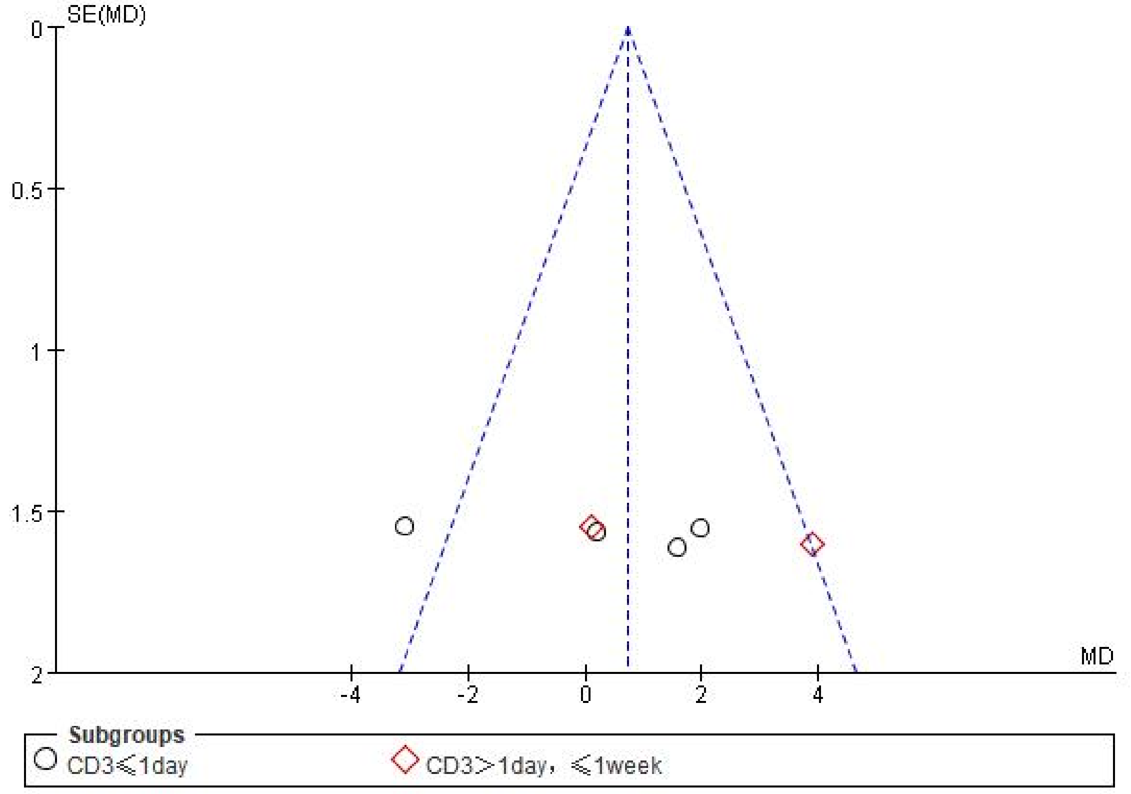

9.14CD4≤1day，CD4＞1day，≤1week

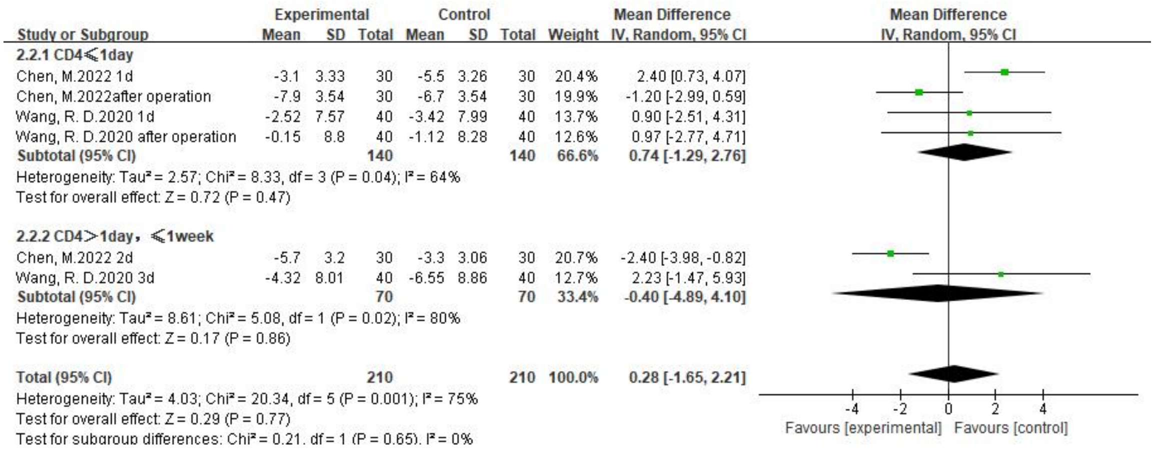

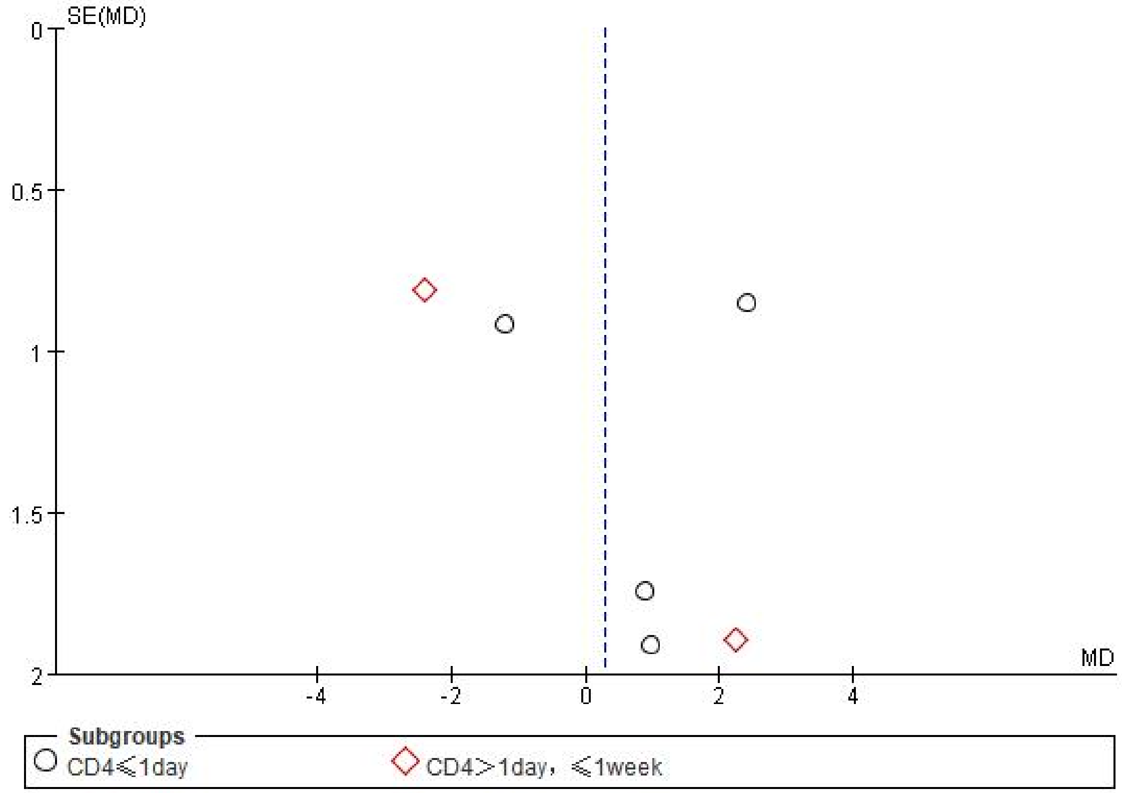

9.15CD8≤1day，CD8＞1day，≤1week

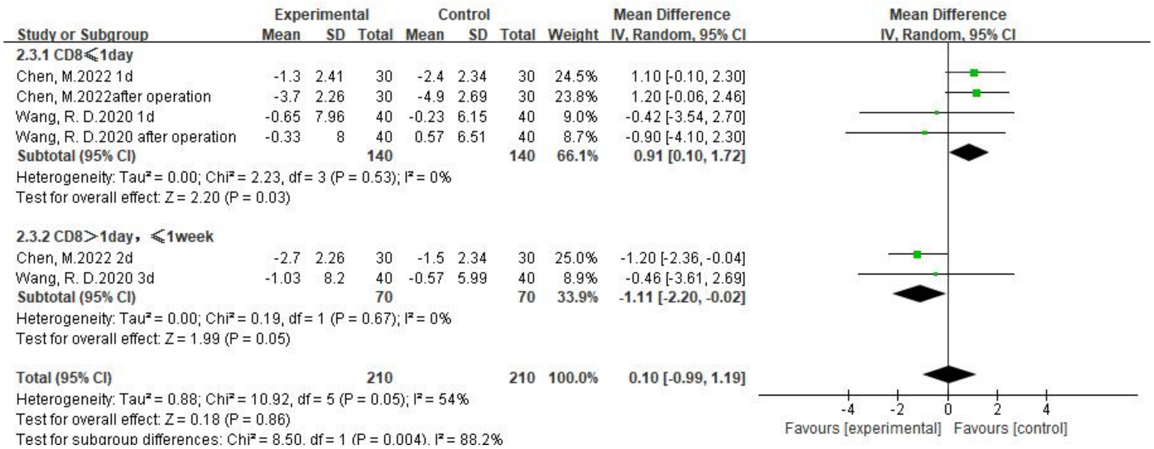

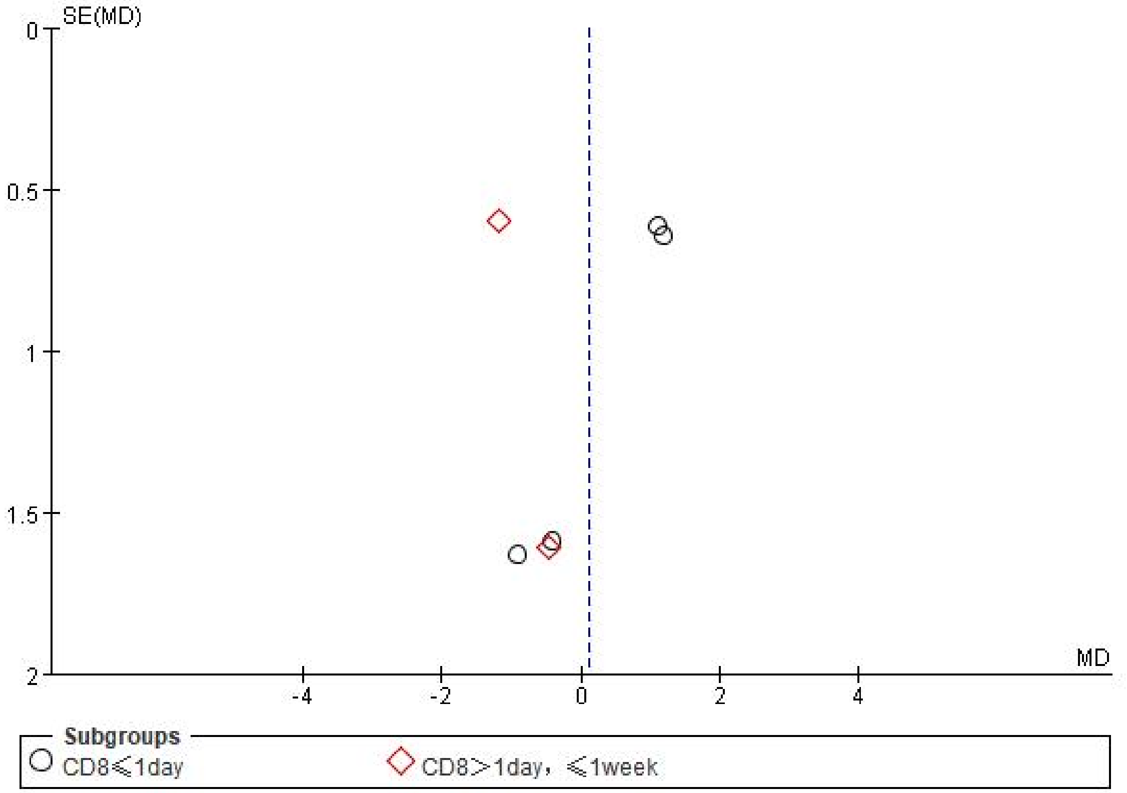

9.16CD4/CD8≤1day，CD4/CD8＞1day，≤1week

Study or Subgroup	Experimental Mean	Experimental SD	Experimental Total	Control Mean	Control SD	Control Total	Weight	Mean Difference IV, Fixed, 95% CI
**2.4.1 CD4/CD8⩽1day**								
Wang, R. D.2020 1d	-0.08	0.55	40	-0.14	0.65	40	27.7%	0.06 [-0.20, 0.32]
Wang, R. D.2020 after operation	0	0.59	40	-0.1	0.64	40	26.5%	0.10 [-0.17, 0.37]
**Subtotal (95% CI)**			**80**			**80**	**54.2%**	**0.08 [-0.11, 0.27]**
Heterogeneity: Chi² = 0.04, df = 1 (P = 0.84); I² = 0%								
Test for overall effect: Z = 0.83 (P = 0.41)								
**2.4.2 CD4/CD8>1day，⩽1week**								
Wang, R. D.2020 3d	-0.13	0.054	40	-0.25	0.66	40	45.8%	0.12 [-0.09, 0.33]
**Subtotal (95% CI)**			**40**			**40**	**45.8%**	**0.12 [-0.09, 0.33]**
Heterogeneity: Not applicable								
Test for overall effect: Z = 1.15 (P = 0.25)								
**Total (95% CI)**			**120**			**120**	**100.0%**	**0.10 [-0.04, 0.24]**
Heterogeneity: Chi² = 0.12, df = 2 (P = 0.94); I² = 0%								
Test for overall effect: Z = 1.38 (P = 0.17)								
Test for subgroup differences: Chi² = 0.08, df = 1 (P = 0.78), I² = 0%								

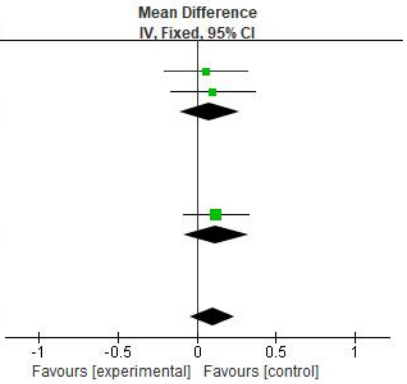

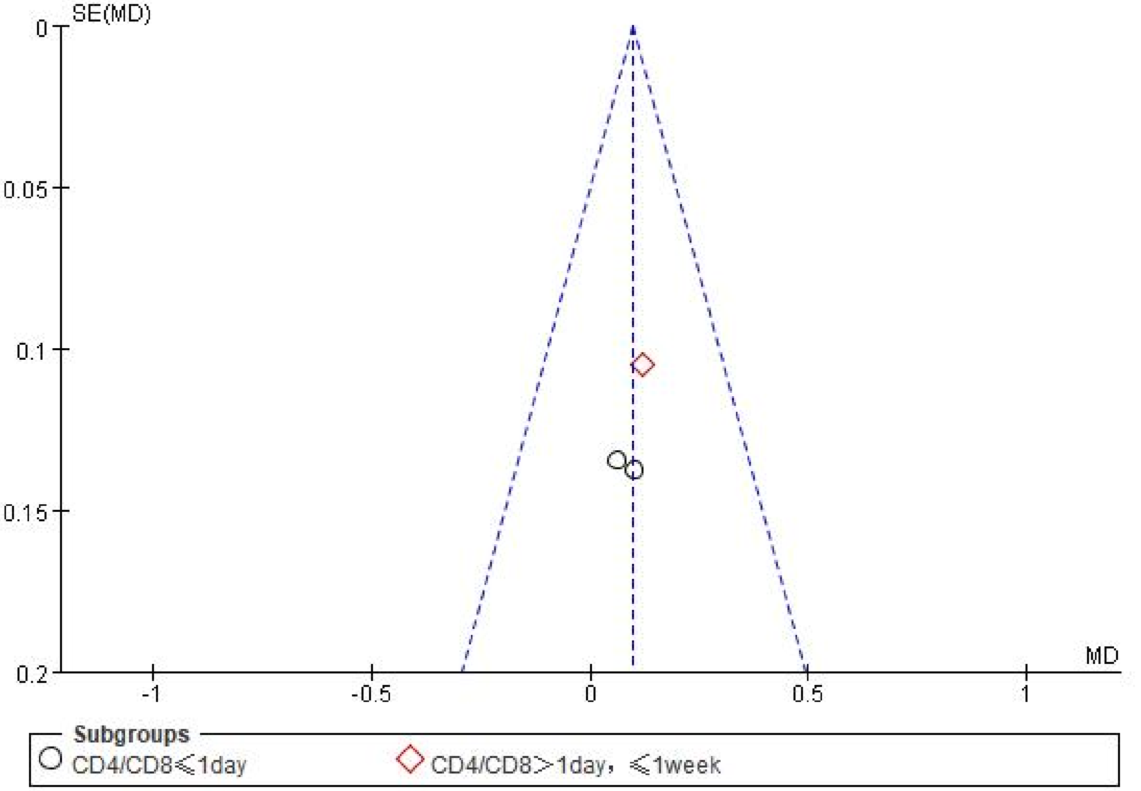

Morphine

9.17CD3≤1day，CD3＞1day，≤1week

Study or Subgroup	Experimental Mean	Experimental SD	Experimental Total	Control Mean	Control SD	Control Total	Weight	Mean Difference IV, Fixed, 95% CI
**2.1.1 CD3≤1day**								
Bakr, M. A. E. M.2016KETOROL1d	-10.98	6.15	20	-8.47	3.63	20	10.2%	-2.51 [-5.64, 0.62]
Bakr, M. A. E. M.2016KETOROL90min	-19.37	6.24	20	-19.17	4.95	20	8.2%	-0.20 [-3.69, 3.29]
Bakr, M. A. E. M.2016TRAM1d	-10.98	6.15	20	-11.89	5.99	20	7.0%	0.91 [-2.85, 4.67]
Bakr, M. A. E. M.2016TRAM90min	-19.37	6.24	20	-21.6	5.1	20	8.0%	2.23 [-1.30, 5.76]
Li, W. K.2008 1d	-16.2	7.25	16	-13.7	8.57	16	3.3%	-2.50 [-8.00, 3.00]
Li, W. K.2008 4h	-15	8.37	16	-6.5	9.08	16	2.7%	-8.50 [-14.55, -2.45]
Li, W. K.2008after operation	-3	9.56	16	-1.6	10.51	16	2.1%	-1.40 [-8.36, 5.56]
Shen, J. C.2014 1d	-6.69	7.4	30	-5.26	7.26	29	7.1%	-1.43 [-5.17, 2.31]
Shen, J. C.2014 2h	-3.12	7.4	30	-3.1	7.26	29	7.1%	-0.02 [-3.76, 3.72]
Wang, Z. Y.2006TRAM+LORN1d	-3.44	7.4	14	-5.13	7.26	15	3.5%	1.69 [-3.65, 7.03]
Wang, Z. Y.2006TRAM+LORN90min	-1.46	7.4	14	-1.95	7.26	15	3.5%	0.49 [-4.85, 5.83]
Wang, Z. Y.2006TRAM1d	-3.44	7.4	14	-0.04	7.26	15	3.5%	-3.40 [-8.74, 1.94]
Wang, Z. Y.2006TRAM90min	-1.46	7.4	14	-0.61	7.26	15	3.5%	-0.85 [-6.19, 4.49]
**Subtotal (95% CI)**			**244**			**246**	**69.6%**	**-0.79 [-1.98, 0.41]**
Heterogeneity: Chi² = 13.74, df = 12 (P = 0.32); I² = 13%								
Test for overall effect: Z = 1.29 (P = 0.20)								
**2.1.2 CD3＞1day，≤1week**								
Li, W. K.2008 3d	-21.8	9.27	16	-14.9	10.26	16	2.2%	-6.90 [-13.68, -0.12]
Shen, J. C.2014 2d	-13.26	7.4	30	-8.74	7.26	29	7.1%	-4.52 [-8.26, -0.78]
Shen, J. C.2014 5d	-9.93	7.4	30	-6.91	7.26	29	7.1%	-3.02 [-6.76, 0.72]
Wang, Z. Y.2006TRAM+LORN2d	-10.69	7.4	14	-8.74	7.26	15	3.5%	-1.95 [-7.29, 3.39]
Wang, Z. Y.2006TRAM+LORN3d	-8.25	7.4	14	-2.48	7.26	15	3.5%	-5.77 [-11.11, -0.43]
Wang, Z. Y.2006TRAM2d	-10.69	7.4	14	-2.35	7.26	15	3.5%	-8.34 [-13.68, -3.00]
Wang, Z. Y.2006TRAM3d	-8.25	7.4	14	-1.45	7.26	15	3.5%	-6.80 [-12.14, -1.46]
**Subtotal (95% CI)**			**132**			**134**	**30.4%**	**-4.89 [-6.70, -3.08]**
Heterogeneity: Chi² = 4.70, df = 6 (P = 0.58); I² = 0%								
Test for overall effect: Z = 5.29 (P < 0.00001)								
**Total (95% CI)**			**376**			**380**	**100.0%**	**-2.03 [-3.03, -1.03]**
Heterogeneity: Chi² = 32.17, df = 19 (P = 0.03); I² = 41%								
Test for overall effect: Z = 3.99 (P < 0.0001)								
Test for subgroup differences: Chi² = 13.73, df = 1 (P = 0.0002), I² = 92.7%								

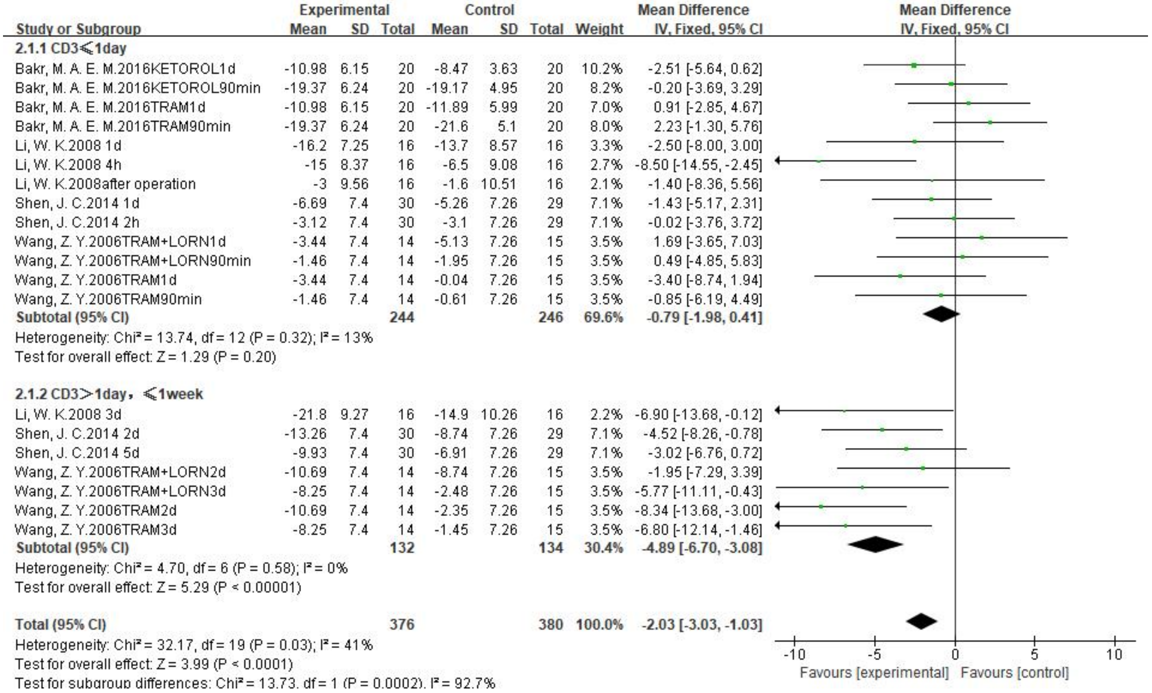

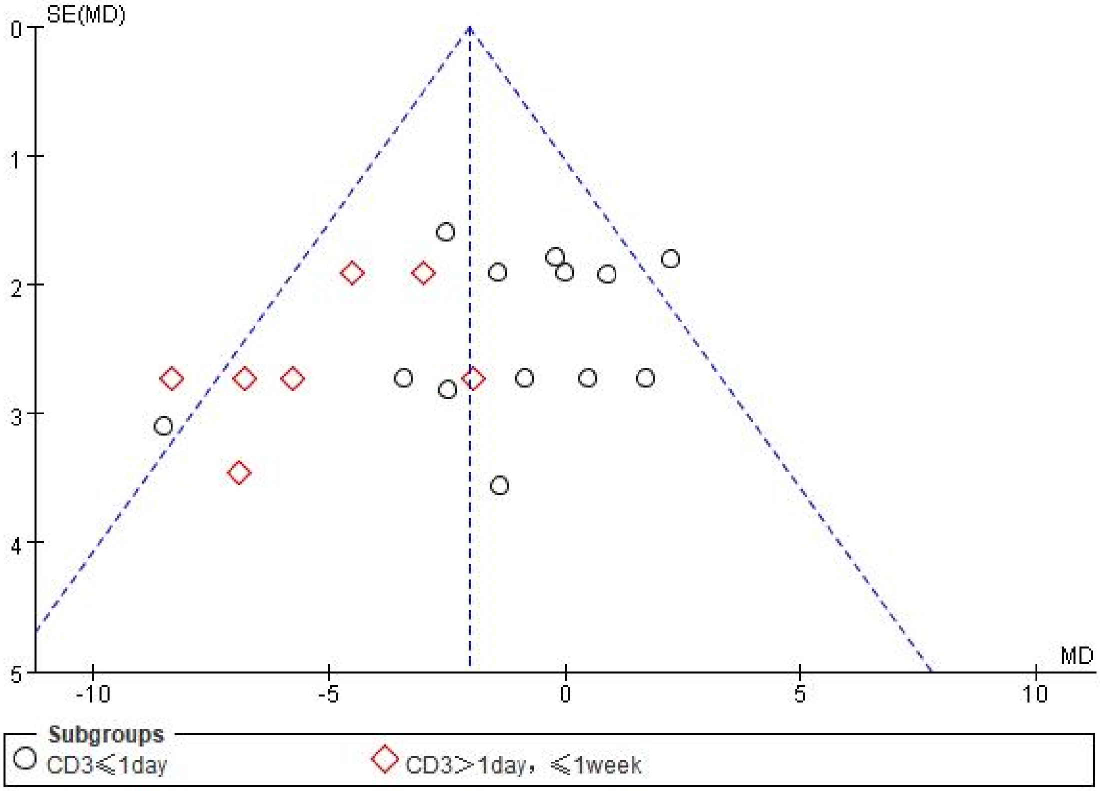

9.18CD4≤1day，CD4＞1day，≤1week

Study or Subgroup	Experimental Mean	Experimental SD	Experimental Total	Control Mean	Control SD	Control Total	Weight	Mean Difference IV, Random, 95% CI
**2.2.1 CD4≤1day**								
Bakr, M. A. E. M.2016KETOROL1d	-21	3.07	20	-6.32	3.15	20	5.5%	-14.68 [-16.61, -12.75]
Bakr, M. A. E. M.2016KETOROL90min	-21.13	2.99	20	-13.54	2.67	20	5.5%	-7.59 [-9.35, -5.83]
Bakr, M. A. E. M.2016TRAM1d	-21	3.07	20	-5.1	5.62	20	5.3%	-15.90 [-18.71, -13.09]
Bakr, M. A. E. M.2016TRAM90min	-21.13	2.99	20	-17.2	4.59	20	5.4%	-3.93 [-6.33, -1.53]
Li, W. K.2008 1d	-16.9	7.38	16	-14.6	8.88	16	4.6%	-2.30 [-7.96, 3.36]
Li, W. K.2008 4h	-10.9	10.26	16	-4.3	11.06	16	4.0%	-6.60 [-13.99, 0.79]
Li, W. K.2008after operation	-0.6	7.38	16	-0.9	7.96	16	4.7%	0.30 [-5.02, 5.62]
Shen, J. C.2014 1d	-4.51	5.47	30	-3.53	6.59	29	5.2%	-0.98 [-4.08, 2.12]
Shen, J. C.2014 2h	-1.95	5.47	30	-1.99	6.59	29	5.2%	0.04 [-3.06, 3.14]
Wang, Z. Y.2006TRAM+LORN1d	-5.19	5.47	14	-6.49	6.59	15	4.9%	1.30 [-3.10, 5.70]
Wang, Z. Y.2006TRAM+LORN90min	-0.29	5.47	14	-0.79	6.59	15	4.9%	0.50 [-3.90, 4.90]
Wang, Z. Y.2006TRAM1d	-5.19	5.47	14	-4.53	6.59	15	4.9%	-0.66 [-5.06, 3.74]
Wang, Z. Y.2006TRAM90min	-0.29	5.47	14	-0.31	6.59	15	4.9%	0.02 [-4.38, 4.42]
**Subtotal (95% CI)**			**244**			**246**	**65.1%**	**-4.02 [-7.60, -0.44]**
Heterogeneity: Tau² = 38.93; Chi² = 192.75, df = 12 (P < 0.00001); I² = 94%								
Test for overall effect: Z = 2.20 (P = 0.03)								
**2.2.2 CD4>1day，≤1week**								
Li, W. K.2008 3d	-17.5	6.6	16	-15.2	8.82	16	4.6%	-2.30 [-7.70, 3.10]
Shen, J. C.2014 2d	-8.95	5.47	30	-5.38	6.59	29	5.2%	-3.57 [-6.67, -0.47]
Shen, J. C.2014 5d	-6.73	5.47	30	-4.94	6.59	29	5.2%	-1.79 [-4.89, 1.31]
Wang, Z. Y.2006TRAM+LORN2d	-4.98	5.47	14	-5.26	6.59	15	4.9%	0.28 [-4.12, 4.68]
Wang, Z. Y.2006TRAM+LORN3d	3.93	5.47	14	-1.09	6.59	15	4.9%	5.02 [0.62, 9.42]
Wang, Z. Y.2006TRAM2d	-4.98	5.47	14	-0.92	6.59	15	4.9%	-4.06 [-8.46, 0.34]
Wang, Z. Y.2006TRAM3d	3.93	5.47	14	-0.86	6.59	15	4.9%	4.79 [0.39, 9.19]
**Subtotal (95% CI)**			**132**			**134**	**34.9%**	**-0.34 [-3.06, 2.39]**
Heterogeneity: Tau² = 9.05; Chi² = 19.04, df = 6 (P = 0.004); I² = 68%								
Test for overall effect: Z = 0.24 (P = 0.81)								
**Total (95% CI)**			**376**			**380**	**100.0%**	**-2.71 [-5.53, 0.11]**
Heterogeneity: Tau² = 36.96; Chi² = 258.32, df = 19 (P < 0.00001); I² = 93%								
Test for overall effect: Z = 1.89 (P = 0.06)								
Test for subgroup differences: Chi² = 2.58, df = 1 (P = 0.11), I² = 61.2%								

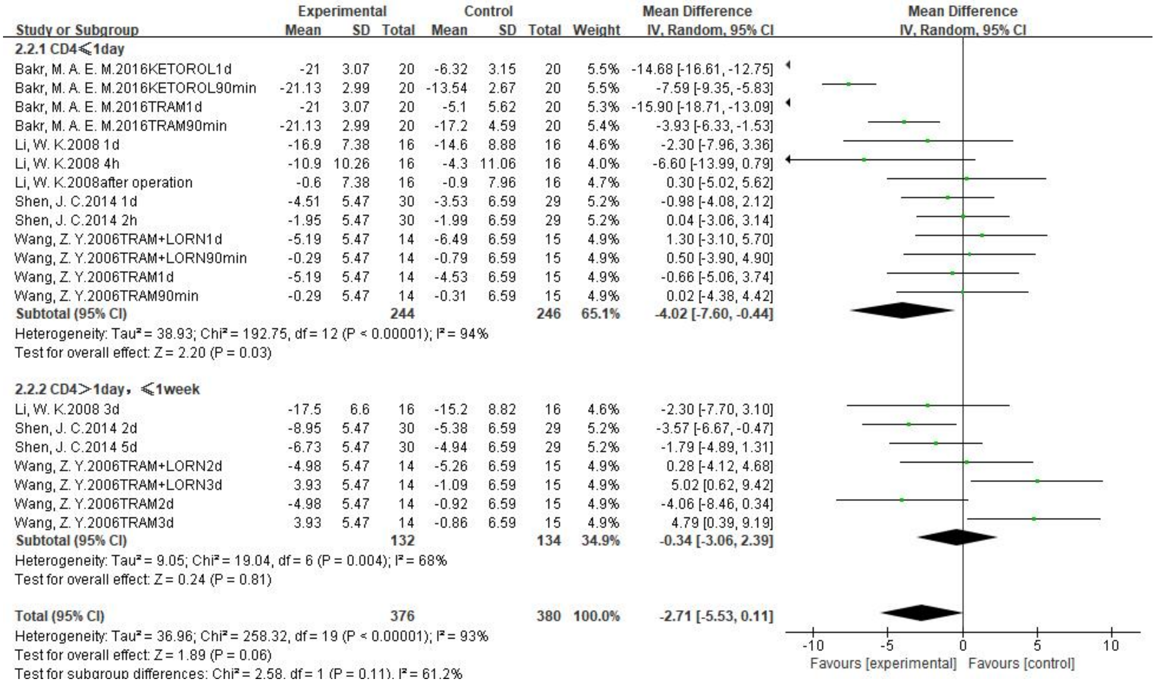

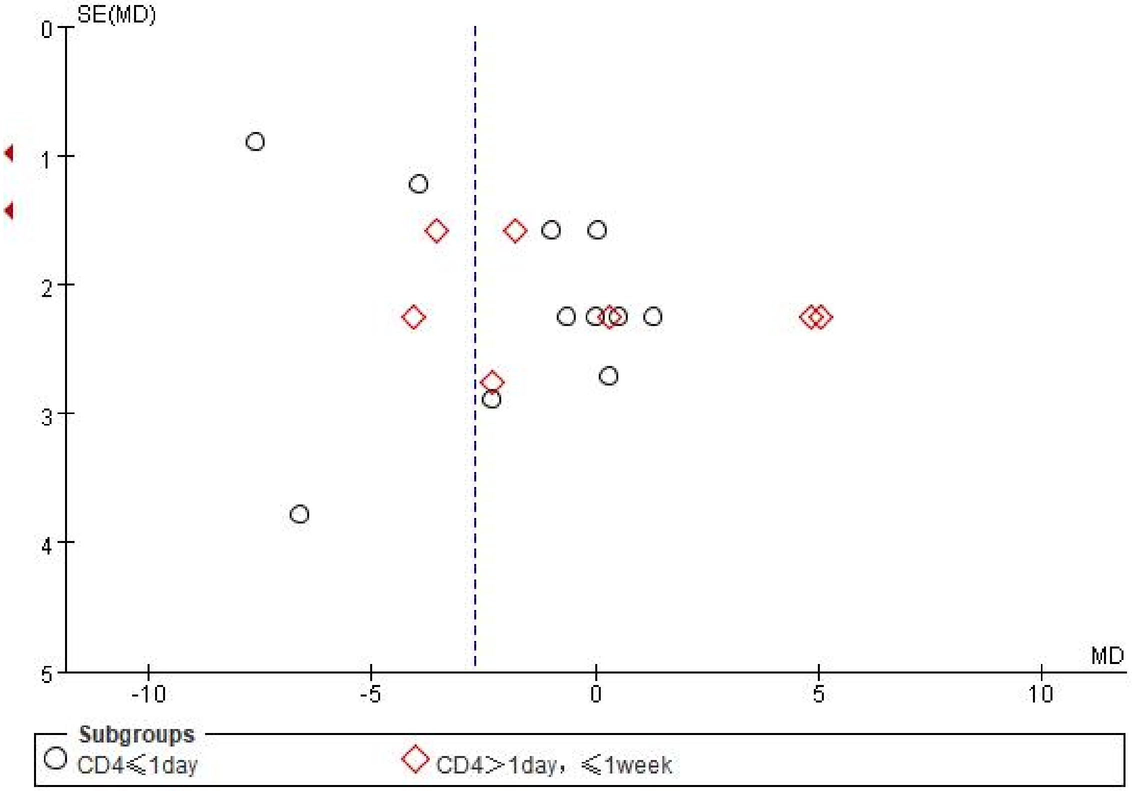

9.19CD8≤1day，CD8＞1day，≤1week

Study or Subgroup	Experimental Mean	Experimental SD	Experimental Total	Control Mean	Control SD	Control Total	Weight	Mean Difference IV, Fixed, 95% CI
**2.3.1 CD8≤1day**								
Bakr, M. A. E. M.2016KETOROL1d	-14.33	3.44	20	-4.34	3.61	20	10.6%	-9.99 [-12.18, -7.80]
Bakr, M. A. E. M.2016KETOROL90min	-14.39	3.35	20	-13.13	4.86	20	7.6%	-1.26 [-3.85, 1.33]
Bakr, M. A. E. M.2016TRAM1d	-14.33	3.44	20	-6.38	4.27	20	8.8%	-7.95 [-10.35, -5.55]
Bakr, M. A. E. M.2016TRAM90min	-14.39	3.35	20	-12.54	3.45	20	11.4%	-1.85 [-3.96, 0.26]
Li, W. K.2008 1d	0.3	7.61	16	0.9	7	16	2.0%	-0.60 [-5.67, 4.47]
Li, W. K.2008 4h	0.8	7.45	16	0.9	6.19	16	2.3%	-0.10 [-4.85, 4.65]
Li, W. K.2008after operation	0.4	7.08	16	0.3	7.28	16	2.1%	0.10 [-4.88, 5.08]
Shen, J. C.2014 1d	-0.89	5.31	30	-0.75	5.49	29	6.7%	-0.14 [-2.90, 2.62]
Shen, J. C.2014 2h	0.41	5.31	30	-0.78	5.49	29	6.7%	1.19 [-1.57, 3.95]
Wang, Z. Y.2006TRAM+LORN1d	0.21	5.31	14	1.37	5.49	15	3.3%	-1.16 [-5.09, 2.77]
Wang, Z. Y.2006TRAM+LORN90min	0.36	5.31	14	0.32	5.49	15	3.3%	0.04 [-3.89, 3.97]
Wang, Z. Y.2006TRAM1d	0.21	5.31	14	1.15	5.49	15	3.3%	-0.94 [-4.87, 2.99]
Wang, Z. Y.2006TRAM90min	0.36	5.31	14	0.07	5.49	15	3.3%	0.29 [-3.64, 4.22]
**Subtotal (95% CI)**			**244**			**246**	**71.3%**	**-2.90 [-3.75, -2.06]**
Heterogeneity: Chi² = 82.10, df = 12 (P < 0.00001); I² = 85%								
Test for overall effect: Z = 6.74 (P < 0.00001)								
**2.3.2 CD8＞1day，≤1week**								
Li, W. K.2008 3d	0.7	6.79	16	1.3	7.28	16	2.1%	-0.60 [-5.48, 4.28]
Shen, J. C.2014 2d	-1.82	5.31	30	-1.44	5.49	29	6.7%	-0.38 [-3.14, 2.38]
Shen, J. C.2014 5d	-1.5	5.31	30	-1.08	5.49	29	6.7%	-0.42 [-3.18, 2.34]
Wang, Z. Y.2006TRAM+LORN2d	0.31	5.31	14	1.72	5.49	15	3.3%	-1.41 [-5.34, 2.52]
Wang, Z. Y.2006TRAM+LORN3d	0.7	5.31	14	0.77	5.49	15	3.3%	-0.07 [-4.00, 3.86]
Wang, Z. Y.2006TRAM2d	0.31	5.31	14	0.02	5.49	15	3.3%	0.29 [-3.64, 4.22]
Wang, Z. Y.2006TRAM3d	0.7	5.31	14	0.04	5.49	15	3.3%	0.66 [-3.27, 4.59]
**Subtotal (95% CI)**			**132**			**134**	**28.7%**	**-0.29 [-1.62, 1.04]**
Heterogeneity: Chi² = 0.66, df = 6 (P = 1.00); I² = 0%								
Test for overall effect: Z = 0.43 (P = 0.67)								
**Total (95% CI)**			**376**			**380**	**100.0%**	**-2.16 [-2.87, -1.44]**
Heterogeneity: Chi² = 93.30, df = 19 (P < 0.00001); I² = 80%								
Test for overall effect: Z = 5.92 (P < 0.00001)								
Test for subgroup differences: Chi² = 10.54, df = 1 (P = 0.001), I² = 90.5%								

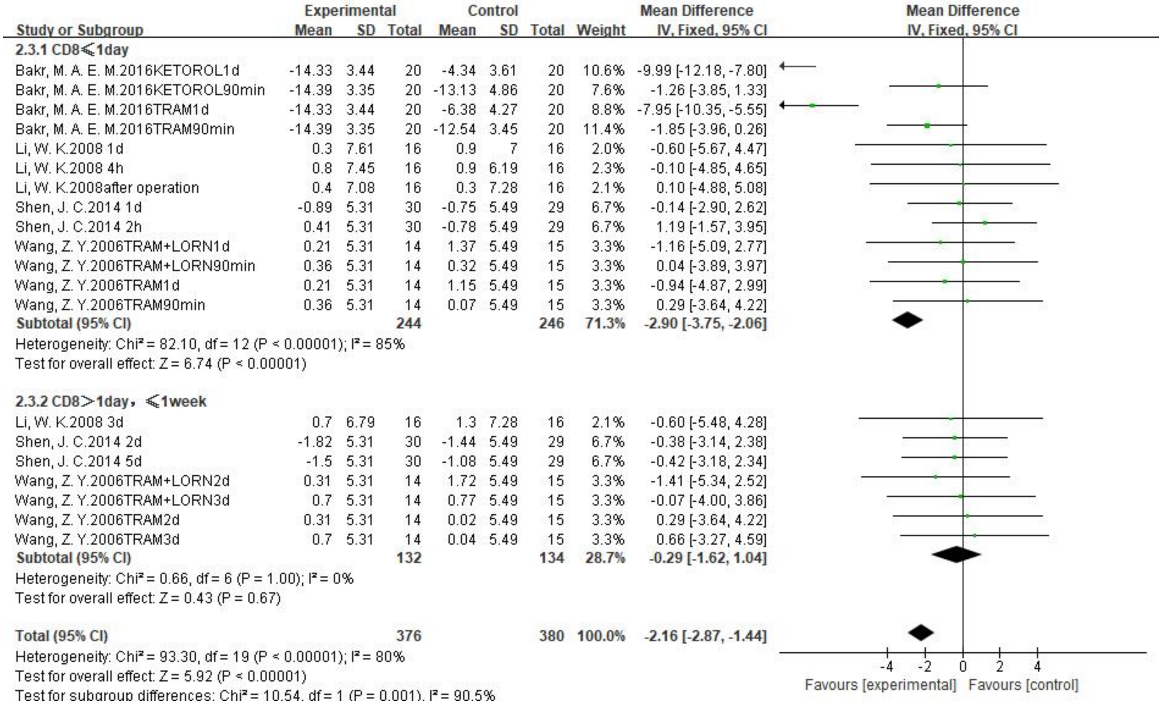

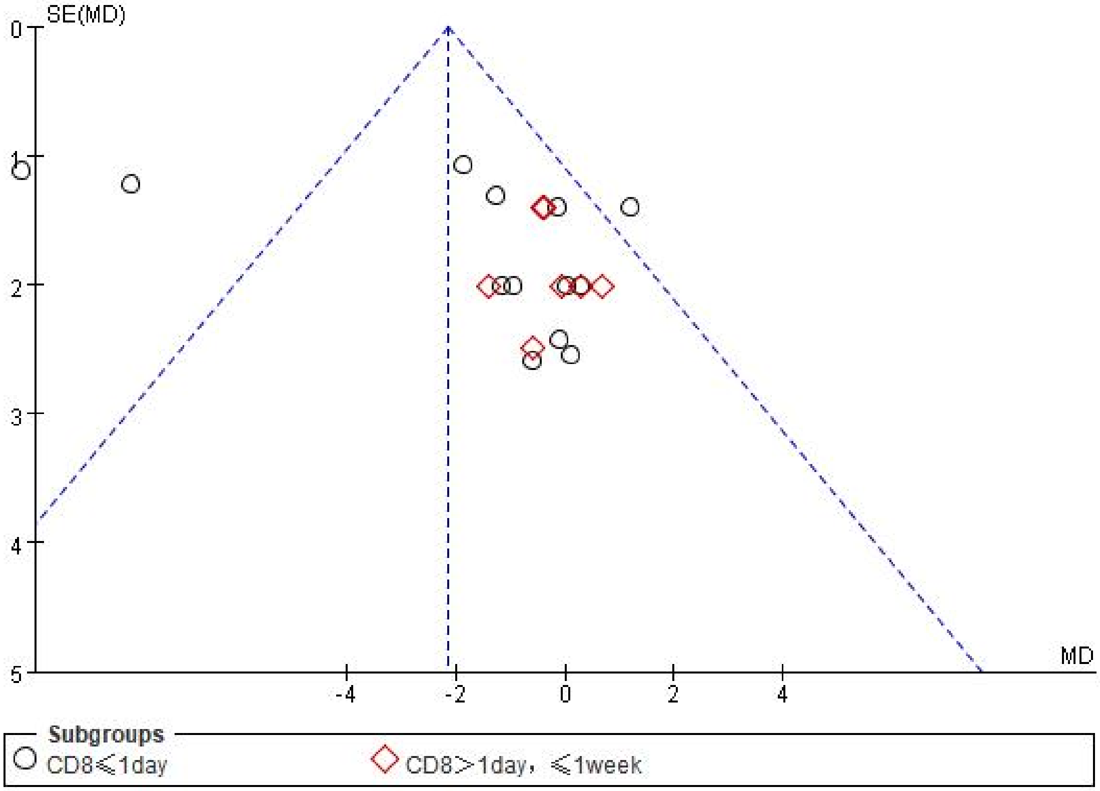

9.20CD4/CD8≤1day，CD4/CD8＞1day，≤1week

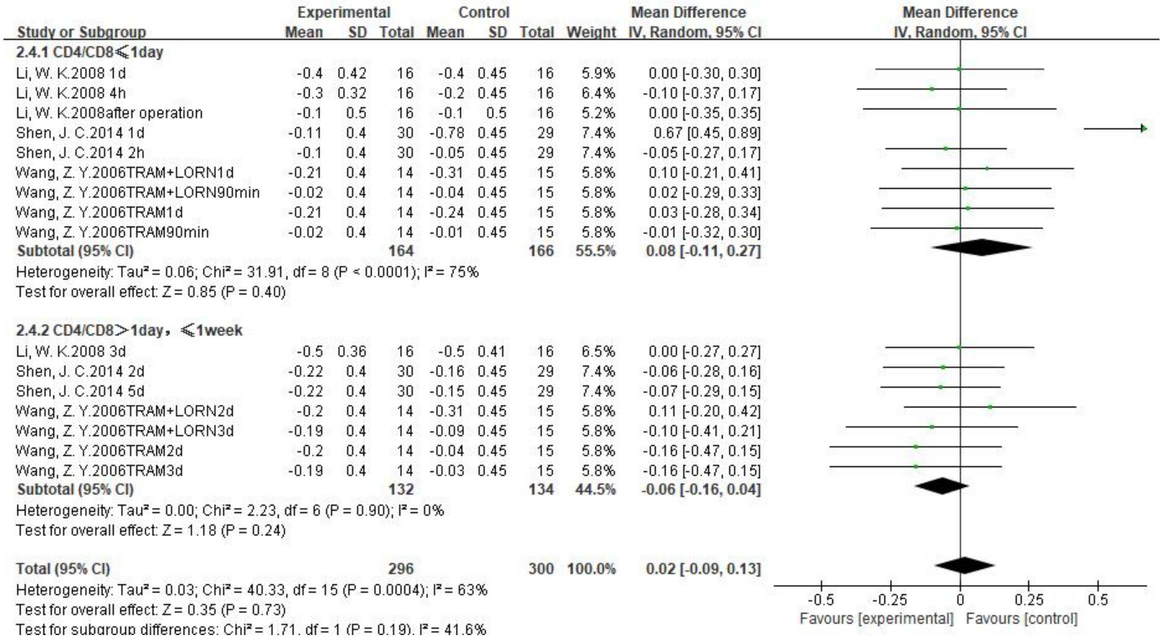

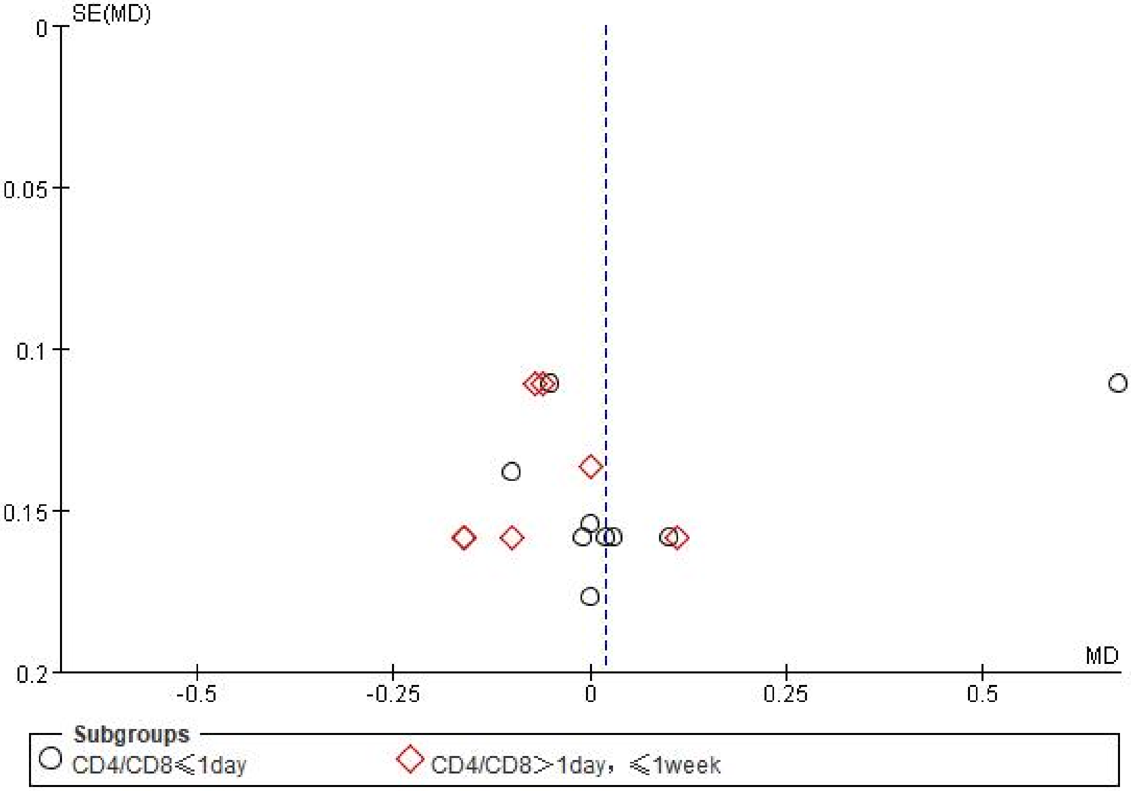

Dezocine

9.21CD3≤1day，CD3＞1day，≤1week

Study or Subgroup	Experimental Mean	Experimental SD	Experimental Total	Control Mean	Control SD	Control Total	Weight	Mean Difference IV, Fixed, 95% CI
**2.1.1 CD3⩽1day**								
Gao, Y. F.2014 1d	-7.82	12.36	20	-8.09	13.13	20	16.1%	0.27 [-7.63, 8.17]
Gao, Y. F.2014after operation	0.89	12.36	20	0.32	13.13	20	16.1%	0.57 [-7.33, 8.47]
Shao, Y. J.2018 1d	-12.86	12.36	44	-22.18	13.13	44	35.5%	9.32 [3.99, 14.65]
**Subtotal (95% CI)**			**84**			**84**	**67.7%**	**5.08 [1.23, 8.94]**
Heterogeneity: Chi² = 5.11, df = 2 (P = 0.08); I² = 61%								
Test for overall effect: Z = 2.58 (P = 0.010)								
**2.1.2 CD3>1day，⩽1week**								
Gao, Y. F.2014 3d	-12.73	12.36	20	-14.45	13.13	20	16.1%	1.72 [-6.18, 9.62]
Gao, Y. F.2014 5d	-0.83	12.36	20	-10.68	13.13	20	16.1%	9.85 [1.95, 17.75]
**Subtotal (95% CI)**			**40**			**40**	**32.3%**	**5.78 [0.20, 11.37]**
Heterogeneity: Chi² = 2.03, df = 1 (P = 0.15); I² = 51%								
Test for overall effect: Z = 2.03 (P = 0.04)								
**Total (95% CI)**			**124**			**124**	**100.0%**	**5.31 [2.13, 8.48]**
Heterogeneity: Chi² = 7.18, df = 4 (P = 0.13); I² = 44%								
Test for overall effect: Z = 3.28 (P = 0.001)								
Test for subgroup differences: Chi² = 0.04, df = 1 (P = 0.84), I² = 0%								

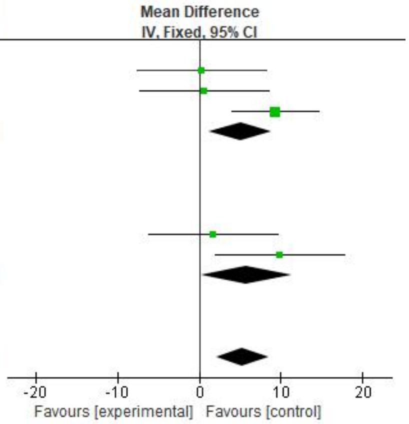

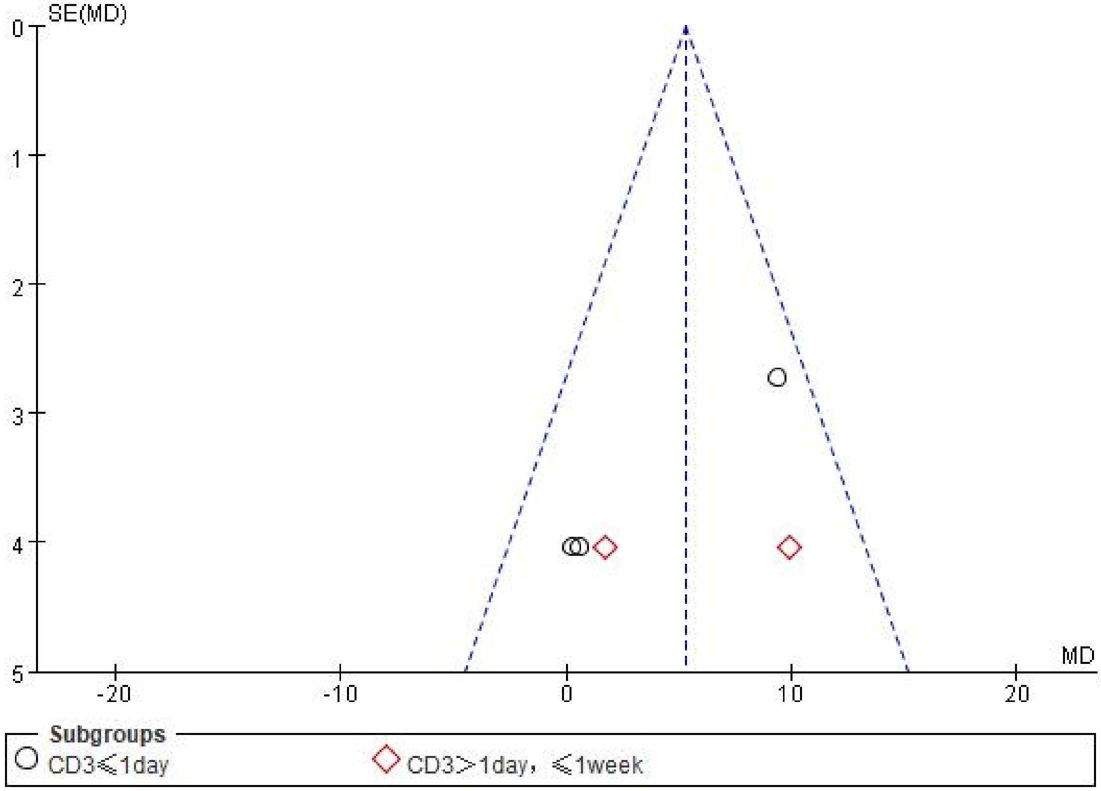

9.22CD4≤1day，CD4＞1day，≤1week

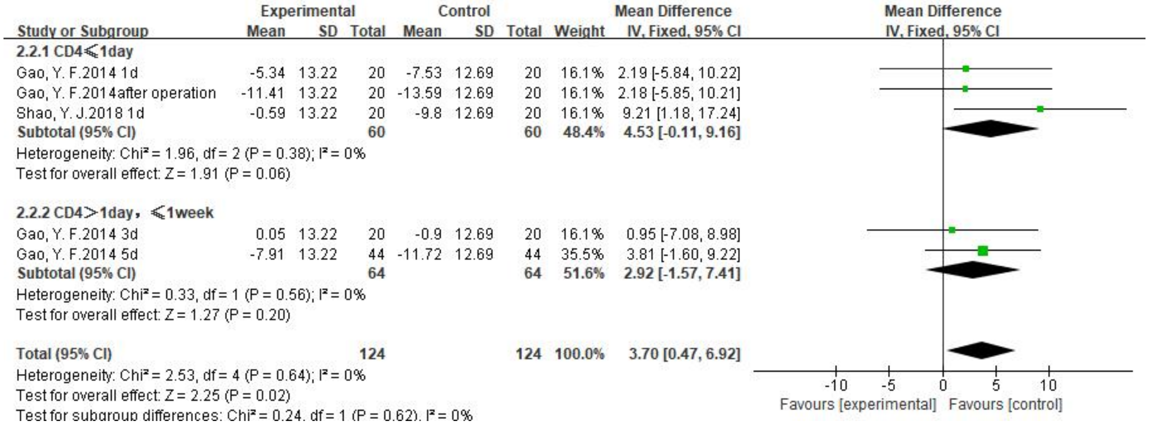

Study or Subgroup	Experimental Mean	Experimental SD	Experimental Total	Control Mean	Control SD	Control Total	Weight	Mean Difference IV, Fixed, 95% CI
**2.2.1 CD4≤1day**								
Gao, Y. F.2014 1d	-5.34	13.22	20	-7.53	12.69	20	16.1%	2.19 [-5.84, 10.22]
Gao, Y. F.2014after operation	-11.41	13.22	20	-13.59	12.69	20	16.1%	2.18 [-5.85, 10.21]
Shao, Y. J.2018 1d	-0.59	13.22	20	-9.8	12.69	20	16.1%	9.21 [1.18, 17.24]
**Subtotal (95% CI)**			**60**			**60**	**48.4%**	**4.53 [-0.11, 9.16]**
Heterogeneity: Chi² = 1.96, df = 2 (P = 0.38); I² = 0%								
Test for overall effect: Z = 1.91 (P = 0.06)								
**2.2.2 CD4>1day，≤1week**								
Gao, Y. F.2014 3d	0.05	13.22	20	-0.9	12.69	20	16.1%	0.95 [-7.08, 8.98]
Gao, Y. F.2014 5d	-7.91	13.22	44	-11.72	12.69	44	35.5%	3.81 [-1.60, 9.22]
**Subtotal (95% CI)**			**64**			**64**	**51.6%**	**2.92 [-1.57, 7.41]**
Heterogeneity: Chi² = 0.33, df = 1 (P = 0.56); I² = 0%								
Test for overall effect: Z = 1.27 (P = 0.20)								
**Total (95% CI)**			**124**			**124**	**100.0%**	**3.70 [0.47, 6.92]**
Heterogeneity: Chi² = 2.53, df = 4 (P = 0.64); I² = 0%								
Test for overall effect: Z = 2.25 (P = 0.02)								
Test for subgroup differences: Chi² = 0.24, df = 1 (P = 0.62), I² = 0%								

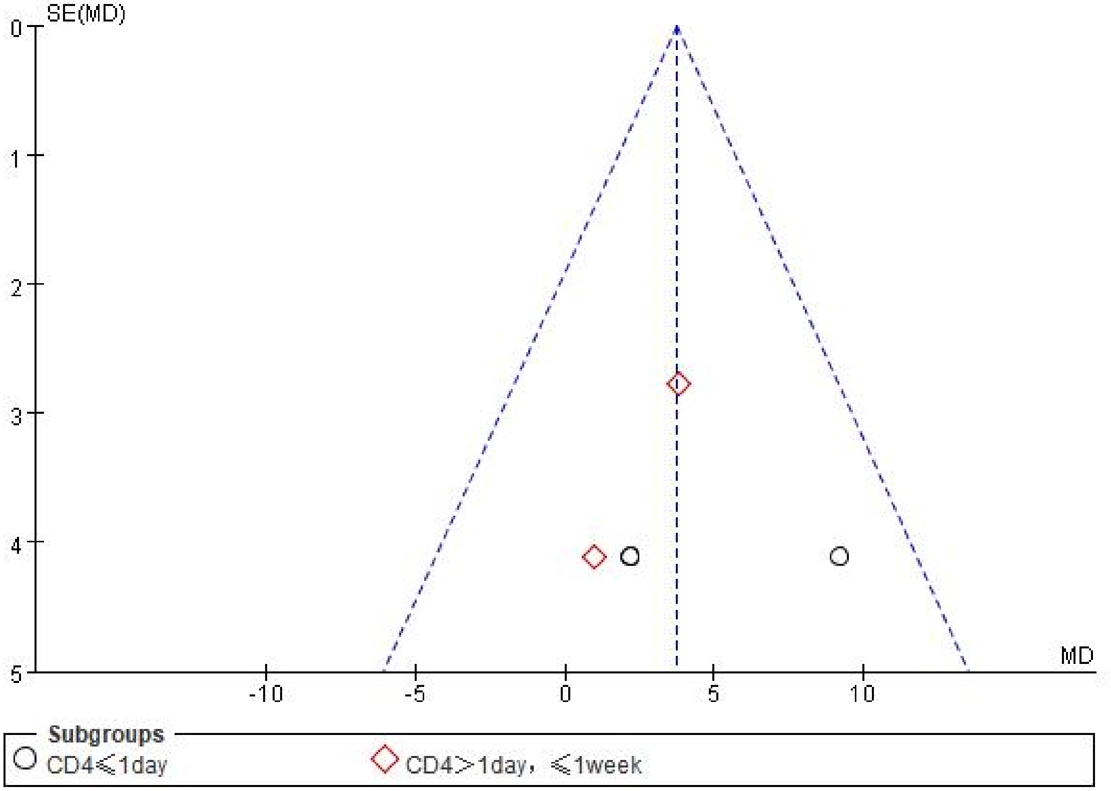

9.23CD8≤1day，CD8＞1day，≤1week

Study or Subgroup	Experimental Mean	Experimental SD	Experimental Total	Control Mean	Control SD	Control Total	Weight	Mean Difference IV, Fixed, 95% CI
**2.3.1 CD8≤1day**								
Gao, Y. F.2014 1d	0	11.59	20	0.03	11.39	20	16.1%	-0.03 [-7.15, 7.09]
Gao, Y. F.2014after operation	-0.06	11.59	20	0.08	11.39	20	16.1%	-0.14 [-7.26, 6.98]
Shao, Y. J.2018 1d	0.79	11.59	44	0.96	11.39	44	35.5%	-0.17 [-4.97, 4.63]
**Subtotal (95% CI)**			**84**			**84**	**67.7%**	**-0.13 [-3.60, 3.35]**
Heterogeneity: Chi² = 0.00, df = 2 (P = 1.00); I² = 0%								
Test for overall effect: Z = 0.07 (P = 0.94)								
**2.3.2 CD8＞1day，≤1week**								
Gao, Y. F.2014 3d	-0.17	11.59	20	-0.09	11.39	20	16.1%	-0.08 [-7.20, 7.04]
Gao, Y. F.2014 5d	0.24	11.59	20	0.38	11.39	20	16.1%	-0.14 [-7.26, 6.98]
**Subtotal (95% CI)**			**40**			**40**	**32.3%**	**-0.11 [-5.15, 4.93]**
Heterogeneity: Chi² = 0.00, df = 1 (P = 0.99); I² = 0%								
Test for overall effect: Z = 0.04 (P = 0.97)								
**Total (95% CI)**			**124**			**124**	**100.0%**	**-0.12 [-2.98, 2.74]**
Heterogeneity: Chi² = 0.00, df = 4 (P = 1.00); I² = 0%								
Test for overall effect: Z = 0.08 (P = 0.93)								
Test for subgroup differences: Chi² = 0.00, df = 1 (P = 1.00), I² = 0%								

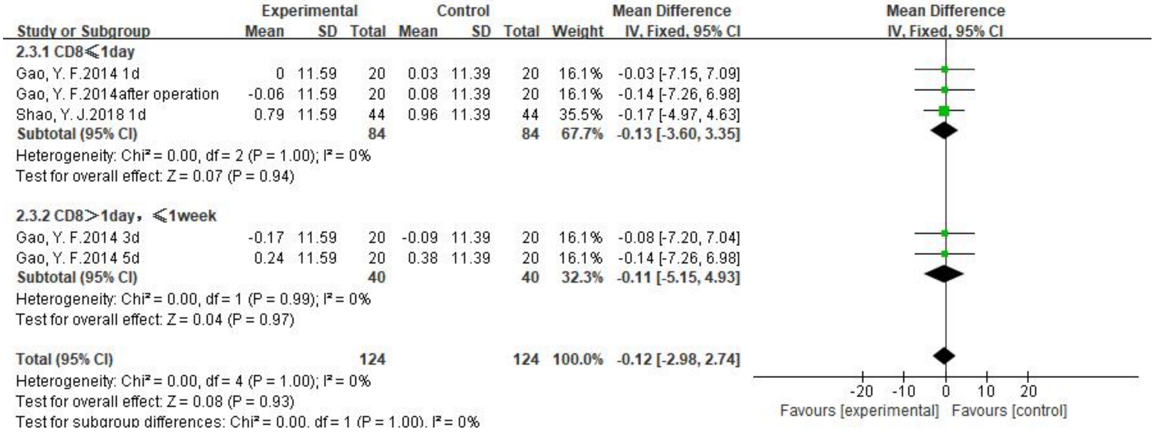

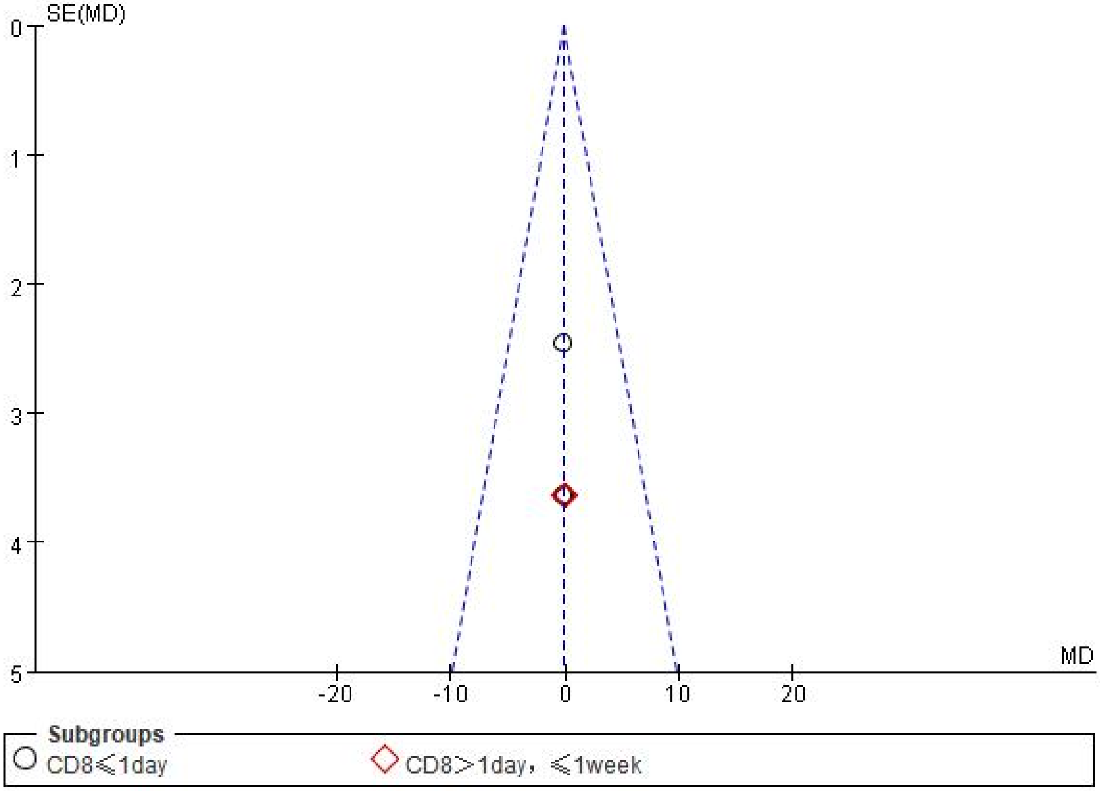

Tramadol

9.24CD3≤1day，CD3＞1day，≤1week

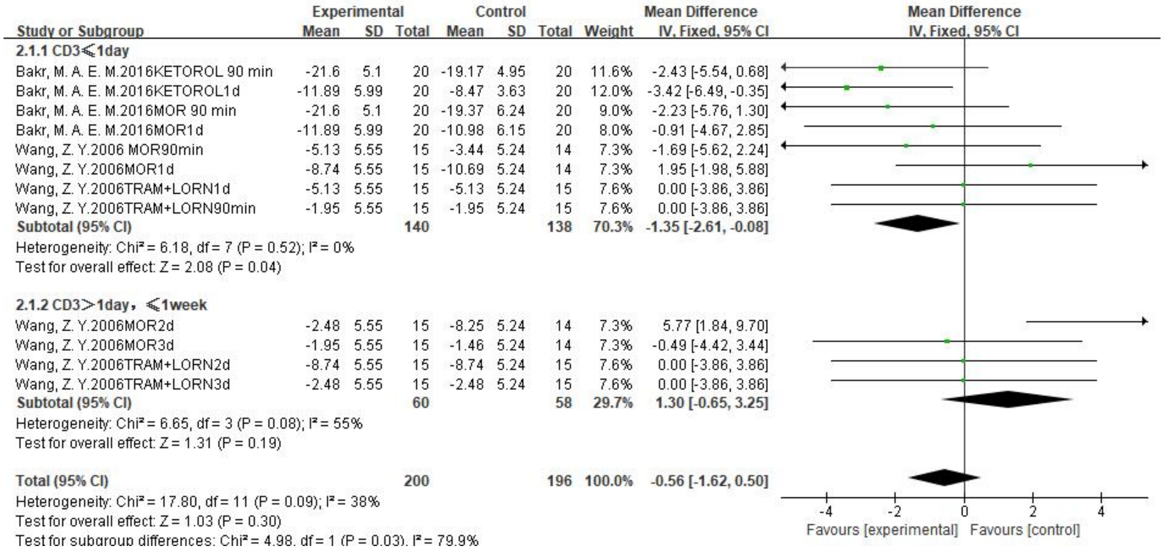

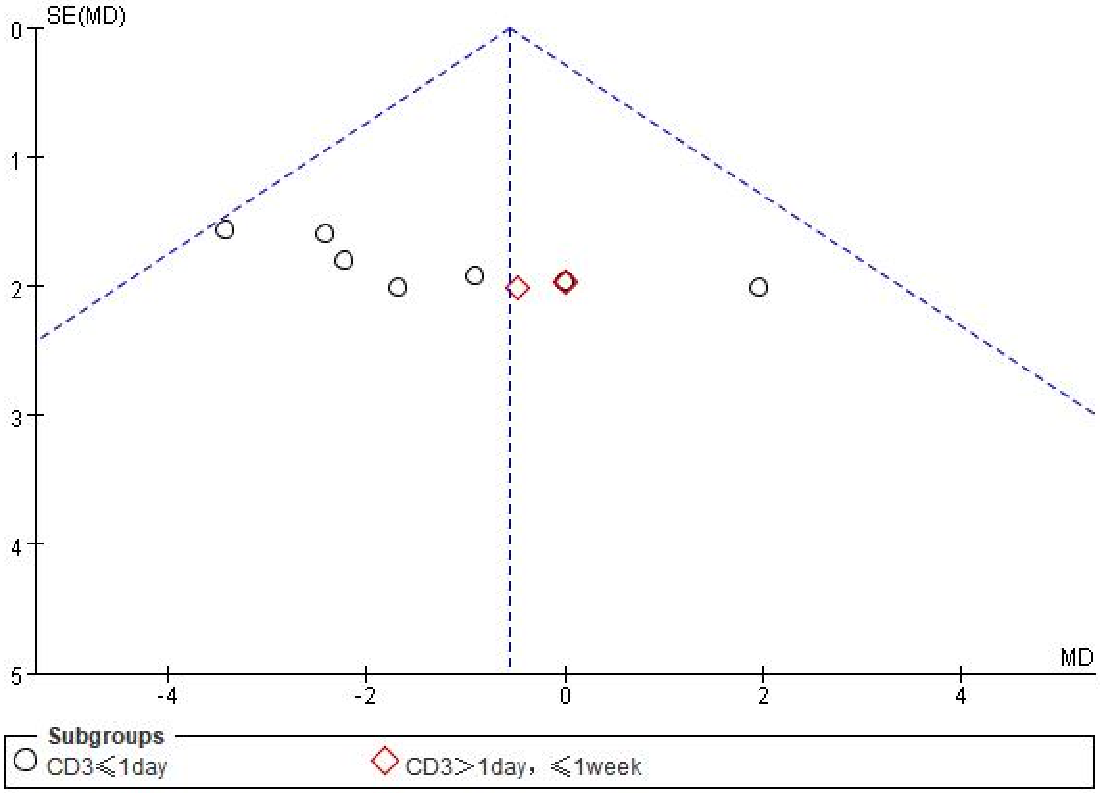

9.25CD4≤1day，CD4＞1day，≤1week

Study or Subgroup	Experimental Mean	Experimental SD	Experimental Total	Control Mean	Control SD	Control Total	Weight	Mean Difference IV, Random, 95% CI
**2.2.1 CD4≤1day**								
Wang, Z. Y.2006TRAM+LORN90min	-17.2	4.59	20	-13.54	2.67	20	8.6%	-3.66 [-5.99, -1.33]
Wang, Z. Y.2006TRAM+LORN1d	-5.1	5.62	20	-6.32	3.15	20	8.4%	1.22 [-1.60, 4.04]
Wang, Z. Y.2006MOR1d	-17.2	4.59	20	-21.13	2.99	20	8.5%	3.93 [1.53, 6.33]
Wang, Z. Y.2006 MOR90min	-5.1	5.62	20	-21	3.07	20	8.4%	15.90 [13.09, 18.71]
Bakr, M. A. E. M.2016MOR1d	-6.49	5.11	15	-5.19	2.97	14	8.3%	-1.30 [-4.32, 1.72]
Bakr, M. A. E. M.2016MOR 90 min	-5.26	5.11	15	-4.98	2.97	14	8.3%	-0.28 [-3.30, 2.74]
Bakr, M. A. E. M.2016KETOROL1d	-6.49	5.11	15	-6.49	2.97	15	8.3%	0.00 [-2.99, 2.99]
Bakr, M. A. E. M.2016KETOROL 90 min	-0.79	5.11	15	-0.79	2.97	15	8.3%	0.00 [-2.99, 2.99]
**Subtotal (95% CI)**			**140**			**138**	**66.9%**	**1.98 [-2.24, 6.19]**
Heterogeneity: Tau² = 34.93; Chi² = 129.91, df = 7 (P < 0.00001); I² = 95%								
Test for overall effect: Z = 0.92 (P = 0.36)								
**2.2.2 CD4＞1day，≤1week**								
Wang, Z. Y.2006TRAM+LORN3d	-1.09	5.11	15	3.93	2.97	14	8.3%	-5.02 [-8.04, -2.00]
Wang, Z. Y.2006TRAM+LORN2d	-0.79	5.11	15	-0.29	2.97	14	8.3%	-0.50 [-3.52, 2.52]
Wang, Z. Y.2006MOR3d	-5.26	5.11	15	-5.26	2.97	15	8.3%	0.00 [-2.99, 2.99]
Wang, Z. Y.2006MOR2d	-1.09	5.11	15	-1.09	2.97	15	8.3%	0.00 [-2.99, 2.99]
**Subtotal (95% CI)**			**60**			**58**	**33.1%**	**-1.38 [-3.76, 1.01]**
Heterogeneity: Tau² = 3.56; Chi² = 7.55, df = 3 (P = 0.06); I² = 60%								
Test for overall effect: Z = 1.13 (P = 0.26)								
**Total (95% CI)**			**200**			**196**	**100.0%**	**0.87 [-2.16, 3.90]**
Heterogeneity: Tau² = 26.52; Chi² = 150.27, df = 11 (P < 0.00001); I² = 93%								
Test for overall effect: Z = 0.56 (P = 0.58)								
Test for subgroup differences: Chi² = 1.84, df = 1 (P = 0.17), I² = 45.7%								

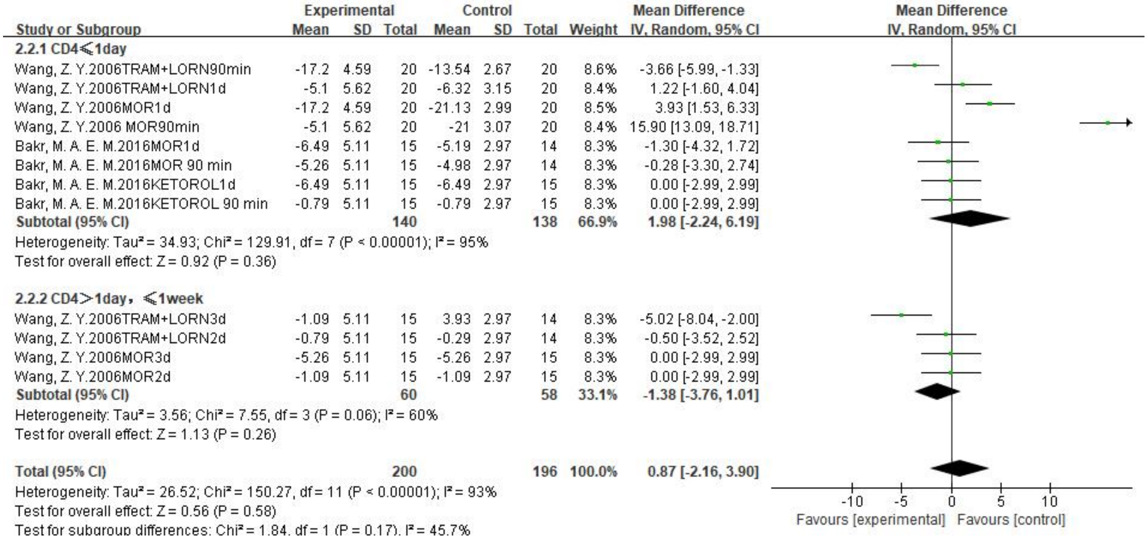

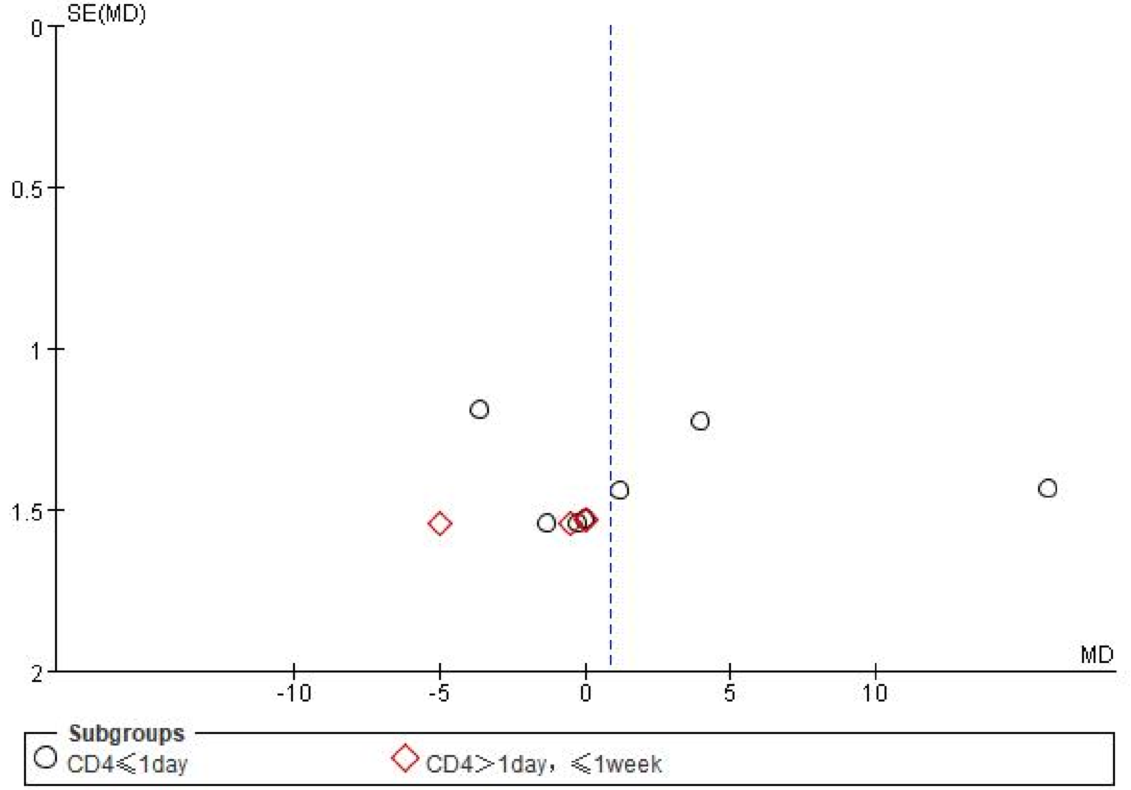

9.26CD8≤1day，CD8＞1day，≤1week

Study or Subgroup	Experimental Mean	Experimental SD	Experimental Total	Control Mean	Control SD	Control Total	Weight	Mean Difference IV, Random, 95% CI
**2.3.1 CD8⩽1day**								
Bakr, M. A. E. M.2016KETOROL 90 min	-12.54	3.45	20	-13.13	4.86	20	8.4%	0.59 [-2.02, 3.20]
Bakr, M. A. E. M.2016KETOROL1d	-6.38	4.27	20	-4.34	3.61	20	8.7%	-2.04 [-4.49, 0.41]
Bakr, M. A. E. M.2016MOR 90 min	-12.54	3.45	20	-14.39	3.35	20	9.2%	1.85 [-0.26, 3.96]
Bakr, M. A. E. M.2016MOR1d	-6.38	4.27	20	-14.33	3.44	20	8.7%	7.95 [5.55, 10.35]
Wang, Z. Y.2006 MOR90min	1.37	3.86	15	0.21	3.815	14	8.1%	1.16 [-1.63, 3.95]
Wang, Z. Y.2006MOR1d	1.72	3.86	15	0.31	3.815	14	8.1%	1.41 [-1.38, 4.20]
Wang, Z. Y.2006TRAM+LORN1d	1.37	3.86	15	1.37	3.815	15	8.2%	0.00 [-2.75, 2.75]
Wang, Z. Y.2006TRAM+LORN90min	0.32	3.86	15	0.32	3.815	15	8.2%	0.00 [-2.75, 2.75]
**Subtotal (95% CI)**			**140**			**138**	**67.5%**	**1.39 [-0.74, 3.52]**
Heterogeneity: Tau² = 7.68; Chi² = 38.61, df = 7 (P < 0.00001); I² = 82%								
Test for overall effect: Z = 1.28 (P = 0.20)								
**2.3.2 CD8＞1day，⩽1week**								
Wang, Z. Y.2006MOR2d	0.77	3.86	15	0.7	3.815	14	8.1%	0.07 [-2.72, 2.86]
Wang, Z. Y.2006MOR3d	0.32	3.86	15	0.36	3.815	14	8.1%	-0.04 [-2.83, 2.75]
Wang, Z. Y.2006TRAM+LORN2d	1.72	3.86	15	1.72	3.815	15	8.2%	0.00 [-2.75, 2.75]
Wang, Z. Y.2006TRAM+LORN3d	0.77	3.86	15	0.77	3.815	15	8.2%	0.00 [-2.75, 2.75]
**Subtotal (95% CI)**			**60**			**58**	**32.5%**	**0.01 [-1.38, 1.39]**
Heterogeneity: Tau² = 0.00; Chi² = 0.00, df = 3 (P = 1.00); I² = 0%								
Test for overall effect: Z = 0.01 (P = 0.99)								
**Total (95% CI)**			**200**			**196**	**100.0%**	**0.95 [-0.53, 2.43]**
Heterogeneity: Tau² = 5.01; Chi² = 41.82, df = 11 (P < 0.0001); I² = 74%								
Test for overall effect: Z = 1.26 (P = 0.21)								
Test for subgroup differences: Chi² = 1.14, df = 1 (P = 0.29), I² = 12.4%								

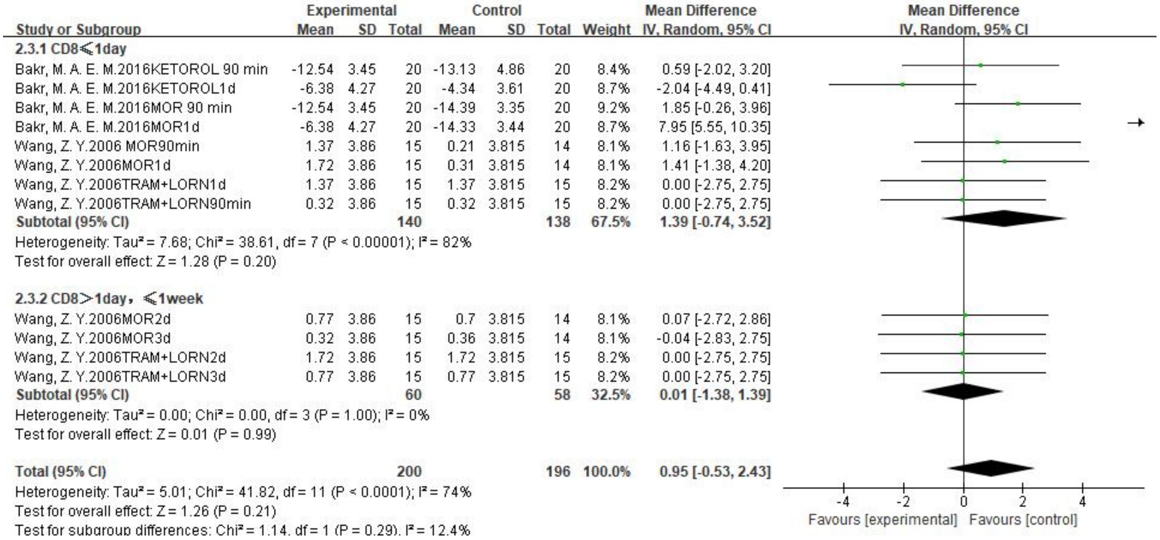

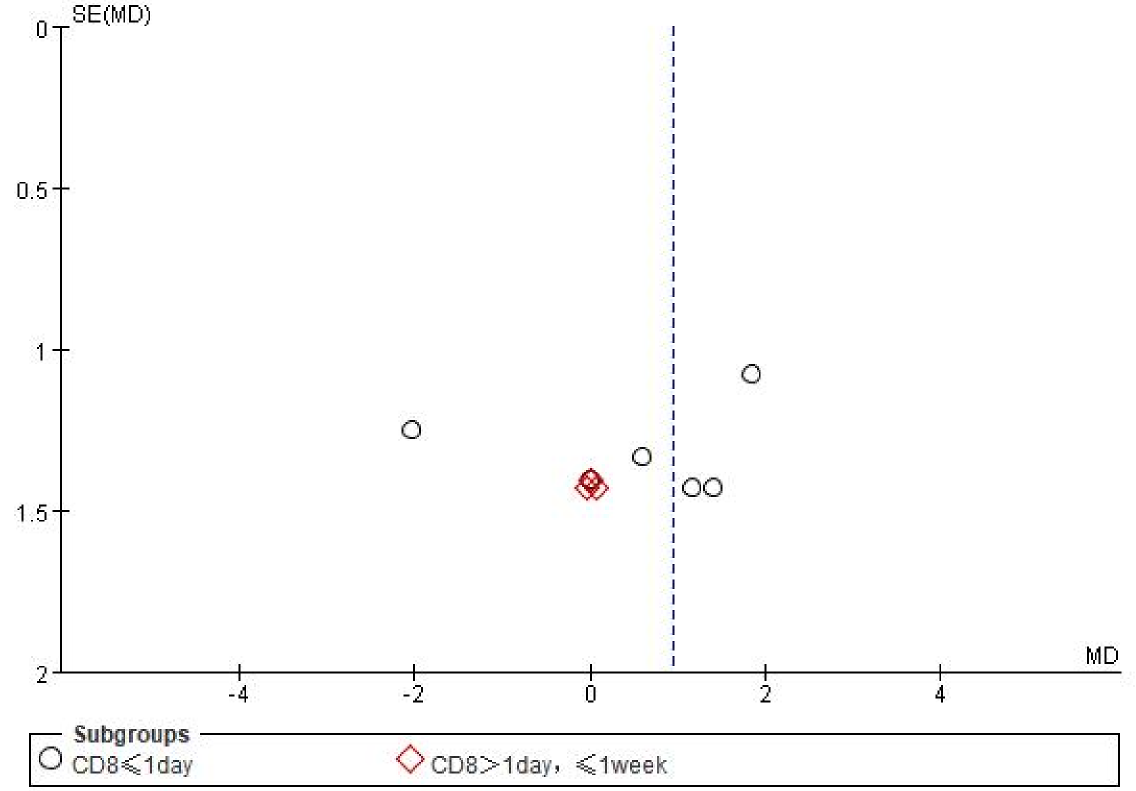

Supplement: Supplementary file 9 [file DataSheet9.pdf]
