# Supplementary material for: What are the beneficial treatment strategies in maintaining T lymphocyte subsets after cancer surgery? A systematic review and network meta-analysis
Source: Front Immunol. 2026 Jul 14;17:1854279. doi: 10.3389/fimmu.2026.1854279 (PMC13408238; doi:10.3389/fimmu.2026.1854279)
Supplement: Supplementary file 10 [file Table1.docx]

1. Zhang L, Ren Y, Liu Y. Comparison of the Effects of Lobectomy on Immunologic Function Between Video-Assisted Thoracoscopic Surgery and Traditional Open Surgery for Non-Small-Cell Lung Cancer. Am J Ther. 2016 Nov/Dec;23(6):e1406-e1413. doi: 10.1097/MJT.0000000000000254. PMID: 25909924.
2. Zhao G, Xiao G, Huang MX, Long HK. [Effect of laparoscopic radical operation on systemic immunity in patients with colorectal cancer]. Zhonghua Wei Chang Wai Ke Za Zhi. 2005 Sep;8(5):407-9. Chinese. PMID: 16224653.
3. Peng B, Zheng JH, Li H. Effect of retroperitoneal laparoscopic radical nephrectomy of renal carcinoma (nephroma) on perioperative cell immunity. J Endourol. 2008 Sep;22(9):2161-4. doi: 10.1089/end.2008.0174. PMID: 18811573.
4. Han, L, Cheng, H, Liu, J, Gao, W, Wang, H, Xie, H, Zhang, X & Song, Y 2018, 'Effect of video-assisted thoracoscopic surgery on immune function and trauma in patients with non-small cell lung cancer', International journal of clinical and experimental medicine, vol. 11, no. 11, pp. 12437-12444.
5. Zhang LB, Wang B, Wang XY, Zhang L. Influence of video-assisted thoracoscopic lobectomy on immunological functions in non-small cell lung cancer patients. Med Oncol. 2015 Jul;32(7):201. doi: 10.1007/s12032-015-0639-2. Epub 2015 Jun 17. PMID: 26081016.
6. Lv W, Ding B, Qian L, Wu W, Wen Y. Safety of Breast Cancer Mastoscopic Surgery from the Perspective of Immunity and Adipokines. J Invest Surg. 2022 Mar;35(3):632-638. doi: 10.1080/08941939.2021.1919945. Epub 2021 May 17. PMID: 33998356.
7. Zhao J, Niu N, He Z. Effect of Thymosin on Inflammatory Factor Levels, Immune Function, and Quality of Life in Lung Cancer Patients Undergoing Radical Thoracoscopic Surgery. Evid Based Complement Alternat Med. 2022 Jul 4;2022:8749999. doi: 10.1155/2022/8749999. Retraction in: Evid Based Complement Alternat Med. 2023 Dec 13;2023:9794761. doi: 10.1155/2023/9794761. PMID: 35832513; PMCID: PMC9273385.
8. Zhou Y, Huang J, Bai Y, Li C, Lu X. Effects of preemptive analgesia with flurbiprofen ester on lymphocytes and natural killer cells in patients undergoing esophagectomy: A randomized controlled pilot study. Thorac Cancer. 2017 Nov;8(6):649-654. doi: 10.1111/1759-7714.12502. Epub 2017 Sep 11. PMID: 28892265; PMCID: PMC5668486.
9. Shao, Ya-Jie & Liao, Zhi-Pin & Wu, Yan-Hui. (2018). Impact of postoperative analgesia with dezocine plus ropivacaine versus fentanyl plus ropivacaine on stress response and immune function in patients with gastric cancer. World Chinese Journal of Digestology. 26. 616-622. 10.11569/wcjd.v26.i10.616.
10. Gao Y, Kang M, Niu L, Xu L, Xie X, Chen D, Zhu L, Wang F. The effects of radiotherapy after thoracic and laparoscopic surgery on patients with esophageal cancer and on their prognoses. Am J Transl Res. 2021 Jun 15;13(6):6446-6456. PMID: 34306385; PMCID: PMC8290759.
11. Liu M, Wang H, Du S, Li W, Xuan F, Zhao Y, Li N. Laparoscopic Radical Hysterectomy Combined with Neoadjuvant Chemotherapy for Cervical Cancer Patients Effectively Improves Immune Function. Dis Markers. 2022 Sep 14;2022:3611174. doi: 10.1155/2022/3611174. Retraction in: Dis Markers. 2023 Jun 21;2023:9781835. doi: 10.1155/2023/9781835. PMID: 36157208; PMCID: PMC9492327.
12. Li, K. & Yang, J.. (2009). The effect of different nutrition on the immune function of patients with colorectal cancer. 9. 1288-1291.
13. Mi L, Zhong B, Zhang DL, Zhou YB, Wang DS. [Effect of early oral enteral nutrition on clinical outcomes after gastric cancer surgery]. Zhonghua Wei Chang Wai Ke Za Zhi. 2012 May;15(5):464-7. Chinese. PMID: 22648840.
14. Ding D, Feng Y, Song B, Gao S, Zhao J. Effects of preoperative and postoperative enteral nutrition on postoperative nutritional status and immune function of gastric cancer patients. Turk J Gastroenterol. 2015 Mar;26(2):181-5. doi: 10.5152/tjg.2015.3993. PMID: 25835119.
15. Marano L, Porfidia R, Pezzella M, Grassia M, Petrillo M, Esposito G, Braccio B, Gallo P, Boccardi V, Cosenza A, Izzo G, Di Martino N. Clinical and immunological impact of early postoperative enteral immunonutrition after total gastrectomy in gastric cancer patients: a prospective randomized study. Ann Surg Oncol. 2013 Nov;20(12):3912-8. doi: 10.1245/s10434-013-3088-1. Epub 2013 Jul 10. PMID: 23838912.
16. Liu H, Ling W, Shen ZY, Jin X, Cao H. Clinical application of immune-enhanced enteral nutrition in patients with advanced gastric cancer after total gastrectomy. J Dig Dis. 2012 Aug;13(8):401-6. doi: 10.1111/j.1751-2980.2012.00596.x. PMID: 22788925.
17. HUANG, Xunbo & YUAN, Shenyi & CHEN, Qiaofeng & SHEN, Dongjie & Li, X. & WANG, Tianxiang. (2010). The clinical significance of preoperative administration of enteral immunonutrition to patients with malignant gastrointestinal tumors. Pharmaceutical Care and Research. 10. 450-452. 10.5428/pcar20100616.
18. Liu, Z. & Yu, J.. (2011). Effect of immune enhanced enteral nutrition on postoperative immune function and inflammatory responses in gastric cancer patients with radical gastrectomy. 18. 66-67+69.
19. Chen, H. & Jia, J.-G & Li, F. & Yang, L. & Yang, P. & Sun, J.-B. (2006). Effects of early postoperative nutritional support on immune system and inflammatory responses after gastrointestinal surgey. Chinese Journal of Clinical Nutrition. 14. 144-148.
20. Wang, H.-X & Xia, Y. & Shao, S.-Y. (2011). Influence of enteral nutrition during the preoperative and postoperative periods on postoperative nutritional status and immunologic function in patients with gastric cancer. Journal of Xi'an Jiaotong University (Medical Sciences). 32. 375-378.
21. Zhao H, Zhao H, Wang Y, Jing H, Ding Q, Xue J. Randomized clinical trial of arginine-supplemented enteral nutrition versus standard enteral nutrition in patients undergoing gastric cancer surgery. J Cancer Res Clin Oncol. 2013 Sep;139(9):1465-70. doi: 10.1007/s00432-013-1466-5. Epub 2013 Jun 29. Retraction in: J Cancer Res Clin Oncol. 2015 Mar;141(3):573. doi: 10.1007/s00432-015-1913-6. PMID: 23812551; PMCID: PMC11824343.
22. Li B, Liu HY, Guo SH, Sun P, Gong FM, Jia BQ. The postoperative clinical outcomes and safety of early enteral nutrition in operated gastric cancer patients. J BUON. 2015 Mar-Apr;20(2):468-72. PMID: 26011337.
23. Sorensen D, McCarthy M, Baumgartner B, Demars S. Perioperative immunonutrition in head and neck cancer. Laryngoscope. 2009 Jul;119(7):1358-64. doi: 10.1002/lary.20494. PMID: 19459146.
24. Liang B, Wang S, Ye YJ, Yang XD, Wang YL, Qu J, Xie QW, Yin MJ. Impact of postoperative omega-3 fatty acid-supplemented parenteral nutrition on clinical outcomes and immunomodulations in colorectal cancer patients. World J Gastroenterol. 2008 Apr 21;14(15):2434-9. doi: 10.3748/wjg.14.2434. PMID: 18416476; PMCID: PMC2705104.
25. Ma BQ, Chen SY, Jiang ZB, Wu B, He Y, Wang XX, Li Y, Gao P, Yang XJ. Effect of postoperative early enteral nutrition on clinical outcomes and immune function of cholangiocarcinoma patients with malignant obstructive jaundice. World J Gastroenterol. 2020 Dec 14;26(46):7405-7415. doi: 10.3748/wjg.v26.i46.7405. PMID: 33362392; PMCID: PMC7739166.
26. Tang Jingshuang, Xu Wei, Effect of postoperative enteral and parenteral nutrition on the immunity and inflammatory reaction of esophageal cancer patients, Journal of Practical Oncology, Volume 34, Issue 2, 2019, Pages 155-159, ISSN 1001-1692, <https://doi.org/10.13267/j.cnki.syzlzz.2019.02.012.>
27. Yang J, Zhang X, Li K, Zhou Y, Hu Y, Chen X, Liang S, Jiang L. Effects of EN combined with PN enriched with n-3 polyunsaturated fatty acids on immune related indicators and early rehabilitation of patients with gastric cancer: A randomized controlled trial. Clin Nutr. 2022 Jun;41(6):1163-1170. doi: 10.1016/j.clnu.2022.03.018. Epub 2022 Apr 6. PMID: 35500316.
28. Ding H, Xu J, You J, Qin H, Ma H. Effects of enteral nutrition support combined with enhanced recovery after surgery on the nutritional status, immune function, and prognosis of patients with esophageal cancer after Ivor-Lewis operation. J Thorac Dis. 2020 Dec;12(12):7337-7345. doi: 10.21037/jtd-20-3410. PMID: 33447423; PMCID: PMC7797812.
29. Liu, H. & Ling, W. & Cao, H.. (2011). Effects of immune-enhanced enteral nutrition and parenteral nutrition on immune and nutritional function in elderly patients with gastric cancer after total gastrectomy. Journal of Shanghai Jiaotong University (Medical Science). 31. 1000-1004. 10.3969/j.issn.1674-8115.2011.07.029.
30. Zong L, Li H, Li S. Effects of neoadjuvant chemotherapy combined with enteral nutrition on perioperative immunity, inflammation and intestinal flora in gastric cancer patients. J BUON. 2019 May-Jun;24(3):1113-1119. PMID: 31424669.
31. Cai J, Wang H, Zhou S, Wu B, Song HR, Xuan ZR. [Effect of Sijunzi Decoction and enteral nutrition on T-cell subsets and nutritional status in patients with gastric cancer after operation: a randomized controlled trial]. Zhong Xi Yi Jie He Xue Bao. 2008 Jan;6(1):37-40. Chinese. doi: 10.3736/jcim20080108. PMID: 18184544.
32. Gu RM, Wen X, Wei D, Ming XZ, Li G, Chen HQ. [Effect of intraoperative intraperitoneal chemotherapy and postoperative nutritional support on intestinal permeability and cellular immune function in patients with advanced gastric cancer]. Zhonghua Wei Chang Wai Ke Za Zhi. 2012 May;15(5):468-72. Chinese. PMID: 22648841.
33. Wang, M.-Q & Zhou, J.. (2018). Effect of general anesthesia combined with thoracic paravertebral block on Postoperative Immunity and tumor markers in patients with primary liver cancer. Chinese Journal of Cancer Prevention and Treatment. 25. 1174-1177.
34. Xing R, Yang Y, Zhang M, Wang H, Tan M, Gao C, Yang C, Zhai M, Xie Y. Effect of Transcutaneous Electrical Acupoint Stimulation Combined with Transversus Abdominis Plane Block on Postoperative Recovery in Elderly Patients Undergoing Laparoscopic Gastric Cancer Surgery: A Randomized Controlled Trial. Pain Ther. 2022 Dec;11(4):1327-1339. doi: 10.1007/s40122-022-00429-2. Epub 2022 Sep 13. PMID: 36098938; PMCID: PMC9633915.
35. Woo JH, Baik HJ, Kim CH, Chung RK, Kim DY, Lee GY, Chun EH. Effect of Propofol and Desflurane on Immune Cell Populations in Breast Cancer Patients: A Randomized Trial. J Korean Med Sci. 2015 Oct;30(10):1503-8. doi: 10.3346/jkms.2015.30.10.1503. Epub 2015 Sep 12. PMID: 26425050; PMCID: PMC4575942.
36. Xin, Li & Dan, Yang & Yan, Chen & Jiang, Shen & Tao, Hong. (2019). Effect of two types of anesthesia on postoperative recovery of patients with gastric cancer and changes in the levels of their T lymphocyte subsets. Tropical Journal of Pharmaceutical Research. 18. 429. 10.4314/tjpr.v18i2.30.
37. Zhu J, Zhang XR, Yang H. Effects of combined epidural and general anesthesia on intraoperative hemodynamic responses, postoperative cellular immunity, and prognosis in patients with gallbladder cancer: A randomized controlled trial. Medicine (Baltimore). 2017 Mar;96(10):e6137. doi: 10.1097/MD.0000000000006137. PMID: 28272202; PMCID: PMC5348150.
38. Zhu R, Xiang J, Tan M. Effects of different anesthesia and analgesia on cellular immunity and cognitive function of patients after surgery for esophageal cancer. Minerva Chir. 2020 Dec;75(6):449-456. doi: 10.23736/S0026-4733.20.08283-8. Epub 2020 Aug 6. PMID: 32773737.
39. Zhang T, Fan Y, Liu K, Wang Y. Effects of different general anaesthetic techniques on immune responses in patients undergoing surgery for tongue cancer. Anaesth Intensive Care. 2014 Mar;42(2):220-7. doi: 10.1177/0310057X1404200209. PMID: 24580388.
40. Liu S, Gu X, Zhu L, Wu G, Zhou H, Song Y, Wu C. Effects of propofol and sevoflurane on perioperative immune response in patients undergoing laparoscopic radical hysterectomy for cervical cancer. Medicine (Baltimore). 2016 Dec;95(49):e5479. doi: 10.1097/MD.0000000000005479. PMID: 27930529; PMCID: PMC5266001.
41. Wang L, Liang S, Chen H, Xu Y, Wang Y. The effects of epidural anaesthesia and analgesia on T lymphocytes differentiation markers and cytokines in patients after gastric cancer resection. BMC Anesthesiol. 2019 Jun 12;19(1):102. doi: 10.1186/s12871-019-0778-7. PMID: 31185917; PMCID: PMC6560762.
42. Lin Y, Miao Z, Wu Y, Ge FF, Wen QP. Effect of low dose naloxone on the immune system function of a patient undergoing video-assisted thoracoscopic resection of lung cancer with sufentanil controlled analgesia - a randomized controlled trial. BMC Anesthesiol. 2019 Dec 19;19(1):236. doi: 10.1186/s12871-019-0912-6. PMID: 31856760; PMCID: PMC6923917.
43. Zong S, Du J, Chen Y, Tao H. Application effect of dexmedetomidine combined with flurbiprofen axetil and flurbiprofen axetil monotherapy in radical operation of lung cancer and evaluation of the immune function. J BUON. 2021 Jul-Aug;26(4):1432-1439. PMID: 34565001.
44. Ai Z. Effects of dexmedetomidine combined with dezocine on T lymphocytes, NK cells and cognitive function in elderly patients with gastrointestinal cancer after radical surgery. Cell Mol Biol (Noisy-le-grand). 2023 Oct 31;69(10):56-62. doi: 10.14715/cmb/2023.69.10.7. PMID: 37953585.
45. Wang K, Li C. Effects of dexmedetomidine on inflammatory factors, T lymphocyte subsets and expression of NF-κB in peripheral blood mononuclear cells in patients receiving radical surgery of colon carcinoma. Oncol Lett. 2018 May;15(5):7153-7157. doi: 10.3892/ol.2018.8205. Epub 2018 Mar 7. PMID: 29725437; PMCID: PMC5920235.
46. Bai, Y. B., Chen, B., Zhang, H. L., et al. (2020). Effects of oxycodone combined with dexmedetomidine on immune function of patients undergoing radical mastectomy with general anesthesia. Anti - Tumor Pharm, 10(4), 461 - 466.
47. Chen, M. & Han, B. & Yi, M. & Li, X.. (2022). Effects of parecoxib sodium on perioperative immune function and tumor micrometastasis in patients with lung cancer. Chinese Journal of Anatomy and Clinics. 27. 568-573. 10.3760/cma.j.cn101202-20220222-00059.
48. Gao, Y.-F & Yuan, W. & Ding, X.-Y & Huo, X.-W & Jing, G.-X & Lü, Y.. (2014). Effects of postoperative multimodal analgesia with dezocine and flurblprofen on cellular immune function in patients after radical gastric cancer surgery. Journal of Xi'an Jiaotong University (Medical Sciences). 35. 669-673 and 713. 10.7652/jdyxb201405020.
49. Li, W.-K & Wang, X.-X & Chen, Z.-P & Li, S.-S. (2008). Effects of sulfentanil and morphine on T-lymphocyte subsets during postoperative analgesia in malignant bone tumor radical correction patients. Chinese Journal of Cancer Prevention and Treatment. 15. 1261-1263.
50. Shen JC, Sun HL, Zhang MQ, Liu XY, Wang Z, Yang JJ. Flurbiprofen improves dysfunction of T-lymphocyte subsets and natural killer cells in cancer patients receiving post-operative morphine analgesia. Int J Clin Pharmacol Ther. 2014 Aug;52(8):669-75. doi: 10.5414/CP202027. PMID: 24755130.
51. Wang RD, Zhu JY, Zhu Y, Ge YS, Xu GL, Jia WD. Perioperative analgesia with parecoxib sodium improves postoperative pain and immune function in patients undergoing hepatectomy for hepatocellular carcinoma. J Eval Clin Pract. 2020 Jun;26(3):992-1000. doi: 10.1111/jep.13256. Epub 2019 Aug 12. PMID: 31407484.
52. Wang ZY, Wang CQ, Yang JJ, Sun J, Huang YH, Tang QF, Qian YN. Which has the least immunity depression during postoperative analgesia--morphine, tramadol, or tramadol with lornoxicam? Clin Chim Acta. 2006 Jul 15;369(1):40-5. doi: 10.1016/j.cca.2006.01.008. Epub 2006 Feb 17. PMID: 16487501.
53. Bakr MA, Amr SA, Mohamed SA, Hamed HB, Abd El-Rahman AM, Mostafa MA, El Sherif FA. Comparison Between the Effects of Intravenous Morphine, Tramadol, and Ketorolac on Stress and Immune Responses in Patients Undergoing Modified Radical Mastectomy. Clin J Pain. 2016 Oct;32(10):889-97. doi: 10.1097/AJP.0000000000000338. PMID: 26710216.
54. Li, A. G., Chen, Z. J., Jiang, Y. H., He, L., Zou, L. Q., & Huang, Z. J. (2018). The application of dexmedetomidine combined with ropivacaine in postoperative analgesia of colon cancer and its effect on patients' immune function. Anti - Tumor Pharmacy, 8(2), 179 - 183.
55. Wang, D., Yao, S. Z., Ju, F., & Li, M. L. (2018). The effect of thymosin α1 combined with interventional therapy on liver function and T cell subsets in patients with advanced liver cancer. Anti - Tumor Pharmacy, 8(3), 451 - 454+477.
56. Chen C, Duan XT, Li GY, Hao XJ, Wang WL, Shen YF, Zhang SH. Evaluation of the efficacy of aparatinib and carrilizumab combined with transcatheter arterial chemoembolization in the treatment of primary hepatocellular carcinoma. Eur Rev Med Pharmacol Sci. 2023 May;27(9):4135-4144. doi: 10.26355/eurrev_202305_32322. PMID: 37203839.
57. Li, X., Xu, P., Wang, C. B., Li, C., Huang, J. B., & Chen, W. X. (2020). Study on the clinical effect of sorafenib in patients with hepatocellular carcinoma and the imaging characteristics of diffusion - weighted imaging. Journal of Practical Medical Imaging, 21(2), 121 - 124.
58. Lin S, Zang M. Effects of Apatinib Mesylate Monotherapy on the Incidence of Adverse Reactions and Immune Function in Patients with Breast Cancer after Radical Mastectomy. Evid Based Complement Alternat Med. 2022 Aug 10;2022:4022282. doi: 10.1155/2022/4022282. Retraction in: Evid Based Complement Alternat Med. 2023 Dec 6;2023:9861238. doi: 10.1155/2023/9861238. PMID: 35990841; PMCID: PMC9385297.
59. Quan Y, Liu JG, Cai YC, Zhang JR. [Changes of immune function in liver cancer patients after transcatheter arterial chemoembolizaton combined with interstitial therapy]. Nan Fang Yi Ke Da Xue Xue Bao. 2009 Nov;29(11):2288-90. Chinese. PMID: 19923089.
60. Zhao, Yu-Shan & Wen, Shu-Wei & Chang, Jun-Ping & Zhang, Xiao-Qian & Dang, Zhi-Jun. (2016). Lienal polypeptide injection combined with transcatheter hepatic arterial chemoembolization for treating primary hepatic cancer: Curative effect and influence on cellular immune function. World Chinese Journal of Digestology. 24. 2384. 10.11569/wcjd.v24.i15.2384.
61. Li T, Li ZW, Wen HC. [Study on the efficacy and safety of high dose thymopentin combined with trans-artery chemoembolization for primary liver cancer]. Zhonghua Zhong Liu Za Zhi. 2007 Dec;29(12):941-2. Chinese. PMID: 18478937.
62. Yu H, Xu C, Li Q. Clinical Efficacy and Safety of Tumor Cytoreductive Surgery plus Hyperthermic Intraperitoneal Chemotherapy for Ovarian Cancer. Evid Based Complement Alternat Med. 2023 Apr 12;2023:6412679. doi: 10.1155/2023/6412679. Retraction in: Evid Based Complement Alternat Med. 2023 Dec 13;2023:9824296. doi: 10.1155/2023/9824296. PMID: 37089719; PMCID: PMC10115522.
63. Gu RM, Wen X, Wei D, Ming XZ, Li G, Chen HQ. [Effect of intraoperative intraperitoneal chemotherapy and postoperative nutritional support on intestinal permeability and cellular immune function in patients with advanced gastric cancer]. Zhonghua Wei Chang Wai Ke Za Zhi. 2012 May;15(5):468-72. Chinese. PMID: 22648841.
64. Nie Z, Huang Y, Li S, Li L. Effect of operation-introducing hyperthermic intraperitoneal chemotherapy on cellular immune function in patients with adenocarcinoma of the esophagogastric junction. Asian J Surg. 2022 Jan;45(1):559-560. doi: 10.1016/j.asjsur.2021.09.028. Epub 2021 Oct 20. PMID: 34656412.
65. Zhou L, Zhang T, Sun Y, Fan R, Xu L, Yue S, Yao R. Effect of preoperative infusion chemotherapy combined with hyperthermia on sPD-L1 and CEA levels and overall survival of elderly patients undergoing radical resection of lung cancer. J BUON. 2019 Mar-Apr;24(2):572-577. PMID: 31128008.
66. Zhang J, Song C, Liu B. Efficacy of cisplatin plus paclitaxel as chemotherapy in patients with cervical cancer after laparoscopic nerve-sparing extensive hysterectomy and its effect on immune function. Pak J Pharm Sci. 2022 Jan;35(1(Special)):355-359. PMID: 35236647.
67. Cesana GC, Romano F, Piacentini G, Scotti M, Brenna A, Bovo G, Vaghi M, Aletti G, Caprotti R, Kaufman H, Uggeri F. Low-dose interleukin-2 administered pre-operatively to patients with gastric cancer activates peripheral and peritumoral lymphocytes but does not affect prognosis. Ann Surg Oncol. 2007 Apr;14(4):1295-304. doi: 10.1245/s10434-006-9239-x. Epub 2007 Jan 17. PMID: 17225981.
68. Dai CM, Jin S, Zhang JZ. [Effect of Dahuang Zhechong Pills combined with TACE on VEGF, MMP-2, TGF-β1 and immune function of patients with primary liver cancer (blood stasis and collaterals blocking type)]. Zhongguo Zhong Yao Za Zhi. 2021 Feb;46(3):722-729. Chinese. doi: 10.19540/j.cnki.cjcmm.20200716.501. PMID: 33645040.
69. Wu, B. & Xuan, Z.-R & Cai, J. & Song, H.-R & Wang, H. & Yang, H.-B. (2007). Effect of early perioperative enteral nutrition support with rhubarb and Sijunzi decoction on immune function in malnourished surgical patients with gastric cancer. 15. 295-299.
70. Wang JY, Ma GW, Dai SQ, Rong TH, Wang X, Lin P, Ye WF, Zhang LJ, Li XD, Zhang X, Yao GY. [Effect of cellular immune supportive treatment on immunity of esophageal carcinoma patients after modern two-field lymph node dissection]. Ai Zheng. 2007 Jul;26(7):778-81. Chinese. PMID: 17626759.
71. Zhao, S. X., Liu, H. L., Fan, Z., Jin, Z. Z., & Wang, J. J. (2017). The effect of Yupingfeng Powder adjuvant therapy on the curative effect of primary liver cancer and anti-tumor immunity. Anti - Tumor Pharmacy, 7(6).
72. Cai J, Wang H, Zhou S, Wu B, Song HR, Xuan ZR. [Effect of Sijunzi Decoction and enteral nutrition on T-cell subsets and nutritional status in patients with gastric cancer after operation: a randomized controlled trial]. Zhong Xi Yi Jie He Xue Bao. 2008 Jan;6(1):37-40. Chinese. doi: 10.3736/jcim20080108. PMID: 18184544.
73. Huang, Z. R., Chen, Y. M., Lin, H., Lin, J. P., & Chen, N. J. (2019). The regulatory effect of Zilongjin Tablets on immune function of patients with lung cancer after operation. Chinese Traditional and Herbal Drugs, 50(12), 2941 - 2944.
74. Zhao X, Cui L, Wang W, Su Q, Li X, Wu J. Influence of psychological intervention on pain and immune functions of patients receiving lung cancer surgery. Pak J Med Sci. 2016 Jan-Feb;32(1):155-9. doi: 10.12669/pjms.321.8935. PMID: 27022366; PMCID: PMC4794516.
75. Lengacher CA, Kip KE, Post-White J, Fitzgerald S, Newton C, Barta M, Jacobsen PB, Shelton MM, Moscoso M, Johnson-Mallard V, Harris E, Loftus L, Cox C, Le N, Goodman M, Djeu J, Widen RH, Bercu BB, Klein TW. Lymphocyte recovery after breast cancer treatment and mindfulness-based stress reduction (MBSR) therapy. Biol Res Nurs. 2013 Jan;15(1):37-47. doi: 10.1177/1099800411419245. Epub 2011 Nov 14. PMID: 22084404.
76. Chen L, Sun L, Lang Y, Wu J, Yao L, Ning J, Zhang J, Xu S. Fast-track surgery improves postoperative clinical recovery and cellular and humoral immunity after esophagectomy for esophageal cancer. BMC Cancer. 2016 Jul 11;16:449. doi: 10.1186/s12885-016-2506-8. PMID: 27401305; PMCID: PMC4940721.
77. Zhao, X. (2020). Effect of hypothermia prevention in patients undergoing gastrointestinal cancer surgery. International Journal of Clinical and Experimental Medicine, 13(10), 7756 - 7761.
78. Zhao JL, Nie YQ, Yang P, Jiang DZ, Zhang FW. Effect of selective lymph node dissection on immune function in patients with T1 stage non-small cell lung cancer: a randomized controlled trial. Transl Cancer Res. 2021 Jun;10(6):2918-2931. doi: 10.21037/tcr-21-524. PMID: 35116601; PMCID: PMC8797331.
79. Chen, X.-Y & Zhang, N.-C & Yin, Z.-X & Li, C.-Q & Chen, Z.-Y & Zhu, L.-G. (2017). Regulatory effect of mannanglcopeptide on postoperative immune function in patients with malignant thoracic tumor. Chinese Journal of New Drugs. 26. 1818-1821.
80. Zhu D, Chen X, Wu J, Ju Y, Feng J, Lu G, Ouyang M, Ren B, Li Y. [Effect of perioperative intestinal probiotics on intestinal flora and immune function in patients with colorectal cancer]. Nan Fang Yi Ke Da Xue Xue Bao. 2012 Aug;32(8):1190-3. Chinese. PMID: 22931620.
81. Zhang L, Wang N, Zhou S, Ye W, Yao Q, Jing G, Zhang M. Preventive effect of ulinastatin on postoperative complications, immunosuppression, and recurrence in esophagectomy patients. World J Surg Oncol. 2013 Apr 10;11:84. doi: 10.1186/1477-7819-11-84. PMID: 23575450; PMCID: PMC3626858.
82. Li Y, Yang GL, Yuan HY, Bai DJ, Wang K, Lin CR, Hu MB, Feng MH. Effects of perioperative cimetidine administration on peripheral blood lymphocytes and tumor infiltrating lymphocytes in patients with gastrointestinal cancer: results of a randomized controlled clinical trial. Hepatogastroenterology. 2005 Mar-Apr;52(62):504-8. PMID: 15816467.
