# Supplementary material for: What are the beneficial treatment strategies in maintaining T lymphocyte subsets after cancer surgery? A systematic review and network meta-analysis
Source: Front Immunol. 2026 Jul 14;17:1854279. doi: 10.3389/fimmu.2026.1854279 (PMC13408238; doi:10.3389/fimmu.2026.1854279)
Supplement: Supplementary file 11 [file Table2.docx]

| **PubMed (1414)** | **Results** |
| --- | --- |
| Radiotherapy：  (((((((((radiotherapy[MeSH Terms]) OR (Radiotherapies[Title/Abstract])) OR (Radiation Therapy[Title/Abstract])) OR (Radiation Therapies[Title/Abstract])) OR (Radiation Treatment[Title/Abstract])) OR (Radiation Treatments[Title/Abstract])) OR (Targeted Radiotherapies[Title/Abstract])) OR (Targeted Radiotherapy[Title/Abstract])) OR (Targeted Radiation Therapy[Title/Abstract])) AND ((((((((T lymphocyte subsets[MeSH Terms]) OR (T Lymphocyte Subset[Title/Abstract])) OR (T Cell Subsets[Title/Abstract])) OR (T-Cell Subset[Title/Abstract])) OR (CD3+T[Title/Abstract])) OR (CD4+T[Title/Abstract])) OR (CD8+T[Title/Abstract])) OR (Tregs[Title/Abstract]))  Chemotherapy：  ((chemotherapy[MeSH Terms]) OR (Chemotherapies[Title/Abstract])) AND ((((((((T lymphocyte subsets[MeSH Terms]) OR (T Lymphocyte Subset[Title/Abstract])) OR (T Cell Subsets[Title/Abstract])) OR (T-Cell Subset[Title/Abstract])) OR (CD3+T[Title/Abstract])) OR (CD4+T[Title/Abstract])) OR (CD8+T[Title/Abstract])) OR (Tregs[Title/Abstract]))  Surgery：  ((((((((T lymphocyte subsets[MeSH Terms]) OR (T Lymphocyte Subset[Title/Abstract])) OR (T Cell Subsets[Title/Abstract])) OR (T-Cell Subset[Title/Abstract])) OR (CD3+T[Title/Abstract])) OR (CD4+T[Title/Abstract])) OR (CD8+T[Title/Abstract])) OR (Tregs[Title/Abstract])) AND (((((((Surgery[MeSH Terms]) OR (Operative Procedures[Title/Abstract])) OR (Operative Procedure[Title/Abstract])) OR (Operative Surgical Procedures[Title/Abstract])) OR (Surgical Procedures[Title/Abstract])) OR (Surgical Procedure[Title/Abstract])) OR (Operative Surgical Procedure[Title/Abstract]))  Recurrence：  ((((((recurrence[MeSH Terms]) OR (Recurrences[Title/Abstract])) OR (Recrudescence[Title/Abstract])) OR (Recrudescences[Title/Abstract])) OR (Relapse[Title/Abstract])) OR (Relapses[Title/Abstract])) AND ((((((((T lymphocyte subsets[MeSH Terms]) OR (T Lymphocyte Subset[Title/Abstract])) OR (T Cell Subsets[Title/Abstract])) OR (T-Cell Subset[Title/Abstract])) OR (CD3+T[Title/Abstract])) OR (CD4+T[Title/Abstract])) OR (CD8+T[Title/Abstract])) OR (Tregs[Title/Abstract]))  Molecular Therap：  ((((((((T lymphocyte subsets[MeSH Terms]) OR (T Lymphocyte Subset[Title/Abstract])) OR (T Cell Subsets[Title/Abstract])) OR (T-Cell Subset[Title/Abstract])) OR (CD3+T[Title/Abstract])) OR (CD4+T[Title/Abstract])) OR (CD8+T[Title/Abstract])) OR (Tregs[Title/Abstract])) AND (((targeted therapy[MeSH Terms]) OR (Molecular Targeted Therapies[Title/Abstract])) OR (Targeted Molecular Therapy[Title/Abstract])) | 1414 |
| **Embase (2629)** | **Results** |
| #1.‘radiotherapy’ OR ‘Radiotherapies' OR 'Radiation Therapy' OR 'Radiation Therapies' OR 'Radiation Treatment' OR 'Radiation Treatments' OR 'Targeted Radiotherapies' OR 'Targeted Radiotherapy' OR 'Targeted Radiation Therapy’ | 2629 |
| #2.‘Chemotherapy’/exp OR ‘Chemotherapies’ |  |
| #3. ’Surgery’/exp OR ‘Operative Procedures’ OR ‘Operative Procedure’ OR ‘Operative Surgical Procedures’ OR ‘Surgical Procedures’ OR ‘Surgical Procedure’ OR ‘Operative Surgical Procedure’ |  |
| #4.‘recurrence’/exp OR ‘Recurrences’ OR ‘Recrudescence’ OR ‘Recrudescences’ OR ‘Relapse’ OR ‘Relapses’ |  |
| #5. ‘targeted therapy’/exp OR ‘Molecular Targeted Therapies’ OR ‘Targeted Molecular Therapy’ |  |
| #6.‘T lymphocyte subsets’/exp OR ‘T Lymphocyte Subset’ OR ‘T Cell Subsets’ OR ‘T-Cell Subset’ OR 'CD3+T' OR 'CD4+T' OR 'CD8+T’ OR ‘Tregs' |  |
| #7. #1 AND #6 |  |
| #8. #2 AND #6 |  |
| #9. #3 AND #6 |  |
| #10. #4 AND #6 |  |
| #11. #5 AND #6 |  |
| **Cochrane Library (1240)** | **Results** |
| #1：MeSH descriptor: [Radiotherapy] explode all trees | 1240 |
| #2：Radiotherapies or Radiation Therapy or Radiation Therapies or Radiation Treatment or Radiation Treatments or Targeted Radiotherapies or Targeted Radiotherapy or Targeted Radiation Therapy |  |
| #3：#1 or #2 |  |
| #4：MeSH descriptor: [T-Lymphocyte Subsets] explode all trees |  |
| #5：T Lymphocyte Subset or T Cell Subsets or T Cell Subset or CD3T or CD4T or CD8T or Tregs |  |
| #6：#4 or #5 |  |
| #7：MeSH descriptor: [Drug Therapy] explode all trees |  |
| #8：chemotherapy or Chemotherapies |  |
| #9：#7 or #8 |  |
| #10：MeSH descriptor: [General Surgery] explode all trees |  |
| #11：Operative Procedures or Operative Procedure or Operative Surgical Procedures or Surgical Procedures or Surgical Procedure or Operative Surgical Procedure |  |
| #12：#10 or #11 |  |
| #13：MeSH descriptor: [Recurrence] explode all trees |  |
| #14：Recurrences or Recrudescence or Recrudescences or Relapse or Relapses |  |
| #15：#13 or #14 |  |
| #16：MeSH descriptor: [Molecular Targeted Therapy] explode all trees |  |
| #17：Molecular Targeted Therapies or Targeted Molecular Therapy |  |
| #18：#16 or #17 |  |
| #19：randomized controlled trial |  |
| #20：#3 and #6 and #19 |  |
| #21：#9 and #6 and #19 |  |
| #22：#12 and #6 and #19 |  |
| #23：#15 and #6 and #19 |  |
| #24：#18 and #6 and #19 |  |
