# Supplementary material for: What are the beneficial treatment strategies in maintaining T lymphocyte subsets after cancer surgery? A systematic review and network meta-analysis
Source: Front Immunol. 2026 Jul 14;17:1854279. doi: 10.3389/fimmu.2026.1854279 (PMC13408238; doi:10.3389/fimmu.2026.1854279)
Supplement: Supplementary file 13 [file Table4.docx]

Anesthesia

Analgesia

Nutrition

SACT

Others
