# Supplementary material for: What are the beneficial treatment strategies in maintaining T lymphocyte subsets after cancer surgery? A systematic review and network meta-analysis
Source: Front Immunol. 2026 Jul 14;17:1854279. doi: 10.3389/fimmu.2026.1854279 (PMC13408238; doi:10.3389/fimmu.2026.1854279)
Supplement: Supplementary file 14 [file Table5.docx]

# Table S4 NOS scale for retrospective study

|  | Selection |  |  |  | Comparability | Exposure |  |  |  |
| --- | --- | --- | --- | --- | --- | --- | --- | --- | --- |
|  | Representativeness of the exposed cohort | Selection of the non-exposed cohort | Ascertainment of exposure | Definition of Controls | Comparability of cohorts on the basis of the design or analysis | Ascertainment of exposure | Same method of ascertainment for cases and controls | Non-Response rate | Total |
| Peng, B.2008 | 1 | 1 | 1 | 1 | 1 | 1 | 1 | 0 | 7 |
| Han, L.2018 | 1 | 1 | 1 | 1 | 1 | 1 | 0 | 0 | 6 |
| Zhang,L.B.2015 | 1 | 1 | 1 | 1 | 1 | 0 | 1 | 1 | 7 |
| Gu, R. M.2012 | 1 | 1 | 1 | 0 | 1 | 1 | 1 | 1 | 7 |
| Xin, L.2019 | 1 | 1 | 1 | 1 | 1 | 1 | 1 | 1 | 8 |
| Zhu, R.2020 | 1 | 1 | 1 | 1 | 0 | 1 | 1 | 1 | 7 |
| Li, X.2019 | 1 | 1 | 1 | 0 | 1 | 1 | 1 | 1 | 7 |
| Quan, Y.2009 | 1 | 1 | 1 | 1 | 1 | 0 | 1 | 1 | 7 |
| Gu, R. M.2012 | 1 | 1 | 1 | 1 | 1 | 1 | 1 | 1 | 8 |

NOS, Newcastle Ottawa Scale
