# Supplementary material for: What are the beneficial treatment strategies in maintaining T lymphocyte subsets after cancer surgery? A systematic review and network meta-analysis
Source: Front Immunol. 2026 Jul 14;17:1854279. doi: 10.3389/fimmu.2026.1854279 (PMC13408238; doi:10.3389/fimmu.2026.1854279)
Supplement: Supplementary file 14 [file Table3.docx]

Title	Trial phase	Author	Year	Test Number	Sex	Age	Total number of people	Tumor type	Experimental Group	The number of people in the experimental group	Data of T lymphocyte subsets in the experimental group	Control group	Number of people in the control group
Comparison of the Effect of Lobectomy on Immunologic Function Between Video-Assisted Thoracoscopic Surgery and Traditional Open Surgery for Non-Small-Cell Lung Cancer	/	Zhang, L.	2016	/	VATS Group: 26 men, 14 women<br>TOS Group: 31 men, 9 women	VATS group: 58.4±9.2 years old<br>TOS Group: 57.7±9.6 years old	80	Non-small cell lung cancer: Stage I<br>VATS group: 28 cases<br>TOS group: 28 cases<br>Phase II<br>VATS group: 3 cases<br>TOS group: 8 cases<br>Phase III<br>VATS group: 9 cases<br>TOS Group: 4 cases	Video-assisted thoracoscopic surgery	40	Proportion of CD4+ T cells: 33.35 ± 6.47%<br>Proportion of CD8+ T cells: 23.25 ± 7.45%<br>Proportion of NK cells: 18.97 ± 7.89%<br>Total number of lymphocytes: 1.71 ± 0.84 × 10^9/L<br>Three days after the operation<br>Proportion of CD4+ T cells: 36.51 ± 7.45%<br>Proportion of CD8+ T cells: 25.34 ± 8.12%<br>Proportion of NK cells: 13.31 ± 6.84%<br>Total number of lymphocytes: 1.01 ± 0.34 × 10^9/L<br>Seven days after the operation<br>Proportion of CD4+ T cells: 36.89 ± 8.15%<br>Proportion of CD8+ T cells: 22.01 ± 8.64%<br>Proportion of NK cells: 11.89 ± 5.12%<br>Total number of lymphocytes: 1.11 ± 0.47 × 10^9/L	Traditional open surgery	40
Effect of laparoscopic radical operation on systemic immunity in patients with colorectal cancer	/	Zhao, L.	2015	/	Male 34, female 26	Experimental group: 57.1±12.5 years old<br>Control group: 57.9±10.6 years old	60	Colorectal cancer, no staging	Laparoscopic surgery	30	Laparoscopic surgery group (30 cases) CD3(%) Preoperative: 38.4±0.21 postoperative day 3: 0.97±0.2 Spostoperative day 7: 1.83±0.31 CD4(%) Preoperative: 0.62±0.2 Spostoperative day 3: 0.29±0.14 postoperative day 7: 0.60±0.18 CD8(%) Preoperative: 0.17±0.12 postoperative day 3: 0.30±0.09 postoperative day 7: 0.37±0.18 CD4/CD8 preoperative: 1.70±0.31 postoperativeday 3: 1.63±0.48 postoperative day 7: 1.83±0.31	Traditional open surgery	30
Effect of retroperitoneal laparoscopic radical nephrectomy of renal carcinoma (nephrectomy) on perioperative cell immunity	/	Peng, B.	2008	/	Laparoscopy group (LN group): 14 men and 12 women<br>Open Surgery Group (ON Group): 15 men and 11 women	Laparoscopic group (LN group): The age range was 25 to 78 years old, with an average age of 58.67 ± 11.48 years old<br>Open surgery group (ON group): The age range was 27 to 82 years, with an average age of 52.53 ± 15.12 years	52	Renal cancer: Laparoscopic group (LN group): The tumor diameters ranged from 1.5 to 5.5 cm, with an average diameter of 4.2 cm<br>Open surgery group (ON group): The tumor diameters ranged from 1.5 to 6 centimeters, with an average diameter of 4.5 centimeters.	Retroperitoneal laparoscopic radical nephrectomy group (LN group)	26	Preoperative:<br>CD3 level: 70.81 ± 9.33<br>CD4 level: 44.73 ± 7.87<br>CD8 level: 22.92 ± 6.23<br>Postoperative<br>One day<br>CD3 level: 67.82 ± 10.82<br>CD4 level: 41.20 ± 8.22<br>CD8 level: 23.01 ± 6.73<br>Three days<br>CD3 level: 65.71 ± 11.21<br>CD4 level: 41.42 ± 9.68<br>CD8 level: 23.04 ± 8.33<br>Five days<br>CD3 level: 69.65 ± 10.17<br>CD4 level: 47.02 ± 9.27<br>CD8 level: 21.42 ± 5.92	Open radical nephrectomy group (ON group)	26
Effect of video-assisted thoracoscopic surgery on immune activation and trauma in patients with non-small cell lung cancer	/	Han, L.	2019	/	Experimental group (minimally invasive thoracoscopic surgery group): 50 males and 25 females<br>Control group (traditional thoracotomy group): 52 males and 23 females	Experimental group: 61.42±5.82 years old<br>Control group: 62.35±4.78 years old	150	Non-small cell lung cancer: Stage I and stage II, with specific proportions of 66.67% and 33.33% respectively. Lymph node metastasis: The majority of patients have lymph node metastasis, with a specific proportion of 62.67%.<br>Tumor size: The diameters of the tumors are mainly concentrated around 3 centimeters, with an average diameter of approximately 3.25 centimeters.	Minimally invasive thoracoscopic surgery group	[illegible]	Experimental Group:<br>CD4<br>Before treatment: 36.81<br>After treatment: 35.19<br>CD8<br>Before treatment: 28.37<br>After treatment: 25.61	Traditional thoracotomy group	75
Influence of video-assisted thoracoscopic lobectomy on immunological functions in non-small cell lung cancer patients	/	Zhang, L. B.	2015	/	Male: 68 (55.37%)<br>Female: 54 (45.53%)	Endoscopic group (VATS): Average age 58.8 ± 5.7 years (range: 38-69 years)<br>Open Group (TOS): Average age 56.7 ± 4.8 years (range: 33-73 years)	122	Non-small cell lung cancer: Endoscopic group (VATS): 20 cases were at stage Ia, 24 cases at stage Ib, and 17 cases at stage IIa, all of postoperative pathological staging.<br>Open group (TOS): 23 cases were at stage Ia, 20 cases at stage Ib, and 18 cases at stage IIa, all of postoperative pathological staging.	Surgical incision:<br>Endoscopic aperture: The 7th to 8th intercostal space along the mid-axillary line.<br>The main incision is located at the 4th to 5th intercostal space along the mid-axillary line, approximately 3 to 5 cm in length.<br>Assistant holes: 7-8 intercostal space along the scapular line, 1-2.	61	Preoperative:<br>CD3+ T cells: 52.6 ± 6.9 %<br>CD4+ T cells: 31.5 ± 6.8 %<br>CD8+ T cells: 28.2 ± 7.1 %<br>CD4+/CD8+ ratio: 1.1 ± 0.1<br>Day 1 after the operation<br>CD3+ T cells: 35.1 ± 3.4 %<br>CD4+ T cells: 22.4 ± 4.1 %<br>CD8+ T cells: 21.5 ± 3.5 %<br>CD4+/CD8+ ratio: 1.0 ± 0.1 *<br>Day 3 after the operation<br>CD3+ T cells: 44.2 ± 4.7 *<br>CD4+ T cells: 28.1 ± 4.6 *<br>CD8+ T cells: 25.3 ± 4.6 *<br>CD4+/CD8+ ratio: 1.1 ± 0.1<br>Day 5 after the operation<br>CD3+ T cells: 49.6 ± 5.9 *,&<br>CD4+ T cells: 32.5 ± 5.7 *,&<br>CD8+ T cells: 27.1 ± 5.8<br>CD4+/CD8+ ratio: 1.2 ± 0.2	Surgical incision: The 5th to 6th intercostal space along the mid-axillary line, approximately 15 to 20 cm in length.	61
Safety of Breast Cancer Macroscopic Surgery from the Perspective of Immunity and Adipokines	/	Li, W.	2022	ChiCTR-2000-035-918	female	Open surgery group: The average age was 48.29 years (standard deviation 5.52).<br>Endoscopic surgery group: The average age was 51.06 years (standard deviation 10.38)	42	Breast Cancer: Open Surgery Group:<br>Grade I: 3 cases (14.3%)<br>Grade II: 13 cases (61.9%)<br>Grade III: 5 cases (23.8%)<br>Endoscopic Surgery Group:<br>Grade I: 6 cases (28.6%)<br>Grade II: 11 cases (52.4%)<br>Grade III: 4 cases (19.0%)	Establish the operating space: inject swelling fluid, perform liposuction, and insert Trocar.<br>Surgery: Lymph node biopsy under laparoscopy and tumor resection.	21	"CD4+ T cells:<br>Preoperative: 48.54 ± 7.87%<br>One hour after the operation: 38.87 ± 9.51%<br>One day after the operation: 35.49 ± 9.19%<br>Five days after the operation: 44.19 ± 8.59%<br>Seven days after the operation: 43.54 ± 10.30%<br>CD8+ T cells:<br>Preoperative: 22.87 ± 7.73%<br>One hour after the operation: 17.57 ± 6.69%<br>One day after the operation: 21.03 ± 6.62%<br>Five days after the operation: 21.78 ± 7.34%<br>7 days after the operation: 21.80 ± 6.47%"	Open surgery	21
Effect of Thymosin on Inflammatory Factor Levels, Immune Function, and Quality of Life in Lung Cancer Patients Undergoing Radical Thoracoscopic Surgery	Phase II	Zhao, J.	2022	/	Experimental group: 51 males and 9 females<br>Control group: 48 males and 12 females	Experimental group: Average age 64.19 ± 12.68 years old<br>Control group: Average age 62.24 ± 11.53 years old	120	Experimental Group:<br>Squamous cell carcinoma: 32 cases<br>Adenocarcinoma: 19 cases<br>Alveolar cell carcinoma: 24 cases<br>Other types: 5 cases<br>Control group:<br>Squamous cell carcinoma: 30 cases<br>Adenocarcinoma: 22 cases<br>Alveolar cell carcinoma: 21 cases<br>Other types: 7 cases<br>Tumor staging<br>Experimental Group<br>Stage Ia: 18 cases<br>Stage Ib: 30 cases<br>StageIIa: 12 cases<br>Control group<br>Stage Ia: 20 cases<br>Stage Ib: 29 cases<br>StageIIa: 11 cases	On the day of the operation, thymosin was administered subcutaneously at a dose of 10-20 mg once a day. A course of treatment lasted for 21 days, with a total of 2 courses.	60	"Experimental Group<br>Preoperative<br>Percentage of CD4+ T cells: 37.21 ± 9.26%<br>Percentage of CD8+ T cells: 31.74 ± 9.57%<br>The ratio of CD4+ to CD8+: 1.21 ± 0.29<br>Percentage of NK cells: 24.52 ± 4.45%<br>Postoperative<br>Percentage of CD4+ T cells: 34.18 ± 7.29%*<br>Percentage of CD8+ T cells: 28.58 ± 7.26%*<br>CD4+/CD8+ ratio: 1.11 ± 0.32*<br>Percentage of NK cells: 21.71 ± 4.18%	Only thoracoscopic radical resection of lung cancer was adopted	60
Effects of pre-emptive analgesia with flurbiprofen axetil on lymphocytes and natural killer cells in patients undergoing esophagectomy: A randomized controlled pilot study	/	Zhou, Y.	2017	/	[illegible]	The age range is from 46 to 78, with an average age of approximately 68	75	Esophageal cancer, the specific type of tumor was not mentioned.<br>The tumor stage of the subject was T2N2M0 or below.	Flurbiprofen group (group 3):<br>The patient underwent thoracoscopic and laparoscopic surgeries for radical resection of esophageal cancer, and was given 1 mg/kg of flurbiprofen axetil for pre-emptive analgesia 30 minutes before the operation.<br>The surgical procedures were the same as those of the thoracoscopy group.	25	Flurbiprofen group (group 3):<br>CD3+ cells:<br>T0(30 minutes before surgery): 63.37 ± 8.86%<br>T1 (Completion of thoracic surgery): 64.04 ± 10.87%<br>T2 (at the end of surgery): 59.48 ± 12.42%<br>CD4+ cells:<br>T0(30 minutes before surgery): 31.29 ± 9.79%<br>T1 (end of thoracic surgery): 32.84 ± 12.64%<br>T2 (end of surgery): 33.27 ± 13.36%<br>CD8+ cells:<br>T0(30 minutes before surgery): 27.88 ± 9.33%<br>T1 (end of thoracic surgery): 26.32 ± 9.48%<br>T2 (end of surgery): 21.27 ± 8.34%<br>CD4+/CD8+ ratio<br>T0 (30 minutes before surgery): 1.31 ± 0.37<br>T1 (end of thoracic surgery): 1.39 ± 0.73<br>T2 (End of surgery): 1.85 ± 1.25"	"Open-chest group (group 1):<br>The patient underwent thoracotomy for radical resection of esophageal cancer.<br>The patient was placed in a left lateral position. The esophagus was separated through a posterolateral incision on the right chest, and samples of thoracic lymph nodes were taken.<br>Then the patient was placed in a supine position. The esophagus in the neck was separated and the abdominal cavity was opened to separate the stomach and take lymph nodes to create a tubular stomach.<br>Finally, end-to-end anastomosis of the cervical and esophageal ends was performed.	25/25
Impact of postoperative analgesia with dexmedetomidine, ropivacaine versus fentanyl plus ropivacaine on stress response and immune function in patients with gastric cancer	/	Shen, Y. J.	2018	/	There were 61 male cases and 27 female cases.	53.3 years ± 4.5 years, ranging from 41 to 62 years old	88	Gastric cancer, ASA grade I or II	After the operation, an epidural analgesic pump was connected for epidural analgesia. The ratio of the liquid medicine in the analgesic pump was: dexmedetomidine 30 mg + 0.75% ropivacaine 20 mL + 100 mL 0.9% sodium chloride injection. Fluid-free metering, single press metering of 4 mL, locking time of 5 minutes.	44	"Observation Group"<br>NK cells before surgery: 25.84 6.68<br>Preoperative CD3(%): 67.22 8.93<br>Preoperative CD4(%): 36.74 9.17<br>Preoperative CD8(%): 26.63 7.83<br>NK cells 24 hours after the operation: 20.82 8.23<br>CD3(%) 24 hours after the operation: 54.68 8.33<br>24-hour postoperative CD4(%): 28.83 9.12<br>24-hour postoperative CD8(%): 27.42 8.57"	Both groups of patients strictly completed the operation in accordance with the operation norms of laparoscopic radical gastrectomy for gastric cancer. After the operation, an epidural analgesic pump was connected for epidural analgesia. The ratio of the liquid medicine in the analgesic pump was: 8 μg fentanyl + 0.75% ropivacaine 20 mL + 100 mL 0.9% sodium chloride injection. Fluid-free metering, single press metering of 4 mL, locking time of 5 minutes.	44
The effects of radiotherapy after thoracic and laparoscopic surgery on patients with esophageal cancer and on their prognosis	/	Guo, Y.	2021	/	Postoperative chemotherapy group: 36 males and 23 females.<br>Postoperative radiotherapy group: 31 males and 28 females	Postoperative chemotherapy group: Average age (45.13±10.8) years.<br>Postoperative radiotherapy group: Average age (46.8±14.8) years.	118	Esophageal cancer, experimental group (postoperative radiotherapy group): 27 cases of squamous cell carcinoma, 32 cases of adenocarcinoma. TNM staging:<br>Postoperative chemotherapy group:<br>There were 17 cases in stage I<br>There were 23 cases in stage II<br>There were 19 cases in stage III<br>Postoperative radiotherapy group:<br>15 cases in stage I<br>26 cases in stage II<br>18 cases in stage III<br>Control group (postoperative chemotherapy group): 29 cases of squamous cell carcinoma, 30 cases of adenocarcinoma	Postoperative radiotherapy: TLS surgery: The patient underwent combined thoracoscopic and laparoscopic surgery (TLS) to treat esophageal cancer.<br>Postoperative radiotherapy: Radiotherapy begins 3 weeks after TLS surgery, using the following irradiation methods:<br>Vertical irradiation of the test field<br>Cross irradiation of the left, right and rear fields.<br>Front and rear field irradiation.<br>Cross irradiation of the rear three fields.<br>Radiotherapy dose: 60 Gy per 30 doses, maximum dose ≤ 66 Gy per 30 doses, with an irradiation interval of 1 month.<br>Radiotherapy duration: 6 months.	59	"CD4 + cells (%):<br>Before treatment: 34.71 ± 4.36<br>After treatment: 28.33 ± 2.84<br>CD8+ cells (%):<br>Before treatment: 33.63 ± 3.71<br>After treatment: 38.92 ± 4.68<br>CD3+ cells (%):<br>Before treatment: 61.86 ± 7.34<br>After treatment: 51.28 ± 5.37"	Postoperative chemotherapy: TLS surgery: The patient underwent combined thoracoscopic and laparoscopic surgery (TLS) to treat esophageal cancer.<br>Postoperative chemotherapy: Chemotherapy is initiated 3 weeks after TLS surgery, using the cisplatin and 5-fluorouracil (PF) regimen:<br>Day 1: Cisplatin 100 mg/m²<br>Days 1-5: 5-fluorouracil 1.0 g/m²<br>Chemotherapy interval: Repeated every 21 to 28 days, with a chemotherapy interval of 28 days (during concurrent radiotherapy in the postoperative chemotherapy group).	59
Laparoscopic Radical Hysterectomy Combined with Neoadjuvant Chemotherapy for Cervical Cancer Patients Effectively Improves Immune Function	/	Liu, M.	2022	/	female	Research group: Age range 20-65 years old, with an average age of 44.23 ± 6.38 years old.<br>Control group: Age range 20-65 years old, with an average age of 43.58 ± 7.88 years old.	42	Cervical Cancer Research Group<br>Squamous cell carcinoma: 16 cases<br>Adenocarcinoma: 3 cases<br>Adenosquamous carcinoma: 2 cases<br>Control group<br>Squamous cell carcinoma: 15 cases<br>Adenocarcinoma: 5 cases<br>Adenosquamous carcinoma: 1 case<br>Research group: 14 cases in Ib2 stage and 7 cases in IIa stage<br>Control group: 15 cases in Ib2 stage and 6 cases in IIa stage<br>Tumor size<br>Research group: Average 5.98 ± 1.11 cm<br>Control group: Average 6.12 ± 1.03 cm	Treatment plan: Neoadjuvant chemotherapy + laparoscopic radical hysterectomy<br>Neoadjuvant chemotherapy regimen:<br>Cyclophosphamide: 70mg/m2, intravenous injection, for 1 hours.<br>Cisplatin 60mg/m2, intravenous injection, for 1 hours.<br>Every three weeks constitutes a cycle, with a total of 2 to 3 cycles.	21	Before treatment: CD3+ cells 58.12 ± 2.53%, CD4+ cells 35.12 ± 3.27%, CD8+ cells 33.65 ± 1.33%, CD4+/CD8+ ratio 1.08 ± 0.03<br>After treatment: CD3+ cells 41.21 ± 1.93%, CD4+ cells 31.17 ± 1.88%, CD8+ cells 30.29 ± 1.23%, CD4+/CD8+ ratio 1.05 ± 0.06	Treatment plan: Neoadjuvant chemotherapy + open radical hysterectomy<br>Neoadjuvant chemotherapy regimen: The same as that of the experimental group<br>Surgical plan: Open radical hysterectomy	21
The effect of different nutrition on the immune function of patients with colorectal cancer	/	Li, K.	2009	/	There were 318 male cases (51.16%) and 302 female cases (48.84%)	The age range is 33 to 81 years old, with an average of 62.58 years old	620	Colorectal cancer, no staging	On the first day after the operation: 500 ml of 5% sugar water was pumped into the gastrointestinal nutrition tube at a rate of 30 ml/h.<br>From the second day after the operation: The Dutch Nutricia Nutrison Energy nutrient suspension (1000 ml, 4184 kJ of energy supply, 40 g of protein, pH 6.6, total osmotic pressure 250 mOsm/L) was pumped into the gastrointestinal nutrition tube.<br>From the 2nd to the 4th day after the operation: Provide energy at 1/3, 2/3 of the patient's basal energy expenditure (BEE) and the full amount (6276-8368 kJ), with a fluid volume of 1500-2000 ml and a temperature of 32-42°C, and continuously pump for 20-24 hours.<br>Days 4 to 7 after the operation: The usage method and speed are the same as those on day 4 after the operation. About one week after the operation: Stop the infusion of nutritional preparation and start a semi-liquid diet.	318	[illegible]	Starting from the second day after the operation: Parenteral nutrition preparation (Kabi nutrition solution) were administered via central or peripheral veins, and the calorie calculation standard was the same as that of the EN group.<br>After the anus has passed gas: Start a liquid diet.	318
Effect of early oral enteral nutrition on clinical outcomes after gastric cancer surgery	/	Mi, L.	2012	/	Male 33, female 27	Experimental group: 57.2±9.5 years old<br>Control group: 60.0±10.3 years old	60	Gastric cancer, no staging was mentioned	No nasojejunal tube will be retained after the operation. Start drinking a small amount of water on the first day after the operation.<br>On the second day after the operation, take 500 mL of the enteral nutrition preparation "Ruxian" orally and water once.<br>On the third day after the operation, take 1000 mL of "Ruxian TP" orally once and consume a small amount of liquid food at the same time.<br>Thereafter, continue to take 1000 mL of "Ruxian TP" orally every day and appropriately increase the intake of liquid or semi-liquid foods.	30	[illegible]	"Indwelling nasojejunal tube:"<br>Conventional intravenous nutrition was given after the operation.<br>After the gas is released, remove the nasojejunal tube and start taking oral water.<br>After there is no discomfort, gradually increase the intake of water, liquid and semi-liquid diets.	30
Effects of preoperative and postoperative enteral nutrition on postoperative nutritional status and immune function of gastric cancer patients	/	Ding, D.	2015	/	Experimental group: 35 males and 11 females.<br>Control group: 35 males and 11 females.	42-75 岁	92	Gastric cancer, no staging was mentioned	Preoperative Enteral nutrition group<br>One week before the operation: The patient takes 1000 kcal of complete protein enteral nutrition powder orally per day, while maintaining a semi-liquid diet.<br>On the first day after the operation: Starting 12 hours after the operation, 250 mL of isotonic normal saline was infused through the nasojejunal tube, followed by the infusion of 250 mL of isotonic normal saline + 500 mL of short peptide enteral nutrition suspension (SP).<br>Day 2 after the operation: Only 1000 mL of SP was infused.<br>On the 3rd day after the operation and thereafter: Starting from 72 hours after the operation, 500 mL of Murphy preparation emulsion + 500 mL of SP was infused.<br>From the 3rd to the 5th day after the operation: Only 1000 mL of Murphy was infused.	46	[illegible]	On the first day after the operation: Only 250 mL of isotonic normal saline was infused.<br>On the second day after the operation: Starting from 48 hours after the operation, 250 mL of isotonic normal saline and 500 mL of Murphy were infused through the nasojejunal tube.<br>From the 3rd to the 5th day after the operation: Only 1000 mL of Murphy was infused.	46

Clinical and immunological impact of early postoperative enteral immunonutrition after total gastrectomy in gastric cancer patients: A prospective randomized study	/	Marano, L.	2013	/	There were 34 males and 20 females in the IED group; There were 37 males and 18 females in the SEN group	IED Group: Average age 66.6 years (range 35-78 years); SEN group: Average age 65.1 years (range 49-83 years)	109	Gastric adenocarcinoma, and underwent elective total gastrectomy. N0: No lymph node metastasis (48.7%) N1: Lymph node metastasis (31.1%) N2: Lymph node metastasis (14.8%) N3: Lymph node metastasis (5.4%)	Immune-enhanced diet group: Immune-enhanced diet group (IED): Enteral nutrition through the jejunostomy tube was initiated 6 hours after the operation and continued until the 7th day after the operation	54	[illegible]	[illegible]	55	[illegible]
Clinical application of immune-enhanced enteral nutrition in patients with advanced gastric cancer after total gastrectomy	/	Liu, H.	2012	/	Male: 43; Female: 36	Average age: 58.3 years old; Age range: 26-77 years old	80	Advanced gastric cancer, without staging	TPN + Gln: [illegible]	[illegible]	[illegible]	[illegible]	[illegible]	[illegible]
The clinical significance of perioperative administration of enteral immunonutrition to patients with malignant gastrointestinal tumors	/	Huang, X.	2018	/	There were 12 males and 13 females in the immune-enhanced nutrition group; There were 14 males and 11 females in the traditional formula nutrition group	The age of the immune-enhanced nutrition group was (65.8±8.3) years old, and that of the traditional formula nutrition group was (65.7±7.4) years old	50	Gastric cancer, intestinal cancer, no staging	Ruineng (TPF-T) enteral nutrition emulsion [illegible]	[illegible]	[illegible]	[illegible]	[illegible]	[illegible]
Effect of immune-enhanced enteral nutrition on postoperative immune function and inflammatory responses in gastric cancer patients with radical gastrectomy	/	Liu, Z.	2011	/	There were 31 male cases and 11 female cases	Research Group: 61.38 ± 7.58; Control group: 61.68 ± 7.28	42	Advanced gastric cancer, without staging	[illegible]	[illegible]	[illegible]	[illegible]	[illegible]	[illegible]
Effects of early postoperative nutritional support on immune system and inflammatory responses after gastrointestinal surgery	/	Chen, H.	2006	/	There were 38 male cases and 38 female cases	64.74±18.22	46	There were 21 cases of colon cancer, 13 cases of rectal cancer, 5 cases of gastric cancer, and 1 case each of small intestinal malignant tumor, ampullary cancer and pancreatic cancer. No installments	Early enteral nutrition [illegible]	[illegible]	[illegible]	[illegible]	[illegible]	[illegible]
Influence of enteral nutrition during the preoperative and postoperative periods on postoperative nutritional status and immunological function in patients with gastric cancer	/	Wang, H. X.	2011	/	Male 82, female 44	Research group: Average age 60.4 years old; Control group: Average age 59.8 years old	126	Gastric cancer, no staging was mentioned	[illegible]	[illegible]	[illegible]	[illegible]	[illegible]	[illegible]
Randomized clinical trial of arginine-supplemented enteral nutrition versus standard enteral nutrition in patients undergoing gastric cancer surgery	/	Zhao, H.	2013	/	Male: 40 (55%); Female: 33 (45%)	Median age: 58 years; Age range: 42-74 years old	71	Gastric cancer, no staging was mentioned	An additional 8.8g [illegible]	[illegible]	[illegible]	[illegible]	[illegible]	[illegible]
The postoperative clinical outcomes and safety of early enteral nutrition in operated gastric cancer patients	/	Li, B.	2015	/	Control group: 112 males (56.0%) and 88 females (44.0%); Experimental group: 104 males (52.0%) and 96 females (48.0%)	Control group: Age range 40-79 years old, with an average of 58.8±7.6 years old; Experimental group: Age range 38-78 years old, average 60.0±5.9 years old	400	Gastric adenocarcinoma, no staging was mentioned	Early enteral nutrition [illegible]	[illegible]	[illegible]	[illegible]	[illegible]	[illegible]
Perioperative immunonutrition in head and neck cancer	/	Sorensen, D.	2009	/	Male	From 46 to 73 years old	21	Head and neck squamous cell carcinoma: Stage I: 4 cases; Stage II: 3 cases; Stage III: 3 cases; Stage IV: 7 cases	[illegible]	[illegible]	[illegible]	[illegible]	[illegible]	[illegible]
Impact of postoperative omega-3 fatty acid-supplemented parenteral nutrition on clinical outcomes and immunomodulations in colorectal cancer patients	/	Liang, B.	2008	/	Group FO: 18 males and 10 females; Group SO: 15 males and 6 females	Average age: 55.88 ± 18.38 years (FO group) and 58.19 ± 18.61 years (SO group)	41	Colon cancer and rectal cancer. Staging (TNM): Phase I: 6 people; Phase II: 18 people; Stage III: Radical resection for 18 people	[illegible]	[illegible]	[illegible]	[illegible]	[illegible]	[illegible]
Effect of postoperative early enteral nutrition on clinical outcomes and immune function of cholangiocarcinoma patients with malignant obstructive jaundice	/	Ma, B. Q.	2020	ChiCTR2000034 [illegible]	Experimental group (EEN + PN): 15 males and 11 females; Control group (TPN): 18 males and 12 females	The document mentions that all the subjects were adults, aged between 18 and 70. The average age of the experimental group was 58.88 ± 7.64 years old. The average age of the control group was 58.90 ± 7.42 years	56	Cholangiocarcinoma, accompanied by malignant obstructive jaundice. No installment size	[illegible]	[illegible]	[illegible]	[illegible]	[illegible]	[illegible]
Effect of postoperative enteral and parenteral nutrition on the immunity and inflammatory reaction of esophageal cancer patients	/	Tang, J.	2019	/	Among this group of patients, there were 82 males and 44 females, indicating that the proportion of males among the subjects was approximately 58.3% and that of females was approximately 41.7%	"Age: 36-75 years old, (48.2 ± 3.1) years old, median age: 55 years old."	100	There were 21 cases of cardia cancer, [illegible] cases of middle esophageal cancer and [illegible] cases of lower esophageal cancer, indicating that the tumor types of the subjects included cardia cancer, middle esophageal cancer and lower esophageal cancer. Installment: The article mentioned that "there were [illegible] cases in TNM stage IB, 21 cases in stage IIA, 31 cases in stage IIB, 21 cases in stage IIIA, and 8 cases in stage IIIB", indicating that the TNM stages of the subjects covered stage IB, IIA, IIB, IIIA and IIIB	[illegible]	[illegible]	[illegible]	[illegible]	[illegible]	[illegible]
Effect of EN combined with PN enriched with ω-3 polyunsaturated fatty acids on immune related indicators and early rehabilitation of patients with gastric cancer: A randomized controlled trial	Phase II	Yang, J.	2022	ChiCTR2000 [illegible]	"Male: 82 (6.8%) Female: 38 (3.2%)"	Age range: 27-78 years old; Average age: (57.8 ± 10.7)	120	Gastric adenocarcinoma. There is no distant metastasis, no tumor spread that lymph node metastasis, weight. Tumor size: Average diameter 4.3 [illegible] cm (median 3.1-5.6 cm)	[illegible]	[illegible]	[illegible]	[illegible]	[illegible]	[illegible]

Effects of enteral nutrition support combined with enhanced recovery after surgery on the nutritional status, immune function, and prognosis of patients with esophageal cancer after Ivor Lewis operation	/	Ding H.	2020	/	Experimental group (n=42): 22 males and 20 females, totaling 42 people. Control group (n=58): 29 males and 29 females, totaling 58 people	Experimental group (n=42): Median age 58.42±6.25 years. Control group (n=58): Median age 68.68±5.42 years	100	Lung cancer experimental group (n=42): 4 cases of adenocarcinoma and 38 cases of squamous cell carcinoma. Control group (n=58): 8 cases of adenocarcinoma and 50 cases of squamous cell carcinoma. Tumor staging (TNM) Experimental group (n=42): There are 11 people in Phase I, Phase II: 20 people, There are 11 people in Phase III. Control group (n=58): There are 11 people in Phase I, Phase II: 29 people, Phase III: 18 people	ERAS intervention: [illegible]	42	Experimental group (n=42) CD3+T cells: Preoperative: [illegible]; Postoperative: [illegible]; CD4+T cells: Preoperative: [illegible]; Postoperative: [illegible]; CD4+/CD8+ T cells: Preoperative: [illegible]; Postoperative: [illegible]	EN intervention: [illegible]	58	Control group (n=58) CD3+T cells: Preoperative: [illegible]; Postoperative: [illegible]; CD4+T cells: Preoperative: [illegible]; Postoperative: [illegible]; CD4+/CD8+ T cells: Preoperative: [illegible]; Postoperative: [illegible]
Effects of immune-enhanced enteral nutrition and parenteral nutrition on immune and nutritional function in elderly patients with gastric cancer after total gastrectomy	/	Liu H.	2021	/	Experimental group (glutamine-enhanced enteral nutrition group, Gln group): 17 males and 11 females. Control group (conventional enteral nutrition group, EN group): 21 males and 7 females. PN group (parenteral nutrition group): 19 males and 9 females	The age range was 60 to 83 years old, with a median age of 70.2 years old	84	For elderly patients with advanced gastric cancer, the TNM staging is as follows: Phase Ib: 11 students. Phase II: 42 people. Phase III: 31 people	Experimental group (glutamine-enhanced enteral nutrition group, Gln group): [illegible]	28	"Gln group" [illegible]	Control group (conventional enteral nutrition group, EN group): [illegible]	28+28	"EN group" [illegible]
Effects of neoadjuvant chemotherapy combined with enteral nutrition on postoperative immunity, inflammation and nutritional [illegible] gastric cancer patients	/	Jiang L.	2019	/	Observation group: 20 males and 20 females. Control group: 20 males and 20 females	Aged 35 to 78	96	Gastric cancer [illegible]	On the basis of the control group, enteral nutrition support was provided before the operation. [illegible]	48	[illegible]	[illegible]	48	[illegible]
Effects of Sijunzi decoction and enteral nutrition on T-cell subsets and nutritional status in patients with gastric cancer after operation: A randomized controlled trial	/	Cai J.	2008	/	/	/	59	Gastric cancer, none	Study Group A (n=21): [illegible]; Study Group B (n=20): [illegible]	A: 21; B: 20	[illegible]	[illegible]	18	[illegible]
Effect of intraoperative intraperitoneal chemotherapy and postoperative nutritional support on intestinal permeability and cellular immune function in patients with advanced gastric cancer	/	Cai B. M.	2012	/	There were 48 male cases and 17 female cases	The age range was 25 to 78 years old, with a median age of 58 years old	65	The pathological diagnosis of gastroscopy was advanced gastric adenocarcinoma (T2 and above)	HIIC + EN group: [illegible]; HIIC + PN group: [illegible]	HIIC + EN group: 22 cases; HIIC + PN group: 21 cases	[illegible]	[illegible]	[illegible]	[illegible]
Effect of general anesthesia combined with thoracic paravertebral block on Postoperative Immunity and tumor markers in patients with primary liver cancer	/	Wang M. Q.	2018	/	24 men and 16 women	Age (years): [illegible]	40	Liver cancer [illegible]	Combined with thoracic paravertebral nerve block anesthesia on the basis of the control group. [illegible]	20	[illegible]	[illegible]	20	[illegible]
Effect of Transcutaneous Electrical Acupoint Stimulation Combined with Transversus Abdominis Plane Block on Postoperative Recovery in Elderly Patients Undergoing Laparoscopic Gastric Cancer Surgery: A Randomized Controlled Trial	/	Xing R.	2022	ChiCTR2100042115	Male: 62 (71.0%); Female: 25 (28.7%)	Average age: [illegible] years old; Age range: 60-[illegible] years old	87	Gastric cancer, no staging was mentioned	Experimental group (TEAS + TAPB group, NTG): TEAS (Transcutaneous Electrical Acupoint Stimulation): [illegible]; TAPB (Transversus Abdominis Plane Block): [illegible]	[illegible]	[illegible]	[illegible]	[illegible]	[illegible]
Effect of Propofol and Desflurane on Immune Cell Populations in Breast Cancer Patients: A Randomized Trial	/	Woo, J. H.	2015	KCT0000915	female	The experimental group was [illegible] and the control group was [illegible]	40	Breast cancer: Stage 0: 1 case, Stage I: 23 cases, Stage II: 12 cases, Stage III: 3 cases	Anesthesia induction: [illegible] propofol [illegible]	[illegible]	[illegible]	[illegible]	[illegible]	[illegible]
Effect of two types of anesthesia on postoperative recovery of patients with gastric cancer and changes in the levels of their T lymphocyte subsets	/	Xie L.	2019	/	Experimental Group (GA + EP): Male: 70 people, Female: 30 people. Tumor type: Gastric cancer. Control Group (GA): Male: 68, Female: 32	Group GA: 41-67 years old, with an average of [illegible] years old. GA/EP group: 42-66 years old, with an average of [illegible] years old	200	Gastric cancer, no staging was mentioned	GA + EP [illegible]	[illegible]	[illegible]	GA [illegible]	[illegible]	[illegible]
Effects of combined epidural and general anesthesia on intraoperative hemodynamic responses, postoperative cellular immunity, and prognosis in patients with gallbladder cancer: A randomized controlled trial	/	Zhu J.	2017	/	There are 86 females and 58 males	Group CEGA: Average age [illegible] years. Group GA: Average age [illegible] years	144	Gallbladder cancer: TNM stage I to IV	Combined epidural - general anesthesia (CEGA) group: [illegible]	[illegible]	[illegible]	[illegible]	[illegible]	[illegible]
Effects of different anesthesia and analgesia on cellular immunity and cognitive function of patients after surgery for esophageal cancer	/	Zhu H.	2020	/	Male: 114 (95%); Female: 6 (5%)	Average age: [illegible] years old; Range: 45-78 years old	120	Esophageal cancer, no staging	GA + PCEA: [illegible]; GA + PCIA: [illegible]	[illegible]	[illegible]	[illegible]	[illegible]	[illegible]

Effects of different general anaesthetic techniques on immune responses in patients undergoing surgery for tongue cancer	-	Zhang, T.	2014	ChiCTR-TRC-13003198	Number of male patients: 43 (71.67%); Number of female patients: 17 (28.33%)	Average age: 58.5 years old; Age range: 40-78 years old	30	Tongue cancer. TNM staging: T1N0M0 (32 cases), T2N0M0 (21 cases), T3N0M0 (6 cases), T2N0M0 (1)	TIVA Group (Propofol induction and maintenance)	30	T0 (30 minutes before anesthesia): CD3+ cells 58.3±9.2%, CD3+CD4+ cells 37.5±9.5%, CD3+CD8+ cells 27.1±8.3%, CD4+/CD8+ ratio 1.6±0.8; T1 (1 hour after anesthesia): CD3+ cells 49.4±7.3%, CD3+CD4+ cells 30.4±7.9%, CD3+CD8+ cells 24.2±7.3, CD4+/CD8+ ratio 1.1±0.5; T2 (3 hours after anesthesia): CD3+ cells 48.1±7.7%, CD3+CD4+ cells 31.2±6.8%, CD3+CD8+ cells 27.2±8.9, CD4+/CD8+ ratio 1.1±0.6; T3 (5 hours after anesthesia): CD3+ cells 48.2±8.3%, CD3+CD4+ cells 30.8±9.2, CD3+CD8+ cells 28.2±7.8, CD4+/CD8+ ratio 1.2±0.5; T4 (at the end of surgery): CD3+ cells 48.1±6.7%, CD3+CD4+ cells 31.8±8.2, CD3+CD8+ cells 29.5±9.2, CD4+/CD8+ ratio 1.1±0.8; T5 (24 hours after surgery): CD3+ cells 49.3±8.5, CD3+CD4+ cells 31.2±7.8, CD3+CD8+ cells 26.8±8.3, CD4+/CD8+ ratio 1.1±0.8; T6 (48 hours after surgery): CD3+ cells 57.2±8.8, CD3+CD4+ cells 35.4±8.6, CD3+CD8+ cells 26.7±8.9, CD4+/CD8+ ratio 1.5±1.0; T7 (72 hours after surgery): CD3+ cells 60.3±9.4, CD3+CD4+ cells 38.2±8.4, CD3+CD8+ cells 26.3±9.8, CD4+/CD8+ ratio 1.5±0.8	MIXED	30
Effects of propofol and sevoflurane on postoperative immune responses in patients undergoing laparoscopic radical hysterectomy for cervical cancer	-	Liu, S.	2016	-	female	TIVA 45.86 ± 10.83; SEVO 48.31 ± 9.79	60	Cervical cancer: TIVA group (n=29): Ia2: 7 people (24.13%); Ib1: 10 people (34.48%); Ib2: 3 people (10.34%); IIa1: 5 people (17.24%); IIa2: 2 people (6.89%); IIb: 2 people (6.89%); SEVO Group (n=29): Ia2: 8 people (27.59%); Ib1: 9 people (31.03%); Ib2: 4 (13.79%); IIa1: 4 people (13.79%); IIa2: 3 people (10.34%); IIb: 1 person (3.45%)	TIVA group, induction and maintenance with propofol	30	Two primitive (T0): CD3+ cells: 1.88 ± 0.41 × 10^9/L; CD4+ cells: 1.05 ± 0.33 × 10^9/L; CD8+ cells: 0.69 ± 0.38 × 10^9/L; The ratio of CD4+ to CD8+: 1.76 ± 0.66; Post primitive (T1): CD3+ cells: 1.48 ± 0.32 × 10^9/L (significantly decreased); CD4+ cells: 0.82 ± 0.26 × 10^9/L (significantly decreased); CD8+ cells: 0.64 ± 0.29 × 10^9/L (no significant change); CD4+/CD8+ ratio: 1.37 ± 0.72 (significantly decreased); 24 hours after the operation (T2): CD3+ cells: 1.35 ± 0.41 × 10^9/L (significantly decreased); CD4+ cells: 0.81 ± 0.27 × 10^9/L (significantly decreased); CD8+ cells: 0.63 ± 0.27 × 10^9/L (no significant change); CD4+/CD8+ ratio: 1.38 ± 0.58 (significantly decreased); [illegible]	SEVO	30
The effects of epidural anesthesia and analgesia on T lymphocytes differentiation markers and cytokines in patients after gastric cancer resection	-	Wang, L.	2019	ChiCTR-INR-17012038	General anesthesia group (GA): 11 males (55%), 9 females (45%); Epidural combined with general anesthesia group (EGA): 10 males (50%), 10 females (50%)	General anesthesia group (GA): Average age 59.3 years (standard deviation 6.8); Epidural combined with general anesthesia group (EGA): Average age 58.7 years (standard deviation 6.5)	40	Gastric cancer TNM staging: General Anesthesia group (GA): Stage I: 3 cases (15%); Stage II: 2 cases (10%); Stage III: 12 cases (60%); Stage IV: 3 cases (15%); Epidural combined general anesthesia group (EGA): Stage I: 4 cases (20%); Stage II: 2 cases (10%); Stage III: 10 cases (50%); Stage IV: 4 cases (20%)	Group GA: Patients received intravenous injection of midazolam, sufentanil, propofol and cisatracurium for general anesthesia induction. During the operation, propofol and remifentanil were used for maintenance anesthesia.	20	[illegible]	Group EGA: Patients received epidural anesthesia in the T8-T10 intervertebral space before general anesthesia. During the operation, propofol and remifentanil were used for maintenance anesthesia and esophageal temperature was monitored.	20
Effect of low-dose naloxone on the immune system function of a patient undergoing video-assisted thoracoscopic resection of lung cancer with sufentanil controlled analgesia: A randomized controlled trial	-	Liu, Y.	2019	ChiCTR1900021641	Experimental group (Naloxone group): 17 males (48.57%), 18 females (51.43%); Control group (No naloxone group): 16 males (47.06%), 18 females (52.94%)	Average age: 55.4 years old (experimental group) and 55.4 years old (control group); Age range: 30-65 years old	69	Lung cancer. Lymph node metastasis: Yes in 1 case (2.86%), no in 34 cases (97.14%) (experimental group); Lymph node metastasis: 2 cases (5.88%) were yes, and 32 cases (94.12%) were no (control group)	"Naloxone: Postoperative analgesia regimen: The patient received sufentanil controlled analgesia (PCIA) within 48 hours after the operation. The PCIA solution contains sufentanil at 0.04 μg/kg/h (calculated for 48 hours) and palonosetron at 0.5 mg, and is diluted to 100 mL with normal saline. In addition, 0.05 μg/kg/h of naloxone was added to the PCIA solution (calculated for 48 hours). PCIA Settings: Single dose 2 mL, lock interval 20 minutes, background infusion 2 mL/h.	35	[illegible]	"Non-naloxone: Postoperative analgesia regimen: The patient received sufentanil controlled analgesia (PCIA) within 48 hours after the operation. The PCIA solution contains sufentanil at 0.04 μg/kg/h (calculated for 48 hours) and palonosetron at 0.5 mg, and is diluted to 100 mL with normal saline. This group did not add granulolone. The PCIA setting was the same as that of the experimental group.	34
Application effect of dexmedetomidine combined with flurbiprofen axetil and flurbiprofen axetil monotherapy in radical operation of lung cancer and evaluation of the immune function	-	Zeng, S.	2021	-	Group F (flurbiprofen axetil alone group): 18 males and 12 females, totaling 30 cases. DMF group (dexmedetomidine combined with flurbiprofen axetil group): 19 males and 11 females, totaling 30 cases.	The age range is 35 to 65 years old	60	Lung cancer, no mention of stage size	"DMF Group (dexmedetomidine combined with flurbiprofen axetil group): Twenty-five minutes before anesthesia induction, dexmedetomidine at a dose of 0.5 μg/kg (diluted to 20 mL with normal saline) was intravenously injected, and the injection was completed within 20 minutes. Five minutes before anesthesia induction, flurbiprofen axetil at a dose of 1 mg/kg was intravenously injected. Anesthesia induction: Intravenous injection of midazolam at a dose of 0.06 mg/kg, etomidate at a dose of 0.3 mg/kg, sufentanil at a dose of 0.3 μg/kg, and rocuronium at a dose of 0.6 mg/kg was administered. One minute later, a double-lumen bronchial catheter was inserted for mechanical ventilation. Anesthesia maintenance: Intravenous infusion of propofol at 4-6 mg/kg/h, inhalation of sevoflurane at 1-2%, intravenous infusion of remifentanil at 0.15-0.3 μg/kg/min, and intermittent intravenous injection of rocuronium at 0.05 mg/kg. Postoperative analgesia: Patient-controlled intravenous analgesia (PCIA) was performed using sufentanil. The specific method was as follows: 2 μg/kg of sufentanil and 10 mg of tropisetron were diluted to 100 mL with normal saline. The loading dose of sufentanil is 0.1 μg/kg, the booster dose is 1 mL, the maintenance dose is 2 mL/h, and the lock-in time is 15 minutes.	30	[illegible]	Group F (flurbiprofen axetil alone group): Ten minutes before anesthesia induction, flurbiprofen axetil at a dose of 1 mg/kg was intravenously injected. Anesthesia induction: Intravenous injection of midazolam at a dose of 0.06 mg/kg, etomidate at a dose of 0.3 mg/kg, sufentanil at a dose of 0.3 μg/kg and rocuronium at a dose of 0.6 mg/kg was administered. One minute later, a double-lumen bronchial catheter was inserted for mechanical ventilation. Anesthesia maintenance: Intravenous infusion of propofol at 4-6 mg/kg/h, inhalation of sevoflurane at 1-3%, intravenous infusion of remifentanil at 0.15-0.3 μg/kg/min, and intermittent intravenous injection of rocuronium at 0.05 mg/kg. Postoperative analgesia: Patient-controlled intravenous analgesia (PCIA) was performed using sufentanil. The specific method was as follows: 2 μg/kg of sufentanil and 10 mg of tropisetron were diluted to 100 mL with normal saline. The loading dose of sufentanil is 0.1 μg/kg, the booster dose is 1 mL, the maintenance dose is 2 mL/h, and the lock-in time is 15 minutes.	30
Effects of dexmedetomidine combined with dexamethasone on T lymphocytes, NK cells and cognitive function in elderly patients with gastrointestinal cancer after radical surgery	-	Ai, Z.	2023	-	Experimental Group (Group D): 52, including 26 males and 26 females. Control Group (Group C): 52, including 29 males and 23 females.	Aged 65 to 75	104	Elderly patients with gastrointestinal tumors aged 65 to 75, among whom Specific tumor types, including gastric cancer, colon cancer and rectal cancer. TNM staging: TNM staging I-II.	"Group D: Twenty minutes before the operation: Intravenous infusion of dexmedetomidine (0.5 μg/kg). Anesthesia induction: Tracheal intubation was performed with propofol (1.5-2.0 mg/kg) + remifentanil (0.3 μg/kg) + cisatracurium (0.2 mg/kg). Intraoperative anesthesia maintenance: Propofol (0.10-0.25 μg/(kg·min)) + remifentanil (0.10-0.25 μg/(kg·min)) + intermittent intravenous injection of cisatracurium. Thirty minutes before the end of the operation: Discontinue the infusion of cisatracurium and administer dexmedetomidine (0.5 μg/kg) and dezocine (0.1 mg/kg) intravenously. Twenty minutes before the end of the operation: Discontinue the infusion of propofol and remifentanil. Postoperative: Routine tracheal tube removal and transfer back to a regular ward.	52	[illegible]	Control Group (Group C): Thirty minutes before the operation: Intravenous infusion of sufentanil (1.0μg/kg). Anesthesia induction: Tracheal intubation was performed with propofol (1.5-2.0 mg/kg) + sufentanil (0.3 μg/kg) + cisatracurium (0.2 mg/kg). Intraoperative anesthesia maintenance: Propofol (0.10-0.25 μg/(kg·min)) + remifentanil (0.10-0.25 μg/(kg·min)) + intermittent intravenous injection of cisatracurium. Thirty minutes before the end of the operation: Stop the infusion of cisatracurium and intravenously inject 10ml of 0.9% sodium chloride solution. Twenty minutes before the end of the operation: Discontinue the infusion of propofol and remifentanil. Postoperative: The tracheal tube was routinely removed and the patient was transferred back to a regular ward.	52
Effects of dexmedetomidine on inflammatory factors, T lymphocyte subsets and expression of [illegible] in peripheral blood mononuclear cells in patients receiving radical surgery of colon carcinoma	-	Wang K.	2018	-	Experimental group: 36 males and 36 females. Control group: 33 males and 36 females.	Experimental group: Average age 42.5±4.3 years (range 32-69 years); Control group: Average age 42.3±5.8 years (range 34-67 years)	141	Colorectal cancer, no staging was mentioned	Dexmedetomidine was pumped before the operation (the loading dose of 1 μg/kg for 10-15 minutes), then the pumping dosage was set to 1 μg/(kg·h)	72	[illegible]	Before the operation, the same dose of normal saline as that in the experimental group was pumped.	69
Effects of Oxycodone Combined with Dexmedetomidine on Immune Function of Patients Undergoing Radical Mastectomy with General Anesthesia	-	Bai Y.	2020	-	female	Aged 34 to 63	300	All are stage I or stage II breast cancer	Dexmedetomidine group: Preoperative: 0.5 μg/kg dexmedetomidine is intravenously pumped 30 minutes before anesthesia induction, and the infusion is completed within 15 minutes. Then, the dose of 0.4 μg/kg/h is maintained until 30 minutes before the end of the operation. During the operation: Anesthesia induction and maintenance were the same as those in the oxycodone group. Postoperatively: 0.3 μg/kg/d fentanyl for patient-controlled intravenous analgesia, loading dose 1 mL, continuous dose 1 mL/h. 3. Joint Group: Preoperative: Oxycodone and dexmedetomidine were administered simultaneously, with the same dosage and administration as the above two groups. During the operation: Anesthesia induction and maintenance were the same as those in the oxycodone group. Postoperatively: 0.3 μg/kg/d fentanyl for patient-controlled intravenous analgesia, loading dose 1 mL, continuous dose 1 mL/h.	100+100	[illegible]	Oxycodone Group: Preoperative: 2.5 mg of oxycodone was intravenously injected 30 minutes before the operation. Intraoperative: Anesthesia induction: Intravenous injection of 2 mg/kg propofol, 3 μg/kg sufentanil, 0.15 mg/kg cisatracurium, followed by mechanical ventilation after tracheal intubation. Anesthesia maintenance: Continuous intravenous injection of 6 mg/kg/h propofol, 0.03-0.20 μg/kg/min remifentanil, and inhalation of 1%-3% sevoflurane. Postoperatively: 0.3 μg/kg/d fentanyl for patient-controlled intravenous analgesia, loading dose 1 mL, continuous dose 1 mL/h.	100
Effects of parecoxib sodium on postoperative immune function and tumor micrometastasis in patients with lung cancer	-	Chen, M.	2022	-	There were 31 male cases and 29 female cases	Between 40 and 64 years	60	Non-small cell lung cancer, and at stage I or II of TNM staging	Experimental group (Parecoxib sodium group): Twenty minutes before anesthesia induction: Intravenous injection of 40mg of parecoxib sodium (diluted with 5mL of normal saline). Anesthesia induction: Intravenous injection of midazolam, sufentanil, etomidate, and rocuronium, followed by endotracheal intubation and mechanical ventilation. Anesthesia maintenance: Intravenous infusion of remifentanil, propofol, and rocuronium, continuous inhalation of sevoflurane, to maintain the bispectral index of electroencephalogram between 40 and 60. Postoperative analgesia: A patient-controlled intravenous analgesia pump was used, including sufentanil, dezocine, dexmedetomidine and tropisetron. Twelve hours after the operation, 40mg of parecoxib sodium was intravenously injected (diluted with 5mL of normal saline).	30	[illegible]	"Control group (normal saline group): Twenty minutes before anesthesia induction: 5mL of normal saline is intravenously injected. Anesthesia induction: Intravenous injection of midazolam, sufentanil, etomidate, and rocuronium, followed by endotracheal intubation and mechanical ventilation. Anesthesia maintenance: Intravenous infusion of remifentanil, propofol, and rocuronium, continuous inhalation of sevoflurane, to maintain the bispectral index of electroencephalogram between 40 and 60. Postoperative analgesia: A patient-controlled intravenous analgesia pump was used, including sufentanil, dezocine, dexmedetomidine and tropisetron. Twelve hours after the operation, 5mL of normal saline was intravenously injected.	30
Effects of postoperative multimodal analgesia with dezocine and dexmedetomidine on cellular immune function in patients after radical gastric cancer surgery	-	Gao, Y. F.	2014	-	There were 29 male cases and 11 female cases	Aged 40 to 70	40	Gastric cancer, no staging was mentioned	Experimental Group (DF Group): Postoperative analgesia regimen: dezocine 30 mg + flurbiprofen axetil 150 mg, add tropisetron 6 mg, dilute to 100 mL with normal saline. Analgesic pump parameters: Initial loading dose 4 mL, background dose 2 mL/h, single PCA dose 0.5 mL, lock time 15 minutes, analgesic time 48 hours.	20	[illegible]	Control Group (Group F): Postoperative analgesia regimen: Fentanyl 0.01 mg/(kg·d), add tropisetron 6 mg, dilute to 100 mL with normal saline. Analgesic pump parameters: Initial loading dose 4 mL, background dose 2 mL/h, single PCA dose 0.5 mL, lock time 15 minutes, analgesic time 48 hours.	20

Effects of sufentanil and morphine on T lymphocyte subsets during postoperative analgesia in malignant bone tumor radical resection patients	[illegible]	Li, W. K.	2008	[illegible]	Male: 21 cases Female: 11 cases	Aged 18 to 67 Average age: 32 years old Median age: 40 years old	32	[illegible]	[illegible]	[illegible]	[illegible]	[illegible]	[illegible]
The impact of [illegible] on dysfunction of T-lymphocyte subsets and natural killer cells in cancer patients receiving postoperative morphine analgesia	[illegible]	Shen, J. C.	2014	[illegible]	[illegible]	[illegible]	[illegible]	Gastric cancer, ASA grade I or II	[illegible]	[illegible]	[illegible]	[illegible]	[illegible]
Postoperative analgesia with parecoxib sodium improves postoperative pain and immune function in patients undergoing hepatectomy for hepatocellular carcinoma	[illegible]	Wang, R. D.	2020	[illegible]	[illegible]	[illegible]	[illegible]	[illegible]	[illegible]	[illegible]	[illegible]	[illegible]	[illegible]
Which has the least immunity depression during postoperative analgesia-morphine, tramadol, or tramadol with lornoxicam?	[illegible]	Wang, Z. Y.	2006	[illegible]	[illegible]	Aged 26 to 60	[illegible]	Gastric cancer, ASA grade I or II	[illegible]	[illegible]	[illegible]	[illegible]	[illegible]
Comparison between the effects of intravenous morphine, tramadol, and ketorolac on stress and immune responses in patients undergoing modified radical mastectomy	[illegible]	Rais, M. A. E. M.	[illegible]	[illegible]	female	M: 58.90±12.32 T: 45.25±11.93 K: 46.25±10.42	[illegible]	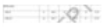	[illegible]	[illegible]	[illegible]	[illegible]	[illegible]
[illegible] Regression on Postoperative Analgesia and Im[illegible]	[illegible]	[illegible]	[illegible]	[illegible]	[illegible]	[illegible]	[illegible]	Colon cancer patients: Group A: 27 cases in stage I and 32 cases in stage II. Group B: 24 cases in stage I and 35 cases in stage II	[illegible]	[illegible]	[illegible]	[illegible]	[illegible]
Effect of Thymosin α1 Combined with Interventional Therapy on Liver Function and Immune Cells of Patients with Advanced Hepatocellular Carcinoma	[illegible]	Wang, D.	2018	[illegible]	Experimental group: 18 males (64.3%), 10 females (35.7%) Control group: 20 males (71.4%), 8 females (28.6%)	[illegible]	56	[illegible]	[illegible]	[illegible]	[illegible]	[illegible]	[illegible]
Evaluation of the efficacy of apatinib and camrelizumab combined with transcatheter arterial chemoembolization in the treatment of primary hepatocellular carcinoma	[illegible]	Chen, C.	2023	[illegible]	[illegible]	[illegible]	[illegible]	[illegible]	[illegible]	[illegible]	[illegible]	[illegible]	[illegible]
Clinical Effect and Diffusion-Weighted Imaging Characteristics of Sorafenib in Patients with Hepatocellular Carcinoma	Phase II	Li, X.	2019	无	[illegible]	[illegible]	[illegible]	[illegible]	[illegible]	[illegible]	[illegible]	[illegible]	[illegible]
Effects of Apatinib Mesylate Monotherapy on the Incidence of Adverse Reactions and Immune Function in Patients with Breast Cancer after Radical Mastectomy	[illegible]	Liu, S.	2022	[illegible]	female	[illegible]	[illegible]	[illegible]	[illegible]	[illegible]	[illegible]	[illegible]	[illegible]
Changes of immune function in liver cancer patients after transcatheter arterial chemoembolization combined with interstitial therapy	Phase II	Quan, Y.	2009	[illegible]	[illegible]	[illegible]	[illegible]	[illegible]	[illegible]	[illegible]	[illegible]	[illegible]	[illegible]
Lienal polypeptide injection combined with transcatheter hepatic arterial chemoembolization for treating primary hepatic cancer: Curative effect and influence on cellular immune function	[illegible]	Zhao, Y. S.	2016	[illegible]	[illegible]	[illegible]	[illegible]	[illegible]	[illegible]	[illegible]	[illegible]	[illegible]	[illegible]

Study on the efficacy and safety of high-dose thymopentin combined with transcatheter arterial chemoembolization in primary liver cancer	/	Li, T.	2007	/	Male: 32 cases; Female: 18 cases	Aged 36 to 68	50	Primary liver cancer, no staging	Thymic pentapeptide treatment was administered after transcatheter arterial chemoembolization (TACE). Intravenous infusion: Dissolve 10 mg of thymic pentapeptide in 250 ml of normal saline once a day for 5 consecutive days (d1-d5). Intramuscular injection: Dissolve 10 mg of thymic pentapeptide in 2 ml of normal saline once a day for 16 consecutive days (d6-d21). Each cycle lasts for 21 days.	25	Changes in T cell subsets in the treatment group: CD4+ T cells: Before treatment: 38.8 ± 8.94 %; One week after treatment: 46.3 ± 9.25 %*; After treatment (3 weeks): 48.2 ± 5.53 *; CD8+ T cells: Before treatment: 28.1 ± 8.23 %; One week after treatment: 29.8 ± 8.84; After treatment (3 weeks): 31.4 ± 8.25 *; NK cells: Before treatment: 18.2 ± 8.32 %; One week after treatment: 21.4 ± 8.25 *; After treatment (3 weeks): 25.4 ± 5.98 △	Only transcatheter arterial chemoembolization (TACE) was performed. The chemotherapy regimen was the same as that of the experimental group, but thymic pentapeptide treatment was not performed.	25
Clinical Efficacy and Safety of Tumor Cytoreductive Surgery plus Hyperthermic Intraoperative Chemotherapy in Ovarian Cancer	/	Yu, H.	2021	/	female	Observation group (n = 33): Aged 27-74 years, with an average age of 50.62 ± 5.73 years. Experimental group (n = 33): Aged 26-76 years, with an average age of 50.74 ± 5.69 years.	66	Ovarian cancer: observation group (n = 33): 18 cases of primary tumors and 15 cases of recurrent tumors. Experimental group (n = 33): 19 cases of primary tumors and 14 cases of recurrent tumors.	Cytoreductive surgery (CRS), hyperthermic intraoperative chemotherapy (HIPEC).	33	Before Treatment: CD3 + (58.33 ± 3.35) %, CD4 + (26.71 ± 2.72) %, CD3 + / CD4 + (1.35 ± 0.14); After treatment: CD3 + (53.59 ± 3.65) %, CD4 + (16.18 ± 3.71) %, CD3 + / CD4 + (0.94 ± 0.11)	Cytoreductive surgery (CRS): The same as the experimental group. Intravenous chemotherapy.	33
Effect of intraoperative intraperitoneal chemotherapy and postoperative nutritional support on intestinal permeability and cellular immune function in patients with advanced gastric cancer	/	Gu, B. M.	2012	/	There were 48 male cases and 17 female cases.	The age range was 25 to 78 years old, with a median age of 58 years old.	65	The pathological diagnosis of gastroscopy was advanced gastric adenocarcinoma (T2 and above).	HIIC + EN group: A group of patients who received intraoperative intraperitoneal chemotherapy (HIIC) and postoperative enteral nutrition support (EN). HIIC + PN group: A group of patients who received intraoperative intraperitoneal chemotherapy (HIIC) and postoperative parenteral nutrition support (PN).	HIIC + EN 组：22 例; HIIC + PN 组：21 例	"HIIC plus EN group: CD4+ (%): One day before the operation: 35.8 ±6.9; 12 days after the operation: 39.5 ±6.7*; CD8+ (%): One day before the operation: 28.2 ±5.5; Twelve days after the operation: 28.8±5.5; CD4/CD8: One day before the operation: 1.88 ±0.36; 12 days after the operation: 2.28 ±0.62*; NK(%): One day before the operation: 15.3 ±3.9; 12 days after the operation: 15.2±4.9*	Group EN	20
Effect of operation and enduring hyperthermic intraperitoneal chemotherapy on cellular immune function in patients with adenocarcinoma of esophagogastric junction	/	Nie, Z.	2021	/	/	/	252	Patients with advanced adenocarcinoma of the esophagogastric junction (AEG).	After the surgery, paclitaxel (PTX) hyperthermic intraperitoneal chemotherapy (HIPEC) was received.	126	CD3+ cells: 65.14 ± 6.35 %; CD3+CD4+ cells: 40.8 ± 7.00 %; CD3+CD8+ cells: 22.87 ± 5.69 %	Only surgery was received, not HIPEC.	126
Effect of preoperative intraarterial chemotherapy combined with hyperthermia on sPD-L1 and CEA levels and overall survival of elderly patients undergoing radical resection of lung cancer	/	Zhou, L.	2019	/	Male: 63 people; Female: 73 people	Individual treatment group: Average age 74.8 ± 4.4 years; Combined treatment group: Average age 72.7 ± 3.1 years	136	Lung cancer: squamous cell carcinoma 65 cases; Adenocarcinoma: 77 cases; Stage II: 54 cases (79.4%); Phase III: 14 people (20.6%)	The treatment method for the combined therapy group: Patients undergoing radical lung cancer resection, preoperative perfusion chemotherapy and high-frequency hyperthermia before and after the operation were received. Chemotherapy regimen: Chemotherapy drugs include mitomycin and cisplatin, and the dosage is adjusted according to the patient's body surface area and physical condition. Hyperthermia plan: High-frequency hyperthermia is administered one hour before the operation, with a frequency of 13.56Hz, a temperature of 41-44°C, a voltage of 200V, and a treatment duration of one hour. Hyperthermia of the same method was performed again 24 hours after perfusion chemotherapy. Treatment course: Chemotherapy is administered every three weeks, for a total of [illegible] cycles.	68	"Combined treatment group: CD3: Before treatment: 868.22; After treatment: 1112.7; CD4: Before treatment: 611.71; After treatment: 781.93; CD8: Before treatment: 782.63; After treatment: 441.05"	Only preoperative perfusion chemotherapy was received, and the chemotherapy regimen was the same as that of the experimental group.	68
Efficacy of cisplatin plus paclitaxel in chemotherapy in patients with cervical cancer after laparoscopic nerve-sparing extensive hysterectomy and its effect on immune function	/	Zhang, J.	2022	/	female	Observation group: Average age (46.3±7.4) years, age range 25-68 years. Control group: Average age (47.2±6.3) years, age range 26-69 years.	44	Cervical cancer experimental group: 19 cases of squamous cell carcinoma and 3 cases of adenocarcinoma. Control group: 18 cases of squamous cell carcinoma, 4 cases of adenocarcinoma	Routine pretreatment: Oral administration of dexamethasone, cimetidine and other drugs. Chemotherapy plan: Day 1: Intravenous infusion of paclitaxel at a dose of 135 mg/m2 for at least 3 hours. Days 1-3: Intravenous infusion of cisplatin at a dose of 25 mg/m2. Chemotherapy cycle: Every 4 weeks constitutes one cycle, and several cycles are conducted in total.	22	Proportion of CD3+ cells: 43.8 6±5.31 % before treatment, 63.23±4.34 % after treatment; CD4+ cell ratio: 44.2 5±4.82 % before treatment, 55.13±4.92 % after treatment; Proportion of CD8+ cells: 48.62±4.71 % before treatment, 33.23±4.17 % after treatment	Days 1-3: Intravenous infusion of cisplatin at a dose of 25 mg/m2. Days 1-5: Intravenous infusion of fluorouracil at 500 mg/m2. Chemotherapy cycle: Every 4 weeks constitutes one cycle, and several cycles are conducted in total.	22
Low-dose interleukin-2 administered pre-operatively to patients with gastric cancer activate peripheral and peritumoral lymphocytes but does not affect prognosis	Phase II	Cesana, G. C.	2007	NCT00005 254	There were 41 males and 27 females	The average age is 65	68	Gastric cancer: Experimental group: 6 cases in stage I, 5 cases in stage II, 14 cases in stage III, and 5 cases in stage IV. Control group: 7 cases in stage I, 5 cases in stage II, 14 cases in stage III, and 6 cases in stage IV.	IL-2 treatment: Patients in the experimental group received subcutaneous injection of low-dose IL-2 (9 million units each time, twice a day for 3 consecutive days). Surgery: The patient underwent gastric cancer surgery 36 hours after the last IL-2 injection.	36	"Case Group: CD3: Preoperative: 1078.36; 7d: 1011.76; 14d: 1181.85; 28d: 1338.32; CD4: Preoperative: 688.468; 7d: 627.481; 14d: 706.585; 28d: 768.4; CD8: Preoperative: 436.677; 7d: 364.336; 14d: 431.382; 28d: 361.544; NK: Preoperative: 238.367; 7d: 241.211; 14d: 271.831; 28d: 338.321"	The patient directly underwent gastric cancer surgery without any preoperative treatment.	32
Effect of Dahuang Zhechong Pills combined with TACE on VEGF, MMP-2, TGF-β1 and immune function of patients with primary liver cancer (blood stasis and collaterals blocking type)	/	Dai, C. M.	2021	/	/	Aged 38 to 75	79	Experimental group: 28 cases of cholangiocellular type and 11 cases of hepatocellular type. Control group: 28 cases of cholangiocellular type and 12 cases of hepatocellular type. Tumor staging: The article mentions that the tumor stage is stage II-III.	"Western medical treatment: Transcatheter arterial chemoembolization (TACE) was performed. Preoperative preparation: Puncture and catheterization, inject anti-chemotherapy drugs (cisplatin, epirubicin hydrochloride) and iodized oil embolization agent. After the operation, basic treatments such as antiemesis, hydration, stomach protection and liver protection were given, and the condition was monitored. After TACE, thymosin alpha1 subcutaneous injection is routinely given twice a week. Traditional Chinese Medicine treatment: Based on Western medical treatment, take Dahuang Zhechong Pills orally twice a day, 3 grams each time. Continue the treatment for [illegible]	39	Experimental group (39 cases): Before treatment: Percentage of CD4+ cells: 31.4 ±4.67 %; Percentage of CD3+ cells: 47.6 ±6.47 %; The ratio of CD4+ to CD8+: 1.22 ±0.21; After treatment: Percentage of CD4+ cells: 40.8 ±4.85 % (significantly higher than before treatment); Percentage of CD3+ cells: 60.8 ±6.45 % (significantly higher than before treatment); CD4+/CD8+ ratio: 1.74 ±0.38 (significantly higher than before treatment)	No traditional Chinese medicine treatment is carried out.	40
Effect of early perioperative enteral nutrition support with rhubarb and Sijunzi decoction on immune function and nutrition of surgical patients with gastric cancer	/	Wu, H.	2007	/	Male: 22 cases; Female: 18 cases	Aged 32 to 77	40	Gastric cancer, except for those with distant metastasis, who undergo radical resection and those who cannot be resected	Rhubarb 10 grams	20	"Research Group": One day before the operation: CD3(%): 71.64 ± 11.36; CD4(%): 44.81 ± 11.06; CD8(%): 27.71 ± 10.75; CD4/CD8: 1.58 ± 1.11; One day after the operation: CD3(%): 58.20 ± 11.08; CD4(%): 30.41 ± 11.87; CD8(%): 27.81 ± 5.40; CD4/CD8: 1.10 ± 0.49; Ten days after the operation: CD3(%): 71.52 ± 11.14 ▲; CD4(%): 48.64 ± 8.8▲; CD8(%): 24.5 ± 7.34; CD4/CD8: 2.34 ± 0.88▲; Note: ▲ indicates that compared with the control group, P < 0.05.	control	20
Effect of cellular immune supportive treatment on immunity of esophageal carcinoma patients after modified radical lymphadenectomy	/	Wang, J. Y.	2007	/	Male: 38, female: 2	The average value was 58.5 for the experimental group and 58 for the control group.	60	Esophageal cancer: IIa: 6, IIb: 15, III: 37, IV: 2	Starting from the 4th day after the operation, 12,500 u of Shenqi injection was intravenously infused daily for 7 days.	30	"Experimental Group": Before surgery: CD3+: 61.8 ± 5.8; CD3+CD4+: 33.2 ± 12.8; CD3+CD8+: 19.2 ± 8.9; CD4+/CD8+: 2.2 ± 1.3; Three days after the operation: CD3+: 60.1 ± 11.6; CD3+CD4+: 33.3 ± 11.3*; CD3+CD8+: 22.4 ± 10.3; CD4+/CD8+: 1.9 ± 1.3*; Nine days after the operation: CD3+: 65.8 ± 9.1*; CD3+CD4+: 37.9 ± 12.9*; CD3+CD8+: 21.9 ± 12.3; CD4+/CD8+: 2.4 ± 1.4**	Two-field cleaning technique	30
Influence of Yupingfeng Powder Adjuvant Therapy on Treatment Effect and Anti-tumor Immunity of Patients with Primary Liver Cancer	/	Zhao, N.	2017	/	There were 21 males and 19 females in the control group, and 22 males and 18 females in the observation group.	Control group: 40-75 years old, with an average of (57.28±6.11) years old. Observation group: 40-74 years old, with an average of (54.73±8.09) years old.	80	"Primary liver cancer: Control group: The maximum diameter of the primary tumor was 2 to 6 cm, with an average of (4.12±0.86) cm. Observation group: The maximum diameter of the primary tumor ranged from 2 to 6 cm, with an average of (4.11±0.75) cm.	Patients in the observation group began to take Yupingfeng Powder early after palliative surgeries (including palliative hepatectomy, hepatic artery ligation, portal vein catheterization chemotherapy, etc.) 5.0g each time, three times a day, and continue the treatment for three months.	40	"Experimental Group (Observation Group): Before treatment: CD3+ T lymphocytes: 62.43 ± 5.98%; CD4+ T lymphocytes: 33.51 ± 4.29%; CD8+ T lymphocytes: 29.62 ± 3.29%; CD4+/CD8+ ratio: 0.87 ± 0.09; One month after treatment: CD3+ T lymphocytes: 68.38 ± 7.11% (*); CD4+ T lymphocytes: 40.27 ± 5.82% (*); CD8+ T lymphocytes: 34.18 ± 3.33% (*); CD4+/CD8+ ratio: 1.46 ± 0.33%	Patients in the control group only received palliative surgeries (including palliative hepatectomy, hepatic artery ligation, portal vein catheterization chemotherapy, etc.).	40
Effect of Sijunzi decoction and enteral nutrition on T-cell subsets and nutritional status in patients with gastric cancer after operation: A randomized controlled trial	/	Cai, J.	2008	/	/	/	70	Gastric cancer	Study Group A (n=21): On the first and second days after the operation, 500 ml of normal saline was injected through the upper nasogastric feeding tube. Enteral nutrition (EN) was administered from the 2nd to the 7th day after the operation, and the EN solution used was [illegible] produced by Nutricia Company. The dosage can be increased gradually from small to large. On the second and third days after the operation, it is 500 ml (flat pour 20-30 ml/h), and on the fourth day after the operation, it is 1500 ml (flat pour 80-100 ml/h). Starting from the 5th day, 2000-2500 ml (flat pour 100-125 ml/h) should be administered evenly with an infusion pump until the 7th day after the operation, gradually transitioning to a semi-liquid diet and a normal diet. The insufficient amounts of fluid, heat and nitrogen are all made up through the parenteral approach. Do not use the Four Gentlemen Decoction. Study Group B (n=18): The nutritional support methods were the same as those in Study Group A. Starting from the second day after the operation, the Four Junzi Decoction (15g of Codonopsis pilosula, 20g of Atractylodes macrocephala, 20g of Poria cocos, and 10g of Glycyrrhiza uralensis) was instilled through a nasogastric tube placed during the operation. After the nasogastric tube was removed during ventilation and defecation after the operation, it was taken orally and continued until the ninth day after the operation, at a rate [illegible]	Study Group A: 21 people; Study Group B: 18 people	Study Group A (n=21): Preoperative: CD3+: 46.46 ± 8.33; CD4+: 42.39 ± 6.31; CD4+/CD8+: 1.65 ± 0.34; Day 1 after the operation: CD3+: 31.77 ± 5.30; CD4+: 34.38 ± 5.23; CD4+/CD8+: 1.32 ± 0.30; Day 10 after the operation: CD3+: 60.36 ± 7.77*; CD4+: 40.07 ± 6.07*; CD4+/CD8+: 1.50 ± 0.36*	Control group (n=30): The patient received routine fluid replacement and parenteral nutrition support (PN) for 9 days after the operation. It provides approximately 83.68-104.6 KJ (20-25 kcal) of non-protein calories per kg each day, with a nitrogen content of 0.16 g/kg, which is equivalent to 1.0 g/kg of branched-chain amino acid. Non-protein calories are provided by glucose and 30% fat emulsion. Fat energy accounts for 30% to 50% of the total non-protein calories, and the ratio of sugar to exogenous regular insulin is 4:1 to 5:1. At the same time, electrolyte, trace elements and vitamins were given. Enteral nutrition was not administered within 7 days. Only from the first day after the operation, 500 ml of normal saline was injected daily through the superior fecal canal as the fluid volume control.	30
Effect of Zilongjin Tablets on immune function of patients with lung cancer after operation	/	Huang, Z. H.	2019	/	There were 21 males and 9 females in the experimental group. In the control group, there were 18 males and 12 females.	The median age of the subjects was 58 years (for the control group) and 58 years (for the experimental group), ranging from 37 to 75 years.	60	Lung cancer: The tumor types of the subjects include adenocarcinoma, squamous cell carcinoma, small cell carcinoma and other types. In the experimental group, there were 14 cases of adenocarcinoma, 14 cases of squamous cell carcinoma, 1 case of small cell carcinoma, and 1 case of other types. In the control group, there were 16 cases of adenocarcinoma, 11 cases of squamous cell carcinoma, 0 cases of small cell carcinoma, and 3 cases of other types. Best allocation. The article mentions that the clinical stages of the subjects include stage I, stage II, and stage IIIA. In the experimental group, there were 13 cases in stage I, 11 cases in stage II, and 6 cases in stage IIIA. In the control group, there were 7 cases in stage I, 14 cases in stage II, and 9 cases in stage IIIA.	Experimental group (treatment group): Starting from the third day after the operation, the patient took 4 tablets of Zilongjin orally every day, 3 times a day, for 12 consecutive days. In addition to taking Zilongjin Tablets, the patient also received the same nutritional support treatment as the control group members. Blood samples were collected from the patients before the operation, on the second day after the operation and on the 14th day after the operation, and indicators such as immunoglobulin and T lymphocyte subsets were detected.	30	Experimental group (treatment group): Preoperative: Proportion of CD3+ cells: 57.31 ± 14.01%; Proportion of CD4+ cells: 35.30 ± 13.46%; Proportion of CD8+ cells: 35.83 ± 11.27%; CD4+/CD8+ ratio: 1.15 ± 0.33; Proportion of NK cells: 8.82 ± 3.53%; Day 2 after the operation: Proportion of CD3+ cells: 50.22 ± 12.34 % (significant decrease *); CD4+ cell ratio: 27.52 ± 11.34 % (significantly decreased); Proportion of CD8+ cells: 35.36 ± 9.51%; CD4+/CD8+ ratio: 0.92 ± 0.30 (significant decrease *); Proportion of NK cells: 7.37 ± 3.98%; Day 14 after the operation: Proportion of CD3+ cells: 57.71 ± 13.67 % (significantly increased *); CD4+ cell ratio: 34.25 ± 13.62 %; Proportion of CD8+ cells: 31.04 ± 10.16 %; CD4+/CD8+ ratio: 1.17 ± 0.35 (significantly increased **); NK cell ratio: 8.70 ± 3.62 (significantly increased **)	Control group: Starting from the third day after the operation, the patient only received conventional nutritional support treatment and did not take Zilongjin Tablets. Blood samples were collected from the patients before the operation, on the second day after the operation and on the 14th day after the operation, and indicators such as immunoglobulin and T lymphocyte subsets were detected.	30

Influence of psychological intervention on pain and immune functions of patients receiving lung cancer surgery	–	Zhao, X.	2016	–	Experimental group: 28 males and 34 females, totaling 62 people. Control group: 38 men, 24 women, totaling 62 people	[illegible]	124	Lung cancer, regardless of stage or size	[illegible]	62	[illegible]	[illegible]	62
Lymphocyte recovery after breast cancer treatment and mindfulness-based stress reduction (MBSR) therapy	–	Lengacher, C. A.	2013	–	female	[illegible]	82	[illegible]	In the MBSR intervention group, there was no detailed intervention	[illegible]	[illegible]	[illegible]	[illegible]
Fast track surgery improves postoperative clinical recovery and cellular and humoral immunity after esophagectomy for esophageal cancer	–	Chen, L.	2016	ChiCTR-TRC-13003813	200 people (7.6%), 67 of whom (2.4%) were female	[illegible]	200	[illegible]	[illegible]	[illegible]	[illegible]	[illegible]	[illegible]
Effect of hypothermia prevention in patients undergoing gastrointestinal cancer surgery	–	Zhao, X.	2020	–	[illegible]	[illegible]	80	[illegible]	Intraoperative heat preservation	[illegible]	[illegible]	control	[illegible]
Effect of selective lymph node dissection on immune function in patients with T1 stage non-small cell lung cancer: A randomized controlled trial	–	Zhao, J. L.	2021	[illegible]	[illegible]	[illegible]	79	[illegible]	Selective lymph node dissection	[illegible]	[illegible]	Systematic lymph node dissection	[illegible]
Regulatory effect of mannatide peptide on postoperative immune function in patients with malignant thoracic tumor	–	Chen, X. Y.	2017	–	[illegible]	[illegible]	114	[illegible]	[illegible]	[illegible]	[illegible]	[illegible]	[illegible]
Effect of perioperative intestinal probiotics on intestinal flora and immune function in patients with colorectal cancer	–	Zhu, D.	2012	–	[illegible]	[illegible]	60	[illegible]	[illegible]	[illegible]	[illegible]	[illegible]	[illegible]
Preventive effect of ulinastatin on postoperative complications, immune suppression, and/or outcome in esophagectomy patients	–	Zhang, L.	2013	–	[illegible]	[illegible]	80	[illegible]	[illegible]	[illegible]	[illegible]	[illegible]	[illegible]
Effects of perioperative cimetidine administration on peripheral blood lymphocytes and tumor infiltrating lymphocytes in patients with gastrointestinal cancer: results of a randomized controlled clinical trial	–	Li, Y.	2005	–	[illegible]	[illegible]	43	[illegible]	[illegible]	[illegible]	[illegible]	[illegible]	[illegible]
